# A comparative genomic and phenotypic study of Vibrio cholerae model strains using hybrid sequencing

**DOI:** 10.1099/mic.0.001502

**Published:** 2024-09-23

**Authors:** Øyvind M. Lorentzen, Christina Bleis, Sören Abel

**Affiliations:** 1 Department of Pharmacy, UiT The Arctic University of Norway, Tromsø, Norway; 2 Division of Infection Control, Norwegian Institute of Public Health, Oslo, Norway

**Keywords:** biofilm, c-di-GMP signalling, hybrid sequencing, motility, QS, *V. cholerae*

## Abstract

Next-generation sequencing methods have become essential for studying bacterial biology and pathogenesis, often depending on high-quality, closed genomes. In this study, we utilized a hybrid sequencing approach to assemble the genome of C6706, a widely used *Vibrio cholerae* model strain. We present a manually curated annotation of the genome, enhancing user accessibility by linking each coding sequence to its counterpart in N16961, the first sequenced *V. cholerae* isolate and a commonly used reference genome. Comparative genomic analysis between *V. cholerae* C6706 and N16961 uncovered multiple genetic differences in genes associated with key biological functions. To determine whether these genetic variations result in phenotypic differences, we compared several phenotypes relevant to *V. cholerae* pathogenicity like genetic stability, acid sensitivity, biofilm formation and motility. Notably, *V. cholerae* N16961 exhibited greater motility and reduced biofilm formation compared to * V. cholerae* C6706. These phenotypic differences appear to be mediated by variations in quorum sensing and cyclic di-GMP signalling pathways between the strains. This study provides valuable insights into the regulation of biofilm formation and motility in *V. cholerae*.

**Impact Statement**

This study utilizes hybrid sequencing and manually curated annotation to provide a high-quality genome of *V. cholerae* C6706 that serves as a user-friendly tool to study this commonly used model strain. We uncover genotypic and phenotypic variance compared to *V. cholerae* N16961, which is widely used as a reference genome. Notably, distinct biofilm formation and motility patterns between the strains appear linked to differences in quorum sensing and c-di-GMP signalling pathways.

## Introduction

*Vibrio cholerae* is the enteric pathogen that causes cholera, an acute diarrhoeal disease that can spread explosively and cause devastating outbreaks [1]. Annually, approximately 3 million people are infected, and 95 000 deaths are associated with the disease [2], making this pathogen a serious global health threat.

So far, the world has experienced seven cholera pandemics, spreading the pathogen globally [1,3]. The most commonly studied strains today all originate from the seventh pandemic and belong to the O1 serotype, biotype El Tor. The El Tor biotype is the currently dominating *V. cholerae* variant, responsible for the ongoing seventh pandemic [1,4]. Among them are *V. cholerae* N16961, C6706, E7946, A1552 and 2010EL-1786 [5,9]. Even though they belong to the seventh pandemic, they differ in origin and belong to different transmission waves within the seventh pandemic [4]. *V. cholerae* C6706 and A1552 were isolated from an outbreak in South America in the 1990s, while *V. cholerae* 2010EL-1786 stems from the 2010 Haiti cholera outbreak [5,9]. N16961 and E7946 were isolated in Asia in the 1970s. N16961 was the first sequenced *V. cholerae* isolate and has been used as a reference genome for the seventh pandemic *V. cholerae* El Tor strains [7]. While other *V. cholerae* variants have been sequenced more recently [9,12], including a *V. cholerae* C6706 strain [9,12], the original N16961 annotation is still widely used [13,19].

*V. cholerae* survives in the aquatic environment year-round in endemic regions and infects humans through contaminated water and food [1,20]. Throughout its life cycle, the pathogen alternates between the aquatic environment and the human host. To successfully survive these transitions, *V. cholerae* depends on multiple cellular processes [1,20, 21]. Biofilm formation plays an important role in the life cycle of *V. cholerae*. Growth in biofilms facilitates survival in the aquatic environment and increases the acid tolerance and infectivity of *V. cholerae* [21,25]. Two of the key regulators of biofilm formation in *V. cholerae* are quorum sensing (QS) and the cyclic diguanylate (c-di-GMP) signalling system. QS is a key bacterial cell–cell communication system that governs collective behaviour in bacteria in response to cell population density and composition [19,26]. Low cell density (LCD) induces biofilm formation through phosphorylation of the response regulator LuxO, which in turn simultaneously represses HapR and activates AphA, which are high-cell- and low-cell-density master QS regulators [19,27,29]. HapR and AphA repress and stimulate the expression of biofilm genes, respectively. In contrast, high cell density leads to dephosphorylation of LuxO, which results in the repression of AphA and the expression of HapR, thereby inhibiting biofilm formation [19,27,29]. QS states can differ between different seventh pandemic *V. cholerae* strains. For instance, the N16961 carries a frameshift mutation in HapR, which disrupts the QS signalling pathway and locks the strain into the state it assumes under LCD conditions [7,30, 31]. One of the mechanisms through which QS represses biofilm formation in *V. cholerae* involves the regulation of c-di-GMP-metabolizing enzymes [28,32,34]. C-di-GMP is a conserved nucleotide-based second messenger that regulates the biofilm formation and motility in bacteria [35,37]. In short, c-di-GMP upregulates the biofilm formation and inhibits motility. C-di-GMP regulates the biofilm formation and motility through multiple regulatory mechanisms, which include flagellum synthesis and function, production of pilis and adhesins and secretion of extracellular polymeric substances [35,37]. Strikingly, many Gram-negative pathogens with complex life cycles also boast complex c-di-GMP signalling systems [37]. In particular, *V. cholerae* possess more than 60 putative c-di-GMP-metabolizing proteins [35,36].

One of the first barriers *V. cholerae* encounters in the human host is the acidic environment of the stomach [20,38]. To overcome acidic conditions, *V. cholerae* employs a response called the ‘acid tolerance response’ (ATR) [39]. This is a cascade of physiological responses that enable the bacterium to counteract the damaging effects of acid stress. One extensively studied component of ATR is the Cad system (*CadABC*), which maintains the intracellular pH balance by converting H^+^ and lysine to cadaverine [20,39,41]. After passage through the stomach, the pathogen enters and colonizes the small intestine to cause the characteristic symptoms of the disease [1,20]. To summarize, the pathogenicity of *V. cholerae* depends on multiple physiological processes including acid tolerance, motility, chemotaxis, biofilm formation and QS [1,19,21].

In this study, we *de novo* assembled the genome of *V. cholerae* C6706 using short- and long-read next-generation sequencing (NGS) technologies. As an effort to improve upon the prior version of the C6706 genome, we also manually curated the genome annotation and linked all ORFs with the corresponding ORF in the N16961 genome. We thereby created an accessible genome with a link to the N16961 reference genome. Finally, we conducted a genetic and phenotypic comparison of *V. cholerae* C6706 and N16961 to reveal the differences between the two strains, thus contributing to the understanding of *V. cholerae* biology.

## Methods

### Strains, growth media and oligonucleotides

All bacterial strains used in this work are listed in Table 1. Unless otherwise noted, cultures were grown from single colonies in Lysogeny Broth (Miller) (LB) media (10 g l^−1^ of tryptone, 10 g l^−1^ of sodium chloride and 5 g l^−1^ of yeast extract) at 37 °C with shaking at 700 r.p.m. Media were supplemented with carbenicillin (50 µg ml^−1^) when *V. cholerae* strains carrying plasmids were grown to maintain the plasmid. For the acid killing assay, the pH of the medium was adjusted to pH 4.6 with a 0.1 M solution of HCl. All oligonucleotides were synthesized by Sigma-Aldrich and purified to remove salts.

**Table 1.** Strains and plasmids used in this study

Strain no.	Strain background	Vector	Insert	References	
3479	*V. cholerae* C6706 QS deficient	None	None	[6]	
1810	*V. cholerae* N16961	None	None	[7]	
3802	*V. cholerae* C6706 QS proficient	None	None	[30]	
2832	*V. cholerae* C6706 QS deficient	pANG01	None	This study	
3002	*V. cholerae* C6706 QS deficient	pOML27	VC1295-mRuby2	This study	
3867	*V. cholerae* N16961	pOML27	VC1295-mRuby2	This study	
3868	*V. cholerae* N16961	pANG01	None	This study	
2844	*E. coli* DH5αpir	None	None	[85]	
2988	*E. coli* DH5αpir	pOML27	VC1295-mRuby2	This study	
2831	*E. coli* DH5αpir	pANG01	None	This study	

### Genomic DNA extraction

One millilitre of liquid culture of *V. cholerae* in the exponential phase was pelleted by centrifugation and resuspended in 50 µl of double-distilled sterile H_2_O. Afterwards, 600 µl of lysis buffer (SDS 2% and 0.1 M EDTA) was added, and the solution was incubated for 5 min at 80 °C to lyse the cells. After incubation, the solution was left to cool down to room temperature. Three microlitres of RNaseA were then added, and the solution was incubated for 60 min at 37 °C. Afterwards, the solution was cooled down to room temperature again before 200 µl protein precipitation solution (7.5 M ammonium acetate) was added. The solution was incubated on ice for 5 min followed by centrifugation to precipitate proteins. The DNA-containing supernatant was recovered, while the remaining pellet was discarded. Genomic DNA (gDNA) was then precipitated by adding 600 µl of isopropanol and gentle mixing. The precipitated gDNA was isolated from the tube with a glass pipette and washed in 600 µl of 70% EtOH. Lastly, the DNA was centrifuged one more time, and the supernatant was discarded. The remaining DNA pellet was carefully dried at room temperature for 30 min and dissolved overnight in Milli-Q filtered water.

### Whole-genome data acquisition, assembly and annotation

The sequencing service was provided by the Norwegian Sequencing Centre (www.sequencing.uio.no), a Norwegian national technology platform hosted by the University of Oslo and supported by the ‘Functional Genomics’ and ‘Infrastructure’ programmes of the Research Council of Norway and the Southeastern Regional Health Authorities.

To create the paired-end fragment libraries for Illumina NGS, gDNA was sheared on a Covaris E220 instrument aiming for a 350 bp fragment size. After fragmentation, the sample was transferred to a 96 plate for a half-volume Kapa Hyper library preparation kit (Roche). Unique dual indexing (Illumina UD adaptor plate, Illumina) was used in the ligation reaction, followed by a clean-up and one round of PCR (four cycles) to boost the library amount. The final library was cleaned twice to get rid of any leftover adapters/dimers. The quality was checked on a Fragment Analyzer instrument using a standard NGS kit (AATI). The library was sequenced on an HiSeq 4000 instrument (150 bp paired end reads) and quantified using quantitative PCR (qPCR) (Kapa Library Quantification Kit, Kapa/Roche).

To conduct PacBio SMRT sequencing, gDNA was sheared to 12 kb fragments using g-tubes from Covaris. The library was prepared using Pacific Biosciences protocol for SMRTbell with PacBio Barcoded Adapters for Multiplex SMRT Sequencing. The sample was pooled together with ten other samples in roughly equimolar ratios. The final library was size selected using 0.45× AMPure PB beads. The library was sequenced on a Pacific Biosciences Sequel instrument using Sequel Polymerase v3.0, SMRT cells v3 LR and Sequencing chemistry v3.0. Loading was performed by diffusion. Sequencing was performed on one SMRT cell. Following sequencing, reads were demultiplexed using the barcoding pipeline on SMRT Link with 26 as the minimum barcode score. Finally, HGAP4 assembly was performed using the Assembly (HGAP4) pipeline on SMRT Link. Assembly was run using 4 Mb as the expected genome size.

The final genome was assembled with the hybrid assembler Unicycler with both short and long reads as input sequences [42]. The genome was automatically annotated with the Rapid Annotation and Subsystem Technology (RAST) web tool hosted on PATRIC [43,44]. Gene annotations in strain C6706 were linked to the previously described *V. cholerae* strains by conducting a blast search with every identified coding sequence (CDS) in C6706 against known genomic features in a selection of *V. cholerae* strains [45]. To improve upon the automated annotation of the C6706 genome, all CDS annotated as hypothetical proteins by RAST were subjected to an additional protein blast search to identify the gene function through comparison of homologues with an *E*-value <0.05 in other bacterial strains.

### Attachment assay

The attachment was used as a proxy for the biofilm formation and determined using a crystal violet (CV) attachment assay as described before but in 24-well plates instead of 96-well plates [46]. Briefly, 2 ml LB medium was inoculated 1 : 100 with an overnight culture of *V. cholerae* and grown statically for 24 h. After 24 h, the bacterial culture was removed, and the plate was gently submerged in filtered water thrice to remove non-adherent cells. Afterwards, plates were dried for 1 h at 55 °C. To quantify the biofilm formation, wells were stained with 2 ml of 0.1% CV for 10 min. After staining, the CV solution was removed, and the plates were again submerged in filtered water to remove non-adherent dye and left to dry at room temperature. The CV was dissolved from the stained biomass with 70% ethanol. Finally, the dissolved dye concentration was quantified by measuring the absorbance at 595 nm in a Spark multimode plate reader (Tecan).

### Motility on semi-solid agar plates

Colony size on semi-solid agar plates was used as a proxy for motility and was assessed as previously described [47]. Briefly, semi-solid agar plates were made with 60 ml of LB media containing 0.3% agarose in 140-mm-diameter Petri dishes. One microlitre of cells grown to the exponential phase in LB medium was stabbed into the surface of the swarmer plates with a sterile inoculation loop. The plates were incubated at 37 °C for 12 h. Afterwards, plates were imaged. To quantify the motility, the images were imported into Fiji, and the diameter of each individual swarm was measured in Fiji with the line and measure function [48].

### Variation analysis and genome comparison

The variation analysis was performed with the variation analysis tool hosted by PATRIC [44]. Mutations were called using standard settings with BWA-mem-strict and FreeBayes as aligner and SNP caller, respectively. Mutations called in homopolymeric regions were omitted. In addition, mutations called when comparing our short read sequences of *V. cholerae* C6706 against the our assembled C6706 genome (BioProject accession PRJNA1109855) (Table S1) were omitted. Genome comparison was conducted with progressiveMauve [49].

### Fluctuation assays

Wild-type *V. cholerae* C6706 and N16961 were grown overnight. A dilution series of the overnight cultures were created and plated onto an LB plate with or without 50 µg ml^−1^ rifampicin. After overnight growth at 37 °C, the rifampicin-resistant and the total number (resistant and susceptible) of colonies were counted. The mutation rate was calculated with the FALCOR web application using the Ma–Sandri–Sarkar maximum likelihood estimator (MSS-MLE) method [50]. The Student’s t-test was used to determine the significance between the observed number of mutational events in C6706 and N16961 based on nine biological replicates [51].

### Acid killing assay

Survival in acidic media was determined as previously described [39]. Briefly, two 100 ml LB cultures were inoculated 1 : 100 with a starter culture of *V. cholerae* C6706 and N16961, respectively. The two cultures were grown to the exponential phase and subsequently diluted to OD_600_ of 0.0125. These were used as inoculum into acidic media (LB adjusted to pH 4.6 with 0.1 M HCl). Samples were taken out at time points 0 min, 25 min, 40 min and 60 min, and c.f.u. values were enumerated by plating on LB agar. The experiment was performed in biological triplicates.

### Bacterial fitness measurements

To start the growth curve experiments, overnight cultures were diluted 1 : 100 into 300 µl LB medium and pipetted into a 100-well honeycomb plates (Oy Growth Curves Ab Ltd., Finland). Growth curves were recorded by measuring OD at 600 nm (OD_600_) every 4 min for 24 h at 37 °C with continuous shaking in a Bioscreen C instrument (Oy Growth Curves Ab Ltd., Finland). Bacterial fitness was quantified as the area under the curve (AUC) in the growth curves with the flux package in R [52]. Each biological replicate is always based on three technical replicates.

### Cloning of VC1295 for the expression and fluorescent microscopy

All primers used in this study are listed in Table 2. *VC1295* was amplified by PCR with its native promotor from gDNA extracted from *V. cholerae* N16961 using primers #153 and #154. The PCR was conducted with Phusion polymerase (New England Biolabs). Afterwards, *VC1295* with its native promotor was cloned onto the pENTRY vector pMaRo1 through a Gibson assembly reaction, which was conducted at 50 °C for 60 min [53]. The correct sequence was confirmed by Sanger sequencing by Macrogen Europe using primers #3 and #4.

**Table 2.** Primers used in this study

No.	Name	Sequence (5′ to 3′)	Ref.
3	P0003_pEntry_F	gatctcgggccccaaataat	[54]
4	P0004_pEntry_R	gcagctggatggcaaataat	[54]This study
153	P0153_VC1295_F	TTTTATAATGCCAACTTTGTACAAAAAAGCAGGCT *gtatcttaacagtattccttgataca*	This study
154	P0154_VC1295_R	TCTTATAATGCCAACTTTGTACAAGAAAGCTGGGTtgcggcatctttaaagtgtgct	This study
159	P0159_pANG_Ins_F	cagtgccaacatagtaagccag	This study
160	P0160_pANG_Ins_R	gaagcatttatcagggttattgtctc	This study

The final vector construct carrying *VC1295-mRuby2* under the control of its native promotor was assembled by a Gateway cloning reaction between the pENTRY vector containing *VC1295* and a pDESTINATION vector carrying *mRuby2* [54,55]. The correct assembly was confirmed by PCR using primers #159 and #160. This vector was then conjugated into *V. cholerae* via the intermediate strain *Escherichia coli* SM10(λpir) [56].

*V. cholerae* strain carrying a plasmid expressing *VC1295*-mRuby2 was grown to the exponential phase. Upon reaching the exponential phase, 2 µl of the suspension was spotted on a 1% agarose patched and imaged with a DeltaVision Elite (GE Healthcare) inverted microscope with a DeltaVision CMOS camera and a UPlanFLN 100× PH NA 1.30 phase-contrast objective (Olympus). The images were processed with softWoRx (GE Healthcare), Fiji and Photoshop CS6 (Adobe) to find a focused slide, crop the area of interest and adjust the levels [48].

## Results

### Building a circularized *V. cholerae* C6706 genome

**Fig. 1.**
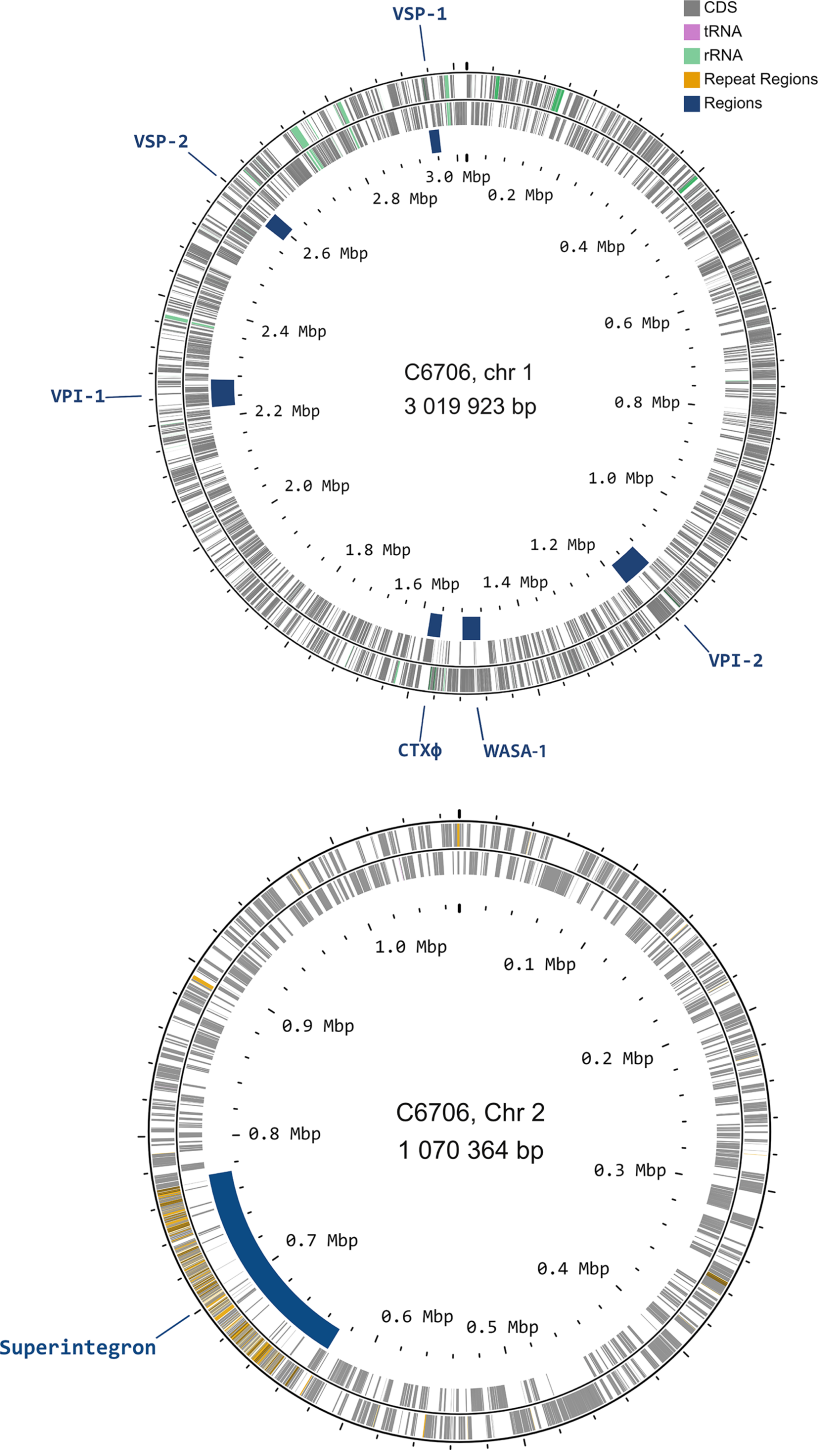
Maps of chromosome 1 and 2 of *V. cholerae* C6706. Each grey bar represents a coding sequence (CDS), pink bar represents tRNAs, green bars represent rRNAs and orange bars represent repeat regions. Important mobile genetic elements (“regions”) are underlined in blue. Maps were created with CGView [86,87].

We created a closed circularized genome for *V. cholerae* C6706 with a hybrid sequencing approach that combines both long- and short-read sequencing technology (Fig. 1) [42]. Long-read sequencing with PacBio sequencing yielded 504 131 subreads with a mean length of 6801 bps generating a total of 1.89×10^9^ bases. The assembly yielded two contigs totalling 4 090 295 bp, with an average read depth of 444×. Short-read sequencing using Illumina technology generated a total of 15 841 698 reads, with an average length of 151 bps, resulting in an average read depth of 585×. The resulting genome consists of two closed, circular chromosomes of 3 019 923 and 1 070 364 bps, respectively. Genome quality evaluation hosted by PATRIC [44] confirmed the assembled genome’s completeness at 100%, with a minimal contamination of 0.6%. Additionally, the genome sequence is of high quality, with coarse and fine consistency values reaching 99.9 and 99.5%, respectively (Table 3) [57]. Automatic annotation with the RAST, hosted by PATRIC, yielded 3 788 CDS [43,44]. To increase the usability, every CDS in the C6706 genome has also been linked to the corresponding CDS in *V. cholerae* N16961 by searching for homologues by blast (Table S2).

**Table 3.** Sequencing and genome assembly metrics and genome quality control

PacBio sequencing	
Subreads	504 131
Subread mean length (bp)	6801
Total reads (bp)	1.89×10^9^
Assembly	
2 contigs total (bp)	4 090 295
Average read depth	444×
Illumina sequencing	
Reads total	15 841 698
Mean length (bp)	151
Average read depth	585×
RAST	
Completeness (%)	100
Coarse consistency (%)	99.9
Fine consistency (%)	99.5
Contamination (%)	0.6

### *V. cholerae* C6706 carries multiple genetic changes compared to N16961

The *V. cholerae* El Tor variant C6706 is a commonly used strain by researchers and represents the wild-type in many studies [13,17, 18, 58, 59]. For a long time, only the genome of *V. cholerae* N16961, another El Tor isolate, was available [7], and researchers have used and are still using it as the de facto reference genome [13,18] despite the potential differences between the El Tor variants [4,9, 10]. To identify the genetic differences between *V. cholerae* C6706 and N16961, we conducted a genome comparison and a variant analysis. The paired-read library of Illumina short-read sequences from our hybrid sequencing of *V. cholerae* C6706 was used as input. The updated closed genome of *V. cholerae* N16961 (GenBank accession numbers: LT906614 and LT906615; PATRIC Genome ID: 243277.254) was used as a reference genome. This identified 76 differences, which included 6 deletions, 3 insertions and 67 point mutations. Of the 67 point mutations, there were 44 nonsynonymous mutations and 23 synonymous mutations (Table S3). A genome comparison of *V. cholerae* C6706 and N16961 showcased that C6706 also contained the West African–South American (WASA)-1 prophage specific to the WASA clade of *V. cholerae* isolates, which are not present in N16961 [4,60]. Similar to what has been observed in other *V. cholerae* strains, chromosome 1 contained a large inversion (Fig. S1, available in the online version of this article) [61,62]. A comparison of the previously sequenced *V. cholerae* C6706 showcased that the inversion varied between assemblies (Fig. S2). Finally, C6706 contained a different variant of the *Vibrio* seventh pandemic island II (VSP-2), which differs from N16961 in the region encompassing VC0511-VC0515 [4,63, 64]. Overall, the genomes of C6706 and N16961 are very similar except for the two abovementioned mobile genetic elements and inversion in chromosome 1.

Several of the identified mutations in C6706 were located in genes previously linked to important biological functions, such as biofilm formation, motility, QS, genome stability and repair and acid tolerance (Table 4). Among the mutated genes was the previously published point mutation (Gly333Ser) in LuxO, which induces a deficiency in QS that locks *V. cholerae* into the same state; it assumes under LCD conditions [30]. We also found multiple mutations in c-di-GMP-metabolizing genes, potentially affecting the c-di-GMP turnover of *V. cholerae* C6706 relative to N16961. This included a frameshift mutation in VC1295, an HD-GYP-domain protein predicted to degrade c-di-GMP [65]. In addition, we identified mutations in genes coding for proteins important for flagellar function and chemotaxis. Lastly, we identified mutations in CadB, an enzyme crucial for acid adaptation in several enteric pathogens and RecA, which is essential for the global response to DNA damage (SOS response), DNA repair and mutagenicity in bacteria [39,41, 66,68].

**Table 4.** Selected genes with changes from N16961 to C6706

Gene accession no. N16961	Gene accession no. C6706	Nucleotide change	aa change	Gene name	Function	Cellular process
VC0280	VCC2568	C686T	Thr229Ile	*cadB*	Lysine/cadaverine antiporter membrane protein CadB	Acid adaptation
VC0543	VCC2308	A914G	Tyr305Cys	*recA*	RecA protein	Stress response
VC0543	VCC2271	217_218insT*	Val74fs†	*hapR*	QS regulator of virulence	QS
VC0653	VCC2204	T1664C	Val555Ala	*rocS*	Diguanylate cyclase/phosphodiesterase	c-di-GMP signalling
VC1021	VCC1859	G997A	Gly333Ser	*luxO*	LuxO global QS regulator	QS
VC1295	VCC1597/1598	1153delT‡	Phe385fs	n/a	c-di-GMP phosphodiesterase (HD-GYP domain)	c-di-GMP signalling
VC1399	VCC1499	837_838insA	Gly279_ Lys280fs	*cheR*	Chemotaxis protein methyltransferase CheR	Motility/chemotaxis
VC1653	VCC1185	C1675T	His559Tyr	*vieS*	Response regulator VieS	c-di-GMP signalling
VC1967	VCC0880	A277G	Thr93Ala	n/a	Methyl-accepting chemotaxis sensor/transducer protein	Motility/chemotaxis
VC2191	VCC0672	C863T	Pro288Leu	*flgK*	Flagellar hook-associated protein FlgK	Motility/chemotaxis
VC2208	VCC0655	G283A	Ala95Thr	*flgT*	Flagellar protein FlgT	Motility/chemotaxis
VC2338	VCC0536	C1237T	Leu413Phe	*lacZ*	Beta-galactosidase	Metabolism
VCA0557	VCCA0844	A926G	Asp309Gly	n/a	Diguanylate cyclase	c-di-GMP signalling
VCA0931	VCCA0133	G1207A	Ala403Thr	n/a	c-di-GMP phosphodiesterase (HD-GYP domain)	c-di-GMP signalling
VCA1084	VCCA0275	G339A	Met113Ile	*lapB*	Type I secretion system ATPase, LssB family LapB	Type II secretion systems

* “* *‘ins’ indicates an insertion between the two given positions within a gene.

# †‘fs’ indicates a frame shift after the first amino acidaa that is affected by the change.

$ ‡‘del’ indicates a deletion of the indicated base at the indicated position.

Our genetic comparison of *V. cholerae* C6706 and N16961 identified a selection of mutations likely affecting the physiology and adaptability of these strains. To understand the role of these mutations, we employed phenotypic assays to accurately quantify differences in key bacterial phenotypes between *V. cholerae* C6706 and N16961.

### Mutation rate is increased in *V. cholerae* C6706

**Fig. 2.**
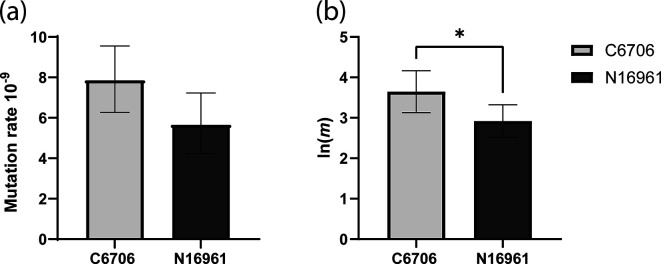
Comparison of mutation rate and the natural logarithm of number of mutations per culture [ln(*m*)] of *V. cholerae* C6706 and N16961 measured with a rifampicin fluctuation assay [50]. (**a**) Mutation rate of *V. cholerae* C6706 and N16961. (**b**) Ln(*m)* of *V. cholerae* C6706 and N16961. Bars depict the means based on nine biological replicates. Error bars represent the 95% confidence intervals. Statistical significance was tested using an unpaired two-tailed T-test, *, *p*<0.05.

The *recA* gene codes for a protein essential for DNA repair and has been shown to affect the mutagenicity of bacteria [66,67, 69]. The A914G substitution identified in C6706 is a missense mutation that results in a tyrosine-to-cysteine change at position 305 in the C-terminal domain of RecA [70]. To assess whether this mutation affects the mutation rate in *V. cholerae* C6706, we quantified the mutation rate and number of mutations per culture (*m*) with a rifampicin fluctuation assay [71]. The mutation rate and *m* were calculated using the FALCOR web application, employing the MSS-MLE method [49]. The mutation rate was higher in *V. cholerae* C6706 (7.85×10^−9^) compared to N16961 (5.65×10^−9^) (Fig. 2a), and we observed a significant increase in mutations per culture (*m*) in *V. cholerae* C6706 (39.3) compared to N16961 (18.2) (*P*=0.0236 in two-tailed t-test comparing the natural logarithm of *m* of C6706 and N1691) (Fig. 2b). We did not identify any additional mutations in genes predicted to be involved in genome stability and repair. This suggests that the observed difference in mutation rates could stem from functional differences in the RecA variants.

### *V. cholerae* C6706 and N16961 have similar tolerance to acidic conditions

**Fig. 3.**
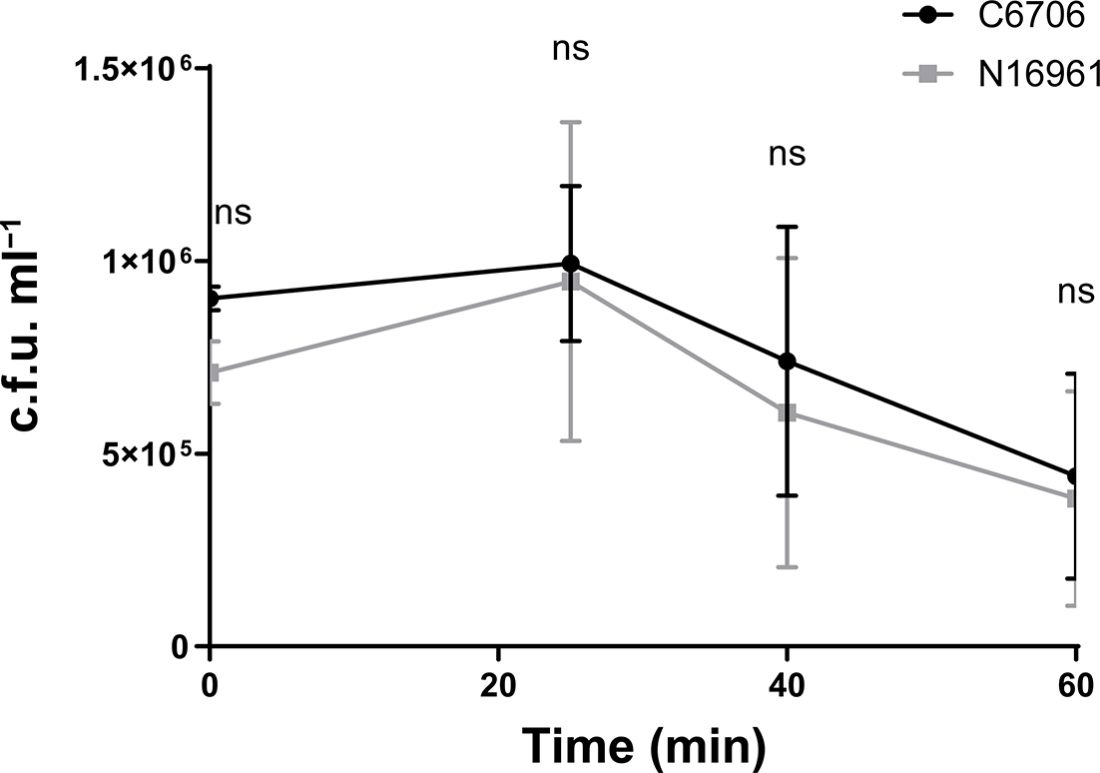
Both *V. cholerae* strains, C6706 and N16961, are equally sensitive to acid. C.f.u. ml^−1^ measurements over time of *V. cholerae* C6706 and N16961 exposed to pH 4.6 in LB medium. The dots represent the mean of three biologically independent replicates and error bars indicate the standard deviation. Statistical significance was tested using a two-tailed T-test for each individual time point; non-significant (ns), *p*>0.05.

To understand the impact of the point mutation in *cadB* (Thr229Ile), we compared the acid tolerance of *V. cholerae* C6706 and N16961. Both strains were exposed to a pH of 4.6 in LB medium, and c.f.u. values were enumerated over time (Fig. 3). The two strains did not grow in pH 4.6. Instead, c.f.u. values declined over time, indicating cell death. There was no significant survival difference between *V. cholerae* C6706 and N16961 in acid at any time point (*P*=0.4–0.9, two-tailed t-test), indicating that the observed mutation in *cadB* does not significantly affect the acid tolerance of *V. cholerae* C6706.

### Biofilm formation and motility differ between *V. cholerae* C6706 and N16961

*V. cholerae* C6706 harboured multiple genetic changes in genes predicted to regulate biofilm formation and motility, including several c-di-GMP-associated genes, *luxO*, and multiple proteins involved in flagellar function and chemotaxis (Table 4). In bacteria, biofilm formation and motility are often inversely regulated, where increasing the biofilm formation leads to decreased motility and vice versa [35,36, 72, 73]. To investigate if the sum of these genetic changes affected biofilm formation and motility in *V. cholerae* C6706 and N16961, we quantified these phenotypes. As already stated, N16961 contains a non-functional *hapR* due to a frameshift mutation that locks it in an LCD QS state, independent of the actual cell density [7,30, 31]. This defective QS is known to affect biofilm formation and motility [22,28]. The strain of *V. cholerae* C6706 sequenced in this study is locked in the same QS state, but due to a different mutation in *luxO* [30]. To compare N16961 to the naïve, QS-proficient C6706, we also included a variant with harbouring wild-type *luxO* [22]. We conducted a variant analysis to identify the genetic differences between QS-proficient C6706 (GenBank accession numbers: CP064350, CP064351; PATRIC Genome ID: 948564.8) sequenced by Weng *et al*. [11] and QS-deficient C6706 (BioProject accession PRJNA1109855) strains, which did not reveal any additional mutations other than the expected G997A in *luxO* (Table S4).

**Fig. 4.**
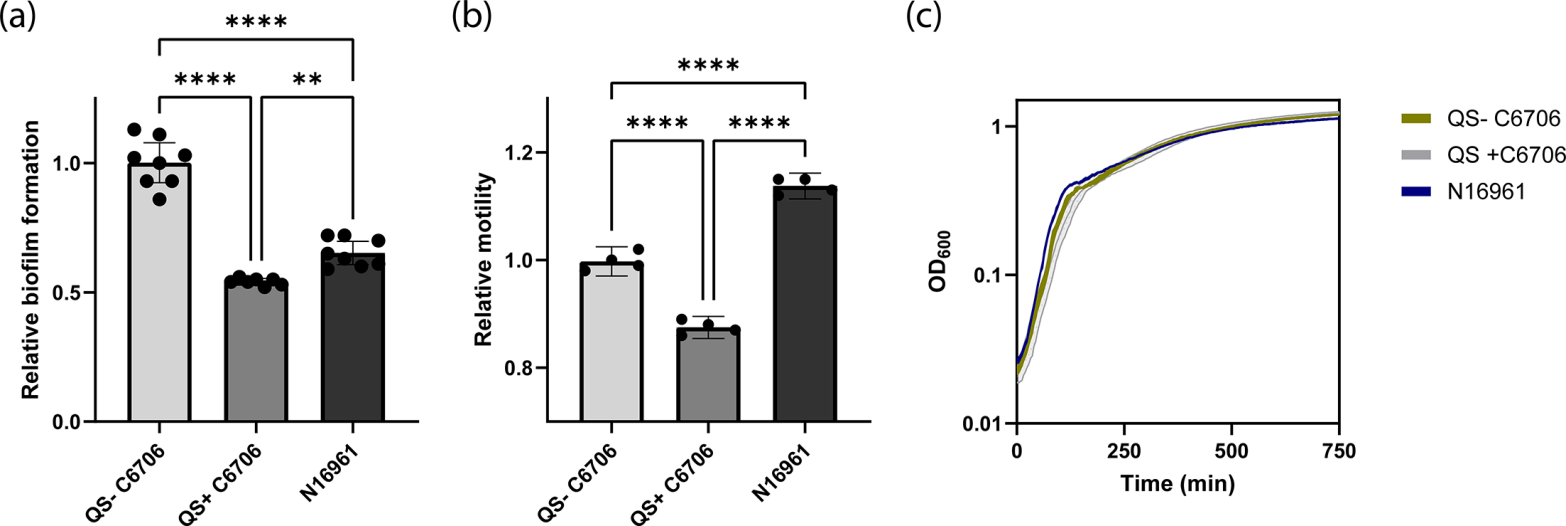
Comparison of the capacity to form biofilms, motility on semi-solid agar plates, and growth of the naturally QS-deficient N16961, a laboratory acquired QS deficient C6706 (QS- C6706), and a naturally QS proficient C6706 (QS+ C6706). (**a**) Relative biofilm formation normalized to QS- C6706 after static growth at 37 °C for 24 hours in 24-well plates. The bars represent the mean of eight (QS- C6706 and N16961) or seven (QS+ C6706) biological replicates and the error bars indicate the 95% confidence interval. Statistical significance was tested using a one-way ANOVA followed by Tukey’s multiple comparisons tests. **, *p*<0.01; ****, *p*<0.0001. (**b**) Relative motility normalized to QS- C6706 after growth on semi-solid agar for 12 hours. The bars represent the mean of four biological replicates and the error bars indicate the 95% confidence interval. Statistical significance was tested using a one-way ANOVA followed by Tukey’s multiple comparisons tests. ****, *p*<0.0001. (**c**) Growth curves of N16961, QS-deficient *V. cholerae* C6706, and QS-proficient C6706 measured as OD_600_ over time.

The QS-deficient variant of *V. cholerae* C6706 formed 85 and 53% more biofilm compared to QS-proficient variant of *V. cholerae* C6706 and QS-deficient N16961, respectively (Fig. 4a, one-way ANOVA, *P*<0.0001). In addition, N16961 formed 20% more biofilm compared to the QS-proficient variant of C6706 (Fig. 4a, one-way ANOVA, *P*<0.01). The QS-deficient *V. cholerae* C6706 exhibited 14% lower motility compared to *V. cholerae* N16961 (Fig. 4b, one-way ANOVA, *P*<0.0001). In contrast, QS-deficient C6706 exhibited a 12% increase in motility compared to QS-proficient C6706 (Fig. 4b, one-way ANOVA, *P*<0.0001). To ensure that the differences in biofilm formation and motility were not due to large variations in bacterial fitness, we recorded growth curves. Comparisons of these curves revealed small differences in the AUC. Although these differences were statistically significant, the resulting minor changes in relative fitness (QS-deficient C6706 : 1.00, N16961 : 0.96, QS-proficient C6706 : 1.03) are unlikely to account for the substantial differences observed in biofilm formation and motility (Fig. 4c, Table S5).

QS-deficient C6706 and N16961 had inverse behaviour when comparing biofilm formation and motility, where the strain with the highest level of biofilm formation had the lowest motility. In contrast, QS-proficient *V. cholerae* C6706 did not exhibit this pattern and had both the lowest biofilm formation and the lowest motility out of the three strains. The observed differences in biofilm formation and motility between the QS-proficient and QS-deficient variants of *V. cholerae* C6706 are likely due to the differences in QS state, as QS is known to impact biofilm formation and motility [22,28]. The same explanation could explain the differences between QS proficient and N16961. However, the QS-deficient variant of *V. cholerae* C6706 and N16961 are locked in the same LCD state. Therefore, the difference in biofilm formation and motility between these two strains is likely not mediated by differences in QS state, but caused by additional mutations (e.g. mutations in the c-di-GMP signalling system or flagellar protein).

### Altered c-di-GMP signalling and QS shape biofilm and motility in *V. cholerae* strains C6706 vs. N16961

**Fig. 5.**
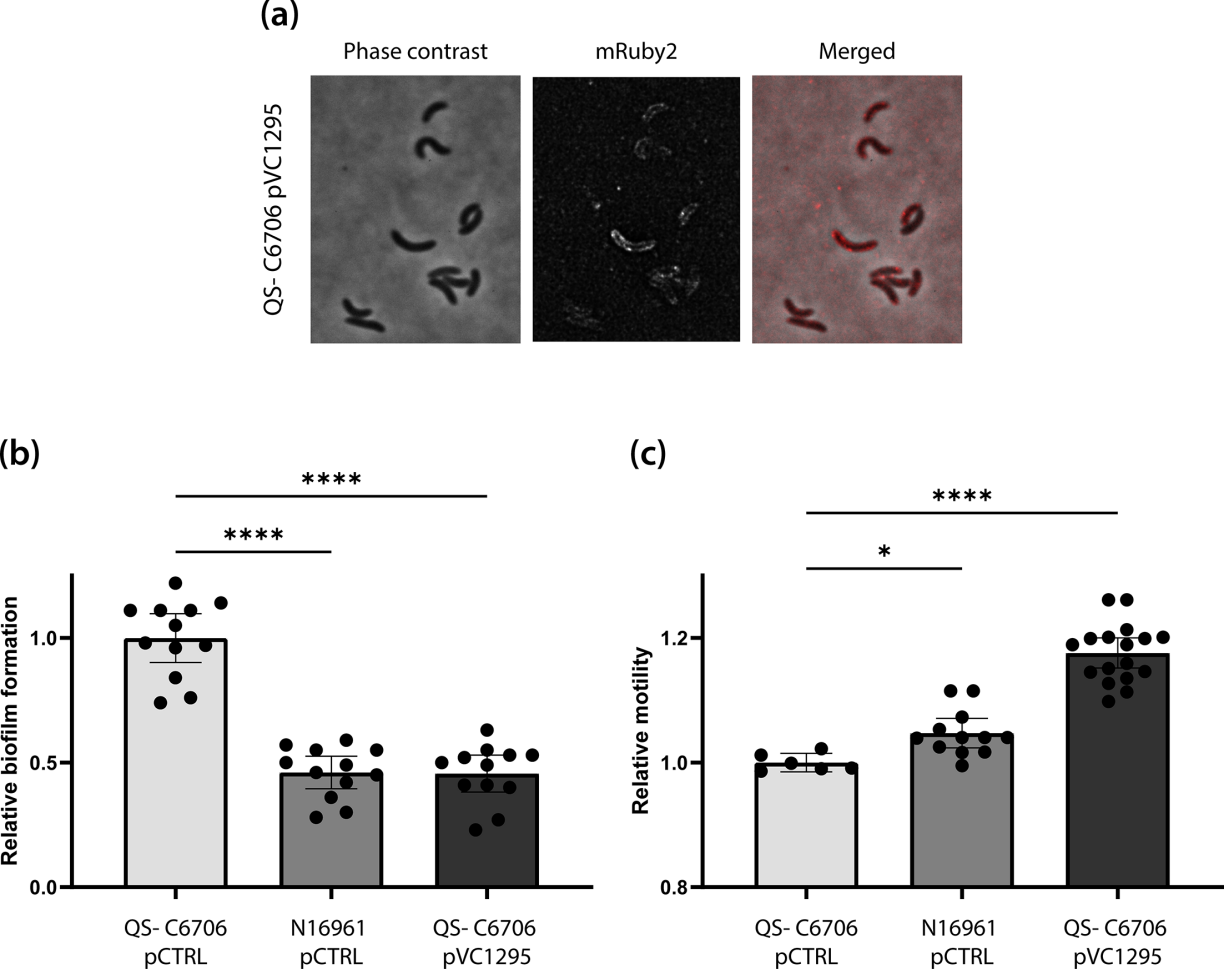
VC1295 is expressed in *V. cholerae* C6706, inhibits biofilm formation and increases motility. (**a**) Fluorescent images of *V. cholerae* C6706 expressing VC1295 fused to a C-terminal mRuby2 on a medium-copy number plasmid under the control of its native promoters in the exponential growth phase (QS− C6706 pVC1295). A phase contrast (PC), a corresponding fluorescence (mRuby2) image and an overlay of both images (merged) are shown. (**b**) Biofilm formation of QS− C6706 pCTRL, N16961 pCTRL and QS− C6706 pVC1295 normalized to QS− C6706 pCTRL after static growth at 37 °C for 24 h in 24-well plates (*n*=12). (**c**) Motility of the same strains test in (**b**) after growth on semi-solid agar at 37 °C for 12 h (*n* ≥ 6). The bars represent the mean and the error bars represent the 95% CI. Statistical significance was tested using a one-way ANOVA followed by the Dunnet multiple comparison correction. *P<0.05; ****P<0.0001.

A comparison of N16961, QS-deficient *V. cholerae* C6706 and QS-proficient C6706 indicated that QS was not the sole cause of the observed differences in biofilm formation and motility. We, therefore, wanted to investigate whether variations in the c-di-GMP signalling system, particularly the frameshift mutation in the putative c-di-GMP-degrading protein VC1295, contributed to the observed phenotypic differences. First, we investigated the expression of VC1295 under the control of its native promotor. In agreement with Koestler and Waters [74] and McKee *et al*. [65], fluorescence microscopy demonstrated that VC1295 was expressed and translated in *V. cholerae* C6706 (Fig. 5a). Furthermore, the protein was correctly translocated to its expected subcellular compartment in the cell membrane.

*V. cholerae* N16961 contains a functional copy of VC1295 and is naturally QS deficient. Therefore, we next investigated the biofilm formation and motility in N16961 harbouring an empty control vector (N16961 pCTRL) and QS-deficient *V. cholerae* C6706 expressing either a plasmid-born VC1295 (QS− C6706 p*VC1295*) or an empty control vector (QS− C6706 pCTRL). Comparing QS-deficient *V. cholerae* C6706 harbouring pVC1295 with N16961 harbouring pCTRL, the two strains displayed identical biofilm formation (Fig. 5b), while QS− C6706 p*VC1295* demonstrated a 12% increase in motility compared to N16961 pCTRL (Fig. 5c). This indicates that the plasmid-borne VC1295 can complement the frameshifted genomic VC1295. Therefore, VC1295 seemed to be expressed and enzymatically active under the tested biofilm and motility assay conditions, and the frameshift mutation in *VC1295* in *V. cholerae* C6706 seems to contribute to the differences in the biofilm formation and motility between C6706 and N16961.

## Discussion

In this study, we have assembled a high-quality, carefully annotated genome of *V. cholerae* C6706 and employed it to conduct a genetic and phenotypic comparison of *V. cholerae* C6706 and N16961. The assessment of genome quality in our assembled genome aligned well with the criteria suggested by Parello *et al*. [57] for defining a high-quality genome with a low contamination score (0.6%), a fine consistency score of 99.5% and a completeness score of 100%. A genetic comparison demonstrates how these strains, isolated from different outbreaks in the seventh pandemic, carry mutations that alter their physiology. Indeed, when comparing our assembled C6706 genome with the genome of N16961, we identified multiple mutations in genes with the potential to affect *V. cholerae* biology (Table 4). One set of striking differences was the multiple mutations in enzymes metabolizing c-di-GMP, a well-known regulator of biofilm and motility in bacteria [35,37]. In addition, we observed mutations in genes involved in acid stress, stress response and genome stability, flagellum biosynthesis and chemotaxis. The comparison of our genome to the previously assembled genomes of *V. cholerae* C6706 only identified the known laboratory-acquired mutation in *luxO* [11,30]. However, a large inversion in chromosome 1 had occurred (Fig. S1 and S2), which seems to occur intermittently in *V. cholerae* seventh pandemic strains, as this has occurred in multiple *V. cholerae* model strains [10,61, 62]. Importantly, a previous study did not find any fitness costs associated with large inversions in chromosome 1 [61].

To better understand if these mutations really affect the biology of *V. cholerae* C6706, we quantified the selected phenotypes. Firstly, this revealed a difference in mutation rate between the two strains (Fig. 2), which could potentially be attributed to a point mutation (C305Y) in RecA. This is a ubiquitous recombinase conserved throughout the bacterial kingdom, which evolves at a slow speed [70,75, 76]. The protein is involved in multiple important cellular processes including the SOS response, genome stability and repair and homologous recombination [66,69]. An increased mutation rate can be beneficial in some circumstances as it has been shown to accelerate evolution and increase the rate of adaptation [77,79].

The most striking phenotypic differences between the strains were differences in the biofilm formation and motility (Fig. 4a, b). QS-deficient *V. cholerae* C6706 formed increased biofilms and larger colonies on semi-solid agar plates compared to the QS-proficient C6706. These findings are in line with the current literature that QS inhibits both motility and biofilm formation [22,28]. Prior studies have demonstrated that QS regulates the biofilm formation through control of multiple c-di-GMP-metabolizing genes [27,28, 32,34, 80]. Typically, c-di-GMP regulates motility and biofilm formation inversely, i.e. high c-di-GMP concentrations increase biofilms and decrease motility, while low c-di-GMP concentrations do the opposite [35,37]. How QS inhibits both biofilm formation and motility at the same time in *V. cholerae* remains unclear. In certain cases, specific c-di-GMP-metabolizing enzymes asymmetrically regulate both biofilm formation and motility [35,81]. Consequently, QS might influence c-di-GMP-degrading enzymes that primarily inhibit biofilm formation without necessarily promoting increased motility. Alternatively, additional signalling pathways could also be involved, thereby modulating biofilm formation or motility independently of c-di-GMP.

*V. cholerae* N16961 exhibited higher motility than both variants of C6706 (Fig. 4b). This is in agreement with a previous study that showed that *V. cholerae* N16961 had increased motility compared to South American *V. cholerae* isolates closely related to C6706 [82]. In addition to increased motility due to QS deficiency, this could come from an altered c-di-GMP metabolism with decreased levels of c-di-GMP in *V. cholerae* N16961, e.g. due to the presence of a functional copy of the putative c-di-GMP-degrading enzyme VC1295. Alternatively, the genetic differences in multiple flagellar and chemotaxis-related proteins could also contribute to the observed difference in motility.

Interestingly, QS-deficient *V. cholerae* C6706 formed more biofilm and had lower motility compared to N16961 even though they should both be locked in the same QS state (Fig. 4a, b) [30]. This indicates that additional signalling pathways other than QS are mediating the observed phenotypic differences. The inverse effect on biofilm formation and motility resembles the effect of increased levels of c-di-GMP [35,37]. While some c-di-GMP-metabolizing enzymes in *V. cholerae* are known to be regulated by QS, many of them are seemingly regulated independently of QS [32,80, 83]. Therefore, we hypothesized that the differences between QS-deficient C6706 and N16961 could be due to the differences in the strains’ QS-independent c-di-GMP signalling systems. Indeed, *V. cholerae* C6706 contains multiple mutations in putative c-di-GMP-metabolizing enzymes (Table 4). This includes a frameshift mutation in VC1295, a functional c-di-GMP-degrading enzyme [65]. Indeed, the expression of a functional copy of VC1295 in QS-deficient C6706 reversed the observed differences in biofilm formation and motility (Fig. 5b, c), indicating that the observed differences were at least partly due to the differences in c-di-GMP metabolism between the strains, although the difference could also be due to additional QS-related signalling pathways as *V. cholerae* C6706 and N16961 contain different mutations, LuxO and HapR, respectively, in the QS signalling pathway. Even though the mutations lead to the same QS state, we cannot exclude that they have additional confounding downstream effects. Altogether, the results of this work are consistent with a model where the observed differences in biofilm formation and motility are due to a combination of effects from QS and c-di-GMP signalling.

*V. cholerae* N16961 and QS-proficient *V. cholerae* C6706 exhibited differences in the biofilm formation and motility, and the difference in motility exceeded the biofilm formation (Fig. 4a, b). In QS-proficient *V. cholerae* C6706, this might be attributed to QS-mediated repression of biofilm formation [28,32, 34, 80]. In addition, it harbours a frameshift mutation in the active c-di-GMP-degrading enzyme VC1295 (Table 4) [65]. This leads to putatively increased c-di-GMP levels and increased biofilm formation, which could partially balance out the QS-mediated biofilm repression (Fig. 4a). N16961 lacks QS-mediated biofilm repression but also lacks the frameshift mutation in VC1295. For motility, the QS state and c-di-GMP levels of QS-proficient * V. cholerae* C6706 would both act to repress motility (Fig. 4b) [22,36, 84]. In contrast, the QS state and putatively lower c-di-GMP levels of *V. cholerae* N16961 would both promote motility (Fig. 4b) [22,36, 84]. Therefore, the observed differences in biofilm formation and motility are consistent with the identified genetic changes between *V. cholerae* C6706 and N16961 (Table 4).

In conclusion, our study offers a genomic analysis of *V. cholerae* C6706, utilizing a hybrid sequencing approach. This yielded a high-quality genome of *V. cholerae* C6706, which we carefully annotated and cross referenced to N16961. We believe that this will be a valuable resource for the scientific community and represents an improvement of the previous version of the *V. cholerae* C6706 genome [11]. By characterizing genotypic and phenotypic differences between *V. cholerae* C6706 and N16961, we have uncovered the potential targets of adaptive evolution in the seventh cholera pandemic. Furthermore, the comparison of the biofilm formation and motility between *V. cholerae* C6706 and N16961 sheds further light on the complex interplay of factors regulating biofilm formation and motility in *V. cholerae* seventh pandemic strains.

## supplementary material

10.1099/mic.0.001502 Uncited Supplementary Material 1.

10.1099/mic.0.001502 Uncited Supplementary Material 2.

## Abbreviations

ATR: acid tolerance response
AUC: area under the curve
CDS: coding sequence
CV: crystal violet
gDNA: genomic DNA
LB: Lysogeny broth (Miller)
LCD: low cell density
MSS-MLE: Ma–Sandri–Sarkar maximum likelihood estimator
NGS: next-generation sequencing
QS: quorum sensing
RAST: Rapid Annotation and Subsystem Technology
WASA: West African–South American
