# Supplementary material for: A comparative genomic and phenotypic study of Vibrio cholerae model strains using hybrid sequencing
Source: Microbiology (Reading). 2024 Sep 23;170(9):001502. doi: 10.1099/mic.0.001502 (PMC11420891; doi:10.1099/mic.0.001502)
Supplement: Uncited Supplementary Material 1. [file mic-170-01502-s001.pdf]

A comparative genomic and phenotypic study of *Vibrio cholerae* model strains using hybrid sequencing

Øyvind M. Lorentzen (orcid.org/0009-0007-3386-0794)[1,#], Christina Bleis[1], Sören Abel (orcid.org/0000-0002-4041-6989)[1,2,#]

[1] Department of Pharmacy, UiT The Arctic University of Norway, Tromsø, Norway

[2] Division of Infection Control, Norwegian Institute of Public Health, Oslo, Norway

[#] corresponding authors: oyvind.m.lorentzen@uit.no and soren.abel@fhi.no

Supplemental material

Contents

Figure S1. Mauve visualization of the *V. cholerae* C6706 and N16961 genomes.

Figure S2. Mauve visualization of the *V. cholerae* C6706 genomes.

Table S1. Variant analysis between the here assembled hybrid *V. cholerae* C6706 genome and the short Illumina reads used for the assembly.

Table S2. Genome annotation of *V. cholerae* C6706 linked to the N16961 annotation.

Table S3. Variant analysis between *V. cholerae* C6706 and N16961.

Table S4. Variant analysis between QS-proficient and QS-deficient *V. cholerae* C6706 strains.

Table S5. *V. cholerae* strains N16961, QS-deficient and QS-proficient C6706 have comparable fitness.

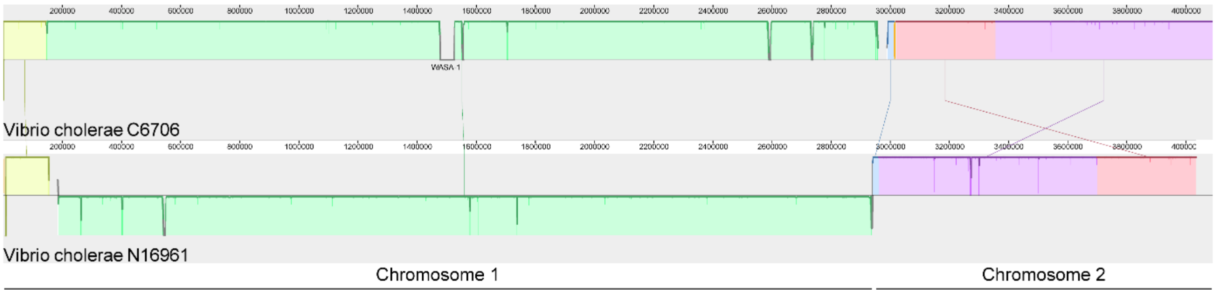

Figure S1. Mauve visualization of the *V. cholerae* C6706 and N16961 genomes.

Genome comparison between *V. cholerae* C6706 and N16961 conducted with progressivMauve. Each genome is visualized on a horizontal track with the genome of *V. cholerae* C6706 at the top. Each genome has a colored similarity plot, where the height of the plot is proportional to the level of sequence identity in that particular region. Colors in the similarity plot indicate a specific segment of the genome across *V. cholerae* C6706 and N16961, which are linked by a line with the same color. A downward pointing similarity plot indicates an alignment to the reverse strand of the genome (i.e. inversion).

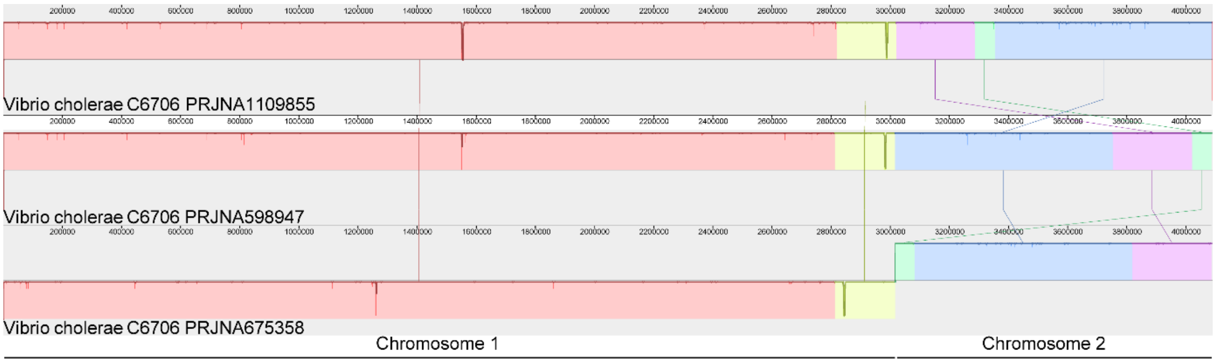

Figure S2. Mauve visualization of *V. cholerae* C6706 genomes.

Genome comparison between different *V. cholerae* C6706 genomes conducted with progressivMauve. Each genome is visualized on a horizontal track with the genome of our newly sequenced *V. cholerae* C6706 genome at the top. Each genome has a colored similarity plot, where the height of the plot is proportional to the level of sequence identity in that particular region. Colors in the similarity plot indicate a specific segment of the genome across *V. cholerae* C6706, which are linked by a line with the same color. A downward pointing similarity plot indicates an alignment to the reverse strand of the genome (i.e. inversion).

Table S1. Variant analysis between the here assembled hybrid *V. cholerae* C6706 genome and the short Illumina reads used for the assembly

Chr	Pos	Ref	Var	Score	Var_cov	Var_frac	Type	Ref_nt	Var_nt	Ref_nt_pos_change	Ref_aa_pos_change	Frameshift	Gene_ID	Function
1	148560	GTGT	GTTGA	177	8	0.89	Insertion							
1	151340	G	A	1568	56	1.00								
1	154306	GTGT	GTTGA	1674	69	0.97	Insertion							
1	1563263	G	A	3633	131	1.00	Synon	aag	aaA	186G>A	Lys62Lys		fig|127906.87.peg.1435	RstB phage-related integrase
1	1569857	C	T	213	9	1.00	Synon	cgc	cgT	927C>T	Arg309Arg		fig|127906.87.peg.1444	RstA phage-related replication protein
1	1569863	T	C	243	9	1.00	Synon	gtt	gtC	933T>C	Val311Val		fig|127906.87.peg.1444	RstA phage-related replication protein
1	1569872	T	G	213	9	1.00	Synon	act	acG	942T>G	Thr314Thr		fig|127906.87.peg.1444	RstA phage-related replication protein
1	1624654	G	T	7821	261	1.00								
1	1705326	A	T	3350	109	1.00	Synon	gtt	gtA	99T>A	Val33Val		fig|127906.87.peg.1552	Mobile element protein
1	2161776	TC	TAT	684	71	0.62	Insertion							
1	2162159	G	A	593	36	0.71								
1	2162176	T	C	1686	83	0.75								
1	2162189	C	T	1646	110	0.63								
1	2199435	A	T	5494	191	1.00	Synon	gtt	gtA	99T>A	Val33Val		fig|127906.87.peg.2000	Mobile element protein
1	2372224	A	G	541	21	0.91								
1	2372499	G	A	1789	64	0.97								
1	2738621	A	G	259	10	1.00							fig|127906.87.rna.92	SSU rRNA ## 16S rRNA, small subunit ribosomal RNA
1	2758471	ATAA	GTAT	564	21	0.95	Synon	acttat	acATAC	594_597delTTATinsATAC	200		fig|127906.87.peg.2491	Translation elongation factor Tu
1	2804807	T	C	7724	277	0.99	Synon	gaa	gaG	1038A>G	Glu346Glu		fig|127906.87.peg.2532	Translation elongation factor Tu
1	2804819	A	G	7898	271	1.00	Synon	att	atC	1026T>C	Ile342Ile		fig|127906.87.peg.2532	Translation elongation factor Tu
1	2804900	G	A	5293	190	1.00	Synon	gac	gaT	945C>T	Asp315Asp		fig|127906.87.peg.2532	Translation elongation factor Tu
1	2805011	C	T	3225	113	1.00	Synon	ctg	ctA	834G>A	Leu278Leu		fig|127906.87.peg.2532	Translation elongation factor Tu
1	2985192	C	T	3706	129	0.99								
1	2985199	ACAC	TCAAC	3381	123	0.98	Insertion							
1	2985218	T	C	5031	181	1.00								
1	2990374	GTTTTTTGA	GTTTTTGA	6144	236	1.00	Deletion							
1	401160	C	T	1884	68	0.99							fig|127906.87.rna.19	SSU rRNA ## 16S rRNA, small subunit ribosomal RNA
1	401435	T	C	150	6	1.00							fig|127906.87.rna.19	SSU rRNA ## 16S rRNA, small subunit ribosomal RNA
1	46547	AGGGGGAT	AGGGGGGAT	5209	192	0.97	Insertion						fig|127906.87.rna.1	SSU rRNA ## 16S rRNA, small subunit ribosomal RNA
2	608054	T	C	4980	185	0.97	Synon	cca	ccG	210A>G	Pro70Pro		fig|127906.87.peg.3256	hypothetical protein
2	651470	T	C	6509	208	1.00	Nonsyn	gta	gCa	110T>C	Val37Ala		fig|127906.87.peg.3306	hypothetical protein
2	660981	A	C	4737	151	1.00								
2	660987	A	C	4725	152	0.99								
2	660997	T	C	4933	158	0.99								
2	661062	T	C	5490	180	0.98								
2	673253	A	G	155	6	1.00								
2	694269	G	A	4314	155	1.00	Nonsyn	gta	Ata	334G>A	Val112Ile		fig|127906.87.peg.3377	hypothetical protein
2	732146	T	C	1599	65	0.98	Synon	ggt	ggC	108T>C	Gly36Gly		fig|127906.87.peg.3444	Lipocalin Blc
2	732158	AGTC	GGTT	819	32	0.94	Synon	gaagtc	gaGGTT	120_123delAGTCinsGGTT	42		fig|127906.87.peg.3444	Lipocalin Blc
2	732187	A	G	303	13	1.00	Nonsyn	aaa	aGa	149A>G	Lys50Arg		fig|127906.87.peg.3444	Lipocalin Blc
2	732290	C	G	1558	58	1.00	Synon	gcc	gcG	252C>G	Ala84Ala		fig|127906.87.peg.3444	Lipocalin Blc
2	732296	C	T	1437	58	0.98	Synon	ggc	ggT	258C>T	Gly86Gly		fig|127906.87.peg.3444	Lipocalin Blc
2	732326	C	T	2924	98	1.00	Synon	gac	gaT	288C>T	Asp96Asp		fig|127906.87.peg.3444	Lipocalin Blc
2	732353	T	C	3733	127	1.00	Synon	ggt	ggC	315T>C	Gly105Gly		fig|127906.87.peg.3444	Lipocalin Blc
2	745924	A	G	3859	164	0.80	Synon	caa	caG	156A>G	Gln52Gln		fig|127906.87.peg.3469	biphenyl-2,3-diol 1,2-dioxygenase III-related protein
2	745939	T	C	3447	155	0.78	Synon	ttt	ttC	171T>C	Phe57Phe		fig|127906.87.peg.3469	biphenyl-2,3-diol 1,2-dioxygenase III-related protein
2	745951	C	T	3470	159	0.77	Synon	gcc	gcT	183C>T	Ala61Ala		fig|127906.87.peg.3469	biphenyl-2,3-diol 1,2-dioxygenase III-related protein
2	746056	G	A	3636	176	0.76	Synon	ggg	ggA	288G>A	Gly96Gly		fig|127906.87.peg.3469	biphenyl-2,3-diol 1,2-dioxygenase III-related protein

2	755991	A	G	3400	117	1.00								
2	761908	A	G	3921	127	0.99	Nonsyn	gat	gGt	179A>G	Asp60Gly		fig|127906.87.peg.3500	Mobile element protein
2	841399	GGTT	GT	1972	65	1.00	Deletion							
2	841410	ATG	AG	1764	65	1.00	Deletion							
2	841419	C	A	1863	65	0.98								
2	841437	C	A	2499	84	1.00								
2	841460	C	A	3107	105	1.00								
2	841478	ATTTTAC	ATTTAC	2132	80	0.99	Deletion							
2	841523	GTTTAAG	GTTAAG	4890	165	0.98	Deletion							

Table S2. Genome annotation of *V. cholerae* C6706 linked to the N16961 annotation

chr	type	start	stop	strand	nucleotide_sequence	aa_sequence	Gene_ID	Locus_tag_C6706	Locus_tag_N16961	gene_name	function	alternative_gene_function	Comments
1	CDS	1	1404	+	gtgtcatcttcgctatggttgcaat	VSSSLWLQCLQRLQEELPAAE	fig|127906.87.peg.1	VCC0001	VC0012	DnaA	Chromosomal replication initiator protein DnaA		
1	CDS	1462	2562	+	atgaaatttaccattgaacgtag	MKFTIERSHLIKPLQQVSGTLG	fig|127906.87.peg.2	VCC0002	VC0013	DnaN	DNA polymerase III beta subunit (EC 2.7.7.7)		
1	CDS	2580	3671	+	atgccgctttcccggttaatgatc	MPLSRLMIQQFRNIKACDIRLS	fig|127906.87.peg.3	VCC0003	VC0014	recF	DNA recombination and repair protein RecF		
1	CDS	3692	6109	+	atgtcgaacaattacgattcatc	MSNNYDSSSIKVLKGLDAVRK	fig|127906.87.peg.4	VCC0004	VC0015	gyrB	DNA gyrase subunit B (EC 5.99.1.3)		
1	CDS	6534	6956	+	atgctgaatcggttatctttgaata	MLNRLSLNIRRFLAGWWLRSF	fig|127906.87.peg.5	VCC0005	VC0016		hypothetical protein	transcription elongation factor GreAB	
1	CDS	6966	7082	+	atgagtaaacaagagatgaaa	MSKQEMKKPQLSLKEKRKLK	fig|127906.87.peg.6	VCC0006	VC0017		hypothetical protein		
1	CDS	7358	7795	+	atgagaactgtagattttactcca	MRTVDFTPLYRNAIGFDRLLNI	fig|127906.87.peg.7	VCC0007	VC0018	ibpA	16 kDa heat shock protein A		
1	CDS	9139	7883	-	atgcagtattcctcttttggcgaaa	MQYSSFGEKFNRYSGITQLMI	fig|127906.87.peg.8	VCC0008	VC0019	avtA	Valine--pyruvate aminotransferase (EC 2.6.1.66)		
1	repeat	9261	9382	+	tccccttcctacttgaagctgcagcggtgttggcggcgttcgttcaccccaatc		fig|127906.87.repeat.1				repeat region		
1	CDS	11524	9458	-	atggcaaaagaatttttaattgac	MAKEFLIELGTEELPPKQLRTL	fig|127906.87.peg.9	VCC0009	VC0020	glyS	Glycyl-tRNA synthetase beta chain (EC 6.1.1.14)		
1	CDS	12456	11539	-	atgcaaaaatacgacatcaaa	MQKYDIKTFQGMILALQDYWA	fig|127906.87.peg.10	VCC0010	VC0021	glyQ	Glycyl-tRNA synthetase alpha chain (EC 6.1.1.14)		
1	CDS	12872	13426	+	atgagcgttttagctatttcgattac	MSVLAISITTVALAEIGDKTQLL	fig|127906.87.peg.11	VCC0011	VC0022		Putative transmembrane protein		
1	CDS	13532	13789	+	atgctgactcgttacatgggatg	MLTRYMGMTPKSQSYLFSFG	fig|127906.87.peg.12	VCC0012	VC0023		NADH dehydrogenase subunit II-related protein		
1	CDS	14124	13876	-	atgactttcaatccgaacattgcc	MTFNPNIATHTLEAEGLRCPEI	fig|127906.87.peg.13	VCC0013	VC0024	tusA	tRNA 5-methylaminomethyl-2-thiouridine synthesis sulfur carrier protein TusA		
1	CDS	14257	14445	+	atgatgttaaccacactcgatag	MMLTTLDRLTIYAVLCFISLCAI	fig|127906.87.peg.14	VCC0014	VC0025		hypothetical protein		
1	CDS	15597	14617	-	atgttcaacgcgttgctgcttactc	MFNALLLTQQDKITHATVTQIN	fig|127906.87.peg.15	VCC0015	VC0026	acul	Acryloyl-CoA reductase AcuI/YhdH (EC 1.3.1.84)		
1	CDS	17377	15845	-	atggggttaaccaatcaaactgg	MGLTNQTGAEYLRQILRSPVY	fig|127906.87.peg.16	VCC0016	VC0027	ilvA	Threonine dehydratase biosynthetic (EC 4.3.1.19)		
1	CDS	19222	17381	-	atgcctaagtaccgttccgccac	MPKYRSATTTHGRNMAGARA	fig|127906.87.peg.17	VCC0017	VC0028	ilvD	Dihydroxy-acid dehydratase (EC 4.2.1.9)		
1	CDS	20170	19232	-	atggcaacaaaaactgctgatta	MATKTADYIWFNGEMIPWANA	fig|127906.87.peg.18	VCC0018	VC0029	ilvE	Branched-chain amino acid aminotransferase (EC 2.6.1.42)		
1	CDS	20466	20182	-	atgcaacgttatctactcgatatta	MQRYLLDIKADDKPVLLERVLF	fig|127906.87.peg.19	VCC0019	VC0030	ilvM	Acetolactate synthase small subunit (EC 2.2.1.6)		
1	CDS	22124	20478	-	atgaccggagcacagttagtcg	MTGAQLVVAALKQQGIKTVFG	fig|127906.87.peg.20	VCC0020	VC0031	ilvG	Acetolactate synthase large subunit (EC 2.2.1.6)		
1	CDS	22658	24181	+	atgggacttgcgatcattcatagc	MGLAIIHSRASIGVQAPPVTVE	fig|127906.87.peg.21	VCC0021	VC0032	comM	AAA+ ATPase superfamily protein YifB/ComM, associated with DNA recombination		
1	CDS	24271	25155	+	atgttggcttacttgctccttacgc	MLAYLLLTLNVTVVVLNSAFHS	fig|127906.87.peg.22	VCC0022	VC0033	YihG	Probable acyltransferase YihG		
1	CDS	25835	25233	-	atgaaaaagctgtttgcactggtt	MKKLFALVATLMLSVSAYAAQ	fig|127906.87.peg.23	VCC0023	VC0034	dsbA/tpcG	Periplasmic thiol:disulfide interchange protein DsbA		
1	CDS	26870	25866	-	atgagcccttttattcaatgaccc	MSPFYSMTQTTFHFDALTPDL	fig|127906.87.peg.24	VCC0024	VC0035		YihE protein, a ser/thr kinase implicated in LPS synthesis and Cpx signalling		
1	CDS	28324	26900	-	atgagtcaggataaaatcgatgt	MSQDKIDVKDVTPKVFNPKTH	fig|127906.87.peg.25	VCC0025	VC0036		Type cbb3 cytochrome oxidase biogenesis protein CcoG, involved in Cu oxidation		
1	repeat	28587	28715	+	ttataccttcctacttgaagctgcagcggtgtttgctacgctcgttcaccccaat		fig|127906.87.repeat.2				repeat region		
1	CDS	29007	28741	-	atgaaatgtcatcgcgtagaaga	MKCHRVEELLQLLEPEWHKD	fig|127906.87.peg.26	VCC0026	VC0037	YihD	Protein YihD		
1	CDS	29238	29116	-	atgcgtcgccgcagtggttttgca	MRRRSGFAVAPVAKKTATAPT	fig|127906.87.peg.27	VCC0027	VC0038		hypothetical protein		
1	CDS	29568	30359	+	atgtcgtttatcaagaaaaccctc	MSFIKKTLASFGIGSAKVDSVL	fig|127906.87.peg.28	VCC0028	VC0039	spo0M	Transcriptional regulator		
1	CDS	30443	31084	+	atgtcgaacagttatggctttaaa	MSNSYGFKEEVANAISHGVGL	fig|127906.87.peg.29	VCC0029	VC0040		FIG01964566: Predicted membrane protein, hemolysin III homolog		
1	CDS	31680	31123	-	atgaaaaaactcatattggtagc	MKKLILVASIVSLSTWASETLQ	fig|127906.87.peg.30	VCC0030	VC0041		hypothetical protein	DUF3157	
1	CDS	33137	31692	-	atgctcaacttacgcccaattacc	MLNLRPITFIIGLVLSKLALFMY	fig|127906.87.peg.31	VCC0031	VC0042	trkH	Trk potassium uptake system protein TrkH		
1	CDS	34525	33149	-	atgaaaatcatcattcttggtgca	MKIIILGAGQVGGTLAENLVGE	fig|127906.87.peg.32	VCC0032	VC0043	TrkA	Trk potassium uptake system protein TrkA		
1	CDS	35867	34587	-	atgaatgttcgcgccgctgctgc	MNVRAAAASALYQVVDLGHS	fig|127906.87.peg.33	VCC0033	VC0044	rsmB/sun	16S rRNA (cytosine(967)-C(5))-methyltransferase (EC 2.1.1.176)		
1	CDS	36870	35923	-	ttgagccaatcactacgtattgtc	MSQSLRIVFAGTPDFAARHLA	fig|127906.87.peg.34	VCC0034	VC0045	fmt	Methionyl-tRNA formyltransferase (EC 2.1.2.9)		
1	CDS	37397	36888	-	atgtctgtattgcaagtattaacat	MSVLQVLTFPDDRLRTVAKPV	fig|127906.87.peg.35	VCC0035	VC0046	def	Peptide deformylase (EC 3.5.1.88)		
1	CDS	37537	38664	+	atggcacgattaacccgttatatt	MARLTRYITYALLPFTLLSGQV	fig|127906.87.peg.36	VCC0036	VC0047		Uncharacterized protein with LysM domain, COG1652		
1	CDS	38664	39779	+	atgaaagatcaggatttagcggc	MKDQDLAAWLALCFTPKLGSI	fig|127906.87.peg.37	VCC0037	VC0048		Rossmann fold nucleotide-binding protein Smf possibly involved in DNA uptake		
1	CDS	39782	40258	+	atgatgatggatattttgatgtatc	MMMDILMYLFETYVHSDADLQ	fig|127906.87.peg.38	VCC0038	VC0049	smg	Protein of unknown function Smg		
1	CDS	40274	40840	+	atgagtcgtaaaattgacccgca	MSRKIDPQLFAAHEHALQSSP	fig|127906.87.peg.39	VCC0039	VC0050	topA_1	Uncharacterized protein YrdD		
1	CDS	42046	40913	-	atgcgtgtgttggttctcggcgctc	MRVLVLGAGQLARMMSLAGA	fig|127906.87.peg.40	VCC0040	VC0051	purK	N5-carboxyaminoimidazole ribonucleotide synthase (EC 6.3.4.18)		
1	CDS	42535	42050	-	atgacagtcggtattatcatgggt	MTVGIIMGSKSDWPTMKHAAE	fig|127906.87.peg.41	VCC0041	VC0052	purE	N5-carboxyaminoimidazole ribonucleotide mutase (EC 5.4.99.18)		
1	CDS	42550	42675	+	gtgttatttatcggacaagcaagc	MLFIGQASPLAAHYTGIFFSSK	fig|127906.87.peg.42	VCC0042	Not annotated as CDS		hypothetical protein	DUF1488 family protein	
1	CDS	42702	43259	+	gtggagaatctacaacaggcgg	MENLQQAVDALRKGCVIAYPT	fig|127906.87.peg.43	VCC0043	VC0054		Threonylcarbamoyl-AMP synthase (EC 2.7.7.87)		
1	CDS	43278	44195	+	gtggagtcgatagtcgataaaca	MESIVDKQAVKHFLLQLQDKIQ	fig|127906.87.peg.44	VCC0044	VC0055	hemF	Coproporphyrinogen III oxidase, aerobic (EC 1.3.3.3)		
1	CDS	44257	45093	+	atggcttctcaaatcgatcagtat	MASQIDQYAVFGNPINHSKSP	fig|127906.87.peg.45	VCC0045	VC0056	aroE	Shikimate 5-dehydrogenase I alpha (EC 1.1.1.25)		
1	CDS	45090	45365	+	atgaaccaatccatcttgtttcccg	MNQSILFPDLQHWDEAKQAV	fig|127906.87.peg.46	VCC0046	VC0057		FIG034389 (not subsystem-based): hypothetical protein		
1	CDS	45897	45346	-	atgagttcaattcgtagctacaaa	MSSIRSYKGIVPKLGEGVYVD	fig|127906.87.peg.47	VCC0047	VC0058		Protein YrdA		
1	repeat	46037	51612	+	tctctataatccgccctcactgatacggcagacgcagtaagcgttagcagtga		fig|127906.87.repeat.3				repeat region		
1	CDS	46086	46226	+	gtgattcagctcggtgttttaggtc	MIQLGVLGREIANENKLKKVF	fig|127906.87.peg.48	VCC0048	VC0059		hypothetical protein		
1	rna	46402	47949	+	ttttaattgaagagtttgatcatggctcagattgaacgctggcggcaggcctaa		fig|127906.87.rna.1	VCCr001	VCr001		SSU rRNA ## 16S rRNA, small subunit ribosomal RNA		
1	rna	48010	48085	+	ggggctatagctcagctgggagagcgcttgcatggcatgcaagaggtctgc		fig|127906.87.rna.2	VCCt001	VCt001		tRNA-Ala-GGC		
1	rna	48373	51261	+	ggttaagtgactaagcgtacacggtggatgcctgggcagtcagaggcgatg		fig|127906.87.rna.3	VCCr002	VCr002		LSU rRNA ## 23S rRNA, large subunit ribosomal RNA		
1	rna	51386	51507	+	ttgcttggcgaccatagcgttttggacccacctgattccatcccgaactcagaa		fig|127906.87.rna.4	VCCr003	VCr003		5S rRNA ## 5S ribosomal RNA		
1	rna	51530	51606	+	ggagcggtagttcagtcggttagaataccggcctgtcacgccgggggtcgc		fig|127906.87.rna.5	VCCt002	VCt002		tRNA-Asp-GTC		
1	rna	51636	51712	+	aggggtgtagctccaattggcagagcagcggattccaaatccgcgtgtgg		fig|127906.87.rna.6	VCCt003	VCt003		tRNA-Trp-CCA		
1	CDS	51945	52325	+	atgtcattcgcgatattgggttta	MSFAILGFIALGGAVGACARFL	fig|127906.87.peg.49	VCC0049	VC0060	ccrB/crcB	Fluoride ion transporter CrcB		
1	CDS	52577	54514	+	atgtcgaaccgtaaacaagcaa	MSNRKQARLEAKRFIDTLSVE	fig|127906.87.peg.50	VCC0050	VC0061	thiC	Phosphomethylpyrimidine synthase ThiC (EC 4.1.99.17)		
1	CDS	54518	55840	+	atggtacgtttggtttttccacgtca	MVRLVFPRHLSALIGHVQYAL	fig|127906.87.peg.51	VCC0051	VC0062	thiE	Uncharacterized domain 1 associated with ThiE / Thiamin-phosphate pyrophosphorylase (EC 2.5.1.3)		
1	CDS	55827	56603	+	atgctcacagataaacagtttctt	MLTDKQFLRYQRQISLAELGE	fig|127906.87.peg.52	VCC0052	VC0063	thiF	Sulfur carrier protein ThiS adenylyltransferase (EC 2.7.7.73)		
1	CDS	56597	56803	+	gtgctaatgactatctacttgaatc	MLMTIYLNQQAIQTDISSSLFH	fig|127906.87.peg.53	VCC0053	VC0064	thiS	Sulfur carrier protein ThiS		
1	CDS	56809	57579	+	atgctcaagattgcagataaaac	MLKIADKTFQSRLFTGTGKFSI	fig|127906.87.peg.54	VCC0054	VC0065	thiG	Thiazole synthase (EC 2.8.1.10)		
1	CDS	57576	58688	+	atgagcttcattcacccacttccaa	MSFITHFQQLGWDDSRLSIYG	fig|127906.87.peg.55	VCC0055	VC0066	thiH	2-iminoacetate synthase (ThiH) (EC 4.1.99.19)		
1	CDS	60706	58913	-	atgtcaaacctacactctcaacg	MSNLHSQRLADFRHWLHTQC	fig|127906.87.peg.56	VCC0056	VC0067	pepQ	Xaa-Pro aminopeptidase (EC 3.4.11.9)		
1	CDS	61780	60839	-	atgcaaattgaaaaattggcccg	MQIEKLARIDLNLLVCLKVLIEE	fig|127906.87.peg.57	VCC0057	VC0068	yidZ	LysR-family transcriptional regulator VC0068		
1	CDS	61885	63087	+	gtgaaacaagcaatgccatctc	MKQAMPSQVSKQQMVLLTLL	fig|127906.87.peg.58	VCC0058	VC0069	mdtL_1	Multidrug resistance transporter, Bcr/CflA family		
1	CDS	63191	63475	+	atgtctctaaccaccctatgaaatg	MSLTTYEMARILEQLDESPEK	fig|127906.87.peg.59	VCC0059	VC0070		FIG01199646: hypothetical protein		
1	CDS	64025	63564	-	atgcaaaccaccaccaaactcg	MQTTTKLDELDRAILKILMDDA	fig|127906.87.peg.60	VCC0060	VC0071	asnC	Transcriptional regulator AsnC		
1	CDS	64113	66407	+	ttgagccgattgcgacactggta	MSRLRHWYNKYALPLAVGLG	fig|127906.87.peg.61	VCC0061	VC0072	YegE	Sensory box/GGDEF family protein		
1	CDS	66467	67252	+	ttgttgattcaactgatttgccaag	MLIQLICQAPQRAQELEQIAAR	fig|127906.87.peg.62	VCC0062	VC0073	rsmJ	16S rRNA (guanine(1516)-N(2))-methyltransferase (EC 2.1.1.242)		
1	CDS	67376	67759	+	gtggctaaacgtctatgcaaact	MAKRLCKLSRHEIATSLSEIER	fig|127906.87.peg.63	VCC0063	VC0074		hypothetical protein		
1	CDS	68742	67855	-	atggtttatctttcagcagtcacc	MVYLSAVTLLWAFSFSLIGVYL	fig|127906.87.peg.64	VCC0064	VC0075	madN	Putative acetate efflux pump, MadN		
1	CDS	69224	68799	-	atgagttatcaacatcttttggttg	MSYQHLLVAVDLSEDSKLLVE	fig|127906.87.peg.65	VCC0065	VC0076	uspA	Universal stress protein A		
								Not annotated as CDS	VC0077		hypothetical protein present in N16961 according to KEGG		
1	CDS	69389	69916	+	atgttgtcgcaagcgatggtaga	MLSQAMVEHLNEQINLEFFSS	fig|127906.87.peg.66	VCC0066	VC0078	ftnA	Bacterial non-heme ferritin (EC 1.16.3.2)		
1	CDS	69992	70315	+	atgatcagtggagataccattcta	MISGDTILFALMLVTAINVARY	fig|127906.87.peg.67	VCC0067	VC0079	uspB	Universal stress protein B		
1	CDS	70457	71644	+	atgagcaaaacagtcgatgtgg	MSKTVDVVIIGAGAAGLMCAA	fig|127906.87.peg.68	VCC0068	VC0080		Uncharacterized protein YhiN		
1	CDS	72552	71641	-	atgaatgaaaacgcgcgttga	MNEKRALSLGLASVLLWSTVA	fig|127906.87.peg.69	VCC0069	VC0081		Uncharacterized transporter VC0081, DMT family		
1	CDS	72675	74207	+	atgcaatggattttgaaaatcaa	MQWIFENQSALWSALVSAGA	fig|127906.87.peg.70	VCC0070	VC0082	mucC	DNA recombination protein RmuC		
1	CDS	74252	75034	+	atgacggacaccaacgtgctag	MTDTNVLANSATDNQETTHFC	fig|127906.87.peg.71	VCC0071	VC0083	ubiE	2-methoxy-6-polyprenyl-1,4-benzoquinol methylase (EC 2.1.1.201) @ Demethylmenaquinone methyltransferase (EC 2.1.1.163)		
1	CDS	75043	75666	+	atgtcattgcttgcaaccatgcca	MSLLATMPLSPLLTAVIETTLN	fig|127906.87.peg.72	VCC0072	VC0084	ubiJ	Ubiquinone biosynthesis protein UbiJ		
1	CDS	75663	77297	+	atgaaacctgcagagctgaaac	MKPAELKRLYRIVKVQLEYGLI	fig|127906.87.peg.73	VCC0073	VC0085	ubiB	Ubiquinone biosynthesis regulatory protein kinase UbiB		
1	CDS	77357	77605	+	atgggtggtatcagtatttggcaa	MGGISIWQLLIIAVIVVLLFGTKI	fig|127906.87.peg.74	VCC0074	VC0086	tatA	Twin-arginine translocation protein TatA		
1	CDS	77609	78010	+	gtgtttgatatcggttttgggaact	MFDIGFWELVLIAIVALVVLGPE	fig|127906.87.peg.75	VCC0075	VC0087	tatB	Twin-arginine translocation protein TatB		
1	CDS	78099	78851	+	atgtcttccgttgaacagaccca	MSSVEQTQPLISHLLELRNRLL	fig|127906.87.peg.76	VCC0076	VC0088	tatC	Twin-arginine translocation protein TatC		
1	CDS	79163	80164	+	atgaaagtcgttcttacctcgctg	MKVVLTSLSLAISLSLAGYAFA	fig|127906.87.peg.77	VCC0077	VC0089	ccp/yhjA	Cytochrome c551 peroxidase (EC 1.11.1.5)		
1	CDS	81625	80270	-	gtgtcactgattttgcaaacgttga	MSLILQTLKQPNVHRQVLAIAL	fig|127906.87.peg.78	VCC0078	VC0090	dinF	DNA-damage-inducible protein F		
1	CDS	82517	81705	-	atgcgccactcagattcacccac	MRHSDSPTSDSYQVPANLVQ	fig|127906.87.peg.79	VCC0079	VC0091		Uncharacterized SAM-dependent O-methyltransferase		
1	CDS	83231	82602	-	atgaagccgttaacccctcgcca	MKPLTPRQQEVFDLIKSKIDET	fig|127906.87.peg.80	VCC0080	VC0092	lexA	SOS-response repressor and protease LexA (EC 3.4.21.88)		
1	CDS	83551	85977	+	atgtcttcaggacacctattatcc	MSSGHLLSRSLLKLPMSVLVK	fig|127906.87.peg.81	VCC0081	VC0093	plsB	Glycerol-3-phosphate acyltransferase (EC 2.3.1.15)		
1	CDS	86907	86053	-	atgaccgctgtaaaagcgcgcg	MTAVKARAYWQLMRMDRPIG	fig|127906.87.peg.82	VCC0082	VC0094	ubiA	4-hydroxybenzoate polyprenyltransferase (EC 2.5.1.39)		
1	CDS	87447	86908	-	atgaatcaattaacttcgctctatt	MNQLTSLYLAALNRVTWQQP	fig|127906.87.peg.83	VCC0083	VC0095	ubiC	Chorismate--pyruvate lyase (EC 4.1.3.40)		
								Not annotated as CDS	VC0096		hypothetical protein present in N16961 according to KEGG		
1	CDS	87651	88061	+	atgctaaaacgttacgtcgtcca	MLKRYVVQIMLALSFIVPFHSH	fig|127906.87.peg.84	VCC0084	VC0097		Similar to flagellar basal body-associated protein FliL		
1	CDS	90160	88181	-	atgggtttattggcacgtcttttctc	MGLLARLFSTAPHHPTQPAEV	fig|127906.87.peg.85	VCC0085	VC0098	tar1	Methyl-accepting chemotaxis sensor/transducer protein		
1	CDS	91368	90535	-	atgcatttactgaccactttaaca	MHLLTTFNNPRAAQAFIDYMA	fig|127906.87.peg.86	VCC0086	VC0099	glpG	Rhomboid protease GlpG (EC 3.4.21.105)		
1	CDS	91695	91375	-	atggaccatttttacacatcgac	MDHFLHIDVNAAQAMMEQKQ	fig|127906.87.peg.87	VCC0087	VC0100	glpE	Thiosulfate sulfurtransferase GlpE (EC 2.8.1.1)		
1	CDS	93223	92456	-	atgattgatacccatgcccacgtt	MIDTHAHVYASEFDHDRDEVI	fig|127906.87.peg.88	VCC0088	VC0103	ycfH2	Putative deoxyribonuclease similar to YcfH, type 2		
1	CDS	93413	93279	-	atggataccgacactctattctcc	MDTDTLFSFLCGQCLKNQVLF	fig|127906.87.peg.89	VCC0089	VC0104		hypothetical protein		
1	CDS	93400	94443	+	gtgtcggtatccattcaaggtcaa	MSVSIQGQFPGRRLRRLRKHI	fig|127906.87.peg.90	VCC0090	VC0105	hemB	Porphobilinogen synthase (EC 4.2.1.24)		
1	CDS	94550	94978	+	atgttaattcgtgaaggttccctga	MLIREGSLSEIVQIVEKIHEFAC	fig|127906.87.peg.91	VCC0091	VC0106	ypeA2	hypothetical protein	GNAT family N-acetyltransferase	
1	CDS	95625	95497	-	atgttgttacgttttggctcattca	MLLPFLAHSGDIRPTYPTLHSV	fig|127906.87.peg.92	VCC0092	No refSeq annotation		hypothetical protein		
1	CDS	95687	98491	+	atggctcatatacctgacaatcc	MAHIPDNPLILIDGSSYLYRAFI	fig|127906.87.peg.93	VCC0093	VC0108	polA2/polI	DNA polymerase I (EC 2.7.7.7)		
1	CDS	99900	99238	-	gtgagcgtaaaaattcattacca	MSVKIHYQNTHFITSAPDIRHL	fig|127906.87.peg.94	VCC0094	VC0111	engB	GTP-binding protein EngB		
1	CDS	100052	100669	+	atgaagaaattagcgctaatcttc	MKKLALILSVLASCSVWAQGS	fig|127906.87.peg.95	VCC0095	VC0112	cc4/cycA	Cytochrome c4		
1	CDS	100871	101509	+	atgcatctatgcccgttatgtcac	MHLCPLCHSENSQAYFADKQ	fig|127906.87.peg.96	VCC0096	VC0113		Putative methyltransferase associated with DUF414		
1	CDS	101509	102057	+	atgagtcgctcaaaaaaatcga	MSRSKKSRKPGTNSNDQLVV	fig|127906.87.peg.97	VCC0097	VC0114	yihI	Der GTPase-activating protein YihI		
1	CDS	102067	102555	+	atgaacgggatgctactggctag	MNGMLLASVGGAIIVALASYA	fig|127906.87.peg.98	VCC0098	VC0115		Periplasmic/membrane protein associated with DUF414		
1	CDS	102694	104082	+	atgtcgtcgtatgtcgtacccagc	MSSYVVPSQQIVWDQAILDKY	fig|127906.87.peg.99	VCC0099	VC0116	hemN	Coproporphyrinogen III oxidase, oxygen-independent (EC 1.3.99.22)		
1	CDS	105342	104146	-	atgattcgtctcatattcctctttgtt	MIRLIFLFVVLGLGLFVGTQYA	fig|127906.87.peg.100	VCC0100	VC0117	HemY	Uncharacterized protein EC-HemY in Proteobacteria (unrelated to HemY-type PPO in GramPositives)		
1	CDS	106538	105339	-	atgaccaacaacaataaaaccg	MTNNNKPQQEPEITVNPSSAA	fig|127906.87.peg.101	VCC0101	VC0118	hemX	Uncharacterized protein EC-HemX		
1	CDS	107289	106522	-	atgacagtactggtcactcgtcct	MTVLVTRPDEQGAELCQLLHC	fig|127906.87.peg.102	VCC0102	VC0119	hemD	Uroporphyrinogen-III synthase (EC 4.2.1.75)		
1	CDS	108285	107350	-	atgaccgagacgccgattcgca	MTETPIRIATRQSPLALWQAN	fig|127906.87.peg.103	VCC0103	VC0120	hemC	Porphobilinogen deaminase (EC 2.5.1.61)		
								Not annotated as CDS	VC0121		hypothetical protein present in N16961 according to KEGG		
1	CDS	108519	111173	+	atgtgcttccaagagcgctttacc	MCFQERFTKPERSVRLITFCW	fig|127906.87.peg.104	VCC0104	VC0122	cyaA	Adenylate cyclase (EC 4.6.1.1)		
1	CDS	111593	111279	-	atgaacgaaactgaatttcatca	MNETEFHQLVDSQLERIEAAID	fig|127906.87.peg.105	VCC0105	VC0123	cyaY	Frataxin homolog CyaY, facilitates Fe-S cluster assembly, interacts with IscS		
1	CDS	111669	111785	+	atgaaaaagaaattcactgcac	MKKKFTALFLLSALALAGCGQ	fig|127906.87.peg.106	VCC0106	VC0124	lppL	Diaminopimelate decarboxylase and/or diaminopimelate epimerase leader peptide		Limited query cover between C6706 and N16961
1	CDS	111818	113071	+	ttggattacttcaattatcaggaag	MDYFNYQEDGQLWAEQVPLA	fig|127906.87.peg.107	VCC0107	VC0125	lysA	Diaminopimelate decarboxylase (EC 4.1.1.20)		
1	CDS	113083	113913	+	atgcatttccattttctaaaatgca	MHFHFSKMHGLGNDFMVVDC	fig|127906.87.peg.108	VCC0108	VC0126	dapF	Diaminopimelate epimerase (EC 5.1.1.7)		
1	CDS	113927	114625	+	ttgtctcaggtaacggcggatgc	MSQVTADALTAQVVAEYLYEH	fig|127906.87.peg.109	VCC0109	VC0127		Protein of unknown function DUF484		
1	CDS	114594	115529	+	atgaagaacgacgaacgcacc	MKNDERTPLPDALAQPLERFY	fig|127906.87.peg.110	VCC0110	VC0128	XerC	Site-specific tyrosine recombinase XerC		
1	CDS	115532	116251	+	atgctgttttaccgcccattggctt	MLFYRPLASIQALTFDLDDTLY	fig|127906.87.peg.111	VCC0111	VC0129	yigB	2-haloalkanoic acid dehalogenase (EC 3.8.1.2)		
1	CDS	116361	118850	+	atgtttacggtctcgcgtctcatcc	MFTVSRLIPDLASANQVLETMI	fig|127906.87.peg.112	VCC0112	VC0130		Sensory box/GGDEF family protein		
1	CDS	119479	118883	-	atgaatgcggcgtgtccggactg	MNAACPDCGLKFQCLCSAVP	fig|127906.87.peg.113	VCC0113	VC0131		Uncharacterized conserved protein YfiP, contains DTW domain		
1	CDS	121020	119476	-	atgagagccttgccctctgcgcc	MRALPFCAMSLAMLLQACTNT	fig|127906.87.peg.114	VCC0114	VC0132		Predicted membrane protein		
1	CDS	122131	121304	-	atgaccacttcagcacacaaac	MTTSAHKHPYKIVASDLDGTLI	fig|127906.87.peg.115	VCC0115	VC0134	yigL	HAD phosphatase YigL		
1	CDS	123262	122246	-	atgaaccctgaaaaaaatcttta	MNPEKNLYPYTQESSLEQALN	fig|127906.87.peg.116	VCC0116	VC0135	ytpA/lypA	Lysophospholipase L2 (EC 3.1.1.5)		
1	CDS	123337	123954	+	atggatatccatgtttggcttgcct	MDIHVWLAYLLTAVVFSLAPGS	fig|127906.87.peg.117	VCC0117	VC0136	rhtB	Homoserine/homoserine lactone efflux protein		
1	CDS	123974	125200	+	atgtacacaacttatgtagcacg	MYTTYVARQPILNAKRHTLGY	fig|127906.87.peg.118	VCC0118	VC0137		Predicted signal transduction protein	EAL-domain protein	
1	CDS	125850	125380	-	atggcaacgaatttgattggtcttg	MATNLIGLDTTQSQKLANALNI	fig|127906.87.peg.119	VCC0119	VC0139	dps	DNA protection during starvation protein		
1	CDS	126349	126020	-	atgcccttcactttattgatcagttt	MPFTLLISLALLIIAAVWFFSHF	fig|127906.87.peg.120	VCC0120	VC0140		hypothetical protein	DUF2500 family protein	
1	CDS	126554	126922	+	atgtcagaaattgagcaagttgtt	MSEIEQVVLRTRKLETLLREQY	fig|127906.87.peg.121	VCC0121	VC0142		hypothetical protein	DUF4145 domain-containing protein	
1	CDS	127425	127682	+	gtgtttttatcctttatctgtttttatatc	MFLSFICFYIFKNGSYFSFICLV	fig|127906.87.peg.122	VCC0122	Not present		hypothetical protein		No hits on patric BLAST
								Not present	VC0143		hypothetical protein present in N16961 according to KEGG		
1	CDS	127799	128428	+	atgagcatgttgagctggatttctc	MSMLSWISVVLSGFISISAYEN	fig|127906.87.peg.123	VCC0123	VC0144		Putative inner membrane protein	lysoplasmalogenase	
1	CDS	128743	128477	-	atgaaagcactactctggttagc	MKALLWLAKAIGFVWLVLLFI	fig|127906.87.peg.124	VCC0124	VC0145		hypothetical protein	DUF1145	
1	CDS	129339	128740	-	atgcaaagacgtcgccagccaa	MQRRRQPNPSQKKAPTGQVF	fig|127906.87.peg.125	VCC0125	VC0146	rsmD	16S rRNA (guanine(966)-N(2))-methyltransferase (EC 2.1.1.171)		
1	CDS	129543	130724	+	atgacggaaaaaaagaagcgt	MTEKKKRGLLSWLGFGDEEKI	fig|127906.87.peg.126	VCC0126	VC0147	ftsY	Signal recognition particle receptor FtsY		
1	CDS	130773	131447	+	gtgatcaggtttcagcaagtcag	MIRFQQVSKAYRGGRQALQKI	fig|127906.87.peg.127	VCC0127	VC0148	ftsE	Cell-division-associated, ABC-transporter-like signaling protein FtsE		
1	CDS	131437	132429	+	atggccgttaagccgggcaatc	MAVKPGNQKISKTTKSTKSKP	fig|127906.87.peg.128	VCC0128	VC0149	ftsX	Cell-division-associated, ABC-transporter-like signaling protein FtsX		
1	CDS	132599	133459	+	atgactaaccaagcgtatccaa	MTNQAYPMALVSQDSLDSYIR	fig|127906.87.peg.129	VCC0129	VC0150	rpoH	RNA polymerase sigma factor RpoH		
1	repeat	133526	133649	+	tatataccttcctacttgaagctgcagcggtgttggctacgttcgttcacccca		fig|127906.87.repeat.4				repeat region		
1	CDS	135061	133661	-	atgggacagaaaaaccacttg	MGQKNHFDVIVIGSGPGGEG	fig|127906.87.peg.130	VCC0130	VC0151	sthA	Soluble pyridine nucleotide transhydrogenase (EC 1.6.1.1)		
1	CDS	135369	136010	+	atgaaatcgttgggaatccgagg	MKSLGIRAQQKEKTRRSLIDA	fig|127906.87.peg.131	VCC0131	VC0152	fabR	Unsaturated fatty acid biosythesis repressor FabR, TetR family		
1	CDS	136050	136421	+	atgtcgagagaaaacaacgttc	MSRENNVQSTRAERKTLILAL	fig|127906.87.peg.132	VCC0132	VC0153	yijD	Inner membrane protein YijD		
1	CDS	137567	136458	-	atggcgactcttgatgtaaaccc	MATLDVNPELYQAQLADKIAR	fig|127906.87.peg.133	VCC0133	VC0154	trmA	tRNA (uracil(54)-C5)-methyltransferase (EC 2.1.1.35) @ tmRNA (uracil(341)-C5)-methyltransferase		
1	CDS	137965	139800	+	atgcaaaaatcagcactggcga	MQKSALAIALASLLTPISYLHA	fig|127906.87.peg.134	VCC0134	VC0156	btuB	Outer membrane vitamin B12 receptor BtuB		
1	CDS	141588	139873	-	atgaataacttcttttcatccaagg	MNNFFSSKDEIVIGVLNMIVVS	fig|127906.87.peg.135	VCC0135	VC0157		Alkaline serine exoprotease A precursor (EC 3.4.21.-)		
1	CDS	141873	142670	+	gtgtcatctccatcttttcctcgtgt	MSSPSFPRVLIFDSGVGGLSV	fig|127906.87.peg.136	VCC0136	VC0158	murI	Glutamate racemase (EC 5.1.1.3)		
1	CDS	143094	142636	-	atgaaatctgacaaatcgatgtt	MKSDKSMLWIALLAVIGAGILS	fig|127906.87.peg.137	VCC0137	VC0159		RNA-binding protein		
1	repeat	143469	150998	+	aagctctttaacaatataaaccaatcaatctgtgtgggcactcgttgatgataa		fig|127906.87.repeat.5				repeat region		
1	CDS	143500	143619	+	gtgtgggcactcgttgatgataat	MWALVDDNQKRFINELSDHLN	fig|127906.87.peg.138	VCC0138	VC0160		hypothetical protein		
1	rna	143644	145191	+	ttttaattgaagagtttgatcatggctcagattgaacgctggcggcaggcctaa		fig|127906.87.rna.7	VCCr004	VCr004		SSU rRNA ## 16S rRNA, small subunit ribosomal RNA		
1	rna	145258	145333	+	gtcccgttcgtctagaggcctaggacaccgccctttcacggcggtaacaggg		fig|127906.87.rna.8	VCCt004	VCt004		tRNA-Glu-TTC		
1	rna	145621	148509	+	ggttaagtgactaagcgtacacggtggatgcctgggcagtcagaggcgatg		fig|127906.87.rna.9	VCCr005	VCr005		LSU rRNA ## 23S rRNA, large subunit ribosomal RNA		
1	rna	148634	148755	+	ttgcttggcgaccatagcgttttggacccacctgattccatcccgaactcagaa		fig|127906.87.rna.10	VCCr006	VCr006		5S rRNA ## 5S ribosomal RNA		
1	CDS	148946	149143	+	gtgtgggcactcgttgatgataat	MWALVDDNQKRFINELSDHLN	fig|127906.87.peg.139	VC0139	VC2572		hypothetical protein		Limited query/subject cover
1	rna	150723	150798	+	gtcccgttcgtctagaggcctaggacaccgccctttcacggcggtaacaggg		fig|127906.87.rna.11	VCCt005	VCt091		tRNA-Glu-TTC		

1	rna	150818	150892	+	gggtcgttagctcagtggtagagcagttggcttttaaccaattggtcataggttc		fig|127906.87.rna.12	VCCt006	VCt090		tRNA-Lys-TTT		
1	rna	150923	150998	+	gggcgattagctcagttgggagagcacctgccttacaagcaggggtcact		fig|127906.87.rna.13	VCCt007	VCt089		tRNA-Val-TAC		
1	repeat	151003	154735	+	tggggttatagctcagctgggagagcgcctgccttgcacgcaggaggtctgc		fig|127906.87.repeat.6				repeat region		
1	rna	151004	151079	+	ggggttatagctcagctgggagagcgcctgccttgcacgcaggaggtctgc		fig|127906.87.rna.14	VCCt008	VCt088		tRNA-Ala-TGC		
1	rna	151367	154255	+	ggttaagtgactaagcgtacacggtggatgcctgggcagtcagaggcgatg		fig|127906.87.rna.15	VCCr007	VCr020		LSU rRNA ## 23S rRNA, large subunit ribosomal RNA		
1	rna	154380	154501	+	ttgcttggcgaccatagcgttttggacccacctgattccatcccgaactcagaa		fig|127906.87.rna.16	VCCr008	VCr019		5S rRNA ## 5S ribosomal RNA		
1	rna	154524	154600	+	ggagcggtagttcagtcggttagaataccggcctgtcacgccgggggtcgc		fig|127906.87.rna.17	VCCt009	VCt087		tRNA-Asp-GTC		
1	CDS	155847	154843	-	atgataacttcatcactccctttaa	MITSSLPLTDLHRHLDGNIRTC	fig|127906.87.peg.140	VCC0140	VC2751	add	Adenosine deaminase (EC 3.5.4.4)		
1	CDS	158470	155939	-	atgcctttaaaacgccaaattcg	MPLKRQIRLKTAIILPFVLTFLF	fig|127906.87.peg.141	VCC0141	VC2750		c-di-GMP phosphodiesterase (EC 3.1.4.52) => Pdel		
1	CDS	159929	158529	-	atgagtagaggatacgtctgggt	MSRGYVWVVDDDSSIRWVME	fig|127906.87.peg.142	VCC0142	VC2749	glnG/ntrC	Nitrogen regulation protein NR(I), GlnG (=NtrC)		
1	CDS	161003	159939	-	atgagtgtgagtgcagaattaag	MSVSAELSQTIINNQVTSVLILL	fig|127906.87.peg.143	VCC0143	VC2748	glnL	Nitrogen regulation protein NtrB (EC 2.7.13.3)		
1	CDS	161726	161136	-	atgtcatcgtctcgtctgctatggc	MSSSRLLWLLISVLWVPQLAA	fig|127906.87.peg.144	VCC0144	VC2747		hypothetical protein	DUF4124 domain-containing protein	
1	CDS	163313	161904	-	atgtcagtagaaaatgttttatcg	MSVENVLSLIQENEVKFVDLR	fig|127906.87.peg.145	VCC0145	VC2746	glnA	Glutamine synthetase type I (EC 6.3.1.2)		
1	CDS	163458	163781	+	atggttttggtgctggaatgtttgc	MVLVLECLLFDELVHQKDPQL	fig|127906.87.peg.146	VCC0146	VC2745		hypothetical protein		
1	CDS	163801	165630	+	atgaccactccgcaaattgaaa	MTTPQIEKLRNIAIIAHVDHGKT	fig|127906.87.peg.147	VCC0147	VC2744	typA	GTP-binding protein TypA/BipA		
1	CDS	165737	166267	+	atgtcgattgtgattatttctggcgg	MSIVIISGGPGAGKTTLLNALA	fig|127906.87.peg.148	VCC0148	VC2743		Predicted ATPase		
1	CDS	166294	167187	+	atgaaattgactcactcttttattaa	MKLTHSFIKQQARLGLNFFRY	fig|127906.87.peg.149	VCC0149	VC2742	rbn	Inner membrane protein YihY, formerly thought to be RNase BN		
1	CDS	167205	167639	+	gtgatagcactgatccaacgag	MIALIQRVSEAAVRVDGEVVG	fig|127906.87.peg.150	VCC0150	VC2741	dtd	D-aminoacyl-tRNA deacylase (EC 3.1.1.96)		
1	CDS	167712	168638	+	atgtttaaacttattacgccgacc	MFKLITPTTDNQLNKYFHFRW	fig|127906.87.peg.151	VCC0151	VC2740	yjcF	Uncharacterized GNAT family acetyltransferase YiiD		
1	CDS	170566	168680	-	atgagaaaaatactggcgattg	MRKILAIGVAGIALLLILVLAGF	fig|127906.87.peg.152	VCC0152	VC2739		AsmA family protein		
1	CDS	172381	170753	-	atgaccgttatggaacatactaa	MTVMEHTKAAQIDLAQYGITG	fig|127906.87.peg.153	VCC0153	VC2738	pckA	Phosphoenolpyruvate carboxykinase [ATP] (EC 4.1.1.49)		
1	CDS	173608	172718	-	atgacgacacaatcaatggcga	MTTQSMANNMLHRYLFKDLS	fig|127906.87.peg.154	VCC0154	VC2736	HslO	33 kDa chaperonin HslO		
1	CDS	174004	173627	-	atgagttccagcgcagaagagg	MSSSAEEVRLDKWLWAARFY	fig|127906.87.peg.155	VCC0155	VC2735	hslR	Ribosome-associated heat shock protein implicated in the recycling of the 50S subunit (S4 paralog)		
1	CDS	174234	175151	+	atggaatttaaacaacttcctccg	MEFKQLPPLAAWPRLLSQNTL	fig|127906.87.peg.156	VCC0156	VC2734	epsC	General secretion pathway protein C		
1	CDS	175197	177221	+	gtgaaatattggctgaaaaaaa	MKYWLKKSSWLLAGSLLSTPL	fig|127906.87.peg.157	VCC0157	VC2733	pulD/gspD	General secretion pathway protein D		Gene seems to be split in half in N16961 according to blast search on patric without refseq locus tag. On KEGG the gene is annotated as one whole pseudogene.
1	CDS	177218	178729	+	atgaccgaaatggtgatctctcc	MTEMVISPAERQSIRRLPFSFA	fig|127906.87.peg.158	VCC0158	VC2732	epsE	General secretion pathway protein E		
1	CDS	178729	179949	+	atggccgcgtttgaatacaaagc	MAAFEYKALDAKGRHKKGVIE	fig|127906.87.peg.159	VCC0159	VC2731	epsF	General secretion pathway protein F		
1	CDS	179990	180430	+	atgaaaaaaatgcgtaaacaa	MKKMRKQTGFTLLEVMVVVV	fig|127906.87.peg.160	VCC0160	VC2730	epsG	General secretion pathway protein G		
1	CDS	180464	181048	+	atgacagcgacacgcggttttac	MTATRGFTLLEILLVLVLVSAS	fig|127906.87.peg.161	VCC0161	VC2729	epsH	General secretion pathway protein H		
1	CDS	181038	181391	+	atgaagagtaaacgcggttttac	MKSKRGFTLLEVLVALAIFATA	fig|127906.87.peg.162	VCC0162	VC2728	gspI	General secretion pathway protein I		
1	CDS	181378	182043	+	atgtggcgaactaaccaagtat	MWRTNQVSSRQNMAGFTLIE	fig|127906.87.peg.163	VCC0163	VC2727	xcpW/epsJ	General secretion pathway protein J		
1	CDS	182033	183043	+	atgcgggctaaacagcgcggc	MRAKQRGVALIVILLLLAVMVS	fig|127906.87.peg.164	VCC0164	VC2726	epsK	General secretion pathway protein K		
1	CDS	183012	184223	+	gtgagcgagtttctgaccgttcga	MSEFLTVRLSSQKEADIPWLV	fig|127906.87.peg.165	VCC0165	VC2725	epsL	General secretion pathway protein L		
1	CDS	184233	184730	+	atgaaagaattatggctcctgtg	MKELLAPVQAWWRSVTPREC	fig|127906.87.peg.166	VCC0166	VC2724	epsM	General secretion pathway protein M		
1	CDS	184732	185490	+	atgaagcgtgctgttggctatggt	MKRAVGYGLLFSTVLMTSVVV	fig|127906.87.peg.167	VCC0167	VC2723	epsN	General secretion pathway protein N		
1	CDS	186369	185542	-	atgtctacaccacaggatctctc	MSTPQDLSHLLPDVINIARAAC	fig|127906.87.peg.168	VCC0168	VC2722	cysQ	3'(2'),5'-bisphosphate nucleotidase (EC 3.1.3.7)		
1	CDS	186940	186392	-	atgtcatccaaatctttaccagaa	MSSKSLPEILDRETVAKSRLFT	fig|127906.87.peg.169	VCC0169	VC2721	nudE	ADP compounds hydrolase NudE		
1	CDS	187666	187079	-	gtgtcaaatccaattactattactg	MSNPITITESAQSHFAKLLAQG	fig|127906.87.peg.170	VCC0170	VC2720	nfuA	[4Fe-4S] cluster carrier protein NfuA		
1	CDS	188242	187754	-	atgtgtgtcggcgattaccgcttt	MCVGDYRFPLSDAVHQLKYQ	fig|127906.87.peg.171	VCC0171	VC2719		Competence protein F homolog, phosphoribosyltransferase domain; protein YhgH required for utilization of DNA as sole source of carbon and energy		Query cover limited in certain strains
1	CDS	188373	188242	-	ttggcgtctgctctgcctgccaag	MASALPAKLGLSTVIAAHAAVY	fig|127906.87.peg.172	VCC0172	Not annotated as CDS		FIG01203574: hypothetical protein		Partly maps to VC2719 and lacks refseq locus tag
1	CDS	188523	189311	+	atggaaatagacggagaaact	MEIDGETVTMALYWQVSGQG	fig|127906.87.peg.173	VCC0173	VC2718	bioH	Pimeloyl-[acyl-carrier protein] methyl ester esterase BioH (EC 3.1.1.85)		Partly overlaps with VC2719
1	CDS	189473	189943	+	atgctagtgtcaccgaccaatgt	MLVSPTNVSVPLIAPSVNVQT	fig|127906.87.peg.174	VCC0174	VC2717		Putative ATP-dependent Lon protease		
1	CDS	192356	190035	-	atgagcaaagctatctgccacc	MSKAICHQIAQELNVRPEQVIA	fig|127906.87.peg.175	VCC0175	VC2716	rps1	Transcription accessory protein (S1 RNA-binding domain)		
1	CDS	192601	193086	+	gtgaaaactaagctgattaccc	MKTKLITRAGYNKLKQELDYL	fig|127906.87.peg.176	VCC0176	VC2715	greB	Transcription elongation factor GreB		
1	CDS	193224	193946	+	atggtggaaaatcataaagtttta	MVENHKVLVVDDDARLRALLE	fig|127906.87.peg.177	VCC0177	VC2714	ompR	Two-component system response regulator OmpR		
1	CDS	194015	195331	+	atgcgaattcgcagttcattacc	MRIRSSFTQSILLFFSLLIASQI	fig|127906.87.peg.178	VCC0178	VC2713	envZ	Osmolarity sensory histidine kinase EnvZ		
1	CDS	196762	195356	-	atgacacaaccgacttcacgta	MTQPTSRKADLVYQLNDRPPI	fig|127906.87.peg.179	VCC0179	VC2712	xanP	Xanthine permease @ Xanthine:proton symporter XanP		
1	repeat	196847	196978	+	ttatcccctctcttattgaagctgcagcggtgttggctccgttcgttcaccccaat		fig|127906.87.repeat.7				repeat region		
1	CDS	199185	197107	-	atgtcacaactccttcagccgtt	MSQLLSAVPLCELSGVGAKVA	fig|127906.87.peg.180	VCC0180	VC2711	recG	ATP-dependent DNA helicase RecG (EC 3.6.4.12)		
1	CDS	201421	199304	-	ttgtatctattcgatagtctaaaag	MYLFDSLKDVAQEYLTEPQIE	fig|127906.87.peg.181	VCC0181	VC2710	spoT	Guanosine-3',5'-bis(diphosphate) 3'-pyrophosphohydrolase (EC 3.1.7.2) / GTP pyrophosphokinase (EC 2.7.6.5), (p)ppGpp synthetase II		
1	CDS	201725	201453	-	atggcacgcgtaacagttcaag	MARVTVQDAVEKIGNRFDLVL	fig|127906.87.peg.182	VCC0182	VC2709	rpoZ	DNA-directed RNA polymerase omega subunit (EC 2.7.7.6)		
1	CDS	202456	201833	-	atgggcaaaggtactcttatatc	MGKGTLYIVSAPSGAGKSSLIA	fig|127906.87.peg.183	VCC0183	VC2708	gmk	Guanylate kinase (EC 2.7.4.8)		
1	CDS	202824	203495	+	atgaatcacttttacccctgcgca	MNHFTPAQQRKALGYLVLFHL	fig|127906.87.peg.184	VCC0184	VC2706	yhhQ	Putative preQ0 transporter YhhQ		
1	CDS	205296	203593	-	atggatattcaaacttggacgttt	MDIQTWTFILVGITFALYIGIAIV	fig|127906.87.peg.185	VCC0185	VC2705	actP	Acetate permease ActP (cation/acetate symporter)		
1	CDS	205573	205307	-	atggcgttcaaatcatctgaaca	MAFKSSEHAQAYWKENLGIM	fig|127906.87.peg.186	VCC0186	VC2704		FIG152265: Sodium:solute symporter associated protein		
1	CDS	205952	207697	+	gtggacgccgttacgattaacag	MDAVTINSFFMIGALLIGISVLL	fig|127906.87.peg.187	VCC0187	VC2703		K(+)/H(+) antiporter NhaP2		
1	CDS	208425	207796	-	atggactcgacctacaccatcat	MDSTYTIIIADDHPLFRNALFQS	fig|127906.87.peg.188	VCC0188	VC2702	narL	Two-component transcriptional response regulator, LuxR family		
1	CDS	210400	208598	-	atgcgcacactgatactctgtttt	MRTLILCFSLLFALLTQPAWAI	fig|127906.87.peg.189	VCC0189	VC2701	dsbD/dipZ	Cytochrome c-type biogenesis protein DsbD, protein-disulfide reductase (EC 1.8.1.8)		
1	CDS	212671	210560	-	gtgacagacccttactcgcaca	MTDPYSHSLAINSLYSQVVDL	fig|127906.87.peg.190	VCC0190	VC2700	pulA_2	Pullulanase (EC 3.2.1.41)		Gene contains frameshift. Annotated as pseudogene VC2700 in KEGG. Generates two hits in patric. Because of frameshift?
1	CDS	214158	212668	-	gtgaataatcctacttcaatctct	MNNPTFNLSRVNRVLLPMLAS	fig|127906.87.peg.191	VCC0191	VC2700	pulA_1	Pullulanase (EC 3.2.1.41)		Gene contains frameshift. Annotated as pseudogene VC2700 in KEGG. Generates two hits in patric. Because of frameshift?
1	CDS	215859	214552	-	atgatttgggtcgaactggctatt	MIWVELAIVLLFIFIGARIGGIGI	fig|127906.87.peg.192	VCC0192	VC2699	dcuA	Anaerobic C4-dicarboxylate transporter DcuA		
1	CDS	217438	215987	-	atggctaccctatctgaagcgcc	MATLSEAPKTATPATRVEEDL	fig|127906.87.peg.193	VCC0193	VC2698	aspA	Aspartate ammonia-lyase (EC 4.3.1.1)		
1	CDS	217683	218621	+	atggtgaatcgtgccgattcagc	MVNRADSARLLSDQDLQGYV	fig|127906.87.peg.194	VCC0194	VC2697		GGDEF domain protein		
1	CDS	218713	219198	+	gtgttcccgatactactgttttattt	MFPILLFLFIAVPVIEIALFIQVG	fig|127906.87.peg.195	VCC0195	VC2696	fxsA	Protein affecting phage T7 exclusion by the F plasmid		
1	CDS	219708	219232	-	atgtttgatatcgctctctacgaac	MFDIALYEPEIAPNTGNIIRLCA	fig|127906.87.peg.196	VCC0196	VC2695	trmL	tRNA (cytidine(34)-2'-O)-methyltransferase (EC 2.1.1.207)		
1	CDS	220378	219761	-	atgccccatctatttcccgatctac	MPHLFPDLPYAYDALEPYIDTH	fig|127906.87.peg.197	VCC0197	VC2694	sodA	Superoxide dismutase [Mn] (EC 1.15.1.1)		
1	CDS	221905	220511	-	atgcgtctaccgttaccgaaattg	MRLPLPKLNNLYGRIFAIFWFT	fig|127906.87.peg.198	VCC0198	VC2693	cpxA	Copper sensory histidine kinase CpxA		
1	CDS	222591	221905	-	atggcacatatcctttgatcgatg	MAHILLIDDDTELTSLLTEVLQY	fig|127906.87.peg.199	VCC0199	VC2692	phoP/cpxR	Copper-sensing two-component system response regulator CpxR		
1	CDS	222779	223282	+	atgaaacttgccaaaaaaatga	MKLAKKMILAAAVLPLTLGTTA	fig|127906.87.peg.200	VCC0200	VC2691	cpxP	P pilus assembly/Cpx signaling pathway, periplasmic inhibitor/zinc-resistance associated protein		
1	CDS	223483	224388	+	atgaaaaaagaatacgctcgtt	MKKEYARLVTMAAWTATAVA	fig|127906.87.peg.201	VCC0201	VC2690	fieF	Ferrous-iron efflux pump FieF		
1	CDS	224651	225613	+	atgattaaaaaaatcggtgtttg	MIKKIGVLTSGGDAPGMNAAI	fig|127906.87.peg.202	VCC0202	VC2689	pfkA	6-phosphofructokinase (EC 2.7.1.11)		
1	CDS	226697	225690	-	atgaaacgcgatttagcaatggc	MKRDLAMAFSRVTEGAALAG	fig|127906.87.peg.203	VCC0203	VC2688	glpX	Fructose-1,6-bisphosphatase, GlpX type (EC 3.1.3.11)		
1	CDS	227018	227260	+	atgtcttttgaagtattagaaaaa	MSFEVLEKLEAKIQTAVDTIAL	fig|127906.87.peg.204	VCC0204	VC2686	zapB	Cell division protein ZapB		
1	CDS	228210	227323	-	atgggatacacacacgctagcc	MGYTHASHIDALNQNIAELSDI	fig|127906.87.peg.205	VCC0205	VC2685	metF	5,10-methylenetetrahydrofolate reductase (EC 1.5.1.20)		
1	CDS	230908	228497	-	atgacccatccccgtcaattgca	MTHPRQLHKFGGSSLADPEC	fig|127906.87.peg.206	VCC0206	VC2684	metL	Aspartokinase (EC 2.7.2.4) / Homoserine dehydrogenase (EC 1.1.1.3)		
1	CDS	232087	230921	-	atgaccacacgcaagccagcc	MTTRKPATIAVRTGIESDTQYH	fig|127906.87.peg.207	VCC0207	VC2683	metB	Cystathionine gamma-synthase (EC 2.5.1.48)		
1	CDS	232355	232672	+	atggcagactggaatggcgaat	MADWNGEYISPYAEHGKKSE	fig|127906.87.peg.208	VCC0208	VC2682	metJ	Methionine repressor MetJ		
1	CDS	234060	232783	-	atgtccgatgacaatcgcctcaa	MSDDNRLNPTPEEQFRQRAL	fig|127906.87.peg.209	VCC0209	VC2681	maeB	NADP-dependent malic enzyme (EC 1.1.1.40)		
1	CDS	234270	234127	-	atgaattggacactcacttatctg	MNWTLTYLHIKPFELAYSGYF	fig|127906.87.peg.210	VCC0210	VC2680		FIG01199906: hypothetical protein		
1	CDS	234586	234368	-	atgaaagctggtatccacccag	MKAGIHPEYKAVNATCSCGNS	fig|127906.87.peg.211	VCC0211	VC2679	rpmE	LSU ribosomal protein L31p @ LSU ribosomal protein L31p, zinc-dependent		
1	CDS	234841	237045	+	atgcgacctccattgcccgagt	MRPSIARVCLPVPLDKSFDYLI	fig|127906.87.peg.212	VCC0212	VC2678	priA	Helicase PriA essential for oriC/DnaA-independent DNA replication		
1	CDS	237351	238358	+	atggcgacaatgaaggatgttg	MATMKDVAQLAGVSTATVSR	fig|127906.87.peg.213	VCC0213	VC2677	cytR	Transcriptional (co)regulator CytR		
1	CDS	238381	238494	+	atgcttgaaaggtttgaactcggt	MLERFELGSFFRSCRSAEIGD	fig|127906.87.peg.214	VCC0214	Not annotated as CDS		FIG01200353: hypothetical protein		
1	CDS	238530	239081	+	gtggctaatagagattatgtaag	MANRDYVRGGRSPKKPTRRK	fig|127906.87.peg.215	VCC0215	VC2676	ftsN	Cell division protein		
1	CDS	239218	239775	+	gtgactactatcgtatcagtgcgt	MTTIVSVRRNNKVVIAGDGQV	fig|127906.87.peg.216	VCC0216	VC2675	hslV	ATP-dependent protease subunit HslV (EC 3.4.25.2)		
1	CDS	239841	241172	+	atgtctgaaatgactccccgcga	MSEMTPREIVSELNRHIIGQDH	fig|127906.87.peg.217	VCC0217	VC2674	hslU	ATP-dependent hsl protease ATP-binding subunit HslU		
1	CDS	241276	242193	+	atgaataactctgtgcaaatttgg	MNNSVQIWLDAARPKTLPLAL	fig|127906.87.peg.218	VCC0218	VC2673	menA	1,4-dihydroxy-2-naphthoate polyprenyltransferase (EC 2.5.1.74)		
1	CDS	242268	242783	+	atggaatacaatacttctgcgctc	MEYNTSALCDIYLDQVDVVEP	fig|127906.87.peg.219	VCC0219	VC2672	rraA	Ribonuclease E inhibitor RraA		
1	CDS	244252	242870	-	atgctaggcaccttgttttccttcgt	MLGTLFSFVAQLMHDKHGLLS	fig|127906.87.peg.220	VCC0220	VC2671	mepA_2	Na+-driven multidrug efflux pump		
1	CDS	245143	244370	-	atgcgtcgtcctgttgtgatgggta	MRRPVVMGNWKLNGSKAMV	fig|127906.87.peg.221	VCC0221	VC2670	tpiA	Triosephosphate isomerase (EC 5.3.1.1)		
1	CDS	245387	245737	+	atgccaaacttagtcatggaata	MPNLVMEYSNSVDERINVQG	fig|127906.87.peg.222	VCC0222	VC2669	hpcD	5-carboxymethyl-2-hydroxymuconate delta-isomerase (EC 5.3.3.10)		
1	CDS	245757	246197	+	atgtcgttacaaacactgttgtttt	MSLQTLLFSFQGRIGRQAFWL	fig|127906.87.peg.223	VCC0223	VC2668		Predicted membrane protein	DUF805 domain containing protein	
1	CDS	246683	246324	-	atgacgcccccacactcacccg	MTPPHSPEPCNQVLPPFDEL	fig|127906.87.peg.224	VCC0224	VC2667		hypothetical protein	DUF3135 domain containing protein	
1	CDS	247439	246822	-	atgaaaacggttgaccgcattct	MKTVDRILHTIKREGTVTAKQL	fig|127906.87.peg.225	VCC0225	VC2666		Predicted transcriptional regulator		
1	CDS	247799	248089	+	atgaatattcgtccattacatgac	MNIRPLHDRVIVERQEVESKS	fig|127906.87.peg.226	VCC0226	VC2665	groES	Heat shock protein 10 kDa family chaperone GroES		
1	CDS	248143	249777	+	atggctgctaaagacgtacgttt	MAAKDVRFGNDARVKMLEGV	fig|127906.87.peg.227	VCC0227	VC2664	groEL	Heat shock protein 60 kDa family chaperone GroEL		
1	CDS	249988	250548	+	atgaacaagccgtttgctttgatg	MNKPFALMALTAAMAAPAALA	fig|127906.87.peg.228	VCC0228	VC2662		hypothetical protein	phaC PHA synthase	
1	CDS	251607	250585	-	atgccgcacatcataacccgaa	MPHIITRKVISVEQNWLQQLAN	fig|127906.87.peg.229	VCC0229	VC2661	epmB	Lysine 2,3-aminomutase (EC 5.4.3.2)		
1	CDS	251640	252206	+	atggctactgttagcacgaatga	MATVSTNEFKGGLKIMLDNEP	fig|127906.87.peg.230	VCC0230	VC2660	efp	Translation elongation factor P		
1	CDS	252682	252320	-	gtgattaataccaacccaaaac	MINTNPKRSDEPVWWSLFGA	fig|127906.87.peg.231	VCC0231	VC2659	frdD	Fumarate reductase subunit D		
1	CDS	253076	252693	-	atgagtaaccgtaaacctatgtt	MSNRKPYVREMKRTWWKDH	fig|127906.87.peg.232	VCC0232	VC2658	frdC	Fumarate reductase subunit C		
1	CDS	253837	253079	-	atgtcagcacaacgtattcaaa	MSAQRIQKVDILRYDPANDAE	fig|127906.87.peg.233	VCC0233	VC2657	frdB	Fumarate reductase iron-sulfur protein (EC 1.3.5.4)		
1	CDS	255645	253837	-	gtgcaaattctcaccacagatat	MQILTTDIAVIGAGGAGLRTAIA	fig|127906.87.peg.234	VCC0234	VC2656	frdA	Fumarate reductase flavoprotein subunit (EC 1.3.5.4)		
1	CDS	255797	255910	+	ttgaagaatgaaaaattgattctc	MKNEKLILLGFCFGIANLSILFA	fig|127906.87.peg.235	VCC0235	Not annotated as CDS		FIG01203668: hypothetical protein	putative membrane protein	
1	CDS	255954	256928	+	atgactaattccgattggatgccg	MTNSDWMPTASISQLKQRATI	fig|127906.87.peg.236	VCC0236	VC2655	epmA/yjeA	Translation elongation factor P Lys34--(R)-beta-lysine ligase		
1	CDS	257058	257492	+	atgcaagagtacattgagttttttc	MQEYIEFFQQNMILSLAWIGIL	fig|127906.87.peg.237	VCC0237	VC2654	glpE_1	Rhodanese-related sulfurtransferase YibN		
1	CDS	257659	258123	+	atggctgaagcagcacaagcc	MAEAAQAPQQQFAIQRIYLKD	fig|127906.87.peg.238	VCC0238	VC2653	secB	Protein-export protein SecB (maintains pre-export unfolded state)		
					atgcacatcgcggtggcgctgtgcattttattcttactgaaaactcaatcgcgcc		From N16961	Not annotated as CDS	VC2652				Annotated as VC2652 in KEGG as a hypothetical prtien. Annotated as a part of VC2652 SecB according to patric.
1	CDS	258313	259347	+	atgagcgaaacacagaaccaa	MSETQNHNSYGKPVEMTVIGA	fig|127906.87.peg.239	VCC0239	VC2651	gpsA	Glycerol-3-phosphate dehydrogenase [NAD(P)+] (EC 1.1.1.94)		
					atgcttaataatggtggcatggtaaaaactggcgagcatcggctcttgctcgg		From N16961	Not annotated as CDS	VC2650				Annotated as VC2650 in KEGG as a hypothetical prtien. Annotated as a part of VC2651 abd VC2649 in the other VC genomes according to patric.
1	CDS	259420	260241	+	atgaaacagtgcgcccacacc	MKQCAHTKVWQTIVAEAREQ	fig|127906.87.peg.240	VCC0240	VC2649	cysE	Serine acetyltransferase (EC 2.3.1.30)		
1	CDS	260896	260357	-	atgtcattaccacacgttatcctta	MSLPHVILTVLSTRDATGYDIT	fig|127906.87.peg.241	VCC0241	VC2647		Transcriptional regulator, PadR family		
1	CDS	263885	261255	-	atgaacgaaaaatacgctgcg	MNEKYAALKSNVSMLGRLLGC	fig|127906.87.peg.242	VCC0242	VC2646	ppc	Phosphoenolpyruvate carboxylase (EC 4.1.1.31)		
1	CDS	265228	264092	-	atgccgttacccagttccttgagt	MPLPSFLEVYEGLISTSSISSTI	fig|127906.87.peg.243	VCC0243	VC2645	argE	N-acetylornithine deacetylase (EC 3.5.1.16)		
1	CDS	265384	266385	+	atgttaaaaaccaccatcatcgg	MLKTTIIGASGYTGAELALMVH	fig|127906.87.peg.244	VCC0244	VC2644	argC	N-acetyl-gamma-glutamyl-phosphate reductase (EC 1.2.1.38)		
1	CDS	266395	267183	+	atgtcagatcttaagctcagtcct	MSDLKLSPLVIKLGGAALDCAI	fig|127906.87.peg.245	VCC0245	VC2643	argB	N-acetylglutamate kinase (EC 2.7.2.8)		
1	CDS	267259	268473	+	atgagtaaagtacaagttaaaa	MSKVQVKKVVVAYSGGLDTS	fig|127906.87.peg.246	VCC0246	VC2642	argG	Argininosuccinate synthase (EC 6.3.4.5)		
1	repeat	268521	268632	+	atcccctcctaattgaagttgcagcggtgttggctacgttcattcaccccaatc		fig|127906.87.repeat.8				repeat region		
1	CDS	268706	270082	+	atggcattatggggcggaagatt	MALWGGRFTQAADSRFKSFN	fig|127906.87.peg.247	VCC0247	VC2641	argH	Argininosuccinate lyase (EC 4.3.2.1)		
1	CDS	270587	270333	-	atggcttgtggagagtgtagcga	MACGECSEHWFWKKIGRCQ	fig|127906.87.peg.248	VCC0248	VC2640		hypothetical protein	DUF3624 domain-containing protein [Vibrio cholerae]	
1	CDS	272276	270822	-	atgaaacagattcacgttgatgtc	MKQIHVDVAVIGGGTAGLGAY	fig|127906.87.peg.249	VCC0249	VC2638	lpd	Dihydrolipoamide dehydrogenase (EC 1.8.1.4)		
1	CDS	273079	272348	-	atgtttacatctaaagaaggtcaa	MFTSKEGQTIPQVTFPTRQGD	fig|127906.87.peg.250	VCC0250	VC2637		Hybrid peroxiredoxin hyPrx5 (EC 1.11.1.15)		
1	CDS	273219	274112	+	atgaacattcgtgatttgaatact	MNIRDFEYLVALADHKHFRKA	fig|127906.87.peg.251	VCC0251	VC2636	oxyR_2	Hydrogen peroxide-inducible genes activator => OxyR		
1	CDS	276663	274186	-	gtgaagttcataaagcgtttatta	MKFIKRLLVFSLICIILGVTTIFG	fig|127906.87.peg.252	VCC0252	VC2635	mrcA	Multimodular transpeptidase-transglycosylase (EC 2.4.1.129) (EC 3.4.-.-)		
1	CDS	276806	277822	+	atgggtaaatcattagttacgggt	MGKSLVTGIDIGHHSIKAAVLK	fig|127906.87.peg.253	VCC0253	VC2634	pilM	Type IV pilus biogenesis protein PilM		
1	CDS	277812	278396	+	atgttgcataaggtcaacttactg	MLHKVNLLPWRDARREAHKR	fig|127906.87.peg.254	VCC0254	VC2633	pilN	Type IV pilus biogenesis protein PilN		
1	CDS	278389	278979	+	atggctagtctacaagagctaga	MASLQELELDEISEWPLLPQL	fig|127906.87.peg.255	VCC0255	VC2632	pilO	Type IV pilus biogenesis protein PilO		
1	CDS	278969	279487	+	atgaaaaataagccacttggatt	MKNKPLGLWLMSLLVLVGCKA	fig|127906.87.peg.256	VCC0256	VC2631	pilP	Type IV pilus biogenesis protein PilP		
1	CDS	279513	281228	+	atgaaaaatggattgaaaactta	MKNGLKTYVAQTWLTLWVGL	fig|127906.87.peg.257	VCC0257	VC2630	pilQ	Type IV pilus biogenesis protein PilQ		
1	CDS	281415	281939	+	atggctgagaaacgcaatatttt	MAEKRNIFLVGPMGAGKSTIG	fig|127906.87.peg.258	VCC0258	VC2629	aroK	Shikimate kinase I (EC 2.7.1.71)		
1	CDS	281967	283052	+	atggagcggattacggtcaactt	MERITVNLGERSYPISIGAGLF	fig|127906.87.peg.259	VCC0259	VC2628	aroB	3-dehydroquinate synthase (EC 4.2.3.4)		
1	CDS	283107	284582	+	atgagcttggcacatgaactgga	MSLAHELDLESQTELLERLQL	fig|127906.87.peg.260	VCC0260	VC2627		Septum-associated cell division protein DamX		
1	CDS	284652	285485	+	atgaaaaagcagcgcgcctttc	MKKQRAFLKWAGGKYSLVED	fig|127906.87.peg.261	VCC0261	VC2626	dam	Methyl-directed repair DNA adenine methylase (EC 2.1.1.72)		
1	CDS	285612	286286	+	atgaaagatttctcattgcacca	MKDFLIAPSILSADFARLGEDV	fig|127906.87.peg.262	VCC0262	VC2625	rpe	Ribulose-phosphate 3-epimerase (EC 5.1.3.1)		
1	CDS	286306	286986	+	ttgaaatccataaaattaattgctt	MKSIKLIAFDLDGTLLDSVPDL	fig|127906.87.peg.263	VCC0263	VC2624	gph	Phosphoglycolate phosphatase (EC 3.1.3.18)		
1	CDS	287114	288130	+	atgagcaaacccattgtattgag	MSKPIVLSGVQPSGELSIGNYI	fig|127906.87.peg.264	VCC0264	VC2623	trpS	Tryptophanyl-tRNA synthetase (EC 6.1.1.2)		
1	CDS	288296	289060	+	atgagattcatgttgctgttgatgc	MRFMLLLMLVGMAHGALANP	fig|127906.87.peg.265	VCC0265	VC2622		ABC-type amino acid transport/signal transduction system, periplasmic component/domain		
1	CDS	289330	291939	+	atggaaatgagaacaaccccg	MEMRTTPNLTRSTLALAISFGL	fig|127906.87.peg.266	VCC0266	VC2621	Xds	Extracellular deoxyribonuclease Xds		
1	CDS	293425	292013	-	atgacagttcagactttacgcca	MTVQTFTPNQTTTLETAKKTIA	fig|127906.87.peg.267	VCC0267	VC2620	rimO	Ribosomal protein S12p Asp88 (E. coli) methylthiotransferase (EC 2.8.4.4)		
1	CDS	293679	294260	+	atgctactgatgatcgataactac	MLLMIDNYDSFTYNLYQYFCE	fig|127906.87.peg.268	VCC0268	VC2619	trpG_2/pabA	Para-aminobenzoate synthase, amidotransferase component (EC 2.6.1.85)		
1	CDS	294658	295869	+	atgacggtggaaatgagtgtag	MTVEMSVDRSSFDEVMVPCY	fig|127906.87.peg.269	VCC0269	VC2618	argD/aruC	Succinylornithine transaminase (EC 2.6.1.81)		
1	CDS	296009	297028	+	atgctagttgttcgtccgattagca	MLVVRPISMADYEALHTCAVE	fig|127906.87.peg.270	VCC0270	VC2617	astA	Arginine N-succinyltransferase (EC 2.3.1.109)		
1	CDS	297038	298495	+	atgacacattggatagccggtga	MTHWIAGEWVAGTGEKLESH	fig|127906.87.peg.271	VCC0271	VC2616	astD	Succinylglutamic semialdehyde dehydrogenase (EC 1.2.1.71)		
1	CDS	298638	299429	+	atgacgccaacagctttattccaa	MTPTALFQSLWNDYIERLCPS	fig|127906.87.peg.272	VCC0272	VC2615		FIG008480: zinc-binding hydrolase		
1	CDS	300140	299508	-	atggttctaggtaaacctcaaac	MVLGKPQTDPTLEWFLSHCH	fig|127906.87.peg.273	VCC0273	VC2614	crp	Cyclic AMP receptor protein		
1	CDS	301252	300383	-	atgtcagccaaacatccgattat	MSAKHPIIAVTGSSGAGTTTTS	fig|127906.87.peg.274	VCC0274	VC2613	cfxP/prkB	Phosphoribulokinase homolog, function unknown		
1	CDS	301558	301349	-	atgatcattccatggcaagagat	MIIPWQEIAPETLDNLIREFVLR	fig|127906.87.peg.275	VCC0275	VC2612		UPF0270 protein YheU		
1	CDS	302675	301686	-	atgatcacgtttattgctgctaagg	MITFIAAKGLKNPHLQTLLPRL	fig|127906.87.peg.276	VCC0276	VC2610		Putative esterase YheT functionally coupled to phosphoribulokinase homolog		
1	CDS	303157	302672	-	atgagtgatgagcacgccaaa	MSDEHAKPRLTLERLWQFSL	fig|127906.87.peg.277	VCC0277	VC2609		FIG01200117: hypothetical protein		
1	CDS	305074	303158	-	atgattacctttctgagattcagc	MITFSEIQLLRGGKPLLEQASA	fig|127906.87.peg.278	VCC0278	VC2608	yheS	Bis-ABC ATPase YheS		
1	CDS	305207	305788	+	ttggtcaaccgagagcaaacga	MVNREQTKAKVLVVYAHPDP	fig|127906.87.peg.279	VCC0279	VC2607	kefF	Glutathione-regulated potassium-efflux system ancillary protein KefG		
1	CDS	305769	307571	+	atgacgccactgaccagtgattt	MTPLTSDFLSSSVVFLSAAVIA	fig|127906.87.peg.280	VCC0280	VC2606	kefB	Glutathione-regulated potassium-efflux system protein KefB		
1	CDS	307581	307781	+	gtgaaacagaaaaaacgtttta	MKQKKRFIAGANCPQCKTAD	fig|127906.87.peg.281	VCC0281	VC2605		Cytoplasmic protein, probably associated with glutathione-regulated potassium-efflux		
1	CDS	307873	308478	+	atgaaaattgaaaaaaacacg	MKIEKNTVASLAYQLTIEDGVV	fig|127906.87.peg.282	VCC0282	VC2604	slyD	FKBP-type peptidyl-prolyl cis-trans isomerase SlyD (EC 5.2.1.8)		
1	CDS	308641	309621	+	atgacacagccttttgcgattgcc	MTQPFAIAIHGGAGTILREQMS	fig|127906.87.peg.283	VCC0283	VC2603	iaaA	Isoaspartyl aminopeptidase (EC 3.4.19.5) @ Asp-X dipeptidase		
1	CDS	309749	311047	+	atgggtaataacgttgtcgttcttg	MGNNVVVLGTQWGDEGKGK	fig|127906.87.peg.284	VCC0284	VC2602	purA	Adenylosuccinate synthetase (EC 6.3.4.4)		
1	CDS	311255	311890	+	atgaagctacgaacggtagccg	MKLRTVAASLLLAWHSSYNPA	fig|127906.87.peg.285	VCC0285	VC2601	motX	Sodium-type flagellar protein MotX		Limited query cover for certain strains
1	CDS	313746	311917	-	atgttattgatgataaaaaagaa	MLLMIKKNIEWLFSHFILNAVLL	fig|127906.87.peg.286	VCC0286	VC2600	yejM_2	Predicted hydrolase of alkaline phosphatase superfamily		
1	CDS	313929	316394	+	atgtctgataccacacatcttgat	MSDTTHLDPFADREADNYDNI	fig|127906.87.peg.287	VCC0287	VC2599	rnr/vavB	3'-to-5' exoribonuclease RNase R		
1	CDS	316402	317145	+	atgagtaatgaattgatttatggc	MSNELIYGIHAVNAVLQRDPAI	fig|127906.87.peg.288	VCC0288	VC2598	rlmB_2	23S rRNA (guanosine(2251)-2'-O)-methyltransferase (EC 2.1.1.185)		
1	CDS	317616	317927	+	atgcagaaccaacgtatccgtat	MQNQRIRIRLKAFDYKLIDAST	fig|127906.87.peg.289	VCC0289	VC2597	rpsJ	SSU ribosomal protein S10p (S20e)		
1	CDS	317942	318571	+	atgattggtctaatcggtcgtaaa	MIGLIGRKVGMTRVFTEDGVS	fig|127906.87.peg.290	VCC0290	VC2596	rplC	LSU ribosomal protein L3p (L3e)		
1	CDS	318588	319190	+	atggaattgatggttaaaggtgc	MELMVKGANALTVSETTFGRE	fig|127906.87.peg.291	VCC0291	VC2595	rplD	LSU ribosomal protein L4p (L1e)		

1	CDS	319187	319489	+	atgatccgtgaagagcgtctact	MIREERLLKVLRAPHISEKATM	fig|127906.87.peg.292	VCC0292	VC2594	rplW	LSU ribosomal protein L23p (L23Ae)		
1	CDS	319506	320330	+	atggctattgttaaatgtaagccg	MAIVKCKPTSAGRRHVVKVVN	fig|127906.87.peg.293	VCC0293	VC2593	rplB	LSU ribosomal protein L2p (L8e)		
1	CDS	320351	320629	+	atgccacgttctctcaagaaagg	MPRSLKKGPFIDLHLLKKVEK	fig|127906.87.peg.294	VCC0294	VC2592	rpsS	SSU ribosomal protein S19p (S15e)		
1	CDS	320640	320972	+	atggaagctatcgctaaacataa	MEAIAKHNFARISPQKARLVAD	fig|127906.87.peg.295	VCC0295	VC2591	rplV	LSU ribosomal protein L22p (L17e)		
1	CDS	320992	321693	+	atgggtcagaaagtacatccaa	MGQKVHPNGIRLGIVKPWNAT	fig|127906.87.peg.296	VCC0296	VC2590	rpsC	SSU ribosomal protein S3p (S3e)		
1	CDS	321705	322115	+	atgctacaacctaaacgtactaa	MLQPKRTKFRKVQTGRNRGL	fig|127906.87.peg.297	VCC0297	VC2589	rplP	LSU ribosomal protein L16p (L10e)		
1	CDS	322115	322306	+	atgaaagcacaagatctacgcg	MKAQDLREKSVEELNSELLNL	fig|127906.87.peg.298	VCC0298	VC2588	rpmC	LSU ribosomal protein L29p (L35e)		
1	CDS	322306	322560	+	atgagcgacaaaattcgtactca	MSDKIRTQLGRVVSDKMDKSI	fig|127906.87.peg.299	VCC0299	VC2587	rpsQ	SSU ribosomal protein S17p (S11e)		
1	CDS	322723	323094	+	atgatccaaatgcaaactatgct	MIQMQTMLDAADNSGARSVM	fig|127906.87.peg.300	VCC0300	VC2586	rplN	LSU ribosomal protein L14p (L23e)		
1	CDS	323107	323424	+	atggcagctaaaatccgtcaaa	MAAKIRQNDEVIVLAGKDKGK	fig|127906.87.peg.301	VCC0301	VC2585	rplX	LSU ribosomal protein L24p (L26e)		
1	CDS	323449	323988	+	atggcgaaactgcatgattacta	MAKLHDYYKSSVVAELTKQFS	fig|127906.87.peg.302	VCC0302	VC2584	rlpE	LSU ribosomal protein L5p (L11e)		
1	CDS	324006	324311	+	atggctaaacaatcaatgaaag	MAKQSMKAREEKRAKLVAQF	fig|127906.87.peg.303	VCC0303	VC2583	rpsN	SSU ribosomal protein S14p (S29e) @ SSU ribosomal protein S14p (S29e), zinc-independent		
1	CDS	324341	324733	+	atgagcatgcaagatccgattt	MSMQDPISDMLTRIRNGQAAN	fig|127906.87.peg.304	VCC0304	VC2582	rpsH	SSU ribosomal protein S8p (S15Ae)		
1	CDS	324748	325281	+	atgtctcgtgttgctaaagcacct	MSRVAKAPVAIPAGVEVKLNG	fig|127906.87.peg.305	VCC0305	VC2581	rplF	LSU ribosomal protein L6p (L9e)		
1	CDS	325291	325644	+	atggataagaaagcatctcgca	MDKKASRIRRATRARRKIAEL	fig|127906.87.peg.306	VCC0306	VC2580	rplR	LSU ribosomal protein L18p (L5e)		
1	CDS	325659	326162	+	atggctaaagaacaacaagttc	MAKEQQVQANDLQEKLIAVNR	fig|127906.87.peg.307	VCC0307	VC2579	rpsE	SSU ribosomal protein S5p (S2e)		
1	CDS	326169	326345	+	atggcaactattaaagtaactca	MATIKVTQTKSSIGRLPKHKAT	fig|127906.87.peg.308	VCC0308	VC2578	rpmD	LSU ribosomal protein L30p (L7e)		
1	CDS	326351	326785	+	atgttattgaatactctatcaccg	MLLNTLSPAAGSKHAPKRLGR	fig|127906.87.peg.309	VCC0309	VC2577	rplO	LSU ribosomal protein L15p (L27Ae)		
1	CDS	326806	328140	+	atggctagaaaaccaggacaa	MARKPGQDFRSAQSGLSELK	fig|127906.87.peg.310	VCC0310	VC2576	secY	Protein translocase subunit SecY		
1	CDS	328179	328292	+	atgaaagttcgtgcttccgttaaa	MKVRASVKKICRNCKVIKRNG	fig|127906.87.peg.311	VCC0311	VC2575	rpmJ	LSU ribosomal protein L36p @ LSU ribosomal protein L36p, zinc-dependent		
1	CDS	328442	328798	+	gtggcccgtatagcaggcattaa	MARIAGINIPDHKHAVIALTAIY	fig|127906.87.peg.312	VCC0312	VC2574	rpsM	SSU ribosomal protein S13p (S18e)		Limited query cover for certain strains
1	CDS	328818	329207	+	atggctaaacaaccaactcgcg	MAKQPTRARKRVRKQVADGV	fig|127906.87.peg.313	VCC0313	VC2573	rpsK	SSU ribosomal protein S11p (S14e)		
1	CDS	329235	329855	+	atggcaagatatttgggtcctaa	MARYLGPKLKLSRREGTDLFL	fig|127906.87.peg.314	VCC0314	VC2572	rpsD	SSU ribosomal protein S4p (S9e) @ SSU ribosomal protein S4p (S9e), zinc-independent		
1	CDS	329880	330872	+	atgcagggttctgtaacagaattt	MQGSVTEFLKPRLVDIEQISTT	fig|127906.87.peg.315	VCC0315	VC2571	rpoA	DNA-directed RNA polymerase alpha subunit (EC 2.7.7.6)		
1	CDS	330899	331285	+	atgcgccatcgtaagagtggtcg	MRHRKSGRQLNRNSSHRKAN	fig|127906.87.peg.316	VCC0316	VC2570	rplQ	LSU ribosomal protein L17p		
1	CDS	331905	331429	-	atgcactatcgttttactgccttac	MHYRFTALLISTLASTSALAFP	fig|127906.87.peg.317	VCC0317	VC2569		hypothetical protein	DUF2780-containing protein	
1	CDS	332667	332047	-	atgtctgaagtaaaatttgaaacc	MSEVKFETVEQKASYGIGLQM	fig|127906.87.peg.318	VCC0318	VC2568	fklB	FKBP-type peptidyl-prolyl cis-trans isomerase FklB (EC 5.2.1.8)		
1	CDS	334218	333409	-	ttgaacatgaaccgccgccacaagaagccgcagcaaactgattatctggcc		From N16961	Not annotated as CDS	VC2567				
1	CDS	334218	333409	-	atgaaccctgtagcgttagctgtt	MNPVALAVWLLVLGNLAASLS	fig|127906.87.peg.319	VCC0319	VC2566		hypothetical protein	EamA family transporter	
1	CDS	334670	334221	-	atgatcaactggcaatgtatccc	MINWQCIPFCQLTTQQLYELLI	fig|127906.87.peg.320	VCC0320	VC2565	ElaA	N-acetyltransferase ElaA		
1	CDS	334896	336278	+	ttgaacgccaactcttttgctgctt	MNANSFAALNLKPELLANLET	fig|127906.87.peg.321	VCC0321	VC2564	dbpA	ATP-dependent 23S rRNA helicase DbpA		
1	CDS	336850	336281	-	atgcaagcacagtttcacgctta	MQAQFHAYLKQHGFSDAECA	fig|127906.87.peg.322	VCC0322	VC2563		cAMP-binding proteins - catabolite gene activator and regulatory subunit of cAMP-dependent protein kinases		
1	CDS	338931	336970	-	atgaccatgatgatcaaacctgt	MTMMIKPVSLAVLGGLLAMAG	fig|127906.87.peg.323	VCC0323	VC2562	cpdB	2',3'-cyclic-nucleotide 2'-phosphodiesterase (EC 3.1.4.16) / 3'-nucleotidase (EC 3.1.3.6)		
1	CDS	339249	340115	+	atggcgcaggatgcggtaactc	MAQDAVTPLRKTPRLAVVEGS	fig|127906.87.peg.324	VCC0324	VC2561	cysG	Uroporphyrinogen-III methyltransferase (EC 2.1.1.107)		
1	CDS	340218	341126	+	atggatcaacagcgtttaaccca	MDQQRLTHLKQLEAESIHIIRE	fig|127906.87.peg.325	VCC0325	VC2560	cysD	Sulfate adenylyltransferase subunit 2 (EC 2.7.7.4)		
1	CDS	341144	342574	+	atgaacaatgcagtcaaagagc	MNNAVKEQLAELGIEGYLNQH	fig|127906.87.peg.326	VCC0326	VC2559	cysN	Sulfate adenylyltransferase subunit 1 (EC 2.7.7.4)		
1	CDS	342585	343232	+	atgagtaagcaagcagatagcc	MSKQADSLGFEQDAKPENVV	fig|127906.87.peg.327	VCC0327	VC2558	cysC	Adenylylsulfate kinase (EC 2.7.1.25)		
1	CDS	344204	343305	-	atggcggacaatatgacgcctg	MADNMTPVVIEHDSQQSQEK	fig|127906.87.peg.328	VCC0328	VC2557		hypothetical protein	TIGR03899 family protein [Vibrio cholerae]	1 ORF in C6706 while N16961 which have 2 ORF. Annotated as 1 pseudogene named VC2557 in KEGG
									VC2556		hypothetical protein		1 ORF in C6706 while N16961 which have 2 ORF. Annotated as 1 pseudogene named VC2557 in KEGG
1	CDS	345036	344515	-	atgaaacgttttatgttgatagcg	MKRFMLIASLIMAPLFSTPLLA	fig|127906.87.peg.329	VCC0329	VC2555		FIG003461: hypothetical protein		
1	CDS	346348	345089	-	atgaaagttatcctgaatttagcg	MKVILNLAWKSLLNRKATALLT	fig|127906.87.peg.330	VCC0330	VC2554	macB_4	ABC-type antimicrobial peptide transport system, permease component	DUF3299 domain-containing protein [Vibrio cholerae]	
1	CDS	347061	346345	-	atgcctgctcaccgttctgatccta	MPAHRSDPTSSVIELNDVVFC	fig|127906.87.peg.331	VCC0331	VC2553	ytrE_1	ABC-type antimicrobial peptide transport system, ATPase component		
1	CDS	347871	347143	-	atgcattacccaacaatgattgc	MHYPTMIALMVGATLSGNVLA	fig|127906.87.peg.332	VCC0332	VC2552		Predicted zinc-binding protein		
1	CDS	348210	347920	-	atgtcgttacctcagctacgtcca	MSLPQLRPTRLSIVAMLSVVL	fig|127906.87.peg.333	VCC0333	VC2551		hypothetical protein	DUF2607 family protein [Vibrio cholerae]	
1	CDS	348994	348443	-	atgaaaaataaaacacttatcg	MKNKTLIALLLACSPTLAFAHN	fig|127906.87.peg.334	VCC0334	VC2550		Protein ytfJ precursor		
1	CDS	349714	349076	-	atgctcgacaaacaaaccatgc	MLDKQTMVTAHNALPGRTTAI	fig|127906.87.peg.335	VCC0335	VC2549	msrA	Peptide-methionine (S)-S-oxide reductase MsrA (EC 1.8.4.11)		
1	CDS	349893	351608	+	atgagacgaaaatcacttcccg	MRRKSLPALISLWLFSAVSYAI	fig|127906.87.peg.336	VCC0336	VC2548	tamA	Outer membrane component of TAM transport system		
1	CDS	351605	355369	+	atgatcaaacgggcgttaaagt	MIKRALKWTKWLSMSLLVLVIL	fig|127906.87.peg.337	VCC0337	VC2547	tamB	Inner membrane component of TAM transport system		
1	CDS	355380	355727	+	gtgcagcacctcgttttgtgtatg	MQHLVFVYGTLRHGESNHTY	fig|127906.87.peg.338	VCC0338	VC2546	ytfP	UPF0131 protein YtfP		
1	CDS	356317	355787	-	atgagcttaaacaatgtgccagc	MSLNNVPAGKSLPDDIYVVEII	fig|127906.87.peg.339	VCC0339	VC2545	ppa	Inorganic pyrophosphatase (EC 3.6.1.1)		
1	CDS	357497	356487	-	atgcagaaaatgcgcaccctcc	MQKMRTLGEFIVEKQHDFPHA	fig|127906.87.peg.340	VCC0340	VC2544	fbp	Fructose-1,6-bisphosphatase, type I (EC 3.1.3.11)		
1	CDS	357803	359158	+	atgcacattcatatcttgggaattt	MHIHILGICGTFMGGVAMLAR	fig|127906.87.peg.341	VCC0341	VC2542	mpl	UDP-N-acetylmuramate:L-alanyl-gamma-D-glutamyl-meso-diaminopimelate ligase (EC 6.3.2.-)		
1	CDS	359171	359800	+	atgaacaacaatcgaaccatta	MNNNRTITLAWTGASGAPYGI	fig|127906.87.peg.342	VCC0342	VC2541	ubiX	Flavin prenyltransferase UbiX		
								Not annotated as CDS	VC2540				Hypothetical protein only annotated as CDS in N16961
1	CDS	360062	361054	+	ttgaaatttcaactgactgcggttg	MKFQLTAVALATLCVASAASA	fig|127906.87.peg.343	VCC0343	VC2539	thiB/tbpA	Thiamin ABC transporter, substrate-binding component		
1	CDS	361064	362656	+	ttgaatcgaactcctcaattaggc	MNRTPQLGFWVAAGVATFVL	fig|127906.87.peg.344	VCC0344	VC2538	thiP	Thiamin ABC transporter, transmembrane component		
1	CDS	362668	363384	+	atgttagctctagataaagtgcgt	MLALDKVRYEYEHEWFEFDLN	fig|127906.87.peg.345	VCC0345	VC2537	thiQ	Thiamin ABC transporter, ATPase component		
1	CDS	364068	363487	-	atgatgaagaatatggcacgtaa	MMKNMARKNQKAPWEEEEE	fig|127906.87.peg.346	VCC0346	VC2536	yjgA	UPF0307 protein YjgA		
1	CDS	364167	365510	+	atggatgtaaaagagcgagttg	MDVKERVALQRQELEAAVAK	fig|127906.87.peg.347	VCC0347	VC2535	pmbA	TldE protein, part of TldE/TldD proteolytic complex		
1	CDS	366943	365582	-	atggccgagcagatcgaatttga	MAEQIEFDQAHLTLQEITEALD	fig|127906.87.peg.348	VCC0348	VC2534	mgtE-2	Mg/Co/Ni transporter MgtE, CBS domain-containing		
1	CDS	367382	367104	-	atgccgcaactaagccaaacc	MPQLSQTVLIQNRLGLHARAA	fig|127906.87.peg.349	VCC0349	VC2533	ptsO	Phosphocarrier protein, nitrogen regulation associated		
1	CDS	368251	367391	-	atgcgtttgatcgtggtcagcgga	MRLIVVSGQSGAGKSVALRVL	fig|127906.87.peg.350	VCC0350	VC2532		RNase adapter protein RapZ		
1	CDS	368710	368264	-	atgcaattgagcgaagtactttca	MQLSEVLSLDCTKSAVQCSSK	fig|127906.87.peg.351	VCC0351	VC2531	ptsN	PTS IIA-like nitrogen-regulatory protein PtsN		Limited query cover for certain strains
1	CDS	369000	368713	-	atgcaaatcaacattcaaggcc	MQINIQGHHIDLTDSMQDYVH	fig|127906.87.peg.352	VCC0352	VC2530	hpf	Ribosome hibernation promoting factor Hpf		
1	CDS	370488	369025	-	atgaaaccgtcattacagctcaa	MKPSLQLKLGQQLAMTPQLQ	fig|127906.87.peg.353	VCC0353	VC2529	rpoN	RNA polymerase sigma-54 factor RpoN		
1	CDS	371269	370544	-	atggccatcttaaaagcacagc	MAILKAQHLAKSYKKRKVVSD	fig|127906.87.peg.354	VCC0354	VC2528	lptB	Lipopolysaccharide ABC transporter, ATP-binding protein LptB		
1	CDS	371766	371272	-	atgaaacgctcacaccttagtctt	MKRSHLSLIACLLASTQAYALS	fig|127906.87.peg.355	VCC0355	VC2527	lptA	Lipopolysaccharide export system protein LptA		
1	CDS	372310	371747	-	atgagtttgtcgcgcattgtttacg	MSLSRIVYVLLLFIASWSLYYL	fig|127906.87.peg.356	VCC0356	VC2525	lptC	Lipopolysaccharide export system protein LptC		
1	CDS	372864	372307	-	atgagttcaaccgtctcaaccttg	MSSTVSTLYGEVEPSLLEIAK	fig|127906.87.peg.357	VCC0357	VC2524	kdsC	3-deoxy-D-manno-octulosonate 8-phosphate phosphatase (EC 3.1.3.45)		
1	CDS	373838	372864	-	atgccccattcatttgattaccaa	MPHSFDYQTVAKQVLATEIHA	fig|127906.87.peg.358	VCC0358	VC2523	ldsD	D-arabinose-5-phosphate isomerase (EC 5.3.1.13)		
1	CDS	374856	373894	-	atgcttattgaagcggtggtcttt	MLIEAVVFLFIGLAILVWGADK	fig|127906.87.peg.359	VCC0359	VC2522	yrbG	Inner membrane protein YrbG, predicted calcium/sodium:proton antiporter		
1	repeat	374907	375041	+	catgactataccctctcctacttgaagctgcagcggtgttggctacgttcgttcac		fig|127906.87.repeat.9				repeat region		
1	CDS	375449	376252	+	atgtctcaatctgacttagtcacc	MSQSDLVTIKNLRFSRSQRVI	fig|127906.87.peg.360	VCC0360	VC2520	mlaF	Phospholipid ABC transporter ATP-binding protein MlaF		
1	CDS	376242	377033	+	atgctaaatgatgtgatgcgatg	MLNDVMRWVSSVGQRTLAVS	fig|127906.87.peg.361	VCC0361	VC2519	mlaE	Phospholipid ABC transporter permease protein MlaE		
1	CDS	377036	377527	+	atgcaacaaacacgaaaaattc	MQQTRKIEFWVGSFVIAGICAI	fig|127906.87.peg.362	VCC0362	VC2518	mlaD	Phospholipid ABC transporter substrate-binding protein MlaD		
1	CDS	377531	378169	+	atgtggaaaaaagcgctactcc	MWKKALLASTLLMPWLATAA	fig|127906.87.peg.363	VCC0363	VC2517	mlaC	Phospholipid ABC transporter shuttle protein MlaC		
1	CDS	378166	378495	+	atgagtcatccacaatggtctgc	MSHPQWSAENEHQFSLIGELL	fig|127906.87.peg.364	VCC0364	VC2516		Phospholipid ABC transporter-binding protein MlaB		
1	CDS	378488	378742	+	gtggatagcgcacaagtccaac	MDSAQVQQILQQALNLEEIHV	fig|127906.87.peg.365	VCC0365	VC2515		Acid stress protein IbaG		
1	CDS	378750	380009	+	atggaaaagtttcgagttattggc	MEKFRVIGSTQPLQGEVTISG	fig|127906.87.peg.366	VCC0366	VC2514	murA	UDP-N-acetylglucosamine 1-carboxyvinyltransferase (EC 2.5.1.7)		
1	CDS	380088	380816	+	atgattgcgttattgcgaatcttgg	MIALLRILAVAVFAVYMFVFGD	fig|127906.87.peg.367	VCC0367	VC2513	plsC	Acyl-CoA:1-acyl-sn-glycerol-3-phosphate acyltransferase (EC 2.3.1.51) @ Acyl-ACP:1-acyl-sn-glycerol-3-phosphate acyltransferase (EC 2.3.1.n4)		
1	CDS	381286	380897	-	atgacaaaagttctgcatactga	MTKVLHTDAAPAAIGPYIQGVI	fig|127906.87.peg.368	VCC0368	VC2512	ridA	2-iminobutanoate/2-iminopropanoate deaminase RidA/TdcF (EC 3.5.99.10)		
1	CDS	381858	381391	-	atgagcaaagagacgaaatta	MSKETKLQVEAIKNGTVIDHIP	fig|127906.87.peg.369	VCC0369	VC2511	pyrI	Aspartate carbamoyltransferase regulatory chain (PyrI)		
1	CDS	382799	381870	-	atggcgaattcgctttaccaaaa	MANSLYQKHIISIAELSRAELEL	fig|127906.87.peg.370	VCC0370	VC2510	pyrB	Aspartate carbamoyltransferase (EC 2.1.3.2)		
1	CDS	384053	383049	-	atggctttcaatttacgtaaccgc	MAFNLRNRNFLKLLDFTPKEIH	fig|127906.87.peg.371	VCC0371	VC2508	argF	Ornithine carbamoyltransferase (EC 2.1.3.3)		
1	CDS	385579	384203	-	atgggcgatacccgatcggaaa	MGDTDRKLFVLDTNILLHEPLA	fig|127906.87.peg.372	VCC0372	VC2507	phoH	Predicted ATPase related to phosphate starvation-inducible protein PhoH		
1	CDS	385653	385766	+	gtgagcgttacgatcattgtcgac	MSVTIIVDLLQTRVDTLCLNLQ	fig|127906.87.peg.373	VCC0373	No refSeq annotation		FIG01202169: hypothetical protein		
1	CDS	386001	388910	+	atgtcatttgctttagggcaacgc	MSFALGQRWISDTESDLGLGT	fig|127906.87.peg.374	VCC0374	VC2506	rapA/hepA	RNA polymerase associated protein RapA		
1	CDS	389007	389744	+	atggcaatgaccgagtacaatc	MAMTEYNPPKDPWIDVIYEDE	fig|127906.87.peg.375	VCC0375	VC2505	rluA_1	LSU rRNA pseudouridine(746) synthase (EC 5.4.99.29) @ tRNA pseudouridine(32) synthase (EC 5.4.99.28)		
1	CDS	389784	390761	+	atgtcactacccactacttcttgc	MSLPTTSLPTVVFLDRATIPRH	fig|127906.87.peg.376	VCC0376	VC2504		Glycerate dehydrogenase (EC 1.1.1.29)		
1	CDS	393700	390839	-	atggaaaagacatacaaccca	MEKTYNPTSIEQDLYKTWEEQ	fig|127906.87.peg.377	VCC0377	VC2503	valS	Valyl-tRNA synthetase (EC 6.1.1.9)		
1	CDS	394243	393794	-	atgcctaccgcaactttttacctga	MPTATFYLISRDSAQATPEGL	fig|127906.87.peg.378	VCC0378	VC2502	holC	DNA polymerase III chi subunit (EC 2.7.7.7)		
1	CDS	395838	394327	-	atggagttcagtgttaagagtgg	MEFSVKSGSPEKQRSACIVVG	fig|127906.87.peg.379	VCC0379	VC2501	pepA	Cytosol aminopeptidase PepA (EC 3.4.11.1)		
1	CDS	396032	397132	+	gtgattattgttagatatttgatccg	MIIVRYLIRETIKSQFAIFFVLFL	fig|127906.87.peg.380	VCC0380	VC2500	lptF	Lipopolysaccharide export system permease protein LptF		
1	CDS	397136	398206	+	gtgtttaaaattcttgattggtatat	MFKILDWYIGRTIVATTALVLVT	fig|127906.87.peg.381	VCC0381	VC2499	lptG	Lipopolysaccharide export system permease protein LptG		
1	CDS	398788	398312	-	atgatgaactacgcgacacttcg	MMNYATLPPAGFFRRLGAWIY	fig|127906.87.peg.382	VCC0382	VC2498		FIG023103: Predicted transmembrane protein	RDD family protein	
1	rna	398976	399060	+	gccgatgtggtgaaattggtagacacgacggattcaaaatccgtttccttcgg		fig|127906.87.rna.18	VCCt010	VCt086		tRNA-Leu-CAA		
1	CDS	399225	400478	+	atggcaagcattaaaatcacgg	MASIKITVDRLQPGLYIRLPVK	fig|127906.87.peg.383	VCC0383	VC2497	rpfG_5	HD-GYP domain		
1	repeat	400635	406162	+	ctccctataatgcgccctcactgacacggcagacgccacaaggtctcaagg		fig|127906.87.repeat.10				repeat region		
1	CDS	400826	400945	+	gtgtgggcactcgttgatgataat	MWALVDDNQKRFINELSDHLN	fig|127906.87.peg.384	VCC0384	VC2495		hypothetical protein		
1	rna	400970	402517	+	ttttaattgaagagtttgatcatggctcagattgaacgctggcggcaggcctaa		fig|127906.87.rna.19	VCr009	VCr018		SSU rRNA ## 16S rRNA, small subunit ribosomal RNA		
1	rna	402578	402654	+	gggtctgtagctcaggtggttagagcgtacgcctgataagcgtaaggtcggtg		fig|127906.87.rna.20	VCCt011	VCt085		tRNA-Ile-GAT		
1	rna	402663	402738	+	ggggttatagctcagctgggagagcgcctgccttgcacgcaggaggtctgc		fig|127906.87.rna.21	VCCt012	VCt084		tRNA-Ala-TGC		
1	rna	403026	405914	+	ggttaagtgactaagcgtacacggtggatgcctgggcagtcagaggcgatg		fig|127906.87.rna.22	VCr010	VCr017		LSU rRNA ## 23S rRNA, large subunit ribosomal RNA		
1	rna	406039	406160	+	ttgcttggcgaccatagcgttttggacccactgattccatcccgaactcagaa		fig|127906.87.rna.23	VCr011	VCr016		5S rRNA ## 5S ribosomal RNA		
1	rna	406206	406281	+	gctgatatggctcagtcggtagagcgcatccttggtaaggatgaggtccccag		fig|127906.87.rna.24	VCCt013	VCt083		tRNA-Thr-GGT		
1	rna	406321	406442	+	ttgtttggcgaccatagcactttggacccactgattccatcccgaactcagaa		fig|127906.87.rna.25	VCr012	VCr025		5S rRNA ## 5S ribosomal RNA		
1	CDS	407289	406498	-	atgaaaacactcttcgctttcttta	MKTLFAFFILIFSNIVYAAPKAE	fig|127906.87.peg.385	VCC0385	VC2494		Uncharacterized protein DUF547		
1	CDS	407992	407390	-	atgtcaggatttcagcaacacac	MSGFQQHTGLVVPLDAANVD	fig|127906.87.peg.386	VCC0386	VC2493	leuD	3-isopropylmalate dehydratase small subunit (EC 4.2.1.33)		
1	CDS	409411	408008	-	atgtcaaaagccaaaactcttta	MSKAKTLYEKIYDAHVVVAAP	fig|127906.87.peg.387	VCC0387	VC2492	leuC	3-isopropylmalate dehydratase large subunit (EC 4.2.1.33)		
1	CDS	410560	409469	-	atgacagacagagattacaaaa	MTDRDYKIAVLPGDGIGPEVM	fig|127906.87.peg.388	VCC0388	VC2491	leuB	3-isopropylmalate dehydrogenase (EC 1.1.1.85)		
1	CDS	412222	410672	-	atgaacaatcaggtgattatattc	MNNQVIIFDTTLRDGEQALSAS	fig|127906.87.peg.389	VCC0389	VC2490	leuA	2-isopropylmalate synthase (EC 2.3.3.13)		
1	CDS	412605	413240	+	atgacattacgtaaagtcggccg	MTLRKVGRPSQQTQARDQLIN	fig|127906.87.peg.390	VCC0390	VC2489		Transcriptional regulator, AcrR family		
1	CDS	413370	413903	+	atgagagctatggaaccgatcg	MRAMEPIAKLDLAFSLLCLQHI	fig|127906.87.peg.391	VCC0391	VC2488		putative membrane-associated phospholipid phosphatase, PAP2 superfamily		
1	CDS	413900	414988	+	ttgaaaatactgtatggcgttcaa	MKILYGVQGTGNGHIARSRAM	fig|127906.87.peg.392	VCC0392	VC2487		Glycosyltransferase		
1	CDS	415294	416253	+	atgttagataaaaaagacgcaa	MLDKKDAMSAIASYRMESTLR	fig|127906.87.peg.393	VCC0393	VC2485	leuO	Probable transcriptional activator for leuABCD operon		
1	CDS	416430	418235	+	atgacgaacttagatttccacatc	MTNLDFHIVKRIRQRIAQGGDI	fig|127906.87.peg.394	VCC0394	VC2484		Long-chain-fatty-acid--CoA ligase (EC 6.2.1.3)		
1	CDS	418720	420441	+	atggaaatgctatctggcgcaga	MEMLSGAEMIVQSLINEGVEQ	fig|127906.87.peg.395	VCC0395	VC2483	ilvI	Acetolactate synthase large subunit (EC 2.2.1.6)		
1	CDS	420443	420937	+	atgagacacatttatttcactgcta	MRHIISLLLENQPGALSRVVGL	fig|127906.87.peg.396	VCC0396	VC2482	ilvH	Acetolactate synthase small subunit (EC 2.2.1.6)		
1	repeat	420938	421068	+	tttatcccttcctacttgaagctgcagcggtgttggctacgttcgttcaccccaa		fig|127906.87.repeat.11				repeat region		
1	CDS	422398	421169	-	atggccaaagtttcactggaaaa	MAKVSLEKDRIKILLLEGLHPS	fig|127906.87.peg.397	VCC0397	VC2481	serA	D-3-phosphoglycerate dehydrogenase (EC 1.1.1.95)		
1	CDS	423308	422652	-	atgactcaagatgaaatgaaaa	MTQDEMKKAAGWAALKYVEK	fig|127906.87.peg.398	VCC0398	VC2480	rpiA	Ribose-5-phosphate isomerase A (EC 5.3.1.6)		
1	CDS	424002	423418	-	atgacagattctcgtacacacctt	MTDSRTHLRLQIRQRRQSLSA	fig|127906.87.peg.399	VCC0399	VC2479		5-formyltetrahydrofolate cyclo-ligase (EC 6.3.3.2)		
1	CDS	424510	424202	-	atgagtaatcaagcggttgacat	MSNQAVDIEILGKVTRVNCPA	fig|127906.87.peg.400	VCC0400	VC2478	zapA	Z-ring-associated protein ZapA		
1	CDS	424752	425327	+	atgagcaaaaatagacttcctgc	MSKNRLPAYPALANELRSASL	fig|127906.87.peg.401	VCC0401	VC2476		FIG037488: Putative conserved exported protein precursor	YecA family protein	Limited query cover for certain strains
1	CDS	425348	426556	+	atgacggacagccagcagccg	MTDSQQPNISKPISFDVIAGG	fig|127906.87.peg.402	VCC0402	VC2475	ubiH	2-polyprenyl-6-methoxyphenol hydroxylase		
1	CDS	426571	427797	+	atgcaaagcgttgatgttgtaatt	MQSVDVVIIGGGMVGLALAAA	fig|127906.87.peg.403	VCC0403	VC2474	ubiF/visC	2-polyprenylphenol hydroxylase		
1	CDS	428104	427898	-	atgagactgtttaagcgctataca	MRLFKRYTPGMIAKHVSRLFK	fig|127906.87.peg.404	VCC0404	VC2473		Uncharacterized protein VC2473	DUF1107 containing protein	
1	CDS	429709	428738	-	atggactggcaaaatcgtttttca	MDWQNRFSVLNLSSHDPLPE	fig|127906.87.peg.405	VCC0405	VC2472	ygfZ	tRNA-modifying protein YgfZ		
1	CDS	429862	430122	+	atgtacaccgctgaacaaaaag	MYTAEQKARIKWACRRGMLE	fig|127906.87.peg.406	VCC0406	VC2471	cptB	Succinate dehydrogenase flavin-adding protein, antitoxin of CptAB toxin-antitoxin		
1	CDS	430154	430516	+	atgatcgcattgctgattatgctgt	MIALLIMLLMLWALFVSSIPLVL	fig|127906.87.peg.407	VCC0407	VC2470		hypothetical protein		
1	CDS	432193	430541	-	atgtcgagttggatgactcgctac	MSSWMTRYSLGIGNFMNADR	fig|127906.87.peg.408	VCC0408	VC2469	nadB	L-aspartate oxidase (EC 1.4.3.16)		
1	CDS	432653	433225	+	atgaacgagcaactgaccgatc	MNEQLTDQVLIERVQNGDKQ	fig|127906.87.peg.409	VCC0409	VC2467	rpoE	RNA polymerase sigma factor RpoE		
1	CDS	433239	433877	+	atgactatggtgaatagaatggc	MTMVNRMADKEKLSALMDGE	fig|127906.87.peg.410	VCC0410	VC2466	rseA	Sigma factor RpoE negative regulatory protein RseA		
1	CDS	433874	434839	+	atgaagaaactttttgatcagtgc	MKKLLISALTLFCVNSTTAFAE	fig|127906.87.peg.411	VCC0411	VC2465	rseB	Sigma factor RpoE negative regulatory protein RseB precursor		
1	CDS	434836	435306	+	atgatgacggcattggctacggt	MMTALATVTKVVPAQQGFQV	fig|127906.87.peg.412	VCC0412	VC2464	rseC	Sigma factor RpoE regulatory protein RseC		
1	CDS	435419	437212	+	atgaagcacattcgtaactttcg	MKHIRNFSIIAHIDHGKSTLSDR	fig|127906.87.peg.413	VCC0413	VC2463	lepA	Translation elongation factor LepA		
1	CDS	437304	438200	+	atggcgaacacattctcactgat	MANTFSLILVIVTLVTGIVWTLE	fig|127906.87.peg.414	VCC0414	VC2462	lepB	Signal peptidase I (EC 3.4.21.89)		
1	CDS	438244	438909	+	atgaataaattaacaagtaagc	MNKLTSKLGYTFKETELLNLAL	fig|127906.87.peg.415	VCC0415	VC2461	rnc	Ribonuclease III (EC 3.1.26.3)		
1	CDS	438902	439879	+	atggctgatcaagaatttgatatc	MADQEFDIDAYFASQSEGKSV	fig|127906.87.peg.416	VCC0416	VC2460	era	GTP-binding protein Era		
1	CDS	439949	440674	+	atgtcagacggactgcaacgct	MSDGLQRCFVLHRRPYSESS	fig|127906.87.peg.417	VCC0417	VC2459	recO	DNA recombination and repair protein RecO		
1	CDS	440671	441402	+	atgagctcaatttatcttggtgtga	MSSIYLGVNIDHVATLRNARG	fig|127906.87.peg.418	VCC0418	VC2458	pdxJ	Pyridoxine 5'-phosphate synthase (EC 2.6.99.2)		
1	CDS	441406	441786	+	atgatcgttggactcggaacgga	MIVGLGTDIAEIERVEKALARS	fig|127906.87.peg.419	VCC0419	VC2457	acpS	Holo-[acyl-carrier-protein] synthase (EC 2.7.8.7)		
1	CDS	442025	443326	+	atgcttagggataacccaatgat	MLRDNPMIRSLFQTRLMSGV	fig|127906.87.peg.420	VCC0420	VC2456		hypothetical protein		
1	CDS	443336	443746	+	atgcgatattggttactcctcattc	MRYWLLLILLLSPWVSASAEL	fig|127906.87.peg.421	VCC0421	VC2455		hypothetical protein	ABC-type sulfate transport system permease component	Limited query cover for certain strains
1	CDS	443739	445031	+	atgactgatcaaacgcgaactt	MTDQTRTSPLVNRLLKMISLC	fig|127906.87.peg.422	VCC0422	VC2454		GGDEF domain protein		
1	CDS	447769	445019	-	atgacgctcactttggcacctac	MTLTLAPTLIIGLLLSALFSFNR	fig|127906.87.peg.423	VCC0423	VC2453	barA_2	Signal transduction histidine-protein kinase BarA (EC 2.7.13.3)		
1	CDS	447974	449296	+	atggcacgtttcttcaaccgaaa	MARFFQPKKHSTLDTKHQPVT	fig|127906.87.peg.424	VCC0424	VC2452	rumA	23S rRNA (uracil(1939)-C(5))-methyltransferase (EC 2.1.1.190)		
1	CDS	449360	451576	+	atggttgcggtacgaagcgcac	MVAVRSAHLNPDQQFELETW	fig|127906.87.peg.425	VCC0425	VC2451	relA	Inactive (p)ppGpp 3'-pyrophosphohydrolase domain / GTP pyrophosphokinase (EC 2.7.6.5), (p)ppGpp synthetase I		
1	CDS	451676	452491	+	atgtcacacccgattgaacaact	MSHPIEQLEQIMRRLRDPEQG	fig|127906.87.peg.426	VCC0426	VC2450	mazG	Nucleoside triphosphate pyrophosphohydrolase MazG (EC 3.6.1.8)		
1	CDS	452680	452540	-	gtgaaatctatcacactcaatttttt	MKSITLNFLFFASNQTKLKQTL	fig|127906.87.peg.427	VCC0427	VC2449		hypothetical protein		
1	CDS	452766	454403	+	atgacgacaaattatattttgtta	MTTNYIFVTGGVVSSLGKGIAA	fig|127906.87.peg.428	VCC0428	VC2448	pyrG	CTP synthase (EC 6.3.4.2)		
1	CDS	454482	455783	+	atgtctaagatcgttaaagttcta	MSKIVKVLGREIIDSRGNPTVE	fig|127906.87.peg.429	VCC0429	VC2447	eno	Enolase (EC 4.2.1.11)		
1	CDS	457096	455885	-	atgcaaatttacctcgttggcggt	MQIYLVGGAVRDQLLQLPVYD	fig|127906.87.peg.430	VCC0430	VC2446	cca	CCA tRNA nucleotidyltransferase (EC 2.7.7.72)		
1	CDS	457189	458778	+	atgtatctgaacttctttgggtttga	MYLNFFGFDELPFSIVPNARYI	fig|127906.87.peg.431	VCC0431	VC2445	gspA/exeA	General secretion pathway protein A		
1	CDS	458778	459548	+	atgtcaaaagtgatgaatgcctt	MSKVMNALKQSQQKYLQAQR	fig|127906.87.peg.432	VCC0432	VC2444		General secretion pathway protein B		
1	CDS	460248	459640	-	gtgaaaaaactgatctgcatggt	MKKLICMVLFSMLAAPTFAQD	fig|127906.87.peg.433	VCC0433	VC2443		Arylsulfatase (EC 3.1.6.1)		
1	CDS	461677	460415	-	atggaaatccttgcgaactacgg	MEILANYGTVLIIVAAVFGFLMA	fig|127906.87.peg.434	VCC0434	VC2442	pitA	Probable low-affinity inorganic phosphate transporter		
1	CDS	462500	461820	-	atgccagtaaatacaatcatggg	MPVNTIMGLFAKSPIKPLQRH	fig|127906.87.peg.435	VCC0435	VC2441		Phosphate transport regulator (distant homolog of PhoU)		
1	CDS	462706	464223	+	atggaaaccgagatagaactga	METEIELKFFVSPDFSTILRAKI	fig|127906.87.peg.436	VCC0436	VC2440		Inorganic triphosphatase (EC 3.6.1.25)		
1	CDS	464631	464467	-	atgaaagccgataatatcaaaa	MKADNIKNETKPISLLSLILLFIT	fig|127906.87.peg.437	VCC0437	Not annotated as CDS		FIG01199642: hypothetical protein		
1	CDS	464708	465964	+	atggcaacgatgtcgtttaagcc	MATMSFKPWERVITDVRLVPK	fig|127906.87.peg.438	VCC0438	VC2439	mcp4	Methyl-accepting chemotaxis sensor/transducer protein		
1	CDS	466088	468934	+	atgtcactgcctagcgcgctttg	MSLPSALLPTAELHYQSLISEH	fig|127906.87.peg.439	VCC0439	VC2438	glnE	Glutamine synthetase adenylyl-L-tyrosine phosphorylase (EC 2.7.7.89) / Glutamate-ammonia-ligase adenylyltransferase (EC 2.7.7.42)		
1	CDS	468998	470428	+	atgaaaccagtgctacctgacta	MKPVLPDYSKAGVLIVGDVML	fig|127906.87.peg.440	VCC0440	VC2437	hldE	D-glycero-beta-D-manno-heptose 1-phosphate adenylyltransferase (EC 2.7.7.70) / D-glycero-beta-D-manno-heptose-7-phosphate kinase (EC 2.7.1.167)		

									VC2437		D-glycero-beta-D-manno-heptose 1-phosphate adenylyltransferase (EC 2.7.7.70) / D-glycero-beta-D-manno-heptose-7-phosphate kinase (EC 2.7.1.167)		ORF split into 2 ORFs in N16961 and not annotated in PATRIC. Annotated as pseudogene named VC2437 in KEGG.
1	CDS	471850	470534	-	atgaaaaaactgcttccattattt	MKKLLPLFVSAALGTLSSAVW	fig|127906.87.peg.441	VCC0441	VC2436	tolC	Outer membrane channel TolC (OpmH)		
1	CDS	472082	472708	+	atgcaagacgcgaatcagcag	MQDANQQPVSFNKKDVEILNI	fig|127906.87.peg.442	VCC0442	VC2435	nudF_1	ADP-ribose pyrophosphatase (EC 3.6.1.13)		
1	CDS	472715	473161	+	atgagtcaattgacagttaaaaa	MSQLTVKKPYHVDLPELMRVY	fig|127906.87.peg.443	VCC0443	VC2434		Uncharacterized protein YqiB		
1	CDS	473186	474004	+	ttgaaagtgtcgtctcaatcagac	MKVSSQSEDSSIKLIQITDTHLI	fig|127906.87.peg.444	VCC0444	VC2433	cpdA	3',5'-cyclic-nucleotide phosphodiesterase (EC 3.1.4.17)		Only 92% query cover in selected strains
1	CDS	474006	474593	+	atgactgctcgtcctgctttgctgc	MTARPALLLYIHGFNSSPLSHI	fig|127906.87.peg.445	VCC0445	VC2432		Esterase YqiA		
1	CDS	474922	476802	+	atgactgaacaatataatgctgg	MTEQYNAGAIEVLNGLEPVRR	fig|127906.87.peg.446	VCC0446	VC2431	parE	DNA topoisomerase IV subunit B (EC 5.99.1.3)		
1	CDS	476806	479091	+	atgtctactgaaatcagctttgatg	MSTEISFDGVEQLPLRKFTED	fig|127906.87.peg.447	VCC0447	VC2430	ParC	DNA topoisomerase IV subunit A (EC 5.99.1.3)		
1	CDS	479337	479140	-	atgaccaaaaaactcaccatag	MTKKLTIVKCPRCGTDVEWGE	fig|127906.87.peg.448	VCC0448	VC2429	yacG	DNA gyrase inhibitor YacG		
1	CDS	479363	479482	+	atggcttatccccttaaaaccggc	MAYPLKTGNAKLCGDTLHQS	fig|127906.87.peg.449	VCC0449	No refSeq annotation		FIG01201569: hypothetical protein		
1	CDS	480219	479479	-	atgactacgcaccagtttgaaca	MTTHQFEHPLNEKTRIYLRVE	fig|127906.87.peg.450	VCC0450	VC2428		Cell division protein ZapD		
1	CDS	480859	480251	-	atgagttttgtcgtagcattgaccg	MSFVVALTGGIASGKTTVANL	fig|127906.87.peg.451	VCC0451	VC2427	coaE	Dephospho-CoA kinase (EC 2.7.1.24)		
1	CDS	481731	480856	-	atggaactgttttacttctaccctg	MELFYFYPWLFPVLATLFGLIV	fig|127906.87.peg.452	VCC0452	VC2426	outO/pilD	Leader peptidase (Prepilin peptidase) (EC 3.4.23.43) / N-methyltransferase (EC 2.1.1.-)		
1	CDS	483015	481789	-	atgaaagcgacccaaaccttac	MKATQTLPLKNYRWKGINSN	fig|127906.87.peg.453	VCC0453	VC2425	epsF_2/pilC	Type IV fimbrial assembly protein PilC		
1	CDS	484746	483058	-	atgctcaccaaccttgttgctatct	MLTNLVAILRQAELISATQEQA	fig|127906.87.peg.454	VCC0454	VC2424	epsE_2/pilB	Type IV fimbrial assembly, ATPase PilB		
1	CDS	485207	484746	-	atgaaagcgtataaaaacaaac	MKAYKNKQQKGFTLIELMIVV	fig|127906.87.peg.455	VCC0455	VC2423	pilA	Type IV pilin PilA		
1	CDS	486342	485452	-	atgaaagagactcacaacagtc	MKETHNSQDRLAYLKQQLPA	fig|127906.87.peg.456	VCC0456	VC2422	nadC	Quinolinate phosphoribosyltransferase [decarboxylating] (EC 2.4.2.19)		
1	CDS	486638	487183	+	atgattgatagtcaaggctggta	MIDSQGWYRLARKVPSPHCD	fig|127906.87.peg.457	VCC0457	VC2421	ampD	1,6-anhydro-N-acetylmuramyl-L-alanine amidase		
1	CDS	488057	487461	-	atgcaggatattggctgcattccg	MQDIGCIPIIFMVRICHSFADRY	fig|127906.87.peg.458	VCC0458	VC2420	fldA_2/fldB	Flavodoxin 2		
1	CDS	488117	489025	+	gtgagcgaagccttatctcccga	MSEALSPDQGLVEQFLDTMW	fig|127906.87.peg.459	VCC0459	VC2419	xerD	Site-specific tyrosine recombinase XerD		
1	CDS	489042	489794	+	atgagcgtactgcgccgactaa	MSVLRRLTWLAFPLLSMAFNV	fig|127906.87.peg.460	VCC0460	VC2418	dsbC	Thiol:disulfide interchange protein DsbC		
1	CDS	489838	491592	+	ttggcctgtcacagtatgatagaa	MACHSMIEIQRRPEPDLSLLPI	fig|127906.87.peg.461	VCC0461	VC2417	recJ	Single-stranded-DNA-specific exonuclease RecJ		Limited query cover for certain strains
1	CDS	493541	491637	-	atgaaaaaaacaatcttaacca	MKKTILTSAIALSFAFPALAAN	fig|127906.87.peg.462	VCC0462	VC2416	yfkN_2	5'-nucleotidase (EC 3.1.3.5); 2',3'-cyclic-nucleotide 2'-phosphodiesterase (EC 3.1.4.16); Putative UDP-sugar hydrolase (EC 3.6.1.45)		
1	CDS	494025	494795	+	atggcttatcaaaggatccgtca	MAYQRIRQPKLSDVIEQELERI	fig|127906.87.peg.463	VCC0463	VC2415	pdhR	Transcriptional repressor for pyruvate dehydrogenase complex		
1	CDS	494845	497505	+	atgtctgacatgaagcatgacgt	MSDMKHDVDALETQEWLAAL	fig|127906.87.peg.464	VCC0464	VC2414	aceE	Pyruvate dehydrogenase E1 component (EC 1.2.4.1)		
1	CDS	497524	499413	+	atggcaatcgaaatttatgtacct	MAIEIYVPDIGADEVEVTEILVK	fig|127906.87.peg.465	VCC0465	VC2413	aceF	Dihydrolipoamide acetyltransferase component of pyruvate dehydrogenase complex (EC 2.3.1.12)		
1	CDS	499681	501108	+	atgagcaaagaaattaaagccc	MSKEIKAQVVVLGAGPAGYSA	fig|127906.87.peg.466	VCC0466	VC2412	lpdA	Dihydrolipoamide dehydrogenase of pyruvate dehydrogenase complex (EC 1.8.1.4)		
1	CDS	501866	502816	+	atgactgcttctttccaacacatct	MTASFQHISVLLNESIEGLAIKI	fig|127906.87.peg.467	VCC0467	VC2409	mraW	16S rRNA (cytosine(1402)-N(4))-methyltransferase (EC 2.1.1.199)		
1	CDS	502826	503146	+	atgcctcgtcagtcgccgcgaa	MPRQSPPNLAKLIALDLLTVGI	fig|127906.87.peg.468	VCC0468	VC2408	ftsL	Cell division protein FtsL		
1	CDS	503143	504885	+	atgaagaagaaagccccagcc	MKKKAPAKASRVSPRVTTAEI	fig|127906.87.peg.469	VCC0469	VC2407	ftsI	Cell division protein FtsI [Peptidoglycan synthetase] (EC 2.4.1.129)		
1	CDS	504902	506389	+	atgaacacgcattcagcgatctc	MNTHSAISELIAPWLELTDPKL	fig|127906.87.peg.470	VCC0470	VC2406	murrE	UDP-N-acetylmuramoyl-dipeptide--2,6-diaminopimelate ligase (EC 6.3.2.13)		
1	CDS	506386	507747	+	atgattcgcgtaatgctttctcagc	MIRVMLSQLSSVLNARLIGEDI	fig|127906.87.peg.471	VCC0471	VC2405	murF	UDP-N-acetylmuramoyl-tripeptide--D-alanyl-D-alanine ligase (EC 6.3.2.10)		
1	CDS	507744	508826	+	atgattatttggcttgctgaactgc	MIIWLAELLQPYFSFFRLFEYL	fig|127906.87.peg.472	VCC0472	VC2404	mraY	Phospho-N-acetylmuramoyl-pentapeptide-transferase (EC 2.7.8.13)		
1	CDS	508850	510172	+	atggaccgttggcaaggcatac	MDRWQGIQHVVVVGLGITGLS	fig|127906.87.peg.473	VCC0473	VC2403	murD	UDP-N-acetylmuramoyl-L-alanine--D-glutamate ligase (EC 6.3.2.9)		
1	CDS	510189	511385	+	ttgtttttatccggatctttcacaaa	MFLSGSFTKLSHWLRTSSPEA	fig|127906.87.peg.474	VCC0474	VC2402	ftsW	Peptidoglycan glycosyltransferase FtsW (EC 2.4.1.129)		
1	CDS	511372	512433	+	atgaacaagaataaaaaattaa	MNKNKKLMVMAGGTGGHVFI	fig|127906.87.peg.475	VCC0475	VC2401	murG	UDP-N-acetylglucosamine--N-acetylmuramyl-(pentapeptide) pyrophosphoryl-undecaprenol N-acetylglucosamine transferase (EC 2.4.1.227)		
1	CDS	512447	513907	+	atgacgattaaacatacccaag	MTIKHTQDLAQIRAMVPEMRR	fig|127906.87.peg.476	VCC0476	VC2400	murC	UDP-N-acetylmuramate--L-alanine ligase (EC 6.3.2.8)		
1	CDS	514089	514871	+	ttgatcaacaaggtacttttagaa	MINKVLLEGQRITRSPQVKQH	fig|127906.87.peg.477	VCC0477	VC2399	ftsQ	Cell division protein FtsQ		
1	CDS	514864	516126	+	atgactaagactgtcgatgacaa	MTKTVDDNIIVGLDIGTATVSA	fig|127906.87.peg.478	VCC0478	VC2398	ftsA	Cell division protein FtsA		
1	CDS	516157	517353	+	atgtttgaaccgatgatggaaat	MFEPMMEMSDDAVIKVVGVG	fig|127906.87.peg.479	VCC0479	VC2397	ftsZ	Cell division protein FtsZ		
1	CDS	517451	518368	+	atgatcagacaacgtactctgaa	MIRQRTLKEIVKTTGVGLHSGI	fig|127906.87.peg.480	VCC0480	VC2396	lpxC	UDP-3-O-[3-hydroxymyristoyl] N-acetylglucosamine deacetylase (EC 3.5.1.108)		
1	CDS	518946	518473	-	atgcgtgatcaccgccctaccgc	MRDHRPTATDELIQASKLKQI	fig|127906.87.peg.481	VCC0481	VC2395		Zn-ribbon-containing, possibly RNA-binding protein and truncated derivatives		
1	CDS	519300	522011	+	atgataactaagctactgacaaa	MITKLLTKVIGSRNDRTLRRLR	fig|127906.87.peg.482	VCC0482	VC2394	secA	Protein translocase subunit SecA		
1	CDS	522161	522559	+	ttgaaacgcatccacattgttgca	MKRIHIVAGIIFNSDQSEIFITKR	fig|127906.87.peg.483	VCC0483	VC2392	mutT	Mutator MutT protein (7,8-dihydro-8-oxoguanine-triphosphatase) (EC 3.6.1.-)		
1	CDS	522674	523483	+	atggttagaattgcgattgcgggt	MVRIAIAGAAGRMGRNLVKAT	fig|127906.87.peg.484	VCC0484	VC2391	dapB	4-hydroxy-tetrahydrodipicolinate reductase (EC 1.17.1.8)		
1	CDS	523699	523571	-	atggaaataggtggcaaaaaa	MEIGGKKTTTHKAIVNKPSFYF	fig|127906.87.peg.485	VCC0485	Not annotated as CDS		FIG01200145: hypothetical protein	1-deoxy-D-xylulose 5-phosphate reductoisomerase	
1	CDS	523966	525105	+	ttgagtaaatcagcactgttagtc	MSKSALLVLEDGTVFRGVSIG	fig|127906.87.peg.486	VCC0486	VC2390	carA	Carbamoyl-phosphate synthase small chain (EC 6.3.5.5)		
1	CDS	525122	528352	+	atgccaaaacgtactgacattca	MPKRTDIQSILILGAGPIVIGQA	fig|127906.87.peg.487	VCC0487	VC2389	carB	Carbamoyl-phosphate synthase large chain (EC 6.3.5.5)		
1	CDS	528924	528442	-	atgagtaaaaagagcatcacgg	MSKKSITALLGIGLVSIGLLYQF	fig|127906.87.peg.488	VCC0488	VC2388		hypothetical protein		
1	CDS	529113	529430	+	atgctggttattccaagtattcacg	MLVIPSIHEQMCLFCLLQCGW	fig|127906.87.peg.489	VCC0489	No refSeq annotation		putative transposase		Limited query cover for certain strains
1	CDS	530152	529535	-	atgattccattatcacacaataat	MIPLSHNNTPTELENYVKLKG	fig|127906.87.peg.490	VCC0490	VC2387		hypothetical protein	TIGR02646 family protein	
1	CDS	531787	530165	-	atgaatgagccaactagaatcc	MNEPTRIPKYVKDKIRQAKQG	fig|127906.87.peg.491	VCC0491	VC2386		Putative ATP binding protein SugR		
1	CDS	532735	531788	-	ttgaatatactaaccacattaaga	MNILTTLREQLLTNNVIMPQEF	fig|127906.87.peg.492	VCC0492	VC2385		Retron-type RNA-directed DNA polymerase (EC 2.7.7.49)		
1	CDS	534796	533936	-	atgttactcgaaggcattgaaac	MLLEGIETLLVLSKEKTMSRTG	fig|127906.87.peg.493	VCC0493	VC2383		Transcriptional regulator VC2383, LysR family		
					atggaactttcactggaagtattgggctgctgtttttagtggcgggagttgcgg		From N16961	Not annotated as CDS	VC2384				Additional ORF in selected strains that partly overlap with VCC0493
1	CDS	535568	534948	-	ttggatacttctctgctttatctgatt	MDTSLLYLIDMFGTAVFAVSG	fig|127906.87.peg.494	VCC0494	VC2382		UPF0126 inner membrane protein YadS		
1	CDS	536481	535651	-	atgcttgttatccggcttatcgcct	MLVIRLIACTFLFITPSLLAKPF	fig|127906.87.peg.495	VCC0495	VC2381	btuF	Vitamin B12 ABC transporter, substrate-binding protein BtuF		
1	CDS	537435	536482	-	atggaaactgggttttcccactttt	METGFSHFYANGALLVMWGA	fig|127906.87.peg.496	VCC0496	VC2380		Adenosylcobinamide-phosphate synthase (EC 6.3.1.10)		
1	CDS	538143	537448	-	atgaaaatcggcatcatcggcg	MKIGIIGAMQQEVAILKDLIEDV	fig|127906.87.peg.497	VCC0497	VC2379	pfs/mtnN	5'-methylthioadenosine nucleosidase (EC 3.2.2.16) @ S-adenosylhomocysteine nucleosidase (EC 3.2.2.9)		
1	CDS	538665	538231	-	atgcacccatcgttttatgccatta	MHPSFYAIIAAFSLTACSQGVD	fig|127906.87.peg.498	VCC0498	VC2378		hypothetical protein	DUF1499 domain-containing protein [Vibrio cholerae]	
1	CDS	540264	538852	-	atgagccaaaacgtttaccaatt	MSQNVYQFIDVNRVDPAKKPL	fig|127906.87.peg.499	VCC0499	VC2377	gltD	Glutamate synthase [NADPH] small chain (EC 1.4.1.13)		
1	CDS	544746	540283	-	atggctctatacgatccaagtctt	MALYDPSLEKDNCGFGLIAHM	fig|127906.87.peg.500	VCC0500	VC2376	gltB	Glutamate synthase [NADPH] large chain (EC 1.4.1.13)		
1	CDS	546743	545274	-	atgggtaaagcaaccggattttt	MGKATGFLEFGRELPKKIDPA	fig|127906.87.peg.501	VCC0501	VC2374	gltD	Glutamate synthase [NADPH] small chain (EC 1.4.1.13)		
1	CDS	551288	546744	-	atggtagataaagcgcaacgttc	MVDKAQRSRGLYTPELEHDA	fig|127906.87.peg.502	VCC0502	VC2373	gltB	Glutamate synthase [NADPH] large chain (EC 1.4.1.13)		
1	CDS	551714	551833	+	atgcaaatagtgtttattttatgcct	MQIVFILCLFIDLTLFEIKREGLK	fig|127906.87.peg.503	VCC0503	VC2372		FIG01200833: hypothetical protein		
1	CDS	551867	552805	+	atgcatttacatcaactggtgaac	MHLHQLVNTIGQDLQRRYGEI	fig|127906.87.peg.504	VCC0504	VC2371		[4Fe-4S]-AdoMet protein YhcC		
1	CDS	552873	554612	+	atgcctgaatttctctctgaatttgt	MPEFLSEFVRFLFAAGLVLGG	fig|127906.87.peg.505	VCC0505	VC2370		Sensory box/GGDEF family protein		
1	CDS	554719	557076	+	atgaaaccgatgaaaaatctcg	MKPMKNLAQYYVDLLVKLGILI	fig|127906.87.peg.506	VCC0506	VC2369	arcB	Aerobic respiration control sensor protein ArcB (EC 2.7.13.3)		
1	CDS	557851	557135	-	atgcaaaccccgcagatccttat	MQTPQILIVEDEQVTRNTLKSI	fig|127906.87.peg.507	VCC0507	VC2368	arcA/fexA	Aerobic respiration control response regulator ArcA		
1	CDS	558166	558549	+	atgatcaagattgacgctagcct	MIKIDASLWLAYQSVKFAFEKF	fig|127906.87.peg.508	VCC0508	VC2367		hypothetical protein		
1	CDS	558579	559064	+	atgagagatataaccccagatc	MRDITPDLCDKYESQVTLLNLI	fig|127906.87.peg.509	VCC0509	VC2366		Ribonuclease E inhibitor RraA		
1	CDS	559543	559061	-	ttggctggtacatccggggctatc	MAGTSGAIVKTAFFCLITLVAP	fig|127906.87.peg.510	VCC0510	VC2365		hypothetical protein		
1	CDS	560091	562550	+	atgcgagtattaaagtttggagg	MRVLKFGGSSLADPERFLRAA	fig|127906.87.peg.511	VCC0511	VC2364	thrA	Aspartokinase (EC 2.7.2.4) / Homoserine dehydrogenase (EC 1.1.1.3)		
1	CDS	562571	563527	+	atgagtgttgtggtgtacgcacc	MSVVVYAPASIGNVSVGFDVL	fig|127906.87.peg.512	VCC0512	VC2363	thrB	Homoserine kinase (EC 2.7.1.39)		
1	CDS	563524	564804	+	atgaagctgtacaacataaaag	MKLYNIKENDEQVSFGQAVRC	fig|127906.87.peg.513	VCC0513	VC2362	thrC	Threonine synthase (EC 4.2.3.1)		
1	CDS	565261	564884	-	atgatccaaggtatccaaatcac	MIQGIQITKAANDDLLNSIWLLI	fig|127906.87.peg.514	VCC0514	VC2361	grcA	Autonomous glycyl radical cofactor		
1	CDS	565607	566467	+	atgtatgagaagttaatcggggc	MYEKLIGAHVSASGGVELAPV	fig|127906.87.peg.515	VCC0515	VC2360	nfo	Endonuclease IV (EC 3.1.21.2)		
1	CDS	566836	567516	+	atgtctgaatcactgacgtggca	MSESLTWHDVIGNEKQQAYFC	fig|127906.87.peg.516	VCC0516	VC2359	ung	Uracil-DNA glycosylase, family 1 (EC 3.2.2.27)		
1	CDS	567606	568157	+	atgatgatggaacgaatccgcc	MMMERIRREHSYMVRLLSILR	fig|127906.87.peg.517	VCC0517	VC2358		Repair of Iron Centers di-iron protein		
1	CDS	568480	568301	-	atggacgaactgcactttgatga	MDELHFDELLEMEISKIRTSRS	fig|127906.87.peg.518	VCC0518	VC2357		hypothetical protein	MULTISPECIES: DUF3545 family protein [Vibrio]	
1	CDS	569173	570603	+	gtgacagacttaattaatttaatg	MTDLINLMNDLLWGSILVYLLV	fig|127906.87.peg.519	VCC0519	VC2356	alsT_3	Sodium:alanine symporter family protein		
1	CDS	570767	571540	+	atgctgattgtcgtttcacctgcga	MLIVVSPAKTLDYESPVSTSNI	fig|127906.87.peg.520	VCC0520	VC2355		UPF0246 protein YaaA	peroxide stress protein YaaA	
								Not annotated as CDS	VC2354				ORF coding for Hypothetical protein that is present in N16961. Partly overlaps with yjjV
1	CDS	571682	572506	+	atgaacgagacgattccacctc	MNETIPPPLEWSLFDTHCHFD	fig|127906.87.peg.521	VCC0521	VC2353	yjjV	Putative deoxyribonuclease YjjV		
1	CDS	572693	573949	+	atgagcctgtttatgagcctcatc	MSLFMSLIGMAVLLGIAVLLSS	fig|127906.87.peg.522	VCC0522	VC2352	nupX_2	Na+ dependent nucleoside transporter NupC		
1	CDS	574443	574261	-	atgcaccaattgctatccttgtca	MHQLLSLSPHSTSLVNGTTICV	fig|127906.87.peg.523	VCC0523	VC2351		FIG01200040: hypothetical protein		
1	CDS	574551	575330	+	atgagcgaattaaaagtagcag	MSELKVAALRALKLMDLTTLNI	fig|127906.87.peg.524	VCC0524	VC2350	deoC	Deoxyribose-phosphate aldolase (EC 4.1.2.4)		
1	CDS	575418	576764	+	atgtctttatctcaagcaaagtatt	MSLSQAKYLPQEIIRRKRDGE	fig|127906.87.peg.525	VCC0525	VC2349	deoA	Thymidine phosphorylase (EC 2.4.2.4)		
1	CDS	576782	578002	+	atgaaaagagcatttattttagttt	MKRAFILVLDSFGIGATADAQA	fig|127906.87.peg.526	VCC0526	VC2348	deoB	Phosphopentomutase (EC 5.4.2.7)		
1	CDS	578085	578810	+	atggcaactccacatattaatgc	MATPHINAQMGDFADVVLMP	fig|127906.87.peg.527	VCC0527	VC2347	deoD	Purine nucleoside phosphorylase (EC 2.4.2.1)		
1	CDS	579513	578890	-	atggatggctcactttttcattccg	MDGSLFSFRVVVRVATVLAVA	fig|127906.87.peg.528	VCC0528	VC2346		Smp-like protein		
1	CDS	579616	580596	+	atggatgctttaacaactttaccg	MDALTTLPIKKHTALLNRFPET	fig|127906.87.peg.529	VCC0529	VC2345	serB	Phosphoserine phosphatase (EC 3.1.3.3)		
1	repeat	580664	580791	+	tatacccttcctacttgaagctgcacgagtgttggctacgttcgttcaccccaat		fig|127906.87.repeat.12				repeat region		
1	CDS	583140	580801	-	atgcaacagaccgaaattctctc	MQQTEILSLVERLIPVYRSGDL	fig|127906.87.peg.530	VCC0530	VC2344	PilZ domain-containing protein	hypothetical protein		
1	CDS	583274	584647	+	gtgaaagctaagcgcgcctatg	MKAKRAYVCNECGADFPRWQ	fig|127906.87.peg.531	VCC0531	VC2343	lon2/radA	DNA repair protein RadA		
1	CDS	584918	587005	+	atggcagatttatcattatacaga	MADLSLYRNIGIFAHVDAGKTT	fig|127906.87.peg.532	VCC0532	VC2342	fusA_2	Translation elongation factor G paralog VC2342		
1	CDS	588804	587119	-	atgatccagcctaatcagttcaa	MIQPNQFNHPPCALPPPNEMI	fig|127906.87.peg.533	VCC0533	VC2341		Long-chain-fatty-acid--CoA ligase (EC 6.2.1.3)		
1	CDS	590164	589010	-	atgcagatgcaacccaaagtcg	MQMQPKVGQFRYYPSVGSLN	fig|127906.87.peg.534	VCC0534	VC2340		Response regulator containing a CheY-like receiver domain and an HD-GYP domain		
1	CDS	591442	590300	-	atgctaacgatttttagaactttttt	MLTIFRTFFALGWVSFGGPAA	fig|127906.87.peg.535	VCC0535	VC2339	srpC	Chromate transport protein ChrA		
1	CDS	591825	594899	+	atgcgcaacttctccgatattcttc	MRNFSDILLSQDWQNPHIVKW	fig|127906.87.peg.536	VCC0536	VC2338	LacZ	beta-galactosidase (EC 3.2.1.23)		LacZ gene split into two according to Patric. Annotated as VC2338 pseudogene in KEGG
1	CDS	595918	594911	-	atggcaaccataaaagacgtag	MATIKDVAREAGVSVATVSRV	fig|127906.87.peg.537	VCC0537	VC2337	galR_2	Galactose operon repressor, GalR-LacI family of transcriptional regulators		
1	CDS	596077	596421	+	atggactcggcgtttttgacggta	MDSAFLTVGYDDFARLDLRIG	fig|127906.87.peg.538	VCC0538	VC2336	ygjH	tRNA-binding protein YgjH		
1	CDS	596969	596418	-	atgtacatctttggctatggaagc	MYIFGYGSLINSASRRLTGQT	fig|127906.87.peg.539	VCC0539	VC2335		hypothetical protein	gamma-glutamylcyclotransferase [Vibrio cholerae]	
1	CDS	597930	596989	-	atggagtttctcatcgatcactgg	MEFLIDHWRFGDSAPKRHPA	fig|127906.87.peg.540	VCC0540	VC2334	zntB	Magnesium and cobalt transport protein CorA		
1	repeat	598026	598157	+	gatattcccttcctacttgaagttgcagcggtgttggctacgttcgttcaccccaa		fig|127906.87.repeat.13				repeat region		
1	CDS	599613	598162	-	atggctaaccttttaatcgtgacc	MANLLIVTDQVSDWQQYFPSE	fig|127906.87.peg.541	VCC0541	VC2333	lysX_2	Ribosomal protein S6 modification protein-related protein		
1	CDS	599710	599808	+	atgctcatggggatgaattcactt	MLMGMNSLAAYLQLQVVWGY	fig|127906.87.peg.542	VCC0542	No refSeq annotation		hypothetical protein		
1	CDS	599818	600909	+	atggacattcgtcgtgccaatag	MDIRRANSADLNALNALEQQL	fig|127906.87.peg.543	VCC0543	VC2332	ypeA_1	GNAT family acetyltransferase VC2332		
1	CDS	601012	602256	+	ttggcttggtttgtgctcatcactgg	MAWFVLITGSVCDEVGEGAK	fig|127906.87.peg.544	VCC0544	VC2330		NnrS protein involved in response to NO		
1	CDS	603369	602338	-	atggcgtactttgctttaggtctag	MAYFALGLATATKNRDGKIIEA	fig|127906.87.peg.545	VCC0545	VC2329	dapD	2,3,4,5-tetrahydropyridine-2,6-dicarboxylate N-succinyltransferase (EC 2.3.1.117)		
1	repeat	603559	603686	+	atccccgtcctacttgaagttgtagcggtgttggctacgttcgttcaccccaatc		fig|127906.87.repeat.14				repeat region		
1	CDS	604084	603776	-	atggctgttatcgtgaagtacgtg	MAVIVKYVVERNGEEKMTFTS	fig|127906.87.peg.546	VCC0546	VC2326		YebG, DNA damage-inducible gene in SOS regulon, expressed in stationary phase		
1	CDS	605114	604200	-	atggctagccatatttcgctcaaa	MASHISLKQLRVFTTITQHKSL	fig|127906.87.peg.547	VCC0547	VC2324		Uncharacterized transcriptional regulator VC2324, LysR family	LysR family transcriptional regulator [Vibrio cholerae]	
1	CDS	605225	606193	+	atgattcgagcagcaaaagcga	MIRAAKAKVMGAPTPMAGLAL	fig|127906.87.peg.548	VCC0548	VC2323	tehA	Uncharacterized protein VC2323	TDT family transporter [Vibrio cholerae]	
1	repeat	606196	606323	+	atcccttcctacttgaagttgcagcggtgttggctacgttcgttcaccccaatc		fig|127906.87.repeat.15				repeat region		
1	CDS	606432	609878	+	gtgtttacggtctaccactccaac	MFTVYHSNQVETLKILLVHLIKI	fig|127906.87.peg.549	VCC0549	VC2322	recC	Exodeoxyribonuclease V gamma chain (EC 3.1.11.5)		
1	CDS	610143	613769	+	atgagtgccgattcaaataagct	MSADSNKLAVTLDTLRFPLHG	fig|127906.87.peg.550	VCC0550	VC2320	recB	Exodeoxyribonuclease V beta chain (EC 3.1.11.5)		
1	CDS	613766	615886	+	atgatgttttgggatagcgaagta	MMFWDSEVAKRLLAQGALRP	fig|127906.87.peg.551	VCC0551	VC2319	recD	Exodeoxyribonuclease V alpha chain (EC 3.1.11.5)		
1	CDS	616101	615916	-	atgaaaaccgttatttgtaattctc	MKTVICNSLQSFWDMADAEFL	fig|127906.87.peg.552	VCC0552	VC2318		hypothetical protein		
1	CDS	617665	616328	-	gtgaaaattcgcagtaccgctct	MKIRSTALVKGFRQSAPYVNA	fig|127906.87.peg.553	VCC0553	VC2316	argA	N-acetylglutamate synthase (EC 2.3.1.1)		
1	CDS	617934	618338	+	atggcaaaaacggaaagcaaa	MAKTESKSKALSKERLLLLLAI	fig|127906.87.peg.554	VCC0554	VC2314		hypothetical protein	DUF2850 domain-containing protein [Vibrio cholerae]	
1	CDS	618409	619515	+	gtgagtaaacgtcttctttctctcg	MSKRLLSLASLALLFGCAQQP	fig|127906.87.peg.555	VCC0555	VC2312	mltA	Membrane-bound lytic murein transglycosylase A		
1	CDS	619613	620428	+	atgcgtgaactcgatactcctgc	MRELDTPASDSYNQRFGGTR	fig|127906.87.peg.556	VCC0556	VC2311	moeB_1	HesA/MoeB/ThiF family protein => sulfur transfer pathway protein CsdL		
1	CDS	620436	620867	+	atgagtccatttcccgctcatccc	MSPFPAHPFGDDINAQTVLET	fig|127906.87.peg.557	VCC0557	VC2310	ygdK	Sulfur acceptor protein => sulfur transfer pathway protein CsdE		
1	CDS	622148	620937	-	atgctcaatcttgatgcaatccgc	MLNLDAIRAQFPALQQIVNGNI	fig|127906.87.peg.558	VCC0558	VC2309	sufS	Cysteine desulfurase (EC 2.8.1.7) => sulfur transfer pathway protein CsdA		
1	CDS	622740	622135	-	atgagcaaacgaattctcgtccc	MSKRILVPVAHGSEEMETVIIV	fig|127906.87.peg.559	VCC0559	VC2308	yajL/thiJ	DJ-1/YajL/PfpI superfamily, includes chaperone protein YajL (former ThiJ), parkinsonism-associated protein DJ-1, peptidases PfpI, Hsp31		
1	CDS	623627	622737	-	atgaatattgtggtacttggcccg	MNIVVLGPGAVGSLWALHLHS	fig|127906.87.peg.560	VCC0560	VC2307	panE	2-dehydropantoate 2-reductase (EC 1.1.1.169)		
1	CDS	624566	623766	-	atgcgtaaatcacttcttgctttaa	MRKSLLALSLLAATSAPVLAAD	fig|127906.87.peg.561	VCC0561	VC2305	tsx/ompK	Outer membrane protein OmpK		
1	CDS	624894	625475	+	atggaagagataatgttgtttcca	MEEIMLFPLSSVVLPEGKMKLI	fig|127906.87.peg.562	VCC0562	VC2303		Uncharacterized protein, similar to the N-terminal domain of Lon protease	ATP-dependent protease [Vibrio cholerae]	
1	CDS	625566	626150	+	atgtttaatccatcacctcatacgt	MFNPSPHTFGRQEWLECMEK	fig|127906.87.peg.563	VCC0563	VC2302	siqW	RNA polymerase ECF-type sigma factor		
1	CDS	626147	626827	+	atgagttatcaccctaattatgctt	MSYHPNYALLAAYAEGGLDSA	fig|127906.87.peg.564	VCC0564	VC2301		Putative transcriptional activator ChrR	transcriptional regulator [Vibrio cholerae]	
1	CDS	628303	626915	-	atggttcagcaaccccggtccca	MVQQPRSHSWLELIVSYFDRF	fig|127906.87.peg.565	VCC0565	VC2300		AmpG permease		
1	CDS	628920	628372	-	atggctaagtcatggctattggca	MAKSWLLATVALVSSCVWAG	fig|127906.87.peg.566	VCC0566	VC2299	ppiA	Peptidyl-prolyl cis-trans isomerase PpiA precursor (EC 5.2.1.8)		
1	CDS	629488	628913	-	atgaaaaaactggttcttgcggc	MKKLVLAASIALLTACSAPQQS	fig|127906.87.peg.567	VCC0567	VC2298		Hypothetical lipoprotein YajG precursor		
1	CDS	630660	629530	-	atgaaaactgagcttactttgcttg	MKTELTLLEQTLTLHRFPKRNI	fig|127906.87.peg.568	VCC0568	VC2297	rlmG	23S rRNA (guanine(1835)-N(2))-methyltransferase (EC 2.1.1.174)		
1	CDS	630825	631145	+	atgattcaagatgttattgaaaag	MIQDVIEKKLSDEFQPEFLKVI	fig|127906.87.peg.569	VCC0569	VC2296	bolA	Cell division protein BolA		
1	CDS	631582	632970	+	ttgcgcaatgaaagaatcttttc	MRNERIFFPIKVVQVRMITIKK	fig|127906.87.peg.570	VCC0570	VC2295	nqrA	Na(+)-translocating NADH-quinone reductase subunit A (EC 1.6.5.8)		
1	CDS	632976	634223	+	atgggccttaaaaagtttcttgaa	MGLKKFLEDIEHHFEPGGKHE	fig|127906.87.peg.571	VCC0571	VC2294	nqrB	Na(+)-translocating NADH-quinone reductase subunit B (EC 1.6.5.8)		
1	CDS	634210	634983	+	atggcaagcaataacgatagca	MASNNDSIKKTLFVVIALSLVC	fig|127906.87.peg.572	VCC0572	VC2293	nqrC	Na(+)-translocating NADH-quinone reductase subunit C (EC 1.6.5.8)		
1	CDS	634983	635615	+	atgtctagcgcaaaagagctga	MSSAKELKKSVLAPVLDNNPIA	fig|127906.87.peg.573	VCC0573	VC2292	nqrD	Na(+)-translocating NADH-quinone reductase subunit D (EC 1.6.5.8)		
1	CDS	635623	636219	+	atggaacattatattagtctgctgg	MEHYISLLVKSIFIENMALSFFL	fig|127906.87.peg.574	VCC0574	VC2291	nqrE	Na(+)-translocating NADH-quinone reductase subunit E (EC 1.6.5.8)		
1	CDS	636253	637479	+	atgtctactattatttttggtgtagtg	MSTIIFGVVMFTLIILALVLVILFA	fig|127906.87.peg.575	VCC0575	VC2290	nqrF	Na(+)-translocating NADH-quinone reductase subunit F (EC 1.6.5.8)		
1	CDS	637625	638629	+	gtgagaaattggcttgttgcatta	MRNWLVALASLLLLAGCEKPA	fig|127906.87.peg.576	VCC0576	VC2289	apbE	FAD:protein FMN transferase (EC 2.7.1.180) @ FAD:protein FMN transferase (EC 2.7.1.180), NqrBC-associated		
1	CDS	638646	638879	+	atgaacaccttttgattactttgg	MNTFLITFGFFLAVIAAMAVGY	fig|127906.87.peg.577	VCC0577	VC2288		Probable exported or periplasmic protein in ApbE locus	(Na+)-NQR maturation NqrM [Vibrio cholerae]	
1	CDS	638988	640070	+	atgcaagacagaattcgtaaaa	MQDRIRKIIHVDMDCFFAAVEI	fig|127906.87.peg.578	VCC0578	VC2287	dinB/dinP	DNA polymerase IV (EC 2.7.7.7)		
1	CDS	640316	641086	+	ttggcaaaaaaatcggccagaga	MAKNRPENTMKKSFLTLALLM	fig|127906.87.peg.579	VCC0579	VC2286		Methyl-accepting chemotaxis protein		
1	CDS	641150	642667	+	atgaatttaaataactttagcctac	MNLNNFSLRWLTTLNALAVVL	fig|127906.87.peg.580	VCC0580	VC2285		Diguanylate cyclase (EC 2.7.7.65)		
1	CDS	642824	644179	+	atggcacaatcaaattctcgtga	MAQSNSRETFSSRLGFILAAA	fig|127906.87.peg.581	VCC0581	VC2283		Sodium-dependent transporter		
1	CDS	645309	644248	-	gtggcaaaggctaaaaagcac	MAKAKKHGDFSFLGETIPPSS	fig|127906.87.peg.582	VCC0582	VC2282		FIG003737: Predicted deacylase	Succinylglutamate desuccinylase/aspartoacylase family protein [Vibrio cholerae]	
1	CDS	646201	645296	-	atgaaaattggtattctgtcgcgt	MKIGILSRNASLYSTKRLIEACI	fig|127906.87.peg.583	VCC0583	VC2281	lysX_1/rimK	Ribosomal protein S6--L-glutamate ligase		
1	CDS	646641	646198	-	atgaaagaaaaaatgattgtcg	MKEKMIVGWREELSLPGLGIE	fig|127906.87.peg.584	VCC0584	VC2280		hypothetical protein sometimes fused to ribosomal protein S6 glutaminyl transferase	ATP-dependent zinc protease	
1	CDS	648347	646875	-	gtgtctgagttccaaaccgaaat	MSEFQTEISKLSSNPIWPFFAT	fig|127906.87.peg.585	VCC0585	VC2279	pepD	Cytosol nonspecific dipeptidase (EC 3.4.13.18)	aminoacyl-histidine dipeptidase	
1	CDS	649226	650518	+	atgctggaaaagctttttaaactc	MLEKLFKLSEYGTNVRTEILAG	fig|127906.87.peg.586	VCC0586	VC2278	purP	Xanthine/uracil/thiamine/ascorbate permease family protein		
1	CDS	650556	651020	+	atgagcaaaaaattcgttatcac	MSKKFVITWDNMQHYCRELA	fig|127906.87.peg.587	VCC0587	VC2277	gpt	Xanthine-guanine phosphoribosyltransferase (EC 2.4.2.22)		
1	CDS	651117	652364	+	atgtcagaagcgagcagcaaa	MSEASSKNLSETLFQNHKQAI	fig|127906.87.peg.588	VCC0588	VC2276	frsA	Fermentation/respiration switch protein		
1	CDS	652498	652887	+	atgtcagagatgaccaaaaccc	MSEMTKTPTHYRLLSTLKAMG	fig|127906.87.peg.589	VCC0589	VC2275	crl	Curlin genes transcriptional activator		
1	CDS	653008	654141	+	atgaccacaaatcaacgaagc	MTTNQRSIKAPQTVVVKLGTS	fig|127906.87.peg.590	VCC0590	VC2274	proB	Glutamate 5-kinase (EC 2.7.2.11) / RNA-binding C-terminal domain PUA		
1	CDS	654153	655403	+	atggatttaaccgtactcggtaa	MDLTVLGKAAKAASFQLATAS	fig|127906.87.peg.591	VCC0591	VC2273	proA	Gamma-glutamyl phosphate reductase (EC 1.2.1.41)		
1	CDS	655503	655952	+	atgcattgtcctttctgttctgaaa	MHCPFCSENDTKVIDSRLVAD	fig|127906.87.peg.592	VCC0592	VC2272	nrdR	Ribonucleotide reductase transcriptional regulator NrdR		
1	CDS	655978	657081	+	atgcctatgtttacctcttttgatca	MPMFTSFDHQMMSRAIELAW	fig|127906.87.peg.593	VCC0593	VC2271	ribD	Diaminohydroxyphosphoribosylaminopyrimidine deaminase (EC 3.5.4.26) / 5-amino-6-(5-phosphoribosylamino)uracil reductase (EC 1.1.1.193)		
1	CDS	657086	657739	+	atgtttacaggtattgttgaagcg	MFTGIVEAVGKLTAIIPKGSDV	fig|127906.87.peg.594	VCC0594	VC2270	ribE	Riboflavin synthase eubacterial/eukaryotic (EC 2.5.1.9)		
1	CDS	657780	658889	+	atgccaattagcaccccacaag	MPISTPQEIIEDIRQGKMVILMI	fig|127906.87.peg.595	VCC0595	VC2269	ribBA	3,4-dihydroxy-2-butanone 4-phosphate synthase (EC 4.1.99.12) / GTP cyclohydrolase II (EC 3.5.4.25)		
1	CDS	659154	659624	+	atgaaagtgatcgagggtggttt	MKVIEGGFPAPNAKIAIVISRFN	fig|127906.87.peg.596	VCC0596	VC2268	ribH	6,7-dimethyl-8-ribityllumazine synthase (EC 2.5.1.78)		

1	CDS	659624	660094	+	atgggggccagtgtgaaacca	MGASVKPAARRNARQFALQA	fig|127906.87.peg.597	VCC0597	VC2267	nusB	Transcription termination protein NusB		
1	CDS	660185	661159	+	atgtttggtgaatttaatttaatcga	MFGEFNLIDKYFSNRQAQRKI	fig|127906.87.peg.598	VCC0598	VC2266	thiL	Thiamine-monophosphate kinase (EC 2.7.4.16)		
1	CDS	661156	661665	+	atgagcgatccacgcagtaatc	MSDPRSNLSLSNPWHLLATGI	fig|127906.87.peg.599	VCC0599	VC2265	pgpA	Phosphatidylglycerophosphatase A (EC 3.1.3.27)		
1	CDS	662089	661706	-	atgtttccccatctcgttggcttgg	MFPHLVGLGINEPTQIERYSLF	fig|127906.87.peg.600	VCC0600	VC2264		UPF0325 protein YaeH	DUF3461 family protein [Vibrio cholerae]	
1	repeat	662127	662260	+	gatataccctcctacttgaagctgcagcggtgttagctacgttcgttcacccca		fig|127906.87.repeat.16				repeat region		
1	CDS	664995	662365	-	atgccgtatcaatcccctatcaca	MPYQSPITFQEPQLTVESLKQ	fig|127906.87.peg.601	VCC0601	VC2262	glnD	[Protein-PII] uridylyltransferase (EC 2.7.7.59) / [Protein-PII]-UMP uridylyl-removing enzyme		
1	CDS	665942	665100	-	atgtctataaaaatcaagaatgc	MSIKIKNAVEIEKMRVAGRLAA	fig|127906.87.peg.602	VCC0602	VC2261	map	Methionine aminopeptidase (EC 3.4.11.18)		
1	CDS	666283	667011	+	atggcatctgtatcaatgcgcga	MASVSMRDMLTAGVHFGHQT	fig|127906.87.peg.603	VCC0603	VC2260	rpsB	SSU ribosomal protein S2p (SAe)		
1	CDS	667143	667985	+	atggctgttactgctgctctagtaa	MAVTAALVKELRERTGAGMM	fig|127906.87.peg.604	VCC0604	VC2259	tsf	Translation elongation factor Ts		
1	CDS	668173	668904	+	atgacaacgaaccctaaaccac	MTTNPKPAYQRILLKLSGEAL(	fig|127906.87.peg.605	VCC0605	VC2258	pyrH	Uridylate kinase (EC 2.7.4.22)		
1	CDS	668958	669515	+	gtgattaatgagatcaaaaaag	MINEIKKDAQERMEKSVEALK	fig|127906.87.peg.606	VCC0606	VC2257	frr	Ribosome recycling factor		
1	CDS	669607	670359	+	atgcaaaatctatctctttcccaa	MQNLSLSQDVLPKHIAVIMDG	fig|127906.87.peg.607	VCC0607	VC2256	ispU/uppS	Undecaprenyl diphosphate synthase (EC 2.5.1.31)		
1	CDS	670377	671219	+	ttgaagcagcgaatcattacagc	MKQRIITALILAPLVILGILYLPF	fig|127906.87.peg.608	VCC0608	VC2255	cdsA	Phosphatidate cytidylyltransferase (EC 2.7.7.41)		
1	CDS	671268	672476	+	atgcgtaaactaaccattttaggt	MRKLTILGATGSIGASTLKVIA	fig|127906.87.peg.609	VCC0609	VC2254	dxr	1-deoxy-D-xylulose 5-phosphate reductoisomerase (EC 1.1.1.267)		
1	CDS	672473	673831	+	atgaccgacatattgtggaacttt	MTDILWNFIAFIIALGILVAVHEF	fig|127906.87.peg.610	VCC0610	VC2253	rseP	Intramembrane protease RasP/YluC, implicated in cell division based on FtsL cleavage		
1	CDS	673880	676291	+	atggcgatgaaacagattctgct	MAMKQILLATLLATSVSANGAI	fig|127906.87.peg.611	VCC0611	VC2252	bamA	Outer membrane protein assembly factor YaeT		
1	CDS	676306	676815	+	ttgaaaaacatgatcaaagcag	MKNMIKAASLGLIILSSSMMAN	fig|127906.87.peg.612	VCC0612	VC2251	skp/ompH	Periplasmic chaperone of outer membrane proteins Skp @ Outer membrane protein H precursor		
1	CDS	676825	677880	+	atgaccacactcacttagccga	MTTLTLAELATITGGELFGDPT	fig|127906.87.peg.613	VCC0613	VC2250	lpxD	UDP-3-O-[3-hydroxymyristoyl] glucosamine N-acyltransferase (EC 2.3.1.191)		
1	CDS	677965	678417	+	ttgactactgataagaaaacgat	MTTDKKTMNITEIQSLLPHRYF	fig|127906.87.peg.614	VCC0614	VC2249	fabZ	3-hydroxyacyl-[acyl-carrier-protein] dehydratase, FabZ form (EC 4.2.1.59)		
1	CDS	678419	679207	+	atgattcacgaaactgcacaaa	MIHETAQIHPTSVVEEGAIIGAI	fig|127906.87.peg.615	VCC0615	VC2248	lpxA	Acyl-[acyl-carrier-protein]--UDP-N-acetylglucosamine O-acyltransferase (EC 2.3.1.129)		
1	CDS	679368	680507	+	gtgaatagaccattacggattgg	MNRPLRIGIVVGELSGDTLGE(	fig|127906.87.peg.616	VCC0616	VC2247	lpxB	Lipid-A-disaccharide synthase (EC 2.4.1.182)		
1	CDS	680532	681152	+	atggcgaagaaaccaagcattc	MAKKPSIELPPFEIPAGYALIA(	fig|127906.87.peg.617	VCC0617	VC2246	rnhB	Ribonuclease HII (EC 3.1.26.4)		
1	CDS	681191	684670	+	atgtcagacccaaagttcatcca	MSDPKFIHLRIHSDFSMVDGL	fig|127906.87.peg.618	VCC0618	VC2245	dnaE	DNA polymerase III alpha subunit (EC 2.7.7.7)		
1	CDS	684725	685684	+	atgagcctcaattttcttgattttga	MSLNFLDFEKPIVELETKIQALI	fig|127906.87.peg.619	VCC0619	VC2244	accA	Acetyl-coenzyme A carboxyl transferase alpha chain (EC 6.4.1.2)		
1	CDS	685824	687146	+	atggacgatctctatctccacttg	MDDLYLHFVQQLARYPFRKIL	fig|127906.87.peg.620	VCC0620	VC2242	tilS	tRNA(Ile)-lysidine synthetase (EC 6.3.4.19)		
1	CDS	687182	687505	+	atggggaaagcaatgaacaaa	MGKAMNKMVIALALGGCAMS	fig|127906.87.peg.621	VCC0621	VC2241		cytochrome c554		
1	CDS	687882	688406	+	atgagatttgaacgaaatgattta	MRFERNDLSGFLGKSFIYNYD	fig|127906.87.peg.622	VCC0622	VC2240	padC	Phenolic acid decarboxylase (EC 4.1.1.-)		
1	CDS	688566	688904	+	atgaaaaaaatcgaagccatca	MKKIEAIIKPFKLDDVREALAE	fig|127906.87.peg.623	VCC0623	VC2239	glnB-2	Nitrogen regulatory protein P-II		
1	CDS	688972	689817	+	atgaaaaaacgttactggatgg	MKKRYWMAPLLIAAAGLAAFV	fig|127906.87.peg.624	VCC0624	VC2238		UPF0294 protein YafD		
1	CDS	691432	689834	-	atgcgagttaaattcagttgggta	MRVKFSWVLALLLVGCQSPQ(	fig|127906.87.peg.625	VCC0625	VC2237	mltD	Membrane-bound lytic murein transglycosylase D		
1	CDS	692262	691504	-	atgttacagatcaaaagcatacc	MLQIKSIPAFDDNYIWLIQNRD	fig|127906.87.peg.626	VCC0626	VC2236	gloB	Hydroxyacylglutathione hydrolase (EC 3.1.2.6)		
1	CDS	692300	693037	+	atgaaaccagcacgcagtgata	MKPARSDKALENPHSWAQLK	fig|127906.87.peg.627	VCC0627	VC2235		FIG005121: SAM-dependent methyltransferase (EC 2.1.1.-)		
1	CDS	693578	693108	-	atgaacaaacaggtggaaattt	MNKQVEIFTDGSCLGNPGPG	fig|127906.87.peg.628	VCC0628	VC2234	rnhA	Ribonuclease HI (EC 3.1.26.4)		
1	CDS	693635	694378	+	atgaatactagcaacaatgcag	MNTSNNAEYQRIVVLDTETTG	fig|127906.87.peg.629	VCC0629	VC2233	dnaQ	DNA polymerase III epsilon subunit (EC 2.7.7.7)		
1	CDS	694387	695646	+	atgttgaggattctcgcggcgata	MLRILAAIVGCLISLSSLAAQQS	fig|127906.87.peg.630	VCC0630	VC2232		FIG004599: Hypothetical protein	TIGR03503 family protein [Vibrio cholerae]	
1	CDS	698237	695793	-	atggaaatcttgctctctaccttaa	MEILLSTLIMLLILGACLYQRTS	fig|127906.87.peg.631	VCC0631	VC2231	fadE	Acyl-coenzyme A dehydrogenase FadE (EC 1.3.8.-)		
1	CDS	698555	699130	+	atgtaccaggatttaattagaagt	MYQDLIRSELTEAADVLQKFL	fig|127906.87.peg.632	VCC0632	VC2230	gmhA	D-sedoheptulose 7-phosphate isomerase (EC 5.3.1.28)		
1	CDS	699193	700038	+	atgtgtgaattgctcggcatgag	MCELLGMSANVPTDICFSFTG	fig|127906.87.peg.633	VCC0633	VC2229		Putative glutamine amidotransferase YafJ	class II glutamine amidotransferase [Vibrio cholerae]	
1	CDS	700008	700124	+	atgtcaggcaccaagccaacct	MSGTKPTSRLSDIPFLLEAAA\	fig|127906.87.peg.634	VCC0634	Not annotated as CDS or not present		hypothetical protein		
1	CDS	700817	700179	-	atgaagagtattgttgttttaatttc	MKSIVVLISGNGTNLQAIIDAC	fig|127906.87.peg.635	VCC0635	VC2227	purN	Phosphoribosylglycinamide formyltransferase (EC 2.1.2.2)		
1	CDS	701860	700820	-	gtgagcggtaataatccatctctc	MSGNNPSLSYKDAGVDIDAG	fig|127906.87.peg.636	VCC0636	VC2226	purM	Phosphoribosylformylglycinamidine cyclo-ligase (EC 6.3.3.1)		
1	CDS	702027	702653	+	atgaaaattgttgaagttaagca	MKIVEVKHPLVKHKLGLMREG	fig|127906.87.peg.637	VCC0637	VC2225	upp	Uracil phosphoribosyltransferase (EC 2.4.2.9)		
1	CDS	702673	702801	+	atggcttcctcattatctttcaccgt	MASSLSFTVHSPYPTANSDTP	fig|127906.87.peg.638	VCC0638	Not annotated as CDS or not present		hypothetical protein		
1	CDS	703001	704422	+	atgacgcgaaagaaatttagttg	MTRKKFSWILVTLIIFGFFVTSL	fig|127906.87.peg.639	VCC0639	VC2224		GGDEF domain protein		
1	CDS	705506	704520	-	atgtcccaaccgactgaaaaac	MSQPTEKRLNKFISETGYCSR	fig|127906.87.peg.640	VCC0640	VC2223	rluF	LSU rRNA pseudouridine(2604) synthase (EC 5.4.99.21)		
1	CDS	706150	705572	-	atgtcgcaagaggatttagaact	MSQEDLELFQQMMGDVKTIT	fig|127906.87.peg.641	VCC0641	VC2222	smrA	Uncharacterized protein VC_2222	DNA endonuclease SmrA [Vibrio cholerae]	
1	CDS	706472	706332	-	atggacttctggctcgatcttctttt	MDFWLDLLFGNAVGLSSMIVII	fig|127906.87.peg.642	VCC0642	VC2221		Uncharacterized protein VC2221	DUF3149 domain-containing protein [Vibrio]	
1	rna	706702	706778	+	cggtgaatagcgcagtttggtagcgcatctggtttgggaccagagggtcggg		fig|127906.87.rna.26				tRNA-Pro-TGG		
1	CDS	707819	706953	-	atgctcacacgattcgcttgctgt	MLTRFACCLLWLFIQPLHAEPI	fig|127906.87.peg.643	VCC0643	VC2220		hypothetical protein	TraB/GumN family protein [Vibrio cholerae]	
1	CDS	707922	708038	+	ttgtccgagccaactcacgatttg	MSEPTHDLLPQSCVMSIGFLC	fig|127906.87.peg.644	VCC0644	VC2219		hypothetical protein		
1	CDS	708233	708120	-	atgctctgcctgcacccaatctca	MLCLHPISQRHQPMALPKIQG	fig|127906.87.peg.645	VCC0645	VC2218		FIG01203553: hypothetical protein		
1	CDS	708266	710917	+	atgctgaagaagagtcttatctcg	MLKKSLISIAVFTSLSGCALNT	fig|127906.87.peg.646	VCC0646	VC2217	chb	beta-N-acetylglucosaminidase (EC 3.2.1.52)		
1	CDS	711494	711054	-	atgaaacgcacaatgatgacag	MKRTMMTLALLATFSGQALAT	fig|127906.87.peg.647	VCC0647	VC2216		CopG protein		
1	CDS	714223	711503	-	atgaaccattttgctttagcgctgc	MNHFALALRGLNCMGCARKL	fig|127906.87.peg.648	VCC0648	VC2215		Lead, cadmium, zinc and mercury transporting ATPase (EC 3.6.3.3) (EC 3.6.3.5); Copper-translocating P-type ATPase (EC 3.6.3.4)		
1	CDS	714424	715848	+	atgacggttaaaactcgttttgcg	MTVKTRFAPSPTGYLHVGGAF	fig|127906.87.peg.649	VCC0649	VC2214	gltX	Glutamyl-tRNA synthetase (EC 6.1.1.17)		
1	CDS	716144	717109	+	atgaaaaagctagcggcaatta	MKKLAAIISATLLFASSAAVAE\	fig|127906.87.peg.650	VCC0650	VC2213	ompA	Outer membrane protein A precursor		
1	CDS	717896	717198	-	atgaatatagataaacactgtga	MNIDKHCDWGANVANTATAS	fig|127906.87.peg.651	VCC0651	VC2212		Hypothetical protein, specific for Vibrio	Fe3+-citrate ABC transporter substrate-binding protein [Vibrio cholerae]	Limited query cover for certain strains
1	CDS	718316	720379	+	atggcagttttatgcccagcgcg	MAVLCPARVSVAENKKFKLHT	fig|127906.87.peg.652	VCC0652	VC2211	viuA	Ferric vibriobactin receptor ViuA		
1	CDS	720683	721498	+	atgagtaacgaagtcgagcgcc	MSNEVERVYPRLLDFVRKKY\	fig|127906.87.peg.653	VCC0653	VC2210	viuB	Vibriobactin utilization protein ViuB		
1	CDS	721744	728997	+	gtgggtttaaagatgaaagaaa	MGLKMKEMTAMQAAYWLGR	fig|127906.87.peg.654	VCC0654	VC2209	vibF	Non-ribosomal peptide synthetase modules, siderophore biosynthesis		vibF split into 2 ORF in N16961 in PATRIC. Annotated as 1 ORF in KEGG.
1	CDS	730260	729127	-	atgaaaaaaaatattttatctatc	MKKNILSIISALWMMLQLPSAH	fig|127906.87.peg.655	VCC0655	VC2208	FlgT	Flagellar protein FlgT		
1	CDS	730388	731056	+	atgcgcgccatagtatatctgga	MRAIVYLEMKRMKKWLSLVP\	fig|127906.87.peg.656	VCC0656	VC2207	FlgO	Flagellar protein FlgO		Limited query cover for certain strains
1	CDS	731067	731501	+	atgaaatctagcttactactccta	MKSSLLLLVAALMMTGCQPLC	fig|127906.87.peg.657	VCC0657	VC2206	FlgP	Flagellar protein FlgP		
1	CDS	732036	731611	-	atggcagcattaaaagacttggt	MAALKDLVEFQLKNAQELVTV	fig|127906.87.peg.658	VCC0658	VC2205	FlgN	Flagellar biosynthesis protein FlgN		
1	CDS	732463	732140	-	atggcaggtattgataacatacg	MAGIDNIRAGQSLNTTSRTNV	fig|127906.87.peg.659	VCC0659	VC2204	FlgM	Negative regulator of flagellin synthesis FlgM (anti-sigma28)		
1	CDS	733337	732594	-	atgcatccgaagatcgatctcca	MHPKIDLHSHFITKCRAFCEIF	fig|127906.87.peg.660	VCC0660	VC2203	FlgA	Flagellar basal-body P-ring formation protein FlgA		Limited query cover for certain strains
1	CDS	733418	734344	+	atgacgggtattcttgattctgtga	MTGILDSVNQRTQLVGQNRLE	fig|127906.87.peg.661	VCC0661	VC2202	CheV	Chemotaxis protein CheV (EC 2.7.3.-)		
1	CDS	734355	735182	+	atgactgctataacgattagcga	MTAITISDQEYRDFCRFLESQ(	fig|127906.87.peg.662	VCC0662	VC2201	CheR_2	Chemotaxis protein methyltransferase CheR (EC 2.1.1.80)		
1	CDS	735402	735797	+	atggctatttcttttgacagagccc	MAISFDRALGIHQHTVGVRER	fig|127906.87.peg.663	VCC0663	VC2200	FlgB	Flagellar basal-body rod protein FlgB		
1	CDS	735802	736218	+	atgagcttattcagcgtatttaatg	MSLFSVFNVTGSAMSAESVRL	fig|127906.87.peg.664	VCC0664	VC2199	FlgC	Flagellar basal-body rod protein FlgC		
1	CDS	736236	736943	+	atggccggagtcaataatgttgg	MAGVNNVGQSGLSYIDQLKAI	fig|127906.87.peg.665	VCC0665	VC2198	FlgD	Flagellar basal-body rod modification protein FlgD		
1	CDS	736972	738276	+	atgtcatatgtctctcttagtggtta	MSYVSLSGLSAAQMDLNTTSN	fig|127906.87.peg.666	VCC0666	VC2197	FlgE	Flagellar hook protein FlgE		
1	CDS	738459	739208	+	atggaccgcgcattgtttcttgcc	MDRALFLAMSGAKQNMQAM(	fig|127906.87.peg.667	VCC0667	VC2196	FlgF	Flagellar basal-body rod protein FlgF		
1	CDS	739228	740016	+	atgcaaccagcactatgggtca	MQPALWVSKTGLDAQQTNIA	fig|127906.87.peg.668	VCC0668	VC2195	FlgG	Flagellar basal-body rod protein FlgG		
1	CDS	740040	740816	+	atgaaacgtctacttgcttctagc	MKRLLASSLLILLSGCSLMQPF	fig|127906.87.peg.669	VCC0669	VC2194	FlgH	Flagellar L-ring protein FlgH		
1	CDS	740907	741992	+	atgagaaaattcacgattttattg	MRKFTILLMLLLASSAQAARIK	fig|127906.87.peg.670	VCC0670	VC2193	FlgL	Flagellar P-ring protein FlgI		
1	CDS	742003	742941	+	atgattaataattccaatgacatt	MINNSNDIGFIQDIAGLDKLRQ	fig|127906.87.peg.671	VCC0671	VC2192	FlgJ	Flagellar protein FlgJ [peptidoglycan hydrolase]		
1	CDS	743121	744995	+	atggcgtctgatcttctgaatgtg	MASDLLNVGTQSVLTAQRQLN	fig|127906.87.peg.672	VCC0672	VC2191	FlgK	Flagellar hook-associated protein FlgK		
1	CDS	745008	746201	+	atgttaaaccgaatctctagcttc	MLNRISSFHNYQAVQNDIRRQ	fig|127906.87.peg.673	VCC0673	VC2190	FlgL	Flagellar hook-associated protein FlgL		
1	CDS	746341	746460	+	ttgccgctcaccgaaatcggcaa	MPLTEIGNFEWLIELDRQCNS	fig|127906.87.peg.674	VCC0674	Not annotated as CDS		FIG01202338: hypothetical protein		
1	CDS	746609	747748	+	atgaccattaacgtaaataccaa	MTINVNTNVSAMTAQRYLTKA	fig|127906.87.peg.675	VCC0675	VC2188		Flagellin protein FlaC		Partly overlaps with FlaD
1	CDS	748303	749436	+	atggcggtgaatgtaaacacca	MAVNVNTNVSAMTAQRYLTS	fig|127906.87.peg.676	VCC0676	VC2187		Flagellin protein FlaD		
1	rna	749804	749720	-	gcggaagtggcgaaattggtagacgcaccagatttaggttctggcgccgcg		fig|127906.87.rna.27	VCCt014	VCt081		tRNA-Leu-TAG		
1	rna	749920	749846	-	aggtctgtcgccaagcggtaaggcaacgggttttgatcccgtcatacctagg		fig|127906.87.rna.28	VCCt015	VCt080		tRNA-Gln-TTG		
1	rna	750043	749959	-	gcggaagtggcgaaattggtagacgcaccagatttaggttctggcgccgcg		fig|127906.87.rna.29	VCCt016	VCt079		tRNA-Leu-TAG		
1	rna	750161	750087	-	aggtctgtcgccaagcggtaaggcaacgggttttgatcccgtcatacccagg		fig|127906.87.rna.30	VCCt017	VCt078		tRNA-Gln-TTG		
1	rna	750264	750179	-	gcggtggtggcggaattggtagacgcgctagcttcaggtgttagtgtccttagg		fig|127906.87.rna.31	VCCt018	VCt077		tRNA-Leu-CAG		
1	rna	750363	750287	-	ggctacgtagctcagttggttagagcacatcactcataatgatggggtcacag		fig|127906.87.rna.32	VCCt019	VCt076		tRNA-Met-CAT		
1	rna	750473	750389	-	gcggaagtggcgaaattggtagacgcaccagatttaggttctggcgccgcg		fig|127906.87.rna.33	VCCt020	VCt075		tRNA-Leu-TAG		
1	rna	750589	750515	-	aggtctatcgccaagcggtaaggcaacgggttttgatcccgtcatcccatggt		fig|127906.87.rna.34	VCCt021	VCt074		tRNA-Gln-TTG		
1	rna	750700	750616	-	gcggaagtggcgaaattggtagacgcaccagatttaggttctggcgccgcg		fig|127906.87.rna.35	VCCt022	VCt073		tRNA-Leu-TAG		
1	rna	750816	750742	-	aggtctgtcgccaagcggtaaggcaacgggttttgatcccgtcatacctagg		fig|127906.87.rna.36	VCCt023	VCt072		tRNA-Gln-TTG		
1	rna	750919	750834	-	gcggtggtggcggaattggtagacgcgctagcttcaggtgttagtgtccttagg		fig|127906.87.rna.37	VCCt024	VCt071		tRNA-Leu-CAG		
1	rna	751019	750943	-	ggctacgtagctcagttggttagagcacatcactcataatgatggggtcacag		fig|127906.87.rna.38	VCCt025	VCt070		tRNA-Met-CAT		
1	rna	751171	751097	-	aggtctgtcgccaagcggtaaggcaacgggttttgatcccgtcatacctagg		fig|127906.87.rna.39	VCCt026	VCt069		tRNA-Gln-TTG		
1	rna	751283	751199	-	gcggaagtggcgaaattggtagacgcaccagatttaggttctggcgccgca		fig|127906.87.rna.40	VCCt027	VCt068		tRNA-Leu-TAG		
1	rna	751393	751308	-	gcggtggtggcggaattggtagacgcgctagcttcaggtgttagtgtccttcg		fig|127906.87.rna.41	VCCt028	VCt067		tRNA-Leu-CAG		
1	rna	751498	751422	-	ggctacgtagctcagttggttagagcacatcactcataatgatggggtcacag		fig|127906.87.rna.42	VCCt029	VCt066		tRNA-Met-CAT		
1	CDS	751667	751512	-	ttggtgatggtttttgatcataaac	MVMVFDHKHASLFKKNPNGW	fig|127906.87.peg.677	VCC0677	Not annotated as CDS		hypothetical protein		
1	CDS	752867	751776	-	atgggttttaaatgtggcatcgtcg	MGFKCGIVGLPNVGKSTLFNA	fig|127906.87.peg.678	VCC0678	VC2185	ychF	GTP-binding and nucleic acid-binding protein YchF		
1	CDS	753468	752878	-	ttgagtcaaccaatcaaactcctt	MSQPIKLLVGLANPGPEYAKT	fig|127906.87.peg.679	VCC0679	VC2184	pth	Peptidyl-tRNA hydrolase (EC 3.1.1.29)		
1	CDS	754581	753637	-	gtgcctgatatgaagctatttgctg	MPDMKLFAGNAIPELAQRIAD	fig|127906.87.peg.680	VCC0680	VC2183	prsA	Ribose-phosphate pyrophosphokinase (EC 2.7.6.1)		
1	CDS	755484	754612	-	atgatccacggcacaaccgtgt	MIHGTTVWPSPAKLNLFLYITG	fig|127906.87.peg.681	VCC0681	VC2182	ipk	4-diphosphocytidyl-2-C-methyl-D-erythritol kinase (EC 2.7.1.148)		
1	CDS	756098	755481	-	atgaatgctctcgtacgtcgccta	MNALVRRLLPGLASLLLLAGC	fig|127906.87.peg.682	VCC0682	VC2181	lolB	Outer membrane lipoprotein component of lipoprotein transport system LolB		
1	CDS	756257	757516	+	atgtctttgcttgccattggaatca	MSLLAIGINHNTASVELREKVA	fig|127906.87.peg.683	VCC0683	VC2180	hemA	Glutamyl-tRNA reductase (EC 1.2.1.70)		
1	CDS	757546	758634	+	atgaaagcgtcgattttaagcaa	MKASILSKLESLVERYEEVQHI	fig|127906.87.peg.684	VCC0684	VC2179	prfA	Peptide chain release factor 1		
1	CDS	758638	759498	+	atgtcagttaccattgaagcggc	MSVTIEAALKAATEQLQQSGS	fig|127906.87.peg.685	VCC0685	VC2178	prmC/hemK	Peptide chain release factor N(5)-glutamine methyltransferase (EC 2.1.1.297)		
1	CDS	759498	759881	+	atgtacgaaggattaaaacattt	MYEGLKHFHLFTIGVSVVMLS	fig|127906.87.peg.686	VCC0686	VC2177	sirB2	FIG002082: Protein SirB2		
1	CDS	759897	760709	+	atgctgaatttatgtgatgaagat	MLNLCDEDFDAMELVEGALAL	fig|127906.87.peg.687	VCC0687	VC2176	sirB1	FIG002708: Protein SirB1		
1	CDS	760729	761580	+	atggaacacaaaattgtccatgt	MEHKIVHVGDIPVANDKPFTLF	fig|127906.87.peg.688	VCC0688	VC2175	kdsA	2-Keto-3-deoxy-D-manno-octulosonate-8-phosphate synthase (EC 2.5.1.55)		
1	repeat	761635	761770	+	catgtctataccctcctacttgaagctgcagcggtgttggctacgttcgttcacc		fig|127906.87.repeat.17				repeat region		
1	CDS	762122	763783	+	atgaaacaaggcctcattctaaa	MKQGLILKSVLSAAIIASLAGC/	fig|127906.87.peg.689	VCC0689	VC2174	ushA	UDP-sugar hydrolase (EC 3.6.1.45); 5'-nucleotidase (EC 3.1.3.5)		
1	CDS	763917	764399	+	atgactcctgcaatcaatcttgca	MTPAINLAKKNKISHTIHQYEH	fig|127906.87.peg.690	VCC0690	VC2172	ybaK	Cys-tRNA(Pro) deacylase YbaK		
1	CDS	765723	764470	-	atgaaaaatgccatacaaggcc	MKNAIQGAQMLFVAFGALVLV	fig|127906.87.peg.691	VCC0691	VC2171	pyrP/uraA	Uracil permease @ Uracil:proton symporter UraA		
1	CDS	765944	767092	+	atgcgctatttagctgtgttgatga	MRYLAVLMMGLLTFPAFALTR	fig|127906.87.peg.692	VCC0692	VC2168		hypothetical protein	DUF2066 domain-containing protein [Vibrio cholerae]	
1	CDS	767736	767299	-	atgaccacctccagtattgcaat	MTTSSIAMQPRTAFYRWLALS	fig|127906.87.peg.693	VCC0693	VC2167		hypothetical protein	DUF2069 domain-containing protein [Vibrio cholerae]	
1	CDS	768299	767739	-	atgatttcgatagtggtgctctatta	MISIVVLYYSRHGSTQALARQI	fig|127906.87.peg.694	VCC0694	VC2166	wrbA	Trp repressor-binding protein		
1	CDS	768646	768296	-	atgtcagtcgtgatttatcacaatc	MSVVIYHNPKCSKSRETLALLE	fig|127906.87.peg.695	VCC0695	VC2165	arsC	Uncharacterized protein YfgD, not an arsenate reductase	arsenate reductase (glutaredoxin) [Vibrio cholerae]	
1	CDS	770123	768669	-	atgaaattcttcccgacacgtacc	MKFFPTRTLLCLCIAAPCLPAI/	fig|127906.87.peg.696	VCC0696	VC2164	yfgC_1	Exported zinc metalloprotease YfgC precursor		
1	CDS	770307	770543	+	atggaaccgattattctcgatttgc	MEPIILDLREQRCPMTLLLVKR	fig|127906.87.peg.697	VCC0697	VC2163		FIG028274: hypothetical protein	sulfurtransferase TusA family protein [Vibrio cholerae]	
1	CDS	770536	771600	+	atgtttgaaatggtcagtcgttggt	MFEMVSRWYKRRFSDPHAAS	fig|127906.87.peg.698	VCC0698	VC2162	tqsA	Putative permease PerM (= YfgO)		
1	CDS	771791	773662	+	atgaggtttagtcagaagatcgtt	MRFSQKIVAASSALLLCVIALL	fig|127906.87.peg.699	VCC0699	VC2161		Methyl-accepting chemotaxis sensor/transducer protein		
1	CDS	774231	773764	-	atgaatacgcttactgctggaac	MNTLTAGTPAPAFSLPDQNGN	fig|127906.87.peg.700	VCC0700	VC2160	bcp	Thiol peroxidase, Bcp-type (EC 1.11.1.15)		
1	CDS	774788	774246	-	atgactcagcatcttgtgatcacc	MTQHLVITAVGTDRPGICNEV	fig|127906.87.peg.701	VCC0701	VC2159	gcvR	Glycine cleavage system transcriptional antiactivator GcvR		
1	CDS	775066	775944	+	atgttttcaggaagtatcgttgcgt	MFSGSIVALITPFTPDGEVDYIS	fig|127906.87.peg.702	VCC0702	VC2157	dapA	4-hydroxy-tetrahydrodipicolinate synthase (EC 4.3.3.7)		
1	CDS	776002	777021	+	atgaagttttctcgccagctagttg	MKFSRQLVLGSLAVLVLSACS	fig|127906.87.peg.703	VCC0703	VC2156	bamC/nlpB	Outer membrane beta-barrel assembly protein BamC		
1	CDS	777326	777114	-	atggagatactgtttaaccctgg	MEILFNPWVISLIVVAVIVGNIA	fig|127906.87.peg.704	VCC0704	VC2154		hypothetical protein	DUF2897 family protein [Vibrio cholerae]	
1	CDS	778002	777328	-	atgaccccagagcagctgacc	MTPEQLTGQTDTHLISVMVGC	fig|127906.87.peg.705	VCC0705	VC2153	vanYB	FIG009095: D,D-carboxypeptidase family protein		
1	CDS	779138	778005	-	atgacagatagccctgtactggc	MTDSPVLALAKELISRQSVTPA	fig|127906.87.peg.706	VCC0706	VC2152	dapE	N-succinyl-L,L-diaminopimelate desuccinylase (EC 3.5.1.18)		
1	CDS	779516	779169	-	atgacgatcactttgtatggcattc	MTITLYGIPNCDTMKKARKWL	fig|127906.87.peg.707	VCC0707	VC2150	spxA	FIG138056: a glutathione-dependent thiol reductase		
1	CDS	779767	780054	+	atggaactgagtcctgtattcgca	MELSPVFARRLYLALLVESLEF	fig|127906.87.peg.708	VCC0708	VC2149		hypothetical protein	helix-turn-helix domain-containing protein [Vibrio cholerae]	
1	CDS	780477	780121	-	atgaaaaacacgactctcattcc	MKNTTLIPSEQTQQEALKIAKA	fig|127906.87.peg.709	VCC0709	VC2147		hypothetical protein	MULTISPECIES: DUF2956 domain-containing protein [Vibrio]	
1	CDS	780621	781085	+	gtgcgttattcgattgaacagtatc	MRYSIEQYDSQGFLKAPIWLW	fig|127906.87.peg.710	VCC0710	VC2146	yfeZ	Membrane protein	DUF2919 domain-containing protein [Vibrio cholerae]	
1	CDS	781151	782059	+	atgtttaagtcacagaccgcaat	MFKSQTAILPEAGPFALYTLLK	fig|127906.87.peg.711	VCC0711	VC2145	yfeX	Predicted dye-decolorizing peroxidase (DyP), YfeX-like subgroup		
1	CDS	782183	783319	+	atggccatgacggtaaataccaa	MAMTVNTNVSALVAQRHLNSA	fig|127906.87.peg.712	VCC0712	VC2144	FlaF	Flagellin protein FlaF		
1	CDS	783609	784742	+	atggcagtgaatgtaaataccaa	MAVNVNTNVAAMTAQRYLTG	fig|127906.87.peg.713	VCC0713	VC2143	FlaD	Flagellin protein FlaD		
1	CDS	784978	786108	+	atggcaattaatgtaaacacgaa	MAINVNTNVSAMTAQRYLNGA	fig|127906.87.peg.714	VCC0714	VC2142	FlaA	Flagellin protein FlaA		
1	CDS	786182	786643	+	atggaaataccatcctacgcatc	MEIPSYASNIQPYGSQSGTKIA	fig|127906.87.peg.715	VCC0715	VC2141	FlaG	Flagellar protein FlaG		
1	CDS	786665	788665	+	atgagtttaggcccgatggggat	MSLGPMGMNTGFDINGMVSK	fig|127906.87.peg.716	VCC0716	VC2140	FliD	Flagellar cap protein FliD		
1	CDS	788667	788972	+	gtggaagcagaactgcaacgtt	MEAELQRLSDLDQQIATLLAE	fig|127906.87.peg.717	VCC0717	VC2139		Polar flagellar rod protein FlaI		
1	CDS	788983	789393	+	atgcgtggttctttacaggcgtac	MRGSLQAYKKVSVDSQLSAA	fig|127906.87.peg.718	VCC0718	VC2138	fliS	Flagellar biosynthesis protein FliS		
1	CDS	789625	791091	+	atgcagagtttagcgaaactact	MQSLAKLLVIEDDAAIRLNLSV	fig|127906.87.peg.719	VCC0719	VC2137	ntrC/flrA	Flagellar regulatory protein FleQ		
1	CDS	791206	792258	+	atgagcagcgcagtgcaagag	MSSAVQEQHSHLDSLEDQVE	fig|127906.87.peg.720	VCC0720	VC2136	flrB	Flagellar sensor histidine kinase FleS		
1	CDS	792261	793694	+	atggctcaaagcaaagtcttaat	MAQSKVLIVEDDEGLREALIDT	fig|127906.87.peg.721	VCC0721	VC2135	flrC	Flagellar two-component response regulator FleR		
1	CDS	793770	794081	+	atgagacttgacggtttaaataa	MRLDGLNNEMQAMMFEAMN	fig|127906.87.peg.722	VCC0722	VC2134	fliE	Flagellar hook-basal body complex protein FliE		
1	CDS	794097	795839	+	gtggccgatgataaacaacaa	MADDKQTTDLALTNSGGEHA	fig|127906.87.peg.723	VCC0723	VC2133	FliF	Flagellar M-ring protein FliF		
1	CDS	795832	796878	+	atggctaaagacaataaagatg	MAKDNKDGGEVVESTIDISEIF	fig|127906.87.peg.724	VCC0724	VC2132	FliG	Flagellar motor switch protein FliG		
1	CDS	796971	797771	+	atgtctggtgaaagaaagcgtg	MSGERKRGFIRPGTDDATVTF	fig|127906.87.peg.725	VCC0725	VC2131	FliH	Flagellar assembly protein FliH		
1	CDS	797775	799094	+	atgttagcgttagctgatcgcctt	MLALADRLAQYKVEGHTTRPN	fig|127906.87.peg.726	VCC0726	VC2130	FliI	Flagellum-specific ATP synthase FliI		
1	CDS	799109	799552	+	atggataacgcgttagatttcttg	MDNALDFLLEQAQESEDKAVL	fig|127906.87.peg.727	VCC0727	VC2129	FliJ	Flagellar protein FliJ		
1	CDS	799695	799573	-	atgacctgcgcagatcaactcat	MTCADQLISNSLGLTPFCENL/	fig|127906.87.peg.728	VCC0728	Not annotated as CDS		FIG01201841: hypothetical protein	PTS glucose transporter subunit IIABC [Vibrio cholerae]	
1	CDS	799731	801740	+	atgaatgtgaatctcaccctcc	MNVNLTPPVEPSKIAPTAKSS/	fig|127906.87.peg.729	VCC0729	VC2128	FliK	Flagellar hook-length control protein FliK		
1	CDS	801775	802278	+	atggccgaagaagatctaggaa	MAEEDLGTEAPKGKKKLLIIIIA	fig|127906.87.peg.730	VCC0730	VC2127	FliL	Flagellar basal body-associated protein FliL		
1	CDS	802286	803335	+	gtgactgatttattaagccaaga	MTDLLSQDEIDALLHGVDSVD	fig|127906.87.peg.731	VCC0731	VC2126	FliM	Flagellar motor switch protein FliM		
1	CDS	803406	803816	+	atggcaaaaagcgaagatgaa	MAKSEDERLAEEWAAALGED	fig|127906.87.peg.732	VCC0732	VC2125	FliN	Flagellar motor switch protein FliN		
1	CDS	803820	804209	+	atgcgaaaattggcggctcttgtt	MRKLAALFLIPVPGLAATSSQL	fig|127906.87.peg.733	VCC0733	VC2124	FliO	Flagellar biosynthesis protein FliO		
1	CDS	804187	805086	+	atgaaacggactcaaagactta	MKRTQRLNLTSWLTTGLLTWI	fig|127906.87.peg.734	VCC0734	VC2123	FliP	Flagellar biosynthesis protein FliP		
1	CDS	805099	805368	+	atgacaccagagatatttgttgag	MTPEIFVELFKESLWLVLIMVC	fig|127906.87.peg.735	VCC0735	VC2122	FliQ	Flagellar biosynthesis protein FliQ		
1	CDS	805377	806159	+	atggaatatcccgccagtgtcgt	MEYPASVVLDFIANYFWPYTR	fig|127906.87.peg.736	VCC0736	VC2121	FliR	Flagellar biosynthesis protein FliR		
1	CDS	806169	807299	+	ttggcagaatccgacggacaag	MAESDGQERTEEATPRRLQQ	fig|127906.87.peg.737	VCC0737	VC2120	FlhB	Flagellar biosynthesis protein FlhB		Limited query cover for selected strains
1	CDS	807867	807307	-	atgagcaaaaacgatcaccga	MSKNDHRITHNKSDKDNLDDI	fig|127906.87.peg.738	VCC0738	VC2119	SmrB	UPF0115 protein YfcN	endonuclease SmrB [Vibrio cholerae]	
1	CDS	807934	808866	+	ttggataagattttcgttgaggaa	MDKIFVEEAVSELHTLQDMIRV	fig|127906.87.peg.739	VCC0739	VC2118	prmB	Ribosomal protein L3 N(5)-glutamine methyltransferase (EC 2.1.1.298)		
1	CDS	809096	810181	+	atggcaggaaacagtatcggac	MAGNSIGQHFRVTTFGESHGI	fig|127906.87.peg.740	VCC0740	VC2116	aroC	Chorismate synthase (EC 4.2.3.5)		

1	CDS	810886	810263	-	atgctgctgagtgttttgtatatcat	MLLSVLYIIGITAEAMTGALSAC	fig|127906.87.peg.741	VCC0741	VC2115		UPF0126 inner membrane protein YicG	trimeric intracellular cation channel family protein [Vibrio]	
1	CDS	811250	811780	+	atgaaccatcaataccaagacc	MNHQYQDLIDIFNQTFYADYN	fig|127906.87.peg.742	VCC0742	VC2113	epmC	Translation elongation factor P Lys34 hydroxylase		
1	CDS	811819	812082	+	atgattattgaatttgaagagaaa	MIIEFEEKLLDLIDAQVVNASSD	fig|127906.87.peg.743	VCC0743	VC2112		Uncharacterized protein YfcL	YfcL family protein [Vibrio cholerae]	
1	CDS	812290	812817	+	atgaaaattattgccttcggtgcc	MKIIAFGASTSSTSINKALATFT	fig|127906.87.peg.744	VCC0744	VC2111		hypothetical protein	NAD(P)H-dependent oxidoreductase [Vibrio cholerae]	
1	CDS	814911	812896	-	atgtcatccatttcacacgcccag	MSSISHAQLGWNDAGTPVSD	fig|127906.87.peg.745	VCC0745	VC2110	mnmC	tRNA (5-methylaminomethyl-2-thiouridylate)-methyltransferase (EC 2.1.1.61) / FAD-dependent cmnm(5)s(2)U34 oxidoreductase		
1	CDS	815038	816249	+	atgaaacgagtcgtcatcaccg	MKRVVITGMGIISSIGNNVEEV	fig|127906.87.peg.746	VCC0746	VC2109	fabB	3-oxoacyl-[acyl-carrier-protein] synthase, KASI (EC 2.3.1.41)		ORF split into 2 in N16961. Not refseq annotation in PATRIC. Annotated as 1 pseudogene named VC2109 in KEGG.
1	CDS	816453	817598	+	atgaaaattctgattgatgagaa	MKILIDENMPYAQALFSQLGEV	fig|127906.87.peg.747	VCC0747	VC2108	pdxB	Erythronate-4-phosphate dehydrogenase (EC 1.1.1.290)		
1	CDS	817582	818595	+	atgagccaacaatttaatgttgcc	MSQQFNVAIFGATGAVGETMI	fig|127906.87.peg.748	VCC0748	VC2107	asd2	Aspartate-semialdehyde dehydrogenase (EC 1.2.1.11)		
1	CDS	819129	818677	-	atgtcagacaataaccaagcgc	MSDNNQALKDAGLKVTLPRLK	fig|127906.87.peg.749	VCC0749	VC2106	fur	Ferric uptake regulation protein FUR		
1	CDS	819495	819995	+	atgaataaaacactctctaagct	MNKTLSKLYQPNIVKWALNSW	fig|127906.87.peg.750	VCC0750	VC2105		hypothetical protein	DUF4442 domain-containing protein [Vibrio cholerae]	ORF split into 2 in N16961 with refseq annotation for both ORFs in PATRIC. Annotated as 1 pseudogene named VC2105 in KEGG
									VC2104				ORF split into 2 in N16961 with refseq annotation for both ORFs in PATRIC. Annotated as 1 pseudogene named VC2105 in KEGG
1	CDS	820877	819999	-	atggatatccgcgccctacgcta	MDIRALRYFVELVNQQSFTKA	fig|127906.87.peg.751	VCC0751	VC2103	CidR	Transcriptional regulator CidR, LysR family		
1	CDS	820993	821433	+	atgcaaaacgttacgcgtactttt	MQNVTRTFQFKHSLLIVVQVA	fig|127906.87.peg.752	VCC0752	VC2101	CidA	Holin-like protein CidA		
1	CDS	821439	822128	+	atgtcgacttcattactcagcctttt	MSTSLLSLFCFLLTLLFYYASK	fig|127906.87.peg.753	VCC0753	VC2100		CidA-associated membrane protein CidB		
1	CDS	822729	822202	-	atggcaagtgtaggtatcttcttg	MASVGIFFGSDTGNTEAIAKM	fig|127906.87.peg.754	VCC0754	VC2099	fldA	Flavodoxin 1		
1	CDS	822999	822778	-	atgctttacgactacatgaacatc	MLYDYMNMLESVGLDLLFASI	fig|127906.87.peg.755	VCC0755	VC2098		Uncharacterized protein VC2098	DUF2788 domain-containing protein [Vibrio cholerae]	
1	CDS	823868	823095	-	atgtcagcacttcttaactacaaa	MSALLNYKLEGSGETVVLIHGI	fig|127906.87.peg.756	VCC0756	VC2097	ybfF	Esterase ybfF (EC 3.1.-.-)		
1	CDS	823961	824494	+	atgaaaacaattgaggttgatga	MKTIEVDEDLYRYIASQTLHIG	fig|127906.87.peg.757	VCC0757	VC2096	seqA	SeqA protein, negative modulator of initiation of replication		
1	CDS	824603	826249	+	atggctatgcaccctcgtgctgg	MAMHPRAGQKARQEDLHNIP	fig|127906.87.peg.758	VCC0758	VC2095	pgm	Phosphoglucomutase (EC 5.4.2.2)		
1	CDS	827072	826299	-	atgtcaaaaagtggtggtgatca	MSKSGGDQMELKHLMDWVIR	fig|127906.87.peg.759	VCC0759	VC2094		hypothetical protein	DUF1853 family protein [Vibrio cholerae]	
1	CDS	827137	827895	+	atgaacaatttacagctagaagc	MNNLQLEALLNETLSPQLIKDY	fig|127906.87.peg.760	VCC0760	VC2093		GTP cyclohydrolase 1 type 2 homolog YbgI		
1	CDS	829236	827947	-	atggcggataagaaagcgacc	MADKKATLHIEGKAPIELPIMD	fig|127906.87.peg.761	VCC0761	VC2092	gltA	Citrate synthase (si) (EC 2.3.3.1)		
1	CDS	829632	830024	+	gtgagcaagcccgtgaaagaa	MSKPVKERKSRPVNLDLQTIS	fig|127906.87.peg.762	VCC0762	VC2091	sdhC	Succinate dehydrogenase cytochrome b-556 subunit		Limited query cover for selected strains
1	CDS	830018	830362	+	atggtaaagcatgtctcttccttg	MVKHVSSFGRNGVHDYLLIRA	fig|127906.87.peg.763	VCC0763	VC2090	sdhD	Succinate dehydrogenase hydrophobic membrane anchor protein		
1	CDS	830363	832129	+	gtgactattccagttcgttcttttga	MTIPVRSFDAVVIGAGGAGMR	fig|127906.87.peg.764	VCC0764	VC2089	sdhA	Succinate dehydrogenase flavoprotein subunit (EC 1.3.5.1)		
1	CDS	832144	832854	+	atgaaactcaacttctccttatatc	MKLNFSLYRYNPDVDNKPYMI	fig|127906.87.peg.765	VCC0765	VC2088	sdhB	Succinate dehydrogenase iron-sulfur protein (EC 1.3.5.1)		
1	CDS	832943	835753	+	atgcacaacggcgtgatgaagg	MHNGVMKAWLESSHLAGANA	fig|127906.87.peg.766	VCC0766	VC2087	sucA	2-oxoglutarate dehydrogenase E1 component (EC 1.2.4.2)		
1	CDS	835782	836996	+	atgaccattgaaattctggttcca	MTIEILVPDLPESVADATVATW	fig|127906.87.peg.767	VCC0767	VC2086	sucB	Dihydrolipoamide succinyltransferase component (E2) of 2-oxoglutarate dehydrogenase complex (EC 2.3.1.61)		
1	CDS	837164	838330	+	atgaatttgcatgaatatcaagct	MNLHEYQAKQLFAEFGLPVPE	fig|127906.87.peg.768	VCC0768	VC2085	sucC	Succinyl-CoA ligase [ADP-forming] beta chain (EC 6.2.1.5)		
1	CDS	838330	839202	+	atgtcgattcttatcaataaagac	MSILINKDTKVICQGFTGGQGT	fig|127906.87.peg.769	VCC0769	VC2084	sucD	Succinyl-CoA ligase [ADP-forming] alpha chain (EC 6.2.1.5)		
1	CDS	840127	839309	-	gtgtttgctcacatcattcacatgg	MFAHIIHMVTTNMIELLLPSLLA	fig|127906.87.peg.770	VCC0770	VC2083	znuB	Zinc ABC transporter, permease protein ZnuB		
1	CDS	840872	840084	-	atggctattctgattgaattgcaa	MAILIELQDICVDFEQRRVLDN	fig|127906.87.peg.771	VCC0771	VC2082	znuC	Zinc ABC transporter, ATP-binding protein ZnuC		
1	CDS	840956	841840	+	atgttatcgagatattttttacttact	MLSRYFLLTVAALFAQSASAIE	fig|127906.87.peg.772	VCC0772	VC2081	znuA	Zinc ABC transporter, substrate-binding protein ZnuA		
1	CDS	842043	845387	+	gtgtttcgcacattgttgtatggct	MFRTLLYGCLLNILIPSLVLALD	fig|127906.87.peg.773	VCC0773	VC2080	cdhR	DNA-binding response regulator, AraC family		
1	CDS	846159	846389	+	atgaaattgtcacaaatgcaag	MKLSQMQAGERGIILALDGLS	fig|127906.87.peg.774	VCC0774	VC2078	feoA	Ferrous iron transporter-associated protein FeoA		
1	CDS	846386	848662	+	atgaagtatcaagtactcaccgt	MKYQVLTVGNPNSGKTTLFN	fig|127906.87.peg.775	VCC0775	VC2077	feoB	Ferrous iron transporter FeoB		
1	CDS	848659	848889	+	gtgattttaaatgaactcaaagc	MILNELKAAIESKNGATRQELA	fig|127906.87.peg.776	VCC0776	VC2076	feoC	Ferrous iron transport protein C		
1	CDS	849579	849151	-	atggcgcatttgaactgcatccc	MAHFELHPQLQKDTDVIGHFP	fig|127906.87.peg.777	VCC0777	VC2075		Histidine triad family protein		
1	CDS	851463	849685	-	atgatggcattcctcccctatttcc	MMAFLPYFLELKGKRVNIQALI	fig|127906.87.peg.778	VCC0778	VC2074	argS	Arginyl-tRNA synthetase (EC 6.1.1.19)		
1	CDS	851618	852214	+	atgcaacaacaactttatgcgac	MQQQLYATGLAPQQLLEKLDV	fig|127906.87.peg.779	VCC0779	VC2073		Protein YecM		
1	CDS	854982	852211	-	gtgcatatcagccccaatgatac	MHISPNDTHQYRYITLSNGLRT	fig|127906.87.peg.780	VCC0780	VC2072	ptra_2	Protease III precursor (EC 3.4.24.55)		
1	CDS	855180	855647	+	atgaagatttatattatgcgtcacc	MKIYIMRHGEAQQFAPSDAQR	fig|127906.87.peg.781	VCC0781	VC2070	sixA	Phosphohistidine phosphatase SixA		
1	CDS	855991	858072	+	atgccatttgcggacaaattgcc	MPFADKLPSIPQRSMPAIGAP	fig|127906.87.peg.782	VCC0782	VC2069	flhA	Flagellar biosynthesis protein FlhA		
1	CDS	858107	859594	+	ttgaaaataaaacgattttttgcca	MKIKRFFAKDMKTALLQVKEE	fig|127906.87.peg.783	VCC0783	VC2068	flhF	Flagellar biosynthesis protein FlhF		
1	CDS	859608	860495	+	atgacaaacaaaatgatatatga	MTNKMIYDQASGLRRLTQPSV	fig|127906.87.peg.784	VCC0784	VC2067	FleN	Flagellar synthesis regulator FleN		
1	CDS	860488	861222	+	gtgaataaagcgcttacatacga	MNKALTYDQYGNLNSQQLFLE	fig|127906.87.peg.785	VCC0785	VC2066	fliA	RNA polymerase sigma factor for flagellar operon		
1	CDS	861256	861636	+	ttgaataaaaacatgaagatcct	MNKNMKILIVDDFSTMRRIVKN	fig|127906.87.peg.786	VCC0786	VC2065		Chemotaxis regulator - transmits chemoreceptor signals to flagellar motor components CheY		
1	CDS	861692	862411	+	atgatttcattagaacaggctaa	MISLEQAKELVQLLEQGRQDD	fig|127906.87.peg.787	VCC0787	VC2064	cheZ	Chemotaxis response - phosphatase CheZ		
1	CDS	862421	864778	+	atgagctacgaattagacgaag	MSYELDEDILQDFLVEAGEILE	fig|127906.87.peg.788	VCC0788	VC2063		Signal transduction histidine kinase CheA		
1	CDS	864805	865938	+	atggcgattaaagtattagttgttg	MAIKVLVVDDSSFFRRRVSEII	fig|127906.87.peg.789	VCC0789	VC2062		Chemotaxis response regulator protein-glutamate methylesterase CheB (EC 3.1.1.61)		
1	CDS	865948	866724	+	atgatcgtttggagtgtagcaaa	MIVWSVANQKGGVGKTTTTIT	fig|127906.87.peg.790	VCC0790	VC2061		ParA-like protein		
1	CDS	866717	867733	+	atgagtagtgcattgatatccagc	MSSALISSEQALNDYFTALLDE	fig|127906.87.peg.791	VCC0791	VC2060		CheW domain protein		
1	CDS	867772	868266	+	atgtctcaggcgaatgaagtgaa	MSQANEVKVRKEKSNDEVLQ	fig|127906.87.peg.792	VCC0792	VC2059		Positive regulator of CheA protein activity (CheW)		
1	CDS	868266	868745	+	atggttgatttgatttggttaaccc	MVDLIWLTPPVIAGGSVFVVLC	fig|127906.87.peg.793	VCC0793	VC2058		ATPase involved in DNA repair		
1	CDS	869119	869742	+	atgttagaagttaaaaatctcacc	MLEVKNLTAIRDERILFESLSFE	fig|127906.87.peg.794	VCC0794	VC2057	ccmA	ABC transporter involved in cytochrome c biogenesis, ATPase component CcmA		
1	CDS	869739	870407	+	atgatgcctgcttttattcagtttatt	MMPAFIQFIHRELLIAFRRQAD	fig|127906.87.peg.795	VCC0795	VC2056	ccmB	ABC transporter involved in cytochrome c biogenesis, CcmB subunit		
1	CDS	870498	871250	+	atgtggaaatggcttcatccctat	MWKWLHPYAKPDAAYQLCGH	fig|127906.87.peg.796	VCC0796	VC2055	ccmC	Cytochrome c-type biogenesis protein CcmC, putative heme lyase for CcmE		
1	CDS	871250	871456	+	atgcattttgactctttcagtgatttt	MHFDSFSDFLAMGGYAAYVW	fig|127906.87.peg.797	VCC0797	VC2054	ccmD	Cytochrome c-type biogenesis protein CcmD, interacts with CcmCE		
1	CDS	871453	871935	+	atgaacccaagacgtaaaaag	MNPRRKKRLGVVLAILFGLSA	fig|127906.87.peg.798	VCC0798	VC2053	ccmE	Cytochrome c-type biogenesis protein CcmE, heme chaperone		
1	CDS	871932	873887	+	atgattgctgaaattggccactttg	MIAEIGHFALIVSLSLAILLSVLP	fig|127906.87.peg.799	VCC0799	VC2052	ccmF	Cytochrome c heme lyase subunit CcmF		
1	CDS	873888	874442	+	gtgaataagaagattttatttatcc	MNKKILFIPLVAFLLLAGVFATC	fig|127906.87.peg.800	VCC0800	VC2051	dsbE	Cytochrome c-type biogenesis protein CcmG/DsbE, thiol:disulfide oxidoreductase		
1	CDS	874439	874915	+	atgaaaaagtggttgtttgcggtc	MKKWLFAVCAAMLISLSAFAT	fig|127906.87.peg.801	VCC0801	VC2050	ccmL	Cytochrome c heme lyase subunit CcmL		Only annotated with refseq annotation in KEGG
1	CDS	874918	876132	+	atgtggatgttttggatctcgacc	MWMFWISTLLLVAIAVVFVIIPF	fig|127906.87.peg.802	VCC0802	VC2049	ccmH	Cytochrome c heme lyase subunit CcmH		Only annotated with refseq annotation in KEGG
1	CDS	876203	876949	+	atggttggccgcttatcttggatta	MVGRLSWITLFTLFLVGCSSAI	fig|127906.87.peg.803	VCC0803	VC2048	mlaA/vacJ	Outer-membrane-phospholipid-binding lipoprotein MlaA		Limited query cover for selected strains
1	CDS	877633	876962	-	atggacataaaaagtgcaataa	MDIKSAIILITSASSALGSTLATI	fig|127906.87.peg.804	VCC0804	VC2047		Oxidoreductase, short-chain dehydrogenase/reductase family		Limited query cover for selected strains
1	CDS	878514	878011	-	atgcaagtccatacattagataa	MQVHTLDKAGVISELHFGQGI	fig|127906.87.peg.805	VCC0805	VC2046		Potential queD like 2		
1	CDS	879213	878629	-	atggcatttgaattacccgctcttc	MAFELPALPYAKDALEPHISAE	fig|127906.87.peg.806	VCC0806	VC2045	sodB	Superoxide dismutase [Fe] (EC 1.15.1.1)		
1	CDS	879465	879797	+	atggaaactattgacaaaatcaa	METIDKIKQQIAENPILLYMKGS	fig|127906.87.peg.807	VCC0807	VC2044	grxD	Monothiol glutaredoxin GrxD		
1	CDS	879882	881825	+	atgacccggttatttattgcagaa	MTRLFIAEKPSLARAIADALPK	fig|127906.87.peg.808	VCC0808	VC2043	topB	DNA topoisomerase III (EC 5.99.1.2)		
1	CDS	881846	882766	+	atgatcccactgatttaccatcct	MIPLIYHPIYSQLDLPVGHRYP	fig|127906.87.peg.809	VCC0809	VC2042	acuC	Histone deacetylase/AcuC/AphA family protein		
1	repeat	882794	882920	+	aaaaagccgctgaattatcagcggcttttctaatgtggcggagagataggga		fig|127906.87.repeat.18				repeat region		
1	rna	882919	882829	-	ggagagatggctgagtggttgaaagcaccggtcttgaaaaccggcgatggt		fig|127906.87.rna.43	VCCt030	VCt065		tRNA-Ser-TGA		
1	rna	883160	883084	-	cggactgtagcgcagcttggtagcgcaccgtcatggggtgtcgggggtcgg		fig|127906.87.rna.44	VCCt031	VCt064		tRNA-Pro-GGG		
1	CDS	885026	883224	-	atggtagatagcggaaatacat	MVDSGNTYGDRVSRLVGWGI	fig|127906.87.peg.810	VCC0810	VC2041	YejM	Inner membrane protein YejM, alkaline phosphatase superfamily		
1	CDS	885276	885061	-	atgcctattacatctaaatacagt	MPITSKYSDEHVESILTEIAAVL	fig|127906.87.peg.811	VCC0811	VC2040	YejL	UPF0352 protein YejL	DUF1414 family protein [Vibrio cholerae]	
1	CDS	885361	886362	+	atgagcctattcctttctaacgtga	MSLFLSNVILHQLRKNDNDELY	fig|127906.87.peg.812	VCC0812	VC2039	yejK	Nucleoid-associated protein NdpA		
1	CDS	886525	887730	+	atggcacagaccttattgaatca	MAQTLLNQGVRKKVVMAIFAA	fig|127906.87.peg.813	VCC0813	VC2038		hypothetical protein	Hpt domain-containing protein [Vibrio cholerae]	
1	CDS	889210	887765	-	atgttacaagcccaacaacgcc	MLQAQQRLPSLTQVIVSLGLFI	fig|127906.87.peg.814	VCC0814	VC2037	mleN/yqkI	Malate-2H(+)/Na(+)-lactate antiporter YqkI/MleN		
1	CDS	889635	890747	+	atgagagtaggtttagtaggttgg	MRVGLVGWRGMVGSVLMQR	fig|127906.87.peg.815	VCC0815	VC2036	asd	Aspartate-semialdehyde dehydrogenase (EC 1.2.1.11)		
1	CDS	891475	890837	-	atgcaaagctttgaaattgctata	MQSFEIAIFLQFFLGLVAAVNP	fig|127906.87.peg.816	VCC0816	VC2035		UPF0056 inner membrane protein YchE	YchE family NAAT transporter [Vibrio]	
1	CDS	892102	894786	+	atgcctgttactaatctggctgaa	MPVTNLAELDALVARVKAAQA	fig|127906.87.peg.817	VCC0817	VC2033	adhE	Acetaldehyde dehydrogenase (EC 1.2.1.10) / Alcohol dehydrogenase (EC 1.1.1.1)		
1	CDS	895771	894896	-	atgacatggattttctcaccctgt	MTWIFLTLFAAFMQAWRNAFC	fig|127906.87.peg.818	VCC0818	VC2032		Permease of the drug/metabolite transporter (DMT) superfamily		
1	CDS	897448	895889	-	atgtttgaattcccttctttctcgca	MFEFPSFSQHSIKNDVLSGLTY	fig|127906.87.peg.819	VCC0819	VC2031		Sulfate permease		
1	CDS	900960	897802	-	atgaaaagaatgttaattaacgc	MKRMLINATQKEELRVALVDG	fig|127906.87.peg.820	VCC0820	VC2030	rne	Ribonuclease E (EC 3.1.26.12)		
1	CDS	901572	902519	+	atgaacgaaatcagaactcaac	MNEIRTQVQFIDIDEDMAGQR	fig|127906.87.peg.821	VCC0821	VC2028	rluC	LSU rRNA pseudouridine(955/2504/2580) synthase (EC 5.4.99.24)		
1	CDS	903201	902620	-	atgcaaaattaccaactagttttta	MQNYQLVLASTSPFRQQILAK	fig|127906.87.peg.822	VCC0822	VC2027	yceF/maf	Maf-like protein YceF		
1	CDS	903349	903873	+	atgcaaaaggtaaaaataccgc	MQKVKIPRTVDPAKAAQKRLD	fig|127906.87.peg.823	VCC0823	VC2026		FIG01269488: protein, clustered with ribosomal protein L32p	23S rRNA accumulation protein YceD [Vibrio cholerae]	
1	CDS	903908	904078	+	atggccgtacaacaaaaccgta	MAVQQNRKTRSRRGMRRSHI	fig|127906.87.peg.824	VCC0824	VC2025	rpmF	LSU ribosomal protein L32p @ LSU ribosomal protein L32p, zinc-independent		
1	CDS	904088	905113	+	ttgcaaaatttaaccgttgcacttg	MQNLTVALDAMGGDFGPRVT	fig|127906.87.peg.825	VCC0825	VC2024	plsX	Phosphate:acyl-ACP acyltransferase PlsX (EC 2.3.1.n2)		
1	CDS	905119	906069	+	atgtatagcaaaattttaggtact	MYSKILGTGSYLPSQVRTNAD	fig|127906.87.peg.826	VCC0826	VC2023	fabH-1	3-oxoacyl-[acyl-carrier-protein] synthase, KASIII (EC 2.3.1.180)		
1	CDS	906139	907062	+	atgagtaagtttgctatcgtatttc	MSKFAIVFPGQGSQAVGMLA	fig|127906.87.peg.827	VCC0827	VC2022	fabD	Malonyl CoA-acyl carrier protein transacylase (EC 2.3.1.39)		
1	CDS	907078	907824	+	atgagtcaattcatgaatctagac	MSQFMNLEGKVALVTGASRG	fig|127906.87.peg.828	VCC0828	VC2021	fabG	3-oxoacyl-[acyl-carrier protein] reductase (EC 1.1.1.100), FadG		
1	CDS	908060	908296	+	atgagcaacatcgaagaacgc	MSNIEERVKKIIVEQLGVDEAE	fig|127906.87.peg.829	VCC0829	VC2020	acpP	Acyl carrier protein		
1	CDS	908385	909629	+	gtgtccaagcgtcgcgttgttgtc	MSKRRVVVTGMGMLSPVGNT	fig|127906.87.peg.830	VCC0830	VC2019	fabF	3-oxoacyl-[acyl-carrier-protein] synthase, KASII (EC 2.3.1.179)		
1	CDS	909707	910510	+	atgtactgggtcaatggtcaacg	MYWVNGQRRNEVPIHDRSFQ	fig|127906.87.peg.831	VCC0831	VC2018	pabC	Aminodeoxychorismate lyase (EC 4.1.3.38)		
1	CDS	910507	911523	+	gtgattaaaaagcgggtattggt	MIKKRVLVLVALIGIVAGSYFY\	fig|127906.87.peg.832	VCC0832	VC2017		Murein endolytic transglycosylase MltG		
1	CDS	911520	912158	+	atgaacgcaaaatttatcgtaatt	MNAKFIVIEGLEGAGKSTAIQV	fig|127906.87.peg.833	VCC0833	VC2016	tmk	Thymidylate kinase (EC 2.7.4.9)		
1	CDS	912151	913125	+	gtgtctaatctcaattctctttacc	MSNLNSLYPWLVPVWQPWQ/	fig|127906.87.peg.834	VCC0834	VC2015	holB	DNA polymerase III delta prime subunit (EC 2.7.7.7)		
1	CDS	913116	913883	+	atgtttgtagattctcattgtcatct	MFVDSHCHLDKLDYQDLHAD	fig|127906.87.peg.835	VCC0835	VC2014		Uncharacterized metal-dependent hydrolase YcfH	YchF/TatD family DNA exonuclease [Vibrio cholerae]	
1	CDS	914327	915757	+	atgtttaagaacgcttttgcgaatc	MFKNAFANLQKVGKALMLPVS	fig|127906.87.peg.836	VCC0836	VC2013	ptsG	PTS system, glucose-specific IIC component / PTS system, glucose-specific IIB component (EC 2.7.1.199)		
1	CDS	915970	915869	-	atgacattcagtgcaattatcatg	MTFSAIIMMIIGLGITWGGAAIC	fig|127906.87.peg.837	VCC0837	Not annotated as CDS		hypothetical protein	Select seq ref|WP_119470446.1| MetS family NSS transporter small subunit [Vibrio cholerae]	Limited query/subject cover for certain strains
1	CDS	917430	915970	-	atgaagcgagaacaatgggga	MKREQWGSRVGFVLAAVGSA	fig|127906.87.peg.838	VCC0838	VC2012		Sodium-dependent transporter, SNF family		
1	CDS	918527	917937	-	atgcgtcaaataaatctatttattc	MRQINLFILNTLFLLFAPLSAAE	fig|127906.87.peg.839	VCC0839	VC2011		hypothetical protein	YnhF family membrane protein [Vibrio cholerae]	
1	CDS	918627	918752	+	atgttattacttgttgtataaccaa	MLLLCCITKEVSMEQDLKFALA	fig|127906.87.peg.840	VCC0840	VC2010		hypothetical protein	YnhF family membrane protein [Vibrio cholerae]	
1	CDS	918771	919928	+	atgaagagtggtggagttgtttct	MKSGGVVSDARTELDTQRLA	fig|127906.87.peg.841	VCC0841	VC2009		Serine protease		
1	CDS	921415	919970	-	atgagctcaactttgcgtagaac	MSSTLRRTKIVATLGPSTESPE	fig|127906.87.peg.842	VCC0842	VC2008	pykA-1	Pyruvate kinase (EC 2.7.1.40)		
1	CDS	921860	923077	+	atgtacatggctcaacccggcca	MYMAQPGHIDHIKQINAGRVY	fig|127906.87.peg.843	VCC0843	VC2007	mlc	Mlc, transcriptional repressor of MalT (the transcriptional activator of maltose regulon) and manXYZ operon		
1	CDS	923182	924126	+	atgtcaggtgttttgaacacggta	MSGVLNTVDQRTNLVGENRLI	fig|127906.87.peg.844	VCC0844	VC2006		Chemotaxis protein CheV (EC 2.7.3.-)		
1	CDS	924556	924200	-	atgaacccaataggcgcagga	MNPIGAGSVHTLPSNTTLKSQ	fig|127906.87.peg.845	VCC0845	VC2005		hypothetical protein		
1	CDS	925616	924696	-	atgcgcactattgcggcactttc	MRTIAALFLSFSLLGCSATPTC	fig|127906.87.peg.846	VCC0846	VC2004		Uncharacterized iron-regulated protein	ChaN family lipoprotein [Vibrio cholerae]	
1	CDS	926512	925694	-	atgaccttttgtgcccgctgtgtg	MTFLCPLCEHPLTLNQNTYAC	fig|127906.87.peg.847	VCC0847	VC2003	rrmA	23S rRNA (guanine(745)-N(1))-methyltransferase (EC 2.1.1.187)		
1	CDS	927671	926667	-	atgaacattatttaccccagtcta	MNIIYPSLLTLLVASLPPAAYA	fig|127906.87.peg.848	VCC0848	VC2002		hypothetical protein	DUF2860 domain-containing protein [Vibrio cholerae]	
1	repeat	927954	928082	+	atataccctcctacttgaagctgcagcggtgttggctgcattcgttcaccccaa		fig|127906.87.repeat.19				repeat region		
1	CDS	928996	928106	-	atggatttactctctttgaaaaccg	MDLLSLKTVTTLSDCVTIAEQD	fig|127906.87.peg.849	VCC0849	VC2001	yeaD	Aldose 1-epimerase family protein YeaD		
1	CDS	930139	929144	-	atgactatcaaagtaggtattaa	MTIKVGINGFGRIGRFVFRAAC	fig|127906.87.peg.850	VCC0850	VC2000	gapA	NAD-dependent glyceraldehyde-3-phosphate dehydrogenase (EC 1.2.1.12)		
1	CDS	930478	930882	+	gtgaaattcgaatcaaaagatct	MKFESKDLHKPDEYWREHLTI	fig|127906.87.peg.851	VCC0851	VC1998		Peptide-methionine (R)-S-oxide reductase MsrB (EC 1.8.4.12)		
1	CDS	931867	931043	-	atgaatttgactaaaccattactta	MNLTKPLLILASLVLLSGCFEN	fig|127906.87.peg.852	VCC0852	VC1997		hypothetical protein	DUF2989 domain-containing protein [Vibrio cholerae]	
1	CDS	931971	932258	+	atggacacccaacaactgatta	MDTQQLINAITPEAYQRLLYAV	fig|127906.87.peg.853	VCC0853	VC1996		hypothetical protein	YeaC family protein [Vibrio]	
1	CDS	933369	932356	-	atggcaagaaaacatatttatatc	MARKHIYIAYTGGTIGMKKSD	fig|127906.87.peg.854	VCC0854	VC1995	ansA	L-asparaginase I, cytoplasmic (EC 3.5.1.1)		
1	CDS	935336	933486	-	atgaaatcactatttcgttttgttgg	MKSLFRFVGLILKGIWKAITFIR	fig|127906.87.peg.855	VCC0855	VC1994	sppA	Signal peptide peptidase SppA (protease 4)		
1	CDS	935566	937566	+	atgtacccaaatctatttcagcct	MYPNLFQPLDLGFTQLKNRVL	fig|127906.87.peg.856	VCC0856	VC1993	fadH	2,4-dienoyl-CoA reductase [NADPH] (EC 1.3.1.34)		
1	CDS	938473	937640	-	atggaaaaaaaaacccctgctga	MEKKTLLTHCPDAPGLISKITN	fig|127906.87.peg.857	VCC0857	VC1992	purU	Formyltetrahydrofolate deformylase (EC 3.5.1.10)		
1	CDS	938743	938946	+	atggataaagaacttctcgcacc	MDKELLARKLYVERVEALLGD	fig|127906.87.peg.858	VCC0858	VC1991		hypothetical protein		
1	CDS	938966	940978	+	atgagattgtcattcgcaccgctg	MRLSFAPLLKTCHNRAISVAQ	fig|127906.87.peg.859	VCC0859	VC1990	dinG	DinG family ATP-dependent helicase YoaA		
1	CDS	940975	941688	+	atgagcgtaaaaattctcgcctta	MSVKILALDTATERCSVALLVC	fig|127906.87.peg.860	VCC0860	VC1989		tRNA threonylcarbamoyladenosine biosynthesis protein TsaB		
1	CDS	941748	942053	+	atggtttcaattaatggattacctc	MVSINGLPPARLPGTNKTSKT	fig|127906.87.peg.861	VCC0861	VC1988		Chromosome segregation ATPase		
1	CDS	942070	942627	+	atgaagatgcatgtgctgaaatt	MKMHVLKLLIPTMALLLSACAS	fig|127906.87.peg.862	VCC0862	VC1987	slp	Starvation lipoprotein Slp paralog		
1	CDS	942624	943478	+	atgaactctatttcaaccacttatc	MNSISTTYLLEQGQLAAIEVGN	fig|127906.87.peg.863	VCC0863	VC1986		Predicted hydrolase/acyltransferase		
1	CDS	943603	945303	+	gtggataaaccttggctttcacgt	MDKPWLSRYPKDVPETINPDC	fig|127906.87.peg.864	VCC0864	VC1985	fadD	Long-chain-fatty-acid--CoA ligase (EC 6.2.1.3)		
1	CDS	945413	946534	+	gtgaattatcaaattattagccaa	MNYQIISQVGDLQRVCLAARD	fig|127906.87.peg.865	VCC0865	VC1984	rnd	Ribonuclease D (EC 3.1.26.3)		
1	CDS	946747	948594	+	atgtctgcgacacattacctcaat	MSATHYLNQLNHRYLNIHRVK	fig|127906.87.peg.866	VCC0866	VC1983		Oligoendopeptidase F-like protein		
1	CDS	949221	948670	-	gtgaatctttacctgctgttattggc	MNLYLLLLAISLLSLSLQWPPL	fig|127906.87.peg.867	VCC0867	VC1981		Membrane protein	Rhombosortase [Vibrio cholerae]	
1	CDS	949225	949827	+	atgtcccgttattgttccgagtgtg	MSRYCSECGKARKACLCPSIV	fig|127906.87.peg.868	VCC0868	VC1980		Uncharacterized conserved protein YfiP, contains DTW domain	DTW domain-containing protein [Vibrio cholerae]	
1	CDS	951146	949821	-	atgcaagtatccctaaaccctga	MQVSLNPEWLARNNDEHKIRI	fig|127906.87.peg.869	VCC0869	VC1979	dgt	Deoxyguanosinetriphosphate triphosphohydrolase (EC 3.1.5.1)		
1	CDS	951787	951203	-	atgcaagagagccatttttcgcc	MQESHFFAHLARMKLIQRWPI	fig|127906.87.peg.870	VCC0870	VC1978	yfbR	5'-deoxynucleotidase YfbR (EC 3.1.3.89)		
1	CDS	953154	951940	-	atgcaaaatatcgggatgtcgtc	MQNIGMSSKLDNVCYDIRGPV	fig|127906.87.peg.871	VCC0871	VC1977	alaA	Alanine transaminase (EC 2.6.1.2)		
1	CDS	953369	954676	+	ttgttgcatttttatcaggccgttgc	MLHFYQAVAQLMEHIRGAKAH	fig|127906.87.peg.872	VCC0872	VC1976	menF	Isochorismate synthase (EC 5.4.4.2) @ Menaquinone-specific isochorismate synthase (EC 5.4.4.2)		
1	CDS	954673	956385	+	atgaaccgcgatcaagctttattg	MNRDQALLNRLWSRVLLEELS	fig|127906.87.peg.873	VCC0873	VC1975	menD	2-succinyl-5-enolpyruvyl-6-hydroxy-3-cyclohexene-1-carboxylic-acid synthase (EC 2.2.1.9)		
1	CDS	956375	957166	+	atgctctctaacccaactgcacttt	MLSNQLHFAKPTARTPLVVLV	fig|127906.87.peg.874	VCC0874	VC1974	menH	2-succinyl-6-hydroxy-2,4-cyclohexadiene-1-carboxylate synthase (EC 4.2.99.20)		
1	CDS	957191	958057	+	atggctaaaactacaggcatttc	MAKTTGISEQELYAPVIWQDC	fig|127906.87.peg.875	VCC0875	VC1973	menB	Naphthoate synthase (EC 4.1.3.36)		
1	CDS	958145	959143	+	atgcgtcatgcaaccctctatcgc	MRHATLYRYQLPMDSGVILRN	fig|127906.87.peg.876	VCC0876	VC1972	menC	O-succinylbenzoate synthase (EC 4.2.1.113)		
1	CDS	959119	960549	+	atggcgctcggaatcttccctgtg	MALGIFPVSLWQPWLERDPK	fig|127906.87.peg.877	VCC0877	VC1971	menE	O-succinylbenzoic acid--CoA ligase (EC 6.2.1.26)		
1	CDS	961738	960578	-	atgaaaggattcttcaatcttagtc	MKGFFNLSHLSAGFTTVLVGY	fig|127906.87.peg.878	VCC0878	VC1970	ydcO/benE	Benzoate transport protein		
1	CDS	961829	962374	+	atgactgacgtgatgtttaagtca	MTDVMFKSQIANQLKNLRKSR	fig|127906.87.peg.879	VCC0879	VC1968		Transcriptional regulator yidN, Cro/CI family		
1	CDS	964358	962427	-	atgtactcaagctcctcggggttc	MYSSSSGLSYIVGPAWDTADC	fig|127906.87.peg.880	VCC0880	VC1967		Methyl-accepting chemotaxis sensor/transducer protein		
1	CDS	964357	964548	+	atggtttttattgtccaaatgccta	MVFIVQMPKAVTKIPTTLNVTN	fig|127906.87.peg.881	VCC0881	No refSeq annotation		FIG01200472: hypothetical protein		
1	CDS	966528	964657	-	atggcaaacccaactacggctc	MANPTTALLTERRFLPYFITQF	fig|127906.87.peg.882	VCC0882	VC1966	lplT	Lysophospholipid transporter LplT / 2-acylglycerophosphoethanolamine acyltransferase (EC 2.3.1.40)		
1	CDS	967131	966541	-	atggctagacgaaacgatcata	MARRNDHTREELVALTLDRVK	fig|127906.87.peg.883	VCC0883	VC1965		Transcriptional regulator, AcrR family		
1	CDS	967306	967166	-	atgttatggggctttgcagccgat	MLWGFAADLSSQSHYSQFISC	fig|127906.87.peg.884	VCC0884	Not annotated as CDS		hypothetical protein		
1	CDS	967325	968200	+	atgaaacgtttgttttcgcttatcgc	MKRLFSLIALIMVSVAVTPIAEA	fig|127906.87.peg.885	VCC0885	VC1964		hypothetical protein	Tim44 domain-containing protein [Vibrio]	
1	CDS	968355	968678	+	gtggcgcaattcaaacatcaac	MAQFKHQQLSQAEQDKLDAA	fig|127906.87.peg.886	VCC0886	VC1963		COGs COG3492	DUF1244 domain-containing protein [Vibrio cholerae]	
1	CDS	969233	968745	-	atgaaaaaaagtatttttgccctg	MKKSIFALSALTLILVGCDNQQ	fig|127906.87.peg.887	VCC0887	VC1962	nlpE	Lipoprotein		
1	CDS	969788	969525	-	atgtcattgctggaatttttccgcc	MSLLEFFRPQKKTSASVAKER	fig|127906.87.peg.888	VCC0888	VC1961	minE	Cell division topological specificity factor MinE		
1	CDS	970605	969793	-	atgtcgcgcattattgtcgtcacg	MSRIIVVTSGKGGVGKTTSSA	fig|127906.87.peg.889	VCC0889	VC1960	minD	Septum site-determining protein MinD		
1	CDS	971288	970626	-	atgtcgaaaaaccctgacctga	MSKNPDLKGSSFTLSVLHLSD	fig|127906.87.peg.890	VCC0890	VC1959	minC	Septum site-determining protein MinC		
1	CDS	971488	971766	+	atgctttgctctatttacaaaagtc	MLCSIYKSPKKEGTYLYIPKRD	fig|127906.87.peg.891	VCC0891	VC1957		Protein YcgL		
1	CDS	971791	972762	+	ttgaaaaaattattatcgatagta	MKKLLSIVLGLALSAPVLANEV	fig|127906.87.peg.892	VCC0892	VC1956	mltB	Membrane-bound lytic murein transglycosylase B		
1	CDS	972926	973873	+	ttgtttgctgaacgcgcgagcca	MFAERASQMALFAVLIQHQSF	fig|127906.87.peg.893	VCC0893	VC1955		Transcriptional regulator, LysR family		
1	CDS	974216	975421	+	atgtcatcactcctcggtatgggc	MSSLLGMGAILLVAWLFSTNR	fig|127906.87.peg.894	VCC0894	VC1953		Na+ dependent nucleoside transporter NupC		
1	CDS	975685	978225	+	atgaagcgctattgtttagccgcc	MKRYCLAAVITASLGVSYSAQ	fig|127906.87.peg.895	VCC0895	VC1952	chiD	Chitinase (EC 3.2.1.14)		
1	CDS	978492	979586	+	atgtcgatcaaaaaaaggtatgt	MSIKKRYVALLVAIGVAIGWVT	fig|127906.87.peg.896	VCC0896	VC1951	torY	Trimethylamine-N-oxide reductase associated c-type cytochrome, TorY		

1	CDS	979609	982056	+	atgactaaaatcacccgccgtg	MTKITRRGFLKGTGMAAGAM	fig|127906.87.peg.897	VCC0897	VC1950	torZ	Trimethylamine-N-oxide reductase (EC 1.7.2.3), TorZ		
1	CDS	983972	982146	-	atgctcgatgctcaacaagaaa	MLDAQQETYVEKISFILLNQLI	fig|127906.87.peg.898	VCC0898	VC1949	pvcA	PvcA protein, related to known isonitrile synthases		
1	CDS	984079	983942	-	atgagccaagatgtcattctcatt	MSQDVILICAQITQQENDRIEY	fig|127906.87.peg.899	VCC0899	VC1948		FIG01200939: hypothetical protein		
1	CDS	984331	985221	+	atgctaaatgagttaaaacgcat	MLNELKRIVIFNKVVECGSFTC	fig|127906.87.peg.900	VCC0900	VC1947		Transcriptional regulator, LysR family		
1	CDS	986815	985352	-	atgtccacaaaaataaccgaca	MSTKITDIVIIGAGPVGLMSAYL	fig|127906.87.peg.901	VCC0901	VC1945	mhpA	FAD monooxygenase, PheA/TfdB family		
1	CDS	987648	986800	-	atgccgtacaaaattaccgctct	MPYKITALQPFGVIVEPKHAPI	fig|127906.87.peg.902	VCC0902	VC1944	pvcB	PvcB protein, related to amino acid oxidizing enzymes		
1	CDS	988806	987946	-	atgactgctcaaaatattgatgg	MTAQNIDGTLISQTVRSEVAA	fig|127906.87.peg.903	VCC0903	VC1942	folD	Methenyltetrahydrofolate cyclohydrolase (EC 3.5.4.9) / Methylenetetrahydrofolate dehydrogenase (NADP+) (EC 1.5.1.5)		
1	rna	989008	989084	+	gcgcccttagctcagttggatagagcacgtgccttctaagcatgtggtcacag		fig|127906.87.rna.45	VCCt032	VCt063		tRNA-Arg-TCT		
1	CDS	989436	990731	+	atgaaacgcatccccgcattgg	MKRIPALDSIRGLLLVLITFNHL	fig|127906.87.peg.904	VCC0904	VC1941		OpgC protein		
1	CDS	991044	991706	+	atgagcaagcccctttatgtgttt	MSKPLYVFDLDDTLIDGDCAM	fig|127906.87.peg.905	VCC0905	VC1940		Phosphoserine phosphatase (EC 3.1.3.3)		
1	CDS	992373	991753	-	atgaatacggcactttgggact	MNTALLGLFIPTFFFVSITPGM	fig|127906.87.peg.906	VCC0906	VC1939		Transporter, LysE family	LysE family translocator [Vibrio cholerae]	
1	CDS	993116	992667	-	atggaatcgcttttaaaagatttc	MESLLKDFPVITKIVVAWGEM	fig|127906.87.peg.907	VCC0907	VC1938		Predicted thioesterase		
1	CDS	993877	993206	-	atggcgcgtattctgaaagtgct	MARILKVLFFLLIVKPLVFIGLG	fig|127906.87.peg.908	VCC0908	VC1937		Acyl-CoA:1-acyl-sn-glycerol-3-phosphate acyltransferase (EC 2.3.1.51)		
1	CDS	994817	993885	-	atgctcaacattcctcaacactc	MLNIPQHSLHLMLVILTILVVG	fig|127906.87.peg.909	VCC0909	VC1936		Phosphatidate cytidylyltransferase (EC 2.7.7.41)		
1	CDS	995578	994820	-	gtgcggattggtggtaccacatc	MRIGGTTSPIASHSFYVATRG	fig|127906.87.peg.910	VCC0910	VC1935		CDP-alcohol phosphatidyltransferase family protein		
1	CDS	997540	995657	-	atggtcttgctgtcaatctgggc	MVLLSIWAMVRFTNTYNMLTK	fig|127906.87.peg.911	VCC0911	VC1934		GGDEF domain protein		
1	CDS	998058	997588	-	atgagaacaatgaaggtcatgt	MRTMKVMLLTLLALISSKLFAC	fig|127906.87.peg.912	VCC0912	VC1933		hypothetical protein	oxidoreductase [Vibrio cholerae]	
1	CDS	998247	998390	+	ttgtcattaccgtgtcataaacata	MSLPCHKHKGEEIDMEHVEM	fig|127906.87.peg.913	VCC0913	VC1932		FIG01202096: hypothetical protein		
1	CDS	998618	1000087	+	atgtcagtgtggaatgctgttcat	MSVWNAVHLSRRFAALPQAF	fig|127906.87.peg.914	VCC0914	VC1931		UPF0061 protein YdiU		
1	CDS	1000319	1001317	+	atgttcaagccgcttaccctaatt	MFKPLTLIAASILAVTSFNAAAN	fig|127906.87.peg.915	VCC0915	VC1929	dctP	TRAP-type C4-dicarboxylate transport system, periplasmic component		
1	CDS	1001385	1002047	+	atggaacagtcatggttttcgaga	MEQSWFSRVGQFTDSIEEFLI	fig|127906.87.peg.916	VCC0916	VC1928		TRAP-type C4-dicarboxylate transport system, small permease component		
1	CDS	1002051	1003412	+	atggccgttgcactgttatttattct	MAVALLFILVIGMMIVGVPIAIS	fig|127906.87.peg.917	VCC0917	VC1927	dctM	TRAP-type C4-dicarboxylate transport system, large permease component		
1	CDS	1004868	1003528	-	atgtgtgatgtatttttgattgatga	MCDVFLIDDESDIRIALAQSFE	fig|127906.87.peg.918	VCC0918	VC1926	dctD	C4-dicarboxylate transport transcriptional regulatory protein		
1	CDS	1006664	1004871	-	atgccatccattcgtcgtctcagc	MPSIRRLSLMLWVMFIVLVSM	fig|127906.87.peg.919	VCC0919	VC1925	dctB	Signal transduction histidine kinase regulating C4-dicarboxylate transport system		
1	CDS	1006654	1006872	+	atggatggcatccttggctcttatc	MDGILGSYQQILSLHHTENAQ	fig|127906.87.peg.920	VCC0920	No refSeq annotation		hypothetical protein	protein TolB [Vibrio cholerae O1 str. EC-0009]	
1	CDS	1007163	1008464	+	atgcaagttactgttgaaacgcta	MQVTVETLEGLQRRLNITVPA	fig|127906.87.peg.921	VCC0921	VC1923	tig	Cell division trigger factor (EC 5.2.1.8)		
1	CDS	1008570	1009196	+	atgagctaccaagaaaaaaat	MSYQEKNAMSPIFDALVPMV	fig|127906.87.peg.922	VCC0922	VC1922	clpP	ATP-dependent Clp protease proteolytic subunit ClpP (EC 3.4.21.92)		
1	CDS	1009276	1010556	+	atgacagacaaaagcaaagga	MTDKSKEGGSSKLLYCSFCGI	fig|127906.87.peg.923	VCC0923	VC1921	clpX	ATP-dependent Clp protease ATP-binding subunit ClpX		
1	CDS	1010679	1013039	+	atgaacttggagcgttccgagcg	MNLERSERIEIPVLPLRDVVVY	fig|127906.87.peg.924	VCC0924	VC1920	lon	ATP-dependent protease La (EC 3.4.21.53) Type I		
1	CDS	1013223	1013495	+	gtgaataagacacaactagtag	MNKTQLVEQIAANADISKASA	fig|127906.87.peg.925	VCC0925	VC1919	hupB	DNA-binding protein HU-beta		
1	CDS	1013648	1015507	+	atgatggatcgattacgcgaagg	MMDRLREGVNSIAVKIILGLIIL	fig|127906.87.peg.926	VCC0926	VC1918	ppiD	Peptidyl-prolyl cis-trans isomerase PpiD (EC 5.2.1.8)		
1	CDS	1015642	1015953	+	atgcaaatcaaaaccaaaatag	MQIKTKIVTLFLSLCLPTLPLLA	fig|127906.87.peg.927	VCC0927	VC1917	comEA	DNA uptake protein		
1	CDS	1016136	1016813	+	atgtcttctcatacgccagtggtta	MSSHTPVVTVDGPSGAGKGT	fig|127906.87.peg.928	VCC0928	VC1916	cmk	Cytidylate kinase (EC 2.7.4.25)		
1	CDS	1016922	1018592	+	atgactgaatcttttgctcaactct	MTESFAQLFEEFLNETQFQQ	fig|127906.87.peg.929	VCC0929	VC1915	rpsA	SSU ribosomal protein S1p		
1	CDS	1018880	1019158	+	atgactaagtccgaactgataga	MTKSELIERLCAEQTHLSAKEI	fig|127906.87.peg.930	VCC0930	VC1914	ihfB	Integration host factor beta subunit		
1	CDS	1019297	1019578	+	atgaaaattataaagatagtcgc	MKIIKIVAVLALFLIALALGSQN	fig|127906.87.peg.931	VCC0931	VC1913		Membrane protein	LapA family protein [Vibrio cholerae]	
1	CDS	1019590	1020759	+	atgttagaaatactgttcctgttatt	MLEILFLLLPIAAAYGWYMGHR	fig|127906.87.peg.932	VCC0932	VC1912		Lipopolysaccharide assembly protein LapB		
1	CDS	1020832	1021527	+	atgaacgacccaaaagtgattg	MNDPKVIVALDYDNLADALAF	fig|127906.87.peg.933	VCC0933	VC1911	pyrF	Orotidine 5'-phosphate decarboxylase (EC 4.1.1.23)		
1	CDS	1022355	1021585	-	atgattcatccatctgctatccaa	MIHPSAIQQLLEPINHFLQCPT	fig|127906.87.peg.934	VCC0934	VC1910	miaE	tRNA-(ms[2]io[6]A)-hydroxylase		
1	CDS	1023187	1022423	-	atgaatataggtttaatcattgctc	MNIGLIIALVAILLILVLGYNIML	fig|127906.87.peg.935	VCC0935	VC1909		ATPase involved in DNA repair		
1	CDS	1023241	1023390	+	gtgatcccgacaatttactctgttc	MIPTIYSVDYSRFIMQKPLSHD	fig|127906.87.peg.936	VCC0936	VC1908		hypothetical protein		
1	CDS	1023476	1024450	+	atgaaactgcagcagttaaagta	MKLQQLKYIVEVVNHNLNVSA	fig|127906.87.peg.937	VCC0937	VC1907	cysB	Cys regulon transcriptional activator CysB		
1	CDS	1024512	1025708	+	atgcaagctctattcatacaactc	MQALFIQLDSFLTQTQSFWRY	fig|127906.87.peg.938	VCC0938	VC1906		SAM-dependent methyltransferase		
1	CDS	1026874	1025750	-	atgatcattggcgttcctaaagaa	MIIGVPKEIKNHEYRVGMIPAS	fig|127906.87.peg.939	VCC0939	VC1905	ald	Alanine dehydrogenase (EC 1.4.1.1)		
1	CDS	1027031	1027525	+	atggtggatagttataagaagcc	MVDSYKKPSKDLDRIDRNILNE	fig|127906.87.peg.940	VCC0940	VC1904	lrp	Leucine-responsive regulatory protein, regulator for leucine (or lrp) regulon and high-affinity branched-chain amino acid transport system		
1	CDS	1027673	1027527	-	atgcgcaacactatacgcagttt	MRNTIRSFMAVKQSERPTPLG	fig|127906.87.peg.941	VCC0941	No refSeq annotation		FIG01200788: hypothetical protein		
1	CDS	1027703	1030585	+	atgttcaaagagaacaaaaata	MFKENKNKVETIIKTSEEAPSS	fig|127906.87.peg.942	VCC0942	VC1903	ftsK	DNA translocase FtsK		
1	CDS	1031182	1030661	-	gtgcgtatattatcctcccttaaaa	MRILSSLKTFSQSRLSWLLLLA	fig|127906.87.peg.943	VCC0943	VC1902	dsbB	Periplasmic thiol:disulfide oxidoreductase DsbB, required for DsbA reoxidation		
1	CDS	1032854	1031262	-	atgccgatgtccctcggaaacg	MPMSLGNAFIKNFLGKAPDWY	fig|127906.87.peg.944	VCC0944	VC1901	nhaB	Na+/H+ antiporter NhaB		
1	CDS	1033499	1034338	+	atggtcattaaggcaaaaagcc	MVIKAKSPAGFAEKYIIESIWN	fig|127906.87.peg.945	VCC0945	VC1900	fadR	Transcriptional regulator for fatty acid degradation FadR, GntR family		
1	CDS	1035724	1034573	-	atggctattcacgtagggattatc	MAIHVGIIDQDPVRLVTPLLDH	fig|127906.87.peg.946	VCC0946	VC1899		hypothetical protein	DUF1887 family protein [Vibrio cholerae]	
1	CDS	1037885	1035894	-	atgcagggttccgttattcggcgt	MQGSVIRRMYAGFALIIIMFVV	fig|127906.87.peg.947	VCC0947	VC1898		Methyl-accepting chemotaxis sensor/transducer protein		
1	CDS	1038145	1038495	+	atggcggaagaaaccattttcag	MAEETIFSKIVRREIPADILYQD	fig|127906.87.peg.948	VCC0948	VC1897		YcfF/hinT protein: a purine nucleoside phosphoramidase		
1	CDS	1038514	1039926	+	atggtgacgaatcacgtggttca	MVTNHVVQYIKRPIRSLTLTSL	fig|127906.87.peg.949	VCC0949	VC1896		hypothetical protein	lipoprotein	
1	CDS	1039926	1040315	+	atgagaacgtggctattagcagt	MRTWLLAVLTALLLVGCSANT	fig|127906.87.peg.950	VCC0950	VC1895		YcfL protein: an outer membrane lipoprotein that is part of a salvage cluster		
1	CDS	1040368	1040958	+	atgaaaaaaagtgtcattgccct	MKKSVIALLGLAVILGGCSNKV	fig|127906.87.peg.951	VCC0951	VC1894	lpoB	Lipoprotein YcfM, part of a salvage pathway of unknown substrate		
1	CDS	1040986	1041846	+	atggcaaagattgcatggcgag	MAKIAWREASALDPSLLSLNH	fig|127906.87.peg.952	VCC0952	VC1893		Thiamine kinase (EC 2.7.1.89) @ Adenosylcobinamide kinase (EC 2.7.1.156)		
1	CDS	1042026	1042565	+	atgattatttacttacacggattcg	MIIYLHGFDSNSPGNHEKVLQI	fig|127906.87.peg.953	VCC0953	VC1892		YcfP protein: probably an esterase that is part of a salvage cluster		
1	CDS	1042798	1042682	-	gtggcacgttactcggcagcaa	MARYSAAKSCANTNNRQVFH	fig|127906.87.peg.954	VCC0954	VC1891		FIG01202113: hypothetical protein		
1	CDS	1042923	1044212	+	gtgacacgaattatcgttgtaggt	MTRIIVVGGGAGGLELVTKLGI	fig|127906.87.peg.955	VCC0955	VC1890	ndh	NADH dehydrogenase (EC 1.6.99.3)		
1	CDS	1044861	1044325	-	atgactccggattttcaaatcgtc	MTPDFQIVTQRLQLRLITADEA	fig|127906.87.peg.956	VCC0956	VC1889	rimL	Putative ribosomal-protein-serine acetyltransferase		
1	CDS	1047221	1045146	-	atgaaacagacaaaaacgttga	MKQTKTLTAISVLALSHLMTQS	fig|127906.87.peg.957	VCC0957	VC1888		Hemolysin-related protein RbmC		
1	CDS	1048320	1047556	-	atgaaaagattgatcccactgct	MKRLIPLLLLLIAMPSMAARQF	fig|127906.87.peg.958	VCC0958	VC1887		hypothetical protein		
1	CDS	1051963	1048496	-	atgactacacactctttactctctc	MTTHSLLSLFSAQGAGDKKAI	fig|127906.87.peg.959	VCC0959	VC1886	mfd	Transcription-repair coupling factor		
1	CDS	1052541	1051966	-	atgaatgaccaagaattctttact	MNDQEFFTVHHALTINVEPLA	fig|127906.87.peg.960	VCC0960	VC1885		hypothetical protein		
1	CDS	1052705	1053925	+	atggtttggatttatgtttcatccgat	MVGFMFHPISAFIGLRYLRGR	fig|127906.87.peg.961	VCC0961	VC1884	lolC	Lipoprotein releasing system transmembrane protein LolC		
1	CDS	1053918	1054604	+	atgaataaccttttacgttgtcatc	MNNLLRCHQVCKTYQEGELQ	fig|127906.87.peg.962	VCC0962	VC1883	lolD	Lipoprotein-releasing system ATP-binding protein LolD		
1	CDS	1054605	1055849	+	gtgttttcctctctcgctctgtttattg	MFSSLALFIGGRFSRAKQRNK	fig|127906.87.peg.963	VCC0963	VC1882	lolE	Lipoprotein releasing system transmembrane protein LolE		
					ttgcaagagagttgcgaacaagcgcagcttaaacaatgcaaactctgtttac		From N16961 in PATRIC	Not annotated as CDS	VC1881				Only annotated with refseq annotation in PATRIC
1	CDS	1056494	1055964	-	atgccaagaaaattcattaaacg	MPRKFIKRFMPDHDVIKRQKA	fig|127906.87.peg.964	VCC0964	VC1880		FIG01199889: hypothetical protein	DUF2062 domain-containing protein [Vibrio cholerae]	
1	CDS	1056503	1058746	+	atgactctcttgtcgaattactggt	MTLLSNYWSLISFSITVLSAPY	fig|127906.87.peg.965	VCC0965	VC1879		DNA internalization-related competence protein ComEC/Rec2		
1	CDS	1058777	1060525	+	atgtcattgcactctgacgaatct	MSLHSDESNWQTFKRLWTYIF	fig|127906.87.peg.966	VCC0966	VC1878	msbA	Lipid A export permease/ATP-binding protein MsbA		
1	CDS	1060525	1061535	+	gtggtgatagaaaagatctggtt	MVIEKIWFHRHPLGYLLWPLLV	fig|127906.87.peg.967	VCC0967	VC1877	lpxK	Tetraacyldisaccharide 4'-kinase (EC 2.7.1.130)		
1	CDS	1061516	1061695	+	atggatcaccgtctgcttgaaatc	MDHRLLEIVACPVCKGKLTYD	fig|127906.87.peg.968	VCC0968	VC1876		FIG002473: Protein YcaR in KDO2-Lipid A biosynthesis cluster		
1	CDS	1061695	1062453	+	atgtcatttactgtcgtgatccctg	MSFTVVIPARYQSTRLPGKPLA	fig|127906.87.peg.969	VCC0969	VC1875	kdsB	3-deoxy-manno-octulosonate cytidylyltransferase (EC 2.7.7.38)		
1	CDS	1064088	1062538	-	atgactaccaaagccaaagcc	MTTKAKAAPKRKVANSKRLPD	fig|127906.87.peg.970	VCC0970	VC1874		FIG004684: SpoVR-like protein	SpoVR family protein [Vibrio cholerae]	
1	CDS	1065372	1064101	-	atggcgcaatttatcgacagaac	MAQFIDRRLNGKNKSTVNRQF	fig|127906.87.peg.971	VCC0971	VC1873		UPF0229 protein YeaH	DUF444 family protein [Vibrio cholerae]	
1	CDS	1067451	1065517	-	atgagtatttttgaacactttcagg	MSIFEHFQARYEAAKDEELSIC	fig|127906.87.peg.972	VCC0972	VC1872		Uncharacterized protein YeaG	PrkA family serine protein kinase [Vibrio cholerae]	
1	CDS	1068192	1067692	-	atggaaatgaccaacgctcagc	MEMTNAQRLILSNQYFLMSQL	fig|127906.87.peg.973	VCC0973	VC1871		hypothetical protein	YfbU family protein [Vibrio cholerae]	
1	CDS	1068975	1068313	-	ttggaactcgcacaaatcgcttc	MELAQIASTIILFIVLFWVRKDT	fig|127906.87.peg.974	VCC0974	VC1870		Membrane protein		
1	CDS	1069781	1069041	-	atgtctaccattggtcgaattcac	MSTIGRIHSFESCGTVDGPGIF	fig|127906.87.peg.975	VCC0975	VC1869	pflA	Pyruvate formate-lyase activating enzyme (EC 1.97.1.4)		
1	CDS	1070114	1071994	+	atggatgtaacgcaactttcaca	MDVTQLSQKQKITLFILVLCLG	fig|127906.87.peg.976	VCC0976	VC1868		Methyl-accepting chemotaxis sensor/transducer protein		
1	CDS	1072998	1072012	-	atgaaatcgattcgctatttacctt	MKSIRYLPLLFISTYAAAAQTS	fig|127906.87.peg.977	VCC0977	VC1867		hypothetical protein	Lipid A deacylase LpxR family protein [Vibrio cholerae]	
1	CDS	1075452	1073176	-	atggcagagcaatttgctaaagc	MAEQFAKAWEGFAAGDWQN	fig|127906.87.peg.978	VCC0978	VC1866	pflB	Pyruvate formate-lyase (EC 2.3.1.54)		
1	CDS	1077259	1075718	-	atgtcagacgttatgaacaaaga	MSDVMNKERSDIETKSYRELH	fig|127906.87.peg.979	VCC0979	VC1865		hypothetical protein	DUF3360 domain-containing protein [Vibrio]	
1	CDS	1077585	1077755	+	atgcacgctttgctaacagtaga	MHALLTVEVRMCTECDFVKNL	fig|127906.87.peg.980	VCC0980	Not annotated as CDS		hypothetical protein		
1	CDS	1077976	1078746	+	atgacggatgtacccgcgcttga	MTDVPALEIKNLHKTFGQNEV	fig|127906.87.peg.981	VCC0981	VC1864	occP	Arginine/ornithine ABC transporter, ATP-binding protein AotP		
1	CDS	1078931	1079707	+	atgaaaaagtggttagttgcgac	MKKWLVATALIATTISGVTQAK	fig|127906.87.peg.982	VCC0982	VC1863	argT	Lysine-arginine-ornithine-binding ABC transporter, substrate-binding protein ArgT (TC 3.A.1.3.1)		
1	CDS	1079855	1080595	+	atgctggatttacaaggctatgaa	MLDLQGYEASILKGALLTIEVA	fig|127906.87.peg.983	VCC0983	VC1862	hisQ	Arginine/ornithine ABC transporter, permease protein AotQ		
1	CDS	1080598	1081275	+	atggacttttctctgattatcgaca	MDFSLIIDSLPIYLSGLWTTVW	fig|127906.87.peg.984	VCC0984	VC1861	hisM	ABC transporter, permease protein (cluster 3, basic aa/glutamine/opines)		
1	CDS	1082140	1081334	-	atgaaagttatcagcttcaacata	MKVISFNINGLRARLHQLQALI	fig|127906.87.peg.985	VCC0985	VC1860	xthA	Exodeoxyribonuclease III (EC 3.1.11.2)		
1	CDS	1082338	1084458	+	atgcgccaaatttctgttcagtgg	MRQISVQWKITLLAGLCLVFTS	fig|127906.87.peg.986	VCC0986	VC1859		Methyl-accepting chemotaxis sensor/transducer protein		
1	CDS	1084694	1084482	-	atgtcgactccatcgattccactg	MSTPSIPLNEASDEIKLAVDLIF	fig|127906.87.peg.987	VCC0987	VC1858		Uncharacterized protein YbaM	DUF2496 domain-containing protein [Vibrio cholerae]	
1	CDS	1085246	1084695	-	atgatggagagctacagcaaat	MMESYSKFHTILNELKHSAAQ	fig|127906.87.peg.988	VCC0988	VC1857	priC	Primosomal replication protein N prime prime		
1	CDS	1086019	1085243	-	atggaatggatagaacccacca	MEWIEPTTIMVLALVAFIAGFID	fig|127906.87.peg.989	VCC0989	VC1856		FIG011065: hypothetical protein	TSUP family transporter [Vibrio cholerae]	
1	CDS	1088135	1086060	-	atgctgacaaaaaacattcaag	MLTKNIQDSIRNSYQNLQNQL	fig|127906.87.peg.990	VCC0990	VC1855	dinG	ATP-dependent helicase DinG/Rad3		
1	CDS	1088645	1089679	+	atgaaaaaaactctattagcact	MKKTLLALAVLAAAGSVNAAE	fig|127906.87.peg.991	VCC0991	VC1854	ompT	Outer membrane porin	Porin OmpT [Vibrio cholerae]	
1	CDS	1089798	1090472	+	atgaaacttaacccactgatttta	MKLNPLILGLLLSFSAGHSLAD	fig|127906.87.peg.992	VCC0992	VC1853		putative secreted protein	DUF2057 domain-containing protein [Vibrio cholerae]	
1	CDS	1090959	1090504	-	atgtcctgttattgcggaaatactc	MSCYCGNTQPYSQCCEPIHLI	fig|127906.87.peg.993	VCC0993	VC1852		UPF0225 protein YchJ		Limited query cover for selected strains
1	CDS	1092235	1091021	-	ttgaagtactcgtatgtcgctaga	MKYSYVARQPILDREKSTIGYE	fig|127906.87.peg.994	VCC0994	VC1851		Predicted signal transduction protein	EAL domain-containing protein [Vibrio cholerae]	
1	CDS	1093071	1092331	-	atgcatacactatttatttccgatc	MHTLFISDLHLSPKHPDITASF	fig|127906.87.peg.995	VCC0995	VC1850	lpxH	UDP-2,3-diacylglucosamine diphosphatase (EC 3.6.1.54)		
1	CDS	1093606	1093112	-	atgatcaccctgcataccaatttt	MITLHTNFGDIKIQLNFEKAPV	fig|127906.87.peg.996	VCC0996	VC1849	ppiB	Peptidyl-prolyl cis-trans isomerase PpiB (EC 5.2.1.8)		
1	CDS	1093752	1095131	+	atgttgaagatttataactcactca	MLKIYNSLTRQKEEFKPIVAGK	fig|127906.87.peg.997	VCC0997	VC1848	cysS	Cysteinyl-tRNA synthetase (EC 6.1.1.16)		
1	CDS	1095219	1095740	+	atgtctgtcatcttagggattgatc	MSVILGIDPGSRVTGYGVIRQ	fig|127906.87.peg.998	VCC0998	VC1847	ruvC	Crossover junction endodeoxyribonuclease RuvC (EC 3.1.22.4)		
1	CDS	1095837	1096451	+	gtgattggacgtcttcgcggtacc	MIGRLRGTLIEKLPPQILIEVGG	fig|127906.87.peg.999	VCC0999	VC1846	ruvA	Holliday junction ATP-dependent DNA helicase RuvA (EC 3.6.4.12)		
1	CDS	1096507	1097511	+	atgatagaagccgatcgtttaatt	MIEADRLIAPISNHFKDEEVIDR	fig|127906.87.peg.1000	VCC1000	VC1845	ruvB	Holliday junction ATP-dependent DNA helicase RuvB (EC 3.6.4.12)		
1	CDS	1097970	1099556	+	atgatcgatgtcgttgatttatcgc	MIDVVDLSRLQFALTAMYHFLF	fig|127906.87.peg.1001	VCC1001	VC1844	cydA	Cytochrome d ubiquinol oxidase subunit I (EC 1.10.3.-)		
1	CDS	1099574	1100710	+	atgtttgattacgagatcttacgac	MFDYEILRLIWWVLIGVLLVGF	fig|127906.87.peg.1002	VCC1002	VC1843	cydB	Cytochrome d ubiquinol oxidase subunit II (EC 1.10.3.-)		
1	CDS	1100723	1100830	+	atgtggtatttcgcatggatccttg	MWYFAWILGVLLACAFGIINAL	fig|127906.87.peg.1003	VCC1003	VC1842		Cytochrome d ubiquinol oxidase subunit X (EC 1.10.3.-)		
1	CDS	1101065	1101178	+	atggcaaatcgtgttcagtccata	MANRVQSISIIDHSELFDVGAA	fig|127906.87.peg.1004	VCC1004	VC1841		hypothetical protein		Limited query and subject cover for certain strains. VC1841 annotated as substantially longer ORF in N16961.
1	CDS	1101299	1101730	+	gtgtatgagatttttatcaaaacca	MYEIFIKTMNTFHWPVTVYYEI	fig|127906.87.peg.1005	VCC1005	VC1840		Tol-Pal system-associated acyl-CoA thioesterase		
1	CDS	1101720	1102403	+	gtgactgctgatatttcatttcttga	MTADISFLDLFLQASFLVKMVM	fig|127906.87.peg.1006	VCC1006	VC1839	tolQ	Tol-Pal system protein TolQ		
1	CDS	1102406	1102846	+	atggctggctatcaaaccaaaa	MAGYQTKKREMKAEINVVPYI	fig|127906.87.peg.1007	VCC1007	VC1838	tolR	Tol biopolymer transport system, TolR protein		
1	CDS	1102864	1103934	+	atgaaagagaataagtccagaa	MKENKSRKSNDAKSITISLAMI	fig|127906.87.peg.1008	VCC1008	VC1837	tolA	TolA protein		
1	CDS	1103949	1105301	+	gtgtttaagcgattagtttttgttttg	MFKRLVFVLMLIGTGFSNIANA	fig|127906.87.peg.1009	VCC1009	VC1836	tolB	Tol-Pal system beta propeller repeat protein TolB		
1	CDS	1105333	1105851	+	atgcaacttaacaaagttcttaag	MQLNKVLKSLLIALPVLAVTAC	fig|127906.87.peg.1010	VCC1010	VC1835	pal	Tol-Pal system peptidoglycan-associated lipoprotein PAL		
1	CDS	1105867	1106631	+	atgttcagtaacttaaagcacgtt	MFSNLKHVVMLMLLASAASYA	fig|127906.87.peg.1011	VCC1011	VC1834	cpoB	Cell division coordinator cpoB		
1	CDS	1106815	1106693	-	atgtggctcattgctcttgctctac	MWLIALALHNFHRRRIVHPSQ	fig|127906.87.peg.1012	VCC1012	Not annotated as CDS		hypothetical protein		
1	CDS	1106805	1107866	+	atgagccacatactcgatactat	MSHILDTINTVYPFPPKPIPLRD	fig|127906.87.peg.1013	VCC1013	VC1833	NadA	Quinolinate synthetase (EC 2.5.1.72)		
1	CDS	1108512	1107940	-	atgcaaaccttacaagaaatcc	MQTLQEILQQPELENKLLNQA	fig|127906.87.peg.1014	VCC1014	VC1832		hypothetical protein	YecA family protein [Vibrio cholerae]	
1	CDS	1108726	1110930	+	atggctattcgctcctcgcttaaa	MAIRSSLKKKSILALTIYLAFFL	fig|127906.87.peg.1015	VCC1015	VC1831	luxQ	Two-component system sensor histidine kinase		
1	CDS	1111266	1111718	+	atgaaagctcgtggtccgttttgta	MKARGPFCIRHAAADTFAMVV	fig|127906.87.peg.1016	VCC1016	VC1828	alaE	L-alanine exporter AlaE		
1	CDS	1113058	1111838	-	atgactcagattccgactcagga	MTQIPTQDAALPEAVFFPMTN	fig|127906.87.peg.1017	VCC1017	VC1827	manA	Mannose-6-phosphate isomerase (EC 5.3.1.8)		
1	CDS	1115021	1113120	-	atgatcaaccaattgattaatgcc	MINQLINADLIQLDLQANSKQA	fig|127906.87.peg.1018	VCC1018	VC1826	manP/fruA	PTS system, mannose-specific IIA component (EC 2.7.1.191) / PTS system, mannose-specific IIB component (EC 2.7.1.191) / PTS system, mannose-specific IIC component		
1	CDS	1115393	1116232	+	atgatatttgatgatttgatttcctc	MIFDDLISSVLNERESFHHIWF	fig|127906.87.peg.1019	VCC1019	VC1825	rhaR	Transcriptional regulator, AraC family		
1	CDS	1117003	1116224	-	atgatgcatgaattccaagtcac	MMHEFQVTFLVNDVNASVHV	fig|127906.87.peg.1020	VCC1020	VC1824	fryA	Putative PTS system, nitrogen regulatory IIA component		
1	CDS	1117311	1117012	-	atgaaaattgttgcggtcaccgc	MKIVAVTACPTGIAHTYMAAD	fig|127906.87.peg.1021	VCC1021	VC1823	frwB	PTS system, IIB component		
1	CDS	1117528	1119393	+	gtggatattacccatctgatcgaa	MDITHLIEPEIICLDLKANNKED	fig|127906.87.peg.1022	VCC1022	VC1822	frwABC	PTS system, fructose-specific IIA component (EC 2.7.1.202) / PTS system, fructose-specific IIC component / PTS system, fructose-specific IIB component (EC 2.7.1.202)		
1	CDS	1120871	1119468	-	atgagtaccctaaccgctcaag	MSTLTAQATNNSSNFKKLLST	fig|127906.87.peg.1023	VCC1023	VC1821	frwBC	PTS system, fructose-specific IIC component / PTS system, fructose-specific IIB component (EC 2.7.1.202)		
1	CDS	1121323	1120883	-	atgatcaccgagctcaccaaca	MITELTNIHLIKGNLQANSKNE	fig|127906.87.peg.1024	VCC1024	VC1820	frvA	Phosphotransferase system mannitol/fructose-specific IIA domain		
1	CDS	1123176	1121656	-	atgatttatgcacaaccgggtag	MIYAQPGSDNAVITFKSHYDN	fig|127906.87.peg.1025	VCC1025	VC1819	aldB/aldA	Aldehyde dehydrogenase (EC 1.2.1.3)		
1	CDS	1123460	1125220	+	atgcaacttcaacagcttagcca	MQLQQLSHVNWLVNSWQRSI	fig|127906.87.peg.1026	VCC1026	VC1817	acoR	sigma-54-dependent transcriptional regulator		
1	CDS	1125317	1125532	+	atgaaccaacaagttaacaacc	MNQQVNNPLHGLSLEKIVTRL	fig|127906.87.peg.1027	VCC1027	VC1816		hypothetical protein	DNA-binding protein VF530 [Vibrio metoecus]	
1	CDS	1126250	1125543	-	atgaatatcgtgatcatcggtggc	MNIVIIGGSGGIGLAMVQEALN	fig|127906.87.peg.1028	VCC1028	VC1815		C-factor, putative		
1	CDS	1127632	1126247	-	ttgagtaaaaaaattggcctgtac	MSKKIGLYWFTNDLRVNDNPL	fig|127906.87.peg.1029	VCC1029	VC1814	cry1	Deoxyribodipyrimidine photolyase, single-strand-specific		
1	CDS	1127773	1128168	+	atgaataagccagatctggtgat	MNKPDLVIFFDGQCPLCCNEM	fig|127906.87.peg.1030	VCC1030	VC1812		Cell division inhibitor SulA		
1	CDS	1128292	1128765	+	atggaaaaaattgccattttggtg	MEKIAILVDVQNVYYTCREQY	fig|127906.87.peg.1031	VCC1031	VC1811		hypothetical protein	NYN domain-containing protein [Vibrio cholerae]	
1	CDS	1128960	1128757	-	atgaaccgtaaaaagaaaatta	MNRKKKINQTLQKRLKKQHAK	fig|127906.87.peg.1032	VCC1032	VC1810		hypothetical protein	DUF2986 domain-containing protein [Vibrio cholerae]	
1	CDS	1129590	1129790	+	atgccagagaacaacattcgttt	MPENNIRLIRFREVLTMTGLSR	fig|127906.87.peg.1033	VCC1033	VC1809		Transcriptional regulator, AlpA like		
1	CDS	1130750	1129905	-	gtgacaaacattaacgaattattt	MTNINELFKYSYLTDWIDSNNT	fig|127906.87.peg.1034	VCC1034	VC1808		hypothetical protein		
1	CDS	1131042	1131281	+	atgaaacgtttaattcaaaatcca	MKRLIQNPNLTLFYDESFLSNF	fig|127906.87.peg.1035	VCC1035	VC1807		FIG01201940: hypothetical protein	Inovirus Gp2 family protein [Vibrio cholerae]	
1	CDS	1131464	1131682	+	atgataaaagcagcatgggcaa	MIKAAWASALGIEYFEVKSSVI	fig|127906.87.peg.1036	VCC1036	VC1807		hypothetical protein	inovirus Gp2 family protein [Vibrio cholerae]	Limited query cover for certain strains
					atgaaacgtttaattcaaaatccaaacctgactcttttctatgacgagagtttttta		From N16961		VC1807				Annotated as 1 pseudogene in KEGG named VC1807. Annotated as 2 ORFs e.g. VCC1035 and VCC1036 in other strains
1	CDS	1132703	1131717	-	atgaaaaccactgatttattctcg	MKTTDLFSTLENNILRNSLDST	fig|127906.87.peg.1037	VCC1037	VC1806		hypothetical protein	GTPase family protein [Vibrio cholerae]	
1	CDS	1133256	1132810	-	atgtctaacattttgttcaaaccca	MSNILFKPTHLPISKPFHALLAI	fig|127906.87.peg.1038	VCC1038	VC1805		hypothetical protein	DUF2787 domain-containing protein [Vibrio cholerae]	
1	CDS	1133483	1133307	-	ttggatgtcgagctctactttcacc	MDVELYFHLANRWCYQPDAG	fig|127906.87.peg.1039	VCC1039	VC1804		hypothetical protein	DUF2787 domain-containing protein [Vibrio cholerae]	
1	CDS	1134309	1133848	-	atgtctatatcagagcgcttaaag	MSISERLKSVIDAKFSTLKGAS	fig|127906.87.peg.1040	VCC1040	VC1803		hypothetical protein	helix-turn-helix transcriptional regulator [Vibrio cholerae]	
1	CDS	1134399	1134635	+	atgcttgaatatgtttcagtcaatc	MLEYVSVNQIDPGKELYNKVK	fig|127906.87.peg.1041	VCC1041	VC1802		hypothetical protein		
1	CDS	1134619	1134960	+	atgaatatggaaagtaagaaag	MNMESKKEKDAFLKISRILSTL	fig|127906.87.peg.1042	VCC1042	VC1801		hypothetical protein	primosomal protein [Vibrio cholerae]	
1	CDS	1134965	1135297	+	atgacaaacgtacgggacaca	MTNVRDTSNTCQMKSTTIALV	fig|127906.87.peg.1043	VCC1043	No refSeq annotation		hypothetical protein	helix-turn-helix domain-containing protein [Vibrio cholerae]	Lmited query cover for certain strains
1	CDS	1135299	1136270	+	atgtccgatgaactgaagcaatt	MSDELKQLMTAQQALSDAQE	fig|127906.87.peg.1044	VCC1044	VC1800		hypothetical protein	MarR family transcriptional regulator [Vibrio cholerae]	
1	CDS	1136277	1138034	+	atgaatgcacttgaattagaacg	MNALELERAQYLKRLMSDLDF	fig|127906.87.peg.1045	VCC1045	VC1799		hypothetical protein	DDE-type integrase/transposase/recombinase [Vibrio]	
1	CDS	1138006	1139115	+	atgaacacgctcagagcgcga	MNTLRARLDKAGTSIQALADY	fig|127906.87.peg.1046	VCC1046	VC1798		Eha protein		Lmited query cover for certain strains
1	CDS	1139118	1139579	+	atgaacaatcagaatgataatg	MNNQNDNGLLPFNVGELVSA	fig|127906.87.peg.1047	VCC1047	VC1797		hypothetical protein		
1	CDS	1139721	1139987	+	atgaacgagcattacgaacaa	MNEHYEQKLKQALRQKSVMP	fig|127906.87.peg.1048	VCC1048	No refSeq annotation		Uncharacterized protein, homolog of phage Mu protein gp30		
1	CDS	1140389	1140763	+	atgttagctatagaaagagcaat	MLAIERAIPLVVDALSKDSGLF	fig|127906.87.peg.1049	VCC1049	VC1796		middle operon regulator-related protein		
1	CDS	1140750	1141070	+	atggcacccactgataatcaaa	MAPTDNQRSLVISKLTPSAQV	fig|127906.87.peg.1050	VCC1050	VC1795		Transcriptional regulator		
1	CDS	1141089	1141667	+	atgtcattaagtaccttacaagca	MSLSTLQAELASAKTEYEAKE	fig|127906.87.peg.1051	VCC1051	VC1794		hypothetical protein	glyoxalase [Vibrio cholerae]	
1	CDS	1141728	1142099	+	atggaacatgtgtatcaattagtc	MEHVYQLVDGIVFKGKLQKQV	fig|127906.87.peg.1052	VCC1052	VC1793		hypothetical protein	6-phospho-beta-glucosidase [Vibrio cholerae]	
1	CDS	1142148	1142507	+	atggctgtaatgacatttaacttag	MAVMTFNLEDGFKVGDAQCH	fig|127906.87.peg.1053	VCC1053	VC1792		Phage tail assembly chaperone Mup41, Y		
1	CDS	1142642	1143682	+	atggctaacaatttaaaaacgga	MANNLKTDIVLNLQGDLAQKA	fig|127906.87.peg.1054	VCC1054	VC1791		Phage tape measure protein Mup42		Lmited query cover for certain strains
1	repeat	1143652	1145694	+	ttgtagtggcatagtgaatttggtcacttagttagaggtgatatcatcacctcata		fig|127906.87.repeat.20				repeat region		
1	CDS	1143725	1144033	+	atgacaaaacgtacaagacga	MTKRTRRLFSAEFKLEAAQLV	fig|127906.87.peg.1055	VCC1055	VC1790		Transposase InsN for insertion sequence element IS911		

1	CDS	1144030	1144902	+	ttgataatcaagaaactcaagca	MIIKKLKQSHSVKILCEVFNVHI	fig|127906.87.peg.1056	VCC1056	VC1789		Transposase InsO for insertion sequence element IS911		
1	CDS	1145028	1145594	+	atgattggagatatggttcttggc	MIGDMVLGGKKGAAGAGSLS	fig|127906.87.peg.1057	VCC1057	VC1788		hypothetical protein	phage tail tape measure family protein [Vibrio cholerae]	
1	CDS	1145827	1145690	-	atgattgtggaaagctatcaaac	MIVESYQTIQLADSFCHALSAI	fig|127906.87.peg.1058	VCC1058	VC1787		FIG01201713: hypothetical protein	DUF2787 domain-containing protein [Vibrio cholerae]	
1	CDS	1146300	1145824	-	atgactcatttccctcgccaccgc	MTHFPRHRYLLSQRAYEHRVI	fig|127906.87.peg.1059	VCC1059	VC1786		UPF0758 family protein		
1	CDS	1146444	1146620	+	atgagatttctgaaactaaaaga	MRFLKLKEVMEKTALSRSAIYF	fig|127906.87.peg.1060	VCC1060	VC1785		Predicted transcriptional regulator	AlpA family transcriptional regulator [unclassified Vibrio]	
1	CDS	1149112	1146767	-	atgcgtttcaaaaacgtaaagaa	MRFKNVKKTALMLAMFGMAT	fig|127906.87.peg.1061	VCC1061	VC1784	nanH	Sialidase (EC 3.2.1.18)		
1	CDS	1150671	1149535	-	atggcattaaaggcaatttcagc	MALKAISATRLFDGIQYYENAA	fig|127906.87.peg.1062	VCC1062	VC1783	nagA_2	N-acetylglucosamine-6-phosphate deacetylase (EC 3.5.1.25)		
1	CDS	1151538	1150675	-	atgcgcacactagccatcgatat	MRTLAIDIGGTKIALAIVEEGTII	fig|127906.87.peg.1063	VCC1063	VC1782	nanK	N-acetylmannosamine kinase (EC 2.7.1.60)		
1	CDS	1152236	1151526	-	gtgagaaagaatttttgaatatc	MRKNFLNIEELKRFLNGQTVV	fig|127906.87.peg.1064	VCC1064	VC1781	nanE	N-acetylmannosamine-6-phosphate 2-epimerase (EC 5.1.3.9)		
					ttgtctcaccattttcaaaaaaaactttatttcgtttgaaaaaaatcttcgttatga		From N16961	Not annotated as CDS	VC1780		Hypothetical protein		Only annotated as CDS in N16961
1	CDS	1152463	1153428	+	atgaaaaccattaataagattac	MKTINKITIAILTLSAAASVNAAT	fig|127906.87.peg.1065	VCC1065	VC1779		TRAP-type transport system, periplasmic component, predicted N-acetylneuraminate-binding protein		
1	CDS	1153479	1154000	+	atggagcttaagatgttacgtaa	MELKMLRKIINNIEEIITVPLMA	fig|127906.87.peg.1066	VCC1066	VC1778		TRAP-type transport system, small permease component, predicted N-acetylneuraminate transporter		
1	CDS	1154008	1155291	+	atggttggttcaattttggttggtta	MVGSIFGWLGLLFAGMPVGF	fig|127906.87.peg.1067	VCC1067	VC1777		TRAP-type transport system, large permease component, predicted N-acetylneuraminate transporter		
1	CDS	1155329	1156225	+	atgaaaaaattaacaggtttgat	MKKLTGLIAAPHTPFTKDNKV	fig|127906.87.peg.1068	VCC1068	VC1776		N-acetylneuraminate lyase (EC 4.1.3.3)		
1	CDS	1156354	1157190	+	gtgaatttgcctaaaaatctgatg	MNLPKNLMVRLRSNTEPLSKK	fig|127906.87.peg.1069	VCC1069	VC1775		Sialic acid utilization regulator, RpiR family		
1	CDS	1157372	1158526	+	atgaatatgaaaactctattgact	MNMKTLLTYATLLSVTAFSHV	fig|127906.87.peg.1070	VCC1070	VC1774		N-acetylneuraminate epimerase (EC 5.1.3.24)		
1	CDS	1158541	1159611	+	atggcaatctcatttcaattactc	MAISFQLLPELPVGVKHGVGG	fig|127906.87.peg.1071	VCC1071	VC1773	nanM_1	N-acetylneuraminate epimerase (EC 5.1.3.24)		
1	CDS	1160536	1159676	-	atgagtgcatggccaattcgatg	MSAWPIRWDLLFRYRMIEIIAL	fig|127906.87.peg.1072	VCC1072	VC1772		hypothetical protein	YafY family transcriptional regulator [Vibrio cholerae]	
1	CDS	1160609	1164226	+	atggtccgattaataaaacatca	MVRLIKHQQLLGEYCMNVSIE	fig|127906.87.peg.1073	VCC1073	VC1771		hypothetical protein		
1	CDS	1164208	1166271	+	atggttacaccacagctagaac	MVTPQLEPSSQGPLSTLIEQIS	fig|127906.87.peg.1074	VCC1074	VC1770		hypothetical protein		
1	CDS	1166412	1168793	+	atggaccactcagtacataaca	MDHSVHNKLVSFIWSIADDCL	fig|127906.87.peg.1075	VCC1075	VC1769		Type I restriction-modification system, DNA-methyltransferase subunit M (EC 2.1.1.72)		
1	CDS	1168798	1170138	+	atgagcaatactatgattaagaa	MSNTMIKKMPKYESYKDSCE	fig|127906.87.peg.1076	VCC1076	VC1768		Type I restriction-modification system, specificity subunit S		
1	CDS	1170139	1171551	+	atggcggttgttgttgccagttgca	MAVVVASCTLDKLFSNESILAS	fig|127906.87.peg.1077	VCC1077	VC1767		hypothetical protein	DUF262 domain-containing protein [Vibrio cholerae]	
1	CDS	1171544	1173535	+	atgggtgatcatttatcggtgcaa	MGDHLSVQTELLTLEKIYTDN	fig|127906.87.peg.1078	VCC1078	VC1766		hypothetical protein	DUF262 domain-containing protein [Vibrio cholerae]	
1	CDS	1173572	1176631	+	atggttagtaaaacaaatgaac	MVSKTNEQALEAAIEKGLAGI	fig|127906.87.peg.1079	VCC1079	VC1765	hsdR	Type I restriction-modification system, restriction subunit R (EC 3.1.21.3)		
1	CDS	1176676	1178796	+	atgaccgatagccttaatctttcc	MTDSLNLSSLWPDLSSLNVMI	fig|127906.87.peg.1080	VCC1080	VC1764		putative membrane protein	anti-phage defense ZorAB system ZorA [Vibrio cholerae]	
1	CDS	1178789	1179523	+	atgcgtagaggtgttcgttcgcat	MRRGVRSHTQNSVDEENPYV	fig|127906.87.peg.1081	VCC1081	VC1763		OmpA/MotB domain protein		
1	CDS	1179520	1180971	+	gtgagtctaaatataaaaaagct	MSLNIKKLKVSLPANPFGQAIK	fig|127906.87.peg.1082	VCC1082	VC1762		hypothetical protein	transcriptional regulator [Vibrio cholerae]	
1	CDS	1180962	1184411	+	gtggagttaacctctttaatcagc	MELTSLISKFFSSSDKTSQFEL	fig|127906.87.peg.1083	VCC1083	VC1760		Putative helicase		Limited query cover for certain strains
1	CDS	1184997	1184542	-	atgaaaaaaagaagagagcac	MKKRREHRIPLKEQMLALLEV	fig|127906.87.peg.1084	VCC1084	VC1759		Phage integrase		VC1759 annotated as pseudogene in KEGG, no refseq ref in patric
1	CDS	1186328	1185093	-	atggcaaataatctaaccgcaa	MANNLTARQIQTAKPKDKLYR	fig|127906.87.peg.1085	VCC1085	VC1758		Phage integrase		
1	rna	1186728	1186641	-	ggtgagttgtccgagtggctgaaggagcacgcctggaaagtgtgtatacgg		fig|127906.87.rna.46	VCCt033	VCt062		tRNA-Ser-GGA		
1	CDS	1189990	1186940	-	atggatattgcacgttataccctt	MDIARYTLAKRTSVWVLIALTL	fig|127906.87.peg.1086	VCC1086	VC1757	swrC	RND efflux system, inner membrane transporter		
1	CDS	1191084	1189990	-	atgaatccttcttttactcgttcaat	MNPSFTRSMAAKRSLLSAVIA	fig|127906.87.peg.1087	VCC1087	VC1756	srpA	RND efflux system, membrane fusion protein		
1	CDS	1191878	1191300	-	atgaaaccctggttattgatgatg	MKPWLLMMLTTLLVGCGGTP	fig|127906.87.peg.1088	VCC1088	VC1755		putative lipoprotein	membrane integrity-associated transporter subunit PqiC [Vibrio cholerae]	
1	CDS	1193503	1191875	-	atgagtgatgaacctcaagttaa	MSDEPQVNAEITAQKQLSAIW	fig|127906.87.peg.1089	VCC1089	VC1754	pqiB	Paraquat-inducible protein B		
1	CDS	1194813	1193515	-	atggacaaactaaagaatcatt	MDKLKNHLIGCDECGLITNVPI	fig|127906.87.peg.1090	VCC1090	VC1753	pqiA	Paraquat-inducible protein A		
1	CDS	1195014	1196750	+	atgataaccaaaaagtgtagat	MITKKCRYAMKPRWSWVALLI	fig|127906.87.peg.1091	VCC1091	VC1751		Small-conductance mechanosensitive channel		
1	CDS	1197887	1196766	-	atgagattcatcactactctactg	MRFITTLLCFLLAILQPVQAIPT	fig|127906.87.peg.1092	VCC1092	VC1750		corresponds to STY3950 from Accession AL513382: Salmonella typhi CT18		
1	CDS	1199010	1197898	-	atgaaaacaaatactctgatttt	MKTNTLIFLITLGFSLLAEASDN	fig|127906.87.peg.1093	VCC1093	VC1749		Membrane protein	DUF4056 domain-containing protein [Vibrio cholerae]	
1	CDS	1199848	1199024	-	atgatattacgaacactactgac	MILRTLLTLTVFCFPFTTWASE	fig|127906.87.peg.1094	VCC1094	VC1748		hypothetical protein	TonB-dependent receptor [Vibrio cholerae]	
1	CDS	1200075	1200704	+	atgaaactcagtgagcaaaaa	MKLSEQKRLALIEAAKEEFTQ	fig|127906.87.peg.1095	VCC1095	VC1746	rutR	Transcriptional regulator, AcrR family		
1	CDS	1200737	1202188	+	atgttaccgaccgattttccatttt	MLPTDFPFFHQSAFINGQWIL	fig|127906.87.peg.1096	VCC1096	VC1745	gabD	Succinate-semialdehyde dehydrogenase [NAD(P)+] (EC 1.2.1.16)		
1	CDS	1202450	1202169	-	atgcgtactttgattggggtagtc	MRTLIGVVVTLSYSVALHAAPT	fig|127906.87.peg.1097	VCC1097	VC1744		hypothetical protein	YnjH family protein [Vibrio cholerae]	
1	CDS	1203406	1202495	-	atgctgatcactcaatcacctatc	MLITQSPITEHRFAGQTFYLKR	fig|127906.87.peg.1098	VCC1098	VC1743		pyridoxal phosphate-dependent deaminase, putative		
1	CDS	1203615	1203499	-	ttgttttgggcgtttttttatctggttta	MFWAFFYLVYFVQVGSLAIFQ	fig|127906.87.peg.1099	VCC1099	VC1742		FIG01202883: hypothetical protein		
1	CDS	1203939	1204565	+	atggcattacgcagtagtactaa	MALRSSTKEKILDVAEGLFAEY	fig|127906.87.peg.1100	VCC1100	VC1741	yttP	Predicted transcriptional regulator for fatty acid degradation FadQ, TetR family		
1	CDS	1204621	1206903	+	atgagctctctacgcagaaaatg	MSSLRRKWISDPAFKMFKKVL	fig|127906.87.peg.1101	VCC1101	VC1740	fadE	Acyl-CoA dehydrogenase		
1	CDS	1207102	1208307	+	atgatcatcaaacctaaaattcg	MIIKPKIRGFICTTTHPVGCEAI	fig|127906.87.peg.1102	VCC1102	VC1739		Enoyl-[acyl-carrier-protein] reductase [NADH] (EC 1.3.1.9), FabV => refractory to triclosan		VC1739/38 are annotated as 2 ORFs instead of 1 ORF as in the other strains. Annotated as 1 ORF pseudogene named VC1738 in KEGG
									VC1738				VC1739/38 are annotated as 2 ORFs instead of 1 ORF as in the other strains. Annotated as 1 ORF pseudogene named VC1738 in KEGG
1	CDS	1208575	1208411	-	atgctccgctttttgttttgctacag	MLRFLFCYSTYQSMRSQNIDC	fig|127906.87.peg.1103	VCC1103	Not annotated as CDS		Phage shock protein C		
1	CDS	1208829	1208611	-	atggctaaagaagacgtaattga	MAKEDVIEMQGTVLDTLPNTM	fig|127906.87.peg.1104	VCC1104	VC1737	infA	Translation initiation factor 1		
1	CDS	1209614	1208913	-	atgagctcagatattcagcaaat	MSSDIQQIRIGLTNNHPCSYLA	fig|127906.87.peg.1105	VCC1105	VC1736		Arginyl-tRNA--protein transferase (EC 2.3.2.8)		
1	CDS	1210349	1209618	-	atgagctatttatctgactgaattatc	MAIYLTELSPETLTFPSPFTALI	fig|127906.87.peg.1106	VCC1106	VC1735	aat	Leucyl/phenylalanyl-tRNA--protein transferase (EC 2.3.2.6)		
1	CDS	1210573	1210409	-	atgggtcgcggcgaaaattcttc	MGRGENSSYPQTNQIHALALA	fig|127906.87.peg.1107	VCC1107	VC1733		hypothetical protein		Limited query cover in certain strains
1	CDS	1210616	1211896	+	atggaaagtttgactctacaacc	MESLTLQPIELISGEVNLPGSK	fig|127906.87.peg.1108	VCC1108	VC1732	aroA	3-phosphoshikimate 1-carboxyvinyltransferase (EC 2.5.1.19)		
1	CDS	1212235	1211987	-	atgagccataaaaaaccgatca	MSHKKPITEFELLLIANQLIQDH	fig|127906.87.peg.1109	VCC1109	VC1731		Protein YciN		
1	CDS	1212572	1215202	+	atgggtaaatcactcgttatcgtc	MGKSLVIVESPAKAKTINKYLG	fig|127906.87.peg.1110	VCC1110	VC1730	topA	DNA topoisomerase I (EC 5.99.1.2)		
1	CDS	1215739	1215605	-	atgcaatcggttggctatacgtta	MQSVGYTLSNPWPNTNQFLK	fig|127906.87.peg.1111	VCC1111	VC1728		FIG01199823: hypothetical protein		
1	CDS	1215871	1217088	+	atggctggagtattaggcatgatt	MAGVLGMILAGGEGSRLKPLT	fig|127906.87.peg.1112	VCC1112	VC1727	glgC	Glucose-1-phosphate adenylyltransferase (EC 2.7.7.27)		
1	CDS	1217090	1218544	+	atggaacaattcaacgtttggtta	MEQFNVWFTVSEVQGLVKSG	fig|127906.87.peg.1113	VCC1113	VC1726	glgA	Glycogen synthase, ADP-glucose transglucosylase (EC 2.4.1.21)		
1	CDS	1218732	1219550	+	atgtcttcccctatgactgccattg	MSSPMTAIAEKVLFHKTYLHPT	fig|127906.87.peg.1114	VCC1114	VC1725		Beta-ketoadipate enol-lactone hydrolase, putative		
1	CDS	1219866	1219555	-	atgaaaatctacacggccactc	MKIYTATHPLEAHMLMQLLHQ	fig|127906.87.peg.1115	VCC1115	VC1724		hypothetical protein	DUF2007 domain-containing protein [Vibrio cholerae]	
1	CDS	1220589	1219900	-	atgtataaaaaactcatcatcgc	MYKKLIIAIVLIAIAALLVTQFSQ	fig|127906.87.peg.1116	VCC1116	VC1723	yjdZ	COG0398: uncharacterized membrane protein	TVP38/TMEM64 family protein [Vibrio cholerae]	
1	CDS	1221531	1220944	-	atggataaaccagtattaaaaga	MDKPVLKDSMRLFEQLGRVK	fig|127906.87.peg.1117	VCC1117	VC1722		Positive regulator of competence TfoX		
1	CDS	1222900	1221890	-	atggctacgattaaagatgtggc	MATIKDVARLAGVSTTTVSHVI	fig|127906.87.peg.1118	VCC1118	VC1721	purR	Purine nucleotide synthesis repressor		
1	CDS	1223969	1223307	-	atgatgcaagaactgaaaatcc	MMQELKILNEKRAEIYWWLSS	fig|127906.87.peg.1119	VCC1119	VC1720	torD	TorA-specific chaperone protein, TorD		
1	CDS	1224194	1224898	+	atgagctatcacgtacttgtggttg	MSYHVLVVEDDLVTRSKLSGY	fig|127906.87.peg.1120	VCC1120	VC1719	torR	Trimethylamine-N-oxide operon transcriptional regulatory protein TorR		
1	CDS	1225819	1224998	-	atgtttgagctgaaaaaaatattt	MFELKKIFSSLLMPLPALLIIGF	fig|127906.87.peg.1121	VCC1121	VC1718		Membrane Protein Functionally coupled to the MukBEF Chromosome Partitioning Mechanism		
1	CDS	1225936	1226718	+	gtgacagaagatcgcaatttcga	MTEDRNFDDIAHKFAKNIYGSI	fig|127906.87.peg.1122	VCC1122	VC1717	smtA	tRNA uridine 5-oxyacetic acid(34) methyltransferase (EC 2.1.1.-)		
1	CDS	1226730	1228067	+	atgagtgagtttactcaggatact	MSEFTQDTVQKPIDELVTWVK	fig|127906.87.peg.1123	VCC1123	VC1716	mukF	Chromosome partition protein MukF		
1	CDS	1228048	1228770	+	atgtcatcgaccgatattaatgaa	MSSTDINEYMPENLAKAIANPL	fig|127906.87.peg.1124	VCC1124	VC1715	mukE	Chromosome partition protein MukE		
1	CDS	1228767	1233242	+	atgattgaacgaggtaaatatca	MIERGKYQSLTMINWNGFFAR	fig|127906.87.peg.1125	VCC1125	VC1714	mukB	Chromosome partition protein MukB		
1	CDS	1233767	1233324	-	atggataggtttgacgaaagaat	MDRFDERILQELQLDGRVSNV	fig|127906.87.peg.1126	VCC1126	VC1713		Transcriptional regulator, AsnC family		
1	CDS	1233875	1234459	+	atggattacaccttatttgggccg	MDYTLFGPLLAFAFVSTFSPG	fig|127906.87.peg.1127	VCC1127	VC1712		Transporter, LysE family	LysE family translocator [Vibrio cholerae]	
1	CDS	1236876	1234507	-	atgcacagcgaagttaccccta	MHSEVTPIHHYKKYWAECFGT	fig|127906.87.peg.1128	VCC1128	VC1711		UPF0313 [4Fe-4S] protein YgiQ		
1	CDS	1237156	1239612	+	atgagtgtgaatgtatcaaccgta	MSVNVSTVQPQSIGLKTRYAP	fig|127906.87.peg.1129	VCC1129	VC1710		hypothetical protein	EAL domain-containing protein [Vibrio cholerae]	
1	CDS	1242514	1239746	-	atgaaaattcgattattagtcagc	MKIRLLVSILCILFAGCALQQTN	fig|127906.87.peg.1130	VCC1130	VC1709		Probable zinc protease pqqL (EC 3.4.99.-)		
1	CDS	1242738	1243424	+	atgctttattctctgatttccgtattc	MLYSLISVFAPMLLGAQIILTLV	fig|127906.87.peg.1131	VCC1131	VC1708		hypothetical protein	putative membrane protein [Vibrio cholerae HC-1A2]	
1	CDS	1243541	1243741	+	atggatctgagcaacgtacgaa	MDLSNVRNFDSVILLGIVNEKL	fig|127906.87.peg.1132	VCC1132	VC1707		Uncharacterized protein VC1707	DUF4250 domain-containing protein [Vibrio cholerae]	
1	CDS	1244665	1243748	-	atgatagagctgaaacatttgaa	MIELKHLKTLISLRDTGSLTATA	fig|127906.87.peg.1133	VCC1133	VC1706	metR	Transcriptional activator MetR		
1	CDS	1245061	1247346	+	atgacaacgacacacattcttgc	MTTTHILGYPRIGEKRELKFAL	fig|127906.87.peg.1134	VCC1134	VC1704	metE	5-methyltetrahydropteroyltriglutamate--homocysteine methyltransferase (EC 2.1.1.14)		
1	CDS	1248019	1247621	-	atgaaaaaaatcgtcctgagca	MKKIVLSSVIASTLLLVGCSSG	fig|127906.87.peg.1135	VCC1135	VC1703		hypothetical protein		
1	CDS	1248317	1248021	-	atgtggtatgtgatttttgcgcagg	MWYVIFAQDVENSLEKRLSVR	fig|127906.87.peg.1136	VCC1136	VC1702		YciL protein		
1	CDS	1248811	1248392	-	atgaatcaaaccgtaaattcacc	MNQTVNSPKGQLLLRTLAMPA	fig|127906.87.peg.1137	VCC1137	VC1701		Acyl-CoA thioesterase YciA, involved in membrane biogenesis		
1	CDS	1249515	1248970	-	atgaaacaactgctcgactttatt	MKQLLDFIPLIIFFALYKFYDIYV	fig|127906.87.peg.1138	VCC1138	VC1700	yciB	Intracellular septation protein IspA		
1	CDS	1249972	1249748	-	atggaattacaacgtcaccactg	MELQRHHCYDLIHIGMRELLEI	fig|127906.87.peg.1139	VCC1139	VC1699		Mu-like prophage protein gp16		
1	CDS	1250797	1250150	-	atgagtaaacgcttgtatcaaca	MSKRLYQQIETLWDYLQLHQC	fig|127906.87.peg.1140	VCC1140	VC1698		Uncharacterized SAM-binding protein YdcF	YdcF family protein [Vibrio cholerae]	
1	CDS	1251620	1250790	-	atgaaaagagttgtcgtgatagg	MKRVVVIGAGWLGLPLAQWLI	fig|127906.87.peg.1141	VCC1141	VC1697		Protein yeeZ precursor		
1	CDS	1252008	1251724	-	atgctgcgcagcctattgttgccg	MLRSLLLPQQQLKKVIAMKFT	fig|127906.87.peg.1142	VCC1142	VC1696		DNA-binding protein inhibitor Id-2-related protein		
1	CDS	1252309	1253151	+	atggaacataaccaattcgattc	MEHNQFDSLLPPQMAERAAIT	fig|127906.87.peg.1143	VCC1143	VC1695		Formate efflux transporter FocA		
1	CDS	1253831	1254013	+	atgagtgatgttaataaagtcga	MSDVNKVESGEKRSLEWKSF	fig|127906.87.peg.1144	VCC1144	VC1694	torE	TorE protein		
1	CDS	1254060	1255244	+	atgaaatcgttaattttaaaaatg	MKSLILKMWHTMTRPAVHISL	fig|127906.87.peg.1145	VCC1145	VC1693	torC	Trimethylamine-N-oxide reductase associated c-type cytochrome, TorC		
1	CDS	1255268	1257730	+	atggcgattacacgaagaagttt	MAITRRSFLKGVATTSAASIIGI	fig|127906.87.peg.1146	VCC1146	VC1692	torA	Trimethylamine-N-oxide reductase (EC 1.7.2.3), TorA		
1	CDS	1257757	1257876	+	atgcgcgttatccctcaagccag	MRVIPQASVQRWLFSSLLSVN	fig|127906.87.peg.1147	VCC1147	No refSeq annotation		FIG01206054: hypothetical protein		
1	CDS	1257937	1259061	+	atgagccatctcagattagcgaa	MSHLRLANGREILVQQADIELE	fig|127906.87.peg.1148	VCC1148	VC1690	rafA	Alpha-1,6-galactosidase, putative		
1	CDS	1259113	1259673	+	atgtggccttcactgggtttggtgc	MWPSLGLVDAMRVSDDVERN	fig|127906.87.peg.1149	VCC1149	VC1689		Alpha-galactosidase		
1	CDS	1260945	1259737	-	atgaactctctaaaatggccaaa	MNSLKWPNMANHRVLVSLFN	fig|127906.87.peg.1150	VCC1150	VC1688	mntX	Manganese transport protein MntX		
1	CDS	1261131	1262036	+	atgattttagtcgttggtcataaaa	MILVVGHKNPDSDSICSALVAA	fig|127906.87.peg.1151	VCC1151	VC1687	ppaC	Manganese-dependent inorganic pyrophosphatase (EC 3.6.1.1)		
1	CDS	1262171	1262560	+	atgtccaatccattaaccgcaga	MSNPLTAEQMALINQYDQEQF	fig|127906.87.peg.1152	VCC1152	VC1686		hypothetical protein	DUF2750 domain-containing protein [Vibrio cholerae]	
1	CDS	1263367	1262597	-	atgatgaaaagagggattgcgc	MMKRGIALGLLTLISHCAFGQE	fig|127906.87.peg.1153	VCC1153	VC1685		hypothetical protein	DUF2927 domain-containing protein [Vibrio cholerae]	
1	CDS	1264156	1263368	-	atgagtgcgctgttagaagtcac	MSALLEVTNLHKDFVTRSGFL	fig|127906.87.peg.1154	VCC1154	VC1684	sapF	Peptide ABC transporter, ATP-binding protein SapF		
1	CDS	1265148	1264153	-	atgccgttacttgatattcgtcact	MPLLDIRHLTIEIDTPQGLVKAV	fig|127906.87.peg.1155	VCC1155	VC1683	sapD	Peptide ABC transporter, ATP-binding protein SapD		
1	CDS	1266035	1265148	-	atgttaagcgatagtatttatcaa	MLSDSIYQEEHIPTQFERFWR	fig|127906.87.peg.1156	VCC1156	VC1682	sapC	Peptide ABC transporter, permease protein SapC		
1	CDS	1266984	1266022	-	atgttcgtctatacggtccgtaaa	MFVYTVRKFNLFIITLLILTMVG	fig|127906.87.peg.1157	VCC1157	VC1681	sapB	Peptide ABC transporter, permease protein SapB		
1	CDS	1268567	1266984	-	atgactctgctggcaggctgcgc	MTLLAGCGENIDHSKIRSSGFY	fig|127906.87.peg.1158	VCC1158	VC1680	sapA	Peptide ABC transporter, substrate-binding protein SapA		
1	CDS	1269736	1268720	-	atgcaacagagcttgctcggag	MQQSLLGESPAFLAVLDKVSR	fig|127906.87.peg.1159	VCC1159	VC1679	pspF	Psp operon transcriptional activator		
1	CDS	1269946	1270614	+	atgggtatttttctcgttttgcaga	MGIFSRFADIVNSNISALLDKA	fig|127906.87.peg.1160	VCC1160	VC1678	pspA	Phage shock protein A @ Suppressor of sigma54-dependent transcription, PspA-like		
1	CDS	1270628	1270861	+	atgtcctcatttttatcgctgtgcc	MSSFFIAVPLIVFCIFVAPLWLI	fig|127906.87.peg.1161	VCC1161	VC1677	pspB	Phage shock protein B		
1	CDS	1270827	1271243	+	atgaaaccccaaactggagaa	MKPQTGEIAMSKRELYRDPYN	fig|127906.87.peg.1162	VCC1162	VC1676	pspC	Phage shock protein C		
1	CDS	1271415	1272446	+	atgaacaaaaccctttggcctt	MNKTLLAFSICSTLLVGCGKEL	fig|127906.87.peg.1163	VCC1163	VC1675	mexA	RND efflux system, membrane fusion protein		
1	CDS	1272448	1273527	+	atgaaaataattcgttacaccgc	MKIIRYTAWLAHLLLIAGCSEP	fig|127906.87.peg.1164	VCC1164	VC1674	ttgG	RND efflux system, membrane fusion protein		
1	CDS	1273520	1276618	+	atgagtgaacagaagcagacc	MSEQKQTPPENNENQGVAAY	fig|127906.87.peg.1165	VCC1165	VC1673	czcA	RND efflux system, inner membrane transporter		
1	CDS	1276705	1277286	+	atgatgaatgcggaacagggcg	MMNAEQGVCAWAMNHPLER	fig|127906.87.peg.1166	VCC1166	VC1672	tag	DNA-3-methyladenine glycosylase (EC 3.2.2.20)		
1	CDS	1278563	1277373	-	atgtcagaaggtaaaaagacc	MSEGKKTRLVTAGRNKKWTL	fig|127906.87.peg.1167	VCC1167	VC1671	metC	Cystathionine beta-lyase (EC 4.4.1.8)		
1	CDS	1280098	1278644	-	atggacaaggtttatcacttcctc	MDKVYHFLTLLSIGIYWLLVAG	fig|127906.87.peg.1168	VCC1168	VC1670	cls	Cardiolipin synthase, bacterial type ClsA		
1	CDS	1280714	1282000	+	atggctgcagcgggtctgcggt	MAAAGSAVGLGNVWGFPTQA	fig|127906.87.peg.1169	VCC1169	VC1669		Putative sodium-dependent transporter		
1	CDS	1282099	1282806	+	atgtttgatattctgtttacccatcc	MFDILFTHPDFLLINKHPNVSV	fig|127906.87.peg.1170	VCC1170	VC1668		Pseudouridine synthase (EC 4.2.1.70), VC1668 type		
1	CDS	1283485	1282865	-	atgcttgatcgctacgcgataaa	MLDRYAIKIIRWPLHQAAGLLD	fig|127906.87.peg.1171	VCC1171	VC1667	ynjF	Putative phosphatidylglycerophosphate synthase		
1	CDS	1284140	1283478	-	atgatattttctctcgaaaacctcg	MIFSLENLAILKQDGHCLLAEF	fig|127906.87.peg.1172	VCC1172	VC1666	ynjD	ABC transporter, ATP-binding protein YnjD		
1	CDS	1285806	1284106	-	atgcttagaactgcctattggggt	MLRTAYWGFILLCVAPILPGLA	fig|127906.87.peg.1173	VCC1173	VC1665	ynjC	ABC transporter, permease protein YnjC		
1	CDS	1286968	1285820	-	atgaaaaaaatgctgacttttctc	MKKMLTFLTTCALSFGVSAQS	fig|127906.87.peg.1174	VCC1174	VC1664	ynjB	ABC transporter, substrate-binding protein YnjB		
1	CDS	1287619	1287176	-	atgaagcttagttcaaaatcatta	MKLSSKSLLAAITLPVLMTACA	fig|127906.87.peg.1175	VCC1175	VC1663	hslJ	Heat shock protein HslJ		
1	CDS	1287766	1288032	+	atggagcaattggagtttttcaat	MEQLEFFNIVSPCIGVCSVDEI	fig|127906.87.peg.1176	VCC1176	VC1662		Predicted Fe-S protein		
1	CDS	1288253	1288038	-	atgaaacgcaatagcaagagtt	MKRNSKSYRLKLIREVATRKQ	fig|127906.87.peg.1177	VCC1177	VC1661		hypothetical protein		
1	CDS	1289515	1288418	-	atgaacagcctgtttcgcattaaa	MNSLFRIKAVLVKEFRQLSRDI	fig|127906.87.peg.1178	VCC1178	VC1660	ybhS	Domain of unknown function / Efflux ABC transporter, permease protein	ABC transporter permease [Vibrio cholerae]	
1	CDS	1290438	1289512	-	atgagcgattacgccatccatgc	MSDYAIHAENVVKQFGHFTAII	fig|127906.87.peg.1179	VCC1179	No refSeq annotation		Efflux ABC transporter, ATP-binding protein		
1	CDS	1291395	1290448	-	atgattcaatctctctcaatagca	MIQSLSIASLLALLLSACSAEN	fig|127906.87.peg.1180	VCC1180	VC1659		Membrane-fusion protein	HlyD family efflux transporter periplasmic adaptor subunit [Vibrio cholerae]	
1	CDS	1292817	1291564	-	gtgaaagactctcaggaagtga	MKDSQEVTHFSAARSSKWTQ	fig|127906.87.peg.1181	VCC1181	VC1658		Serine transporter	HAAAP family serine/threonine permease [Vibrio cholerae]	
1	CDS	1293126	1293001	-	atgacagcgattgtgcgcgaaa	MTAIVREKRAAYQRRFPLGID	fig|127906.87.peg.1182	VCC1182	VC1657		FIG01202512: hypothetical protein		
1	CDS	1293153	1294016	+	atgcgtactcgttttcctgctgaat	MRTRFPAELLLVVTTLLAAGG	fig|127906.87.peg.1183	VCC1183	VC1656		Permease of the drug/metabolite transporter (DMT) superfamily		
1	CDS	1295451	1294099	-	atgaatgtaatgaaaacaaacc	MNVMKTNHSVVAAAQLKQLN	fig|127906.87.peg.1184	VCC1184	VC1655		Mg/Co/Ni transporter MgtE, CBS domain-containing		
1	CDS	1296155	1299685	+	gtgcgtgctgcggattatttggat	MRAADYLDPNEQAYVEQKST	fig|127906.87.peg.1185	VCC1185	VC1653	vieS	Sensory box sensor histidine kinase/response regulator VieS		
1	CDS	1299690	1301444	+	atgaaaataatgatagtagaag	MKIMIVEDDRIQATSLKLKLRQ	fig|127906.87.peg.1186	VCC1186	VC1652	vieA	Response regulator VieA		
1	CDS	1301456	1303117	+	atggctgtacctacttttgctgaat	MAVPTFAELKVLIIDDAPIVIAS	fig|127906.87.peg.1187	VCC1187	VC1651	vieB	Response regulator VieB		
1	CDS	1305598	1303151	-	gtgtcttttcaacaacgtatccaa	MSFQQRIQHHPIAWACVIAGL	fig|127906.87.peg.1188	VCC1188	VC1650	prt/vcc	Microbial collagenase, secreted (EC 3.4.24.3)		
1	CDS	1305926	1307572	+	atgaacaaaacatttttatcagga	MNKTFLSGVVGTLLFTSFQFS	fig|127906.87.peg.1189	VCC1189	VC1649		trypsin, putative		
1	CDS	1309416	1307653	-	atgtctgatctcaatttattgcgcta	MSDLNLLRYYARLTPLGVGHD	fig|127906.87.peg.1190	VCC1190	VC1647		ABC transporter, substrate-binding protein (cluster 5, nickel/peptides/opines)		
1	CDS	1309646	1309810	+	atgttcatatcacccgttaacgaa	MFISPVNEQTMAAYYQQRLQ	fig|127906.87.peg.1191	VCC1191	VC1646		FIG01201865: hypothetical protein		
1	CDS	1309981	1310589	+	atggcaatccgtaatgtggtatttg	MAIRNVVFDVGNVIVKWAPQD	fig|127906.87.peg.1192	VCC1192	VC1645	yihX	HAD superfamily hydrolase		
1	CDS	1311896	1310706	-	atgcataaaccactctccatagc	MHKPLSIAVLLTVALVGCQSE	fig|127906.87.peg.1193	VCC1193	VC1644		hypothetical protein	DUF3103 domain-containing protein [Vibrio cholerae]	
1	CDS	1313985	1312282	-	atggtatccaatcaacataacaa	MVSNQHNNAHRTIIAGTTNSLI	fig|127906.87.peg.1194	VCC1194	VC1643		Methyl-accepting chemotaxis sensor/transducer protein		
1	CDS	1314263	1314664	+	atgccaacaaccgatcaagcga	MPTTDQAIAITLPSLIHRIGSDT	fig|127906.87.peg.1195	VCC1195	VC1642		hypothetical protein	ribosome recycling factor [Vibrio cholerae]	
1	CDS	1314756	1315529	+	atgcatattattcctgtggcgtcag	MHIIPVASDPKTCSCGVNENS	fig|127906.87.peg.1196	VCC1196	VC1641		FOG: EAL domain protein		
1	CDS	1315957	1315679	-	atgaaattcgaagcagtattacg	MKFEAVLRTDLGKGASRRLRN	fig|127906.87.peg.1197	VCC1197	VC1640	rplY	LSU ribosomal protein L25p		
1	CDS	1317488	1316115	-	atgccaattaaatctcgggcgtta	MPIKSRALRHLSLKTRLLLAAA	fig|127906.87.peg.1198	VCC1198	VC1639	phoQ	Sensor histidine kinase VC_1639		
1	CDS	1318137	1317478	-	atgaaaatattagttgttgaagat	MKILVVEDDVQLGQQILSALE	fig|127906.87.peg.1199	VCC1199	VC1638	mprA	Two-component system response regulator QseB		
1	CDS	1318550	1318149	-	atgcagatttttcgtatgcagcaa	MQIFRMQQRPQASLLLLLLLL	fig|127906.87.peg.1200	VCC1200	VC1637		Membrane protein	PepSY domain-containing protein [Vibrio]	
1	repeat	1318711	1318838	+	tataccaaaactacttggagttgcaggtaggcggcaagagagtgaatcccc		fig|127906.87.repeat.21				repeat region		
1	CDS	1320605	1318821	-	atgtatacgttacgaccttatcaa	MYTLRPYQADSVKAVVHYFR	fig|127906.87.peg.1201	VCC1201	VC1636		ATP-dependent RNA helicase YejH		
1	CDS	1320739	1321434	+	atgcgtttagacaaattcctgtgt	MRLDKFLCDALGTTRKEATQL	fig|127906.87.peg.1202	VCC1202	VC1635	rsuA	SSU rRNA pseudouridine(516) synthase (EC 5.4.99.19)		
1	CDS	1321753	1322958	+	atgtctgcctcaaccgtgaatac	MSASTVNTQQTPQMSLLLYIV	fig|127906.87.peg.1203	VCC1203	VC1634	bcr	Multidrug resistance transporter => Bicyclomycin resistance protein Bcr-1		
1	CDS	1322958	1323617	+	atggcgaattaccatttagaaatt	MANYHLEIQNVVNTALAELEA	fig|127906.87.peg.1204	VCC1204	VC1633		hypothetical protein	DUF2913 family protein [Vibrio cholerae]	
1	CDS	1325120	1323684	-	atgaaaaaggtattagtgttagg	MKKVLVLGASGYVGAQLLPLL	fig|127906.87.peg.1205	VCC1205	VC1632		Predicted nucleoside-diphosphate-sugar epimerases		
1	CDS	1325248	1326624	+	gtgtctgttgtctctcaagttttatc	MSVVSQVLSHMRGDFFRRLIA	fig|127906.87.peg.1206	VCC1206	VC1631		Na+-driven multidrug efflux pump		
1	CDS	1326750	1327421	+	atgttacagctaatagacctatgc	MLQLIDLCKGYVDGDEFHPVL	fig|127906.87.peg.1207	VCC1207	VC1630		AttE component of AttEFGH ABC transport system		
1	CDS	1327402	1329855	+	atgttatggcccgtagttaaggcc	MLWPVVKALLGHYRRYPLQIC	fig|127906.87.peg.1208	VCC1208	VC1629		ABC transporter, fused permease protein		
1	CDS	1329856	1330974	+	atgcgccgctttcgtttgcgcttga	MRRFRLRLKPWVWLSLGALF	fig|127906.87.peg.1209	VCC1209	VC1628		AttH component of AttEFGH ABC transport system		
1	CDS	1331253	1332401	+	atgtctgacatgattcgagatttct	MSDMIRDFFKMESAGGILLVIA	fig|127906.87.peg.1210	VCC1210	VC1627	nhaA	Na+/H+ antiporter NhaA type		
1	CDS	1332999	1335884	+	atgagtacagcctttgaagtcga	MSTAFEVDNHIATLFSTQVPLL	fig|127906.87.peg.1211	VCC1211	VC1625	dat	Diaminobutyrate--2-oxoglutarate aminotransferase (EC 2.6.1.76) / L-2,4-diaminobutyrate decarboxylase (EC 4.1.1.86)		ORF is plit into 2 ORFs and lacking seqref in N16961 according to PATRIC. Annotated as VC1625 and pseudogene in KEGG
1	CDS	1335898	1337142	+	atgtctattctacagattggtgcag	MSILQIGAGGVGWVVAHKAAC	fig|127906.87.peg.1212	VCC1212	VC1624		Carboxynorspermidine synthase (EC 1.5.1.43)		
1	repeat	1337177	1337306	+	ctatacccttcctacttgaagctgcagcggtgttggctacgttcgttcacccca		fig|127906.87.repeat.22				repeat region		

1	CDS	1337408	1338541	+	atgctaaaagacgaattgagaa	MLKDELRTPYFMIDEAKLIANL	fig|127906.87.peg.1213	VCC1213	VC1623	nspC	Carboxynorspermidine decarboxylase (EC 4.1.1.96)		
1	CDS	1339315	1338677	-	atgtcaatcaaatcatgggctctg	MSIKSWALPLFLCTVTTSALAN	fig|127906.87.peg.1214	VCC1214	VC1622	oprF	T1SS peptidoglycan-associated lipoprotein LapL		
1	CDS	1340654	1339317	-	atggcccacactggggagaac	MAHTGENVLKGLPLKAIALAIA	fig|127906.87.peg.1215	VCC1215	VC1621	bepC	Type I secretion system, outer membrane component LapE		
1	CDS	1341051	1347806	+	atgggaattcatgctttgttgtcttta	MGIHALLSLTNLAANQLLVIDK	fig|127906.87.peg.1216	VCC1216	VC1620		T1SS secreted agglutinin RTX		Limited query/subject cover for certain strains
1	CDS	1348464	1347916	-	atgtcaacggaaaccattattca	MSTETIIQEVDHWLNQVVIGLN	fig|127906.87.peg.1217	VCC1217	VC1619		hypothetical protein	DUF1415 domain-containing protein [Vibrio cholerae]	
1	CDS	1349738	1348521	-	atgattgaactgcattctccccaa	MIELHSPQYLKVTFALAFGSFL	fig|127906.87.peg.1218	VCC1218	VC1618		Uncharacterized MFS-type transporter		
1	CDS	1349844	1350713	+	atggaagttcgacaactgcgtca	MEVRQLRHFVTVAEWESFTLA	fig|127906.87.peg.1219	VCC1219	VC1617		Transcriptional regulator, LysR family		
1	CDS	1350977	1350744	-	atgaaacgcgtggttttgtacata	MKRVVLYIKDKCPHCKDAQRY	fig|127906.87.peg.1220	VCC1220	VC1616		Glutaredoxin		
1	CDS	1351145	1351657	+	atgagtatgcaccctgcactgaa	MSMHPALKYIQGYPQHIVEQV	fig|127906.87.peg.1221	VCC1221	VC1615		UTP pyrophosphatase (EC 3.6.1.9)		
1	CDS	1351654	1352742	+	atgaacactccacttaagccaa	MNTPLKPKHGQKTNRKPKAN	fig|127906.87.peg.1222	VCC1222	VC1614	rlmF	23S rRNA (adenine(1618)-N(6))-methyltransferase (EC 2.1.1.181)		
1	repeat	1352756	1352883	+	tatacccttcctagttaaagttgcagcggtgttggctacgttcgttcaccccaatc		fig|127906.87.repeat.23				repeat region		
1	CDS	1352941	1353165	+	atgagaattgaagacgttccact	MRIEDVPLYAPCQPHWLCVPI	fig|127906.87.peg.1223	VCC1223	VC1613		hypothetical protein		
1	CDS	1353882	1353169	-	atgaaaaatgtgtttggtttaggg	MKNVFGLGLIIALAGCVTVTET	fig|127906.87.peg.1224	VCC1224	VC1612		Type IV pilus (Tfp) assembly protein PilF		
1	CDS	1355032	1354091	-	gtgccgattagaatccccgacc	MPIRIPDQLPASDVLRNENIFV	fig|127906.87.peg.1225	VCC1225	VC1611	metA	Homoserine O-succinyltransferase (EC 2.3.1.46)		
1	CDS	1355173	1355547	+	atgcgacctcatttaacccttattc	MRPHLTLIRGLPGSGKSTLAK	fig|127906.87.peg.1226	VCC1226	VC1610		Predicted kinase	ATP-binding protein [Vibrio cholerae]	
1	CDS	1356699	1355551	-	atgaccctatttgagctatttaaag	MTLFELFKAELKALFTNPVVVL	fig|127906.87.peg.1227	VCC1227	VC1609		Efflux ABC transporter, permease protein		
1	CDS	1357844	1356696	-	atgaagcacgccatcccccgtc	MKHAIPRQWTLLRHDKWLFS	fig|127906.87.peg.1228	VCC1228	VC1608		Domain of unknown function / Efflux ABC transporter, permease protein		
1	CDS	1358837	1357863	-	atgaaaacggtaaaaacgacc	MKTVKTTLMTLTAVAVVGWIG	fig|127906.87.peg.1229	VCC1229	VC1607		Membrane-fusion protein	HlyD family secretion protein [Vibrio cholerae]	
1	CDS	1360279	1358849	-	atgaaagtgaaatctctctctctg	MKVKSLSLAICSILFMPIAHAAF	fig|127906.87.peg.1230	VCC1230	VC1606		Efflux transport system, outer membrane factor (OMF) lipoprotein		
1	CDS	1360680	1360447	-	atgagaagagatgcttttggaat	MRRDAFGICLSKDMLCEHLHS	fig|127906.87.peg.1231	VCC1231	Not annotated as CDS		hypothetical protein	cation transport ATPase [Vibrio cholerae]	
1	CDS	1361009	1362625	+	atggtgtggagcagtatcaccttc	MVWSSITFRKRMLIIMTLSGLI	fig|127906.87.peg.1232	VCC1232	VC1605	dpiB	Tricarboxylate transport sensor protein TctE => Citrate response regulator CitA		
1	CDS	1362625	1363311	+	atgaaaacccgactcctgatcat	MKTRLLIIEDDAAIAQLHHRYLS	fig|127906.87.peg.1233	VCC1233	VC1604	citT	Tricarboxylate transport transcriptional regulator TctD => Citrate response regulator CitB		
1	CDS	1363377	1364153	+	atgcaatggcgtttttgtttgacaa	MQWRFCLTTVLGVVLSFSPSV	fig|127906.87.peg.1234	VCC1234	VC1603		ABC-type amino acid transport/signal transduction system, periplasmic component/domain		
1	CDS	1364181	1365158	+	atgagcaacccaagcagtacta	MSNPSSTILTESGTNELEIIEFH	fig|127906.87.peg.1235	VCC1235	VC1602		Chemotaxis protein CheV (EC 2.7.3.-)		
1	CDS	1365290	1366093	+	atgcgcatatcttggtatccattc	MRISWLSILLWFSTSAFANHQ	fig|127906.87.peg.1236	VCC1236	VC1601		hypothetical protein		
1	CDS	1366208	1366897	+	atggttgaaatttttattgtcgtcat	MVEIFIVVILLVLFFFVIQKYVIR	fig|127906.87.peg.1237	VCC1237	VC1600		Potential queD like		
1	CDS	1367961	1366900	-	atggatgctaggttatttgacaat	MDARLFDNTQTLRASVLCGLS	fig|127906.87.peg.1238	VCC1238	VC1599		GGDEF domain protein		
											GGDEF domain protein		
1	rna	1368233	1368309	+	gcgtccgtagctcagttggttagagcaccaccttgacatggtgggggtcggtg		fig|127906.87.rna.47	VCCt034	VCt061		tRNA-Val-GAC		
1	rna	1368331	1368407	+	gcgtctatagctcagttggttagagcaccaccttgacatggtgggggtcggtg		fig|127906.87.rna.48	VCCt035	VCt060		tRNA-Val-GAC		
1	CDS	1369338	1368577	-	atgctgagcaacggatacttaaa	MLSNGYLKRYVRCCLLIIGLCP	fig|127906.87.peg.1239	VCC1239	VC1598		ABC-type amino acid transport/signal transduction system, periplasmic component/domain		
1	CDS	1369550	1370983	+	atgtcgcagcctgtagatagaaa	MSQPVDRKTFLGIFFTVFLPMI	fig|127906.87.peg.1240	VCC1240	VC1597		Uncharacterized MFS-type transporter		
1	CDS	1371427	1372491	+	atgtctgagcttaagtttaatcctg	MSELKFNPVDHPHRRYNPLTG	fig|127906.87.peg.1241	VCC1241	VC1596	galT	Galactose-1-phosphate uridylyltransferase (EC 2.7.7.10)		
1	CDS	1372557	1373717	+	atgtctgaattaatccaaaacgtg	MSELIQNVTTTFAQLFGYDATI	fig|127906.87.peg.1242	VCC1242	VC1595	galK	Galactokinase (EC 2.7.1.6)		
1	CDS	1373726	1374778	+	atgaacgcgttattcaccagcat	MNALFTSMTAQVAYDGQPAK	fig|127906.87.peg.1243	VCC1243	VC1594	galM	Aldose 1-epimerase (EC 5.1.3.3)		
1	CDS	1376082	1374796	-	atggacacagataaccgctgga	MDTDNRWRIALRSIRGELIIIGM	fig|127906.87.peg.1244	VCC1244	VC1593		GGDEF domain protein		
1	CDS	1377022	1376168	-	atgaatcacatccacccctacca	MNHIHPYQNELSDILRKQRITP	fig|127906.87.peg.1245	VCC1245	VC1592		Diguanylate cyclase/phosphodiesterase domain 2 (EAL)	EAL-domain protein	
1	CDS	1378018	1377260	-	atgagaggtttaacccataaagt	MRGLTHKVALVTGAANGIGLA	fig|127906.87.peg.1246	VCC1246	VC1591		Oxidoreductase, short-chain dehydrogenase/reductase family		
1	CDS	1379718	1378015	-	atgcaatccaacattcacttgcg	MQSNIHLRSGAQLIAQQLEAIC	fig|127906.87.peg.1247	VCC1247	VC1590	budB/alsS	Acetolactate synthase, catabolic (EC 2.2.1.6)		
1	CDS	1380642	1379857	-	atgaacccttttactctctgcccac	MNPLLSAHCSCSQEIAQQFAH	fig|127906.87.peg.1248	VCC1248	VC1589	alsD/alsC	Alpha-acetolactate decarboxylase (EC 4.1.1.5)		
1	CDS	1380751	1381629	+	atggaatttcgttacatgaagtac	MEFRYMKYFAAVAEAKHFTRA	fig|127906.87.peg.1249	VCC1249	VC1588	alsR	Transcriptional regulator of alpha-acetolactate operon AlsR		
1	CDS	1382245	1381805	-	atgcacgactcgaaaacgcacc	MHDSKTHNLTTQYGYLIGVLG	fig|127906.87.peg.1250	VCC1250	VC1587		Membrane protein	DUF417 family protein [Vibrio cholerae]	
1	CDS	1382692	1384203	+	atgcatatgtcaaaaagcttttttaa	MHMSKSFLIISMGFVAVSVQA	fig|127906.87.peg.1251	VCC1251	VC1585	katB	Catalase KatE (EC 1.11.1.6)		
1	CDS	1384300	1384833	+	atgaaatggctatggcagctcgc	MKWLWQLALIGLLVLSPFRLS	fig|127906.87.peg.1252	VCC1252	VC1584		AnkB protein		
1	CDS	1384873	1385388	+	atgaatcaatttactttgtttgctgtc	MNQFTLFAVVAFFSSSVLAQE	fig|127906.87.peg.1253	VCC1253	VC1583	sodC	Superoxide dismutase [Cu-Zn] precursor (EC 1.15.1.1)		
1	CDS	1387949	1385433	-	atgaatgcgccacgttcgctctca	MNAPRSLSALSTTANPALSTL	fig|127906.87.peg.1254	VCC1254	VC1582		Hypothetical transmembrane protein coupled to NADH-ubiquinone oxidoreductase chain 5 homolog	YbcC family protein [Vibrio cholerae]	
1	CDS	1389496	1387946	-	atgtcggaactgtcaccccagtg	MSELSPQWLLVSLYLGGLVLS	fig|127906.87.peg.1255	VCC1255	VC1581	nuoL	NADH dehydrogenase, subunit 5		
1	CDS	1389615	1390487	+	atgcgacatctgaattttcaccat	MRHLNFHHLHYFWTVAKEGH	fig|127906.87.peg.1256	VCC1256	VC1580		Transcriptional regulator VC1580, LysR family		
1	CDS	1390551	1392221	+	atgccatacaattctgcgacaga	MPYNSATDLLQREGYGVVHN	fig|127906.87.peg.1257	VCC1257	VC1579	dltA	Enterobactin synthetase component F (EC 2.7.7.-)		
1	CDS	1392205	1392441	+	atgaaacaagccattgaaaata	MKQAIENILIERLQTSIEGISSIL	fig|127906.87.peg.1258	VCC1258	VC1578		hypothetical protein	Lipid A modification system glycine carrier protein AlmF [Vibrio cholerae]	
1	CDS	1392443	1393264	+	gtgcgtattttttggttaaaagcgt	MRIFWLKALTSGLFYLPLSIKN	fig|127906.87.peg.1259	VCC1259	VC1577		hypothetical protein	Lipid A biosynthesis lauroyl acyltransferase [Vibrio cholerae]	
1	CDS	1393420	1393737	+	atgtccatctcaactcaaattacc	MSISTQITFFEFLTPLVASGQK	fig|127906.87.peg.1260	VCC1260	VC1576		RNA-binding domain protein		
1	CDS	1393767	1394225	+	atgtcgaatacacaccaaggct	MSNTHQGLKAVAILESAKGIVS	fig|127906.87.peg.1261	VCC1261	VC1575		Uncharacterized protein XCC0925	DUF2127 domain-containing protein [Vibrio cholerae]	
1	CDS	1394303	1394653	+	atgaatccgattgtggtcagtgtc	MNPIVVSVGLLICSNVFMTFAV	fig|127906.87.peg.1262	VCC1262	VC1574		Uncharacterized membrane protein PA1577	DMT family protein [Vibrio cholerae]	
1	CDS	1396183	1394798	-	atgactcgcgaataccgaattga	MTREYRIEKDSMGEVKVPADA	fig|127906.87.peg.1263	VCC1263	VC1573	fumC	Fumarate hydratase class II (EC 4.2.1.2)		
1	CDS	1396579	1396199	-	atggaaaacagaacgtggttat	MENRTWLYNTFFKAQDRFLAY	fig|127906.87.peg.1264	VCC1264	VC1572		hypothetical protein		
1	repeat	1396695	1396825	+	gtatacccttcctacttgaagctgcagcagtgttggctacgttcgttcaccccaa		fig|127906.87.repeat.24				repeat region		
1	CDS	1397102	1398439	+	atgttagataccctcatgctgtcg	MLDTLMLSRIQFAANISFHILFF	fig|127906.87.peg.1265	VCC1265	VC1571	appC/qxtA	Cytochrome d ubiquinol oxidase subunit I (EC 1.10.3.-)		
1	CDS	1398436	1399428	+	atgatgcagtatttgccagaatt	MMQYLPEIYLLLLGFTVFMYAV	fig|127906.87.peg.1266	VCC1266	VC1570	appB/qxtB	Cytochrome d ubiquinol oxidase subunit II (EC 1.10.3.-)		
1	CDS	1400203	1399502	-	atgaagtctttatctcctgctcctt	MKSLSPAPLVELQQICKHYTS	fig|127906.87.peg.1267	VCC1267	VC1568		ABC-type antimicrobial peptide transport system, ATPase component		
1	CDS	1401480	1400203	-	atgttgccaataagacaaattatg	MLPIRQIMQEMAAEKLRLSLTI	fig|127906.87.peg.1268	VCC1268	VC1567		ABC-type antimicrobial peptide transport system, permease component		
1	CDS	1402698	1401484	-	atgcgcctcttgttacagcaatct	MRLLLQQSWQTLLAHRLKSVL	fig|127906.87.peg.1269	VCC1269	VC1566		ABC-type antimicrobial peptide transport system, permease component		
1	CDS	1403957	1402698	-	atgaataagggctttctcaccgc	MNKGFLTACVLTSLMASSSAF	fig|127906.87.peg.1270	VCC1270	VC1565		Type I secretion outer membrane protein, TolC family		
1	CDS	1405083	1403950	-	atggcaaaacgttggacacttct	MAKRWTLLATLVGVGLAGGY	fig|127906.87.peg.1271	VCC1271	VC1564	macA	Membrane-fusion protein	efflux RND transporter periplasmic adaptor subunit [Vibrio cholerae]	ORF split into 2 in N16961 strain, both ORFs annotated in PATRIC. ORF annotated as 1 pseudogene ORF named VC1563 in KEGG.
									VC1563				ORF split into 2 in N16961 strain, both ORFs annotated in PATRIC. ORF annotated as 1 pseudogene ORF named VC1563 in KEGG.
1	CDS	1406384	1405260	-	atgaaactatccaccttagcttta	MKLSTLALAPITAALLTFNASA	fig|127906.87.peg.1272	VCC1272	VC1562		MBL-fold metallo-hydrolase superfamily		
1	CDS	1406496	1407425	+	atggtcgcccccgaaatcaattt	MVAPEINLRSIDLNLLTILEKLL	fig|127906.87.peg.1273	VCC1273	VC1561	syrM1	Transcriptional regulator, LysR family		
1	CDS	1409664	1407490	-	atggagcacaataaagcgggt	MEHNKAGSSGQCPVMHGGL	fig|127906.87.peg.1274	VCC1274	VC1560	katG	Catalase-peroxidase KatG (EC 1.11.1.21)		
1	CDS	1410077	1411513	+	atggctaagtctatttttcctaacg	MAKSIFPNDFLWGGAVAAHQ	fig|127906.87.peg.1275	VCC1275	VC1558	bglA	6-phospho-beta-glucosidase (EC 3.2.1.86)		
1	CDS	1411576	1412583	+	atggcaaccataaacgatgtgtc	MATINDVCKLAGVSKATVSRV	fig|127906.87.peg.1276	VCC1276	VC1557		Transcriptional regulator, LacI family		
1	CDS	1412718	1413080	+	atgacaatacatcgtattaatcca	MTIHRINPGTRWSDITVFNGIA	fig|127906.87.peg.1277	VCC1277	VC1556	rutC	RidA/YER057c/UK114 superfamily, group 2, YoaB-like protein		
1	CDS	1413091	1413561	+	atgtttattggtcatatcacgcagc	MFIGHITQRQFCAALSPQLQR	fig|127906.87.peg.1278	VCC1278	VC1555	tabA	hypothetical protein	DUF386 domain-containing protein [Vibrio cholerae]	
1	CDS	1414347	1413637	-	atgccttcaatcatcgtcggccac	MPSIIVGHRGVAGTYPENTRV	fig|127906.87.peg.1279	VCC1279	VC1554	ugpQ	Glycerophosphoryl diester phosphodiesterase (EC 3.1.4.46)		
1	CDS	1415139	1414378	-	gtgactttattatcccaccgccaa	MTLLSHRQKQILEYLQQTHGV	fig|127906.87.peg.1280	VCC1280	VC1553	glpR	Glycerol-3-phosphate regulon repressor GlpR		
1	CDS	1416345	1415164	-	gtgaaaaataatcagaactcca	MKNNQNSNIHLLRSAKHAEPV	fig|127906.87.peg.1281	VCC1281	VC1552	ugpC	Glycerol-3-phosphate ABC transporter, ATP-binding protein UgpC (TC 3.A.1.1.3)		
1	CDS	1417177	1416335	-	atgaaaagtaatcgactctccga	MKSNRLSDHLILIAGALFMLVP	fig|127906.87.peg.1282	VCC1282	VC1551	ugpE	Glycerol-3-phosphate ABC transporter, permease protein UgpE (TC 3.A.1.1.3)		
1	CDS	1418068	1417187	-	gtgcaacgtcgacacatttcgc	MQRRQHFAHTPLPYLLLAPQI	fig|127906.87.peg.1283	VCC1283	VC1550	ugpA	Glycerol-3-phosphate ABC transporter, permease protein UgpA (TC 3.A.1.1.3)		
1	CDS	1419448	1418144	-	atgaataaggtatggttaaccgg	MNKVWLTGLLTATASGVFSAS	fig|127906.87.peg.1284	VCC1284	VC1549	ugpB	Glycerol-3-phosphate ABC transporter, substrate-binding protein UgpB		
1	CDS	1419767	1420525	+	atgaaaagatgtctctcacttcttt	MKRCLSLLCVSALLPAHAASL	fig|127906.87.peg.1285	VCC1285	VC1548		TonB system biopolymer transport component; Chromosome segregation ATPase		
1	CDS	1420525	1421898	+	atgacacttaaaaagaccacct	MTLKKTTLALALSLPFAFASVA	fig|127906.87.peg.1286	VCC1286	VC1547		MotA/TolQ/ExbB proton channel family protein		
1	CDS	1421908	1422447	+	atgaattcagcattctctttcctac	MNSAFSFLLDYWQPLDEFMA	fig|127906.87.peg.1287	VCC1287	VC1546		Ferric siderophore transport system, biopolymer transport protein ExbB		
1	CDS	1422444	1422848	+	atgaggctagggcgcagaacg	MRLGRRTSKQEEAQIDLTSML	fig|127906.87.peg.1288	VCC1288	VC1545	exbD	Biopolymer transport protein ExbD/TolR		
1	CDS	1422848	1423468	+	atgggacgcttgatcttagccag	MGRLILASPIALLVTLALFSLMA	fig|127906.87.peg.1289	VCC1289	VC1544		putative TonB-dependent receptor		
1	CDS	1423465	1424643	+	atgaaacgacgaatcttaagtta	MKRRILSYLLLSCLSVTTYAAE	fig|127906.87.peg.1290	VCC1290	VC1543		TPR domain protein, putative component of TonB system		
1	repeat	1424663	1424792	+	atataccttcctacttgaagttgcagcggtgttggctacgttcgttcaccccaat		fig|127906.87.repeat.25				repeat region		
1	CDS	1424950	1425798	+	atgcttatacgactgacgctcac	MLIRLTLTAAAIMLAQQTLAND	fig|127906.87.peg.1291	VCC1291	VC1542	ligA_2	DNA ligase (ATP) (EC 6.5.1.1)		
1	CDS	1426788	1425805	-	atgcgtttaagcgttgtactggctc	MRLSVVLAALLLLTGCEPRLAI	fig|127906.87.peg.1292	VCC1292	VC1541		hypothetical protein	DUF3080 domain-containing protein [Vibrio cholerae]	
1	CDS	1428365	1426992	-	gtgcatcgttacaaaaaagaag	MHRYKKEASNLIKLATPVLIAS	fig|127906.87.peg.1293	VCC1293	VC1540		Multidrug efflux transporter MdtK/NorM (MATE family)		
1	CDS	1428823	1428620	-	atgagaaacttcacggaaaagt	MRNFTEKSVTCPHCGHGIRLT	fig|127906.87.peg.1294	VCC1294	No refSeq annotation		Uncharacterized protein VC1539.5	CPXCG motif-containing cysteine-rich protein [Vibrio cholerae]	
1	CDS	1429847	1428981	-	atgtggccagcaattattcaaca	MWPAIIQQLSEQLGKDFHLVE	fig|127906.87.peg.1295	VCC1295	VC1539		Ribulosamine/erythrulosamine 3-kinase potentially involved in protein deglycation		
1	CDS	1430370	1430711	+	gtggttgtagaaaccgatggata	MVVETDGYLALIEHLSFNMDV	fig|127906.87.peg.1296	VCC1296	VC1538		Type IIA topoisomerase, B subunit		
1	CDS	1431221	1430721	-	atgttgacggtcaagcaaacgtt	MLTVKQTFSLLILGFLVGCTAT	fig|127906.87.peg.1297	VCC1297	VC1537	nlpC	Lipoprotein NlpC		
									VC1536				ORF split into 2 in N16961 strain, both ORFs annotated in PATRIC. ORF annotated as 1 pseudogene ORF named VC1535 in KEGG.
1	CDS	1431310	1433331	+	atggctttgacaaaaaatttatcc	MALTKNLSLTQTLGAVFLCITIL	fig|127906.87.peg.1298	VCC1298	VC1535		Methyl-accepting chemotaxis sensor/transducer protein		ORF split into 2 in N16961 strain, both ORFs annotated in PATRIC. ORF annotated as 1 pseudogene ORF named VC1535 in KEGG.
1	CDS	1434124	1433333	-	atgagtgaacaaattgagctgat	MSEQIELIRLAIFFSVLVICALW	fig|127906.87.peg.1299	VCC1299	VC1534		Fatty acid hydroxylase family (carotene hydroxylase/sterol desaturase)		
1	CDS	1434224	1434961	+	atgagaattcacgcgtttcatcac	MRIHAFHHLYQYRLERSTKPF	fig|127906.87.peg.1300	VCC1300	VC1533		Uncharacterized conserved protein YfiP, contains DTW domain		
1	CDS	1435124	1436032	+	atgtattacggctttgatgtgggc	MYYGFDVGGTKIEFGAFNEQL	fig|127906.87.peg.1301	VCC1301	VC1532		N-acetyl-D-glucosamine kinase (EC 2.7.1.59)		
1	repeat	1436080	1436204	+	tccccttcctacttgaagttgcagcggtgttggctgcgttcattcaccccaatca		fig|127906.87.repeat.26				repeat region		
1	CDS	1436514	1436239	-	atggctcgtacaattatttacacc	MARTIIYTYKNQEKTLTFSYEK	fig|127906.87.peg.1302	VCC1302	VC1531		hypothetical protein	DUF2960 domain-containing protein [Vibrio cholerae]	
1	CDS	1436558	1436719	+	atgactgcgatgatagcacaag	MTAMIAQAKSGIAAIFSTFFAM	fig|127906.87.peg.1303	VCC1303	VC1530		FIG01200934: hypothetical protein		
1	CDS	1436730	1437155	+	atgaactatttcattcgtcagtcttc	MNYFIRQSWLFFFTVTSSFHV	fig|127906.87.peg.1304	VCC1304	VC1529		ABC-type amino acid transport/signal transduction system, periplasmic component/domain		Limited query cover for certain strains
1	CDS	1437152	1437508	+	ttgaataccgaacaacaagtcg	MNTEQQVGAHLGFSIVNELKK	fig|127906.87.peg.1305	VCC1305	VC1528		ABC-type amino acid transport/signal transduction system, periplasmic component/domain		
1	CDS	1439418	1437598	-	atgattgcttctgcacaccagaa	MIASAHQNPQRPNLPLLGFAA	fig|127906.87.peg.1306	VCC1306	VC1527		Molybdopterin-guanine dinucleotide biosynthesis protein MobB / Molybdopterin molybdenumtransferase (EC 2.10.1.1)		
1	CDS	1440026	1439415	-	atgataaccacacaaacacttc	MITTQTLPTLQPSDTSWVILAG	fig|127906.87.peg.1307	VCC1307	VC1526	mobA	Molybdenum cofactor guanylyltransferase (EC 2.7.7.77)		VCC1307 and VCC1308 partly overlaps
1	CDS	1440702	1439980	-	atgacgatcaaacttaccgcac	MTIKLTAQQLSMRFKDRVLFH	fig|127906.87.peg.1308	VCC1308	VC1525	ecfA3	Tungstate ABC transporter, ATP-binding protein		VCC1307 and VCC1308 partly overlaps
1	CDS	1441409	1440711	-	atgagtctttggcaaacaaccct	MSLWQTTLDALHLLVSLDPKL	fig|127906.87.peg.1309	VCC1309	VC1524		Tungstate ABC transporter, permease protein		
1	CDS	1442231	1441416	-	atgaaaaaaacaacctttaccc	MKKTTFTLAAASLILVSYSASS	fig|127906.87.peg.1310	VCC1310	VC1523		Tungstate ABC transporter, substrate-binding protein		
1	CDS	1442535	1443989	+	atgtcaccagttttggatctcaatc	MSPVLDLNQTHLYQAFSVLLV	fig|127906.87.peg.1311	VCC1311	VC1522		Sigma-54 dependent response regulator		
1	CDS	1443979	1446012	+	atgaaagctaaatgggcttggtg	MKAKWAWWQKWRHRFQTTV	fig|127906.87.peg.1312	VCC1312	VC1521		Two-component system sensor histidine kinase		
1	CDS	1446224	1447816	+	ttgatttctacagcgaatatcactc	MISTANITQQFGAKPLFENISV	fig|127906.87.peg.1313	VCC1313	VC1520		Bis-ABC ATPase YbiT		
1	CDS	1448756	1447923	-	gtggctaaaccttccatcatcaa	MAKPSIIKTSENPLQTIEVEVY	fig|127906.87.peg.1314	VCC1314	VC1519	fdhD	Sulfur carrier protein FdhD		
1	CDS	1448981	1449457	+	atgactgaacaaacttctcctga	MTEQTSPESLTDGAHSKTDSA	fig|127906.87.peg.1315	VCC1315	VC1518		hypothetical protein	DUF3305 domain-containing protein [Vibrio cholerae]	
1	CDS	1449447	1450049	+	gtggcaactgaaaacaggttttt	MATENRFFSRWSQRKLTAES	fig|127906.87.peg.1316	VCC1316	VC1517		hypothetical protein	DUF3306 domain-containing protein [Vibrio cholerae]	
1	CDS	1450248	1451909	+	atgttaaacaacctactacaaca	MLNNLLQQATSPNGRARQYA	fig|127906.87.peg.1317	VCC1317	VC1516		Iron-sulfur cluster-binding protein		
1	CDS	1451920	1452591	+	atgcaagagcaatctatgcgttc	MQEQSMRSQSTHSQSMRSD	fig|127906.87.peg.1318	VCC1318	VC1515		Putative formate dehydrogenase-specific chaperone		
1	CDS	1452669	1452866	+	atgaaaaaagagaaccaagta	MKKENQVNQSRRDLLKGLGT	fig|127906.87.peg.1319	VCC1319	VC1514		Formate dehydrogenase subunit or accessory protein		
1	CDS	1452878	1455733	+	atgagactcatcaaacgttcaga	MRLIKRSDSVTKEQNQLGISR	fig|127906.87.peg.1320	VCC1320	VC1513	fdhF	Formate dehydrogenase-O, major subunit (EC 1.2.1.2)		ORF split into 2 ORFs in N16961, both lacking refseq in PATRIC. Annotated as 1 pseudogene ORF named VC1513 in KEGG.
1	CDS	1455745	1456353	+	atggcaagaatgaaattcctgtg	MARMKFLCDTKRCIECNGCV	fig|127906.87.peg.1321	VCC1321	VC1512	fdhB	Formate dehydrogenase-O, iron-sulfur subunit (EC 1.2.1.2); Putative formate dehydrogenase iron-sulfur subunit (EC 1.2.1.2)		
1	CDS	1456360	1457427	+	atgagacaacaactttctcgcgc	MRQQLSRATRFVLPLLAALLC	fig|127906.87.peg.1322	VCC1322	VC1511	fdnI	Formate dehydrogenase -O, gamma subunit (EC 1.2.1.2)		
1	CDS	1457414	1457836	+	atgaatcaagatagagcgcaca	MNQDRAHKSVWFVYIASLLTP	fig|127906.87.peg.1323	VCC1323	VC1510		hypothetical protein		
1	CDS	1457973	1458713	+	atgagcttaccgtatcgacatgtt	MSLPYRHVVILTGAGISAESGI	fig|127906.87.peg.1324	VCC1324	VC1509	cobB	NAD-dependent protein deacetylase of SIR2 family		
1	CDS	1459330	1458848	-	atgccttcttttgatattgtttcagaa	MPSFDIVSEIDAVELRNAVENS	fig|127906.87.peg.1325	VCC1325	VC1508		UPF0234 protein Yitk		
1	CDS	1460403	1459342	-	atgccactgaaaaccgatgaat	MPLKTDELRTQALGPMPTPAE	fig|127906.87.peg.1326	VCC1326	VC1507		2-keto-3-deoxy-D-arabino-heptulosonate-7-phosphate synthase I alpha (EC 2.5.1.54)		
1	CDS	1460679	1461155	+	atgctgtcacccttagcaaaagc	MLSPLAKANLCLNLFGFFKVPI	fig|127906.87.peg.1327	VCC1327	VC1506		hypothetical protein	DUF4442 domain-containing protein [Vibrio cholerae]	
1	CDS	1462497	1461148	-	ttgattaccgtacaacagcaaga	MITVQQQDAATLTIVSQMTGS	fig|127906.87.peg.1328	VCC1328	VC1505		Uncharacterized protein SO_0942		ORF split into 2 ORFs in N16961, both with refseq in PATRIC. Annotated as 1 pseudogene ORF named VC1505 in KEGG.
									VC1504				ORF split into 2 ORFs in N16961, both with refseq in PATRIC. Annotated as 1 pseudogene ORF named VC1505 in KEGG.
1	CDS	1462599	1463432	+	atgattaacattggtcaaattaat	MINIGQINSLEVVKFAEFGVFLI	fig|127906.87.peg.1329	VCC1329	VC1503	cvfB	hypothetical protein	S1 RNA-binding domain-containing protein [Vibrio cholerae]	
1	CDS	1465035	1463614	-	gtgcatccgaacatttcccttccc	MHPNISLPEPFIASMAKILPDP	fig|127906.87.peg.1330	VCC1330	VC1502	yebU	16S rRNA (cytosine(1407)-C(5))-methyltransferase (EC 2.1.1.178)		
1	CDS	1467795	1465156	-	atgagtcaagaaaatacaacgc	MSQENTTQTSYTPEIRKRRGIS	fig|127906.87.peg.1331	VCC1331	VC1501		Uncharacterized protein YebT	PqiB family protein [Vibrio cholerae]	
1	CDS	1469049	1467805	-	atgacctcctcatcctctttatctca	MTSSSSLSHHASQSGQVRLC	fig|127906.87.peg.1332	VCC1332	VC1500	yebS	Inner membrane protein YebS	Paraquat-inducible protein A [Vibrio cholerae]	
1	CDS	1470894	1469098	-	atgacgtatttcaacgacactatt	MTYFNDTISRSWLITQVKKHRA	fig|127906.87.peg.1333	VCC1333	VC1499		Efflux ABC transporter, permease/ATP-binding protein		
1	CDS	1470905	1471453	+	atgcatcctattttagtgatgcgca	MHPILVMRIVRSSRSEVFIFQM	fig|127906.87.peg.1334	VCC1334	VC1498	msrC	Free methionine-(R)-sulfoxide reductase, contains GAF domain		Limited query cover for certain strains
1	CDS	1471554	1472180	+	atggaaaacactgaaaagttaa	MENTEKLKNSKEVIAYIAECFP	fig|127906.87.peg.1335	VCC1335	VC1497	proQ	RNA chaperone ProQ		
1	CDS	1472198	1474195	+	atgaaatgccgttcaaaaatatc	MKCRSKISLIAASLWLAAFSAC	fig|127906.87.peg.1336	VCC1336	VC1496	prc	Tail-specific protease precursor (EC 3.4.21.102)		
1	CDS	1474349	1475020	+	atgaaatggatatttacttgcgtga	MKWIFTCVMSLLFTASSPFLSA	fig|127906.87.peg.1337	VCC1337	VC1495		FOG: WD40 repeat		
1	CDS	1475219	1476181	+	atggctcacacacctcaagcca	MAHTPQAKYRLDYQPPSHTIT	fig|127906.87.peg.1338	VCC1338	VC1494	pepN	Membrane alanine aminopeptidase N (EC 3.4.11.2)		Limited query/subject cover for certain strains
1	CDS	1476345	1477793	+	atgagtcgtcaagaccctcaag	MSRQDPQESVYLMLDIIRAGV	fig|127906.87.peg.1339	VCC1339	Not present		putative site-specific recombinase	Recombinase/integrase	
1	CDS	1477853	1478164	+	atgaatagcacagataaacgac	MNSTDKRLFDFVLKVATNTYIC	fig|127906.87.peg.1340	VCC1340	Not present		hypothetical protein		
1	CDS	1478202	1478852	+	atgatttcaacagttactatcgaa	MISTVTIEVSPEEAHLHLLQRQ	fig|127906.87.peg.1341	VCC1341	Not present		hypothetical protein		
1	CDS	1478951	1481227	+	gtgaaacatattgataccttttga	MKHIDTFLKLSPKAAARINKHA	fig|127906.87.peg.1342	VCC1342	Not present		hypothetical protein	DNA-directed RNA polymerase [Vibrio cholerae]	
1	CDS	1481555	1482490	+	atgactaaaactactattactgttt	MTKTTITVSIADITFDRAFQFRT	fig|127906.87.peg.1343	VCC1343	Not present		hypothetical protein		
1	CDS	1482596	1483135	+	atgatttccaaaaatgaccttggc	MISKNDLGKPPKYDRALKVLK	fig|127906.87.peg.1344	VCC1344	Not present		FIG01200969: hypothetical protein	resolvase [Vibrio cholerae]	
1	CDS	1483277	1483864	+	atggcatttaagattgtttacgcaa	MAFKIVYAKKELAGIELTGYVN	fig|127906.87.peg.1345	VCC1345	Not present		FIG01203606: hypothetical protein	single-stranded DNA-binding protein	
1	CDS	1483865	1484224	+	ttgattgaccgcttcagtgtgcgg	MIDRFSVRIKPLSTNEMHLGRI	fig|127906.87.peg.1346	VCC1346	Not present		hypothetical protein	RusA family crossover junction endodeoxyribonuclease [Vibrio cholerae]	
1	CDS	1484237	1485871	+	atggctcaagaaagcaatttctta	MAQESNFLHKEPCPKCGSKD	fig|127906.87.peg.1347	VCC1347	Not present		Phage primase/helicase protein Gp4A		
1	CDS	1485871	1487925	+	atgatgcaagaaaggctctgtta	MMQERLCYDIETDGLIEDVTR	fig|127906.87.peg.1348	VCC1348	Not present		Phage DNA-directed DNA polymerase (EC 2.7.7.7)		
1	CDS	1487950	1488381	+	atgtttggtctattcaagaaaaaa	MFGLFKKKAKAAQVTLHKVEN	fig|127906.87.peg.1349	VCC1349	Not present		hypothetical protein	tellurite resistance TerB family protein [Vibrio cholerae]	
1	CDS	1488599	1489435	+	ttgtttgaccttgatattttgccttcc	MFDLDIFAFQANATAMEEVFL	fig|127906.87.peg.1350	VCC1350	Not present		phage exonuclease, putative		
1	CDS	1489618	1489902	+	gtgcctcttaaagacgctgaacg	MPLKDAERIIQHYEEGKENMS	fig|127906.87.peg.1351	VCC1351	Not present		hypothetical protein		
1	CDS	1489899	1490156	+	atgaaaatgtcacttatcttgagc	MKMSLILSKVLKFIIRALVKEAA	fig|127906.87.peg.1352	VCC1352	Not present		hypothetical protein		
1	CDS	1490156	1490278	+	atgcgcccgtctcgcctacgtaa	MRPSRLRKRERFDDYEPEDN	fig|127906.87.peg.1353	VCC1353	Not present		hypothetical protein		
1	CDS	1490289	1490882	+	atgtacatcgctatcgagggaaa	MYIAIEGNIGAGKSTVLEPLAA	fig|127906.87.peg.1354	VCC1354	Not present	dck	2-amino-4-hydroxy-6-hydroxymethyldihydropteridine pyrophosphokinase (EC 2.7.6.3) / Deoxyadenosine kinase (EC 2.7.1.76) @ Deoxyguanosine kinase (EC 2.7.1.113)		
1	CDS	1491087	1491356	+	atgattttcaaagttcatacccctag	MIFKVHTLVKLIVNRKLEGFKIM	fig|127906.87.peg.1355	VCC1355	Not present		hypothetical protein		
1	CDS	1491376	1491963	+	atgccttacattaccctagaagg	MPYITLEGNVGVGKSTLLHRL	fig|127906.87.peg.1356	VCC1356	Not present		ORF5	AAA family ATPase [Vibrio cholerae]	
1	CDS	1492057	1492893	+	gtgaaaaaggctatttcttcaggt	MKKAISSGAYTLLMLLLAVSVF	fig|127906.87.peg.1357	VCC1357	Not present		hypothetical protein		
1	CDS	1492991	1493254	+	atgaacatttcattacgtgacctc	MNISLRDLLPVVIVPIVTWIMST	fig|127906.87.peg.1358	VCC1358	Not present		hypothetical protein		
1	CDS	1493291	1493443	+	atgcaagtaaagaacaagaaa	MQVKNKKGEIFTVSQEHYETY	fig|127906.87.peg.1359	VCC1359	Not present		hypothetical protein		
1	CDS	1494332	1493895	-	atgttcgactccatagattttgaat	MFDSIDFESVTIGSRRSSANE	fig|127906.87.peg.1360	VCC1360	Not present		hypothetical protein		
1	CDS	1495039	1495455	+	atgcgtaaagtctgtgcatctgct	MRKVCASAGCKSVTSSRYCS	fig|127906.87.peg.1361	VCC1361	Not present		hypothetical protein	HNH endonuclease [Vibrio cholerae]	
1	CDS	1496048	1495932	-	atggttgtatttaacctcgatgtac	MVVFNLDVLTEVALYRDLEVR	fig|127906.87.peg.1362	VCC1362	Not present		hypothetical protein		

1	CDS	1496254	1496048	-	atgaacgttgaaaaactagctca	MNVEKLAQAIEKANAGKQAFV	fig|127906.87.peg.1363	VCC1363	Not present		hypothetical protein		
1	CDS	1496562	1496350	-	atgaaactaaacagcgcacag	MKLNSAQTKRLVKALYDDEVT	fig|127906.87.peg.1364	VCC1364	Not present		hypothetical protein		
1	CDS	1497263	1496619	-	atgaaaacttcagaaatcatcaa	MKTSEIIKALETSAKAMNESTI(	fig|127906.87.peg.1365	VCC1365	Not present		hypothetical protein	MULTISPECIES: antirestriction protein [Vibrio]	
1	CDS	1497474	1497662	+	atggtgctgaatgtcgaactattg	MVLNVELLDGATVEYKDVFYL	fig|127906.87.peg.1366	VCC1366	Not present		FIG01207287: hypothetical protein		
1	CDS	1498037	1498543	+	atgggtagacctagtaagtctatt	MGRPSKSIAELKANGTYRPNK	fig|127906.87.peg.1367	VCC1367	Not present		hypothetical protein	phage terminase small subunit P27 family [Vibrio cholerae]	
1	CDS	1498524	1499129	+	atggagggcaagctcattaacta	MEGKLINYYTLLGLYVLASVAS	fig|127906.87.peg.1368	VCC1368	Not present		hypothetical protein		
1	CDS	1499126	1499806	+	atgaacaaatatctagttttggca	MNKYLVLALATVATFFAVGVK	fig|127906.87.peg.1369	VCC1369	Not present		hypothetical protein		
1	CDS	1499834	1500049	+	gtgacaaagcctaagaaattcc	MTKPKKFLINTIVDAQPFSSGE	fig|127906.87.peg.1370	VCC1370	Not present		hypothetical protein		
1	CDS	1500049	1500231	+	atgacactttccggtttggtggtc	MTLSGLVVELHPELDPSEIRHI	fig|127906.87.peg.1371	VCC1371	Not present		hypothetical protein		
1	CDS	1500240	1502000	+	atgaacccaactaaggctcaaa	MNPTKAQIERVRKELVNHNLT	fig|127906.87.peg.1372	VCC1372	Not present		Phage terminase, large subunit		
1	CDS	1502010	1503335	+	atgggtttattgaactggctctttaa	MGLLNWLFKSSKEPQSKSINP	fig|127906.87.peg.1373	VCC1373	Not present		Phage portal protein		
1	CDS	1503298	1504146	+	atgctgagaaagagcaaggtaa	MLRKSKVKTLQFDVQGVSNE(	fig|127906.87.peg.1374	VCC1374	Not present		hypothetical protein	HK97 family phage prohead protease [Vibrio cholerae]	
1	CDS	1504245	1505435	+	atgaaatttgacattaaatctatcc	MKFDIKSIEAITNTAEMKQVMT	fig|127906.87.peg.1375	VCC1375	Not present		hypothetical protein	phage major capsid protein [Vibrio cholerae]	
1	CDS	1505450	1506001	+	atgagtcacgttattcttagccgc	MSHVILSRAFTEVVPFEQVASI	fig|127906.87.peg.1376	VCC1376	Not present		TonB-dependent receptor		
1	CDS	1506013	1506360	+	atgagacgtggtgctttgcgtcac	MRRGALRHRLQVYAPEAYLF(	fig|127906.87.peg.1377	VCC1377	Not present		hypothetical protein	head-tail adaptor protein [Vibrio parahaemolyticus]	
1	CDS	1506347	1506958	+	atgtcgaagtatgacttggtttcttt	MSKYDLVSFELEFNGRVDLAC	fig|127906.87.peg.1378	VCC1378	Not present		hypothetical protein		
1	CDS	1506961	1507329	+	gtggaattaacacttattgaattcc	MELTLIEFLKPHLGGMQGYIAK	fig|127906.87.peg.1379	VCC1379	Not present		hypothetical protein		
1	CDS	1507343	1507813	+	atggcaggtatcggtcgtggtac	MAGIGRGTRGIDTKLLYNLTA(	fig|127906.87.peg.1380	VCC1380	Not present		FIG01202693: hypothetical protein		
1	CDS	1507866	1508201	+	atgaatcgcaatgaactactaaa	MNRNELLKRLMNKPVKFTVD(	fig|127906.87.peg.1381	VCC1381	Not present		FIG01206989: hypothetical protein		
1	CDS	1508246	1508536	+	atgctttattggggattgacctttga	MLYWGLTFEEFNKQPMALIYR	fig|127906.87.peg.1382	VCC1382	Not present		hypothetical protein		
1	CDS	1508553	1510865	+	atggcaggaaagaacgttacga	MAGKNVTIGNVNIAMSANAAK	fig|127906.87.peg.1383	VCC1383	Not present		hypothetical protein	phage tail tape measure protein [Vibrio parahaemolyticus]	
1	CDS	1510867	1511448	+	atggttcaggttttacctagccaa	MVQVLPSQLKLTEVSITNYHRY	fig|127906.87.peg.1384	VCC1384	Not present		hypothetical protein		
1	CDS	1511459	1511998	+	atggcttcaacacaaattctatta	MASTQILLLELRYKDQSTGEV	fig|127906.87.peg.1385	VCC1385	Not present		hypothetical protein		
1	CDS	1512001	1512396	+	atgttcagtaaagcagaagaag	MFSKAEEVIREFRGQEHVRG(	fig|127906.87.peg.1386	VCC1386	Not present		hypothetical protein		
1	CDS	1512400	1518342	+	atgggtttagctgcggctactgttg	MGLAAATVAWISVGLSVGATI	fig|127906.87.peg.1387	VCC1387	Not present		hypothetical protein		
1	CDS	1518343	1518708	+	atgaatgaaccgtactttcctcac	MNEPYFPHVLGEERTVYFLKS	fig|127906.87.peg.1388	VCC1388	Not present		hypothetical protein		
1	CDS	1518725	1519732	+	atgtattaccacaatatcgcgtgg	MYYHNIAWNIQTTNDCFLDCA	fig|127906.87.peg.1389	VCC1389	Not present		hypothetical protein	radical SAM protein [Vibrio cholerae]	
1	CDS	1519732	1520289	+	atggctgtcttaatgcgtcctgtcc	MAVLMRPVRLFLEAQEARGLK	fig|127906.87.peg.1390	VCC1390	Not present		hypothetical protein		
1	CDS	1520363	1520638	+	atggataaagtccagcttatccg	MDKVQLIRQIIGVPLPVSSAYR	fig|127906.87.peg.1391	VCC1391	Not present		hypothetical protein	peptidase M15 [Vibrio cholerae]	
1	CDS	1521124	1520702	-	atggattttaatgttcatacaactg	MDFNVHTTVSKDPIIVLKEKKS	fig|127906.87.peg.1392	VCC1392	Not present		hypothetical protein		
1	CDS	1522391	1521126	-	atggctgaaactctaaaggtaaa	MAETLKVKNFLVIKNAEIDIKRI	fig|127906.87.peg.1393	VCC1393	Not present		hypothetical protein	ATP-binding protein [Vibrio cholerae]	
1	CDS	1523220	1522708	-	atgcgtctaactcaactaacccc	MRLTQLTPSEAAVFEQIQFLDI	fig|127906.87.peg.1394	VCC1394	Not present		FIG01203269: hypothetical protein		
1	CDS	1523626	1523501	-	gtgggttgcttgttttttttgctcaat	MGCLFFFAQYWLWFVTNNSL	fig|127906.87.peg.1395	VCC1395	Not present		hypothetical protein		
1	CDS	1523829	1524125	+	atgttaaataacaacgtaacgga	MLNNNVTELVTEIVLPQCDDS	fig|127906.87.peg.1396	VCC1396	Not present		hypothetical protein		
1	CDS	1524169	1524405	+	atggtagtggctcaaagagaag	MVVAQREGLAAGDLVDALDM	fig|127906.87.peg.1397	VCC1397	Not present		hypothetical protein	ArsR family transcriptional regulator [Vibrio cholerae]	
1	CDS	1524478	1527102	+	atggactcaaataaacctgtagc	MDSNKPVAILREDYKPSDYLID	fig|127906.87.peg.1398	VCC1398	VC1494		Membrane alanine aminopeptidase N (EC 3.4.11.2)		Limited query/subject cover for certain strains.
1	CDS	1527232	1527372	+	atggtcaccaccgagcaaggtt	MVTTEQGLRLQLPATRLRPFL	fig|127906.87.peg.1399	VCC1399	VC1493		hypothetical protein		
										gdhB	NAD-specific glutamate dehydrogenase (EC 1.4.1.2), large form		
1	CDS	1527561	1532402	+	atgactgcgcgtgaaactttgatc	MTARETLMPVLLEKVYQLIQDI	fig|127906.87.peg.1400	VCC1400	VC1492	gdhB	NAD-specific glutamate dehydrogenase (EC 1.4.1.2), large form		
1	CDS	1532476	1533486	+	atgctttaccgcttagccagagct	MLYRLARAGFFQLDAEKAHDL	fig|127906.87.peg.1401	VCC1401	VC1491	pyrD	Dihydroorotate dehydrogenase (quinone) (EC 1.3.5.2)		
1	CDS	1533641	1534183	+	atgcttaaaccaagtgacaaatg	MLKPSDKWSWYFSDSEGYLM	fig|127906.87.peg.1402	VCC1402	VC1490	zapC	Cell division protein ZapC		
1	CDS	1534643	1536769	+	atgaatcagtatctagcggtgac	MNQYLAVTSNGLENLLVEELT	fig|127906.87.peg.1403	VCC1403	VC1488	rimL	23S rRNA (guanine(2445)-N(2))-methyltransferase (EC 2.1.1.173) / 23S rRNA (guanine(2069)-N(7))-methyltransferase (EC 2.1.1.264)		
1	CDS	1536778	1537020	+	atgataaccttgtacagcactga	MITLYSTEGCHLCEMAYALLNI	fig|127906.87.peg.1404	VCC1404	VC1487		Glutaredoxin-like domain-containing protein PA3033		Limited query cover for certain strains
1	CDS	1536998	1538917	+	atggcattaattaccatccataac	MALITIHNGLLAFGDHPLLDHA	fig|127906.87.peg.1405	VCC1405	VC1486	uup	Bis-ABC ATPase Uup		
1	CDS	1538923	1540617	+	atgagtcgtattactttaaacgttt	MSRITFKRSILASAVLLATQTAI	fig|127906.87.peg.1406	VCC1406	VC1485		hypothetical protein	DUF3466 family protein [Vibrio cholerae]	
1	CDS	1540785	1540958	+	atgaagagacaaaagcgtgat	MKRQKRDRLERAQSQGYKAG	fig|127906.87.peg.1407	VCC1407	VC1484		Ribosome modulation factor		Limited query cover for certain strains
1	CDS	1541559	1541041	-	atgcagaacaaacgtgattcgta	MQNKRDSYNREDLLASSQGE	fig|127906.87.peg.1408	VCC1408	VC1483	fabA	3-hydroxyacyl-[acyl-carrier-protein] dehydratase, FabA form (EC 4.2.1.59) @ Trans-2-decenoyl-[acyl-carrier-protein] isomerase (EC 5.3.3.14)		
1	CDS	1543273	1541624	-	atgacccaaagcatctggcgca	MTQSIWRSVTPQFEQFNKVFE	fig|127906.87.peg.1409	VCC1409	VC1482		Lon protease homolog YcbZ		Limited query cover for certain strains
1	CDS	1543437	1543886	+	atgaaatatcaacaacttgaaaa	MKYQQLENLECGWKWQYLIN	fig|127906.87.peg.1410	VCC1410	VC1481	matP	Macrodomain Ter protein YcbG		
1	CDS	1543900	1544223	+	atgaatatgctttatgtaatacttgt	MNMLYVILVAAIIIFWLVAIDRP	fig|127906.87.peg.1411	VCC1411	VC1480		hypothetical protein	DUF3634 family protein [Vibrio cholerae]	
1	CDS	1544382	1544693	+	atgttgagcattttcctcacaatttt	MLSIFLTIFSQSTLADDADLNR	fig|127906.87.peg.1412	VCC1412	VC1479		hypothetical protein		
1	repeat	1545593	1546866	+	ttgtagtggtcaactaaaattggccacggttttagagttttcccaatatataatcgtt		fig|127906.87.repeat.27				repeat region		
1	CDS	1546474	1545602	-	ttgataatcaagaaactcaagca	MIIKKLKQSHSVKILCEVFNVHI	fig|127906.87.peg.1413	VCC1413	VC1478		Transposase InsO for insertion sequence element IS911		
1	CDS	1546788	1546471	-	atgacaaaacgtacaaaacgta	MTKRTKRTRRLFSAEFKLEAA	fig|127906.87.peg.1414	VCC1414	VC1477		Transposase InsN for insertion sequence element IS911		Limited query cover for N16961
1	CDS	1547699	1547133	-	atgcgagtattaagaatgtcaga	MRVLRMSEVSIADRIKKAIIDN(	fig|127906.87.peg.1415	VCC1415	VC1476		Phage protein		
1	CDS	1547861	1549429	+	atgatagacatgttccacataaa	MIDMFHINIPFKKDVIIEMGDC(	fig|127906.87.peg.1416	VCC1416	VC1475		phage replication protein Cri		
1	CDS	1549434	1549718	+	atgaaagttgtatacatgggcatt	MKVVYMGISHRKGISNKGLGK	fig|127906.87.peg.1417	VCC1417	VC1474		hypothetical protein		
1	CDS	1549848	1550540	+	atgaacttgaatcaactcacggg	MNLNQLTGADLMAARTHIGLS	fig|127906.87.peg.1418	VCC1418	VC1473		hypothetical protein		
1	CDS	1550618	1550785	+	atgcagggacatcatacaagag	MQGHHTREDAKTTEATEQRL	fig|127906.87.peg.1419	VCC1419	VC1472		hypothetical protein		
1	CDS	1550778	1550945	+	atgactaaatacgtgcggctcaa	MTKYVRLNNVGAAWIKSTREK	fig|127906.87.peg.1420	VCC1420	VC1471		hypothetical protein		
1	CDS	1552550	1551984	-	atgcgagtattaagaatgtcaga	MRVLRMSEVSIADRIKKAIIDN(	fig|127906.87.peg.1421	VCC1421	VC1470		Phage protein		
1	CDS	1552712	1554280	+	atgatagacatgttccacataaa	MIDMFHINIPFKKDVIIEMGDC(	fig|127906.87.peg.1422	VCC1422	VC1469		phage replication protein Cri		
1	CDS	1554285	1554569	+	atgaaagttgtatacatgggcatt	MKVVYMGISHRKGISNKGLGK	fig|127906.87.peg.1423	VCC1423	VC1468		hypothetical protein		
1	CDS	1554699	1555391	+	atgaacttgaatcaactcacggg	MNLNQLTGADLMAARTHIGLS	fig|127906.87.peg.1424	VCC1424	VC1467		hypothetical protein		
1	CDS	1555469	1555636	+	atgcagggacatcatacaagag	MQGHHTREDAKTTEATEQRL	fig|127906.87.peg.1425	VCC1425	VC1466		hypothetical protein		
1	CDS	1555629	1555796	+	atgactaaatacgtgcggctcaa	MTKYVRLNNVGAAWIKSTREK	fig|127906.87.peg.1426	VCC1426	Not present		hypothetical protein		Limited query/subject cover ffor certain strains
1	CDS	1557401	1556835	-	atgcgagtattaagaatgtcaga	MRVLRMSEVSIADRIKKAIIDN(	fig|127906.87.peg.1427	VCC1427	Not present		Phage protein		
1	CDS	1557563	1559131	+	atgatagacatgttccacataaa	MIDMFHINIPFKKDVIIEMGDC(	fig|127906.87.peg.1428	VCC1428	Not present		phage replication protein Cri		
1	CDS	1559136	1559420	+	atgaaagttgtatacatgggcatt	MKVVYMGISHRKGISNKGLGK	fig|127906.87.peg.1429	VCC1429	Not present		hypothetical protein		
1	CDS	1559550	1560242	+	atgaacttgaatcaactcacggg	MNLNQLTGADLMAARTHIGLS	fig|127906.87.peg.1430	VCC1430	Not present		hypothetical protein		
1	CDS	1560320	1560487	+	atgcagggacatcatacaagag	MQGHHTREDAKTTEATEQRL	fig|127906.87.peg.1431	VCC1431	Not present		hypothetical protein		
1	CDS	1560480	1561064	+	atgactaaatacgtgcggctcaa	MTKYVRLNNVGAAWIKSTREK	fig|127906.87.peg.1432	VCC1432	VC1465		hypothetical protein		Limited query cover for certain strains + Refseq doesnt not match with upstream/downstream genes
1	repeat	1561062	1563433	+	tagtgcgcattatgtggcgcggcattatgttgaggggcagtcgtcagtaccatt		fig|127906.87.repeat.28				repeat region		
1	CDS	1561892	1561554	-	atgaagataaaagaaaggcta	MKIKERLANQRKAINKTQAQM	fig|127906.87.peg.1433	VCC1433	VC1464		RstR phage-related transcriptional repressor		
1	CDS	1562018	1563097	+	atgaaaaagcagattttcactctt	MKKQIFTLDELQLDTNASPFVI	fig|127906.87.peg.1434	VCC1434	VC1463		RstA phage-related replication protein		
1	CDS	1563078	1563455	+	atgaaattatgggtgattaatatg	MKLWVINMKSRFVVFGASHSI	fig|127906.87.peg.1435	VCC1435	VC1462		RstB phage-related integrase		
1	CDS	1563455	1563598	+	atggacttaacccattatgtctgg	MDLTHYVWNEALYFAVVKAVI	fig|127906.87.peg.1436	VCC1436	Not present		hypothetical protein		
1	CDS	1563591	1563839	+	atgtttagctcactgaaaaacaa	MFSSLKNKLNTFKSTLSLGVFI	fig|127906.87.peg.1437	VCC1437	VC1461	cep	colonization factor		
1	CDS	1563946	1565133	+	atgcgctattttctactgtttttgaca	MRYFLLFLTLLFLSPSVTASAIN	fig|127906.87.peg.1438	VCC1438	VC1460		hypothetical protein		
1	CDS	1565139	1565423	+	atgatggacaccctttatgactgg	MMDTLYDWLIDGFTWLVIKLG	fig|127906.87.peg.1439	VCC1439	VC1459	ace	Accessory cholera enterotoxin		
1	CDS	1565420	1566619	+	atgagtatctttattcatcacggcg	MSIFIHHGAPGSYKTSGALWL	fig|127906.87.peg.1440	VCC1440	VC1458	zot	Zona occludens toxin		
1	CDS	1566718	1567494	+	atggtaaagataatatttgtgtttt	MVKIIFVFFIFLSSFSYANDDKL	fig|127906.87.peg.1441	VCC1441	VC1457	ctxA	Enterotoxin, A subunit (NAD(+)--diphthamide ADP- ribosyltransferase) (EC 2.4.2.36)		
1	CDS	1567491	1567865	+	atgattaaattaaaatttggtgtttt	MIKLKFGVFFTVLLSSAYAHGT	fig|127906.87.peg.1442	VCC1442	VC1456	ctxB	Enterotoxin, B subunit		
1	repeat	1567975	1570346	+	tagtgcgtattatgtggcgcggcattatgttgaggggcagtcgtcagtaccatt		fig|127906.87.repeat.29				repeat region		
1	CDS	1568805	1568467	-	atgaagataaaagaaaggcta	MKIKERLANQRKAINKTQAQM	fig|127906.87.peg.1443	VCC1443	VC1455		RstR phage-related transcriptional repressor		
1	CDS	1568931	1570010	+	atgaaaaagcagattttcactctt	MKKQIFTLDELQLDTNASPFVI	fig|127906.87.peg.1444	VCC1444	VC1454		RstA phage-related replication protein		
1	CDS	1569991	1570362	+	atgaaattatgggtgattaatatg	MKLWVINMKSRFVVFGASHSI	fig|127906.87.peg.1445	VCC1445	VC1453		RstB phage-related integrase		
1	CDS	1570456	1570680	+	atgagtttgaaaccatacactta	MSLKPYTLMDVYDSLEDLNNN	fig|127906.87.peg.1446	VCC1446	VC1452		RstC phage-related antirepressor		
1	CDS	1584685	1571048	-	atgggaaaaccattttggagaa	MGKPFWRSVEYFFTGNYSAD	fig|127906.87.peg.1447	VCC1447	VC1451		RTX toxins determinant A and related Ca2+-binding proteins		
1	CDS	1585104	1584709	-	atgctgctcagccagcattcgcc	MLLSQHSPLHRRYVVAEWLQ	fig|127906.87.peg.1448	VCC1448	VC1450		RTX toxin activating lysine-acyltransferase (EC 2.3.1.-)		
1	CDS	1585543	1585196	-	atgatggagaagttcgttcagac	MMEKFVQTVSSVNYNKGVFS	fig|127906.87.peg.1449	VCC1449	VC1449		hypothetical protein		
1	CDS	1585967	1588075	+	atgatatccaacaatgaaagac	MISNNERLTLICIHFYLSIISGNI	fig|127906.87.peg.1450	VCC1450	VC1448		RTX toxin transporter, ATP-binding protein		Limited query cover for certain strains
1	CDS	1588072	1589433	+	atgaacaccatcatgaagcgttt	MNTIMKRFFGQKSPHQPTEHI	fig|127906.87.peg.1451	VCC1451	VC1447		RTX toxin transporter, determinant D		
1	CDS	1589436	1591601	+	atgaacacccgttttacaaccga	MNTRFTTDAHRLAEQTLPQES	fig|127906.87.peg.1452	VCC1452	VC1446		RTX toxin transporter, ATP-binding protein		
1	CDS	1593374	1591662	-	atggattctgaatacacagagaa	MDSEYTEKVNELTLELQRSIEI	fig|127906.87.peg.1453	VCC1453	VC1445		Two-component system sensor histidine kinase		
1	CDS	1594594	1593377	-	gtgtcatctcacgtatacaaaac	MSSHVYKTRSMRLKTTVSTQ\	fig|127906.87.peg.1454	VCC1454	VC1444		hypothetical protein	histidine kinase [Vibrio cholerae]	
1	CDS	1595019	1594663	-	atggcagccgaaaaactcaca	MAAEKLTKARLAQILITLLVLIS	fig|127906.87.peg.1455	VCC1455	VC1443		hypothetical protein		
1	CDS	1595278	1596705	+	atgagccaagaaaagcagcttc	MSQEKQLEQNYNYTVVRQFT	fig|127906.87.peg.1456	VCC1456	VC1442	ccoN	Cytochrome c oxidase (cbb3-type) subunit CcoN (EC 1.9.3.1)		
1	CDS	1596719	1597339	+	atgagttctaattctaataatcgc	MSSNSNNRHELLERNVGLLAI	fig|127906.87.peg.1457	VCC1457	VC1441	ccoO	Cytochrome c oxidase (cbb3-type) subunit CcoO (EC 1.9.3.1)		
1	CDS	1597351	1597515	+	atggatatcggtactattcacagt	MDIGTIHSIWTIVLFICFIGVVW	fig|127906.87.peg.1458	VCC1458	VC1440	ccoQ	Cytochrome c oxidase (cbb3-type) subunit CcoQ (EC 1.9.3.1)		
1	CDS	1597523	1598503	+	atgactacattctggagtctttgga	MTTFWSLWIIVITVGTLLGCAIL	fig|127906.87.peg.1459	VCC1459	VC1439	ccoP	Cytochrome c oxidase (cbb3-type) subunit CcoP (EC 1.9.3.1)		
1	CDS	1598582	1599058	+	atggtaaagccttggtataaaca	MVKPWYKQFWPWFLIILPLTV	fig|127906.87.peg.1460	VCC1460	VC1438	ccoH	Putative analog of CcoH, COG3198		
1	CDS	1599063	1601435	+	atggacaaaacgtgttatcactg	MDKTCYHCGEDVPAGTNFHV	fig|127906.87.peg.1461	VCC1461	VC1437	ccoL	Type cbb3 cytochrome oxidase biogenesis protein CcoI; Copper-translocating P-type ATPase (EC 3.6.3.4)		
1	CDS	1601439	1601603	+	gtggaaagcttatatttgctcatcc	MESLYLLIPIAIVLVCVAVGIFLV	fig|127906.87.peg.1462	VCC1462	VC1436	ccoS	Type cbb3 cytochrome oxidase biogenesis protein CcoS, involved in heme b insertion		
1	CDS	1601600	1602274	+	gtgagtcctgatttatttggggctt	MSPDYLGAWVVGLLGAGHCL	fig|127906.87.peg.1463	VCC1463	VC1435		Heavy-metal-associated domain (N-terminus) and membrane-bounded cytochrome biogenesis cycZ-like domain, possible membrane copper tolerance protein		
1	CDS	1602364	1603116	+	atgatttctgaaaagcctgctgcc	MISEKPAAKRIQSGGCAIHCQI	fig|127906.87.peg.1464	VCC1464	VC1434	fnr	Fumarate and nitrate reduction regulatory protein		
1	CDS	1603246	1604193	+	atgagcatttatagtaaaattcttg	MSIYSKILVVADINHDEQPALA	fig|127906.87.peg.1465	VCC1465	VC1433		Universal stress protein E		
1	CDS	1604320	1605252	+	atgacagcgcaaactcaagag	MTAQTQELTKAQQYNFNKLQI	fig|127906.87.peg.1466	VCC1466	VC1432	ttcA	tRNA-(cytosine32)-2-thiocytidine synthetase TtcA		
1	CDS	1605988	1605329	-	atgaaatcaaaatcttacctcata	MKSKSYLILAPLFLSLSAPALA(	fig|127906.87.peg.1467	VCC1467	VC1431		Hypothetical protein, bax gene locus	DUF2987 domain-containing protein [Vibrio cholerae]	
1	CDS	1606790	1605990	-	atgcataagcgaataaccattac	MHKRITITLVWLIIVFIAMFGVY	fig|127906.87.peg.1468	VCC1468	VC1430		Putative Bax protein		
1	CDS	1607589	1606783	-	atgaataacgatatcgaaagag	MNNDIEREFNLGGSVEKALSG	fig|127906.87.peg.1469	VCC1469	VC1429		Probable proline and glycine rich transmembrane protein gene in bax		
1	CDS	1607914	1609047	+	gtgggagaaatacagacgttga	MGEIQTLNEQHSVGKPVIRLS	fig|127906.87.peg.1470	VCC1470	VC1428	potA	Spermidine/putrescine import ABC transporter ATP-binding protein PotA (TC 3.A.1.11.1)		
1	CDS	1609028	1609888	+	atgatgagcaaaaagcttaatct	MMSKKLNLQNAIVTLIVGWLVI	fig|127906.87.peg.1471	VCC1471	VC1427	potB	Spermidine/putrescine import ABC transporter permease protein PotB (TC 3.A.1.11.1)		
1	CDS	1609888	1610658	+	atgggacgttcaattagattagc	MGRSIRFSFMSLVYLFLYLPIIV	fig|127906.87.peg.1472	VCC1472	VC1426	potC	Spermidine/putrescine import ABC transporter permease protein PotC (TC 3.A.1.11.1)		
1	CDS	1610761	1611792	+	atgaaaaaactggctactcttta	MKKLATLLAGSACALSLLSGT/	fig|127906.87.peg.1473	VCC1473	VC1425	potD	Spermidine/putrescine import ABC transporter substrate-binding protein PotD (TC 3.A.1.11.1)		
1	CDS	1611904	1612941	+	atgaaaaataaactgtttgcgac	MKNKLFASALCAAALFTTNAM	fig|127906.87.peg.1474	VCC1474	VC1424		Spermidine/putrescine import ABC transporter substrate-binding protein PotD (TC 3.A.1.11.1)		
1	CDS	1613822	1613088	-	gtgaataaacataatgagtttga	MNKHNEFDDNEEVVVIERRDK	fig|127906.87.peg.1475	VCC1475	VC1423		Chromosome segregation ATPase		
1	CDS	1613997	1613839	-	atggtgagattaattgcaatagc	MVRLIAIALLIALAFVLIRYRTNE	fig|127906.87.peg.1476	VCC1476	VC1422a		hypothetical protein		
1	CDS	1614500	1615876	+	atgaataacctgcaatcttttctgc	MNNLQSFLQTVDSLVWGPPLI	fig|127906.87.peg.1477	VCC1477	VC1422		Sodium/glycine symporter GlyP		
1	CDS	1616587	1615949	-	atggttatccagaatttcgaagct	MVIQNFEAFFIAITILTLTPGLD1	fig|127906.87.peg.1478	VCC1478	VC1421		Transporter, LysE family		
1	CDS	1617412	1616675	-	atgaaaaaattgattttcttgttgtc	MKKLIFLLSALLLNSCSTNPVY	fig|127906.87.peg.1479	VCC1479	VC1420		hypothetical protein	DUF2931 family protein [Vibrio cholerae]	
1	CDS	1618300	1617557	-	atgaagttattgaataatcttgca	MKLLNNLAIKKLISVVVLFIALFI	fig|127906.87.peg.1480	VCC1480	VC1419		Antitoxin TsiV1 to TseL		
1	CDS	1620222	1618297	-	atggattcatttaattattgcgtgca	MDSFNYCVQCNPEENWLELE	fig|127906.87.peg.1481	VCC1481	VC1418		Lipase TseL		
1	CDS	1621052	1620225	-	atggaaaagatgcttttacaatg	MEKMLLQWLSEQTSNVYWLA	fig|127906.87.peg.1482	VCC1482	VC1417		FIG01201135: hypothetical protein	DUF4123 domain-containing protein [Vibrio cholerae]	
1	CDS	1624555	1621064	-	atggcgacattagcgtacagca	MATLAYSIEVEGLEDETLVVR(	fig|127906.87.peg.1483	VCC1483	VC1416		VgrG protein		
1	repeat	1622907	1625393	+	cgactttcccactccaccgtcaggtgttggttgtgcttaatctgggtaaattggtc		fig|127906.87.repeat.30				repeat region		
1	CDS	1625201	1624683	-	atgccaactccatgttatatctcta	MPTPCYISIEGQTQGLITAGAC	fig|127906.87.peg.1484	VCC1484	VC1415		T6SS component Hcp		
1	CDS	1625489	1626973	+	atgaatgcgttcaataaattagta	MNAFNKLVKHSKKISNFNHLS	fig|127906.87.peg.1485	VCC1485	VC1414		Thermostable carboxypeptidase 1 (EC 3.4.17.19)		
1	CDS	1627195	1628931	+	atggcccaactctcgtttaaaaa	MAQLSFKNKIIALIIAIISLTIITSY	fig|127906.87.peg.1486	VCC1486	VC1413		Methyl-accepting chemotaxis protein II (mcp-II) (aspartate chemoreceptor protein)		
1	CDS	1630541	1629006	-	atgagtcataacgctgacaatga	MSHNADNEMQPLSGWALFFG	fig|127906.87.peg.1487	VCC1487	VC1411	emrB/vceB	Multidrug efflux system EmrAB-OMF, inner-membrane proton/drug antiporter EmrB (MFS type)		
1	CDS	1631738	1630551	-	atgaattcaaataatagcaacac	MNSNNSNTEFDGVRAAQSRK	fig|127906.87.peg.1488	VCC1488	VC1410	emrA/vceA	Multidrug efflux system EmrAB-OMF, membrane fusion component EmrA		
1	CDS	1633206	1631752	-	gtgaaaaatagcgttcaaacgg	MKNSVQTVGLLHKAAPYFTAS	fig|127906.87.peg.1489	VCC1489	VC1409	mdtP	Outer membrane factor (OMF) lipoprotein associated wth EmrAB-OMF efflux system		
1	CDS	1633313	1633915	+	atgcgggtaaaaagtgaagaa	MRVKSEEKRQAILEIAKDSFTK	fig|127906.87.peg.1490	VCC1490	VC1408	yfiR	Transcriptional regulator, AcrR family		
1	CDS	1635167	1633974	-	atgtcttttctcaacttggattaag	MSFSQLGLSDVLVQTVAQLGY	fig|127906.87.peg.1491	VCC1491	VC1407		ATP-dependent RNA helicase VC1407		
1	CDS	1636647	1635349	-	atgccatttggaaaaaagctgc	MPFWKKAAAQSEVEQHNHDS	fig|127906.87.peg.1492	VCC1492	VC1406		Methyl-accepting chemotaxis sensor/transducer protein		
1	CDS	1638435	1636744	-	atgaaatggtttaataatttatcga	MKWFNNLSITQKMIALVGCLL/	fig|127906.87.peg.1493	VCC1493	VC1405	tsr	Methyl-accepting chemotaxis sensor/transducer protein		
1	CDS	1638771	1638893	+	ttggaaaatcttgaaattcaagac	MENLEIQEQTIIKISTKNIDADL/	fig|127906.87.peg.1494	VCC1494	Not annotated as CDS		FIG01203726: hypothetical protein		
1	CDS	1639027	1640952	+	atggcgctcaagaaacatatca	MALKKHITTSASSSFSPSNHS	fig|127906.87.peg.1495	VCC1495	VC1403		Methyl-accepting chemotaxis transducer dimer protein		
1	CDS	1640965	1642527	+	atgaccagtgaagccctgtaga	MTSEALVDNELEHEENLTQYV	fig|127906.87.peg.1496	VCC1496	VC1402		Positive regulator of CheA protein activity (CheW) / Positive regulator of CheA protein activity (CheW)		
1	CDS	1642548	1643624	+	atgaagattttagttgtcgatgatt	MKILVVDDSALMRTTISDILQN	fig|127906.87.peg.1497	VCC1497	VC1401		Chemotaxis response regulator protein-glutamate methylesterase CheB (EC 3.1.1.61)		
1	CDS	1643611	1644267	+	atggcaatcattaaacgcagcg	MAIIKRSAATDNPNIEILSTPEC	fig|127906.87.peg.1498	VCC1498	VC1400		FOG: HEAT repeat		
1	CDS	1644278	1645123	+	atgaatgagattgtcattaccgat	MNEIVITDTDFCRFRDYFYQKT	fig|127906.87.peg.1499	VCC1499	VC1399		Chemotaxis protein methyltransferase CheR (EC 2.1.1.80)		
1	CDS	1645123	1645494	+	atgaaaaaagtaatggtcgttga	MKKVMVVDDASTVRMYHKAL	fig|127906.87.peg.1500	VCC1500	VC1398		Chemotaxis regulator - transmits chemoreceptor signals to flagellar motor components CheY		Limited query cover for cetain strains
1	CDS	1645491	1647776	+	atgaacccaataatgcaaaact	MNPIMQNFIIESRDLIESAARG	fig|127906.87.peg.1501	VCC1501	VC1397		Signal transduction histidine kinase CheA		
1	CDS	1647766	1648026	+	atgcccgttgaatataaaaaccc	MPVEYKNRVAYLVDHVGVEE/	fig|127906.87.peg.1502	VCC1502	VC1396		hypothetical protein		
1	CDS	1648019	1648399	+	atgtctaaaagcattctgattgttg	MSKSILIVDDSSTVLMSVDAMI	fig|127906.87.peg.1503	VCC1503	VC1395		Chemotaxis regulator - transmits chemoreceptor signals to flagellar motor components CheY		
1	CDS	1648407	1649642	+	atgattcgttacctatttgaacggc	MIRYLFERLENNKKRYLFFTIT	fig|127906.87.peg.1504	VCC1504	VC1394		Methyl-accepting chemotaxis transducer domain protein		
1	CDS	1650011	1649697	-	atggcgtggttaatcctctttctcg	MAWLILFLAGICEIAWAIGLKYS	fig|127906.87.peg.1505	VCC1505	VC1393	sugE	small multidrug resistance family (SMR) protein		
1	CDS	1651671	1650157	-	atggaaaagatcaatctggtatg	MEKINLVWLKRDLRLTDHAPL(	fig|127906.87.peg.1506	VCC1506	VC1392	cry2	Cryptochrome		
1	CDS	1653233	1651827	-	atgcattttgatcctattcccgctg	MHFDPIPAADAPHPRIIFASVA	fig|127906.87.peg.1507	VCC1507	VC1391		Uncharacterized MFS-type transporter		
1	CDS	1653320	1654270	+	atgggacgcgaaattgcaaaaa	MGREIAKMGLPMKLDDLALFL	fig|127906.87.peg.1508	VCC1508	VC1390		Transcriptional regulator, LysR family		
1	CDS	1654427	1654248	-	gtgacttcgcataaaaaagccta	MTSHKKAYLAQKICPVCQRPF	fig|127906.87.peg.1509	VCC1509	VC1389		Uncharacterized protein Caur_1846	DUF2256 domain-containing protein [Vibrio cholerae]	
1	CDS	1655679	1654663	-	atgactaagactcgcattctgctc	MTKTRILLSDSTDPWFNLAVEI	fig|127906.87.peg.1510	VCC1510	VC1388	lplA	Lipoate-protein ligase A		
1	CDS	1657156	1655804	-	atgtttattggatttgactatggtac	MFIGFDYGTANCSVASMQDG	fig|127906.87.peg.1511	VCC1511	VC1386		Uncharacterized chaperone protein YegD	Molecular chaperone [Vibrio cholerae]	
1	CDS	1657855	1657373	-	atgaaaaagacattacttgctctg	MKKTLLALALLGASSTAMADS	fig|127906.87.peg.1512	VCC1512	VC1384		hypothetical protein	porin family protein [Vibrio cholerae]	
1	CDS	1661924	1657995	-	ttgacttcgtcacagcccacacc	MTSSQPTPDSAAQPKANSAA	fig|127906.87.peg.1513	VCC1513	VC1382	hrpA	ATP-dependent helicase HrpA		
1	CDS	1661936	1662091	+	ttgttatctttccaaaaaactgcgc	MLSFQKTARIVPQSFSNTPMIF	fig|127906.87.peg.1514	VCC1514	Not annotated as CDS		hypothetical protein		
1	CDS	1662126	1662476	+	gtgaaagcctctgaactgaaaa	MKASELKTILNQLPDDLDPDV	fig|127906.87.peg.1515	VCC1515	VC1380		hypothetical protein		
1	CDS	1662529	1662987	+	atgtttaatacactcacctccctgt	MFNTLTSLFKQLLEGQDLAQH	fig|127906.87.peg.1516	VCC1516	VC1379		hypothetical protein		
1	CDS	1663217	1663089	-	atggttgtccacaaagccgagta	MVVHKAEYKNGKYTLRLLAGE	fig|127906.87.peg.1517	VCC1517	VC1378		FIG01201643: hypothetical protein		
1	CDS	1663803	1663273	-	atgaattacaaccgtgttgtgtgc	MNYNRVVCFDLEMCCWNEN(	fig|127906.87.peg.1518	VCC1518	VC1377		hypothetical protein	exonuclease domain-containing protein [Vibrio]	
1	CDS	1665404	1663821	-	atgctcaaacatgtgcgcgtgat	MLKHVRVIVPLLVLLFSLLLTVF	fig|127906.87.peg.1519	VCC1519	VC1376		GGDEF domain protein		
1	CDS	1665942	1666574	+	atgaatatcgacctaaacctgat	MNIDLNLIPTTFDMLHAGLAAS	fig|127906.87.peg.1520	VCC1520	VC1375		hypothetical protein	DUF2760 domain-containing protein [Vibrio cholerae]	

1	CDS	1666574	1668469	+	atggagatgccaattcattccgc	MEMPIHSAKYSVGIDLGTTHC	fig|127906.87.peg.1521	VCC1521	VC1374		DnaK-related protein		
1	CDS	1668529	1671357	+	atgtcatcaccacgttttctggtcg	MSSPRFLVGIDLGTTNTVVAF	fig|127906.87.peg.1522	VCC1522	VC1373		DnaK-related protein		
1	CDS	1672626	1671469	-	atgatggatcttcttttttagaaat	MMDLSFLEIATVINNIACSAIAA	fig|127906.87.peg.1523	VCC1523	VC1372		GGDEF domain protein		
1	CDS	1673021	1673701	+	atggaacaagataaatttacaca	MEQDKFTHVFRLPGSIQVRIAI	fig|127906.87.peg.1524	VCC1524	VC1371		hypothetical protein		
1	CDS	1675083	1673752	-	ttgatcaactcattaaaccgcaa	MINSLNRKMTLLFIASLLLVGFI	fig|127906.87.peg.1525	VCC1525	VC1370		GGDEF domain protein		
1	CDS	1676096	1675080	-	atgtttttggctcggcaatcccga	MFLARQSRRWIVYLSILAIAFV	fig|127906.87.peg.1526	VCC1526	VC1369		ABC transporter, substrate-binding protein (cluster 10, nitrate/sulfonate/bicarbonate)		
1	CDS	1676477	1676253	-	atgttttgggacacactcgaacg	MFWDTLERVNRLRQQAMNNF	fig|127906.87.peg.1527	VCC1527	VC1368		hypothetical protein		
1	CDS	1676729	1678675	+	atgaaactctattttaaacgctcg	MKLYFKRSLLVFSLMSSFCVA	fig|127906.87.peg.1528	VCC1528	VC1367	yedQ	GGDEF domain protein		
1	CDS	1679342	1678647	-	atgaaaaaagccgtcgtcgtgtt	MKKAVVVFSGGQDSTTCLVQ	fig|127906.87.peg.1529	VCC1529	VC1366	queC/exsB	7-cyano-7-deazaguanine synthase (EC 6.3.4.20)		
1	CDS	1680005	1679355	-	atgtttgagattatccaaggcgaa	MFEIIQGEGVFTGVPAVFVRL(	fig|127906.87.peg.1530	VCC1530	VC1365	queE	7-carboxy-7-deazaguanine synthase (EC 4.3.99.3)		
1	CDS	1680070	1680222	+	atggagatagcgatcgaggtca	MEIAIEVKLNASIPFLVQASAIL	fig|127906.87.peg.1531	VCC1531	Not annotated as CDS		hypothetical protein		
1	CDS	1680368	1681189	+	atgtacaaactgattgcgctcga	MYKLIALDMDGTLLNSQKQISI	fig|127906.87.peg.1532	VCC1532	VC1364	yidA	Predicted hydrolase		
1	CDS	1682193	1681255	-	atgcgctattttcctctgtttttggat	MRYFPLFLDLTNKPVLVVGGG	fig|127906.87.peg.1533	VCC1533	VC1363		Precorrin-2 oxidase (EC 1.3.1.76) @ Sirohydrochlorin ferrochelatase activity of CysG (EC 4.99.1.4)		
1	CDS	1682648	1683676	+	atggcgaataaactcactgttctt	MANKLTVLASVVAASSAMLSL	fig|127906.87.peg.1534	VCC1534	VC1362	aapJ	L-amino acid ABC transporter (Glu/Asp/His/...), substrate-binding protein AapJ		
1	CDS	1683802	1685007	+	atgaaacccacaaataaagcg	MKPTNKATPETSDAHSKHTNL	fig|127906.87.peg.1535	VCC1535	VC1361	glnM	L-amino acid ABC transporter (Glu/Asp/His/...), permease protein 1 AapQ		
1	CDS	1685010	1686107	+	atggcaacacatcaatttcaacc	MATHQFQPSLPPPSNTTGVV(	fig|127906.87.peg.1536	VCC1536	VC1360	yhdY	L-amino acid ABC transporter (Glu/Asp/His/...), permease protein 2 AapM		
1	CDS	1686130	1686885	+	atgacgcaacaactcaatcaac	MTQQLNQDFMIQIKDMNKWY	fig|127906.87.peg.1537	VCC1537	VC1359		L-amino acid ABC transporter (Glu/Asp/His/...), ATP-binding protein AapP		
1	CDS	1687130	1687801	+	atgaatacgccaatgtttaccag	MNTPMFTRTSSLERTLETNKV	fig|127906.87.peg.1538	VCC1538	VC1358	yccA	Putative TEGT family carrier/transport protein		
1	CDS	1687915	1688244	+	atgctggaataccaaggcaaaa	MLEYQGKTIDTDAQGYLLDFT	fig|127906.87.peg.1539	VCC1539	VC1356	tusE	tRNA 2-thiouridine synthesis protein TusE		
1	CDS	1688594	1688310	-	atgaaaaaaatggaaaaacag	MKKMEKQCSKFIVSGHVQGV	fig|127906.87.peg.1540	VCC1540	VC1355	yccX	Acylphosphate phosphohydrolase (EC 3.6.1.7)		
1	CDS	1688831	1690024	+	atgactcctgcaatttatttggtca	MTPAIYLVKGRDKSLRRKHPW	fig|127906.87.peg.1541	VCC1541	VC1354	rmlI	23S rRNA (cytosine(1962)-C(5))-methyltransferase (EC 2.1.1.191)		
1	CDS	1692996	1690066	-	atgacaggaattaggctttagct	MTGIRLLACCGVQICSVFLLIIS	fig|127906.87.peg.1542	VCC1542	VC1353		GGDEF domain protein		
1	CDS	1693647	1694120	+	atgattcaaatcggacaaacctt	MIQIGQTLPDVQLSQRTSEGT	fig|127906.87.peg.1543	VCC1543	VC1350		Antioxidant, putative		
1	CDS	1698221	1694226	-	atgcgctcgataaaagcgatgtt	MRSIKAMFVLLFCLMAILAVAM	fig|127906.87.peg.1544	VCC1544	VC1349		FOG: CheY-like receiver		
1	CDS	1698343	1699482	+	atgtcaatggaagaccttagcca	MSMEDLSQCTILIVDDSPDNIA	fig|127906.87.peg.1545	VCC1545	VC1348		Response regulator c-di-GMP phosphodiesterase, RpfG family		
1	CDS	1700108	1699464	-	atgagtttaattctctatggttattg	MSLILYGYWRSSAAYRVRIALI	fig|127906.87.peg.1546	VCC1546	VC1347		Maleylacetoacetate isomerase (EC 5.2.1.2) @ Glutathione S-transferase, zeta (EC 2.5.1.18)		
1	CDS	1701127	1700108	-	atgaaattagcgactctgaaaaa	MKLATLKNGMRDGQLVVVSR	fig|127906.87.peg.1547	VCC1547	VC1346		Fumarylacetoacetase (EC 3.7.1.2)		
1	CDS	1702270	1701134	-	atgcataaatggatcactttccg	MHKWITFPHREGTCSRQAHA	fig|127906.87.peg.1548	VCC1548	VC1345	hmgA	Homogentisate 1,2-dioxygenase (EC 1.13.11.5)		
1	CDS	1703336	1702263	-	atgatggatagcgtaaacccac	MMDSVNPLGTDGFEFVEYTA	fig|127906.87.peg.1549	VCC1549	VC1344	lly/hppD	4-hydroxyphenylpyruvate dioxygenase (EC 1.13.11.27)		
1	CDS	1703371	1703529	+	atggctgaaatgatccataaag	MAEMIHKENPRPVNGLTHPKF	fig|127906.87.peg.1550	VCC1550	Not annotated as CDS		hypothetical protein		
1	CDS	1703796	1704902	+	atgagtctgattaataccaacg	MSLINTQRLVDHFLQLIQIDSE	fig|127906.87.peg.1551	VCC1551	VC1343		Uncharacterized peptidase YqjE		
1	repeat	1704921	1705550	+	aataaaaatccgccttcttagaag	gcggtttaatataagcccccctaaaaggg	fig|127906.87.repeat.31				repeat region		
1	CDS	1705424	1704987	-	atgggcgactacagaagttcatc	MGDYRSSSHVYWRCKYHIVW	fig|127906.87.peg.1552	VCC1552	Not annotated as CDS or not present	tnpA	Mobile element protein		
1	CDS	1707038	1705590	-	ttgttggcatatgttcgcgcatttt	MLAYVRAFWLMLGFTFCMGL	fig|127906.87.peg.1553	VCC1553	VC1342		Membrane-associated phospholipid phosphatase		
1	CDS	1707544	1707068	-	atggccaatatctcactctcttcg	MANISLSSASSADLQQLNQLM	fig|127906.87.peg.1554	VCC1554	VC1341		Histone acetyltransferase HPA2		
1	CDS	1709595	1707718	-	atgtctgcctatcaagagcaata	MSAYQEQYQWAKEQPEAFW	fig|127906.87.peg.1555	VCC1555	VC1340	acsA/prpE	Propionate--CoA ligase (EC 6.2.1.17)		
1	CDS	1710802	1709600	-	atgaacgagacagtgccacaa	MNETVPQIRIPATYMRGGTSK	fig|127906.87.peg.1556	VCC1556	VC1339	mii	2-methylaconitate isomerase		
1	CDS	1713417	1710811	-	atgaacagtctgtatcgtaaagc	MNSLYRKALSPSPAQSQVDFF	fig|127906.87.peg.1557	VCC1557	VC1338	acnA	2-methylcitrate dehydratase (2-methyl-trans-aconitate forming) (EC 4.2.1.117)		
1	CDS	1714646	1713513	-	atgtctgctgcaaaagagttagg	MSAAKELGGAGLRGQSAGST	fig|127906.87.peg.1558	VCC1558	VC1337	prpC	2-methylcitrate synthase (EC 2.3.3.5)		
1	CDS	1715718	1714822	-	atgagtttaagtcctggcgccaa	MSLSPGAKFRLAVKTHHPLQI	fig|127906.87.peg.1559	VCC1559	VC1336	prpB	Methylisocitrate lyase (EC 4.1.3.30)		
1	CDS	1716433	1715729	-	atgaaaagtagtccgttagtccg	MKSSPLVRQASTEKESTKSEN	fig|127906.87.peg.1560	VCC1560	VC1335	ydfH	Propionate catabolism operon transcriptional regulator of GntR family		
1	CDS	1717066	1718037	+	atgctgaagtttttaaaccctccc	MLKFFKPSLTAAMLAATFSFS	fig|127906.87.peg.1561	VCC1561	VC1334		Tripartite tricarboxylate transporter TctC family		
1	CDS	1718289	1718786	+	atgtcggacctgccaaccaaaa	MSDLPTKIFTKENLLCRDRVG	fig|127906.87.peg.1562	VCC1562	VC1333		Tripartite tricarboxylate transporter TctB family		
1	CDS	1718796	1720325	+	atgttagacggaattttacaagg	MLDGILQGLATAVMPTNLMMV	fig|127906.87.peg.1563	VCC1563	VC1332		Tripartite tricarboxylate transporter TctA family		
1	CDS	1721273	1720476	-	atgctcaaacggcctttttagcgc	MLKRPFSALLIILLAACSTHHSI	fig|127906.87.peg.1564	VCC1564	VC1330		probable lipoprotein YPO2292		
1	CDS	1722262	1721624	-	atgaaaacagttatttctttgcttg	MKTVISLLATSILFASSVQAETT	fig|127906.87.peg.1565	VCC1565	VC1329		Opacity protein-related protein	outer membrane beta-barrel protein [Vibrio cholerae]	
1	CDS	1723679	1722672	-	atggatgctgtcaaatcatttgca	MDAVKSFAIKHLKEGAIYAVLF	fig|127906.87.peg.1566	VCC1566	VC1328	mglC	Galactose/methyl galactoside ABC transporter, permease protein MglC (EC 3.6.3.17)		
1	CDS	1725234	1723690	-	gtgttgctgaattttgcataataa	MLSEFLHNKSDAMVNQTHEFI	fig|127906.87.peg.1567	VCC1567	VC1327	mglA	Galactose/methyl galactoside ABC transporter, ATP-binding protein MglA (EC 3.6.3.17)		
1	CDS	1726378	1725404	-	atgagaaagttaactgttttagca	MRKLTVLAMLSAGMGFGTQA	fig|127906.87.peg.1568	VCC1568	VC1325	mglB	Galactose/methyl galactoside ABC transporter, substrate-binding protein MglB (EC 3.6.3.17)		
1	CDS	1726877	1727359	+	atgattctagccgttcctagccgc	MILAVPSRHGVPFNHFAKAPS	fig|127906.87.peg.1569	VCC1569	VC1323		hypothetical protein	Dinitrogenase iron-molybdenum cofactor [Vibrio cholerae]	
1	CDS	1727356	1727553	+	atgacgtatctactggctttcggtc	MTYLLAFGAFVLFAVLMTLGV	fig|127906.87.peg.1570	VCC1570	VC1322		hypothetical protein	(Na+)-NQR maturation NqrM [Vibrio cholerae]	
1	CDS	1730705	1727634	-	gtggcttctgactcaattcctcgc	MASDSIPRRVLKKRRRAQKNI	fig|127906.87.peg.1571	VCC1571	VC1321		Membrane carboxypeptidase (penicillin-binding protein)		
1	CDS	1731081	1731785	+	atgtctaaccaaccccagtctcta	MSNQPSLYIIEDDTKLREMLAE	fig|127906.87.peg.1572	VCC1572	VC1320		Two-component transcriptional response regulator, OmpR family		
1	CDS	1731811	1733133	+	atgcgccgcatttacatcgagtc	MRRIYIESLVSLIVLFFGSLVSY	fig|127906.87.peg.1573	VCC1573	VC1319	rstB	Two-component system sensor histidine kinase		
1	CDS	1733346	1734119	+	atgaaaaagatcgcactatttat	MKKIALFITASLIAGNALAAQTY	fig|127906.87.peg.1574	VCC1574	VC1318	ompV	Outer membrane protein OmpV		
1	CDS	1734217	1735137	+	atgaacccacgcattgattatta	MNPRIDYLKSLSTLAAEIYPDIR	fig|127906.87.peg.1575	VCC1575	VC1317	virK	VirK/YbjX family protein	DUF535 domain-containing protein [Vibrio cholerae]	
1	CDS	1735641	1735165	-	atggagaaactgaacatcattg	MEKLNIICVDDQREVLSAVLQ	fig|127906.87.peg.1576	VCC1576	VC1316		Putative chemotaxis protein CheY		
1	CDS	1736959	1735610	-	atgtccaacttagcggccgttatt	MSNLAAVIERYFNHADRCITLR	fig|127906.87.peg.1577	VCC1577	VC1315		Two-component system sensor histidine kinase		
1	CDS	1738384	1736969	-	atgatgcgcccttatcttaaatat	MMRPYLKYIIPLLIPMLVLMLP	fig|127906.87.peg.1578	VCC1578	VC1314		Sodium-dependent anion transporter family		
1	CDS	1738766	1740397	+	atgtttctaaaaaacttgtcgata	MFLKNLSIGKKIAASFSVIAVIS	fig|127906.87.peg.1579	VCC1579	VC1313		Methyl-accepting chemotaxis sensor/transducer protein		
1	CDS	1740509	1741732	+	atgcattttaaagccacgctgctt	MHFKATLLSLSIAATLPSFSLS	fig|127906.87.peg.1580	VCC1580	VC1312		Arginine racemase (EC 5.1.1.9) @ Lysine racemase (EC 5.1.1.5) @ Ornithine racemase (EC 5.1.1.12)		
1	CDS	1741853	1742731	+	atgccaacaataaaatctccttt	MPTIKSPLLIAILSLLAAMITIQS	fig|127906.87.peg.1581	VCC1581	VC1311	rhtA	Threonine/homoserine exporter RhtA		
1	CDS	1743272	1742799	-	atgtcttatttgcctttggatcaata	MSYLPLDQYQRKWIFTHASMI	fig|127906.87.peg.1582	VCC1582	VC1310		hypothetical protein	DUF2947 domain-containing protein [Vibrio cholerae]	
1	CDS	1743942	1743367	-	atgaatggattaaacagcgaat	MNGLNSECHFYQTDGDIVVR(	fig|127906.87.peg.1583	VCC1583	VC1309	ydaF/rimJ	Ribosomal-protein-S5p-alanine acetyltransferase (EC 2.3.1.128)		
1	CDS	1745483	1743942	-	gtgcgtcttgaagtcctctgtgaa	MRLEVLCEDRLGLTRELLDILA	fig|127906.87.peg.1584	VCC1584	VC1308	tyrR	Transcriptional repressor protein TyrR		
1	CDS	1746616	1745597	-	atgagcgatcttaaaaccaaac	MSDLKTKQIFSEPLQGSLETP	fig|127906.87.peg.1585	VCC1585	VC1307		hypothetical protein		ORF split into 2 ORFs in N16961, none with refseq in PATRIC. Annotated as 1 pseudogene ORF named VC1307 in KEGG.
									VC1307				ORF split into 2 ORFs in N16961, none with refseq in PATRIC. Annotated as 1 pseudogene ORF named VC1307 in KEGG.
1	CDS	1747986	1746613	-	atgcagcgtattcgtcaagaaat	MQRIRQEMSDFIHRGLDSHVF	fig|127906.87.peg.1586	VCC1586	VC1306		Conserved protein YcjX with nucleoside triphosphate hydrolase domain		
1	CDS	1748396	1748211	-	atgcctcgttttattcagattctaca	MPRFIQILQIIAVVIGAVVGYDL	fig|127906.87.peg.1587	VCC1587	VC1305		hypothetical protein		
1	CDS	1750062	1748545	-	atgacggtaatacgcaagcaa	MTVIRKQDVISSVADALQYISY	fig|127906.87.peg.1588	VCC1588	VC1304	fumB	Fumarate hydratase class I, aerobic (EC 4.2.1.2)		
1	CDS	1750238	1751614	+	atgaataacaaccaaactttgtc	MNNNQTLSIEHKKLTYHPDIAH	fig|127906.87.peg.1589	VCC1589	VC1303	pabB	Para-aminobenzoate synthase, aminase component (EC 2.6.1.85)		
1	CDS	1751811	1752425	+	ttgcaaaacattagccgtcacga	MQNISRHEFIQQFQLHLPAAY	fig|127906.87.peg.1590	VCC1590	VC1302		Uncharacterized Nudix hydrolase NudL	CoA pyrophosphatase [Vibrio cholerae]	
1	CDS	1752724	1753977	+	atgaatactacaacagcggcta	MNTTTAATSTARSASKWTYKI	fig|127906.87.peg.1591	VCC1591	VC1301		Serine transporter	septum formation protein [Vibrio cholerae]	
1	CDS	1754070	1755431	+	atgattagtgtatttgatatctataa	MISVFDIYKIGVGPSSSHTVGP	fig|127906.87.peg.1592	VCC1592	VC1300		L-serine dehydratase, beta subunit (EC 4.3.1.17) / L-serine dehydratase, alpha subunit (EC 4.3.1.17)		
1	CDS	1755888	1755520	-	atgatgatgaaaacagaattata	MMMKTELYKEFMFEAAHHLP	fig|127906.87.peg.1593	VCC1593	VC1299	queD	6-carboxy-5,6,7,8-tetrahydropterin synthase (EC 4.1.2.50)		
1	repeat	1756018	1756151	+	aatatacccaaactacttggagt	tgcaggtaggcggcaagtgagtgagtcc	fig|127906.87.repeat.32				repeat region		
1	CDS	1756286	1757917	+	atgaactcatccattcttagtaaa	MNSSILSKLLLTLITVFALVLAG	fig|127906.87.peg.1594	VCC1594	VC1298		Methyl-accepting chemotaxis sensor/transducer protein		
1	CDS	1758064	1759464	+	atgacttacgcgcctgtaaatga	MTYAPVNDVLSGKLAVDSEVT	fig|127906.87.peg.1595	VCC1595	VC1297	asnC	Asparaginyl-tRNA synthetase (EC 6.1.1.22)		
1	CDS	1760378	1759530	-	atgtcaatatctcttcattcatac	MSIISSIHTPIVLTIAGSDSGGG	fig|127906.87.peg.1596	VCC1596	VC1296	thiD	Hydroxymethylpyrimidine phosphate kinase ThiD (EC 2.7.4.7)		
1	repeat	1760527	1760659	+	tgttataccccaaactacttggagt	tgcaggtaggcggcgacaagtgagtcatccc	fig|127906.87.repeat.33				repeat region		
1	CDS	1760914	1762173	+	atgaaaaacacgatttaccatg	MKNTIYHAPLTFGLYALAGVLF	fig|127906.87.peg.1597	VCC1597	VC1295		Response regulator		2 ORFs in C6706 both annotated
1	CDS	1762173	1762364	+	atggcgttagccgatgtgatga	MALADVYDALISARVYKPAFS	fig|127906.87.peg.1598	VCC1598	VC1295		Response regulator		2 ORFs in C6706 both annotated
1	CDS	1762402	1762521	+	gtgttggcgacgctctgctttgcg	MLATLCFANAQSMTMERNEN	fig|127906.87.peg.1599	VCC1599	VC1294		FIG01201641: hypothetical protein		
1	CDS	1762733	1763923	+	atgttcgaaaaagtcgttgccgc	MFEKVVAAPADPILGLTEEFK	fig|127906.87.peg.1600	VCC1600	VC1293	aspC	Aspartate aminotransferase (EC 2.6.1.1)		
1	CDS	1764228	1766117	+	atggacgccgagttaatcgaga	MDAELIEIQQFLQQHPPFDAL	fig|127906.87.peg.1601	VCC1601	VC1291		Predicted signal-transduction protein containing cAMP-binding and CBS domains		
1	CDS	1766123	1766824	+	atgttcgattctgcgatttactggc	MFDSAIYWPARFAHLAQQAR(	fig|127906.87.peg.1602	VCC1602	VC1290		DEDDh 3'-5' exonuclease domain of the epsilon subunit of DNA polymerase III		
1	CDS	1768846	1766963	-	atgaaattaacactaaaacaca	MKLTLKHKLIGASLSAVVVMAT	fig|127906.87.peg.1603	VCC1603	VC1289		Methyl-accepting chemotaxis sensor/transducer protein		
1	CDS	1769251	1770888	+	atgattcgtgtgtccagcgccgtg	MIRVSSAVQRHAQKLIVLFSLL	fig|127906.87.peg.1604	VCC1604	VC1288	mdoG	Glucans biosynthesis protein G precursor		
1	CDS	1770907	1773072	+	atgacaaacccaatggtgaac	MTNPMVEQGASQLMGGSAM	fig|127906.87.peg.1605	VCC1605	VC1287	opgH/mdoH	Glucans biosynthesis glucosyltransferase H		
1	CDS	1774240	1773218	-	atggcaagaattaaagatgttgc	MARIKDVAELAGVNRSTVSRII	fig|127906.87.peg.1606	VCC1606	VC1286		Transcriptional regulator, LacI family		
1	CDS	1775099	1774341	-	atgaaggtaattttaacgcggat	MKVIFNADDFGLTQGVNQGIV	fig|127906.87.peg.1607	VCC1607	VC1285		Cellobiose phosphotransferase system YdjC-like protein		
1	CDS	1776444	1775122	-	atgtctcgaaatgcattaaagct	MSRNALKLAVIGGGSSYTPEL	fig|127906.87.peg.1608	VCC1608	VC1284	licH/celF	6-phospho-beta-glucosidase (EC 3.2.1.86)		
1	CDS	1776761	1776426	-	atggaacaagaacttatcgtgat	MEQELIVMEIICNAGEARSLCY	fig|127906.87.peg.1609	VCC1609	VC1283	chbA/celC	PTS system, glucosaminyl-1,4-glucosamine-specific IIA component		
1	CDS	1778139	1776808	-	atgaagctttatgatgcgataatt	MKLYDAIIGVEKHIAPIAAKVG	fig|127906.87.peg.1610	VCC1610	VC1282	licC/celB	PTS system, glucosaminyl-1,4-glucosamine-specific IIC component		
1	CDS	1778532	1778227	-	atgaaaaagatcctctgctgctg	MKKILLCCSAGMSTSMLVKKM	fig|127906.87.peg.1611	VCC1611	VC1281	celA	PTS system, glucosaminyl-1,4-glucosamine-specific IIB component		
1	CDS	1778787	1780070	+	atgaaattaaaaaaattggctctt	MKLKKLALFTAISLAISGHSFAI	fig|127906.87.peg.1612	VCC1612	VC1280	deaA	GlcNAc-GlcN deacetylase		
1	CDS	1781839	1780235	-	atgacaaaagaaattgataagt	MTKEIDKYSIDNTDYTVGQDN	fig|127906.87.peg.1613	VCC1613	VC1279	opuD	High-affinity choline uptake protein BetT		
1	CDS	1782214	1782693	+	gtggaaaaacacgaagaagta	MEKHEEVLVALRQIIRAIDLHS	fig|127906.87.peg.1614	VCC1614	VC1278		Transcriptional regulator, MarR family		
1	CDS	1783333	1782695	-	atggataaaccaatcagagtgg	MDKPIRVVMVDDHQVVLDGF	fig|127906.87.peg.1615	VCC1615	VC1277	nreC	Two-component transcriptional response regulator, LuxR family		
1	CDS	1784681	1783326	-	atgcctctcaaagccaaattgat	MPLKAKLILLALLPLLIATASISV	fig|127906.87.peg.1616	VCC1616	VC1276	nreB	Two-component system sensor histidine kinase		
1	CDS	1786071	1784788	-	atgattggtattgtgatgttttcgtt	MIGIVMFFVALFALLLGFPVAF	fig|127906.87.peg.1617	VCC1617	VC1275		TRAP dicarboxylate transporter, DctM subunit, unknown substrate 6		
1	CDS	1786614	1786081	-	atgagaagtctgatttatgttgag	MRSLIYVERLFNRLSDLLGLLS	fig|127906.87.peg.1618	VCC1618	VC1274		TRAP dicarboxylate transporter, DctQ subunit, unknown substrate 6		
1	CDS	1787834	1786716	-	atggataaccaaggacgtcaa	MDNQGRQTMSLIHQSVSRVM	fig|127906.87.peg.1619	VCC1619	VC1273		TRAP transporter solute receptor, unknown substrate 6		
1	CDS	1788594	1788196	-	atgagactagctcataagcgta	MRLAHKRKVQAKLQKRIKAIV	fig|127906.87.peg.1620	VCC1620	VC1272		hypothetical protein		
1	CDS	1788767	1789441	+	atgcccactttgcaactgtacaa	MPTLQLYNHQIHFTPFVIKGLL	fig|127906.87.peg.1621	VCC1621	VC1271		hypothetical protein	DUF2982 domain-containing protein [Vibrio cholerae]	
1	CDS	1790091	1789435	-	atgtctttacaatatcaggtggtg	MSLQYQVVPVTAFAQNCSIVV	fig|127906.87.peg.1622	VCC1622	VC1270	pksB	Hypothetical metal-binding enzyme, YcbL homolog		
1	CDS	1790703	1790155	-	gtgcaaacaacacgtcgagatt	MQTTRRDFLKLTAGGLILAAC	fig|127906.87.peg.1623	VCC1623	VC1269		FIG001587: exported protein	DUF882 domain-containing protein [Vibrio cholerae]	
1	CDS	1792420	1790849	-	atgaaaagccgagtcgttgccg	MKSRVVAAIALLFFVIPCSATQ	fig|127906.87.peg.1624	VCC1624	VC1268		L,D-transpeptidase YcbB		
1	CDS	1793752	1792670	-	atgcaaccgatggtgactgatg	MQPMVTDATRRHLLQVALFG	fig|127906.87.peg.1625	VCC1625	VC1267		hypothetical protein	DUF1513 domain-containing protein [Vibrio cholerae]	
1	CDS	1794740	1793733	-	atgaaaaaaacagtgactctgc	MKKTVTLLAVSLLAACQSAPD	fig|127906.87.peg.1626	VCC1626	VC1266		Iron-regulated protein A precursor		
1	CDS	1796135	1794750	-	atgaagtcgtcttatctctgtattt	MKSSLSLLLLTSALSNFALATE	fig|127906.87.peg.1627	VCC1627	VC1265		Probable thiol oxidoreductase with 2 cytochrome c heme-binding sites		
1	CDS	1797604	1796342	-	atgaacgttaagtctattctcttc	MNVKSILSSTILASLTLTTSASF	fig|127906.87.peg.1628	VCC1628	VC1264	irpA	Iron-regulated protein A precursor		
1	CDS	1798434	1797784	-	atgcaaatgttgagaaacgtgc	MQMLRNVHPVTEIQVSANMA	fig|127906.87.peg.1629	VCC1629	VC1263	ribA	GTP cyclohydrolase II (EC 3.5.4.25)		
1	CDS	1798781	1798605	-	gtggacaattcggttgttaccca	MDNSVVTQLKKEFYAQLSALQ	fig|127906.87.peg.1630	VCC1630	VC1262		hypothetical protein	3-demethylubiquinone-9 3-methyltransferase [Vibrio cholerae HC-7A1]	
1	CDS	1800178	1798964	-	atgtatcgagataaaaccgcctt	MYRDKTALLFIMSTLVTGLCG	fig|127906.87.peg.1631	VCC1631	VC1261	setA	Sugar efflux transpoter		
1	CDS	1800429	1801328	+	atgcgacctttaattgaaaatgtg	MRPLIENVMSGISQDWVLREF	fig|127906.87.peg.1632	VCC1632	VC1260		Transcriptional regulator, AraC family		
1	CDS	1802353	1801355	-	ttgaaaggtcataccatgagtca	MKGHTMSQYVVCALYKFVELI	fig|127906.87.peg.1633	VCC1633	VC1259		Rhodanese domain protein UPF0176	rhodanese-related sulfurtransferase [Vibrio cholerae]	
1	CDS	1805250	1802566	-	atgagcgatctagctaaagaga	MSDLAKEITPVNIEDELRSSYL	fig|127906.87.peg.1634	VCC1634	VC1258	gyrA	DNA gyrase subunit A (EC 5.99.1.3)		
1	CDS	1805602	1806339	+	atggcttcaatagacttggcttcg	MASIDLASTPLTATQNVDPNE	fig|127906.87.peg.1635	VCC1635	VC1257	ubiG	3-demethylubiquinol 3-O-methyltransferase (EC 2.1.1.64) @ 2-polyprenyl-6-hydroxyphenyl methylase (EC 2.1.1.222)		
1	CDS	1806788	1809073	+	atgaaccaacaacttactgtcac	MNQQLTVTKRDGRKENIDLDI	fig|127906.87.peg.1636	VCC1636	VC1256	nrdA	Ribonucleotide reductase of class Ia (aerobic), alpha subunit (EC 1.17.4.1)		
1	CDS	1809157	1810302	+	atgaaggcaacaatggcttaca	MKATMAYSTFTQKKNDQLKEI	fig|127906.87.peg.1637	VCC1637	VC1255	nrdB	Ribonucleotide reductase of class Ia (aerobic), beta subunit (EC 1.17.4.1)		
1	CDS	1810302	1810580	+	atggctgcaatcaaaatcaaca	MAAIKINKLLTIESNSSHTLLES	fig|127906.87.peg.1638	VCC1638	VC1254	ascD	YfaE: ferredoxin involved with ribonucleotide reductase diferric-tyrosyl radical (Y*) cofactor maintenance		
1	CDS	1811032	1810778	-	atgtttcaacactcgaaaatcttg	MFQHSKILVWYKIASQKVVLG	fig|127906.87.peg.1639	VCC1639	No refSeq annotation		FIG01199704: hypothetical protein	30S ribosomal protein S6 modification protein [Vibrio cholerae 2740-80]	
					ttggcgttgcagcttcaagtagga	aggggaaaagccattgaatatgaagttg	From N16961	Not annotated as CDS	VC1253				Only annotated as CDS in N16961 in KEGG. Not in PATRIC
1	CDS	1812520	1811282	-	atgttgaaaatagcaatgctcag	MLKIAMLSTGEEVLHGDIVDSN	fig|127906.87.peg.1640	VCC1640	VC1252	yfaY	ADP-ribose pyrophosphatase of COG1058 family (EC 3.6.1.13) / Nicotinamide-nucleotide amidase paralog YfaY, no functional activity		
1	CDS	1812693	1813592	+	gtgctcaagaaaaacatcacct	MLKKNITSMMLLTFSACVSAQ	fig|127906.87.peg.1641	VCC1641	VC1250		Ubiquitin-protein ligase		
1	CDS	1813858	1814370	+	atgaacagcacatttattgtgaa	MNSTFIVNFVGKATPATIKQLA	fig|127906.87.peg.1642	VCC1642	VC1249		Glycine cleavage system regulatory protein		
1	CDS	1814601	1816211	+	atgttgaagctagagaatatcaa	MLKLENIKVSYKLAAIVIVGIVG	fig|127906.87.peg.1643	VCC1643	VC1248		Methyl-accepting chemotaxis sensor/transducer protein		
1	CDS	1816304	1816654	+	atgagcaaattaacggcagata	MSKLTADIEANLALFIQETQES	fig|127906.87.peg.1644	VCC1644	VC1247		hypothetical protein	DUF2750 domain-containing protein [Vibrio cholerae]	
1	CDS	1816833	1817408	+	atgcaattaactaagctgacgcc	MQLTKLTQEIFDHLYRDISEFR	fig|127906.87.peg.1645	VCC1645	VC1246		hypothetical protein	nucleoside triphosphate pyrophosphohydrolase family protein [Vibrio cholerae]	
1	CDS	1818548	1817793	-	atgattcgtgttaacagcttacag	MIRVNSLQVDSRLLPLSLQAN	fig|127906.87.peg.1646	VCC1646	VC1245	btuD	Vitamin B12 ABC transporter, ATP-binding protein BtuD		
1	CDS	1819542	1818535	-	atgaacgacaagatggattttca	MNDKMDFHRLLQHKQQRWQ	fig|127906.87.peg.1647	VCC1647	VC1244	btuC	Vitamin B12 ABC transporter, permease protein BtuC		
1	CDS	1820639	1819611	-	atgacgaagtctcttccgcca	MTKSLFRQSFLFDSLDLDHPM	fig|127906.87.peg.1648	VCC1648	VC1242	astE	Succinylglutamate desuccinylase (EC 3.5.1.96)		
1	CDS	1821557	1820955	-	atgatccgaatttggttactgcgt	MIRIWLLRHGKTQGPAALNGI	fig|127906.87.peg.1649	VCC1649	VC1240	cobC	Alpha-ribazole-5'-phosphate phosphatase (EC 3.1.3.73)		
1	CDS	1822103	1821564	-	gtgacggttcatttcgttttaggtg	MTVHFVLGGARSGKSSYAEL	fig|127906.87.peg.1650	VCC1650	VC1239	cobP/cobU	Adenosylcobinamide kinase (EC 2.7.1.156) / Adenosylcobinamide-phosphate guanylyltransferase (EC 2.7.7.62)		
1	CDS	1822897	1822112	-	atggcggccatttacgttatcaa	MAAILRYQLELFLLAVSFFSRI	fig|127906.87.peg.1651	VCC1651	VC1238	cobS	Cobalamin synthase (EC 2.7.8.26)		
1	CDS	1823922	1822897	-	atgttagatcgctcattcagcgc	MLDRSFSAEIQQRIDQKTKPL(	fig|127906.87.peg.1652	VCC1652	VC1237	cobT	Nicotinate-nucleotide--dimethylbenzimidazole phosphoribosyltransferase (EC 2.4.2.21)		
1	CDS	1824319	1824206	-	ttgcctttttattggcaggttattc	MPFLLAGLFGFGFGDLWLAIM	fig|127906.87.peg.1653	VCC1653	No refSeq annotation		FIG01203663: hypothetical protein	putative membrane protein [Vibrio cholerae BJG-01]	
1	repeat	1824403	1824532	+	ttatacccaaacaacttggagttg	caggtaggcggcaagtaagtgaatccc	fig|127906.87.repeat.34				repeat region		
1	CDS	1824979	1824533	-	atgttgaactggcaacaggtgtt	MLNWQQVLDFANQGNPKPER	fig|127906.87.peg.1654	VCC1654	VC1236		Peptide-methionine (R)-S-oxide reductase MsrB (EC 1.8.4.12)		
1	CDS	1825325	1826698	+	atgtcagtggttaccctgcagca	MSVVTLAALVIFAGLLLYLFGL	fig|127906.87.peg.1655	VCC1655	VC1235	tcyP	L-cystine uptake protein TcyP, sodium:anion symporter family		
1	CDS	1826844	1828268	+	atgcagcaagaacaccagccc	MQQEHQPTFFFFDYETWGVN	fig|127906.87.peg.1656	VCC1656	VC1234	sbcB	Exodeoxyribonuclease I (EC 3.1.11.1)		
1	CDS	1828333	1828674	+	atgggactgatttttctctgtctgtt	MGLIFLCLLAGINLQTWLGIAIF	fig|127906.87.peg.1657	VCC1657	VC1233		UPF0299 membrane protein YohJ	CidA/LrgA family protein [Vibrio cholerae]	
1	CDS	1828665	1829339	+	atgtggctgatcatcacacttgg	MWLIITLGVFFVARWVAQKCT	fig|127906.87.peg.1658	VCC1658	VC1232		Inner membrane protein YohK	LrgB family protein [Vibrio cholerae]	
1	CDS	1829509	1830396	+	atgagaaaccgtattgaacaag	MRNRIEQALQQMPASFAPYLF	fig|127906.87.peg.1659	VCC1659	VC1231	cdd	Cytidine deaminase (EC 3.5.4.5)		
1	CDS	1830555	1831430	+	atgtttgaacaaatcattgccatt	MFEQIIAILFPVMSLVLIGYLIGL	fig|127906.87.peg.1660	VCC1660	VC1229		Membrane protein	AEC family transporter [Vibrio cholerae]	
1	CDS	1831584	1832759	+	atgtttgggaccgcgactcgggt	MFGTATRDNATRVLLLGSGEL	fig|127906.87.peg.1661	VCC1661	VC1228	purT	Formate-dependent phosphoribosylglycinamide formyltransferase		
					gtgtgcgaaagctgtgaagattc	attactaattcagctcttatcttcatcagcac	From N16961	Not annotated as CDS	VC1227		hypothetical protein		Only annotated as CDS in N16961 in KEGG. Not in PATRIC
1	CDS	1833508	1832852	-	atgcgagatcctgagttttggcat	MRDPEFWHNKWAANQIGFHL	fig|127906.87.peg.1662	VCC1662	VC1226	tpm	Thiopurine S-methyltransferase (EC 2.1.1.67)		
1	CDS	1833776	1833910	+	ttgcagcttcaaataggaagaa	MQLQIGRRYVMDNKFLQLKIQ	fig|127906.87.peg.1663	VCC1663	Not present		hypothetical protein		
1	CDS	1833977	1834756	+	atgagtctattgctacgtaccac	MSLLLRTTALMLLLLSRAPAMA	fig|127906.87.peg.1664	VCC1664	VC1224		hypothetical protein	Phosphoribosylglycinamide formyltransferase [Vibrio cholerae]	
					atgcgcaaattgccaagatgcga	tcagcctcacgcgatctctatcaacaagg	From N16961	Not annotated as CDS	VC1223		hypothetical protein		Only annotated as CDS in N16961 in KEGG. Not in PATRIC
1	CDS	1835181	1834885	-	atggcgctcacaaaggccgaa	MALTKAELAEALFEQLGMSKR	fig|127906.87.peg.1665	VCC1665	VC1222	ihfA	Integration host factor alpha subunit		
1	CDS	1838007	1835620	-	atgaaattcagcgaatcatggc	MKFSESWLREWVNPAITTDEL	fig|127906.87.peg.1666	VCC1666	VC1220	pheT	Phenylalanyl-tRNA synthetase beta chain (EC 6.1.1.20)		
1	CDS	1839017	1838034	-	atgcaacatctacaagagattat	MQHLQEIIANATSAINAAESLV	fig|127906.87.peg.1667	VCC1667	VC1219	pheS	Phenylalanyl-tRNA synthetase alpha chain (EC 6.1.1.20)		
1	CDS	1839165	1839049	-	ttggctttaatcttggctttcggtac	MALILAFGTMYSYLKTPRIPYG	fig|127906.87.peg.1668	VCC1668	VC1218		FIG01202210: hypothetical protein		
1	CDS	1839308	1839793	+	atgagagtagaaatcgtaccga	MRVEIVPIRPEFDAEICQIIQSV	fig|127906.87.peg.1669	VCC1669	VC1217		Histone acetyltransferase HPA2		
1	CDS	1840908	1839790	-	atgcaatcattcaaatgggatca	MQSFKWDQYFETGLEEVDEQ	fig|127906.87.peg.1670	VCC1670	VC1216		GGDEF domain protein		
1	rna	1841230	1841155	-	gcgcacactagctcagttggtag	agcgcaaccttgccaaggttgaggtcacgg	fig|127906.87.rna.49	VCCt036	VCt059		tRNA-Gly-GCC		
1	rna	1841327	1841241	-	gccctggtggtgaaatcggtaga	cacaaggggatttaaaatccctcgacttcg	fig|127906.87.rna.50	VCCt037	VCt058		tRNA-Leu-TAA		
1	rna	1841467	1841392	-	gcgcacactagctcagttggtag	agcgcaaccttgccaaggttgaggtcacgg	fig|127906.87.rna.51	VCCt038	VCt057		tRNA-Gly-GCC		
1	rna	1841543	1841470	-	ggcgcgttggcagagtggccatg	cagcggattgcaaaatccgttggacctcgg	fig|127906.87.rna.52	VCCt039	VCt056		tRNA-Cys-GCA		
1	CDS	1842358	1841801	-	atgcgtttcaacattcctaaccttc	MRFNIPNLLSLLRLFLIPVFVVT	fig|127906.87.peg.1671	VCC1671	VC1215	pgsA	CDP-diacylglycerol--glycerol-3-phosphate 3-phosphatidyltransferase (EC 2.7.8.5)		

1	CDS	1844238	1842406	-	gtgtcgacacagtttgattctgcc	MSTQFDSAPFLKTVTNQPGVY	fig|127906.87.peg.1672	VCC1672	VC1214	uvrC	Excinuclease ABC subunit C		
1	CDS	1844882	1844238	-	ttgattagtgttttccttgtagatgat	MISVFLVDDHELVRTGIRRIIED	fig|127906.87.peg.1673	VCC1673	VC1213	uvrY	BarA-associated response regulator UvrY (= GacA = SirA)		Limited query cover for certain strains
1	CDS	1845258	1847621	+	ttgaagattgaacaagggtttgta	MKIEQGFVLTRHARDVAGQT	fig|127906.87.peg.1674	VCC1674	VC1212	polB	DNA polymerase II (EC 2.7.7.7)		
1	CDS	1849692	1847593	-	atgctgagttttaataagtacttac	MLSFNKYLPTVRVKNELTKVSQ	fig|127906.87.peg.1675	VCC1675	VC1211		PTS system, cellobiose-specific IIC component		
1	CDS	1850568	1849801	-	atgactctgccggtgattgaccc	MTLPVIDPKQPFQPEYQNAIAD	fig|127906.87.peg.1676	VCC1676	VC1210		hypothetical protein	Nucleotidyltransferase [Vibrio cholerae]	
1	CDS	1850787	1851353	+	atgccaaaggcaagtgaactca	MPKASELKKGFAIISNGKTLLIK	fig|127906.87.peg.1677	VCC1677	VC1209	yeiP	Elongation factor P-like protein		
1	CDS	1851358	1851678	+	atggctgagcttatttctatcgacc	MAELISIDDTIDTAYDIFLEMAP	fig|127906.87.peg.1678	VCC1678	VC1208		dsDNA mimic protein, highly acidic	YciU family protein [Vibrio cholerae]	
1	CDS	1851817	1852764	+	atgcgattcatcccattgcttagcc	MRFIPLLSLICTLVAFPSLANEY	fig|127906.87.peg.1679	VCC1679	VC1207		FIG01199745: hypothetical protein	DUF3187 family protein [Vibrio cholerae]	
1	CDS	1852881	1853591	+	ttgtctccacgctatctgcaaatca	MSPRYLQIKQFMLDNIESGRW	fig|127906.87.peg.1680	VCC1680	VC1206	frlR/hutC	Histidine utilization repressor		
1	CDS	1853695	1854903	+	atgaattgtgtgttgaccgaagcc	MNCVLTEARLVTMQPGVQGY	fig|127906.87.peg.1681	VCC1681	VC1205	hutI	Imidazolonepropionase (EC 3.5.2.7)		
1	CDS	1854893	1855903	+	atgaatcctaatttcaccactgag	MNPNFTTEHTWQGRHDPED	fig|127906.87.peg.1682	VCC1682	VC1204	hutG	Formiminoglutamase (EC 3.5.3.8)		
1	CDS	1855922	1857619	+	atgacacagtcatctgcacaag	MTQSSAQGTRLDTQRTIRAPR	fig|127906.87.peg.1683	VCC1683	VC1203	hutU	Urocanate hydratase (EC 4.2.1.49)		
1	CDS	1857631	1859166	+	atgttgcacctgatgatcaaacc	MLHLMIKPGQLSLKQLRQVSR	fig|127906.87.peg.1684	VCC1684	VC1202	hutH	Histidine ammonia-lyase (EC 4.3.1.3)		
1	CDS	1859315	1860040	+	atgttcctgactgcataccattca	MFLTAYHSNQQHQFQFTRQQ	fig|127906.87.peg.1685	VCC1685	VC1201		FIG01199954: hypothetical protein	DUF3581 domain-containing protein [Vibrio cholerae]	
1	CDS	1861326	1860115	-	gtgcatcaggtttctaaattgctgt	MHQVSKLLSCFIGFSLFSTLLY	fig|127906.87.peg.1686	VCC1686	VC1200		Secreted trypsin-like serine protease		
1	CDS	1862037	1861576	-	atgaaaaacttagaattaaagg	MKNLELKVPPVAVFLVALALIH	fig|127906.87.peg.1687	VCC1687	VC1199		Putative protein-S-isoprenylcysteine methyltransferase		
1	CDS	1865128	1862093	-	atgttaccaagactccatcacca	MLPRLHHQSDVDPVVLTFLHE	fig|127906.87.peg.1688	VCC1688	VC1198		Uncharacterized protein YdiJ	FAD-binding oxidoreductase [Vibrio cholerae]	
1	CDS	1865329	1866024	+	atggaaaaaacgatgaaaaaa	MEKTMKKNQIVTTEDILLMLC	fig|127906.87.peg.1689	VCC1689	VC1197		hypothetical protein	DUF3334 family protein [Vibrio cholerae]	
1	CDS	1866155	1866838	+	atggataaacaaaaagcccta	MDKQKALKKIAKCLELGNSAN	fig|127906.87.peg.1690	VCC1690	VC1196		hypothetical protein	DUF2786 domain-containing protein [Vibrio cholerae]	
1	CDS	1866938	1867609	+	gtgttgaacaaaaccactttgctg	MLNKTTLLLAAVVALSGCQAT	fig|127906.87.peg.1691	VCC1691	VC1195		Probable lipoprotein YiaD	OmpA family protein [Vibrio cholerae]	
1	CDS	1867710	1868726	+	atgcgtttgcggagtttatggctgt	MRLRSLWLFLLFSLPLAAADD	fig|127906.87.peg.1692	VCC1692	VC1194		hypothetical protein	J domain-containing protein [Vibrio cholerae]	
1	CDS	1868877	1869059	+	atgtttagcgtagaaggcatctgt	MFSVEGICDWCKQPKLLTRHE	fig|127906.87.peg.1693	VCC1693	VC1193		hypothetical protein		
1	CDS	1869351	1869749	+	atgctacacactgaattcaaaca	MLHTEFKHFGLHNSPTLSALC	fig|127906.87.peg.1694	VCC1694	VC1191		Superfamily II DNA and RNA helicase		Limited query cover for certain strains
1	CDS	1869915	1871021	+	atgagccttgcagatcaagtcct	MSLADQVLAVNDDLPIRTHQP	fig|127906.87.peg.1695	VCC1695	VC1190	purC/hemH	Phosphoribosylaminoimidazole-succinocarboxamide synthase (EC 6.3.2.6)		
1	CDS	1871573	1871223	-	gtgttcgattacaccaccttttgg	MFDYTTFLGASCAKKWTFSE	fig|127906.87.peg.1696	VCC1696	VC1189		Uncharacterized protein VC1189	transporter [Vibrio cholerae]	
1	CDS	1872039	1873727	+	atgaataacgataaacgtccgc	MNNDKRPLYISYAGPALLSTP	fig|127906.87.peg.1697	VCC1697	VC1188	maeA/sfcA	NAD-dependent malic enzyme (EC 1.1.1.38)		
1	CDS	1873770	1874099	+	atgcaactatttacttatcttgctcc	MQLFTYLAPAGQALLPYLSEIS	fig|127906.87.peg.1698	VCC1698	VC1187		hypothetical protein	DUF3392 domain-containing protein [Vibrio cholerae]	
1	CDS	1874072	1874836	+	atgggcacaaagaaaccatca	MGTKKPSNLTLRAVVFSALAH	fig|127906.87.peg.1699	VCC1699	VC1186		SanA protein		
1	CDS	1876268	1874937	-	gtgaaagtgctgccaagagtgtt	MKVLPRVLLGLVAVTIVTVLLY	fig|127906.87.peg.1700	VCC1700	VC1185		diguanylate cyclase (GGDEF domain)		
1	CDS	1877767	1876526	-	atgcgcttcactcctgagtcagtg	MRFTPESVRQQFPALAQIVND	fig|127906.87.peg.1701	VCC1701	VC1184	csd	Cysteine desulfurase (EC 2.8.1.7)		
1	CDS	1878407	1877751	-	atgttacgttctcaccccaatacc	MLRSHPNTLHITKLSHSAIHQL	fig|127906.87.peg.1702	VCC1702	VC1183		FIG010427: hypothetical protein	2OG-Fe dioxygenase family protein [Vibrio cholerae]	
1	CDS	1878885	1879841	+	atgagtaacgtgaaacacagca	MSNVKHSKLLILGSGPAGYTA	fig|127906.87.peg.1703	VCC1703	VC1182	trxB	Thioredoxin reductase (EC 1.8.1.9)		
1	CDS	1879961	1881748	+	atggacaagaaaaaacagcgt	MDKKKQRSLNLWLKQQSKLA	fig|127906.87.peg.1704	VCC1704	VC1181	cydD	Efflux ABC transporter for glutathione/L-cysteine, essential for assembly of bd-type respiratory oxidases => CydD subunit		
1	CDS	1881741	1883462	+	atgcgtgacctgctgccttaccta	MRDLLPYLKLYKKHWFGLTLG	fig|127906.87.peg.1705	VCC1705	VC1180	cydC	Efflux ABC transporter for glutathione/L-cysteine, essential for assembly of bd-type respiratory oxidases => CydC subunit		
1	CDS	1884595	1883630	-	atgagcgaaaagttacaaaaa	MSEKLQKVLARAGHGSRREL	fig|127906.87.peg.1706	VCC1706	VC1179	rluB	LSU rRNA pseudouridine(2605) synthase (EC 5.4.99.22)		
1	CDS	1885348	1884728	-	atgagccagtttttttatgttcatcc	MSQFFYVHPENPQTRLINQAV	fig|127906.87.peg.1707	VCC1707	VC1178		YciO protein, TsaC/YrdC paralog		
1	CDS	1886223	1885351	-	atgagaattgacctgcatagtca	MRIDLHSHTTASDGRFTYQQL	fig|127906.87.peg.1708	VCC1708	VC1177		FIG00031715: Predicted metal-dependent phosphoesterases (PHP family)		
1	CDS	1886288	1886425	+	atgcaagactctctgttagggctc	MQDSLLGLTMLQEFNPNHKP	fig|127906.87.peg.1709	VCC1709	VC1176		Trp operon leader peptide		
1	CDS	1886559	1888130	+	atgaataaggccatcaacataa	MNKAINIKKTAPLEVLHSELPY	fig|127906.87.peg.1710	VCC1710	VC1174	trpE	Anthranilate synthase, aminase component (EC 4.1.3.27)		
1	CDS	1888133	1888738	+	atggccaatattctgtttatcgata	MANILFIDNFDSFTYNLVDQFR	fig|127906.87.peg.1711	VCC1711	VC1173	trpG	Anthranilate synthase, amidotransferase component (EC 4.1.3.27)		
1	CDS	1888759	1889757	+	atgcaaaccattattaataaactc	MQTIINKLYEQQGLTQAESQQ	fig|127906.87.peg.1712	VCC1712	VC1172	trpD	Anthranilate phosphoribosyltransferase (EC 2.4.2.18)		
1	CDS	1889763	1891172	+	atgtccgagcagttgtctgagca	MSEQLSEHISVQTAQMAQVLV	fig|127906.87.peg.1713	VCC1713	VC1171	trpC	Indole-3-glycerol phosphate synthase (EC 4.1.1.48) / Phosphoribosylanthranilate isomerase (EC 5.3.1.24)		
1	CDS	1891213	1892403	+	atggctaaattaaacgcctattttg	MAKLNAYFGEFGGQFVPQILV	fig|127906.87.peg.1714	VCC1714	VC1170	trpB	Tryptophan synthase beta chain (EC 4.2.1.20)		
1	CDS	1892403	1893209	+	atgaaccgttatcaagccctattt	MNRYQALFQRLSAAQQGAFV	fig|127906.87.peg.1715	VCC1715	VC1169	trpA	Tryptophan synthase alpha chain (EC 4.2.1.20)		
1	CDS	1893405	1894781	+	atgtgcttattgctaaatgtaaaca	MCLLLNVNIPCKKQEQNFCPF	fig|127906.87.peg.1716	VCC1716	VC1168		Proton/glutamate symporter @ Sodium/glutamate symporter		
1	CDS	1895461	1894883	-	ttggctcagatgtacttttattactc	MAQMYFYYSAMNAGKSTTLL	fig|127906.87.peg.1717	VCC1717	VC1167	tdk	Thymidine kinase (EC 2.7.1.21)		
1	CDS	1897333	1895558	-	atgcgtagccattattgtggtcac	MRSHYCGHLNKSLVGQTVEL	fig|127906.87.peg.1718	VCC1718	VC1166	aspS	Aspartyl-tRNA synthetase (EC 6.1.1.12)		
1	CDS	1897625	1898494	+	gtgacgtcctctcttcccttgcgac	MTSSLPLRLGLTLWSHPAWQ	fig|127906.87.peg.1719	VCC1719	VC1165		UPF0759 protein YecE	DUF72 domain-containing protein [Vibrio cholerae]	
1	CDS	1898632	1899360	+	atgaacccaaaagatactctctt	MNPKDTLFSAPIDKIGDFTFDE	fig|127906.87.peg.1720	VCC1720	VC1164	cmoA	Carboxy-S-adenosyl-L-methionine synthase		
1	CDS	1899472	1900443	+	atgttcaactttgccaacgtttacc	MFNFANVYQQIAQHPQLQLW	fig|127906.87.peg.1721	VCC1721	VC1163	cmoB	tRNA ho5U(34) carboxymethyltransferase		
1	CDS	1900563	1901300	+	atgtttaagcgaattgcacccttt	MFKRIAPFAALSLLSGCALTN	fig|127906.87.peg.1722	VCC1722	VC1162		FIG008443: hypothetical protein	ATP-dependent Zn protease [Vibrio cholerae]	
1	CDS	1901309	1902817	+	atgacttcgaaaattcctttttatat	MTSKIPFYISVFLLFATGITLSV	fig|127906.87.peg.1723	VCC1723	VC1161		FIG139976: hypothetical protein	inactive transglutaminase family protein [Vibrio cholerae]	
1	CDS	1902817	1903806	+	atggggttctccttttcgttctcgga	MGFSFSFSDYTSPFKLRRKGI	fig|127906.87.peg.1724	VCC1724	VC1160		FIG002781: Alpha-L-glutamate ligase family protein		
1	CDS	1904981	1903887	-	atggagcacaacaccgatacg	MEHNTDTVYNFSAGPAALPK	fig|127906.87.peg.1725	VCC1725	VC1159	serC	Phosphoserine aminotransferase (EC 2.6.1.52)		
1	CDS	1906368	1905103	-	atgaaatcaataaaaatgatcg	MKSIKMIGAVGGAVALGLCWF	fig|127906.87.peg.1726	VCC1726	VC1158		hypothetical protein	YdgA family protein [Vibrio cholerae]	
1	CDS	1906591	1908546	+	gtgaatcgctacgccctgctcatt	MNRYALLILDNNPFSIEQWRQ	fig|127906.87.peg.1727	VCC1727	VC1157		Two-component system sensor histidine kinase		ORF split into 2 ORFs in N16961, both with refseq in PATRIC. Annotated as 1 pseudogene ORF named VC1156 in KEGG.
									VC1156				ORF split into 2 ORFs in N16961, both with refseq in PATRIC. Annotated as 1 pseudogene ORF named VC1156 in KEGG.
1	CDS	1908543	1909040	+	atgaacaaattttttgattttatgtgt	MNKFLILCVDDEREVLDSVVQ	fig|127906.87.peg.1728	VCC1728	VC1155		Response regulator		
1	CDS	1909231	1909779	+	atgcgtaaaacaatcgttgcaac	MRKTIVATALLLVAGSALAEG	fig|127906.87.peg.1729	VCC1729	VC1154		hypothetical protein		
1	CDS	1910462	1909875	-	atgaatgagcaacagtttttcgac	MNEQQFFDYVTKFGAYQKRS	fig|127906.87.peg.1730	VCC1730	VC1153		Regulator of competence-specific genes		
1	CDS	1910840	1911760	+	atgagtcacctatctttttactggtt	MSHLSFYWLPETKHKLIQGLE	fig|127906.87.peg.1731	VCC1731	VC1152		Predicted signal transduction protein	HDOD domain-containing protein [Vibrio cholerae]	
1	CDS	1911781	1912686	+	atgctgtcagggatgatctttgttt	MLSGMIFVFAPLVVGYLFIIRN	fig|127906.87.peg.1732	VCC1732	VC1151		Lysine exporter LysO		
1	CDS	1912875	1913384	+	atgaaacgcctgagcactgtttta	MKRLSTVLLMSVVSASAYADN	fig|127906.87.peg.1733	VCC1733	VC1150		hypothetical protein		
1	CDS	1913494	1915140	+	atggtatcagaacacaaatccg	MVSEHKSAQVNFDSLLKIFTV	fig|127906.87.peg.1734	VCC1734	VC1149		Glutamate decarboxylase, eukaryotic type (EC 4.1.1.15)		
1	CDS	1915448	1916302	+	atgaatacattagaaaaaattca	MNTLEKIQKNLENFSKSERKV	fig|127906.87.peg.1735	VCC1735	VC1148		Phosphogluconate repressor HexR, RpiR family		
1	CDS	1917640	1916393	-	atgttccagtttatgaccgcgaca	MFQFMTATRIIFGEGSLESSLS	fig|127906.87.peg.1736	VCC1736	VC1147	dhaT	Iron-containing alcohol dehydrogenase (EC 1.1.1.1)		
1	CDS	1917949	1918212	+	atgtttgtggtcatttttggtcgtcct	MFVVIFGRPGCPYCVRAKEHA	fig|127906.87.peg.1737	VCC1737	VC1146	grxA	Glutaredoxin 1		
1	CDS	1918414	1920090	+	gtgaacatcgacgtcgttttgttgc	MNIDVVLLLEQNPILLIFVVLAI	fig|127906.87.peg.1738	VCC1738	VC1145	aspT	Putative transport protein YbjL		
1	CDS	1922429	1920159	-	atgcttaacaaagaattagagtc	MLNKELESSLNGAFARARDK	fig|127906.87.peg.1739	VCC1739	VC1144	clpA	ATP-dependent Clp protease ATP-binding subunit ClpA		
1	CDS	1922788	1922468	-	atgagtaaaaactttgaatggat	MSKNFEWISPDFDLLEKEKTA	fig|127906.87.peg.1740	VCC1740	VC1143	clpS	ATP-dependent Clp protease adaptor protein ClpS		
1	CDS	1923261	1923482	+	atggctacaggtacagtgaaatc	MATGTVKWFNNAKGFGFICP	fig|127906.87.peg.1741	VCC1741	VC1142	cspD	Cold shock protein of CSP family => CspD (naming convention as in E.coli)		
1	CDS	1925806	1923581	-	atgcctaccaataaacctacaat	MPTNKPTIIYTITDEAPALATYS	fig|127906.87.peg.1742	VCC1742	VC1141	icd	Isocitrate dehydrogenase [NADP] (EC 1.1.1.42); Monomeric isocitrate dehydrogenase [NADP] (EC 1.1.1.42)		
1	CDS	1926114	1926839	+	atgtcatctcgcttgtccagcgat	MSSRLSSDATSSSSDQRKSH	fig|127906.87.peg.1743	VCC1743	VC1140	rluE	LSU rRNA pseudouridine(2457) synthase (EC 5.4.99.20)		
1	repeat	1926859	1926983	+	tatatagccaaactacttggagttgcaggtaggcggcaaatgagtgaatccc		fig|127906.87.repeat.35				repeat region		
1	CDS	1927672	1927040	-	atgaatccagcaagcccatttgc	MNPASPFATLTDRIDWQKVD	fig|127906.87.peg.1744	VCC1744	VC1139	hisE/hisI	Phosphoribosyl-AMP cyclohydrolase (EC 3.5.4.19) / Phosphoribosyl-ATP pyrophosphatase (EC 3.6.1.31)		
1	CDS	1928442	1927669	-	atgttggcaaaacggataattcc	MLAKRIIPCLDVRDGQVVKGV	fig|127906.87.peg.1745	VCC1745	VC1138	hisF	Imidazole glycerol phosphate synthase cyclase subunit		
1	CDS	1929161	1928424	-	atgattattcccgcattagatttga	MIIPALDLIEGQVVRLYQGDY	fig|127906.87.peg.1746	VCC1746	VC1137	hisA	Phosphoribosylformimino-5-aminoimidazole carboxamide ribotide isomerase (EC 5.3.1.16)		
1	CDS	1929815	1929204	-	atgactcagaatgtggtgattatt	MTQNVVIIDTGCANISSVKFAI	fig|127906.87.peg.1747	VCC1747	VC1136	hisH	Imidazole glycerol phosphate synthase amidotransferase subunit HisH		
1	CDS	1930888	1929815	-	gtgagcaaacaacaaaaaattc	MSKQQKILFIDRDGTLIVEPPV	fig|127906.87.peg.1748	VCC1748	VC1135	hisB	Histidinol-phosphatase (EC 3.1.3.15) / Imidazoleglycerol-phosphate dehydratase (EC 4.2.1.19)		
1	CDS	1931980	1930940	-	atggaaaaactggctcgtcaac	MEKLARQQIQALTPYLSARRI	fig|127906.87.peg.1749	VCC1749	VC1134	hisC	Histidinol-phosphate aminotransferase (EC 2.6.1.9)		
1	CDS	1933279	1931984	-	atgagaaccgtggtttggcaatc	MRTVVWQSLSEAQQESILER	fig|127906.87.peg.1750	VCC1750	VC1133	hisD	Histidinol dehydrogenase (EC 1.1.1.23)		
1	CDS	1934180	1933284	-	atgcaaacacaacgactaaga	MQTQRLRIAIQKKGRLSQECQ	fig|127906.87.peg.1751	VCC1751	VC1132	hisG	ATP phosphoribosyltransferase (EC 2.4.2.17) => HisGl		
1	CDS	1936190	1934589	-	atgaatttaatcgatttgcttcttcg	MNLIDFASSPLSVLPPLVALSL	fig|127906.87.peg.1752	VCC1752	VC1131		Tetracycline resistance, MFS efflux pump => Tet(35)		
1	CDS	1936884	1937291	+	atgtcggaaatcactaagacttta	MSEITKTLLNIRSLRAYARELT	fig|127906.87.peg.1753	VCC1753	VC1130	hns/vicH	DNA-binding protein H-NS		
1	CDS	1938643	1937339	-	atgaaatttcctggccaacgcaa	MKFPGQRKSKHYFPVHARDP	fig|127906.87.peg.1754	VCC1754	VC1129		Inosine-guanosine kinase (EC 2.7.1.73)		
1	CDS	1938908	1940023	+	atgtctgtagagcacagcgcaa	MSVEHSANSQKKVIVGMSGG	fig|127906.87.peg.1755	VCC1755	VC1128	mnmA	tRNA-specific 2-thiouridylase MnmA (EC 2.8.1.13)		
1	CDS	1940046	1940663	+	gtggctaacgcaatttacgaccg	MANAIYDRTIAFAGICQAVALV	fig|127906.87.peg.1756	VCC1756	VC1127	hflD	High frequency lysogenization protein HflD		
1	CDS	1940721	1942091	+	atggaactgtcagcattgactgc	MELSALTAVSPVDGRYGSKT	fig|127906.87.peg.1757	VCC1757	VC1126	purB	Adenylosuccinate lyase (EC 4.3.2.2) @ SAICAR lyase (EC 4.3.2.2)		
1	CDS	1942709	1942182	-	atgagattggctaaatgttggcta	MRLAKCWLLLLCIWLSGCSAE	fig|127906.87.peg.1758	VCC1758	VC1125		FIG002577: Putative lipoprotein precursor		
1	CDS	1943242	1942706	-	ttgctcgcgagcggacttggttac	MLASGLGYAAVANAAQAEHK	fig|127906.87.peg.1759	VCC1759	VC1124		FIG026291: Hypothetical periplasmic protein	Putative periplasmic protein [Vibrio cholerae]	
1	CDS	1943744	1943205	-	atggcgttagtggcgatatcatta	MALVAISLWFDLLWALAVLGQ	fig|127906.87.peg.1760	VCC1760	VC1123		FIG024285: Hypothetical protein	DUF2878 domain-containing protein [Vibrio cholerae]	
1	CDS	1944988	1943747	-	atgttaaatagctcgtcgatggaa	MLNSSSMEISRSLTSWQKGA	fig|127906.87.peg.1761	VCC1761	VC1122	cfa	S-adenosyl-L-methionine dependent methyltransferase, similar to cyclopropane-fatty-acyl-phospholipid synthase		
1	CDS	1945787	1944978	-	atgcctgtgccccaagatagcca	MPVPQDSTEWQSCLMVGQV	fig|127906.87.peg.1762	VCC1762	VC1121		FIG001571: Hypothetical protein	DUF1365 domain-containing protein [Vibrio cholerae]	
1	CDS	1947064	1945787	-	atgaaaattgcaatcattggctcg	MKIAIIGSGISGLTCGYYLHRY	fig|127906.87.peg.1763	VCC1763	VC1120		Amine oxidase, flavin-containing		
1	CDS	1947803	1947078	-	atgaccgcggtgttcattaccgg	MTAVFITGATSGIGKQLALDYA	fig|127906.87.peg.1764	VCC1764	VC1119	sdh	Oxidoreductase, short-chain dehydrogenase/reductase family		
1	CDS	1948219	1947800	-	atggacgtacaacacgtcgcgg	MDVQHVAQFYQQLDKSQLHR	fig|127906.87.peg.1765	VCC1765	VC1118		FIG002994: Putative transcriptional regulator	nuclear transport factor 2 family protein [Vibrio cholerae]	
1	CDS	1949301	1948438	-	atgaaacgtatattgttattcttag	MKRILLFLATNLAVVLVLSVVL	fig|127906.87.peg.1766	VCC1766	VC1117	htpX	Protease HtpX		
1	CDS	1949567	1949737	+	atggctgaagcacatagctacc	MAEAHSYQMAIDLLCCHLGIS	fig|127906.87.peg.1767	VCC1767	VC1116		hypothetical protein		
1	CDS	1950514	1949819	-	atgagaaatgcaatttttatcgca	MRNAIFIAGTDTDVGKTVASK	fig|127906.87.peg.1768	VCC1768	VC1115	bioD	Dethiobiotin synthase BioD (EC 6.3.3.3)		
1	CDS	1951308	1950511	-	atgacagccacggagctttgtgt	MTATELCVELKDKSAIAQAFG	fig|127906.87.peg.1769	VCC1769	VC1114	bioC	Malonyl-[acyl-carrier protein] O-methyltransferase (EC 2.1.1.197)		
1	CDS	1952465	1951311	-	atgacgctgcgctttaagtcgcg	MTLRFKSRIEQALNEREHSGL	fig|127906.87.peg.1770	VCC1770	VC1113	bioF	8-amino-7-oxononanoate synthase (EC 2.3.1.47)		
1	CDS	1953501	1952449	-	gtggaagttcgtcataactggac	MEVRHNWTVAEVKALLDKPF	fig|127906.87.peg.1771	VCC1771	VC1112	bioB	Biotin synthase (EC 2.8.1.6)		
1	CDS	1953634	1954914	+	atggatttagccttcgaccgttgc	MDLAFDRCHIWHPYTSTLTPL	fig|127906.87.peg.1772	VCC1772	VC1111	bioA	Adenosylmethionine-8-amino-7-oxononanoate aminotransferase (EC 2.6.1.62)		
1	CDS	1956328	1955021	-	atgctggattctaaattacttcgta	MLDSKLLRTELDETAAKLARR	fig|127906.87.peg.1773	VCC1773	VC1110	SerS	Seryl-tRNA synthetase (EC 6.1.1.11)		
1	CDS	1957781	1956432	-	gtgagtaattacagcttagattttt	MSNYSLDFSGDEDFRPLAAR	fig|127906.87.peg.1774	VCC1774	VC1108	rarA	Replication-associated recombination protein RarA		
1	CDS	1958402	1957806	-	atgaacaaatggttagccctgtta	MNKWLALLFCSFSAIAAPKDT	fig|127906.87.peg.1775	VCC1775	VC1107	lolA	Outer membrane lipoprotein carrier protein LolA		
1	CDS	1959118	1958498	-	atgaattattccctgatcgatctca	MNYSLIDLSAIECPESDLFIEG	fig|127906.87.peg.1776	VCC1776	VC1106		Protein of unknown function VC1106	YecA family protein [Vibrio cholerae]	
1	CDS	1959305	1960255	+	atggagggcgtgttagatcatttg	MEGVLDHLMRDMLTQINDYD	fig|127906.87.peg.1777	VCC1777	VC1105		tRNA-dihydrouridine(16) synthase		
1	CDS	1961757	1960288	-	atgaatcagcgcatgagtttaac	MNQRMSLTWIICAPLFIVFVLA	fig|127906.87.peg.1778	VCC1778	VC1104		GGDEF domain protein		
1	CDS	1962718	1961924	-	atgatccgtttgcaagatattcag	MIRLQDIQVTFNPGTILENRAL	fig|127906.87.peg.1779	VCC1779	VC1103	ssuB	ABC transporter, ATP-binding protein (cluster 15, trp?)		
1	CDS	1963659	1962715	-	atgtctgcttttgcctttttggcgct	MSAFAFFGALELGLLYGLVAL	fig|127906.87.peg.1780	VCC1780	VC1102		ABC transporter, permease protein (cluster 15, trp?)		
1	CDS	1964739	1963774	-	atgagagcaggcaaagttattg	MRAGKVIATAVLAGAALLSSQ	fig|127906.87.peg.1781	VCC1781	VC1101		ABC transporter, substrate-binding protein (cluster 15, trp?)		
1	CDS	1965177	1965587	+	atgaccgatcaagaaagaatcg	MTDQERIELQKNNPLHGLKLE	fig|127906.87.peg.1782	VCC1782	VC1100		hypothetical protein	DNA-binding protein VF530 [Vibrio cholerae]	
1	CDS	1966162	1965710	-	atgaataataaagtcggcatagt	MNNKVGIVHSLKDGQKYMDI	fig|127906.87.peg.1783	VCC1783	VC1099		UPF0208 membrane protein YfbV	DUF412 domain-containing protein [Vibrio cholerae]	
1	CDS	1966515	1967711	+	atgtctaagctggttttagttttaaa	MSKLVLVLNCGSSSLKFAVVD	fig|127906.87.peg.1784	VCC1784	VC1098		Acetate kinase (EC 2.7.2.1)		
1	CDS	1967847	1969991	+	atgtcccgtactattatgcttatcc	MSRTIMLIPISAGVGLTSVSMG	fig|127906.87.peg.1785	VCC1785	VC1097	pta	BioD-like N-terminal domain / Phosphate acetyltransferase (EC 2.3.1.8)		
1	CDS	1971036	1970089	-	atgggcaagctaattaaaggca	MGKLIKGKWHDVWYDTKQHD	fig|127906.87.peg.1786	VCC1786	VC1096	yqjG	Glutathione S-transferase, omega (EC 2.5.1.18)		
1	CDS	1972178	1971186	-	atgagcgtagagaaaaaattat	MSVEKKLLLDVKDLKVHFNIT	fig|127906.87.peg.1787	VCC1787	VC1095	oppF	Oligopeptide ABC transporter, ATP-binding protein OppF (TC 3.A.1.5.1)		
1	CDS	1973146	1972175	-	atgagtttattagatgtcaaagat	MSLLDVKDLRVEFSTQDGIVT	fig|127906.87.peg.1788	VCC1788	VC1094	oppD	Oligopeptide ABC transporter, ATP-binding protein OppD (TC 3.A.1.5.1)		
1	CDS	1974076	1973174	-	atgttgacgaaaaaagaaaatt	MLTKKENLAAIENFSQNLEIQG	fig|127906.87.peg.1789	VCC1789	VC1093	oppC	Oligopeptide ABC transporter, permease protein OppC (TC 3.A.1.5.1)		
1	CDS	1975010	1974090	-	atgcttaaattcatagcaaaaag	MLKFIAKRIFEAIPTMLVLITISF	fig|127906.87.peg.1790	VCC1790	VC1092	oppB	Oligopeptide ABC transporter, permease protein OppB (TC 3.A.1.5.1)		
1	CDS	1976790	1975159	-	atgtataagaacaaaatcaccc	MYKNKITQALLLGAGLAVATT	fig|127906.87.peg.1791	VCC1791	VC1091	oppA	Oligopeptide ABC transporter, substrate-binding protein OppA (TC 3.A.1.5.1)		
1	CDS	1977414	1978226	+	atgcgctacggttggtttatattca	MRYGWFIFIWCVSSFTWASPL	fig|127906.87.peg.1792	VCC1792	VC1089	phnD	ABC transporter, substrate-binding protein (cluster 12, methionine/phosphonates)		
1	CDS	1978230	1979834	+	atgtcgatacgcttaaagaccat	MSIRLKTMLGVALIEALLLTILIT	fig|127906.87.peg.1793	VCC1793	VC1088		Two-component system sensor histidine kinase		
1	CDS	1981200	1979863	-	atggataacatgcacgatgaga	MDNMHDEKLNVLLLDDENDIL	fig|127906.87.peg.1794	VCC1794	VC1087		Response regulator		
1	CDS	1982833	1981223	-	atgcaaagcaaccgtgaaattc	MQSNREILLVDDEPAVLNALK	fig|127906.87.peg.1795	VCC1795	VC1086		Response regulator		
1	CDS	1984137	1982833	-	atggtagacaaactacgcactt	MVDKLRTSGAAMREQENDCH	fig|127906.87.peg.1796	VCC1796	VC1085		Two-component system sensor histidine kinase		
1	CDS	1985468	1984149	-	atggcaaagagtcgtttattgctc	MAKSRLLLSELLDQLSFALCIV	fig|127906.87.peg.1797	VCC1797	VC1084	kinE	Two-component system sensor histidine kinase		
1	CDS	1986063	1985479	-	atgagcattgtactttccgcagat	MSIVLSADHKDALQEFMNISM	fig|127906.87.peg.1798	VCC1798	VC1083		Chemotaxis protein CheC -- inhibitor of MCP methylation		
1	CDS	1986425	1986060	-	atggcgattcgactaacggtagc	MAIRLTVADDSKMSRKSVIRAI	fig|127906.87.peg.1799	VCC1799	VC1082		Chemotaxis protein CheC -- inhibitor of MCP methylation		
1	CDS	1987602	1986460	-	atgacagtagaagcgatgaaa	MTVEAMKVVCVDDDDFMLKA	fig|127906.87.peg.1800	VCC1800	VC1081		Response regulator		
1	CDS	1987964	1987599	-	atggagcagagtgtcgcattaa	MEQSVAFNISTIAKMVGSDLV	fig|127906.87.peg.1801	VCC1801	VC1080		hypothetical protein	Hpt domain-containing protein [Vibrio cholerae]	
1	CDS	1988210	1989199	+	atgccgaaccacggagatttttt	MPNHGDFLLNTQQLSAMTPA	fig|127906.87.peg.1802	VCC1802	VC1079		Threonylcarbamoyl-AMP synthase (EC 2.7.7.87) / SUA5 domain with internal deletion		
1	CDS	1989244	1989384	+	gtgagctcacgcagcaaactag	MSSRSKLDNQTEMSADISLVL	fig|127906.87.peg.1803	VCC1803	VC1078		hypothetical protein		
1	CDS	1989463	1989951	+	atgtctgatgtttctaccaccgtcg	MSDVSTTVDFATQTVQPVVES	fig|127906.87.peg.1804	VCC1804	VC1077		hypothetical protein		
1	CDS	1990788	1990000	-	atgagcaaagtgatcgtgcaga	MSKVIVQTGQFKGLRKQPLHN	fig|127906.87.peg.1805	VCC1805	VC1076		Transcriptional regulator, AraC family		
1	CDS	1991343	1990801	-	atgaaagcacgtcatcttttacct	MKARHLLPVLAAAITFPALSSP	fig|127906.87.peg.1806	VCC1806	VC1075		Phospholipid-binding protein		
1	CDS	1991627	1992457	+	atgttactttctgcatccgctttgct	MLLSASALLWLPLALAIMAGFA	fig|127906.87.peg.1807	VCC1807	VC1074		CAAX amino terminal protease family protein		
1	CDS	1995726	1992547	-	atgcctgcagtgcaggcggcaa	MPAVQAAINSDASVVGTESQ	fig|127906.87.peg.1808	VCC1808	VC1073		Chitodextrinase (EC 3.2.1.14)		
1	CDS	1996258	1996115	-	gtgtggggttattctgactcttggg	MWGYSDSWVNDLAAIFIKTC	fig|127906.87.peg.1809	VCC1809	Not annotated as CDS		hypothetical protein		
1	CDS	1997606	1996377	-	atgttgtcccaactgaatgcccat	MLSQLNAHVRQYMLVTFNYW	fig|127906.87.peg.1810	VCC1810	VC1071		MFS-type efflux pump ArsJ specific for 1-arseno-3-phosphoglycerate		
1	CDS	1998148	1997651	-	atgacgcatccaacttgggaatt	MTHPTWELPVADNQASLVLTF	fig|127906.87.peg.1811	VCC1811	VC1070		Protein tyrosine phosphatase (EC 3.1.3.48)		
1	CDS	1999163	1998162	-	atggcaattaaagtcggaattaa	MAIKVGINGFGRIGRLALRASF	fig|127906.87.peg.1812	VCC1812	VC1069	gap	NAD-dependent glyceraldehyde-3-phosphate dehydrogenase (EC 1.2.1.12) for arsenate detoxification		
1	CDS	1999601	1999260	-	atgttaccccaccagtttttcaaat	MLPHQFFKLLADETRVRCLLM	fig|127906.87.peg.1813	VCC1813	VC1068	smtB	Arsenical resistance operon repressor		
1	CDS	2002164	2000143	-	atgctacttaacgctttttcacgcc	MLLNAFSRRVFLWLGWLLIST	fig|127906.87.peg.1814	VCC1814	VC1067	yeaP	GGDEF domain protein		
1	CDS	2003054	2002311	-	atgatactgtctgccttacaatttc	MILSALQFLIAILFSIVAAYVAGL	fig|127906.87.peg.1815	VCC1815	VC1066		hypothetical protein		
1	CDS	2003105	2003407	+	atggaccagtttttacaacagatc	MDQFLQQIFVVIHQIPVGRVST	fig|127906.87.peg.1816	VCC1816	VC1065		DNA base-flipping protein		
1	CDS	2003892	2003461	-	atgaaaaaggttctacttattgtta	MKKVLLIVISILFGSVLVGCQM	fig|127906.87.peg.1817	VCC1817	VC1064		Lipoprotein-related protein		
1	CDS	2004043	2004903	+	atgagtaaaccgttagacgaatt	MSKPLDELLSLLQLEKLEEGL	fig|127906.87.peg.1818	VCC1818	VC1063	tesB	Acyl-CoA thioesterase II (EC 3.1.2.-)		
1	CDS	2005370	2004912	-	atgttgctcgacaaagttgatcgc	MLLDKVDRQILAILQQDCTVSL	fig|127906.87.peg.1819	VCC1819	VC1062		DNA-binding transcriptional activator DecR, AsnC family		
1	CDS	2005492	2006559	+	atgtgtaccgaccatcagtggat	MCTDHQWINSAIRKIEADYQR	fig|127906.87.peg.1820	VCC1820	VC1061	sbnA	Pyridoxal-5'-phosphate-dependent enzyme beta superfamily (fold type II)		
1	CDS	2007745	2006684	-	ttggagtttttgttagattacggcct	MEFLLDYGLFLAKIVTVVVALV	fig|127906.87.peg.1821	VCC1821	VC1060	sohB	Possible protease sohB (EC 3.4.21.-)		
1	CDS	2007904	2008647	+	gtgaattattcagtctcaacagac	MNYSVSTDALKEKVILVTGAG	fig|127906.87.peg.1822	VCC1822	VC1059	yciK	Dehydrogenases with different specificities (related to short-chain alcohol dehydrogenases)		
1	CDS	2009178	2008741	-	atgagtacacccttctggcaaac	MSTPFWQSKTLDQMTEAEWE	fig|127906.87.peg.1823	VCC1823	VC1058		UPF0260 protein YcgN	YcgN family cysteine cluster protein [Vibrio cholerae]	
1	CDS	2009316	2009564	+	atggattgtcgattaggctgcggc	MDCRLGCGACCIAPSISSPIP	fig|127906.87.peg.1824	VCC1824	VC1057		UPF0153 protein YeiW	YkgJ family cysteine cluster protein [Vibrio]	Limited query cover for certain strains
1	CDS	2010276	2009674	-	atgcgtaccagtcacatgctgga	MRTSHMLEHLMEALRCLPGV	fig|127906.87.peg.1825	VCC1825	VC1056	recR	Recombination protein RecR		
1	CDS	2010620	2010291	-	atgtttggtaaaggcggaatggc	MFGKGGMGNLMKQAQQMQE	fig|127906.87.peg.1826	VCC1826	VC1055	ybaB	Nucleoid-associated protein YaaK		
1	CDS	2012769	2010691	-	atgagttatcttgctttagcacgca	MSYLALARKWRPTRFSEVVG	fig|127906.87.peg.1827	VCC1827	VC1054	dnaZX	DNA polymerase III subunits gamma and tau (EC 2.7.7.7)		
1	CDS	2013333	2012788	-	atgacaactgaaactctttcactc	MTTETLSLIKSSIKSIPDYPKKG	fig|127906.87.peg.1828	VCC1828	VC1053	apt	Adenine phosphoribosyltransferase (EC 2.4.2.7)		
1	CDS	2013863	2013465	-	atgcgttcatccttacccacgttttttt	MRSSLPRFLFSVIGITSLCLGIL	fig|127906.87.peg.1829	VCC1829	VC1052	ybaN	Inner membrane protein YbaN	DUF454 domain-containing protein [Vibrio cholerae]	Limited query cover for certain strains
1	CDS	2013994	2013863	-	atggcaaaccaagagttcaaa	MANQEFKEPFNIYYFLGVVAV	fig|127906.87.peg.1830	VCC1830	VC1051		nr0040.cl00013		
1	CDS	2015255	2014164	-	atgtataaatctcacatcccgga	MYKSHIPEDCEEEAQITAAKLI	fig|127906.87.peg.1831	VCC1831	VC1050		Two-component transcriptional response regulator, LuxR family		
1	CDS	2015503	2016378	+	atgaaactagatgacctaaacc	MKLDDLNLFRLVVENGSYTST	fig|127906.87.peg.1832	VCC1832	VC1049		Transcriptional regulator, LysR family		

1	CDS	2017005	2016457	-	atggaagcgctcgatttattgctc	MEALDLLLNRRSIAKLDAPAP	fig|127906.87.peg.1833	VCC1833	VC1048	ydjA	Putative NAD(P)H nitroreductase YdjA		
1	CDS	2019257	2017131	-	atggataacaacaatgcatttca	MDNNNAFQLSFDEQHYAWLA	fig|127906.87.peg.1834	VCC1834	VC1047	fadJ	Enoyl-CoA hydratase (EC 4.2.1.17) / Delta(3)-cis-delta(2)-trans-enoyl-CoA isomerase (EC 5.3.3.8) / 3-hydroxyacyl-CoA dehydrogenase (EC 1.1.1.35) / 3-hydroxybutyryl-CoA epimerase (EC 5.1.2.3)		
1	CDS	2020564	2019257	-	atgggcaaacaggaagtcagg	MGKQEVRTRSGERVAIVAGLI	fig|127906.87.peg.1835	VCC1835	VC1046	fadL	3-ketoacyl-CoA thiolase (EC 2.3.1.16)		
1	CDS	2021095	2021658	+	gtgtcgatattgaatctgtttgga	MSILNLFGKKVSQRPVNNDMI	fig|127906.87.peg.1836	VCC1836	VC1045	sigV	RNA polymerase ECF-type sigma factor		
1	CDS	2021651	2022382	+	atggatgaactggaatttcgccg	MDELEFRRKVMSDPKQRDND	fig|127906.87.peg.1837	VCC1837	VC1044		hypothetical protein	DUF3379 domain-containing protein [Vibrio cholerae]	
1	CDS	2022642	2022794	+	atgaacaacatgcgtctgtttaa	MNNMRLFKKSLLAVTVTLATQ	fig|127906.87.peg.1838	VCC1838	VC1043		Long-chain fatty acid transport protein		Split into 2 ORFs in C6706
1	CDS	2022794	2023918	+	atgtctcgcaacccagcggcga	MSRNPAAMALFDEMALSLGF	fig|127906.87.peg.1839	VCC1839	VC1043		Long-chain fatty acid transport protein		Split into 2 ORFs in C6706
1	CDS	2024221	2025519	+	atgaaaaccaataagactctcc	MKTNKTLLSCAVAFGLASVST	fig|127906.87.peg.1840	VCC1840	VC1042		Long-chain fatty acid transport protein		
1	CDS	2026013	2025546	-	atgcagaaggtactcgtggtgtg	MQKVLVVCMGNICRSPTAEA	fig|127906.87.peg.1841	VCC1841	VC1041	yfkJ	Low molecular weight protein tyrosine phosphatase (EC 3.1.3.48)		
1	CDS	2026099	2026704	+	atgtccggcgatgaaatcaaac	MSGDEIKQERHKARQQKVKE	fig|127906.87.peg.1842	VCC1842	VC1040	btuR/cobA	Cob(I)alamin adenosyltransferase (EC 2.5.1.17)		
1	CDS	2028887	2026776	-	atgaaaaaactgtttatttctcttct	MKKLFISLLGLVVLIVGALLALV	fig|127906.87.peg.1843	VCC1843	VC1039	asmA	AsmA protein		
1	CDS	2029626	2028985	-	atgtctgagaataatcaatgtgtc	MSENNQCVIVGIAGASASGKS	fig|127906.87.peg.1844	VCC1844	VC1038	udk	Uridine kinase (EC 2.7.1.48)		
1	CDS	2030900	2029803	-	atgggcaaaaaaggggaagtt	MGKKGEVMQSIQSKQDLCRW	fig|127906.87.peg.1845	VCC1845	VC1037	mrp	[4Fe-4S] cluster assembly scaffold protein Mrp (=ApbC)		
1	CDS	2031037	2033088	+	atggcaaacgatccaagaaaa	MANDPRKLLVTCALPYANGSI	fig|127906.87.peg.1846	VCC1846	VC1036	metG	Methionyl-tRNA synthetase (EC 6.1.1.10)		
1	CDS	2033392	2034207	+	atgtttgtgacaatttctctggttct	MFVTISLVLSLAILGLIYTLSQR	fig|127906.87.peg.1847	VCC1847	VC1035		hypothetical protein		
1	CDS	2035041	2034280	-	atgaccaaaaccgtattccacct	MTKTVFHLGVTEADLNGATLA	fig|127906.87.peg.1848	VCC1848	VC1034		Uridine phosphorylase (EC 2.4.2.3)		
1	repeat	2035243	2035440	+	tatccccttcctacttgaagctgcagcggtgttggctacgttcgctcaccccaat		fig|127906.87.repeat.36				repeat region		
1	CDS	2037808	2035502	-	atgtgtactaaacaccatgcttgt	MCTKHHACRSKSLTQPMHSH	fig|127906.87.peg.1849	VCC1849	VC1033	zntA	Lead, cadmium, zinc and mercury transporting ATPase (EC 3.6.3.3) (EC 3.6.3.5); Copper-translocating P-type ATPase (EC 3.6.3.4)		
1	CDS	2038337	2037921	-	atggactcactgaaagtcagcg	MDSLKVSDYMTLQAVTFSPDI	fig|127906.87.peg.1850	VCC1850	VC1031		CBS domain protein		
1	CDS	2038566	2039540	+	gtgacttctatttttcctcgacgat	MTSIFSSTIIANPEILQFIFQSLP	fig|127906.87.peg.1851	VCC1851	VC1029		GGDEF domain protein		
1	CDS	2040007	2039555	-	atggaccatcgcgtatcggtgca	MDHRVSVQKEDFSVAQEYEA	fig|127906.87.peg.1852	VCC1852	VC1028	moaE	Molybdopterin synthase catalytic subunit MoaE (EC 2.8.1.12)		
1	CDS	2040254	2040009	-	atgattaaggtgctgtttttgccca	MIKVLFFAQTRELVECDSLVVI	fig|127906.87.peg.1853	VCC1853	VC1027	moaD	Molybdopterin synthase sulfur carrier subunit		
1	CDS	2040730	2040251	-	atgagccaactcactcatattaa	MSQLTHINASGEANMVDVSNI	fig|127906.87.peg.1854	VCC1854	VC1026	moaC	Cyclic pyranopterin monophosphate synthase (EC 4.6.1.17)		
1	CDS	2041253	2040741	-	atgggtcacgcagaaagcaaa	MGHAESKFQAANIAVLTVSDT	fig|127906.87.peg.1855	VCC1855	VC1025	moaB	Molybdenum cofactor biosynthesis protein MoaB		
1	CDS	2042302	2041313	-	gtggcgcaacaattcgaagaca	MAQQFEDRFQRKFYYLRLSIT	fig|127906.87.peg.1856	VCC1856	VC1024	moaA	GTP 3',8-cyclase (EC 4.1.99.22)		Limited query cover for certain strains
1	CDS	2042608	2043498	+	atgtctctatacgaaaacaaaaa	MSLYENKKVVAIGGGHGLGRI	fig|127906.87.peg.1857	VCC1857	VC1023		FIG002813: LPPG:FO 2-phospho-L-lactate transferase like, CofD-like		
1	CDS	2043828	2043487	-	atgagagaatggatcaaccaaa	MREWINQSKIDLLAKEIGEENV	fig|127906.87.peg.1858	VCC1858	VC1022	luxU	Phosphorelay protein LuxU		
1	CDS	2045241	2043832	-	atgcaacataatcaatctttgcag	MQHNQSLQKTKYVLMVEDTA	fig|127906.87.peg.1859	VCC1859	VC1021	luxO	Regulatory protein LuxO		
1	CDS	2047660	2045630	-	atgagcaaagcatttgaattggtt	MSKAFELVSDYQPAGDQPTA	fig|127906.87.peg.1860	VCC1860	VC1018	uvrB	Excinuclease ABC subunit B		uvrB is split into 2 ORFs in N16961 where only one has refseq annotation in PATRIC. Annotated as 1 ORF named VC1018 in KEGG
									No refSeq annotation				uvrB is split into 2 ORFs in N16961 where only one has refseq annotation in PATRIC. Annotated as 1 ORF named VC1018 in KEGG
1	rna	2048247	2048322	+	tcctccttagttcagtcggtagaacggcggactgttaatccgtatgtcgcaggtt		fig|127906.87.rna.53	VCCt040	VCt055		tRNA-Asn-GTT		
1	CDS	2048509	2049123	+	atgccgggatcggaagcaaac	MPGSEANIYITMTEYLLLLIGTV	fig|127906.87.peg.1861	VCC1861	VC1017	rnfA	Electron transport complex protein RnfA		
1	CDS	2049123	2049710	+	atgagtaccatagtcatcgctgtt	MSTIVIAVIALAALAAVFGAILGI	fig|127906.87.peg.1862	VCC1862	VC1016	rnfB	Electron transport complex protein RnfB		
1	CDS	2049719	2052043	+	atgttgtcattaatcgaacaaatc	MLSLIEQIKSGKLWDFPGGIHP	fig|127906.87.peg.1863	VCC1863	VC1015	rnfC	Electron transport complex protein RnfC		
1	CDS	2052043	2053089	+	gtggccttctttattgctagctccc	MAFFIASSPHLRSKRSTADVM	fig|127906.87.peg.1864	VCC1864	VC1014	rnfD	Electron transport complex protein RnfD		
1	CDS	2053101	2053730	+	atgctgacagcaattcgaaaaa	MLTAIRKNGLILAVFACVSTGL	fig|127906.87.peg.1865	VCC1865	VC1013	rnfG	Electron transport complex protein RnfG		
1	CDS	2053732	2054424	+	atgaatgaaaaccgaactctga	MNENRTLMLNGMWNNNPALV	fig|127906.87.peg.1866	VCC1866	VC1012	rnfE	Electron transport complex protein RnfE		
1	CDS	2054451	2055092	+	atgaataacgcgaaacggattg	MNNAKRIEILERLRAHNPKPET	fig|127906.87.peg.1867	VCC1867	VC1011	nth	Endonuclease III (EC 4.2.99.18)		
1	CDS	2055133	2055549	+	atgtccaaccatcgtattttgcac	MSNHRILHTMLRVGDLDKSIEI	fig|127906.87.peg.1868	VCC1868	VC1010	gloA	Lactoylglutathione lyase (EC 4.4.1.5)		
1	CDS	2055711	2056148	+	atgaatataaaagagtgggaaa	MNIKEWERHTLLADIAMQESD	fig|127906.87.peg.1869	VCC1869	VC1009		hypothetical protein	DUF2753 domain-containing protein [Vibrio cholerae]	
1	CDS	2057081	2056197	-	atgaataaatgggttatcacaag	MNKWVITSLFAISTLSASVASA	fig|127906.87.peg.1870	VCC1870	VC1008	motY	Sodium-type flagellar protein MotY		
1	CDS	2057154	2057285	+	atgcaaaaatctagccagcacg	MQKSSQHDTGMTVPKQLACR	fig|127906.87.peg.1871	VCC1871	VC1007		hypothetical protein		
1	CDS	2057355	2058029	+	atgactgataaaaatgacgcgc	MTDKNDALTLKKRFRGYFPVV	fig|127906.87.peg.1872	VCC1872	VC1006	rnt	Ribonuclease T		
1	CDS	2058130	2059455	+	atgaatccagtagtcatttccgttt	MNPVVISVCVMLLLALMRVNV	fig|127906.87.peg.1873	VCC1873	VC1005	yuiF	Histidine permease YuiF		
1	CDS	2061097	2059583	-	atgtgtggtattgttggaatcgtgc	MCGIVGIVGTTPVNQSIYDALT	fig|127906.87.peg.1874	VCC1874	VC1004	purF	Amidophosphoribosyltransferase (EC 2.4.2.14)		
1	CDS	2061621	2061130	-	atgaactggttagatattgtcattt	MNWLDIVILSVIGLSALISLVRG	fig|127906.87.peg.1875	VCC1875	VC1003	cvpA	Colicin V production protein		
1	CDS	2062280	2061684	-	atggccagtaaatttcaaagccg	MASKFQSRLVGTIVLVAIGVIVI	fig|127906.87.peg.1876	VCC1876	VC1002	dedD	Septum-associated cell division protein DedD		
1	CDS	2063546	2062281	-	atgactcaaaagcctgttcctca	MTQKPVPQATSPLAVWLDYLA	fig|127906.87.peg.1877	VCC1877	VC1001	folC	Dihydrofolate synthase (EC 6.3.2.12) @ Folylpolyglutamate synthase (EC 6.3.2.17)		
1	CDS	2064517	2063591	-	atgagttggctcgaaaagatttta	MSWLEKILEKSNIVSSRKASIP	fig|127906.87.peg.1878	VCC1878	VC1000	accD	Acetyl-coenzyme A carboxyl transferase beta chain (EC 6.4.1.2)		
1	CDS	2065485	2064691	-	atgagaatcgcgttaggtattgag	MRIALGIEYDGTHYYGWQRQR	fig|127906.87.peg.1879	VCC1879	VC0999	truA	tRNA pseudouridine(38-40) synthase (EC 5.4.99.12)		
1	CDS	2070145	2065607	-	ttggccatttatcagctcaatcctc	MAIYQLNPQAFENQNIHTLIPG	fig|127906.87.peg.1880	VCC1880	VC0998		Probable type IV pilus assembly FimV-related transmembrane protein		
1	CDS	2070138	2070356	+	atggccaataacgtttgttgcacc	MANNVCCTVTLEEGRNWLAID	fig|127906.87.peg.1881	VCC1881	No refSeq annotation		FIG01201069: hypothetical protein		
1	CDS	2072307	2070637	-	atgagtgaagctgaggctcgtcc	MSEAEARPSNFIRQIIDKDLAD	fig|127906.87.peg.1882	VCC1882	VC0997	glnS	Glutaminyl-tRNA synthetase (EC 6.1.1.18)		
1	CDS	2074126	2072555	-	gtgaatattcttggatatgcgcaa	MNILGYAQKLGKALMLPIATLP	fig|127906.87.peg.1883	VCC1883	VC0995	nagE	PTS system, N-acetylglucosamine-specific IIC component / PTS system, N-acetylglucosamine-specific IIB component (EC 2.7.1.193)		
1	CDS	2074595	2075731	+	atgtatgcgctaacgaactgcaa	MYALTNCKIYTGNDVLVKHAV	fig|127906.87.peg.1884	VCC1884	VC0994	nagA	N-acetylglucosamine-6-phosphate deacetylase (EC 3.5.1.25)		
1	CDS	2075734	2076948	+	atgaatggcggacagattggta	MNGGQIGNVDLVKQLNSAAV	fig|127906.87.peg.1885	VCC1885	VC0993	nagC	N-acetylglucosamine-6P-responsive transcriptional repressor NagC, ROK family		
1	CDS	2077043	2078629	+	atggatttgatacttctttctgccg	MDLILLSAAFLAGFIALKCHLPI	fig|127906.87.peg.1886	VCC1886	VC0992	ybaL	Putative glutathione-regulated potassium-efflux system protein KefB		
1	CDS	2078825	2080489	+	atgtgctctatctttggcatcttaga	MCSIFGILDIKTDAATLRPIALE	fig|127906.87.peg.1887	VCC1887	VC0991	asnB	Asparagine synthetase [glutamine-hydrolyzing] (EC 6.3.5.4)		
1	CDS	2081134	2080637	-	atgaaacgttggtatctactttact	MKRWYLLYCKRGEQQRAKMI	fig|127906.87.peg.1888	VCC1888	VC0990	rfaH	Transcriptional activator RfaH		
1	CDS	2081355	2081489	+	atggagcattcacgcccaagttt	MEHSRPSLVASPFYEKLVCAT	fig|127906.87.peg.1889	VCC1889	No refSeq annotation		FIG01203873: hypothetical protein		
1	CDS	2081642	2083123	+	atgtccaaccttaatatctttaaac	MSNLNIFKQPNAFYLIFSIEFW	fig|127906.87.peg.1890	VCC1890	VC0988	tppB	Di-tripeptide/H+ symporter DtpA		
1	CDS	2084185	2083223	-	atgaataatcataaaaaacttgg	MNNHKKLGILLANLGTPQAPT	fig|127906.87.peg.1891	VCC1891	VC0987	hemH	Ferrochelatase, protoheme ferro-lyase (EC 4.99.1.1)		
1	CDS	2084956	2084312	-	atgcgcatcattcttctcggtgctc	MRIILLGAPGAGKGTQAQFIMI	fig|127906.87.peg.1892	VCC1892	VC0986	adk	Adenylate kinase (EC 2.7.4.3)		
1	CDS	2087237	2085291	-	atggatcaattgtttgaaacagaa	MDQLFETEVNGAIMSETATTN	fig|127906.87.peg.1893	VCC1893	VC0985	htpG	Chaperone protein HtpG		
1	CDS	2087462	2088310	+	atgagtcatattggtactaaattca	MSHIGTKFILAEKFTFDPLSNT	fig|127906.87.peg.1894	VCC1894	VC0984	toxR	Transcriptional activator ToxR		
1	CDS	2088320	2088841	+	atgcaaaatagacacatcgcca	MQNRHIAMGILLLSLLLSSWLY	fig|127906.87.peg.1895	VCC1895	VC0983	toxS	Transmembrane regulatory protein ToxS		
1	CDS	2088882	2089175	+	atgaacaaagcccaaatcgag	MNKAQIEIYYCRQCNWMLRSA	fig|127906.87.peg.1896	VCC1896	VC0982		Selenoprotein W-related protein		
1	CDS	2089804	2089256	-	atgaaactattttttgtctcagatct	MKLFFVSDLHGSLSATEQALA	fig|127906.87.peg.1897	VCC1897	VC0981	yfcE	Phosphodiesterase YfcE		
1	CDS	2090219	2091133	+	atgagaattttaattactggagga	MRILITGGTGFVGFELIKLLSSH	fig|127906.87.peg.1898	VCC1898	No refSeq annotation	rcp	Cell division inhibitor Slr1223 (YfcH in EC), contains epimerase/dehydratase and DUF1731 domains		
1	CDS	2091176	2092003	+	atgaccaaatcgattttcattactg	MTKSIFITGCSSGIGYMAAKQL	fig|127906.87.peg.1899	VCC1899	VC0979	bdhA	Putative NAD(P)-dependent oxidoreductase EC-YbbO		
1	CDS	2092078	2092194	+	ttgagtaaaaacatcaatatctat	MSKNINIYQALHVVRQKMTLN	fig|127906.87.peg.1900	VCC1900	VC0978		FIG01200650: hypothetical protein		
1	CDS	2092432	2093286	+	atgcaagcagcgaagattattg	MQAAKIIDVSQHNFHQVLQHS	fig|127906.87.peg.1901	VCC1901	VC0977		FIG000875: Thioredoxin domain-containing protein EC-YbbN		
1	CDS	2093717	2094637	+	atggctattgattcattgatcacta	MAIDSLITIAVLVLAVVIFISSAV	fig|127906.87.peg.1902	VCC1902	VC0976	hflK	Protein QmcA (possibly involved in integral membrane quality control)		
1	CDS	2094645	2095094	+	gtggtagagctactctcacaattg	MVELLSQLNFWHWLALGLLLL	fig|127906.87.peg.1903	VCC1903	VC0975	ybbJ	Putative activity regulator of membrane protease YbbK		
1	CDS	2095557	2095138	-	atgaatattagccagattgccaa	MNISQIAKLTSLTAKSIRLYEEK	fig|127906.87.peg.1904	VCC1904	VC0974	cueR	Copper resistance transcriptional regulator CueR (MerR family)		
1	CDS	2096430	2095618	-	atgaatttatttaaaacgaccata	MNLFKTTILFSTITMAVGCSNL	fig|127906.87.peg.1905	VCC1905	VC0973		VvgS protein		
1	CDS	2097559	2096510	-	atggacaaaatgtttaaacgtac	MDKMFKRTLLGAAVAMASTGA	fig|127906.87.peg.1906	VCC1906	VC0972	chiP	Chitin outer membrane porin		
1	CDS	2100199	2098190	-	atgtcagatgtcgcgcaacgatt	MSDVAQRLTELRKTLHEHGVI	fig|127906.87.peg.1907	VCC1907	VC0971	ligA	DNA ligase (NAD(+)) (EC 6.5.1.2)		
1	CDS	2101207	2100332	-	atgcaggaactgcgattcgtattg	MQELRFVLIIVGALAIAALLFHG	fig|127906.87.peg.1908	VCC1908	VC0970	zipA	Cell division protein ZipA		
1	CDS	2101437	2102189	+	atgcagatcagctcacgttccgg	MQISSRSGFGYFSYGIRLALTI	fig|127906.87.peg.1909	VCC1909	VC0969	cysZ	Sulfate transporter, CysZ-type		
1	CDS	2102312	2103280	+	atgagcaaaatctacgaagaca	MSKIYEDNSLTIGNTPLVRLNK	fig|127906.87.peg.1910	VCC1910	VC0968	cysK	Cysteine synthase (EC 2.5.1.47)		
1	CDS	2103578	2103835	+	atgtacgagaagcaagtagaa	MYEKQVEITAENGLHTRPAAQ	fig|127906.87.peg.1911	VCC1911	VC0966	ptsH	Phosphotransferase system, phosphocarrier protein HPr		
1	CDS	2103969	2105690	+	atgatttcaggcattctagcatctc	MISGILASPGIAIGKALLLQEDE	fig|127906.87.peg.1912	VCC1912	VC0965	ptsI	Phosphoenolpyruvate-protein phosphotransferase of PTS system (EC 2.7.3.9)		
1	CDS	2105799	2106308	+	atgggtctgtttgacaaacttaag	MGLFDKLKKLVSDDSANAGAI	fig|127906.87.peg.1913	VCC1913	VC0964	crr	PTS system, glucose-specific IIA component (EC 2.7.1.199)		
1	CDS	2107584	2106421	-	atgagtggcaacaaatttgacat	MSGNKFDILVVGGGMVGAAA	fig|127906.87.peg.1914	VCC1914	VC0963		2-polyprenyl-3-methyl-6-methoxy-1,4-benzoquinol hydroxylase		
1	CDS	2107838	2109262	+	atgagtaagaaactgctaattaa	MSKKLLIKTWGCQMNEYDSSI	fig|127906.87.peg.1915	VCC1915	VC0962		tRNA-i(6)A37 methylthiotransferase (EC 2.8.4.3)		
1	CDS	2109330	2110433	+	ttgagcaataaaatcataacctta	MSNKIITLEINLEPSDNHRLASL	fig|127906.87.peg.1916	VCC1916	VC0961	ybeZ	Phosphate starvation-inducible protein PhoH, predicted ATPase		
1	CDS	2110430	2110894	+	atgagtattgaactggatttgcaa	MSIELDLQLAVENEHGLPSEAI	fig|127906.87.peg.1917	VCC1917	VC0960	ybeY	Metal-dependent hydrolase YbeY, involved in rRNA and/or ribosome maturation and assembly		
1	CDS	2110964	2111839	+	atgaacgaagacaattctcaga	MNEDNSQNSEGPSRKSFFER	fig|127906.87.peg.1918	VCC1918	VC0959		Magnesium and cobalt efflux protein CorC		
1	CDS	2111880	2113397	+	atgaacagcgtattatctcatcgc	MNSVLSHRLMRPLAAAFVGVI	fig|127906.87.peg.1919	VCC1919	VC0958	int	Apolipoprotein N-acyltransferase / Copper homeostasis protein CutE		
1	CDS	2113923	2113453	-	atgccaaaacagaaagcaagt	MPKQKASYEALLEEVVETLKH	fig|127906.87.peg.1920	VCC1920	VC0957		FIG002095: hypothetical protein	zinc ribbon-containing protein [Vibrio cholerae]	
1	CDS	2114053	2116629	+	atgcaagagcaatacaacccg	MQEQYNPQDIEHKVQQHWDN	fig|127906.87.peg.1921	VCC1921	VC0956	leuS	Leucyl-tRNA synthetase (EC 6.1.1.4)		
1	CDS	2116806	2117420	+	atgcgtaacactcttttccctactt	MRNTLFPTLKWSLLVLMTSLL	fig|127906.87.peg.1922	VCC1922	VC0954	lptE/rlpB	LPS-assembly lipoprotein LptE		
1	CDS	2117426	2118454	+	atgcgcatttacgctgaaaaact	MRIYAEKLAESLHKTLYPIYLVI	fig|127906.87.peg.1923	VCC1923	VC0953	holA	DNA polymerase III delta subunit (EC 2.7.7.7)		
1	CDS	2118523	2118840	+	ttgcaaggtgaagcacttaaaga	MQGEALKDFLFDKADDMKAV	fig|127906.87.peg.1924	VCC1924	VC0952	rsfS	Ribosomal silencing factor RsfA		
1	CDS	2118843	2119313	+	gtgaagattcaactgatcgccgt	MKIQLIAVGTKMPKWVEEGFQ	fig|127906.87.peg.1925	VCC1925	VC0951	rlmH	23S rRNA (pseudouridine(1915)-N(3))-methyltransferase (EC 2.1.1.177)		
1	CDS	2119320	2121236	+	atgttacgccagcataccccta	MLRQHTPIRDHQAETHLFRNR	fig|127906.87.peg.1926	VCC1926	VC0950	spoVD/mrdA	Peptidoglycan D,D-transpeptidase MrdA (EC 3.4.16.4)		
1	CDS	2121233	2122354	+	atgaagatggatcctgcaaccg	MKMDPATGQNRALFERFHIDL	fig|127906.87.peg.1927	VCC1927	VC0949	mrdB/rodA	Rod shape-determining protein RodA		
1	CDS	2122354	2123145	+	atgaaccgaatttatttatacttac	MNRIYLYLLIVLILAGCSSPGGI	fig|127906.87.peg.1928	VCC1928	VC0948	rlpA	Septum-associated rare lipoprotein A		
1	CDS	2123237	2124412	+	atgaaaaaatcaatcctcaaatc	MKKSILKSVLASSITLSITLSSTA	fig|127906.87.peg.1929	VCC1929	VC0947		D-alanyl-D-alanine carboxypeptidase (EC 3.4.16.4)		
1	CDS	2124560	2124838	+	atgctgaacatcaactctgatgc	MLNINSDAKLKDLLEFPCSFTY	fig|127906.87.peg.1930	VCC1930	VC0945		Proposed lipoate regulatory protein YbeD		
1	CDS	2125026	2125676	+	atggaaaaccagctgttggtgag	MENQLLVRRLGRQDYTPVWQ	fig|127906.87.peg.1931	VCC1931	VC0944	lipB	Octanoate-[acyl-carrier-protein]-protein-N-octanoyltransferase (EC 2.3.1.181)		
1	CDS	2125669	2126634	+	atgagtaaaccaattcagatgga	MSKPIQMERGVKYRDADKMA	fig|127906.87.peg.1932	VCC1932	VC0943	lipA	Lipoyl synthase (EC 2.8.1.8)		
1	CDS	2126830	2127156	+	atgaatcatatcggcttatttgccc	MNHIGLFALTAAALLAGCSSN	fig|127906.87.peg.1933	VCC1933	VC0942		hypothetical protein	YitT family protein [Vibrio cholerae]	
1	CDS	2128570	2127320	-	atgcttaagcgtgatatgaacat	MLKRDMNIADYDPELYAAIQE	fig|127906.87.peg.1934	VCC1934	VC0941	glyA	Serine hydroxymethyltransferase (EC 2.1.2.1)		
1	CDS	2128911	2129507	+	atggaaaaacattcacataaag	MEKHSHKEDWIAILTGTFLVA	fig|127906.87.peg.1935	VCC1935	VC0940		Uncharacterized UPF0750 membrane protein		
1	repeat	2129520	2129640	+	gttataccaaaactacttggagttgcaggtaggcggcaagagagtgaatccc		fig|127906.87.repeat.37				repeat region		
1	CDS	2130092	2129658	-	ttgcaacagcgtcttaaaatagg	MQQRLKIGRQIYQQRPVSLVL	fig|127906.87.peg.1936	VCC1936	VC0939		Hypothetical protein VpsQ	VanZ family protein [Vibrio cholerae]	
1	CDS	2130778	2130071	-	gtgaacgcttttttcagtttggatg	MNAFFQFGWRVGVASSALWV	fig|127906.87.peg.1937	VCC1937	VC0938		Hypothetical protein VpsP		
1	CDS	2132988	2130775	-	atgacaggcaaacaattcactc	MTGKQFTQEEVIDIRQYFNVLI	fig|127906.87.peg.1938	VCC1938	VC0937	cpsD	Capsular polysaccharide synthesis enzyme CpsD, exopolysaccharide synthesis		
1	CDS	2133540	2133013	-	atgatgaagtacctactctcttttt	MMKYLLSFLLCLLVWPSLAVA	fig|127906.87.peg.1939	VCC1939	VC0936	cpsC	Capsular polysaccharide synthesis enzyme CpsC, polysaccharide export		
1	CDS	2134740	2133544	-	atggaaccgattaaactgacttc	MEPIKLTSLFSASLVTLGVTYA	fig|127906.87.peg.1940	VCC1940	VC0935	cpsB	Capsular polysaccharide synthesis enzyme CpsB		
1	CDS	2136205	2134811	-	atgaaggaaaaaagcagaata	MKEKSRIRITNYHGKFFYRLID	fig|127906.87.peg.1941	VCC1941	VC0934	cpsA	Capsular polysaccharide synthesis enzyme CpsA, sugar transferase		
1	CDS	2136698	2137303	+	atgaaacccgtaccattaccact	MKPVPLPLVLLIAFFPSMNAFA	fig|127906.87.peg.1942	VCC1942	VC0933		hypothetical protein		ORF is split into 2 ORFs in N16961, both has refseq annotation in PATRIC. Annotated as 1 pseudogene ORF named VC0932 in KEGG
									VC0932				ORF is split into 2 ORFs in N16961, both has refseq annotation in PATRIC. Annotated as 1 pseudogene ORF named VC0932 in KEGG
1	CDS	2137479	2138795	+	atgctttcgtttcacaaaactctat	MLSFHKTLFFAFLLFVFWLPIP	fig|127906.87.peg.1943	VCC1943	VC0931	rbmD	RbmD, similar to Lipid A core - O-antigen ligase and related enzymes		
1	CDS	2141823	2138950	-	atgacgtctcactatattgcacttg	MTSHYIALAVGLLSLSSNVVQA	fig|127906.87.peg.1944	VCC1944	VC0930	rbmC	Hemolysin-related protein RbmC		
1	CDS	2142087	2143313	+	gtgctgttatacttaaatcaattca	MLLYLNQFNKEGGILRYIFISLV	fig|127906.87.peg.1945	VCC1945	VC0929		hypothetical protein		
1	CDS	2144310	2143519	-	atgaacaaacgtcattattatctg	MNKRHYYLASCLALLFSTASY	fig|127906.87.peg.1946	VCC1946	VC0928	rbmA	RbmA protein		
1	CDS	2145267	2144530	-	ttgaaacgcattaattttctgggta	MKRINFLGSPMDVASMSETVA	fig|127906.87.peg.1947	VCC1947	VC0927	tagA/cpsF	N-acetylmannosaminyltransferase (EC 2.4.1.187)		
1	CDS	2146444	2145272	-	atgaaatcaacgactgtagctga	MKSTTVADISQCLLNKARTEQ	fig|127906.87.peg.1948	VCC1948	VC0926		Hypothetical protein VpsJ	Aspartate-semialdehyde dehydrogenase [Vibrio cholerae]	
1	CDS	2147517	2146441	-	gtgagaaaaaacattaatctaat	MRKNINLMIATDLKGQGGVAT	fig|127906.87.peg.1949	VCC1949	VC0925	mshA	polysaccharide biosynthesis protein, putative		
1	CDS	2148854	2147514	-	atgtttaccagttcactttatgttaa	MFTSSLYVKAPIWLQNVMLSV	fig|127906.87.peg.1950	VCC1950	VC0924	paaK	CapK protein, putative		
1	CDS	2149285	2148854	-	atgtggttgtaccgtgttggccatt	MWLYRVGHFFHKRRIPLLPQV	fig|127906.87.peg.1951	VCC1951	VC0923		Serine acetyltransferase (EC 2.3.1.30)		Limited query cover for certain strains
1	CDS	2150516	2149296	-	ttgagcgaacgtaatctcaatcg	MSERNLNRWMILIILSAFLLGA	fig|127906.87.peg.1952	VCC1952	VC0922		hypothetical protein	VpsF [Vibrio cholerae]	
1	CDS	2151922	2150513	-	ttgattgctagtctactcaaatatg	MIASLLKYAPVQVFSALSVFAL	fig|127906.87.peg.1953	VCC1953	VC0921		polysaccharide export protein, putative		
1	CDS	2153067	2151919	-	atgagtaaaatattgcttatcatg	MSKILLIMPLSTLNWGEKNLG	fig|127906.87.peg.1954	VCC1954	VC0920		hypothetical protein	Glycosyltransferase family 4 protein [Vibrio cholerae]	
1	CDS	2153618	2153064	-	atgagttcaccttgggagctgatt	MSSPWELIKADLYRHTGQVSI	fig|127906.87.peg.1955	VCC1955	VC0919		Serine acetyltransferase (EC 2.3.1.30)		Limited query cover for certain strains
1	CDS	2154856	2153615	-	atgtttaataaagtttcagtgattg	MFNKVSVIGLGYIGLPTAAVLA	fig|127906.87.peg.1956	VCC1956	VC0918	wbpA/wecC	UDP-N-acetyl-D-mannosamine dehydrogenase (EC 1.1.1.336)		
1	CDS	2155994	2154876	-	gtgaaaaaagttctcatcgtattt	MKKVLIVFGTRPEAIKMAPLV	fig|127906.87.peg.1957	VCC1957	VC0917	wecB	UDP-N-acetylglucosamine 2-epimerase (EC 5.1.3.14)		
1	CDS	2156137	2156226	+	atgtatgtcagcttattatttaaatt	MYVSLLFKFVVINNLIYLKIYGL	fig|127906.87.peg.1958	VCC1958	Not annotated as CDS		hypothetical protein		Limited query cover for certain strains
1	CDS	2156807	2156307	-	atgaaggttaaaggtttatcagta	MKVKGLSVLVVCTGNLCRSPI	fig|127906.87.peg.1959	VCC1959	VC0916		Low molecular weight protein tyrosine phosphatase (EC 3.1.3.48)		Limited query cover for certain strains
1	CDS	2160344	2157234	-	atgtggttgtcagatgtatcggta	MWLSDVSVKRPVAALVLSMLL	fig|127906.87.peg.1960	VCC1960	VC0914		RND efflux system, inner membrane transporter		
1	CDS	2161402	2160344	-	atgaacaaaaacactgtactttc	MNKNTVLSLTLLTLLMNSAVLS	fig|127906.87.peg.1961	VCC1961	VC0913		RND efflux system, membrane fusion protein		
1	rna	2161688	2161772	+	ggaggggttcccgagtggccaaagggagcagactgtaaatctgccggctc		fig|127906.87.rna.54	VCCt041	VCt054		tRNA-Tyr-GTA		
1	rna	2161857	2161941	+	ggaggggttcccgagtggccaaagggagcagactgtaaatctgccggctc		fig|127906.87.rna.55	VCCt042	VCt053		tRNA-Tyr-GTA		
1	rna	2162026	2162110	+	ggaggggttcccgagtggccaaagggagcagactgtaaatctgccggctc		fig|127906.87.rna.56	VCCt043	VCt052		tRNA-Tyr-GTA		
1	rna	2162195	2162279	+	ggaggggttcccgagtggccaaagggagcagactgtaaatctgccggctc		fig|127906.87.rna.57	VCCt044	VCt051		tRNA-Tyr-GTA		
1	CDS	2164146	2162458	-	atgagtagctcaacccaatccat	MSSSTQSMPWWKTAAIYQIYI	fig|127906.87.peg.1962	VCC1962	VC0911	treA/treC	Trehalose-6-phosphate hydrolase (EC 3.2.1.93)		
1	CDS	2165650	2164226	-	atgagcaagatagcgaaacaa	MSKIAKQDVTRLIELVGGESNI	fig|127906.87.peg.1963	VCC1963	VC0910	treB	PTS system, trehalose-specific IIB component (EC 2.7.1.201) / PTS system, trehalose-specific IIC component		
1	CDS	2166903	2165899	-	gtggccttattgaccgctcattctt	MALLTAHSFLINEYQYKRMNR	fig|127906.87.peg.1964	VCC1964	VC0909	treR	Trehalose operon transcriptional repressor		
1	CDS	2167474	2166914	-	ttggccaaacccgccgtatttcttc	MAKPAVFLDRDGVINVDHGYV	fig|127906.87.peg.1965	VCC1965	VC0908	gmhB	D-glycero-beta-D-manno-heptose-1,7-bisphosphate 7-phosphatase (EC 3.1.3.82)		
1	CDS	2167710	2168744	+	atgattgagattaaaagcgtaaa	MIEIKSVNKVFYQGDKQIHALK	fig|127906.87.peg.1966	VCC1966	VC0907	metN	Methionine ABC transporter ATP-binding protein		
1	CDS	2168734	2169411	+	atgtcctttaatacgattgcacaa	MSFNTIAQWFALNSDLLLTATV	fig|127906.87.peg.1967	VCC1967	VC0906	metI	Methionine ABC transporter permease protein		
1	CDS	2169448	2170257	+	atgaagtttagcctgaaaagttta	MKFSLKSLLTIAAAASTLILAGC	fig|127906.87.peg.1968	VCC1968	VC0905	metQ	Methionine ABC transporter substrate-binding protein		
1	CDS	2171166	2170390	-	atgtacgtggttgagttggagtttc	MYVVELEFECFDNTTISAVDKA	fig|127906.87.peg.1969	VCC1969	VC0904		Zn-ribbon-containing, possibly nucleic-acid-binding protein		
1	CDS	2171716	2171168	-	atgacgcaaagcgtagtgcaag	MTQSVVQALSAFSQRFLQHCA	fig|127906.87.peg.1970	VCC1970	VC0903	syd	Syd protein		
1	CDS	2171813	2172658	+	atgagcaaatattccgatgccaa	MSKYSDAKELASLTLGKKTEY	fig|127906.87.peg.1971	VCC1971	VC0902	queF	NADPH-dependent 7-cyano-7-deazaguanine reductase (EC 1.7.1.13)		
1	CDS	2172673	2174934	+	atgagatttctgcgctggataacc	MRFLRWITIATLLLASSWAQAT	fig|127906.87.peg.1972	VCC1972	VC0901		hypothetical protein	tetratricopeptide repeat protein [Vibrio cholerae]	
1	CDS	2175045	2176610	+	atgggcgttctagaacaagatat	MGVLEQDIQAELQKLKSQLEQ	fig|127906.87.peg.1973	VCC1973	VC0900		GGDEF domain family protein		
1	CDS	2176830	2178188	+	atgattattcaagtcagcccagc	MIIQVSPAGSMDLLSQLEVERI	fig|127906.87.peg.1974	VCC1974	VC0899	ygdH	Pyrimidine/purine nucleotide 5'-monophosphate nucleosidase PpnN (EC 3.2.2.4) (EC 3.2.2.10)		
1	CDS	2178299	2179078	+	atgtctcttcatcttgtcattattgat	MSLHLVIIDALNLIRRVHSAQPI	fig|127906.87.peg.1975	VCC1975	VC0898	xni	Flap endonuclease Xni		
1	CDS	2179494	2179108	-	atgaacagtaagtatttactcgct	MNSKYLLALGGLLSGVAVGLG	fig|127906.87.peg.1976	VCC1976	VC0897		FIG00018398: hypothetical regulator	DUF423 domain-containing protein [Vibrio cholerae]	
1	CDS	2180436	2179516	-	atgtccagaagattgcctcccctt	MSRRLPPLNSLKVFEAAARHL	fig|127906.87.peg.1977	VCC1977	VC0896		Glycine cleavage system transcriptional activator GcvA		
1	repeat	2180785	2180969	+	cttggaattgcaggtaggcggcaagagagtgacaaatttgtctggaacaaat		fig|127906.87.repeat.38				repeat region		
1	CDS	2182508	2181057	-	atgaaatttatcgttaaaccccat	MKFIVKPHPEIFVKSESVRKRF	fig|127906.87.peg.1978	VCC1978	VC0894	thi	tRNA 4-thiouridine synthase (EC 2.8.1.4) / Rhodanese-like domain required for thiamine synthesis		
1	repeat	2182673	2182805	+	tgtatatacccaaactacttggagttgcaggtaggcggcaagtgagtgagtgc		fig|127906.87.repeat.39				repeat region		
1	CDS	2183768	2182812	-	atggatgacgaacaacaatgta	MDDEQQCKCPPPGLPAWLGT	fig|127906.87.peg.1979	VCC1979	VC0893	motB	Flagellar motor rotation protein MotB		
1	CDS	2184544	2183780	-	gtggatttagcaacactggttggt	MDLATLVGLIGGMAFVIMAMV	fig|127906.87.peg.1980	VCC1980	VC0892	pomA	Flagellar motor rotation protein MotA		
1	CDS	2184859	2185101	+	atggcgagcaagaaaccggaa	MASKKPENMSFESTIEELEQL	fig|127906.87.peg.1981	VCC1981	VC0891	xseB	Exodeoxyribonuclease VII small subunit (EC 3.1.11.6)		
1	CDS	2185143	2186027	+	atgattgaggcgttatcctcatac	MIEALSSYQQRNNQQLDQWL	fig|127906.87.peg.1982	VCC1982	VC0890	ispA	(2E,6E)-farnesyl diphosphate synthase (EC 2.5.1.10) @ Dimethylallyltransferase (EC 2.5.1.1)		
1	CDS	2186047	2187927	+	atgactcttgatatctcaaaatac	MTLDISKYPTLALANTPDELRS	fig|127906.87.peg.1983	VCC1983	VC0889	dxs	1-deoxy-D-xylulose 5-phosphate synthase (EC 2.2.1.7)		
1	CDS	2188759	2188025	-	atgctcgagattgtttatcaagat	MLEIVYQDEYLVAVNKPAGML	fig|127906.87.peg.1984	VCC1984	VC0888	truC	tRNA pseudouridine(65) synthase (EC 5.4.99.26)		

1	CDS	2189071	2188760	-	atgcagttcaacgccgtattggtg	MQFNAVLVKLLDELEVRLRAA	fig|127906.87.peg.1985	VCC1985	VC0887		Uncharacterized protein YqcC	pseudouridine synthase [Vibrio cholerae]	
1	CDS	2189160	2190197	+	atggacactattcgtaccctcacg	MDTIRTLTALLQNSGCQYQIFD	fig|127906.87.peg.1986	VCC1986	VC0886		hypothetical protein	DUF3549 family protein [Vibrio cholerae]	
1	CDS	2190201	2190503	+	atgattggcgatttgctcttgatac	MIGDLLLILALCFAGFLFWQQR	fig|127906.87.peg.1987	VCC1987	VC0885		hypothetical protein	DUF3301 domain-containing protein [Vibrio cholerae]	
1	CDS	2190993	2190460	-	gtgaccatagcgacccgaaga	MTIATRRTLLIPYTEAFESDFL	fig|127906.87.peg.1988	VCC1988	VC0884		Acetyltransferase		
1	CDS	2191848	2192147	+	atgctgaccaaagacgtgtctca	MLTKDVSQELEEILNSLQQQG	fig|127906.87.peg.1989	VCC1989	VC0882		hypothetical protein		
1	CDS	2192144	2192593	+	atgaagatttgggtagacgccga	MKIWVDADACPKVIRETLIRAA	fig|127906.87.peg.1990	VCC1990	VC0881		Protein YaiI	YaiI/YqxD family protein [Vibrio cholerae]	
1	CDS	2194245	2192659	-	atgagttcatctgcttcaataaatc	MSSSASINQNAPKKPLITRFLD	fig|127906.87.peg.1991	VCC1991	VC0880		Multidrug efflux pump component MtrF		
1	CDS	2194571	2194446	-	atgaaagtgctcagctcgctaaa	MKVLSSLKSAKNRHPDCQIVK	fig|127906.87.peg.1992	VCC1992	VC0879	rpmJ	LSU ribosomal protein L36p @ LSU ribosomal protein L36p, zinc-independent		
1	CDS	2194825	2194568	-	atgaaagcaggcattcatcccg	MKAGIHPDYRKVVFHDTTVDH	fig|127906.87.peg.1993	VCC1993	VC0878	rpmE2	LSU ribosomal protein L31p @ LSU ribosomal protein L31p, zinc-independent		
1	CDS	2194960	2195655	+	atgtatcccattgaacctattgca	MYPIEPIAIIESPYQEKFAVPRC	fig|127906.87.peg.1994	VCC1994	VC0876		tRNA (adenine(37)-N6)-methyltransferase		
1	CDS	2195881	2197596	+	atgcgtaccagtaactatcttcttt	MRTSNYLLSTLKETPNDAEVV	fig|127906.87.peg.1995	VCC1995	VC0875	proS	Prolyl-tRNA synthetase (EC 6.1.1.15), bacterial type		
1	CDS	2197805	2197614	-	atgatgaaaaatgaatctaatat	MMKNESNINISFLLEVAAVLAA	fig|127906.87.peg.1996	VCC1996	Not annotated as CDS		hypothetical protein		
1	CDS	2197838	2198482	+	atggatctaaaatcattacttaac	MDLKSLLNQALSSDLVKKGSE	fig|127906.87.peg.1997	VCC1997	VC0873	yebE	hypothetical protein	DUF533 domain-containing protein [Vibrio cholerae]	
1	CDS	2198585	2198788	+	atgaaagtttatgactgttgtgaa	MKVYDCCELVRELYAQIGSGD	fig|127906.87.peg.1998	VCC1998	VC0872	yaeP	UPF0253 protein YaeP	YaeP family protein [Vibrio cholerae]	
1	CDS	2198794	2198985	+	atgaatctcgcgaattttgccact	MNLANFATMDPIMLMSIINMKL	fig|127906.87.peg.1999	VCC1999	VC0871		Uncharacterized protein BT_4646	DUF4250 domain-containing protein [Vibrio cholerae]	
1	repeat	2199030	2199660	+	aataaaaatccgccttcttagaaggcggttttaatataagccccctaaaaggg		fig|127906.87.repeat.40				repeat region		
1	CDS	2199533	2199096	-	atgggcgactacagaagttcatc	MGDYRSSSHVYWRCKYHIVW	fig|127906.87.peg.2000	VCC2000	VC0870		Mobile element protein		
1	CDS	2203601	2199708	-	atgagaattttgcgtggctcccca	MRILRGSPALSEFRVNKLLTAC	fig|127906.87.peg.2001	VCC2001	VC0869	purL	Phosphoribosylformylglycinamidine synthase, synthetase subunit (EC 6.3.5.3) / Phosphoribosylformylglycinamidine synthase, glutamine amidotransferase subunit (EC 6.3.5.3)		
1	CDS	2203804	2203598	-	atgggtcgtaactcaaaattaac	MGRNSKLTKKMTQTVAFGVTS	fig|127906.87.peg.2002	VCC2002	VC0868		FIG01203363: hypothetical protein		
1	CDS	2203789	2203911	+	ttgagttacgacccatttgcacaa	MSYDPFAQIFEQNDSLLSEDF	fig|127906.87.peg.2003	VCC2003	VC0867		hypothetical protein		
1	CDS	2203911	2205503	+	atgactccttttgcctataaactcc	MTPFAYKLPIRALWLGLLSLLL	fig|127906.87.peg.2004	VCC2004	VC0866	mitF	Membrane-bound lytic murein transglycosylase F (EC 4.2.2.n1)		
1	CDS	2205682	2205813	+	atgttgagacgtaaacgtcaatc	MLRRKRQSKLLNKTVSANRRF	fig|127906.87.peg.2005	VCC2005	VC0865		FIG01201025: hypothetical protein		
1	CDS	2206386	2205859	-	ttgtcagcgtctctattttctgctcca	MSASLFSAQDEQFMRRAIVLA	fig|127906.87.peg.2006	VCC2006	VC0864	tadA/yfhC	tRNA-specific adenosine-34 deaminase (EC 3.5.4.33)		
1	CDS	2208068	2206509	-	atgagtaaccaagctatcaatca	MSNQAINQAPSAKPSGMDRF	fig|127906.87.peg.2007	VCC2007	VC0863		Aminobenzoyl-glutamate transport protein		
					ttgtgctgtgattttcgcttctttatgc	MCCDFRFFMLFSHPFASLLLL	From N16961	Not annotated as CDS	VC0862		hypothetical protein		Only annotated as CDS in N16961
1	CDS	2208879	2208466	-	atgcgcaataagcaatctggtttt	MRNKQSGFSLIEVMISFVLIGV	fig|127906.87.peg.2008	VCC2008	VC0861		Type IV fimbrial biogenesis protein PilV		
1	CDS	2210101	2208869	-	atgattcaatatcaaaaaggtgt	MIQYQKGVATLLITAILLSIALV	fig|127906.87.peg.2009	VCC2009	VC0860		hypothetical protein		
1	CDS	2210691	2210101	-	atggctttgaaaagtgctaacca	MALKSANQQGNTLIEFMVAAL	fig|127906.87.peg.2010	VCC2010	VC0859		Type IV fimbrial biogenesis protein PilW		
1	CDS	2211184	2210672	-	atgcatcgcggctttactttgctgg	MHRGFTLLELLITVAVLTTMLL	fig|127906.87.peg.2011	VCC2011	VC0858		Type IV fimbrial biogenesis protein FimT		
1	CDS	2211281	2211724	+	atggaaatgattcgaaaaattttc	MEMIRKIFCKPSVSQQQGMTL	fig|127906.87.peg.2012	VCC2012	VC0857	fimA	Type IV pilus biogenesis protein PilE		
1	CDS	2212919	2211774	-	atgtcaaaacgtgattttacgaa	MSKRDFYEVLGVGRDASERD	fig|127906.87.peg.2013	VCC2013	VC0856	dnaJ	Chaperone protein DnaJ		
1	CDS	2215027	2213120	-	atgggtaaaatcattggtattgac	MGKIIGIDLGTTNSCVAVLDGD	fig|127906.87.peg.2014	VCC2014	VC0855	dnaK	Chaperone protein DnaK		
1	CDS	2215893	2215291	-	atgagcaacgaagagatcaag	MSNEEIKNKDEQLQQDAVETE	fig|127906.87.peg.2015	VCC2015	VC0854	grpE	Heat shock protein GrpE		
1	CDS	2216039	2216923	+	atgaaaaagccgttcaacgttct	MKKPFNVLAIIGKPRDQQAIQT	fig|127906.87.peg.2016	VCC2016	VC0853	ppnK	NAD kinase (EC 2.7.1.23)		
1	CDS	2217053	2218717	+	atgctggctcacttaagtatcaat	MLAHLSINNFAIVKSLQLELSK	fig|127906.87.peg.2017	VCC2017	VC0852	recN	DNA repair protein RecN		
1	CDS	2218876	2219235	+	atgcagtttactaagtggttcatcg	MQFTKWFIALPLAVTALSGCS	fig|127906.87.peg.2018	VCC2018	VC0851	bamE/smpA	Outer membrane beta-barrel assembly protein BamE		
1	CDS	2219621	2219313	-	atgatgagcagtgaaatgattca	MMSSEMIHVEVVYALPHEQRV	fig|127906.87.peg.2019	VCC2019	VC0850		UPF0125 protein RatB	RnfH family protein [Vibrio cholerae]	
1	CDS	2220055	2219621	-	atgaaacaagtgagtcgttctgc	MKQVSRSALVSFSAEQMFHL	fig|127906.87.peg.2020	VCC2020	VC0849	pasT	Ribosome association toxin RatA		
1	CDS	2220239	2220724	+	atgacaaaaaagaaaaccaat	MTKKKTNTKAGSNTIALNKKA	fig|127906.87.peg.2021	VCC2021	VC0848	smpB	tmRNA-binding protein SmpB		
1	CDS	2221326	2222594	+	atggcaaaaaacacagtaaag	MAKNTVKLTDKEIKASKPREK	fig|127906.87.peg.2022	VCC2022	VC0847		Phage integrase		Limited query cover for certain strains
1	CDS	2223314	2223472	+	atgaatgaaatcattaactatgtg	MNEIINYVVNSGVIHANPLAGI	fig|127906.87.peg.2023	VCC2023	No refSeq annotation		Phage integrase		
1	CDS	2223642	2223878	+	ttggttattcttgaagtgattaagc	MVILEVIKPIIGHRTFIFPSSRN	fig|127906.87.peg.2024	VCC2024	VC0846		Phage integrase		Limited query/subject cover for certain strains
1	CDS	2229071	2224509	-	atgaagattcgtattgtatcattga	MKIRIVSLIVLGFLIGCKHESIIT	fig|127906.87.peg.2025	VCC2025	VC0845		Accessory colonization factor AcfD precursor		
1	CDS	2229244	2229891	+	atgcaaaaaactttatcagcaat	MQKTLSAIFLFLTTLSANAAPYI	fig|127906.87.peg.2026	VCC2026	VC0844	acfA	Accessory colonization factor AcfA		
1	CDS	2230802	2229894	-	atgaataagacacttaggattac	MNKTLRITLLSISGSKTFNIKFR	fig|127906.87.peg.2027	VCC2027	VC0843	tagE	TagE protein		
1	CDS	2231284	2230811	-	atgtactctcctatgaaattttcgt	MYSPMKFFVGTNMNQRVSVI	fig|127906.87.peg.2028	VCC2028	VC0842		hypothetical protein	RDD family protein [Vibrio cholerae]	
1	CDS	2232061	2231300	-	atgaaaagtaagaatcgattttta	MKSKNRFLLISLLSFSTSVFAD	fig|127906.87.peg.2029	VCC2029	VC0841	acfC	Accessory colonization factor AcfC		
1	CDS	2233956	2232076	-	atgaagttttctatcaagttactaa	MKFSIKLLMIFTSIIITISSILTYF	fig|127906.87.peg.2030	VCC2030	VC0840	acfB	Accessory colonization factor AcfB		
1	CDS	2234724	2233963	-	atggaatacgtttacttgatcctat	MEYVYLILFSIVSLILGSFSNVV	fig|127906.87.peg.2031	VCC2031	VC0839	pppA/tcpJ	TCP pilin signal peptidase, TcpA processing		
1	CDS	2235554	2234724	-	atgattgggaaaaaatcttttcaa	MIGKKSFQTNVYRMSKFDTYI	fig|127906.87.peg.2032	VCC2032	VC0838	tcpN/toxT	TCP pilus virulence regulatory protein ToxT, transcription activator		
1	CDS	2236777	2235761	-	atgagatataaaaaaaccttaat	MRYKKTLMLSIMITSFNSFAFN	fig|127906.87.peg.2033	VCC2033	VC0837	tcpF	Toxin co-regulated pilus biosynthesis protein F, putative outer membrane channel for TcpA extrusion		
1	CDS	2237809	2236787	-	atgaaaattatctccaagaagta	MKIISKKYRLELYSMLVDLLND	fig|127906.87.peg.2034	VCC2034	VC0836	tcpE	Toxin co-regulated pilus biosynthesis protein E, anchors TcpT to membrane		
1	CDS	2239295	2237784	-	atgtcaattgatataaagtatttaa	MSIDIKYLSRIDIDREEFFFKDS	fig|127906.87.peg.2035	VCC2035	VC0835	tcpT	Toxin co-regulated pilus biosynthesis protein T, putative ATP-binding translocase of TcpA		
1	CDS	2239743	2239285	-	atgaatatcaaactatcatttattt	MNIKLSFISIAFLSLSFNVAANE	fig|127906.87.peg.2036	VCC2036	VC0834	tcpS	Toxin co-regulated pilus biosynthesis protein S		
1	CDS	2240580	2239759	-	atgaagattagcagtttgaagaa	MKISSLKKGSNFSINIKNIKLDK	fig|127906.87.peg.2037	VCC2037	VC0833	tcpD	Toxin co-regulated pilus biosynthesis protein D		
1	CDS	2241038	2240583	-	atgactagcatatggttacatgac	MTSIWLHESDFRYVNLDVERY	fig|127906.87.peg.2038	VCC2038	VC0832	tcpR	Toxin co-regulated pilus biosynthesis protein R		Limited query cover for certain strains
1	CDS	2242500	2241031	-	atgaaaaaaacaataatcagta	MKKTIISTLVIGLVSGCSNTNLL	fig|127906.87.peg.2039	VCC2039	VC0831	tcpC	Toxin co-regulated pilus biosynthesis protein C, outer membrane protein		
1	CDS	2242955	2242503	-	atgaaaaaaaactattacatagc	MKKNYYIGACIALILSGPTFAQ	fig|127906.87.peg.2040	VCC2040	VC0830	tcpQ	Toxin co-regulated pilus biosynthesis protein Q		Limited query cover for certain strains
1	CDS	2244247	2242955	-	atgagaaaataccaacaaggtg	MRKYQQGVGLLEAILASAVLG	fig|127906.87.peg.2041	VCC2041	VC0829	tcpB	Toxin co-regulated pilus biosynthesis protein B		
1	CDS	2245001	2244327	-	atgcaattattaaaacagcttttta	MQLLKQLFKKKFVKEEHDKKT	fig|127906.87.peg.2042	VCC2042	VC0828	tcpA	Toxin co-regulated pilin A		
1	CDS	2245954	2245544	-	atgcacaaaaaattaaaagcttg	MHKKLKAWGGATGLFVVALG	fig|127906.87.peg.2043	VCC2043	VC0827	tcpH	Toxin co-regulated pilus biosynthesis protein H, transcriptional activator of ToxT promoter		Limited query cover for certain strains
1	CDS	2246604	2245939	-	atggggtatgtccgcgtgatttatc	MGYVRVIYQFPDNLWWNECT	fig|127906.87.peg.2044	VCC2044	VC0826	tcpP	Toxin co-regulated pilus biosynthesis protein P, transcriptional activator of ToxT promoter		
1	CDS	2247045	2248907	+	atgataaaaaaaataatttcggt	MIKKIISVFLLLACIITLAFTAFFY	fig|127906.87.peg.2045	VCC2045	VC0825	tcpI	Toxin co-regulated pilus biosynthesis protein I, chemoreceptor, negative regulator of TcpA		
1	CDS	2249677	2249183	-	atgactgttacatttcaaaataatc	MTVTFQNNPVSISGSFPKVGD	fig|127906.87.peg.2046	VCC2046	VC0824	tpx	Thiol peroxidase, Tpx-type (EC 1.11.1.15)		
1	CDS	2250725	2249787	-	atgagaaaatcgatattgattag	MRKSILISLCLFSSITNACPIGD	fig|127906.87.peg.2047	VCC2047	VC0823		hypothetical protein	peptidase [Vibrio cholerae]	
1	CDS	2255323	2250722	-	atgttcaataaaaaaattacaat	MFNKKITITSTGITLSIFSFMAK	fig|127906.87.peg.2048	VCC2048	VC0822		Putative inner membrane protein		ORF is split into 2 ORFs in N16961. In KEGG ORF in N16961 is annotated as 1 pseudogene ORF named VC0821.
									VC0821				ORF is split into 2 ORFs in N16961. In KEGG ORF in N16961 is annotated as 1 pseudogene ORF named VC0821.
1	CDS	2258441	2255436	-	gtggtaagatattcactcttaatga	MVRYSLLMKVSFAILIFLVGCN	fig|127906.87.peg.2049	VCC2049	VC0820	tagA	Lipoprotein, ToxR-activated gene, TagA		
1	CDS	2258658	2260178	+	atgatttatccaattcctaatagcg	MIYPIPNSETSTVHFKDVYDNY	fig|127906.87.peg.2050	VCC2050	VC0819		Aldehyde dehydrogenase (EC 1.2.1.3)		
1	CDS	2260520	2260738	+	atgccgaaacctcgctacaaaa	MPKPRYKTTNWKQYNKALINF	fig|127906.87.peg.2051	VCC2051	No refSeq annotation		Mobile element protein		
1	CDS	2261060	2261200	+	atgtatcgagtgaagcagttgtta	MYRVKQLLGGRLSLRNYNAQ	fig|127906.87.peg.2052	VCC2052	No refSeq annotation		Mobile element protein		
								Not annotated as CDS	VC0818				Only present in N16961
1	CDS	2262203	2261226	-	atggacagagaaatccaacaa	MDREIQQRLQWVKMYEECGD	fig|127906.87.peg.2053	VCC2053	VC0817		Transposase		
					ttggctttaagtggttcagagcgtctcatcaagattcactttgttcatttaaggcaa		Only in N16961	Not annotated as CDS	VC0816		hypothetical protein		Only present in N16961
1	CDS	2262807	2264168	+	atggaagagctaaccgtacaag	MEELTVQAFIRGEWIDIGIISFP	fig|127906.87.peg.2054	VCC2054	VC0815		hypothetical protein	type II toxin-antitoxin system HipA family toxin [Vibrio cholerae]	
1	CDS	2264165	2264632	+	atgaagccaagcaataaagag	MKPSNKESVQDTVARLRENR	fig|127906.87.peg.2055	VCC2055	VC0814		Predicted transcriptional regulator	XRE family transcriptional regulator [Vibrio cholerae]	
1	CDS	2265060	2265638	+	atgtctgtagaattttacaatcaa	MSVEFYNQNAQQFFSSTVEV	fig|127906.87.peg.2056	VCC2056	VC0813	tehB	Tellurite resistance protein-related protein		
1	CDS	2265642	2268578	+	atgagtgatagacaaagttttctg	MSDRQSFLYSGHKLSSGGAN	fig|127906.87.peg.2057	VCC2057	VC0812		Helicase-related protein		
1	CDS	2268648	2269352	+	atgatagtaaaagaaaaaca	MIVKEKTHIKTSNFKQDLGNQ	fig|127906.87.peg.2058	VCC2058	VC0811		hypothetical protein	NERD domain-containing protein [Vibrio cholerae]	
1	CDS	2270195	2269686	-	atgaatagagcacatgggttttc	MNRAHGFFLFLLSIPTAIISALT	fig|127906.87.peg.2059	VCC2059	VC0810		hypothetical protein		
1	repeat	2270243	2270372	+	tttatacccttcctacttgaagctgcagcggtgttggctacgctcgttcaccccac		fig|127906.87.repeat.41				repeat region		
1	CDS	2272072	2270393	-	atgcctagccactcctcgtttttag	MPSHSSFLDISALFEQCEQSS	fig|127906.87.peg.2060	VCC2060	VC0809		hypothetical protein	SWIM zinc finger domain-containing protein [Vibrio cholerae]	
					atgcaatttttagctttactgcccaacacctgcttacaattaacccaacgagcc		from N16961	Not annotated as CDS	VC0808				
1	CDS	2272295	2272978	+	atgaaatccaaccaaagcttac	MKSNQSLLSNVLINIVIPVVILS	fig|127906.87.peg.2061	VCC2061	VC0807		hypothetical protein	MFS transporter [Vibrio metoecus]	
1	CDS	2274662	2273046	-	atgatacgtttaactgaacttaag	MIRLTELKLPLNHEEGALLDAI	fig|127906.87.peg.2062	VCC2062	VC0806		NAD(FAD)-utilizing dehydrogenase, sll0175 homolog		
1	CDS	2274909	2275232	+	atggcttttgtcgtcaccgataac	MAFVVTDNCIQCKYTDCVAVC	fig|127906.87.peg.2063	VCC2063	VC0804		4Fe-4S dicluster fused with DUF3470		
1	CDS	2276145	2275411	-	atgatttcgaaaacccaactcaa	MISKTQLKLIRALGQKKQRKAH	fig|127906.87.peg.2064	VCC2064	VC0803		RNA methyltransferase, TrmH family		
1	CDS	2276657	2276259	-	atgtcaggaatcaaaagcttaga	MSGIKSLELLLQSMSPELMAG	fig|127906.87.peg.2065	VCC2065	VC0802		hypothetical protein	ACT domain-containing protein [Vibrio cholerae]	
1	repeat	2276779	2276910	+	tgttatacccaaactacttggagttgcaggtaggcggcaagtgagttcatccc		fig|127906.87.repeat.42				repeat region		
1	CDS	2277945	2277004	-	atgactattcctgctgcgcttgatc	MTIPAALDLLLELPSQASSSSG	fig|127906.87.peg.2066	VCC2066	VC0801	citG	Triphosphoribosyl-dephospho-CoA synthase (EC 2.4.2.52)		
1	CDS	2278459	2277923	-	atgagtcaccatccggatctttca	MSHHPDLSVSLDQLLLRKEVR	fig|127906.87.peg.2067	VCC2067	VC0800	citX	Citrate lyase holo-[acyl-carrier-protein] synthase (EC 2.7.7.61)		
1	CDS	2279985	2278456	-	atgagtatcaatacattaaataac	MSINTLNNPLSVLEVERYALTF	fig|127906.87.peg.2068	VCC2068	VC0799	citF	Citrate lyase alpha chain (EC 4.1.3.6)		
1	CDS	2280871	2279996	-	atgaccaaacttcgtcgcagtat	MTKLRRSMLFVPGANAAMLSI	fig|127906.87.peg.2069	VCC2069	VC0798	citE	Citrate lyase beta chain (EC 4.1.3.6)		
1	CDS	2281164	2280868	-	atgaaaattgcccatcccgccttt	MKIAHPAFAGTLESSDLQVRIE	fig|127906.87.peg.2070	VCC2070	VC0797	citD	Citrate lyase gamma chain, acyl carrier protein		
1	CDS	2282271	2281216	-	atggtggatatttataccttctcgc	MVDIYTFSRVSTKNRTKLLQIK	fig|127906.87.peg.2071	VCC2071	VC0796	citC	[Citrate [pro-3S]-lyase] ligase (EC 6.2.1.22)		
1	CDS	2282548	2283894	+	atgaacgagaaaaccctacatt	MNEKTLHLDKKSAVVGGLNLS	fig|127906.87.peg.2072	VCC2072	VC0795	citS	Citrate-sodium symporter (TC 2.A.24.1.1)		
1	CDS	2283887	2284261	+	ttgtttaacccttttaacccttgctcc	MFNPLTLAPWDYLARLARLFF	fig|127906.87.peg.2073	VCC2073	VC0794		Oxaloacetate decarboxylase Na(+) pump, gamma chain (EC 4.1.1.3)		
									No refSeq annotation				ORF is split into 2 ORFs in N16961, only one has refseq annotation in PATRIC. In KEGG ORF in N16961 is annotated as 1 ORF named VC0793m.
1	CDS	2284280	2286079	+	atgactcaggcgatcaaacgcg	MTQAIKRVGVTDVVLRDAHQS	fig|127906.87.peg.2074	VCC2074	VC0793m		Oxaloacetate decarboxylase Na(+) pump, alpha chain (EC 4.1.1.3)		ORF is split into 2 ORFs in N16961, only one has refseq annotation in PATRIC. In KEGG ORF in N16961 is annotated as 1 ORF named VC0793m.
1	CDS	2286095	2287396	+	atggaaaatattttagccatggtg	MENILAMVRDFGLFHLQWGQ	fig|127906.87.peg.2075	VCC2075	VC0792		Oxaloacetate decarboxylase Na(+) pump, beta chain (EC 4.1.1.3)		
1	CDS	2287467	2289164	+	atgaaaatatcactagcgcaatt	MKISLAQFLHTKVCQTLSFQQ	fig|127906.87.peg.2076	VCC2076	VC0791	citA	Tricarboxylate transport sensor protein TctE => Citrate response regulator CitA		
1	CDS	2289178	2289861	+	atgatggaattaatcgacgtattg	MMELIDVLIVEDETSIADVHSF	fig|127906.87.peg.2077	VCC2077	VC0790	citB	Tricarboxylate transport transcriptional regulator TctD => Citrate response regulator CitB		
1	CDS	2290167	2289895	-	atgttagtaagaaaagtttctaga	MLVRKVSRDLASAQARENWL	fig|127906.87.peg.2078	VCC2078	VC0789		FIG01200572: hypothetical protein		
1	CDS	2290662	2290333	-	atgttccatgcccatgtttattttga	MFHAHVYFDPDQAIVAERFRC	fig|127906.87.peg.2079	VCC2079	VC0788		Aromatic ring-cleaving dioxygenase		
1	CDS	2291719	2290787	-	atgttaaaaagccagcaagttgc	MLKSQQVASLHTFMQVAQYT	fig|127906.87.peg.2080	VCC2080	VC0787		Transcriptional regulator, LysR family		
1	CDS	2291823	2293085	+	atggaagttttggttttaggcagtg	MEVLVLGSGVVGLTSAWYLA	fig|127906.87.peg.2081	VCC2081	VC0786	dadA	D-amino acid dehydrogenase (EC 1.4.99.6)		
1	CDS	2293463	2295013	+	atgcagtcatttgttgattttctgaa	MQSFVDFLNGIIWSPALIYLCL	fig|127906.87.peg.2082	VCC2082	VC0784		Putative sodium/proton-dependent alanine carrier protein YrbD		
1	CDS	2297075	2295093	-	atgaaaacagtctttaaacccac	MKTVFKPTLLAVLVAASAPVLA	fig|127906.87.peg.2083	VCC2083	VC0783		Alkyl sulfatase and related hydrolases, MBL-fold metallo-hydrolase superfamily		
1	CDS	2298213	2297293	-	atgctgcgtgattatgatctaaac	MLRDYDLNLLTVFDSVMKLGS	fig|127906.87.peg.2084	VCC2084	VC0781		Transcriptional regulator near Vibriobactin biosynthetic gene custer		
1	CDS	2298942	2298229	-	atggcttttatgatttcaatgccag	MAFMISMPAIELAGLTIQVCQF	fig|127906.87.peg.2085	VCC2085	VC0780	vibD	Phosphopantetheinyl transferase component of siderophore synthetase (EC 2.7.8.-)		
1	CDS	2299784	2298945	-	atggacacaatttgggatgctga	MDTIWDADLQSAALKAEHHRL	fig|127906.87.peg.2086	VCC2086	VC0779	yusV/viuC	Ferric vibriobactin, enterobactin transport system, ATP-binding protein ViuC (TC 3.A.1.14.6)		Limited query cover for certain strains
1	CDS	2300977	2299901	-	atggaaactatcacatctgtagc	METITSVAPEGRSNQTRFWLV	fig|127906.87.peg.2087	VCC2087	VC0778	fepG/viuG	Ferric vibriobactin, enterobactin transport system, permease protein ViuG (TC 3.A.1.14.6)		
1	CDS	2302008	2300956	-	atgaatgagctgatgggaataa	MNELMGIRVASSKRENPTLAG	fig|127906.87.peg.2088	VCC2088	VC0777	yfiZ/viuD	Ferric vibriobactin, enterobactin transport system, permease protein ViuD (TC 3.A.1.14.6)		
1	CDS	2302964	2302005	-	atgaaaaacatttttattttattgtct	MKNIFILLSVILGCLFSGFVSAC	fig|127906.87.peg.2089	VCC2089	VC0776	fepB/viuP	Ferric vibriobactin, enterobactin transport system, substrate-binding protein ViuP (TC 3.A.1.14.6)		
1	CDS	2303166	2304476	+	atgttattggctcaaaagcctttttc	MLLAQKPFWQRHLAYPHINLD	fig|127906.87.peg.2090	VCC2090	VC0775	dhbF/vibH	Amide synthase component of siderophore synthetase		
1	CDS	2305267	2304488	-	atgtcgttggttaacagcaaggtt	MSLVNSKVLLVGSARGIGFSV	fig|127906.87.peg.2091	VCC2091	VC0774	dhbA	2,3-dihydro-2,3-dihydroxybenzoate dehydrogenase (EC 1.3.1.28) of siderophore biosynthesis @ 2,3-dihydro-2,3-dihydroxybenzoate dehydrogenase (EC 1.3.1.28)		
1	CDS	2305444	2306631	+	atggtcatgaagcgtgaagtggt	MVMKREVVGYTTMSEHLIDTC	fig|127906.87.peg.2092	VCC2092	VC0773	dhbC/vibC	Isochorismate synthase (EC 5.4.4.2) @ Isochorismate synthase (EC 5.4.4.2) of siderophore biosynthesis		
1	CDS	2306642	2308273	+	atgacaaccgattttacccccttgg	MTTDFTPWPEALAAQYRQLG	fig|127906.87.peg.2093	VCC2093	VC0772	dhbE/vibE	2,3-dihydroxybenzoate-AMP ligase (EC 2.7.7.58) of siderophore biosynthesis @ 2,3-dihydroxybenzoate-AMP ligase (EC 2.7.7.58)		
1	CDS	2309255	2308374	-	atggctattcctaaaatcgcttctt	MAIPKIASYPLPVSLPTNKVDW	fig|127906.87.peg.2094	VCC2094	VC0771	vibB	Isochorismatase (EC 3.3.2.1) of siderophore biosynthesis		
1	CDS	2310160	2309567	-	atgtcaactaagctagccaacc	MSTKLANPAPLGLMGFGMTTI	fig|127906.87.peg.2095	VCC2095	VC0770	yaaH	Succinate-acetate/proton symporter SatP		
1	CDS	2312054	2310351	-	atgtttaaactcaaacataccgc	MFKLKHTAWWVAMACALPAC	fig|127906.87.peg.2096	VCC2096	VC0769		Chitinase (EC 3.2.1.14)		
1	CDS	2313864	2312311	-	atgactaaaaatattcatgacca	MTKNIHDQRILILDFGSQYTQL	fig|127906.87.peg.2097	VCC2097	VC0768	guaA	GMP synthase [glutamine-hydrolyzing], amidotransferase subunit (EC 6.3.5.2) / GMP synthase [glutamine-hydrolyzing], ATP pyrophosphatase subunit (EC 6.3.5.2)		
1	CDS	2315434	2313971	-	atgctacgaatcgcaaaagaag	MLRIAKEALTFDDVLLVPAHST	fig|127906.87.peg.2098	VCC2098	VC0767	guaB	Inosine-5'-monophosphate dehydrogenase (EC 1.1.1.205) / CBS domain		
1	CDS	2315642	2316982	+	ttgtcttcttctctcgccaatcgaaa	MSSSLANRNIYTVSRLNSEVR	fig|127906.87.peg.2099	VCC2099	VC0766	xseA	Exodeoxyribonuclease VII large subunit (EC 3.1.11.6)		
1	CDS	2317196	2316963	-	atggacaacccatgtccaacatc	MDNPCPTCGQELEWTGQYH	fig|127906.87.peg.2100	VCC2100	VC0765		Uncharacterized protein YfgJ	DNA ligase [Vibrio cholerae]	
1	CDS	2318385	2317501	-	atgacctattgcgcaacagaag	MTYCATEVTFPQGITSLETQV	fig|127906.87.peg.2101	VCC2101	VC0764		Predicted metal-dependent hydrolase	alanyl-tRNA editing protein [Vibrio cholerae]	
1	CDS	2319923	2318439	-	atggtacctgttgttgctcttgttgg	MVPVVALVGRPNVGKSTLFN	fig|127906.87.peg.2102	VCC2102	VC0763	engA	GTP-binding protein EngA		
1	CDS	2321276	2320116	-	atgaagaagctgttcaatcaagt	MKKLFNQVLVAAGVLALLAGC	fig|127906.87.peg.2103	VCC2103	VC0762	bamB	Outer membrane beta-barrel assembly protein BamB		
1	CDS	2321903	2321289	-	gtggaactctacgatactgaaga	MELYDTEEQQVEAIKDWWKE	fig|127906.87.peg.2104	VCC2104	VC0761		UPF0070 protein YfgM	tetratricopeptide repeat protein [Vibrio cholerae]	
1	CDS	2323178	2321940	-	atgaacgattgtctcccaaccca	MNDCLPTQSPLWQKVEGVVK	fig|127906.87.peg.2105	VCC2105	VC0760	hisS	Histidyl-tRNA synthetase (EC 6.1.1.21)		
1	CDS	2324364	2323234	-	atgtctatgcaacatgagtctcct	MSMQHESPIKRRPSTRIYVGD	fig|127906.87.peg.2106	VCC2106	VC0759	ispG	(E)-4-hydroxy-3-methylbut-2-enyl-diphosphate synthase (flavodoxin) (EC 1.17.7.3)		
1	CDS	2325451	2324369	-	atgaatactgaacagcaagatta	MNTEQQDYNQTETTEQVNQF	fig|127906.87.peg.2107	VCC2107	VC0758	rodZ	Cytoskeleton protein RodZ		
1	CDS	2326886	2325765	-	atgaccacagaaaaaatcaatc	MTTEKINLLDFDRKGLRTFFAE	fig|127906.87.peg.2108	VCC2108	VC0757	rlmN	23S rRNA (adenine(2503)-C(2))-methyltransferase @ tRNA (adenine(37)-C(2))-methyltransferase (EC 2.1.1.192)		
1	CDS	2327488	2327060	-	atggctctagaaagaacattttcg	MALERTFSIIKPDAVKRNLIGEI	fig|127906.87.peg.2109	VCC2109	VC0756	ndk	Nucleoside diphosphate kinase (EC 2.7.4.6)		
1	CDS	2328981	2327692	-	atgtctacacagatgtctgtatttt	MSTQMSVFLSTQAAQPQWGE	fig|127906.87.peg.2110	VCC2110	VC0755	pepB	Peptidase B (EC 3.4.11.23)		
1	CDS	2329395	2329201	-	atgaaatggacagattcacgcg	MKWTDSRDIAIELCDKFPDVD	fig|127906.87.peg.2111	VCC2111	VC0754		Protein IscX, believed to be involved in assembly of Fe-S clusters		
1	CDS	2329782	2329444	-	atgccaaagattattgttttgcctc	MPKIIVLPHETLCPEGAVLEAC	fig|127906.87.peg.2112	VCC2112	VC0753	fdc	Ferredoxin, 2Fe-2S		
1	CDS	2331646	2329796	-	atggcattacttcagattgcagac	MALLQIAEPGQSSAPHQHKLA	fig|127906.87.peg.2113	VCC2113	VC0752	hscA	Chaperone protein HscA		
1	CDS	2332185	2331670	-	atgaattattttgaattatttgggct	MNYFELFGLPIQFELDGSLLS	fig|127906.87.peg.2114	VCC2114	VC0751	hscB	Chaperone protein HscB		
1	CDS	2332557	2332234	-	atggccgtcactcttagcgaaac	MAVTLSETAASRVKTFLDNRG	fig|127906.87.peg.2115	VCC2115	VC0750	iscA	Iron-sulfur cluster assembly iron binding protein IscA		
1	CDS	2333001	2332618	-	atggcatacagcgaaaaggtca	MAYSEKVIDHYENPRNVGSFD	fig|127906.87.peg.2116	VCC2116	VC0749	nifU	Iron-sulfur cluster assembly scaffold protein IscU		
1	CDS	2334252	2333038	-	atgaaactgcctatttatctagatt	MKLPIYLDYSATCPVDPRVAE	fig|127906.87.peg.2117	VCC2117	VC0748	iscS	Cysteine desulfurase (EC 2.8.1.7) => IscS		
1	CDS	2334845	2334279	-	atggaaaacttccagccacata	MENFQPHKQCGYGVIMRLTS	fig|127906.87.peg.2118	VCC2118	VC0747	iscR	Iron-sulfur cluster regulator IscR		
1	CDS	2335649	2334915	-	atgatgttagaacgcgtgaaagt	MMLERVKVVLVGTTHSGNIGS	fig|127906.87.peg.2119	VCC2119	VC0746	trmJ	tRNA (cytidine(32)/uridine(32)-2'-O)-methyltransferase (EC 2.1.1.200)		
1	CDS	2335828	2336631	+	atgcatccaatgctaaatatcgc	MHPMLNIAIRAARKAGNHIAKS	fig|127906.87.peg.2120	VCC2120	VC0745	suhB	Inositol-1-monophosphatase (EC 3.1.3.25)		
1	CDS	2337746	2336799	-	atgtttcaaatcctaaaagcaga	MFQILKADKMIDFMRWSKFAF	fig|127906.87.peg.2121	VCC2121	VC0744	secF	Protein translocase subunit SecF		
1	CDS	2339614	2337761	-	gtgctaaaccgttatccgttatgg	MLNRYPLWKYLMVMLTIAVAA	fig|127906.87.peg.2122	VCC2122	VC0743	secD	Protein translocase subunit SecD		
1	CDS	2339968	2339636	-	atgagtttaatttctgtagcacatg	MSLISVAHAAGEGAPQGGGF	fig|127906.87.peg.2123	VCC2123	VC0742	yajC	Protein translocase subunit YajC		
1	CDS	2341212	2340073	-	gtgaaattaaaatttgaactgaa	MKLKFELKKKNGNARRGQLIF	fig|127906.87.peg.2124	VCC2124	VC0741	tgt	Queuine tRNA-ribosyltransferase (EC 2.4.2.29)		
1	CDS	2342499	2341441	-	atgcaagtttcagattttcattttga	MQVSDFHFELPDELIARYPKA	fig|127906.87.peg.2125	VCC2125	VC0739	queA	S-adenosylmethionine:tRNA ribosyltransferase-isomerase (EC 2.4.99.17)		
1	CDS	2342789	2343238	+	atgatcaaagttgaagacatgat	MIKVEDMMTRHPHTLLRTHTL	fig|127906.87.peg.2126	VCC2126	VC0737		CBS domain protein		
1	CDS	2344704	2343391	-	atgacactgactcgtcgccaac	MTLTRRQQIEALEKDWATNPF	fig|127906.87.peg.2127	VCC2127	VC0736	aceA	Isocitrate lyase (EC 4.1.3.1)		
1	CDS	2346471	2344834	-	atgtcaatcaatactgctgaatta	MSINTAELTQHTTETKITQGKL	fig|127906.87.peg.2128	VCC2128	VC0734	aceB	Malate synthase (EC 2.3.3.9)		
1	CDS	2347960	2347055	-	atgaataaatggccaagcctca	MNKWPSLKQLSYLVTLYETRH	fig|127906.87.peg.2129	VCC2129	VC0732		Transcriptional regulator VC0732, LysR family		
1	CDS	2348182	2348787	+	atggtactagttggtcgcaaagc	MVLVGRKAPDFTAAAVLGNGI	fig|127906.87.peg.2130	VCC2130	VC0731		Alkyl hydroperoxide reductase subunit C-like protein		
1	CDS	2349122	2349886	+	atgaaatatcaagttgaagtctgt	MKYQVEVCIDNIESLHNAIAGG	fig|127906.87.peg.2131	VCC2131	VC0730	cutC	Cytoplasmic copper homeostasis protein CutC		
1	repeat	2349892	2350017	+	tatacccaaacgacttggcgttgcaggtaggcggcaagtgagttcatcccca		fig|127906.87.repeat.43				repeat region		
1	CDS	2350089	2350865	+	atggcgaaattggataagaaag	MAKLDKKVYERELELLQIELVK	fig|127906.87.peg.2132	VCC2132	VC0728		Polyphosphate kinase 2 (EC 2.7.4.1)		
1	CDS	2351636	2350926	-	atgcagttcggtcgacatatttca	MQFGRHISGQFNVELESIRSQ	fig|127906.87.peg.2133	VCC2133	VC0727	phoU	Phosphate transport system regulatory protein PhoU		
1	CDS	2352491	2351670	-	atgtactccgccaatcctactttgg	MYSANPTLGYYVPPLDVNNLT	fig|127906.87.peg.2134	VCC2134	VC0726	pstB	Phosphate ABC transporter, ATP-binding protein PstB (TC 3.A.1.7.1)		
1	CDS	2354140	2352494	-	atgttgaattggtttaaatccggtc	MLNWFKSGAPWIWLTGGAVS	fig|127906.87.peg.2135	VCC2135	VC0725		Phosphate ABC transporter, permease protein PstA (TC 3.A.1.7.1)		
1	CDS	2356345	2354150	-	atggtcagacagccattttcattg	MVRQPFSLKERDRARLVKDR	fig|127906.87.peg.2136	VCC2136	VC0724		Phosphate ABC transporter, permease protein PstC (TC 3.A.1.7.1)		
1	CDS	2356544	2358649	+	atgagtgcggataagctgtacat	MSADKLYIDKELSLWLSFNERV	fig|127906.87.peg.2137	VCC2137	VC0723	ppk	Polyphosphate kinase (EC 2.7.4.1)		

1	CDS	2358630	2360132	+	atgacaacagtagtcagtaacg	MTTVVSNAREIAAIDLGSNSFH	fig|127906.87.peg.2138	VCC2138	VC0722	ppx	Exopolyphosphatase (EC 3.6.1.11)		
1	CDS	2361168	2360200	-	atgctaagtcgcgttggttggttga	MLSRVGWLSSLVLVSSTAWAI	fig|127906.87.peg.2139	VCC2139	VC0721	pstS	Phosphate ABC transporter, substrate-binding protein PstS (TC 3.A.1.7.1)		
1	CDS	2362475	2361174	-	atggttgaacgtttaacatggaaa	MVERLTWKKLAWELAFFYLPV	fig|127906.87.peg.2140	VCC2140	VC0720	phoR	Phosphate regulon sensor protein PhoR (SphS) (EC 2.7.13.3)		
1	CDS	2363186	2362497	-	atgtctagaaggattctggttgttg	MSRRILVVEDEAPIREMLCFVL	fig|127906.87.peg.2141	VCC2141	VC0719	phoB	Phosphate regulon transcriptional regulatory protein PhoB (SphR)		
1	CDS	2363423	2364337	+	atgtggtttaaaaactgtatggtg	MWFKNCMVYRVNREVNFNAD	fig|127906.87.peg.2142	VCC2142	VC0718	rdgC	DNA recombination-dependent growth factor RdgC		
1	CDS	2364488	2365888	+	atgacaacgccaaaaaccttttg	MTTPKTFVPELLSPAGSLKNM	fig|127906.87.peg.2143	VCC2143	VC0717		Uncharacterized protease YegQ	U32 family peptidase [Vibrio cholerae]	
1	CDS	2365891	2366145	+	atggcgctactgattaccgccaa	MALLITAKCTNCDMCEPECPN	fig|127906.87.peg.2144	VCC2144	VC0716	yfhL	Uncharacterized ferredoxin-like protein YfhL	YfhL family 4Fe-4S dicluster ferredoxin [Vibrio cholerae]	
1	CDS	2366902	2366180	-	atgaatcctgttattgataccatac	MNPVIDTILSHRSIRAFTQEPIS	fig|127906.87.peg.2145	VCC2145	VC0715	frp	NADPH-flavin oxidoreductase		
1	CDS	2367235	2366912	-	atgcctcatttacgttttcgcgcag	MPHLRFRAVEAHIVESLVPTLL	fig|127906.87.peg.2146	VCC2146	VC0714		hypothetical protein	DUF1904 family protein [Vibrio cholerae]	
1	repeat	2367393	2367520	+	aaaaaaaccagtcctaagactgggtttcgtaataagtggcggagcggacgg		fig|127906.87.repeat.44				repeat region		
1	rna	2367505	2367429	-	ggagcggtagttcagtcggttagaataccggcctgtcacgccgggggtcgcg		fig|127906.87.rna.58	VCCt045	VCt050		tRNA-Asp-GTC		
1	repeat	2367576	2373044	+	ttaaagcctggcgatgttctactctcacatggggagaccccacactaccatcg		fig|127906.87.repeat.45				repeat region		
1	rna	2367699	2367578	-	ttgcttggcgaccatagcgttttggacccacctgattccatcccgaactcagaa		fig|127906.87.rna.59	VCr013	VCr015		5S rRNA ## 5S ribosomal RNA		
1	rna	2370712	2367824	-	ggttaagtgactaagcgtacacggtggatgcctgggcagtcagaggcgatg		fig|127906.87.rna.60	VCr014	VCr014		LSU rRNA ## 23S rRNA, large subunit ribosomal RNA		
1	rna	2371075	2371000	-	gtcccgttcgtctagaggcctaggacaccgccctttcacggcggtaacaggg		fig|127906.87.rna.61	VCCt046	VCt049		tRNA-Glu-TTC		
									VCr013				
1	CDS	2372852	2372655	-	gtgtgggcactcgtcgatgataa	MWALVDDNQKRFINELSDHLN	fig|127906.87.peg.2147	VCC2147	VC0713		hypothetical protein		
1	CDS	2375779	2373206	-	atgcgtcttgacagatttacgagg	MRLDRFTSKFQIAISDAQSLAL	fig|127906.87.peg.2148	VCC2148	VC0711		Chaperone protein ClpB (ATP-dependent unfoldase)		
1	CDS	2376626	2375904	-	atgattctccccaactggaatgca	MILPNWNAPKQVKAFSSTRHC	fig|127906.87.peg.2149	VCC2149	VC0710	yfiH	FIG00003370: Multicopper polyphenol oxidase		
1	CDS	2377603	2376629	-	atggctcatcagattgaattaact	MAHQIELTQTVKDSQLGQRLD	fig|127906.87.peg.2150	VCC2150	VC0709	rluD	LSU rRNA pseudouridine(1911/1915/1917) synthase (EC 5.4.99.23)		
1	CDS	2377758	2378483	+	atgaaataccagactttatcagg	MKYQTLSGLLALSLLFGCSSS	fig|127906.87.peg.2151	VCC2151	VC0708	bamD	Outer membrane beta-barrel assembly protein BamD		
1	CDS	2378885	2379211	+	atgaaaatcaacatcactggtaa	MKINITGKNIEVTSAIRNHIEGK	fig|127906.87.peg.2152	VCC2152	VC0706	raiA	Ribosome-associated inhibitor A		
1	CDS	2379451	2380626	+	atgacagacaaacaatactcac	MTDKQYSLDDIRLRLNELDDQ	fig|127906.87.peg.2153	VCC2153	VC0705	pheA	Chorismate mutase I (EC 5.4.99.5) / Prephenate dehydratase (EC 4.2.1.51)		
1	CDS	2380769	2381848	+	gtgaccaatttttgtaacgaatgg	MTNFCNEWVSYSQMIKRFLSL	fig|127906.87.peg.2154	VCC2154	VC0704	potF	Predicted polyamine sensor NspS, involved in biofilm formation		
1	CDS	2381832	2384207	+	atgaagctaaaccatagaattct	MKLNHRILLLIAPVILLSAAASS	fig|127906.87.peg.2155	VCC2155	VC0703		c-di-GMP phosphodiesterase (EC 3.1.4.52) => biofilm repressor MbaM		
1	CDS	2384194	2384679	+	gtgaatgccgttagaagtgcctttt	MNAVRSAFSTVFPDQEWEFIC	fig|127906.87.peg.2156	VCC2156	VC0702		Inosine/xanthosine triphosphatase		
1	CDS	2385068	2384757	-	atggtacagcaaccttcttttagtg	MVQQPSFSEWQQVLDLVKQA	fig|127906.87.peg.2157	VCC2157	VC0701	trpR	Transcriptional repressor protein TrpR		
1	CDS	2387069	2385123	-	atgacgcgcctgacggtatttaa	MTRLTVFKPRSLVSILMFTTVW	fig|127906.87.peg.2158	VCC2158	VC0700	slt	Soluble lytic murein transglycosylase (EC 4.2.2.n1)		
1	CDS	2387431	2389098	+	atggctgaatacgtatataccatg	MAEYVYTMSRVSKIVPPKRQI	fig|127906.87.peg.2159	VCC2159	VC0698		Energy-dependent translational throttle protein EttA		
1	CDS	2389269	2389640	+	atgatcgaaaaacgtcgcttttca	MIEKRRFSRILYQAPATLSQGI	fig|127906.87.peg.2160	VCC2160	VC0697		FIG01200641: hypothetical protein	PilZ domain-containing protein [Vibrio cholerae]	
1	CDS	2390834	2389707	-	atggccgtagagttgaatcagtta	MAVELNQLRDQIDEVDKQMVE	fig|127906.87.peg.2161	VCC2161	VC0696	tyrA	Chorismate mutase I (EC 5.4.99.5) / Cyclohexadienyl dehydrogenase (EC 1.3.1.12)(EC 1.3.1.43)		
1	CDS	2391920	2390847	-	atgaaaaaaagtgaattgagtg	MKKSELSDVNISNEQILITPDA	fig|127906.87.peg.2162	VCC2162	VC0695	aroF	2-keto-3-deoxy-D-arabino-heptulosonate-7-phosphate synthase I alpha (EC 2.5.1.54)		
1	CDS	2392162	2393838	+	atggatctcatcctctcattactgc	MDLILSLLQQMCVYLVLAYML	fig|127906.87.peg.2163	VCC2163	VC0694	yehU	Sensor histidine kinase BtsS		
1	CDS	2393848	2394561	+	atgttatctgcactactcattgacg	MLSALLIDDERFAREELAELLA	fig|127906.87.peg.2164	VCC2164	VC0693	yehT	Two-component transcriptional response regulator BtsR		
1	CDS	2395580	2394588	-	atgggaccgctttggttggatgtg	MGPLWLDVAGYELSAEDREIL	fig|127906.87.peg.2165	VCC2165	VC0692	nagZ	beta-N-acetylglucosaminidase (EC 3.2.1.52)		
1	CDS	2395686	2396798	+	atggcagaacgcgaactctata	MAERELYIGVMSGTSLDGVDT	fig|127906.87.peg.2166	VCC2166	VC0691	anmK	Anhydro-N-acetylmuramic acid kinase (EC 2.7.1.170)		
1	CDS	2396816	2397739	+	atgaccaacgatgcacttatcgc	MTNDALIAALSHLVSEGRNPD	fig|127906.87.peg.2167	VCC2167	VC0690		N-acetylmuramic acid 6-phosphate etherase (EC 4.2.1.126)		
1	CDS	2399559	2397763	-	atgcacgaagagtctattgctctg	MHEESIALTIAAVGVVGLGCQV	fig|127906.87.peg.2168	VCC2168	VC0689		Sodium/hydrogen exchanger family protein		
1	CDS	2399901	2399578	-	atgggctgtattgtgctgcttgccg	MGCIVLLAACAASESQLVQEG	fig|127906.87.peg.2169	VCC2169	VC0688		hypothetical protein	DUF2799 domain-containing protein [Vibrio cholerae]	
1	CDS	2400172	2401656	+	atgttgtggtttctaacctgtgtcgc	MLWFLTCVAALVGGYFIYGAF	fig|127906.87.peg.2170	VCC2170	VC0687	yjiY	Carbon starvation protein A		
1	CDS	2402743	2401793	-	atgaaaatactgttagccaaccc	MKILLANPRGFCAGVDRAISIV	fig|127906.87.peg.2171	VCC2171	VC0685	ispH	4-hydroxy-3-methylbut-2-enyl diphosphate reductase (EC 1.17.7.4)		
1	CDS	2403224	2402790	-	gtgaaccctattgcctctaactctc	MNPIASNSAVTLHFTIKLKDGS	fig|127906.87.peg.2172	VCC2172	VC0684	fkpB	FKBP-type peptidyl-prolyl cis-trans isomerase SlpA (EC 5.2.1.8)		
1	CDS	2403902	2403387	-	atgtcgaattcgtctctggctctta	MSNSSLALKQSGLRWLWLAL	fig|127906.87.peg.2173	VCC2173	VC0683	lspA	Lipoprotein signal peptidase (EC 3.4.23.36)		
1	CDS	2406767	2403918	-	atgagtgagtataaagataccct	MSEYKDTLNLPETGFPMRGDL	fig|127906.87.peg.2174	VCC2174	VC0682	ileS	Isoleucyl-tRNA synthetase (EC 6.1.1.5)		
1	CDS	2407766	2406798	-	atggaattaatacgcggtattcat	MELIRGIHNIQSRHHGCVLTIG	fig|127906.87.peg.2175	VCC2175	VC0681	ribF	FMN adenylyltransferase (EC 2.7.7.2) / Riboflavin kinase (EC 2.7.1.26)		
1	CDS	2409397	2407835	-	gtgagtaaacgcttactcaagtc	MSKRLLKSGIIVSAMTLISRVL	fig|127906.87.peg.2176	VCC2176	VC0680	murJ/mviN	Peptidoglycan lipid II flippase MurJ		
1	CDS	2409655	2409915	+	ttggcaaataacaaatctgctaa	MANNKSAKKRAIQAEKRRQH	fig|127906.87.peg.2177	VCC2177	VC0679	rpsT	SSU ribosomal protein S20p		
1	CDS	2410286	2409984	-	atgaatctccaagagatggaaa	MNLQEMEKNSAKAVVLLKAM	fig|127906.87.peg.2178	VCC2178	VC0678	hlyU	Transcriptional activator HlyU		
1	CDS	2411399	2410509	-	atgtctcatcttaactataaccac	MSHLNYNHLYYFWMVCKQGS	fig|127906.87.peg.2179	VCC2179	VC0677	nhaR	Transcriptional activator NhaR		
1	CDS	2411794	2412942	+	atgataaaccaagcaacttccc	MINQATSPAAPISLTTKGLRWA	fig|127906.87.peg.2180	VCC2180	VC0676	ntpA	Sodium-dependent phosphate transporter		
1	CDS	2413886	2413035	-	gtgagacagtatttagatctttgtc	MRQYLDLCQRIVDQGVWVEN	fig|127906.87.peg.2181	VCC2181	VC0675	thyA	Thymidylate synthase (EC 2.1.1.45)		
1	CDS	2414711	2413896	-	atgcctcagggttatctgcagtttt	MPQGYLQFSNIDPVLFSIGPLA	fig|127906.87.peg.2182	VCC2182	VC0674	lgt	Prolipoprotein diacylglyceryl transferase		
1	CDS	2415534	2414740	-	gtgaatattgaactcttagctatac	MNIELLAILLLLGAVVGVLAGLL	fig|127906.87.peg.2183	VCC2183	VC0673		Uncharacterized UPF0721 integral membrane protein	sulfite exporter TauE/SafE family protein [Vibrio cholerae]	
1	CDS	2417790	2415544	-	atgctttctcagctaagggacata	MLSQLRDIVEQVSKVEDVYQA	fig|127906.87.peg.2184	VCC2184	VC0672	ptsP	FIG001592: Phosphocarrier protein kinase/phosphorylase, nitrogen regulation associated		
1	CDS	2418311	2417793	-	gtgatcgatggcgatggttaccg	MIDGDGYRLNVGIVICNNHGQ	fig|127906.87.peg.2185	VCC2185	VC0671	rppH	Adenosine (5')-pentaphospho-(5'')-adenosine pyrophosphohydrolase		
1	CDS	2418480	2418334	-	atgcgcggtatgtctcataaaatt	MRGMSHKIVDNLELAQNLSFF	fig|127906.87.peg.2186	VCC2186	VC0670		Adenosine (5')-pentaphospho-(5'')-adenosine pyrophosphohydrolase (EC 3.6.1.-)		
1	CDS	2418777	2418920	+	ttgatccatacctagcctactctc	MIHTLAYSQANKIKLWITSSIDL	fig|127906.87.peg.2187	VCC2187	Not annotated as CDS		hypothetical protein		
1	CDS	2418942	2419607	+	atgaaaccagcaccgacgact	MKPAPTTQQELLTRAQQIAGL	fig|127906.87.peg.2188	VCC2188	VC0668	mutH	DNA mismatch repair endonuclease MutH		
1	CDS	2419824	2420858	+	atgcaatataccaagctgcctca	MQYTKLPHSSLEISKICLGTMT	fig|127906.87.peg.2189	VCC2189	VC0667	yhdN	Tas protein, an NADP(H)-dependent aldo-keto reductase		
1	CDS	2421147	2420896	-	atgcgtcaatctatttacttacaat	MRQSIYLQLAVLLVRAELRREI	fig|127906.87.peg.2190	VCC2190	VC0666		hypothetical protein		
1	CDS	2421588	2421460	-	atgcatatagcgccttgtgtttattc	MHIAPCVYSLAEYFIHKRDKNS	fig|127906.87.peg.2191	VCC2191	No refSeq annotation		FIG01201445: hypothetical protein		
1	CDS	2423059	2421725	-	atgagcactcaattccgtatgga	MSTQFRMDSVPGSLVVVGGT	fig|127906.87.peg.2192	VCC2192	VC0665	vpsR	Transcriptional regulator VpsR		
1	CDS	2424865	2423333	-	atgactgatgctgttcaaaacga	MTDAVQNEINQEQIAQEENKL	fig|127906.87.peg.2193	VCC2193	VC0664	lysS	Lysyl-tRNA synthetase (class II) (EC 6.1.1.6)		
1	CDS	2425795	2424896	-	gtggataccatcgatcaactgga	MDTIDQLDRGVDDVEGLLELA	fig|127906.87.peg.2194	VCC2194	VC0663	prfB	Peptide chain release factor 2 @ programmed frameshift-containing		
1	CDS	2427477	2426164	-	gtgaagcagacgttaaaactag	MKQTLKLADILALGFMLFAFFL	fig|127906.87.peg.2195	VCC2195	VC0662	brnQ	Na(+)-dependent branched-chain amino acid transporter		
1	CDS	2427786	2428508	+	atgaaaaccaataaattaatcaa	MKTNKLINKSFRFKQFSIEEGT	fig|127906.87.peg.2196	VCC2196	VC0661	yfiC	tRNA(1)(Val) (adenine(37)-N(6))-methyltransferase (EC 2.1.1.223)		
1	CDS	2428596	2429867	+	gtgattaaaacctttgccgacctc	MIKTFADLELDPILLEAIEEMGF	fig|127906.87.peg.2197	VCC2197	VC0660	srmB	ATP-dependent RNA helicase SrmB		
1	CDS	2431578	2429989	-	atgtcaactacaccctatttcggc	MSTTPYFGEVSKRRTFAIISHP	fig|127906.87.peg.2198	VCC2198	VC0659	prfC	Peptide chain release factor 3		
1	CDS	2431788	2433845	+	atgaccaaatttcgcaagattca	MTKFRKIHSLAFKQAKSVFLVS	fig|127906.87.peg.2199	VCC2199	VC0658		C-di-GMP phosphodiesterase A-related protein		
1	CDS	2434283	2433834	-	atgacgactgtttttactgcaatg	MTTVFTAMQPTHLDAVWRIEC	fig|127906.87.peg.2200	VCC2200	VC0657	rimI	Ribosomal-protein-S18p-alanine acetyltransferase (EC 2.3.1.128)		
1	CDS	2434687	2434280	-	atgctttctcttcacactatttgtcc	MLSSSHYLSAMDIQQWELIHP	fig|127906.87.peg.2201	VCC2201	VC0656	holD	DNA polymerase III psi subunit (EC 2.7.7.7)		
1	CDS	2435301	2434798	-	atgcttattcgaactgaagcgcc	MLIRTEAPADILVVDQLLKNVF	fig|127906.87.peg.2202	VCC2202	VC0655		Uncharacterized N-acetyltransferase YhbS	Select seq ref|WP_000908540.1| N-acetyltransferase [Vibrio cholerae]	
1	CDS	2435812	2435285	-	gtgttaaacaagattcgcactca	MLNKIRTQLVQNAASILRSPIH	fig|127906.87.peg.2203	VCC2203	VC0654		Uncharacterized protein YhbT	SCP2 domain-containing protein [Vibrio cholerae]	ORF split into 2 ORFs in N16961, only the first ORF is refseq annotated. Annotated as 1 ORF in KEGG
									No refSeq annotation				ORF split into 2 ORFs in N16961, only the first ORF is refseq annotated. Annotated as 1 ORF in KEGG
1	CDS	2436045	2438099	+	atgcctgctcaaacctcatctcag	MPAQTSSQLKHWFAKITSHSF	fig|127906.87.peg.2204	VCC2204	VC0653		diguanylate cyclase/phosphodiesterase (GGDEF & EAL domains) with PAS/PAC sensor(s)		
1	CDS	2438207	2439220	+	atggaacttctctgtccggccgga	MELLCPAGNLPALKTAIDCGAI	fig|127906.87.peg.2205	VCC2205	VC0652		Uncharacterized protease YhbU	U32 family peptidase [Vibrio cholerae]	
1	CDS	2439232	2440110	+	atgaaatatgctttaggtccacta	MKYALGPLLYFWPKQDVESFY	fig|127906.87.peg.2206	VCC2206	VC0651		Uncharacterized peptidase U32 family member YhbV	U32 family peptidase [Vibrio cholerae]	
1	CDS	2441500	2440163	-	atgatgaatcgatcgatttaccga	MMNRSIYRQFVRYTIPTVAAM	fig|127906.87.peg.2207	VCC2207	VC0650	vmrA	Multi antimicrobial extrusion protein (Na(+)/drug antiporter), MATE family of MDR efflux pumps		
1	CDS	2441954	2441547	-	atgctcgatgttaatttagaaaaa	MLDVNLEKMERFAAKVWRKY	fig|127906.87.peg.2208	VCC2208	VC0649	slyA	Transcriptional regulator, MarR family		
1	CDS	2442964	2442053	-	gtgaaatggtttcgattcccgata	MKWFRFPIAVIGLVLLSGCATC	fig|127906.87.peg.2209	VCC2209	VC0648	nlpI	Lipoprotein NlpI		Limited query cover for certain strains
1	CDS	2445209	2443080	-	atgttcgaaaaaccagtagtaaa	MFEKPVVKTFQYGNHTVTLET	fig|127906.87.peg.2210	VCC2210	VC0647	pnp	Polyribonucleotide nucleotidyltransferase (EC 2.7.7.8)		
1	CDS	2445841	2445572	-	atgtctctgaatgcagaaactaa	MSLNAETKAAIVAEYARCEND	fig|127906.87.peg.2211	VCC2211	VC0646	rpsO	SSU ribosomal protein S15p (S13e)		
1	CDS	2446921	2445983	-	atggctcgtcgacgtaaaggtcg	MARRRKGRVIHGVILLDKPTG	fig|127906.87.peg.2212	VCC2212	VC0645	truB	tRNA pseudouridine(55) synthase (EC 5.4.99.25)		
1	CDS	2447319	2446921	-	atgccgaaagaatttagccgtac	MPKEFSRTQRVAQQLQKELAI	fig|127906.87.peg.2213	VCC2213	VC0644	rbfA	Ribosome-binding factor A		
1	CDS	2450117	2447421	-	atgacacaaattaccgttaaagc	MTQITVKALSEEIGTPVDRLLE	fig|127906.87.peg.2214	VCC2214	VC0643	infB	Translation initiation factor 2		
1	CDS	2451629	2450142	-	atgagtaaagaaattttagcggt	MSKEILAVVEAVSNEKAVPRE	fig|127906.87.peg.2215	VCC2215	VC0642	nusA	Transcription termination protein NusA		
1	CDS	2452103	2451648	-	atgactggtttagaaagacaact	MTGLERQLTEMLEAPVVAAGY	fig|127906.87.peg.2216	VCC2216	VC0641	rimP	Bacterial ribosome SSU maturation protein RimP		
1	rna	2452389	2452313	-	cgcggggtggagcagcttggtagctcgtcgggctcataacccgaaggtcgt		fig|127906.87.rna.62	VCCt047	VCt048		tRNA-Met-CAT		
1	rna	2452526	2452443	-	gccgagatggtggaaattggtagacacactagcatgaggtgctagcgcctta		fig|127906.87.rna.63	VCCt048	VCt047		tRNA-Leu-GAG		
1	CDS	2452869	2452534	-	atgtttacagttctacttgtgatttac	MFTVLLVIYLLAAVAIIGLVLIQG	fig|127906.87.peg.2217	VCC2217	VC0640	secA	Protein translocase membrane subunit SecG		
1	CDS	2453067	2452885	-	atgctcgaaaaaacgctgttgtg	MLEKTLLCEFFERSNRKVIFFR	fig|127906.87.peg.2218	VCC2218	Not annotated as CDS		hypothetical protein		
1	CDS	2454466	2453126	-	atgtctgataagagacgttattttg	MSDKRRYFGTDGVRGKVGQY	fig|127906.87.peg.2219	VCC2219	VC0639	glmM	Phosphoglucosamine mutase (EC 5.4.2.10)		
1	CDS	2455328	2454492	-	atgctgaaaatcagtcatgccga	MLKISHADKQLELHSPVVMGIL	fig|127906.87.peg.2220	VCC2220	VC0638	folP	Dihydropteroate synthase (EC 2.5.1.15)		
1	CDS	2457350	2455395	-	ttgagtgacatggcaaaaaattt	MSDMAKNLILWLVIAVVLMSVI	fig|127906.87.peg.2221	VCC2221	VC0637	ftsH	Cell division-associated, ATP-dependent zinc metalloprotease FtsH		
1	CDS	2458080	2457451	-	atgagtaaacagaaacactcgg	MSKQKHSASSTRWLKEHFDD	fig|127906.87.peg.2222	VCC2222	VC0636	rlmE/rrmJ	23S rRNA (uridine(2552)-2'-O)-methyltransferase (EC 2.1.1.166)		
1	CDS	2458200	2458496	+	atgaacctaagcaacaaacaa	MNLSNKQKQHLKGLAHNLKPV	fig|127906.87.peg.2223	VCC2223	VC0635	yhbY	RNA-binding protein YhbY		
1	CDS	2459046	2458573	-	atggaaaaagttccaatgacag	MEKVPMTARGEKLLREELDRL	fig|127906.87.peg.2224	VCC2224	VC0634	greA	Transcription elongation factor GreA		
1	CDS	2459601	2460626	+	atgaacaagactctgattgctctt	MNKTLIALAVSAAAVATGAYAI	fig|127906.87.peg.2225	VCC2225	VC0633	ompU	Outer membrane porin OmpU		
1	CDS	2460724	2462169	+	atgctttttcgcttcatacctgtttgg	MLFRFIPVWLLSISGFLIASPIY	fig|127906.87.peg.2226	VCC2226	VC0632	dacB	D-alanyl-D-alanine carboxypeptidase (EC 3.4.16.4)		
1	CDS	2463424	2462237	-	atggcgagtattgaagccgcttta	MASIEAALAEIKRGVEELIPEEI	fig|127906.87.peg.2227	VCC2227	VC0631		Tyrosyl-tRNA synthetase (EC 6.1.1.1)		
1	CDS	2463555	2464856	+	atgctttctcttttcaatcgtcttcct	MLSLFNRLPWLHRVLITAFSAI	fig|127906.87.peg.2228	VCC2228	VC0630		Membrane protein	Peptidoglycan DD-metalloendopeptidase family protein [Vibrio cholerae]	
1	CDS	2468057	2464938	-	atgttcactctgattgatgcggcg	MFTLIDAALSRTRTMLTLLVFIL	fig|127906.87.peg.2229	VCC2229	VC0629	mdtB	RND multidrug efflux transporter; Acriflavin resistance protein		
1	CDS	2469168	2468068	-	atgtttccgctcaaaacttcttcac	MFPLKTSSRQARDYFLHQPW	fig|127906.87.peg.2230	VCC2230	VC0628	czcB	Membrane-fusion protein	Multidrug efflux RND transporter periplasmic adaptor subunit VexE [Vibrio cholerae]	
1	CDS	2469607	2469266	-	gtgagcgaagttaatgttccatta	MSEVNVPLSFSDAAAKRVKAL	fig|127906.87.peg.2231	VCC2231	VC0627	erpA	Iron-sulfur cluster insertion protein ErpA		
1	CDS	2469876	2469727	-	atgatgaatcggaccaattttggg	MMNRTNFGAHCTPMLQWVKG	fig|127906.87.peg.2232	VCC2232	No refSeq annotation		FIG01202139: hypothetical protein		
1	CDS	2469891	2471189	+	atgaccaaatcagccgagttgta	MTKSAELYQKAQTTIPGGVNS	fig|127906.87.peg.2233	VCC2233	VC0626	hemL	Glutamate-1-semialdehyde 2,1-aminomutase (EC 5.4.3.8)		
1	CDS	2471406	2471251	-	atgacgccaattcagctgcttag	MTPIQLLSNPEVEMGCCDKNE	fig|127906.87.peg.2234	VCC2234	Not annotated as CDS		FIG01200382: hypothetical protein	glutamate-1-semialdehyde aminotransferase [Vibrio mimicus CAIM 1883]	Limited query cover for certain strains
1	CDS	2471443	2472528	+	gtgtcagaaaaaatcaaattagg	MSEKIKLASSHRVLVVALLASA	fig|127906.87.peg.2235	VCC2235	VC0624		UPF0118 membrane protein VC0624	AI-2E family transporter [Vibrio cholerae]	
1	CDS	2472601	2473623	+	atgcaaccttatactgccccgag	MQPYTAPSEIALRQLDYFADK	fig|127906.87.peg.2236	VCC2236	VC0623	rsmC	16S rRNA (guanine(1207)-N(2))-methyltransferase (EC 2.1.1.172)		
1	CDS	2473831	2477220	+	atgtttaggttctatcgaaaacaa	MFRFYRKQKFKRLQNTLMAAI	fig|127906.87.peg.2237	VCC2237	VC0622		Chitin catabolic cascade sensor histidine kinase ChiS		
1	CDS	2478131	2479801	+	atgcttgccaatattaaaaagac	MLANIKKTALAAAVIAAATSVS	fig|127906.87.peg.2238	VCC2238	VC0620		Chitobiose ABC transporter, substrate-binding protein		
1	CDS	2479971	2480957	+	atgggttactttttaagacgattgt	MGYFLRRLSFYFAALLVAATLI	fig|127906.87.peg.2239	VCC2239	VC0619		Chitobiose ABC transporter, permease protein 1		
1	CDS	2480960	2481985	+	atgaaagcactttggaaactctta	MKALWKLLRGNRMAMIGVTIL	fig|127906.87.peg.2240	VCC2240	VC0618		Chitobiose ABC transporter, permease protein 2		
1	CDS	2481988	2482971	+	atgactacgccattaatctcaatc	MTTPLISIRNLCVDYITDAGDVI	fig|127906.87.peg.2241	VCC2241	VC0617		Chitobiose ABC transporter, ATP-binding protein 1		
1	CDS	2483014	2484009	+	atgagcaaaccacatggcgaa	MSKPHGELLVEGKNLVKDFAI	fig|127906.87.peg.2242	VCC2242	VC0616		Chitobiose ABC transporter, ATP-binding protein 2		
1	CDS	2484066	2485790	+	atgctgttactgaccaaccacat	MLLLTNHIGYETQGPKQAVLL	fig|127906.87.peg.2243	VCC2243	VC0615		Glucosaminyl-1,4-glucosamine-6-phosphate hydrolase		
1	CDS	2485787	2486671	+	atgaactactacgtaggcattga	MNYYVVGIDGGGTSCRARIRNC	fig|127906.87.peg.2244	VCC2244	VC0614		Glucosamine kinase GpsK (EC 2.7.1.8)		
1	CDS	2486668	2488581	+	atgagttatcgaattgaatttgcg	MSYRIEFAVLSEQKPDCRFGL	fig|127906.87.peg.2245	VCC2245	VC0613		beta-N-acetylglucosaminidase (EC 3.2.1.52)		
1	CDS	2488700	2491105	+	atgaaatacggctatttcgatt	MKYGYFDNDNREYVITRPDVF	fig|127906.87.peg.2246	VCC2246	VC0612		N,N'-diacetylchitobiose phosphorylase (EC 2.4.1.280)		
1	CDS	2491228	2492640	+	atgattaaatttggtactggcggc	MIKFGTGGWRAFIGEEFTKDN	fig|127906.87.peg.2247	VCC2247	VC0611		N-acetylglucosamine phosphomutase (EC 5.4.2.3)		
1	CDS	2493802	2492771	-	atgagctgcgcactgctgattga	MSCALLIENLTCQYDAQTVLKS	fig|127906.87.peg.2248	VCC2248	VC0610		Ferric iron ABC transporter, ATP-binding protein		
1	CDS	2495427	2493802	-	atgaaagaaaaatattttgcctg	MKEKYFAWKASSLVLTTLLVLI	fig|127906.87.peg.2249	VCC2249	VC0609		Ferric iron ABC transporter, permease protein		
1	CDS	2496524	2495511	-	atgaaaaaactgctcagcctgtc	MKKLLSLSAITLSVLAPSAFAA	fig|127906.87.peg.2250	VCC2250	VC0608		Ferric iron ABC transporter, iron-binding protein		
1	CDS	2497895	2496666	-	atggaactatcaatcacagttac	MELSITVTELRYALDTFFFLISG	fig|127906.87.peg.2251	VCC2251	VC0607	amtB	Ammonium transporter		ORF split into 2 ORFs in N16961, none refseq annotated. Annotated as 1 pseudogene ORF named VC0607 in KEGG
									VC0607				ORF split into 2 ORFs in N16961, none refseq annotated. Annotated as 1 pseudogene ORF named VC0607 in KEGG
1	CDS	2498255	2497917	-	atgaaactgatcaacgcaatca	MKLINAIIKPFKLDDVREALADV	fig|127906.87.peg.2252	VCC2252	VC0606		Nitrogen regulatory protein P-II, GlnK		
1	CDS	2498813	2498433	-	atggaatttgaatttattaaaaata	MEFEFIKNTLLGEYAVRCNME	fig|127906.87.peg.2253	VCC2253	VC0605		hypothetical protein	YacL family protein [Vibrio cholerae]	
1	CDS	2501565	2498968	-	gtgcttgaagcctaccgtaaaca	MLEAYRKHVAERAAEGVVPKI	fig|127906.87.peg.2254	VCC2254	VC0604	acnB	Aconitate hydratase 2 (EC 4.2.1.3) @ 2-methylisocitrate dehydratase (EC 4.2.1.99)		
1	CDS	2504121	2501830	-	atgaaatgggcggtatcattcag	MKWAVSFSVVVGIVFAPLALA	fig|127906.87.peg.2255	VCC2255	VC0603	rssA	bifunctional outer membrane translocase / extracellular lipase, PlpD		
1	CDS	2506694	2504361	-	atgacgagaaaaagtagtaaca	MTRKSSNRSRGRKARSGKSA	fig|127906.87.peg.2256	VCC2256	VC0602	mrcB	Multimodular transpeptidase-transglycosylase (EC 2.4.1.129) (EC 3.4.-.-)		
1	CDS	2509153	2506691	-	ttgtcaccactgcccattattgag	MSPLPIIEVMPKLLSGITNSPQI	fig|127906.87.peg.2257	VCC2257	VC0601	hrpB	ATP-dependent helicase HrpB		
1	CDS	2510577	2509252	-	atgtttaaggttgtattgctggctg	MFKVVLLAVLALSIWSTRIPDM	fig|127906.87.peg.2258	VCC2258	VC0600		esterase/lipase/thioesterase family active site:lipase (Class 3)		
1	CDS	2510815	2511585	+	atgccttcgccattggctatgaat	MPSPLAMNKKRNMHFSPPLQ	fig|127906.87.peg.2259	VCC2259	VC0597	sfsA	Sugar fermentation stimulation protein SfsA		
1	CDS	2511710	2512156	+	atgacagagtctaaaaagaaa	MTESKKKTLGILAIAGVEPYQE	fig|127906.87.peg.2260	VCC2260	VC0596	dksA	RNA polymerase-binding transcription factor DksA		
1	CDS	2512230	2513120	+	atgaactatattggtcgcttcgcc	MNYIGRFAPSPSGPLHFGSLIA	fig|127906.87.peg.2261	VCC2261	VC0595	gluQ	Glutamyl-Q tRNA(Asp) synthetase		
1	CDS	2513211	2514584	+	atgaataataacgattttgaaccc	MNNNDFEPSNTRTYPDLALTI	fig|127906.87.peg.2262	VCC2262	VC0594	pcnB	Poly(A) polymerase (EC 2.7.7.19)		
1	CDS	2514581	2515084	+	atgatgcttgcctacattgcgattg	MMLAYIAIGSNLGDPVAQAQL	fig|127906.87.peg.2263	VCC2263	VC0593		2-amino-4-hydroxy-6-hydroxymethyldihydropteridine pyrophosphokinase (EC 2.7.6.3)		
1	CDS	2515087	2515881	+	atgaaaaaaattaccataaacg	MKKITINDLMKWKQEGRKFAT	fig|127906.87.peg.2264	VCC2264	VC0592	panB	3-methyl-2-oxobutanoate hydroxymethyltransferase (EC 2.1.2.11)		
1	CDS	2515892	2516773	+	atgcaagtttttgctgacatcgcc	MQVFADIAVLREQIKQIKREGR	fig|127906.87.peg.2265	VCC2265	VC0591	panC	Pantoate--beta-alanine ligase (EC 6.3.2.1)		
1	CDS	2517607	2516837	-	atgtatcaaatttattggacggca	MYQIYWTAFCSLLGKEINRFTF	fig|127906.87.peg.2266	VCC2266	VC0590	yadH	Uncharacterized efflux ABC transporter, permease protein YadH		
1	CDS	2518530	2517613	-	atgtacgcactggaaatagaac	MYALEIEQLRKTYAGGFEALK	fig|127906.87.peg.2267	VCC2267	VC0589		Uncharacterized efflux ABC transporter, ATP-binding protein YadG		
1	repeat	2518595	2518792	+	tataccaaaactacttggaattgcagctaggcggcaagtaagtgacaaattt		fig|127906.87.repeat.46				repeat region		
1	CDS	2520560	2518914	-	gtgtttcaggctagatttgcggatc	MFQARFADLNLRCDLFGGVTT	fig|127906.87.peg.2268	VCC2268	VC0587		Na(+)-dependent bicarbonate transporter BicA		
1	CDS	2520708	2521376	+	atgccagaaattaaacaacttttt	MPEIKQLFENNSKWSESIKAE	fig|127906.87.peg.2269	VCC2269	VC0586	can	Carbonic anhydrase, beta class (EC 4.2.1.1)		
1	CDS	2521984	2521451	-	atgaaacacacagttgaagtca	MKHTVEVMISEQEVAQRIREL	fig|127906.87.peg.2270	VCC2270	VC0585	hpt	Hypoxanthine-guanine phosphoribosyltransferase (EC 2.4.2.8)		
1	CDS	2522280	2522891	+	atggacgcatcaatcgaaaaac	MDASIEKRPRTRLSPQKRKLQ	fig|127906.87.peg.2271	VCC2271	VC0583	luxR	Quorum-sensing regulator of virulence HapR		ORF split into 2 ORFs in N16961. N16961 annotated as 1 ORF pseudogene named VC0583 in KEGG
									VC0583				ORF split into 2 ORFs in N16961. N16961 annotated as 1 ORF pseudogene named VC0583 in KEGG
1	CDS	2523971	2523105	-	atgacagataataaaaccgtgc	MTDNKTVPNGAPILYIVPTPIG	fig|127906.87.peg.2272	VCC2272	VC0582	rsmI	16S rRNA (cytidine(1402)-2'-O)-methyltransferase (EC 2.1.1.198)		
1	CDS	2524035	2525846	+	atggctatgaaccaccatcagcc	MAMNHHQRRSVPRLLTPIALS	fig|127906.87.peg.2273	VCC2273	VC0581	lpoA	Penicillin-binding protein activator LpoA		
1	CDS	2525878	2526201	+	atggcggcggactatcttcgccg	MAADYLRRQGLTLVTQNVNYI	fig|127906.87.peg.2274	VCC2274	VC0580		UPF0102 protein YraN	Predicted endonuclease-like protein archaeal Holliday junction resolvase [Vibrio cholerae LMA3984-4]	
1	CDS	2526205	2526795	+	atgctcgatagcattaaagacac	MLDSIKDSFTESIQIQIAAAEAL	fig|127906.87.peg.2275	VCC2275	VC0579	dlaA	DnaA initiator-associating protein DiaA		
1	CDS	2526798	2527367	+	atgaaaagcattagacttcttatc	MKSIRLLIVSGLLATLTGCAGL	fig|127906.87.peg.2276	VCC2276	VC0578		Uncharacterized protein YraP	BON domain-containing protein [Vibrio cholerae]	
1	CDS	2527899	2527435	-	atggatattggtcacatgacacc	MDIGHMTPRRPYLLRAFYDWI	fig|127906.87.peg.2277	VCC2277	VC0577	sspB	Stringent starvation protein B		
1	CDS	2528546	2527911	-	atggctgtagctgccaacaaac	MAVAANKRSVMTLFSSASDM	fig|127906.87.peg.2278	VCC2278	VC0576	sspA	Stringent starvation protein A		
1	CDS	2529383	2528646	-	atgaaaaaatggattgtagttttg	MKKWIVVLFAMLPSLAMAAGA	fig|127906.87.peg.2279	VCC2279	VC0575	fbcH/petC	Ubiquinol-cytochrome C reductase, cytochrome C1 subunit		
1	CDS	2530645	2529380	-	atgcaagctctacttgattgggta	MQALLDWVEKRLPAMNAYKK	fig|127906.87.peg.2280	VCC2280	VC0574	petB	Ubiquinol-cytochrome C reductase, cytochrome B subunit (EC 1.10.2.2)		
1	CDS	2531238	2530645	-	atgagtaatgcgcctctaaatca	MSNAPLNQGRRRFLTATTAV	fig|127906.87.peg.2281	VCC2281	VC0573	petA	Ubiquinol-cytochrome C reductase iron-sulfur subunit (EC 1.10.2.2)		
1	CDS	2531958	2531566	-	atggcagagaatcaatactacg	MAENQYYGTGRRKSSAARVF	fig|127906.87.peg.2282	VCC2282	VC0571	rpsI	SSU ribosomal protein S9p (S16e)		
1	CDS	2532402	2531974	-	atgaaaactttcgttgctaaacca	MKTFVAKPETVKRDWYVVDA	fig|127906.87.peg.2283	VCC2283	VC0570	rplM	LSU ribosomal protein L13p (L13Ae)		
1	CDS	2533729	2532626	-	atgactccgaagcagcgatacg	MTPKQRYEKDLQRTDFYSDE	fig|127906.87.peg.2284	VCC2284	VC0568		Cell division protein ZapE		
1	CDS	2533883	2534320	+	atgccttggatctatgccattgctg	MPWIYAIAGLLVGIAVGMLIAR	fig|127906.87.peg.2285	VCC2285	VC0567	yhcB	Putative cytochrome d ubiquinol oxidase subunit III (EC 1.10.3.-) (Cytochrome bd-I oxidase subunit III)		
1	CDS	2534452	2535819	+	atgaaaaaacctttacttgttttaa	MKKPLLVLTALSLSLSAILSPLF	fig|127906.87.peg.2286	VCC2286	VC0566	degQ/htrA	Outer membrane stress sensor protease DegQ, serine protease		
1	CDS	2535996	2537054	+	atgctgaaattttgggttcgctcaa	MLKFWVRSISLGLLAAIAIIMVT	fig|127906.87.peg.2287	VCC2287	VC0565	degS	Outer membrane stress sensor protease DegS		

1	CDS	2537528	2537175	-	atgagtaacatcatcaaggctct	MSNIIKALEQEMKQDLPQFA	fig|127906.87.peg.2288	VCC2288	VC0564	rplS	LSU ribosomal protein L19p		
1	CDS	2538313	2537570	-	atgtgggttggcatcgttagccta	MWVGIVSLFPEMFRSVTDFG\	fig|127906.87.peg.2289	VCC2289	VC0563	trmD	tRNA (guanine(37)-N(1))-methyltransferase (EC 2.1.1.228)		
1	CDS	2538895	2538341	-	atgtcgatgaaaggtaaagaga	MSMKGKETMSKQNEKLVVGK	fig|127906.87.peg.2290	VCC2290	VC0562	rimM	16S rRNA processing protein RimM		
1	CDS	2539171	2538923	-	atggtaaccattcgtttggcacgt	MVTIRLARHGAKKRPFYQIVV	fig|127906.87.peg.2291	VCC2291	VC0561	rpsP	SSU ribosomal protein S16p		
1	CDS	2540792	2539407	-	atgtttgagaatttaaccgatcga	MFENLTDRLSKTLKNISGKGR	fig|127906.87.peg.2292	VCC2292	VC0560	ffh	Signal recognition particle protein Ffh		
1	CDS	2541005	2541799	+	atggacaacttaatcgcgattgc	MDNLIAIAAAMLYLLSIATIVPG	fig|127906.87.peg.2293	VCC2293	VC0559	ypjD	Inner membrane protein YpjD		
1	CDS	2541882	2543162	+	ttggacgacatatcaacgggtat	MDDISTGILFALLACLIIISAYFS	fig|127906.87.peg.2294	VCC2294	VC0558		Putative membrane protein YfjD	HlyC/CorC family transporter [Vibrio cholerae]	
1	CDS	2543750	2543232	-	atgccattattagacagttttaccg	MPLLDSFTVDHTRMNAPAVR\	fig|127906.87.peg.2295	VCC2295	VC0557	luxS	S-ribosylhomocysteine lyase (EC 4.4.1.21) @ Autoinducer-2 production protein LuxS		
1	CDS	2545373	2543799	-	ttgagtaaatttgccgagcgacta	MSKFAERLQTVANKPEVFQKI	fig|127906.87.peg.2296	VCC2296	VC0556	gshA	Glutamate--cysteine ligase (EC 6.3.2.2)		
1	CDS	2548340	2545485	-	atgagaaaagtcgcgttgtttgg	MRKVALFGFSLLLLAGCSSSD	fig|127906.87.peg.2297	VCC2297	VC0554		FIG015547: peptidase, M16 family / FIG015287: Zinc protease		
1	CDS	2548811	2548350	-	atgctagattggtggaatgactct	MLDWWNDSSALLTSLFAESPI	fig|127906.87.peg.2298	VCC2298	VC0553	yqaA	putative membrane protein	DedA family protein [Vibrio cholerae]	
1	CDS	2549755	2548805	-	atggaaaacaaacgaatcgtca	MENKRIVITQFGTPDVLAMQS	fig|127906.87.peg.2299	VCC2299	VC0552	qorA	NADPH:quinone reductase		
1	CDS	2551008	2549878	-	atggacggattaattaccctatgg	MDGLITLWQETGIANFELGQII	fig|127906.87.peg.2300	VCC2300	VC0551	gcdB	Oxaloacetate decarboxylase Na(+) pump, beta chain (EC 4.1.1.3)		
1	CDS	2552805	2551018	-	atgtctaaaccattagccatcac	MSKPLAITDVVLRDAHQSLFAT	fig|127906.87.peg.2301	VCC2301	VC0550		Oxaloacetate decarboxylase Na(+) pump, alpha chain (EC 4.1.1.3)		
1	CDS	2553100	2552840	-	atgacacatattggaagtctctta	MTHIGSLLLDAATLMVTGMAV	fig|127906.87.peg.2302	VCC2302	VC0549		Oxaloacetate decarboxylase Na(+) pump, gamma chain (EC 4.1.1.3)		
1	rna	2553543	2553467	-	gcgctcgtagctcagctggatagagtacctggctacgaaccaggcggtcag		fig|127906.87.rna.64	VCCt049	VCt046		tRNA-Arg-ACG		
1	rna	2553691	2553615	-	gcgtccgtagctcagctggatagagtacctggctacgaaccaggcggtcag		fig|127906.87.rna.65	VCCt050	VCt045		tRNA-Arg-ACG		
1	rna	2553824	2553748	-	gcgctcgtagctcagctggatagagtacctggctacgaaccaggcggtcag		fig|127906.87.rna.66	VCCt051	VCt044		tRNA-Arg-ACG		
1	rna	2553973	2553897	-	gcgtccgtagctcagctggatagagtacctggctacgaaccaggcggtcag		fig|127906.87.rna.67	VCCt052	VCt043		tRNA-Arg-ACG		
1	rna	2554105	2554029	-	gcgctcgtagctcagctggatagagtacctggctacgaaccaggcggtcag		fig|127906.87.rna.68	VCCt053	VCt042		tRNA-Arg-ACG		
1	rna	2554264	2554172	-	ggtgaggtggccgagaggctgaaggcgctcccctgctaagggagtatacg		fig|127906.87.rna.69	VCCt054	VCt041		tRNA-Ser-GCT		
1	rna	2554363	2554287	-	gcgtccgtagctcagctggatagagtacctggctacgaaccaggcggtcag		fig|127906.87.rna.70	VCCt055	VCt040		tRNA-Arg-ACG		
1	rna	2554489	2554397	-	ggtgaggtggccgagaggctgaaggcgctcccctgctaagggagtatacg		fig|127906.87.rna.71	VCCt056	VCt039		tRNA-Ser-GCT		
1	CDS	2554868	2554671	-	atgcttattttgactcgccgtgttgg	MLILTRRVGETLMIGDEVTVTV	fig|127906.87.peg.2303	VCC2303	VC0548	csrA	Carbon storage regulator		
1	CDS	2556147	2554960	-	gtgaaaaagccccttatcgtgca	MKKPLIVQKFGGTSVGSIERM	fig|127906.87.peg.2304	VCC2304	VC0547		Aspartokinase (EC 2.7.2.4)		
1	CDS	2556319	2556200	-	ttgttgattgagcgtaattttatctg	MLIERNFIWSTKWFNWRKYRL	fig|127906.87.peg.2305	VCC2305	VC0546		FIG01202695: hypothetical protein		
1	CDS	2558933	2556351	-	atgtttatgagcacggatgaggt	MFMSTDEVRRAFLSFFESKGH	fig|127906.87.peg.2306	VCC2306	VC0545	alaS	Alanyl-tRNA synthetase (EC 6.1.1.7)		
1	CDS	2559564	2559106	-	atgagtttcgacttagccacttgc	MSFDLATCRQSALQLLSRRDH	fig|127906.87.peg.2307	VCC2307	VC0544	recX	Regulatory protein RecX		
1	CDS	2560761	2559697	-	atggacgagaataaacagaag	MDENKQKALAAALGQIEKQFG	fig|127906.87.peg.2308	VCC2308	VC0543	recA	RecA protein		
1	CDS	2561401	2560895	-	atgaaaacactgcattccttgag	MKTLHSLSEQLGGLLANQQQI	fig|127906.87.peg.2309	VCC2309	VC0542	pncC	Nicotinamide-nucleotide amidase (EC 3.5.1.42)		
1	CDS	2562698	2561568	-	atgagcattcgtctagataatatc	MSIRLDNISKHFGQFQALAPLS	fig|127906.87.peg.2310	VCC2310	VC0541	cysA	Sulfate and thiosulfate import ATP-binding protein CysA (EC 3.6.3.25)		
1	CDS	2563558	2562695	-	atggcacactctcagcctttacgt	MAHSQPLRVGEKPWVKWSLI	fig|127906.87.peg.2311	VCC2311	VC0540		Sulfate transport system permease protein CysW		
1	CDS	2564420	2563569	-	atgggtcaaacgcttgcacactc	MGQTLAHSRPRQQRVLPGFA	fig|127906.87.peg.2312	VCC2312	VC0539	cysT	Sulfate transport system permease protein CysT		
1	CDS	2565498	2564497	-	atgaaccgaattaaatcgacatt	MNRIKSTLTALLLAGSFQASAA	fig|127906.87.peg.2313	VCC2313	VC0538	cysP	Sulfate and thiosulfate binding protein CysP		
1	repeat	2565718	2565849	+	tttatacccaaacaacttggagttgcaggtaggcggcaagtgagtgagtccc		fig|127906.87.repeat.47				repeat region		
1	CDS	2565882	2566769	+	gtgtctgatttccccactcttgaag	MSDFPTLEDYVGQTPLVRLQF	fig|127906.87.peg.2314	VCC2314	VC0537	cysM	Cysteine synthase B (EC 2.5.1.47)		
1	CDS	2567040	2569625	+	atgaaatcgaacgcctcaccga	MKSNASPSESLSHHTPMMQC	fig|127906.87.peg.2315	VCC2315	VC0535	mutS	DNA mismatch repair protein MutS		
1	CDS	2570725	2569718	-	atgagtgtcagcaataccgtaac	MSVSNTVTKVEEFDFEDEALE	fig|127906.87.peg.2316	VCC2316	VC0534	rpoS	RNA polymerase sigma factor RpoS		
1	CDS	2571734	2570799	-	atgacgctttactctttccgactgg	MTLYSFRLGLLLFCSLLFGCTA	fig|127906.87.peg.2317	VCC2317	VC0533	nlpD	Murein hydrolase activator NlpD		
1	CDS	2572360	2571734	-	atggctaacccaaaagccgaca	MANPKADRLIQFLTEQGITSP(	fig|127906.87.peg.2318	VCC2318	VC0532	pcm	Protein-L-isoaspartate O-methyltransferase (EC 2.1.1.77)		
1	CDS	2573105	2572353	-	atgaagattctactcagtaacga	MKILLSNDDGVYAQGIHALAD	fig|127906.87.peg.2319	VCC2319	VC0531	surE	5'-nucleotidase SurE (EC 3.1.3.5) @ Exopolyphosphatase (EC 3.6.1.11)		
1	CDS	2574192	2573107	-	atgactgatattctctctcctctgg	MTDILSPLAYLCGKPTAKAKLK	fig|127906.87.peg.2320	VCC2320	VC0530	truD	tRNA pseudouridine(13) synthase (EC 5.4.99.27)		
1	CDS	2574698	2574222	-	atgatcagaattggccacggctt	MIRIGHGFDVHRFGGEGPIIIG	fig|127906.87.peg.2321	VCC2321	VC0529	ispF	2-C-methyl-D-erythritol 2,4-cyclodiphosphate synthase (EC 4.6.1.12)		
1	CDS	2575393	2574695	-	atgaacaacatgacagcgattg	MNNMTAIVPAAGVGSRMQAD	fig|127906.87.peg.2322	VCC2322	VC0528	ispD	2-C-methyl-D-erythritol 4-phosphate cytidylyltransferase (EC 2.7.7.60)		
1	CDS	2575674	2575390	-	atgcgagtatttgctttgaccctaa	MRVFALTLSLLLVWLLYTLMW	fig|127906.87.peg.2323	VCC2323	VC0527	ftsB	Cell division protein DivIC (FtsB), stabilizes FtsL against RasP cleavage		
1	CDS	2576592	2575789	-	atgagttattttgagtcgttcattct	MSYFESFILALIQGFTEFLPISS	fig|127906.87.peg.2324	VCC2324	VC0526	uppP	Undecaprenyl-diphosphatase (EC 3.6.1.27)		
1	CDS	2577095	2576589	-	atgatcaccgcttatattggtgtg	MITAYIGVGSNQDRHQSIEAG	fig|127906.87.peg.2325	VCC2325	VC0525		2-amino-4-hydroxy-6-hydroxymethyldihydropteridine pyrophosphokinase (EC 2.7.6.3)		
1	CDS	2577445	2577092	-	atggataaagtgtttattgaacag	MDKVFIEQLEVITTIGVYDWEC	fig|127906.87.peg.2326	VCC2326	VC0524	folB	Dihydroneopterin aldolase (EC 4.1.2.25)		
1	CDS	2577634	2578260	+	atgactccattggcgctcagcatc	MTPLALSMIIFAYLLGSISSAVL	fig|127906.87.peg.2327	VCC2327	VC0523	plsY	Acyl-phosphate:glycerol-3-phosphate O-acyltransferase PlsY (EC 2.3.1.n3)		
1	CDS	2579121	2578309	-	atgaattcgatgctgattgatggc	MNSMLIDGQKMAYLDVGEGE	fig|127906.87.peg.2328	VCC2328	VC0522		Beta-ketoadipate enol-lactone hydrolase (EC 3.1.1.24)		
1	CDS	2580294	2579275	-	atgcgcattattggtattgaaacc	MRIIGIETSCDETGIAIYDDEKG	fig|127906.87.peg.2329	VCC2329	VC0521	gcp	N(6)-L-threonylcarbamoyladenine synthase (EC 2.3.1.234)		ORF split into 2 ORFs in N16961. Latter ORF lacks refseq annotation. Annotated as 1 ORF named VC0521 in KEGG
									VC0521				ORF split into 2 ORFs in N16961. Latter ORF lacks refseq annotation. Annotated as 1 ORF named VC0521 in KEGG
1	CDS	2580337	2580507	+	atgttacctcgccttggcgcgaca	MLPRLGATNKPCPECKRQSAI	fig|127906.87.peg.2330	VCC2330	No refSeq annotation		FIG01201132: hypothetical protein		
1	CDS	2580535	2580750	+	atgccagtagttaaagtacgtga	MPVVKVRENEPFDVALRRFKF	fig|127906.87.peg.2331	VCC2331	VC0520	rpsU	SSU ribosomal protein S21p		
1	CDS	2580780	2581223	+	atggctctgattgagaatctcaaa	MALIENLKEEQKLAMKAKDKP	fig|127906.87.peg.2332	VCC2332	VC0519		Transamidase GatB domain protein		
1	CDS	2581313	2583076	+	atggcaggacacatcccccgca	MAGHIPRSFIDDLLARLDIVDV	fig|127906.87.peg.2333	VCC2333	VC0518	dnaG	DNA primase DnaG		ORF split into 2 ORFs in N16961. Latter ORF lacks refseq annotation. Annotated as 1 ORF named VC0518 in KEGG
									VC0518				ORF split into 2 ORFs in N16961. Latter ORF lacks refseq annotation. Annotated as 1 ORF named VC0518 in KEGG
1	CDS	2583175	2585052	+	atggatcaaaatccgcagtcac	MDQNPQSQLKQLVLRGKEQG	fig|127906.87.peg.2334	VCC2334	VC0517	rpoD	RNA polymerase sigma factor RpoD		
1	rna	2585197	2585272	+	ggccccttagctcagtggttagagcaggcgactcataatcgcttggtccccag		fig|127906.87.rna.72	VCCt057	VCt038		tRNA-Met-CAT		
1	CDS	2585438	2586679	+	atggcaaaaagagtggtcccttt	MAKRVVPLTNTQVKQAKESDI	fig|127906.87.peg.2335	VCC2335	VC0516		Phage integrase		
									VC0515				Region with high degree of sequence diveristy between C6706 and N16961
									VC0514				
									VC0513				
									VC0512				
1	CDS	2587584	2586715	-	atgatcaagaaactcaagcaga	MIKKLKQSHSIKTLCEVFNVHR	fig|127906.87.peg.2336	VCC2337	Not present		Transposase InsO for insertion sequence element IS911		
1	CDS	2587892	2587584	-	atgacaaaacgtacaagacgg	MTKRTRRLFSAEFKLEAAQLV	fig|127906.87.peg.2337	VCC2337	Not present		Transposase InsN for insertion sequence element IS911		
1	CDS	2590154	2588199	-	atgccggtgtttgaggcggtgatt	MPVFEAVINSIHSIEEKGNIDQ	fig|127906.87.peg.2338	VCC2338	Not present		hypothetical protein	ATP-binding protein [Vibrio sp. E4404]	
1	CDS	2592986	2590521	-	atggaacaaggcaaaggtttaa	MEQGKGLTFFGLFDAIDIIQIPI	fig|127906.87.peg.2339	VCC2339	Not present		hypothetical protein	DUF262 domain-containing protein [Vibrio cholerae]	
1	CDS	2594797	2592986	-	atgtcagttgagaataacctagta	MSVENNLVLKPISELLDESFFI	fig|127906.87.peg.2340	VCC2340	Not present		hypothetical protein	DUF262 domain-containing protein [Vibrio cholerae]	
1	CDS	2595109	2595582	+	atgaatcccaaaacttccgatta	MNPKTSDYQKQQRYQENEILE	fig|127906.87.peg.2341	VCC2341	VC0510		UPF0758 family protein	MULTISPECIES: DNA repair protein RadC [Vibrio]	
1	CDS	2595579	2596016	+	atgattgagcaacattacggtga	MIEQHYGELSIDDALHTSLESL	fig|127906.87.peg.2342	VCC2342	VC0509		hypothetical protein	DUF2787 domain-containing protein [Vibrio cholerae]	
1	CDS	2596066	2596509	+	atgtccaaattaaccttcacaac	MSKLTFTTSTLPVSKKLHKLLS	fig|127906.87.peg.2343	VCC2343	VC0508		hypothetical protein	DUF2787 domain-containing protein [Vibrio cholerae]	
									VC0507				
1	CDS	2596828	2597562	+	atgagcaataaaaccagcattg	MSNKTSIEGLSALLHTLMLIPQ	fig|127906.87.peg.2344	VCC2344	VC0506		Transcriptional factor MdcH		
1	CDS	2597593	2597961	+	atgacattaactaacactctactc	MTLTNTLLRYAAMSILFIGLTA(	fig|127906.87.peg.2345	VCC2345	VC0505		hypothetical protein		
1	CDS	2598009	2598236	+	atgaaaaagtcatctctacttatc	MKKSSLLITTALVFSTSSAFAG	fig|127906.87.peg.2346	VCC2346	VC0504		hypothetical protein		
1	CDS	2599694	2598414	-	atgggtcaatttagatttcttgcttt	MGQFRFLALIVAVLCFSVALFL	fig|127906.87.peg.2347	VCC2347	VC0503		Cell wall endopeptidase, family M23/M37		
1	CDS	2599992	2599813	-	gtgtctatcgttttcgccgttgagtt	MSIVFAVEFSNTVLTSSGLDC(	fig|127906.87.peg.2348	VCC2348	Not present		hypothetical protein		
1	CDS	2600390	2600914	+	atgtctaaaaatcaaggatttaca	MSKNQGFTLIELIIAIVILGVIAV	fig|127906.87.peg.2349	VCC2349	VC0502		Fimbrial protein pilin	type II secretion system protein [Vibrio cholerae]	
1	CDS	2601303	2601752	+	atgccgaaaccacgttacaaaa	MPKPRYKTNNWKQYNQALINI	fig|127906.87.peg.2350	VCC2350	No refSeq annotation		Mobile element protein	IS5 family transposase [Vibrio cholerae]	
1	CDS	2601725	2602213	+	gtgtggcgaaaattacatctcgc	MWRKLHLAVDTSTHEIIAAELS	fig|127906.87.peg.2351	VCC2351	Not present		Mobile element protein	IS5 family transposase [Vibrio cholerae]	
1	CDS	2602716	2602868	+	atgaccacgcgaaccagacgta	MTTRTRRTFSAEFRLEAAQLV	fig|127906.87.peg.2352	VCC2352	No refSeq annotation		Transposase InsN for insertion sequence element IS911		
1	CDS	2602895	2603041	+	gtgactccccaagcctcacctat	MTPQASPMTPEQIEIRELKKKL	fig|127906.87.peg.2353	VCC2353	No refSeq annotation		Transposase InsN for insertion sequence element IS911		
1	CDS	2603188	2603676	+	atggcaagcaatggctctgctga	MASNGSADARSIADMVTAQG	fig|127906.87.peg.2354	VCC2354	No refSeq annotation		Transposase InsO for insertion sequence element IS911		
1	CDS	2603743	2603907	+	gtgtcgagttatcggagttttagcc	MSSYRSFSEAREHITRYITGYY	fig|127906.87.peg.2355	VCC2355	No refSeq annotation		Transposase InsO for insertion sequence element IS911		
									VC501		Pseudogene		
									VC500		Pseudogene		
									VC499		Pseudogene		
1	CDS	2604428	2603988	-	atgagttattcaatttacgtagacc	MSYSIYVDGAAPNNQYGCTR	fig|127906.87.peg.2356	VCC2356	VC0498		Ribonuclease HI, Vibrio paralog	Ribonuclease H [Vibrio cholerae]	
1	CDS	2604710	2604510	-	atgagatttctacgacttaaagac	MRFLRLKDVMSLTGLGRSTIYI	fig|127906.87.peg.2357	VCC2357	VC0497		Transcriptional regulator	MULTISPECIES: AlpA family transcriptional regulator [Vibrio]	
1	CDS	2605373	2604774	-	atgtcgaacacaacccaccttcc	MSNTTHLPTITRARTFKDYPLF	fig|127906.87.peg.2358	VCC2358	VC0496		hypothetical protein	Inovirus Gp2 family protein [Vibrio cholerae]	
1	CDS	2606101	2605427	-	atgactcatcaactcaacgtggc	MTHQLNVAHQRAHTITHDRTF	fig|127906.87.peg.2359	VCC2359	VC0495		hypothetical protein	inovirus Gp2 family protein [Vibrio cholerae]	
1	CDS	2606807	2606163	-	atgtctaaaaatttaaccatcaca	MSKNLTITHESTFNGNKVYIDK	fig|127906.87.peg.2360	VCC2360	VC0494		hypothetical protein	inovirus Gp2 family protein [Vibrio cholerae]	
1	CDS	2607192	2608067	+	atgccatcaatcgacatagatga	MPSIDIDEDTSVETTTMTETPT	fig|127906.87.peg.2361	VCC2361	VC0493		hypothetical protein	MarR family transcriptional regulator [Vibrio cholerae]	
1	CDS	2608323	2609489	+	atgaaagacaacagtacaagtc	MKDNSTSAVSSWLGYKIQEYF	fig|127906.87.peg.2362	VCC2362	VC0492		hypothetical protein		
1	CDS	2609482	2609994	+	atgcttaaacctgaaaataattta	MLKPENNLEKEAWEINNPAM(	fig|127906.87.peg.2363	VCC2363	VC0491		hypothetical protein		
1	CDS	2609995	2611956	+	atgtttttcaaatagaaaaggttc	MFFQIEKVVLWSKEAKHKPRV	fig|127906.87.peg.2364	VCC2364	VC0490		Plasmid related protein	DUF3732 domain-containing protein [Vibrio cholerae]	
1	CDS	2612716	2612976	+	atgctttctacttctccatttgttctg	MLSTSPFVLPRKTPFGLGEHL	fig|127906.87.peg.2365	VCC2365	VC0489		Putative hemolysin		ORF split into 2 ORFs in C6706, but 1 ORF in remaining strains
1	CDS	2612986	2614476	+	ttggctgagctgctgctttgcgttc	MAELLLCVRSDVKILANQYLKL	fig|127906.87.peg.2366	VCC2366			Putative hemolysin		
1	CDS	2614733	2615812	+	atgccaatcacaacctatttaaa	MPITTYLNKSRIMLALVLSTVL(	fig|127906.87.peg.2367	VCC2367	VC0488		TRAP transporter solute receptor, unknown substrate 6		
1	CDS	2617705	2615873	-	atgtgtggaattgttggtgcggttg	MCGIVGAVAQRDVAEILVQGL	fig|127906.87.peg.2368	VCC2368	VC0487		Glutamine--fructose-6-phosphate aminotransferase [isomerizing] (EC 2.6.1.16)		
1	CDS	2618538	2617768	-	atgtcgaaacgaaatacacaac	MSKRNTQLRRHTIVKLVNEQG	fig|127906.87.peg.2369	VCC2369	VC0486	srlR	Transcriptional regulator of glmS gene, DeoR family		
1	CDS	2618966	2620378	+	atgaaaaagaccaaaatcgtat	MKKTKIVCTIGPKTESVEKLTE	fig|127906.87.peg.2370	VCC2370	VC0485	pykF	Pyruvate kinase (EC 2.7.1.40)		
1	CDS	2620581	2620450	-	atgttgaatcatcttctggtcagac	MLNHLLVRLTIGCLLVLGIKLSA	fig|127906.87.peg.2371	VCC2371	VC0484		FIG01199891: hypothetical protein		
1	CDS	2621609	2620914	-	atgaaattatttgcaccggtggttt	MKLFAPVVLLLSVLSSPTFAQE	fig|127906.87.peg.2372	VCC2372	VC0483		Protein of unknown function DUF541	oxidative stress defense protein [Vibrio cholerae]	
1	CDS	2622606	2621710	-	atgcgtggtctggattatcggtgg	MRGLDYRWIEALDSVVSKGSF	fig|127906.87.peg.2373	VCC2373	VC0482	iciA	Transcriptional regulator ArgP, LysR family		
1	CDS	2622771	2623406	+	atgaactggtggattctgctacaa	MNWWILLQGFSLGATMIIPIGA	fig|127906.87.peg.2374	VCC2374	VC0481	argO	Arginine exporter protein ArgO		
1	CDS	2624333	2623470	-	atggctggtgaatcgattggtgtt	MAGESIGVEVPIVESFNQVNT\	fig|127906.87.peg.2375	VCC2375	VC0480		Protein involved in stability of MscS mechanosensitive channel		
					atgcgctattttgccaaaattctgacttttggtggtttatttgagtgggatgtttatca		From N16961	Not annotated as CDS	VC0479				
1	CDS	2625705	2624629	-	atgtctaagatctttgattttgtaaa	MSKIFDFVKPGVISGDDVQKV	fig|127906.87.peg.2376	VCC2376	VC0478	fbaA	Fructose-bisphosphate aldolase class II (EC 4.1.2.13)		
1	CDS	2627024	2625861	-	atgtctgtaatcaagatgattgac	MSVIKMIDLDLAGKRVFIRADL	fig|127906.87.peg.2377	VCC2377	VC0477	pgk	Phosphoglycerate kinase (EC 2.7.2.3)		
1	CDS	2628261	2627173	-	gtgccaaagcttagagtatcggt	MPKLRVSVTIAKQSDSDRRDT	fig|127906.87.peg.2378	VCC2378	VC0476	epd	D-erythrose-4-phosphate dehydrogenase (EC 1.2.1.72)		
1	CDS	2630308	2628350	-	atgtccagattcaatccatccccc	MSRFNPSPVSLSVTLGLMFSA	fig|127906.87.peg.2379	VCC2379	VC0475	cirA/irgA	TonB-dependent receptor; Enterobactin receptor IrgA		
1	CDS	2630453	2631349	+	atgcaagatctcagcgccgtaa	MQDLSAVKAFHALCQHKSLTA	fig|127906.87.peg.2380	VCC2380	VC0474	irgB	Iron-regulated virulence regulatory protein irgB		
1	repeat	2631349	2633410	+	aaaaatcccgccgcagcgggatttttatcatcgatttttctttgcgtaacaaag		fig|127906.87.repeat.48				repeat region		
1	CDS	2633418	2631421	-	atgtcttctcgcaaacaacttgcc	MSSRKQLANAIRALSMDGVQK	fig|127906.87.peg.2381	VCC2381	VC0473		Transketolase (EC 2.2.1.1)		
1	CDS	2633455	2633610	+	atgcggaaaatcagtggcggct	MRKISGGYCRKAPLLLQTIYW	fig|127906.87.peg.2382	VCC2382	No refSeq annotation		FIG01199596: hypothetical protein	Membrane-bound lytic murein transglycosylase C [Vibrio cholerae HC-61A1]	
1	CDS	2633783	2634940	+	atggcgattaagcacctgtttact	MAIKHLFTSESVSEGHPDKIAD	fig|127906.87.peg.2383	VCC2383	VC0472	metK	S-adenosylmethionine synthetase (EC 2.5.1.6)		
1	CDS	2635062	2635550	+	atggttcgttttgatctgcactgcc	MVRFDLHCQAEQKIKQCIATA	fig|127906.87.peg.2384	VCC2384	VC0471	sprT	Protein SprT		
1	CDS	2635678	2636373	+	atgatgatttttcgtttcgttaccac	MMIFRFVTTLAASLPLLTFAAP	fig|127906.87.peg.2385	VCC2385	VC0470	dns	Endonuclease I precursor (EC 3.1.21.1) @ Extracellular deoxyribonuclease Dns (EC 3.1.21.-)		
1	CDS	2636511	2637242	+	atgcgcatccctcgaatttatcatc	MRIPRIYHPEPIHQLGTVALSE	fig|127906.87.peg.2386	VCC2386	VC0469	rsmE	16S rRNA (uracil(1498)-N(3))-methyltransferase (EC 2.1.1.193)		
1	CDS	2637256	2638212	+	atgattaaactcggtattgtgatg	MIKLGIVMDPIASINIKKDSSFA	fig|127906.87.peg.2387	VCC2387	VC0468	gshB	Glutathione synthetase (EC 6.3.2.3)		
1	CDS	2638248	2638811	+	atgaatcttaccaatcattttctgg	MNLTNHFLVAMPSMKDPYFKI	fig|127906.87.peg.2388	VCC2388	VC0467		UPF0301 protein YqgE	glutathione synthetase [Vibrio cholerae]	
1	CDS	2638892	2639314	+	atgtcacgtaccgttatggccttt	MSRTVMAFDYGTKSIGSAIGQ	fig|127906.87.peg.2389	VCC2389	VC0466	yqgF	Putative pre-16S rRNA nuclease YqgF		
1	CDS	2640632	2639352	-	atgaattcacagcaacttttgatc	MNSQQLFDDLQQRGLIAQSTF	fig|127906.87.peg.2390	VCC2390	VC0465	tyrS-1	Tyrosyl-tRNA synthetase (EC 6.1.1.1)		
1	CDS	2641538	2640741	-	atgccgatcacgactcaagagt	MPITTQEYLALGDAIAALKTPQ	fig|127906.87.peg.2391	VCC2391	VC0464	ttrR	Transcriptional regulator, LuxR family		
1	CDS	2642762	2641656	-	atggagttgaatcaatatctggat	MELNQYLDGMLTHKASDLYIT	fig|127906.87.peg.2392	VCC2392	VC0463	pilU	Type IV pilus assembly ATPase component PilU		
1	CDS	2643814	2642777	-	atggatatcgctgagttactggaa	MDIAELLEFSVKHNASDLHLSA	fig|127906.87.peg.2393	VCC2393	VC0462	pilT-1	Twitching motility protein PilT		
1	CDS	2643840	2644550	+	atgcgtagtattcaacaaaacat	MRSIQQNIEHITAQIESAQQKC	fig|127906.87.peg.2394	VCC2394	VC0461		Pyridoxal phosphate-containing protein YggS		
1	CDS	2644603	2645421	+	atggaacagagagaagcatcgcc	MEQRSIAFIGAGNMARAIIAGL	fig|127906.87.peg.2395	VCC2395	VC0460	proC	Pyrroline-5-carboxylate reductase (EC 1.5.1.2)		
1	CDS	2645475	2646032	+	atgaattcactgagctttctcatca	MNSLSFLINTLFDLYIMVVILRI\	fig|127906.87.peg.2396	VCC2396	VC0459		Cell division integral membrane protein, YggT and half-length relatives		
1	CDS	2646032	2646322	+	atggctgcagcatggcgcgaag	MAAAWREGDDLLLRLYIQPKA	fig|127906.87.peg.2397	VCC2397	VC0458		UPF0235 protein VC0458	YggU family protein [Vibrio cholerae]	
1	CDS	2646368	2646799	+	atgcgtaaatggatgatgtcacta	MRKWMMSLLLTLLALPSAEC	fig|127906.87.peg.2398	VCC2398	VC0457		FIG003551: hypothetical protein	DUF4426 domain-containing protein [Vibrio cholerae]	
1	CDS	2646869	2647471	+	atgaaaaagatcgttttagccac	MKKIVLATGNQGKVREMADLL	fig|127906.87.peg.2399	VCC2399	VC0456	rdgB	Nucleoside 5-triphosphatase RdgB (dHAPTP, dITP, XTP-specific) (EC 3.6.1.66)		
1	CDS	2647465	2648640	+	atgctaacgcctccatcgcttagc	MLTPPSLSLYIHIPWCVQKCPY	fig|127906.87.peg.2400	VCC2400	VC0455		Oxygen-independent coproporphyrinogen-III oxidase-like protein YggW		
1	CDS	2649781	2648861	-	atgaagccaacagcagacattt	MKPTADILASIIEEVRPLTSKGI	fig|127906.87.peg.2401	VCC2401	VC0454	glsA2	Glutaminase (EC 3.5.1.2)		
1	CDS	2650611	2649892	-	atgagcgaagtcatcacccagg	MSEVITQEYTEDGKVLRRIRSI	fig|127906.87.peg.2402	VCC2402	VC0453	trmB	tRNA (guanine(46)-N(7))-methyltransferase (EC 2.1.1.33)		
1	CDS	2650669	2650830	+	gtgcaagtgttattactgaccgttt	MQVLLLTVYHPALKLFASAAN	fig|127906.87.peg.2403	VCC2403	No refSeq annotation		FIG01204984: hypothetical protein		
1	CDS	2650851	2651912	+	gtgactcctttgcacaggccatc	MTPFAQAILTWYDAYGRKNLF	fig|127906.87.peg.2404	VCC2404	VC0452	mutY	A/G-specific adenine glycosylase (EC 3.2.2.-)		
1	CDS	2651926	2652198	+	atggctcgcaccgtattttgtacc	MARTVFCTRLQKEADGLDFQL	fig|127906.87.peg.2405	VCC2405	VC0451	yggX	FIG001341: Probable Fe(2+)-trafficking protein YggX		
1	CDS	2652300	2653427	+	atgagaaagttagtgttatgtatc	MRKLVLCITALLLSGCSREFIEI	fig|127906.87.peg.2406	VCC2406	VC0450	mltC	Membrane-bound lytic murein transglycosylase C (EC 3.2.1.n1)		
1	rna	2653537	2653612	+	gcccggatagctcagtcggtagagcagaggattgaaaatcctcgtgtcggtc		fig|127906.87.rna.73	VCCt058	VCt037		tRNA-Phe-GAA		
1	rna	2653661	2653736	+	gccggcttagctcagtaggtagagcaactgacttgtaatcagtaggtcgcca		fig|127906.87.rna.74	VCCt059	VCt036		tRNA-Thr-TGT		
1	rna	2653747	2653822	+	gcctcgatagctcagtcggtagagcagaggattgaaaatcctcgtgtcggtg		fig|127906.87.rna.75	VCCt060	VCt035		tRNA-Phe-GAA		
1	rna	2653860	2653935	+	tcctccttagctcagtcggtagagcgacggactgttaatccgcaggtcgctgg		fig|127906.87.rna.76	VCCt061	VCt034		tRNA-Asn-GTT		
1	CDS	2654191	2655816	+	atgaaattaaaaacccaagctta	MKLKTQAYLLSAIILAALLALTA	fig|127906.87.peg.2407	VCC2407	VC0449		N-acetylglucosamine regulated methyl-accepting chemotaxis protein		
1	rna	2656231	2656306	+	gcccggatagctcagtcggtagagcagaggattgaaaatcctcgtgtcggtc		fig|127906.87.rna.77	VCCt062	VCt033		tRNA-Phe-GAA		
1	rna	2656348	2656423	+	gccgacttagctcagtaggtagagcaactgacttgtaatcagtaggtcacca		fig|127906.87.rna.78	VCCt063	VCt032		tRNA-Thr-TGT		
1	rna	2656439	2656514	+	tcctccttagctcagtcggtagagcgacggactgttaatccgcaggtcgctgg		fig|127906.87.rna.79	VCCt064	VCt031		tRNA-Asn-GTT		
1	rna	2656543	2656618	+	gccgacttagctcagtaggtagagcaactgacttgtaatcagtaggtcacca		fig|127906.87.rna.80	VCCt065	VCt030		tRNA-Thr-TGT		
1	rna	2656633	2656708	+	tcctccttagctcagtcggtagagcgacggactgttaatccgcaggtcgctgg		fig|127906.87.rna.81	VCCt066	VCt029		tRNA-Asn-GTT		
1	CDS	2657717	2656863	-	atgcatatttttggcaaaattcttgg	MHIFGKILGAFFGFLFGGPFGA	fig|127906.87.peg.2408	VCC2408	VC0447	djlA	DnaJ-like protein DjlA		
1	CDS	2657876	2660227	+	atgtcatgcttttcccgcacgtttt	MSCFSRTFLAASISAALFAPQI	fig|127906.87.peg.2409	VCC2409	VC0446	lptD/imp	LPS-assembly protein LptD @ Organic solvent tolerance protein precursor		
1	CDS	2660280	2661575	+	atgaaattgtggaaacccacac	MKLWKPTLISVLSALTLFNAHA	fig|127906.87.peg.2410	VCC2410	VC0445	surA	Periplasmic chaperone and peptidyl-prolyl cis-trans isomerase of outer membrane proteins SurA (EC 5.2.1.8)		
1	CDS	2661565	2662557	+	atgagcagtaaacgcatcattgt	MSSKRIIVTAGEPAGIGPDLVL	fig|127906.87.peg.2411	VCC2411	VC0444	pdxA	4-hydroxythreonine-4-phosphate dehydrogenase (EC 1.1.1.262)		
1	CDS	2662571	2663386	+	atgagaaatgatgtccacttagg	MRNDVHLGHKARKRFGQNFL	fig|127906.87.peg.2412	VCC2412	VC0443	ksgA	SSU rRNA (adenine(1518)-N(6)/adenine(1519)-N(6))-dimethyltransferase (EC 2.1.1.182)		
1	CDS	2663458	2663838	+	atggatgtttcgctgccctgtatca	MDVSLPCIKIQVQTRYIEEQSN	fig|127906.87.peg.2413	VCC2413	VC0442	apaG	ApaG protein		
1	CDS	2663849	2664658	+	gtggcgaattatatcgttggagat	MANYIVGDIQGCFDELQQLLK	fig|127906.87.peg.2414	VCC2414	VC0441	apaH	Bis(5'-nucleosyl)-tetraphosphatase, symmetrical (EC 3.6.1.41)		
1	CDS	2665304	2664807	-	atgatgatcagtatgattgccgcc	MMISMIAAMADQRIIGKDNQM	fig|127906.87.peg.2415	VCC2415	VC0440	folA	Dihydrofolate reductase (EC 1.5.1.3)		
1	CDS	2665790	2665317	-	atgttaagctgggatctgtttttcg	MLSWDLFFGLLNDMFFAAIPA	fig|127906.87.peg.2416	VCC2416	VC0439		FIG001826: putative inner membrane protein		
1	CDS	2666551	2665790	-	atggaatccaaacagagagcc	MESKQRAVSRLIAQAGQMLLA	fig|127906.87.peg.2417	VCC2417	VC0438	yjjP	FIG023911: putative membrane protein	threonine/serine exporter ThrE family protein [Vibrio cholerae]	
1	CDS	2667845	2666673	-	atgaaattcgtagatgaagcggt	MKFVDEAVIKVQAGDGGNGV	fig|127906.87.peg.2418	VCC2418	VC0437	obgE	GTP-binding protein Obg		

1	CDS	2668328	2668068	-	atggcacacaaaaaagctggtg	MAHKKAGGSTRNGRDSESKR	fig|127906.87.peg.2419	VCC2419	VC0436	rpmA	LSU ribosomal protein L27p		
1	CDS	2668660	2668349	-	atgtacgctgtttccaatctggtg	MYAVFQSGGKQHRVSEGQTL	fig|127906.87.peg.2420	VCC2420	VC0435	rplU	LSU ribosomal protein L21p		
1	CDS	2668931	2669902	+	atggattttaaagctatccaagcc	MDFKAIQALTANDMAKVNETII	fig|127906.87.peg.2421	VCC2421	VC0434	ispB	Octaprenyl diphosphate synthase (EC 2.5.1.90)		
1	CDS	2671457	2670051	-	atggataaaaaactgggtttagg	MDKKLGLGSLTALVIGSMIGA	fig|127906.87.peg.2422	VCC2422	VC0433	ydgI	Arginine/ornithine antiporter ArcD		
1	CDS	2672866	2671931	-	atgaaagtcgctgttattggtgcc	MKVAVIGAAGGIGQALALLLKN	fig|127906.87.peg.2423	VCC2423	VC0432	mdh	Malate dehydrogenase (EC 1.1.1.37)		
1	CDS	2673146	2673664	+	atgcgagaatatgcacaaatcg	MREYAQIDHLTKVRRTMRNVI	fig|127906.87.peg.2424	VCC2424	VC0431	argR	Arginine pathway regulatory protein ArgR, repressor of arg regulon		
1	CDS	2673988	2674956	+	atgtcattacctaaaataatcaa	MSLPKIIKMGAIAAAVIGSGVA	fig|127906.87.peg.2425	VCC2425	VC0430		TRAP transporter solute receptor, TAXI family precursor		
1	CDS	2675127	2677691	+	atgacgcagacacaacaccg	MTQTTTPSQDVQEMVAQADT	fig|127906.87.peg.2426	VCC2426	VC0429		TRAP-type uncharacterized transport system, fused permease component		
1	CDS	2677813	2678250	+	atgtataaacatattcttgttccgg	MYKHILVPVDLNEQGFADKAV	fig|127906.87.peg.2427	VCC2427	VC0428	uspF	Universal stress protein family 3		
1	CDS	2678401	2678784	+	atgaaaaagacattaagttttgc	MKKTLSFAAIHFTIAFTVAYVL	fig|127906.87.peg.2428	VCC2428	VC0426		Uncharacterized membrane protein SO_4740 / Uncharacterized membrane protein SO_4740	DUF2061 domain-containing protein [Vibrio cholerae]	
1	CDS	2679650	2678853	-	atgcagctctcactcgccggcct	MQLSLAGLWQLSPLTDLSIPQ	fig|127906.87.peg.2429	VCC2429	VC0425		Beta-galactosidase/beta-glucuronidase		
1	CDS	2680219	2679797	-	atgtctcaccaagatgattatcta	MSHQDDYLSVEELIEIQKEETF	fig|127906.87.peg.2430	VCC2430	VC0424	rraB	Ribonuclease E inhibitor RraB		
1	CDS	2680946	2682169	+	atgaatcgtttgtatgtgggctctg	MNRLYVGSEVGQLRRVLLNR	fig|127906.87.peg.2431	VCC2431	VC0423	arcA	Arginine deiminase (EC 3.5.3.6)		
1	CDS	2683710	2682265	-	atgacgatgaaccaaattgaaa	MTMNQIETALLAPAGLTEQDIA	fig|127906.87.peg.2432	VCC2432	VC0422	tldD	TldD protein, part of TldE/TldD proteolytic complex		
1	CDS	2684537	2683710	-	atgcaacgagttggactggtgca	MQRVGLVQMTSGPEISRNLTY	fig|127906.87.peg.2433	VCC2433	VC0421	ramA	FIG003879: Uncharacterized subgroup of the nitrilase superfamily	carbon-nitrogen hydrolase family protein [Vibrio cholerae]	
1	CDS	2688385	2684546	-	atgtggctgctggtcagcctgctg	MWLLVSLLIVLALAVTALRILLP	fig|127906.87.peg.2434	VCC2434	VC0420		FIG005080: Possible exported protein	TIGR02099 family protein [Vibrio cholerae]	
1	CDS	2689903	2688434	-	atgagtgcagagttgctgttaaa	MSAELLLNVTPSETRVAMIEG	fig|127906.87.peg.2435	VCC2435	VC0419	rng/cafA	Ribonuclease G		
1	CDS	2690492	2689929	-	atgacaatatcgaaattagtgttg	MTISKLVLASGSPRRRELLAQI	fig|127906.87.peg.2436	VCC2436	VC0418	maf	Septum formation protein Maf		Limited query cover for certain strains
1	CDS	2690977	2690489	-	atgctcagcagtgatattaaagg	MLSSDIKGRMVILASFLLALILC	fig|127906.87.peg.2437	VCC2437	VC0417	mreD	Rod shape-determining protein MreD		
1	CDS	2691854	2690964	-	atgaaacctatctttggtcgagga	MKPIFGRGPSLQLRLFFAVML	fig|127906.87.peg.2438	VCC2438	VC0416	mreC	Rod shape-determining protein MreC		
1	CDS	2692954	2691911	-	atgttcaaaaaacttcgtggcatg	MFKKLRGMFSNDLSIDLGTAN	fig|127906.87.peg.2439	VCC2439	VC0415	mreB	Rod shape-determining protein MreB		
1	CDS	2696880	2693122	-	gtgaataagtggttagccaaaa	MNKWLAKTLVGLGCFALLSAY	fig|127906.87.peg.2440	VCC2440	VC0414	mshQ	MSHA biogenesis protein MshQ		
1	CDS	2697298	2696873	-	atgatgctctggtctacaacaca	MMLWSTTHRKQQGNALILVVF	fig|127906.87.peg.2441	VCC2441	VC0413	mshP	MSHA biogenesis protein MshP		
1	CDS	2698040	2697270	-	atgagccgtggtttcaccctaatc	MSRGFTLIEMVITIILLGIVGLFL	fig|127906.87.peg.2442	VCC2442	VC0412	mshO	MSHA biogenesis protein MshO		
1	CDS	2698609	2698040	-	atgcctgttaagccgatgattgct	MPVKPMIAKRGFTLVEMIIVIV	fig|127906.87.peg.2443	VCC2443	VC0411	mshD	MSHA pilin protein MshD		
1	CDS	2699087	2698599	-	atgaaaattataggtaaaggtttt	MKIIGKGFTLIELVVVIILLGILSA	fig|127906.87.peg.2444	VCC2444	VC0410	mshC	MSHA pilin protein MshC		
1	CDS	2699651	2699124	-	atgaaaagacaaggtggtttcag	MKRQGGFTLIELVVVIVILGILA	fig|127906.87.peg.2445	VCC2445	VC0409	mshA	MSHA pilin protein MshA		
1	CDS	2700276	2699686	-	atgcacgccgtacgttacgtaag	MHAVRYVSELKNMKKMQQGF	fig|127906.87.peg.2446	VCC2446	VC0408	mshB	MSHA pilin protein MshB		
1	CDS	2700773	2700321	-	atgaaaataggatttgagcgcgt	MKIGFERVRFVLWFVLVVVLLT	fig|127906.87.peg.2447	VCC2447	VC0407	mshF	MSHA biogenesis protein MshF		
1	CDS	2701999	2700776	-	atggcgacgttttattatcagggg	MATFYYQGRNADGSKASGLV	fig|127906.87.peg.2448	VCC2448	VC0406	mshG	MSHA biogenesis protein MshG		
1	CDS	2703735	2702008	-	gtgccaattaataaactgcgtaa	MPINKLRKRLGDLLVEEGIVSE	fig|127906.87.peg.2449	VCC2449	VC0405	mshE	MSHA biogenesis protein MshE		
1	CDS	2704876	2703725	-	atgagtgtggtgaatgaagcatt	MSVVNEALAKSAQRSHSRLSI	fig|127906.87.peg.2450	VCC2450	VC0404	mshN	MSHA biogenesis protein MshN		
1	CDS	2705718	2704873	-	atgtatttatcgcactttggccttga	MYLSHFGLEQLPFHLTPDTEL	fig|127906.87.peg.2451	VCC2451	VC0403	mshM	MSHA biogenesis protein MshM		
1	CDS	2707402	2705723	-	atgcgtaaaatcgtactcgcttca	MRKIVLASVVTSLVGCSMGHR	fig|127906.87.peg.2452	VCC2452	VC0402	mshL	MSHA biogenesis protein MshL		
1	CDS	2707734	2707417	-	gtggttagatatggtgtgctcttgg	MVRYGVLLALIAVTYSYAGEDI	fig|127906.87.peg.2453	VCC2453	VC0401	mshK	MSHA biogenesis protein MshK		
1	CDS	2708377	2707727	-	atgaaatcgggttggcaaaactt	MKSGWQNFESRFSALSQREK	fig|127906.87.peg.2454	VCC2454	VC0400	mshJ	MSHA biogenesis protein MshJ		
1	CDS	2709813	2708374	-	atgaagaaaccgagttggatag	MKKPSWIEKLIAPKVASQQLY	fig|127906.87.peg.2455	VCC2455	VC0399	mshI	MSHA biogenesis protein MshI		
1	CDS	2711838	2709826	-	atgaggtatacaccgacattaa	MRYTPTLKLSTRLVAFVTMTV	fig|127906.87.peg.2456	VCC2456	VC0398	mshH	MSHA biogenesis protein MshH		
1	CDS	2712495	2711962	-	atggcaagccgtggcgtgaaca	MASRGVNKVILIGNLGQDPEV	fig|127906.87.peg.2457	VCC2457	VC0397	ssb	Single-stranded DNA-binding protein		
1	CDS	2712787	2713431	+	atgcaacgagccaactatgcac	MQRANYARTIYLLTTQPKALH	fig|127906.87.peg.2458	VCC2458	VC0396		Transcriptional regulator, LuxR family		
1	CDS	2713601	2714476	+	atgatgattaagaagtgtctattt	MMIKKCLFPAAGYGTRFLPAT	fig|127906.87.peg.2459	VCC2459	VC0395	gtaB/galU	UTP--glucose-1-phosphate uridylyltransferase (EC 2.7.7.9)		
1	CDS	2714632	2717454	+	atggacaaaatcgaagtacgcg	MDKIEVRGARTHNLKNINLTIP	fig|127906.87.peg.2460	VCC2460	VC0394	uvrA	Excinuclease ABC subunit A		
1	CDS	2717519	2719303	+	atggcaactttgctgttaagtggc	MATLLLSGTRLEPHAPNLPLN	fig|127906.87.peg.2461	VCC2461	VC0393		Lipid A core-O-antigen ligase		
1	CDS	2720500	2719382	-	atgacaattcgcagttttatcccg	MTIRSFIPPHRILMGPGPSAIS	fig|127906.87.peg.2462	VCC2462	VC0392	pucG	Serine--pyruvate aminotransferase (EC 2.6.1.51) / L-alanine:glyoxylate aminotransferase (EC 2.6.1.44)		
1	CDS	2720964	2722319	+	gtgagcacatttaacgtcgccaa	MSTFNVAKFGGTSVANFEAM	fig|127906.87.peg.2463	VCC2463	VC0391	lysC	Aspartokinase (EC 2.7.2.4)		
1	CDS	2726072	2722392	-	gtgggaaaagaagtaagacaa	MGKEVRQQLEQQLKQRILLID	fig|127906.87.peg.2464	VCC2464	VC0390	metH	5-methyltetrahydrofolate--homocysteine methyltransferase (EC 2.1.1.13)		
1	CDS	2726269	2727603	+	atgtccgtctactacactttgtgct	MSVYYTLCFLSAAAMLIAFINS	fig|127906.87.peg.2465	VCC2465	VC0389	nhaK	Putative Na+/H+ antiporter		
1	repeat	2727901	2739541	+	ggataaacgtaaaaagagaaagacaaaaagagcagtaagaagagttgg		fig|127906.87.repeat.49				repeat region		
1	rna	2728153	2728077	-	ggagcggtagttcagtcggttagaataccggcctgtcacgccgggggtcgc		fig|127906.87.rna.82	VCCt067	VCt028		tRNA-Asp-GTC		
1	rna	2728297	2728176	-	ttgcttggcgaccatagcgttttggacccacctgattccatcccgaactcagaa		fig|127906.87.rna.83	VCr015	Not annotated as CDS or not present		5S rRNA ## 5S ribosomal RNA		
1	rna	2731310	2728422	-	ggttaagtgactaagcgtacacggtggatgcctgggcagtcagaggcgatg		fig|127906.87.rna.84	VCr016	Not annotated as CDS or not present		LSU rRNA ## 23S rRNA, large subunit ribosomal RNA		
1	rna	2731728	2731653	-	gggcgattagctcagttgggagagcacctgccttacaagcagggggtcact		fig|127906.87.rna.85	VCCt068	Not annotated as CDS or not present		tRNA-Val-TAC		
1	rna	2731833	2731759	-	gggtcgttagctcagtggtagagcagttggcttttaaccaattggtcataggttc		fig|127906.87.rna.86	VCCt069	Not annotated as CDS or not present		tRNA-Lys-TTT		
1	rna	2731928	2731853	-	gtcccgttcgtctagaggcctaggacaccgccctttcacggcggtaacaggg		fig|127906.87.rna.87	VCCt070	Not annotated as CDS or not present		tRNA-Glu-TTC		
1	CDS	2733705	2733508	-	gtgtgggcactcgttgatgataat	MWALVDDNQKRFINELSDHLN	fig|127906.87.peg.2466	VCC2466	VC0388		hypothetical protein		Limited query cover for certain strains
1	rna	2734017	2733896	-	ttgcttggcgaccatagcgttttggacccacctgattccatcccgaactcagaa		fig|127906.87.rna.88	VCr016	VCr012		5S rRNA ## 5S ribosomal RNA		
1	rna	2737030	2734142	-	ggttaagtgactaagcgtacacggtggatgcctgggcagtcagaggcgatg		fig|127906.87.rna.89	VCr017	VCr011		LSU rRNA ## 23S rRNA, large subunit ribosomal RNA		
1	rna	2737393	2737318	-	ggggttatagctcagctgggagagcgcctgccttgcacgcaggaggtctgc		fig|127906.87.rna.90	VCCt071	VCt027		tRNA-Ala-TGC		
1	rna	2737478	2737402	-	gggtctgtagctcaggtggttagagcgtacgcctgataagcgtaaggtcggt		fig|127906.87.rna.91	VCCt072	VCt026		tRNA-Ile-GAT		
1	rna	2739086	2737539	-	ttttaattgaagagtttgatcatggctcagattgaacgctggcggcaggccta		fig|127906.87.rna.92	VCr018	VCr010		SSU rRNA ## 16S rRNA, small subunit ribosomal RNA		
1	CDS	2739230	2739111	-	gtgtgggcactcgttgatgataat	MWALVDDNQKRFINELSDHLN	fig|127906.87.peg.2467	VCC2467	VC0388		hypothetical protein		
1	CDS	2740381	2739620	-	atgcctaatcgtaccgttccaacc	MPNRTVPTLEELLTLNKVQQT	fig|127906.87.peg.2468	VCC2468	VC0386	cysH	Phosphoadenylyl-sulfate reductase [thioredoxin] (EC 1.8.4.8)		
1	CDS	2742107	2740374	-	atgagcgcaaatcaaaaccca	MSANQNPSVQEVLGEVLGPW	fig|127906.87.peg.2469	VCC2469	VC0385	cysI	Sulfite reductase [NADPH] hemoprotein beta-component (EC 1.8.1.2)		
1	CDS	2743930	2742107	-	atgtctacaggaaacactttacc	MSTGNTLPPALAALASPLNDA	fig|127906.87.peg.2470	VCC2470	VC0384	cysJ	Sulfite reductase [NADPH] flavoprotein alpha-component (EC 1.8.1.2)		
1	CDS	2744267	2744037	-	atgacgattccggagttaagag	MTIPELRELYQTQQLVEAIIEPS	fig|127906.87.peg.2471	VCC2471	VC0383		Thymidylate kinase		
1	CDS	2744413	2744264	-	ttgtggggctcaatttagcttctg	MWGLNLASDEFLIIEFFANQY	fig|127906.87.peg.2472	VCC2472	VC0382		ABC-type Fe3+-hydroxamate transport system, periplasmic component		
1	CDS	2745053	2744388	-	atgaaccgaactgcatcggcgg	MNRTASAALVTALMVMPLSAS	fig|127906.87.peg.2473	VCC2473	VC0381		ABC-type Fe3+-hydroxamate transport system, periplasmic component		
1	CDS	2745354	2745142	-	atgatgtttgaattaatctttattttg	MMFELIFIFVFAATLLVTGITLM	fig|127906.87.peg.2474	VCC2474	No refSeq annotation	pspG	Phage shock protein G		
1	CDS	2745385	2745501	+	ttgcagttttcacgccaactttaca	MQFSRQLYKEANHLINIEKNKF	fig|127906.87.peg.2475	VCC2475	VC0380		FIG01203466: hypothetical protein		
1	CDS	2746498	2745515	-	atgactcactcatgcagactctca	MTHSCRLSVAPMLDWTDRHC	fig|127906.87.peg.2476	VCC2476	VC0379		tRNA-dihydrouridine(20/20a) synthase (EC 1.3.1.91)		
1	CDS	2746856	2747320	+	atggtgatggttttggacaacaa	MVMVLDNKLTKQIEEICAARG	fig|127906.87.peg.2477	VCC2477	VC0378	zur	Zinc uptake regulation protein Zur		
1	CDS	2747330	2747791	+	atgcgcgctgaatttgtaaaccc	MRAEFVNPFLASLMNVLKTMA	fig|127906.87.peg.2478	VCC2478	VC0377	cheX	Chemotaxis protein CheX		
1	CDS	2749086	2747908	-	atgtcagaaacggccattcaga	MSETAIQTKTQARQRKVFAHH	fig|127906.87.peg.2479	VCC2479	VC0376		Membrane protein	MBL fold metallo-hydrolase [Vibrio cholerae]	
1	CDS	2750941	2749289	-	atgttgaaaaatatcaatccaac	MLKNINPTQTQAWKALTAHFE	fig|127906.87.peg.2480	VCC2480	VC0374	pgi	Glucose-6-phosphate isomerase (EC 5.3.1.9)		
1	CDS	2751226	2751645	+	atgtggcatcagcacaccatcca	MWHQHTIQLRPRARGFHLITD	fig|127906.87.peg.2481	VCC2481	VC0373		UPF0047 protein YjbQ	YjbQ family protein [Vibrio cholerae]	
1	CDS	2752822	2751728	-	atgaactatatgaaggcggcca	MNYMKAATAYINLEALQHNLC	fig|127906.87.peg.2482	VCC2482	VC0372	alr	Alanine racemase (EC 5.1.1.1)		
1	CDS	2754233	2752827	-	atggcagatccacgtactgataa	MADPRTDNRNRKIPDAQVDAI	fig|127906.87.peg.2483	VCC2483	VC0371	dnaB	Replicative DNA helicase (DnaB) (EC 3.6.4.12)		
1	CDS	2754404	2755168	+	gtgtccaccaaatggctttaggc	MSTKWLLGISLMLSTVAQANE	fig|127906.87.peg.2484	VCC2484	VC0370		Membrane protein	YdiY family protein [Vibrio cholerae]	
1	CDS	2755679	2755230	-	atgcaagttattctacttgataaa	MQVILLDKIGNLGSLGDTVNVK	fig|127906.87.peg.2485	VCC2485	VC0369	rplI	LSU ribosomal protein L9p		
1	CDS	2755940	2755713	-	atggctcgtttcttccgtcgtcgta	MARFFRRRKFCRFTAEGVQE	fig|127906.87.peg.2486	VCC2486	VC0368	rpsR	SSU ribosomal protein S18p @ SSU ribosomal protein S18p, zinc-independent		
1	CDS	2756258	2755956	-	atgaccaatcgaatcgagttgag	MTNRIELSGVVAKALVRSQSP	fig|127906.87.peg.2487	VCC2487	VC0367	priB	Primosomal replication protein N		Limited query cover for certain strains
1	CDS	2756634	2756266	-	atgcgtcattacgaaatcgtattc	MRHYEIVFMVHPDQSEQVAG	fig|127906.87.peg.2488	VCC2488	VC0366	rpsF	SSU ribosomal protein S6p		
1	CDS	2757322	2756846	-	atgaaaggtgatccagtcataat	MKGDPVIIQHLNKVLANELIAIN	fig|127906.87.peg.2489	VCC2489	VC0365	bfr	Bacterioferritin (EC 1.16.3.1)		
1	CDS	2757580	2757392	-	atgtacgtttgtttgtgtcatggtgt	MYVCLCHGVSDKKIRRLVAEC	fig|127906.87.peg.2490	VCC2490	VC0364	bfd	Bacterioferritin-associated ferredoxin		Limited query cover for certain strains
1	repeat	2757882	2759066	+	attaagcgatgatcttagctacaacaccagcacctacagtacggccaccttc		fig|127906.87.repeat.50				repeat region		
1	CDS	2759067	2757883	-	gtgtctaaagaaaaatttgaacg	MSKEKFERTKPHVNVGTIGHV	fig|127906.87.peg.2491	VCC2491	VC0362		Translation elongation factor Tu		
1	CDS	2761286	2759190	-	gtggctcgtaaaactcctattgac	MARKTPIERYRNIGICAHVDAG	fig|127906.87.peg.2492	VCC2492	VC0361		Translation elongation factor G		
1	CDS	2761832	2761362	-	atgccacgtcgtcgcgtcattgg	MPRRRVIGQRKILPDPKFKSEI	fig|127906.87.peg.2493	VCC2493	VC0360	rpsG	SSU ribosomal protein S7p (S5e)		
1	CDS	2762322	2761948	-	atggcaactattaaccagttggtt	MATINQLVRKPRAKQVVKSNV	fig|127906.87.peg.2494	VCC2494	VC0359	rpsL	SSU ribosomal protein S12p (S23e)		
1	CDS	2762782	2762507	-	atgctacatattgttaagcacttag	MLHIVKHLEKLPLLSAYLLDGD	fig|127906.87.peg.2495	VCC2495	VC0358		tRNA 5-methylaminomethyl-2-thiouridine synthase subunit TusB		
1	CDS	2763148	2762792	-	ttgaataaagtcgcatgggtattt	MNKVAWVFHTPPHGSTAGRE	fig|127906.87.peg.2496	VCC2496	VC0357	tusC	tRNA 5-methylaminomethyl-2-thiouridine synthase subunit TusC		
1	CDS	2763540	2763145	-	gtgaaaccactcacttacaccttt	MKPLTYTLVVNGPQYGTQSAF	fig|127906.87.peg.2497	VCC2497	VC0356	tusD	tRNA 5-methylaminomethyl-2-thiouridine synthase subunit TusD		
1	CDS	2764259	2763537	-	gtgacaatgacagaaaccatga	MTMTETMNVDLLLEMESVHVI	fig|127906.87.peg.2498	VCC2498	VC0355		YheO-like PAS domain		
1	CDS	2765237	2764458	-	atgaaatcactttttaaagtgtcac	MKSLFKVSLLAATVMLAVGCC	fig|127906.87.peg.2499	VCC2499	VC0354	fkpA	FKBP-type peptidyl-prolyl cis-trans isomerase FkpA precursor (EC 5.2.1.8)		
1	CDS	2765377	2766357	+	atgcgaagaatttatcactcacto	MRRIYHSLCALLVILMLSGCFF	fig|127906.87.peg.2500	VCC2500	VC0353		FOG: WD40 repeat	vegetatible incompatibility protein HET-E-1 [Vibrio cholerae]	
1	CDS	2766360	2766587	+	atgcaagagatgtcgctaaccc	MQEMSLTQLQERIEDLECKLA	fig|127906.87.peg.2501	VCC2501	VC0352	slyX	Protein SlyX		
1	CDS	2766816	2766628	-	atgtcgcagacactttgggtggc	MSQTLWVALGLVLIVEGLGPL	fig|127906.87.peg.2502	VCC2502	VC0351		Putative inner membrane protein YjeT (clustered with HflC)		
1	CDS	2767857	2766877	-	atgcgtaagttattaatccctagta	MRKLLIPSIVLIIAALLMSMFVIP	fig|127906.87.peg.2503	VCC2503	VC0350	hflC	HflC protein		
1	CDS	2769047	2767860	-	atggcgtggaatgagcctggtaa	MAWNEPGNNNGNNGRDNDP	fig|127906.87.peg.2504	VCC2504	VC0349	hflK	HflK protein		
1	CDS	2770382	2769093	-	ttgtttgaccgttatgaagcggtg	MFDRYEAGERAVLVHVNFTQ	fig|127906.87.peg.2505	VCC2505	VC0348	hflX	Ribosome LSU-associated GTP-binding protein HflX		
1	CDS	2770678	2770415	-	atggctaaggggcaatctctaca	MAKGQSLQDPFLNALRRERIP	fig|127906.87.peg.2506	VCC2506	VC0347	hfq	RNA-binding protein Hfq		
1	CDS	2771797	2770850	-	atgactcaaaagttacctctcgc	MTQKLPLALFLMGPTASGKTD	fig|127906.87.peg.2507	VCC2507	VC0346	miaA	tRNA dimethylallyltransferase (EC 2.5.1.75)		
1	CDS	2773755	2771794	-	atgacgattcgaatcctacccgc	MTIRILPARLANQIAAGEVVER	fig|127906.87.peg.2508	VCC2508	VC0345	mutL	DNA mismatch repair protein MutL		
1	CDS	2775480	2773762	-	atgttgaacgttagccgttttgtatt	MLNVSRFVFLLFGFVLLAIAPC	fig|127906.87.peg.2509	VCC2509	VC0344	amiB	N-acetylmuramoyl-L-alanine amidase (EC 3.5.1.28)		
1	CDS	2775938	2775474	-	atgaacagcaagatttttccta	MNSKIFSLKDEQATIELGRALA	fig|127906.87.peg.2510	VCC2510	VC0343		tRNA threonylcarbamoyladenosine biosynthesis protein TsaE		
1	CDS	2776143	2777252	+	atggactaccaacaactggcaa	MDYQQLANQIKQWAIELGFEK	fig|127906.87.peg.2511	VCC2511	VC0342	queG	Epoxyqueuosine reductase (EC 1.17.99.6) QueG		
1	rna	2777898	2777822	-	cgcggggtggagcagcttggtagctcgtcgggctcataacccgaaggtcgt		fig|127906.87.rna.93	VCCt073	VCt025		tRNA-Met-CAT		
1	rna	2778017	2777941	-	cgcggggtggagcagcttggtagctcgtcgggctcataacccgaaggtcgt		fig|127906.87.rna.94	VCCt074	VCt024		tRNA-Met-CAT		
1	rna	2778136	2778060	-	cgcgggatggagcagcttggtagctcgtcgggctcataacccgaaggtcgt		fig|127906.87.rna.95	VCCt075	VCt023		tRNA-Met-CAT		
1	rna	2778398	2778323	-	gcgacactagctcagttggtagagcgcaaccttgccaaggttgaggtcacg		fig|127906.87.rna.96	VCCt076	VCt022		tRNA-Gly-GCC		
1	rna	2778509	2778433	-	cgcggggtggagcagcttggtagctcgtcgggctcataacccgaaggtcgt		fig|127906.87.rna.97	VCCt077	VCt021		tRNA-Met-CAT		
1	rna	2778624	2778549	-	gcgacactagctcagttggtagagcgcaaccttgccaaggttgaggtcacg		fig|127906.87.rna.98	VCCt078	VCt020		tRNA-Gly-GCC		
1	rna	2778856	2778781	-	gcgacactagctcagttggtagagcgcaaccttgccaaggttgaggtcacg		fig|127906.87.rna.99	VCCt079	VCt019		tRNA-Gly-GCC		
1	rna	2778966	2778891	-	gcgacactagctcagttggtagagcgcaaccttgccaaggttgaggtcacg		fig|127906.87.rna.100	VCCt080	VCt018		tRNA-Gly-GCC		
1	rna	2779198	2779123	-	gcgacactagctcagttggtagagcgcaaccttgccaaggttgaggtcacg		fig|127906.87.rna.101	VCCt081	Not annotated as CDS or not present		tRNA-Gly-GCC		
1	rna	2779308	2779233	-	gcgacactagctcagttggtagagcgcaaccttgccaaggttgaggtcacg		fig|127906.87.rna.102	VCCt082	Not annotated as CDS or not present		tRNA-Gly-GCC		
1	CDS	2780012	2779467	-	atgtctttcagcgaccagaatctg	MSFSDQNLIWIDLEMTGLDPE	fig|127906.87.peg.2512	VCC2512	VC0341	orn	3'-to-5' oligoribonuclease (orn)		
1	CDS	2780135	2781196	+	gtgacaaaaaagaaaaagctt	MTKKKKLTHGQVRRVRNNQH	fig|127906.87.peg.2513	VCC2513	VC0340	rsgA	Ribosome small subunit biogenesis RbfA-release protein RsgA		
1	CDS	2781292	2782149	+	atggataagattaaagttggact	MDKIKVGLQYWIPQHALTRLV	fig|127906.87.peg.2514	VCC2514	VC0339	psd	Phosphatidylserine decarboxylase (EC 4.1.1.65)		
1	CDS	2782357	2783772	+	atgccgcaactggaacgaggtg	MPQLERGLLVKLLICFLLPLGV	fig|127906.87.peg.2515	VCC2515	VC0338		Sodium-dependent anion transporter family		
1	CDS	2783924	2784811	+	atgggttacgaatggctggcact	MGYEWLALAAACLWAIASLLS	fig|127906.87.peg.2516	VCC2516	VC0337		Permease of the drug/metabolite transporter (DMT) superfamily		
1	CDS	2784954	2786486	+	atgtcagctaagaagccaatgg	MSAKKPMALVILDGWGYREI	fig|127906.87.peg.2517	VCC2517	VC0336	gpmI/yibO	2,3-bisphosphoglycerate-independent phosphoglycerate mutase (EC 5.4.2.12)		
1	CDS	2786655	2787803	+	atgacagcaacggatccgcac	MTATDPHAIFSDFLGKTLTRR	fig|127906.87.peg.2518	VCC2518	VC0335	envC	Murein hydrolase activator EnvC		
1	CDS	2788759	2787800	-	gtggattacgatgtatttaatggg	MDYDVFNGDADGILSLVQWR	fig|127906.87.peg.2519	VCC2519	VC0334		hypothetical protein	DHH family phosphoesterase [Vibrio cholerae]	
1	CDS	2789460	2788846	-	gtgcatgcggcacttgagctgttt	MHAALELFNQQGERNITTNHI	fig|127906.87.peg.2520	VCC2520	VC0333		Transcriptional regulator, AcrR family		
1	CDS	2790647	2789580	-	atgactgaattaaaaaatgatcg	MTELKNDRYLRALLKQPVDYT	fig|127906.87.peg.2521	VCC2521	VC0332	hemE	Uroporphyrinogen III decarboxylase (EC 4.1.1.37)		
1	CDS	2791678	2790902	-	gtggaaaagagtgacggcaag	MEKSDGKNAYWCVVSGSDLV	fig|127906.87.peg.2522	VCC2522	VC0331	nudC	NADH pyrophosphatase (EC 3.6.1.22), decaps 5'-NAD modified RNA		
1	CDS	2791862	2792350	+	atgctaagaaaattcaaaaacg	MLRKFKNVQEQWGGSSDIIDH	fig|127906.87.peg.2523	VCC2523	VC0330	rsd	Regulator of sigma D		
1	CDS	2796666	2792461	-	atgaaagacttattaaactttcta	MKDLLNFLKAQHKTEEFDAIKI	fig|127906.87.peg.2524	VCC2524	VC0329	rpoC	DNA-directed RNA polymerase beta' subunit (EC 2.7.7.6)		
1	CDS	2800796	2796771	-	atggtttactcttataccgagaaa	MVYSYTEKKRIRKDFGTRPQV	fig|127906.87.peg.2525	VCC2525	VC0328	rpoB	DNA-directed RNA polymerase beta subunit (EC 2.7.7.6)		
1	CDS	2801402	2801037	-	atgtctattactaacgagcaaatc	MSITNEQILDAIAEMSVMQVVE	fig|127906.87.peg.2526	VCC2526	VC0327	rplL	LSU ribosomal protein L7p/L12p (P1/P2)		
1	CDS	2801957	2801469	-	atggctttaaatcttcaagacaaa	MALNLQDKKAIVAEVNEAASG	fig|127906.87.peg.2527	VCC2527	VC0326	rplJ	LSU ribosomal protein L10p (P0)		
1	CDS	2802928	2802227	-	atggcaaaactaactaagcgta	MAKLTKRMRTIRAKVDVTKEY	fig|127906.87.peg.2528	VCC2528	VC0325	rplA	LSU ribosomal protein L1p (L10Ae)		
1	CDS	2803361	2802933	-	atggctaagaaagttgaagctta	MAKKVEAYVKLQVAAGMANP	fig|127906.87.peg.2529	VCC2529	VC0324	rplK	LSU ribosomal protein L11p (L12e)		
1	CDS	2804040	2803492	-	atgagtgaagctcccaaaaaac	MSEAPKKRWYVVQAFSGFEG	fig|127906.87.peg.2530	VCC2530	VC0323	nusG	Transcription antitermination protein NusG		
1	CDS	2804436	2804056	-	atgaaagcaaacaacgctgaa	MKANNAEAPDSSNAADTLKW	fig|127906.87.peg.2531	VCC2531	VC0322	secE	Protein translocase subunit SecE		
1	repeat	2804659	2805843	+	attaagcgatgatcttagctacaacaccagcacctacagtacggccaccttc		fig|127906.87.repeat.51				repeat region		
1	CDS	2805844	2804660	-	atgtctaaagagaaatttgaacg	MSKEKFERTKPHVNVGTIGHV	fig|127906.87.peg.2532	VCC2532	VC0321		Translation elongation factor Tu		
1	rna	2806032	2805957	-	gctgatatagctcagatggtagagcgcatccttggtaaggatgaggtcggca		fig|127906.87.rna.103	VCCt083	VCt017		tRNA-Thr-GGT		
1	rna	2806120	2806046	-	gcgggcatcgtataatggctattacctcagccttccaagctgatgatgcgggt		fig|127906.87.rna.104	VCCt084	VCt016		tRNA-Gly-TCC		
1	rna	2806240	2806156	-	ggaggggttcccgagtggccaaagggagcagactgtaaatctgccggctc		fig|127906.87.rna.105	VCCt085	VCt015		tRNA-Tyr-GTA		
1	rna	2806351	2806276	-	gccgacttagctcagtaggtagagcaactgacttgtaatcagtaggtcacca		fig|127906.87.rna.106	VCCt086	VCt014		tRNA-Thr-TGT		
1	CDS	2806356	2807483	+	gtggtgcgcattctagtgaatgat	MVRILVNDLGGCNPYLEKKLN	fig|127906.87.peg.2533	VCC2533	VC0320	coaA	Pantothenate kinase (EC 2.7.1.33)		Limited query cover for certain strains
1	CDS	2808495	2807533	-	atgaaagagcacagcgctaaa	MKEHSAKLAILKQLADGDFHS	fig|127906.87.peg.2534	VCC2534	VC0319	birA	Biotin operon repressor / Biotin--protein ligase (EC 6.3.4.9)(EC 6.3.4.10)(EC 6.3.4.11)(EC 6.3.4.15)		
1	CDS	2809565	2808492	-	atggccagtttaagttatccaaa	MASLSYPKTTMQIQLGANLKP	fig|127906.87.peg.2535	VCC2535	VC0318	murB	UDP-N-acetylenolpyruvoylglucosamine reductase (EC 1.3.1.98)		
1	CDS	2809578	2810024	+	atggccgacaagcacgaaaga	MADKHERPMLEFQQFAPIKLP	fig|127906.87.peg.2536	VCC2536	VC0317		Predicted acyltransferase		
1	CDS	2810119	2811459	+	atgattgctcgaagaaacccaa	MIARRNPIEQLPAIAQDPDKFG	fig|127906.87.peg.2537	VCC2537	VC0315	pssA	CDP-diacylglycerol--serine O-phosphatidyltransferase (EC 2.7.8.8)		
1	repeat	2811568	2817507	+	ggataaacgcaaaaagacaaagacaaaaagagcagtaagaagagttgg		fig|127906.87.repeat.52				repeat region		
1	rna	2811820	2811744	-	ggagcggtagttcagtcggttagaataccggcctgtcacgccgggggtcgc		fig|127906.87.rna.107	VCCt087	VCt013		tRNA-Asp-GTC		
1	rna	2811964	2811843	-	ttgcttggcgaccatagcgttttggacccacctgattccatcccgaactcagaa		fig|127906.87.rna.108	VCr019	VCr009		5S rRNA ## 5S ribosomal RNA		
1	rna	2814977	2812089	-	ggttaagtgactaagcgtacacggtggatgcctgggcagtcagaggcgatg		fig|127906.87.rna.109	VCr020	VCr008		LSU rRNA ## 23S rRNA, large subunit ribosomal RNA		
1	rna	2815340	2815265	-	ggggttatagctcagctgggagagcgcctgccttgcacgcaggaggtctgc		fig|127906.87.rna.110	VCCt088	VCt012		tRNA-Ala-TGC		
1	rna	2815425	2815349	-	gggtctgtagctcaggtggttagagcgtacgcctgataagcgtaaggtcggt		fig|127906.87.rna.111	VCCt089	VCt011		tRNA-Ile-GAT		
1	CDS	2817196	2816999	-	gtgtgggcactcgttgatgataat	MWALVDDNQKRFINELSDHLN	fig|127906.87.peg.2538	VCC2538	Not annotated as CDS or not present		hypothetical protein		
									VCr007				
1	CDS	2818319	2817609	-	atgataattcaatgccaagttaa	MIIQCQVKSVQPLATHTYQILL	fig|127906.87.peg.2539	VCC2539	VC0312	fre	NAD(P)H-flavin reductase (EC 1.5.1.41) (EC 1.16.1.3)		
1	CDS	2818604	2818335	-	atgacctataccgtccgcatcgt	MTYTVRIVPNDRQFIIHSGETV	fig|127906.87.peg.2540	VCC2540	VC0311	paaE	Ferredoxin		Limited query cover for certain strains
1	CDS	2820442	2818601	-	atgaattttaaggacttacgggat	MNFKDLRDFLDYLEQRGELKF	fig|127906.87.peg.2541	VCC2541	VC0309	ubiD	3-polyprenyl-4-hydroxybenzoate carboxy-lyase (EC 4.1.1.98)		
1	CDS	2821516	2820524	-	atgcaacaaggatggttgtgcct	MQQGWLCLVLLFLLGLPPYAL	fig|127906.87.peg.2542	VCC2542	VC0308		hypothetical protein		
1	CDS	2822874	2821615	-	atgaatctaacagaactgaaga	MNLTELKNTPVSDLVKLGESL	fig|127906.87.peg.2543	VCC2543	VC0307	rho	Transcription termination factor Rho		
1	CDS	2823398	2823072	-	atgagtgacaaaatttgcagctg	MSDKILQLTDDGFENDVIKAA	fig|127906.87.peg.2544	VCC2544	VC0306	trxA	Thioredoxin		

1	CDS	2823510	2824826	+	atgaaaaagacgcatatcacag	MKKTHITEHKFADFGLQPQVI	fig|127906.87.peg.2545	VCC2545	VC0305	rhlB	ATP-dependent RNA helicase RhlB (EC 3.6.4.13)		
1	CDS	2824834	2826327	+	atgagccaagccgtttcttcacc	MSQAVSSPLYAAIDLGSNSFH	fig|127906.87.peg.2546	VCC2546	VC0304	gppA	Guanosine-5'-triphosphate,3'-diphosphate pyrophosphatase (EC 3.6.1.40) @ Exopolyphosphatase (EC 3.6.1.11)		
1	CDS	2829893	2826450	-	atgcaaggtggttggtaattccc	MQGWLVIPVSLAYLGVLFLIAV	fig|127906.87.peg.2547	VCC2547	VC0303	rpfC	Two-component system sensor histidine kinase		
1	CDS	2831176	2829986	-	atgtttacccccactccctatggtt	MFTPTPYGWISQYFIGFFFAY	fig|127906.87.peg.2548	VCC2548	VC0302	hcaT	Probable 3-phenylpropionic acid transporter		
					ttgcttatatcaacagattcaccgtcaagttaccggtgccaatcggcggatcat		Only in N16961	Not annotated as CDS	VC0301				
1	CDS	2831436	2833259	+	atgcctgataaattcaatatgcag	MPDKFNMQSPPFNRLTAEQQ	fig|127906.87.peg.2549	VCC2549	VC0300		Predicted signal-transduction protein	hybrid sensor histidine kinase/response regulator [Vibrio cholerae]	
1	CDS	2833268	2833897	+	atgaactggttattacgccgttatt	MNWLLRRYWHAKLKGSPYQF	fig|127906.87.peg.2550	VCC2550	VC0299		3'-5' exonuclease domain similar to epsilon subunit of DNA polymerase III, PA3232-type		
1	CDS	2834100	2836049	+	atgagtgaagcccatatttatccg	MSEAHIYPVKQNIKAHTHADN	fig|127906.87.peg.2551	VCC2551	VC0298		Acetyl-CoA synthetase (EC 6.2.1.1)		
1	CDS	2836429	2836881	+	atgactgcaaaatctcgcattcttt	MTAKSRILVLNGPNLNLLGLRE	fig|127906.87.peg.2552	VCC2552	VC0297		3-dehydroquinate dehydratase II (EC 4.2.1.10)		
1	CDS	2836938	2837399	+	atggatatccgcaaaatcaaga	MDIRKIKKLIELVEESGIAELEIS	fig|127906.87.peg.2553	VCC2553	VC0296	accB	Biotin carboxyl carrier protein of acetyl-CoA carboxylase		
1	CDS	2837414	2838757	+	atgttagataaagttgtcatcgcg	MLDKVVIANRGEIALRILRACK	fig|127906.87.peg.2554	VCC2554	VC0295	accC	Biotin carboxylase of acetyl-CoA carboxylase (EC 6.3.4.14)		
1	CDS	2838966	2839853	+	atgccttggattcaaattaaactc	MPWIQIKLNATNDNAEAIGDM	fig|127906.87.peg.2555	VCC2555	VC0293	prmA	Ribosomal protein L11 methyltransferase		
1	CDS	2840041	2840961	+	atggcgggtgtcaccgacagac	MAGVTDRPFRELCLRYGAGM	fig|127906.87.peg.2556	VCC2556	VC0291		tRNA-dihydrouridine synthase DusB		
1	CDS	2840985	2841281	+	atgttcgaacaaaatctgacttca	MFEQNLTSEALTVTTVTSQDQ	fig|127906.87.peg.2557	VCC2557	VC0290	fis	DNA-binding protein Fis		
1	CDS	2841420	2842418	+	atgacaaaaaaacagcgcgcg	MTKKQRATLQDVADQVGVTK	fig|127906.87.peg.2558	VCC2558	VC0289	gntR	Gluconate utilization system Gnt-I transcriptional repressor		
1	CDS	2844291	2842495	-	atgattcatcctattttagccaagg	MIHPILAKVTQNLTERSREARA	fig|127906.87.peg.2559	VCC2559	VC0288	edd	Phosphogluconate dehydratase (EC 4.2.1.12)		
1	CDS	2844803	2844288	-	atggccggtagtagcatcattgtg	MAGSSIIVMGVCACGKSTIGE	fig|127906.87.peg.2560	VCC2560	VC0287	gntK/gntV	Gluconokinase (EC 2.7.1.12)		
1	CDS	2844957	2846333	+	atggacccctactacaaacctc	MDPLLQTSDPTYLLTVAAVAII	fig|127906.87.peg.2561	VCC2561	VC0286	ygbN	Low-affinity gluconate/H+ symporter GntU		
1	CDS	2846346	2846951	+	atgaccattgaacaacgcttacg	MTIEQRLRAIKIVPVIAINDVAH	fig|127906.87.peg.2562	VCC2562	VC0285	eda	4-hydroxy-2-oxoglutarate aldolase (EC 4.1.3.16) @ 2-dehydro-3-deoxyphosphogluconate aldolase (EC 4.1.2.14)		
1	CDS	2847173	2848657	+	atgtctcattttactcgctcaagaa	MSHFTRSRTWVSLAVFAACA	fig|127906.87.peg.2563	VCC2563	VC0284	fiu	Ferrichrome-iron receptor		2 ORFs is joined together as 1 ORF in N16961
1	CDS	2848724	2849215	+	atgtataaccgccgtatgcgtga	MYNRRMRDPNDQERIILTGLQ	fig|127906.87.peg.2564	VCC2564	VC0284		Ferrichrome-iron receptor		2 ORFs is joined together as 1 ORF in N16961
1	CDS	2849236	2849913	+	atgcagcggcaagtcaaaggg	MQRQVKGDNHRRAGLTASLI	fig|127906.87.peg.2565	VCC2565	VC0283		Putative transmembrane protein	Peptidase [Vibrio cholerae]	
1	CDS	2850178	2851818	+	atgacttccgttagcctgcgccac	MTSVSLRHLSIRNRLFLLTVVIL	fig|127906.87.peg.2566	VCC2566	VC0282		Methyl-accepting chemotaxis sensor/transducer protein		
1	CDS	2854071	2851936	-	atgaatattttcgctatcttgaacc	MNIFAILNHMGVFFKEEPVRQ	fig|127906.87.peg.2567	VCC2567	VC0281	cadA	Lysine decarboxylase, inducible (EC 4.1.1.18)		
1	CDS	2855518	2854181	-	atgtcatcaaatgcaaaaaaaa	MSSNAKKIGLIACTGVVAGNM	fig|127906.87.peg.2568	VCC2568	VC0280	cadB	Lysine/cadaverine antiporter membrane protein CadB		
1	CDS	2855795	2855667	-	ttgatatttttagttgtctctcctgata	MIFLVVSPDIFRFFINDMKIKRF	fig|127906.87.peg.2569	VCC2569	Not annotated as CDS		hypothetical protein		
1	CDS	2857777	2856218	-	atgattggaatctgttttcaaatca	MIGICFQINDWVLCIDENKLYR	fig|127906.87.peg.2570	VCC2570	VC0278		Transcriptional activator of cad operon		
1	CDS	2858402	2858004	-	atgtttcagattggagagttagcc	MFQIGELAKRCGVSTDTLRFY	fig|127906.87.peg.2571	VCC2571	VC0277	zntR	Transcriptional regulator ZntR, MerR family		
1	CDS	2858691	2860283	+	atgaacaacgctcgtcctattcaa	MNNARPIHRALLSVSDKTGIVE	fig|127906.87.peg.2572	VCC2572	VC0276	purH	IMP cyclohydrolase (EC 3.5.4.10) / Phosphoribosylaminoimidazolecarboxamide formyltransferase (EC 2.1.2.3)		
1	CDS	2860342	2861631	+	atgcaagttctcatcatcggctctt	MQVLIIGSGGREHALAWKVAC	fig|127906.87.peg.2573	VCC2573	VC0275	purD	Phosphoribosylamine--glycine ligase (EC 6.3.4.13)		
1	CDS	2862420	2861731	-	atgaagcagcagttatccttcctc	MKQQLSFLFLLSGLLAGCSST	fig|127906.87.peg.2574	VCC2574	VC0274		Peptidyl-prolyl cis-trans isomerase (EC 5.2.1.8)		
1	CDS	2862738	2862466	-	atgaacaagacccaattgatcg	MNKTQLIDFIAEKADLTKVQAK	fig|127906.87.peg.2575	VCC2575	VC0273	hupA	DNA-binding protein HU-alpha		
1	CDS	2863110	2864168	+	atgtttctgcttgcgatatatatctc	MFLLAIYISVAIGVSFICSVLEA	fig|127906.87.peg.2576	VCC2576	VC0271	tlyC	CBS-domain-containing protein		
1	CDS	2865403	2864516	-	atgctgattggattggatattggtg	MLIGLDIGGTKIEICVLDKQGSI	fig|127906.87.peg.2577	VCC2577	VC0270	mak	ROK family sugar kinase or transcriptional regulator		
1	CDS	2866661	2865462	-	atgaacaccgcaattccagctcc	MNTAIPAPFFIMHNVIQSYAW	fig|127906.87.peg.2578	VCC2578	VC0269		Mannose-6-phosphate isomerase (EC 5.3.1.8)		
1	CDS	2867611	2867024	-	atgttacaaaaccctctacagct	MLQNPLQLRLEKFEPWQQITF	fig|127906.87.peg.2579	VCC2579	VC0268		Uncharacterized protein YjaG	DUF416 family protein [Vibrio cholerae]	
1	CDS	2867685	2868608	+	atgaacaatttccaccagctgta	MNNFHQLYILTEHDEHYREQII	fig|127906.87.peg.2580	VCC2580	VC0267	ghrA	D-3-phosphoglycerate dehydrogenase (EC 1.1.1.95)		
1	CDS	2868608	2869180	+	atgcttgagtcactgctgacgga	MLESLLTEIRQCTVCEPHLPL	fig|127906.87.peg.2581	VCC2581	VC0266		Hypothetical protein VC0266 (sugar utilization related?)		
1	CDS	2869235	2870473	+	atgcttgaaacattaaccttgtgg	MLETLTLWLQKFDIEIERVIQL	fig|127906.87.peg.2582	VCC2582	VC0265	mscM	Miniconductance mechanosensitive channel YbdG	uracil-DNA glycosylase family protein [Vibrio cholerae]	
1	CDS	2872051	2870735	-	atggaagtggttcatcacggagg	MEVVHHGGKASVTGSCHELR	fig|127906.87.peg.2583	VCC2583	VC0264		Metallo-beta-lactamase family protein, RNA-specific		
1	CDS	2872258	2872142	-	gtgttaagccataacaatcaagc	MLSHNNQAVQLDGSIVMAFTL	fig|127906.87.peg.2584	VCC2584	No refSeq annotation		FIG01201221: hypothetical protein	lipid carrier : UDP-N-acetylgalactosaminyltransferase [Vibrio cholerae]	
1	CDS	2872799	2872245	-	atggttattcgatttcttgattttattt	MVIRFLDFIFALAGLLLLWPVLL	fig|127906.87.peg.2585	VCC2585	VC0263		Lipid carrier : UDP-N-acetylgalactosaminyltransferase (EC 2.4.1.-)		
1	CDS	2873778	2872807	-	atggaaccagataatcaaacac	MEPDNQTPLKILVTGASGFVG	fig|127906.87.peg.2586	VCC2586	VC0262		UDP-glucose 4-epimerase (EC 5.1.3.2)		
1	CDS	2873931	2875796	+	atgtttacccctattcaactgctctt	MFTPIQLLLSAQRHHKRMITLL	fig|127906.87.peg.2587	VCC2587	VC0260	pglF/rfbU	Nucleoside-diphosphate sugar epimerase/dehydratase		
1	CDS	2875804	2876916	+	atgaaagtattgcatgtatataga	MKVLHVYRTCYPETKGGVEQ	fig|127906.87.peg.2588	VCC2588	VC0259	kanE/rfbV	Glycosyltransferase		
1	CDS	2877810	2877307	-	atgaaacatctaataaaaaacta	MKHLIKNYVQKLIKTELDAIQSI	fig|127906.87.peg.2589	VCC2589	VC0258		RfbT protein		ORF split into 2 ORFs in N16961, both refseq annotated
1	CDS	2878911	2878207	-	atgcctctgcggatacgtttctct	MPSADTLSRVMGMINPVALQF	fig|127906.87.peg.2590	VCC2590	VC0257		Transposase		
1	CDS	2879115	2878918	-	atgagcgagtcaatcaacccat	MSESINPFMHFQIIKDYRQESH	fig|127906.87.peg.2591	VCC2591	VC0256		Transposase		
1	repeat	2879289	2879480	+	ggtgtccaaacgatatgatatttgcaacgccaatagacgtgtgatgaacttctc		fig|127906.87.repeat.53				repeat region		
1	CDS	2880069	2879503	-	atgagcggtaacggttactacag	MSGNGYYSEDVLKQMGFSSL	fig|127906.87.peg.2592	VCC2592	VC0252	wbpD	dTDP-4-amino-4,6-dideoxy-D-glucose acyltransferase (EC 2.3.1.209)		
1	CDS	2882616	2880139	-	atgcctgtaaataacgaaaatct	MPVNNENLTSVLDARPFELSE	fig|127906.87.peg.2593	VCC2593	VC0251		Acyl protein synthase/acyl-CoA reductase RfbN		
1	CDS	2883731	2882607	-	atgctaaaactacgtttcccacta	MLKLRFPLPVFRNAREIANGR	fig|127906.87.peg.2594	VCC2594	VC0250		Alcohol dehydrogenase (EC 1.1.1.1); Acetaldehyde dehydrogenase (EC 1.2.1.10)		
1	CDS	2885155	2883740	-	atgtctgtggttgagagaatattta	MSVVERIFNHSESRPDCIAVY	fig|127906.87.peg.2595	VCC2595	VC0249	lcfB/rfbL	Long-chain-fatty-acid--CoA ligase (EC 6.2.1.3)		
1	CDS	2885394	2885161	-	atgtctatctctgaagaaaaaatt	MSISEEKIINLIAGILEVEIGIINK	fig|127906.87.peg.2596	VCC2596	VC0248		hypothetical protein	Acyl carrier protein [Vibrio cholerae]	
1	CDS	2886194	2885442	-	atgatagagctatctaatgttaat	MIELSNVNLHYPVPGHFSHSL	fig|127906.87.peg.2597	VCC2597	VC0247	tagH/rfbI	O-antigen export system, ATP-binding protein		
1	CDS	2886969	2886196	-	atgcttaaagatatagtttcggttt	MLKDIVSVFYNWRIVHLLGVS	fig|127906.87.peg.2598	VCC2598	VC0246	rfbH	O-antigen export system, permease protein		
1	CDS	2888344	2886962	-	atgacagaaaaaaagaagcta	MTEKKKLVVWVPLPPNSSWR	fig|127906.87.peg.2599	VCC2599	VC0245	rfbG	O-antigen export system, polymerase or transferase		
1	CDS	2889453	2888350	-	atgattcctgtatacgaaccaag	MIPVYEPSLDGNERKYLNDCI	fig|127906.87.peg.2600	VCC2600	VC0244	rfbE	GDP-perosamine synthase (EC 2.6.1.102)		
1	CDS	2890585	2889464	-	atgaataaaaaagttgcgttaat	MNKKVALITGITGQDGSYLAEI	fig|127906.87.peg.2601	VCC2601	VC0243	gmd	GDP-mannose 4,6-dehydratase (EC 4.2.1.47)		
1	CDS	2891979	2890588	-	gtgaaagagttaacttgttttaaa	MKELTCFKAYDIRGQLGSELD	fig|127906.87.peg.2602	VCC2602	VC0242	algC/rfbB	Phosphomannomutase (EC 5.4.2.8)		
1	CDS	2893379	2891982	-	atgtttatacctgtaattatggctgg	MFIPVIMAGGSGSRLWPLSRS	fig|127906.87.peg.2603	VCC2603	VC0241	rfbA	Mannose-1-phosphate guanylyltransferase (EC 2.7.7.13) / Mannose-6-phosphate isomerase (EC 5.3.1.8)		
1	CDS	2893821	2894765	+	atgattatcgtaactggcggcgc	MIIVTGGAGMIGSNIIKALNER	fig|127906.87.peg.2604	VCC2604	VC0240	hldD/rfaD	ADP-L-glycero-D-manno-heptose-6-epimerase (EC 5.1.3.20)		
1	CDS	2894924	2896696	+	atgaatattttgatggccctatcc	MNILMALSQLEVTGAEVYATS	fig|127906.87.peg.2605	VCC2605	VC0239	xynD	Polysaccharide deacetylase		
1	CDS	2896710	2897270	+	atggctggctttggtttaattgcaa	MAGFGLIAKLRNKIRVKPNNKI	fig|127906.87.peg.2606	VCC2606	VC0238		transferase, hexapeptide repeat family		
1	CDS	2897272	2898471	+	atgaataataaaataactaaaa	MNNKITKTSIFLTISLLLITPGFS	fig|127906.87.peg.2607	VCC2607	VC0237		O-antigen ligase		
1	CDS	2898471	2899508	+	atgaaaattcttgttattggcccttc	MKILVIGPSWVGDMVMSQSLY	fig|127906.87.peg.2608	VCC2608	VC0236	rfaF	ADP-heptose--lipooligosaccharide heptosyltransferase II		
1	CDS	2899505	2900554	+	atgaaacacctctggtttgaaaa	MKHLWFEKGAYASKAARQYL	fig|127906.87.peg.2609	VCC2609	VC0235		lipopolysaccharide biosynthesis protein, putative		
1	CDS	2900557	2901339	+	atgagcaaagtaaccgtattgat	MSKVTVLIANRLIRLTGNLFKM	fig|127906.87.peg.2610	VCC2610	VC0234		Mannosyltransferase OCH1 and related enzymes		
1	CDS	2901339	2902619	+	atgctgctccgtacgctttatacg	MLLRTLYTLLLTLAAPFLLYGL	fig|127906.87.peg.2611	VCC2611	VC0233	waaA/kdtA	3-deoxy-D-manno-octulosonic acid transferase (EC 2.4.99.12)(EC 2.4.99.13)		
1	CDS	2902616	2902996	+	atgaaaaaattgatcgtcgacct	MKKLIVDLDGTLTQANTSDYR	fig|127906.87.peg.2612	VCC2612	VC0232		Capsular polysaccharide biosynthesis protein		
1	CDS	2902987	2904570	+	atgttcctaattatgtctggtgccta	MFLIMSGAYVGQELESEFGRI	fig|127906.87.peg.2613	VCC2613	VC0231		Capsular polysaccharide biosynthesis protein		
1	CDS	2904567	2905310	+	atgattgtaatcccaatggcggg	MIVIPMAGMSSRFFKAGYTQP	fig|127906.87.peg.2614	VCC2614	VC0230		Nucleoside-diphosphate-sugar pyrophosphorylase involved in lipopolysaccharide biosynthesis/translation initiation factor 2B, gamma/epsilon subunits (eIF-2Bgamma/eIF-2Bepsilon)		
1	CDS	2905307	2906236	+	atgatcaagattgatgatgaagc	MIKIDDEAITFVVQGPVQASAS	fig|127906.87.peg.2615	VCC2615	VC0229		Lipopolysaccharide synthesis protein WavE		
1	CDS	2907199	2906240	-	atgctattcgatcagattacattcc	MLFDQITFVIQGPITPSISTSTSV	fig|127906.87.peg.2616	VCC2616	VC0228		hypothetical protein	WavE lipopolysaccharide synthesis family protein [Vibrio cholerae]	
1	CDS	2907910	2907203	-	ttgatacagacgttcaacgacgc	MIQTFNDAQQKVWFDDALLHE	fig|127906.87.peg.2617	VCC2617	VC0227	kdkA	3-deoxy-D-manno-octulosonic acid kinase (EC 2.7.1.166)		
1	CDS	2908044	2909111	+	atggcattgttttcaatcagcccca	MALFQSAPHSLCILRLSAIGDV	fig|127906.87.peg.2618	VCC2618	VC0225	rfaQ	Lipopolysaccharide core heptosyltransferase I		
1	CDS	2909154	2909933	+	gtgagtaaacccaacgcttgctgt	MSKPTLAVALIVKNEARHLDE	fig|127906.87.peg.2619	VCC2619	VC0224	sunS	Lipopolysaccharide biosynthesis glycosyltransferase		
1	CDS	2911032	2909995	-	atgaaaaagttactggttattcgc	MKKLLVIRNDKIGDFMLAWPS	fig|127906.87.peg.2620	VCC2620	VC0223		ADP-heptose--lipooligosaccharide heptosyltransferase II		
1	CDS	2911143	2911637	+	gtgagccaaaaacgtctttcccg	MSQKRLSRVIYPGTFDPITNGI	fig|127906.87.peg.2621	VCC2621	VC0222	coaD	Phosphopantetheine adenylyltransferase (EC 2.7.7.3)		
1	CDS	2912543	2911734	-	atgcctgaattacctgaagttgaa	MPELPEVEVSRLGISPHLVGG	fig|127906.87.peg.2622	VCC2622	VC0221	mutM	Formamidopyrimidine-DNA glycosylase (EC 3.2.2.23)		
1	CDS	2913035	2912553	-	atgcgcgtttctcccaatcgacgt	MRVSPNRRRRWNNILILSVIAF	fig|127906.87.peg.2623	VCC2623	VC0220		hypothetical protein		
1	CDS	2913429	2913262	-	atggctaaaggcattcgcgaaa	MAKGIREKIRLVSSAGTGHFY	fig|127906.87.peg.2624	VCC2624	VC0219	rpmG	LSU ribosomal protein L33p @ LSU ribosomal protein L33p, zinc-independent		
1	CDS	2913679	2913443	-	atgtcacgagtatgccaagtaac	MSRVCQVTGKRPAVGNNRSH	fig|127906.87.peg.2625	VCC2625	VC0218	rpmB	LSU ribosomal protein L28p @ LSU ribosomal protein L28p, zinc-independent		
1	CDS	2913682	2913816	+	atgtcttctccaaatcgtttcagct	MSSPNRFSSISTLVAEPLSFQI	fig|127906.87.peg.2626	VCC2626	Not annotated as CDS		conserved domain protein		
1	CDS	2914648	2913974	-	atgagcctaaaacagttaccca	MSLKQLPTESMPREKLLQRGI	fig|127906.87.peg.2627	VCC2627	VC0217	radC	UPF0758 family protein	DNA repair protein RadC [Vibrio cholerae]	
1	CDS	2916439	2914760	-	atgctgtcttctctacgaagccgt	MLSSLRSRTKLLLLTVIPLIVITA	fig|127906.87.peg.2628	VCC2628	VC0216		Methyl-accepting chemotaxis sensor/transducer protein		
1	CDS	2916622	2917821	+	atgcaatcattggcaggcaaaa	MQSLAGKKILLGISGGIAAYKC	fig|127906.87.peg.2629	VCC2629	VC0215	coaBC	Phosphopantothenoylcysteine decarboxylase (EC 4.1.1.36) / Phosphopantothenoylcysteine synthetase (EC 6.3.2.5)		
1	CDS	2917980	2918570	+	atggccggcaataaaaaaatca	MAGNKKINRREEILQALAEMLI	fig|127906.87.peg.2630	VCC2630	VC0214	slmA	Transcriptional regulator SlmA, TetR family		
1	CDS	2918576	2919523	+	atgagtcaagataaatacagca	MSQDKYSKPEFSFSLLHPKYV	fig|127906.87.peg.2631	VCC2631	VC0213	htrB	Lipid A biosynthesis lauroyl acyltransferase (EC 2.3.1.241)		
1	CDS	2919534	2920511	+	gtgagcgatcaagacaacggc	MSDQDNGIARHLHNPQFEWR	fig|127906.87.peg.2632	VCC2632	VC0212	msbB	Lipid A biosynthesis myristoyltransferase (EC 2.3.1.243)		
1	CDS	2921241	2920597	-	atgatgaaagcttaccagcgtga	MMKAYQREFIEFALEKQVLKF	fig|127906.87.peg.2633	VCC2633	VC0211	pyrE	Orotate phosphoribosyltransferase (EC 2.4.2.10)		
1	CDS	2922320	2921604	-	atgcgtccagataaccgcgctg	MRPDNRAADQVRPIKITRHYT	fig|127906.87.peg.2634	VCC2634	VC0210	rph	Ribonuclease PH (EC 2.7.7.56)		
1	CDS	2922484	2923350	+	atgatttacagcatgactgcgtac	MIYSMTAYARKEVKGDWGSA	fig|127906.87.peg.2635	VCC2635	VC0209		UPF0701 protein YicC	protein YicC [Vibrio cholerae MJ-1236]	
1	CDS	2923416	2923904	+	atggaacgtttgatcgaaaaact	MERLIEKLLYSSRWIMAPIYLG	fig|127906.87.peg.2636	VCC2636	VC0208		Putative inner membrane protein (Fragment)		
1	CDS	2925456	2924011	-	atggccaaaataacccaaacc	MAKITQTMMAQVLSLVGGSGI	fig|127906.87.peg.2637	VCC2637	VC0207	murP	PTS system, N-acetylmuramic acid-specific IIB component (EC 2.7.1.192) / PTS system, N-acetylmuramic acid-specific IIC component		
1	CDS	2926389	2925487	-	atgaagattgatttaacccgctta	MKIDLTRLVTESRNPASEQIDT	fig|127906.87.peg.2638	VCC2638	VC0206	murQ	N-acetylmuramic acid 6-phosphate etherase (EC 4.2.1.126)		
					atgcctgccacgcttcatgcttttcaatcaatggtttcatggttgagcgtttattttt		Only in N16961	Not annotated as CDS	VC0205		hypothetical protein		
1	CDS	2926620	2927468	+	atgtccgtactacaacgcatcgt	MSVLQRIVSRRTQLSESGRQI	fig|127906.87.peg.2639	VCC2639	VC0204	murR	Transcriptional regulator, RpiR family		
1	CDS	2929456	2927495	-	atgagtgtgctgcgtttaactggt	MSVLRLTGLGALTLLLALVSLC	fig|127906.87.peg.2640	VCC2640	VC0203	fhuB	Ferrichrome transport system permease protein FhuB		
1	CDS	2930412	2929453	-	atgtgcttgatgagtctatggaat	MCLMSLWNVRLRNICRPKAVI	fig|127906.87.peg.2641	VCC2641	VC0202	fhuD	Ferrichrome-binding periplasmic protein precursor (TC 3.A.1.14.3)		
1	CDS	2931188	2930403	-	atgtttcagttggctgatattcaaa	MFQLADIQMVRGGRTILAIDHL	fig|127906.87.peg.2642	VCC2642	VC0201	fhuC	Ferrichrome transport ATP-binding protein FhuC		
1	CDS	2933418	2931319	-	atgcgcaatattcgcccgagtca	MRNIRPSQLGQYIRRALRLHF	fig|127906.87.peg.2643	VCC2643	VC0200	fhuA	Ferrichrome-iron receptor		
1	CDS	2933553	2935382	+	atgttccaatggtttgaaaaactg	MFQWFEKLTEPFPAQEPQTP	fig|127906.87.peg.2644	VCC2644	VC0199		Efflux ABC transporter, permease/ATP-binding protein Atu2242		
1	CDS	2935780	2935418	-	atgcgtttagagtcgattcatcaa	MRLESIHQEVISFERFVWRCLI	fig|127906.87.peg.2645	VCC2645	VC0198		putative membrane protein		
1	CDS	2936250	2935951	-	atggctgagttcggtttaggcgac	MAEFGLGDYLAAEGRLIVRAE	fig|127906.87.peg.2646	VCC2646	VC0197		Aminopeptidase N		
1	CDS	2938105	2936243	-	atgactcttactttgcactctatcg	MTLTLHSIDMTATLMAESSALF	fig|127906.87.peg.2647	VCC2647	VC0196	recQ	ATP-dependent DNA helicase RecQ		
1	CDS	2938245	2939147	+	atgacacccgaccaacaagat	MTPDQQDAKKGILLAISAYTM	fig|127906.87.peg.2648	VCC2648	VC0195	rarD	Uncharacterized inner membrane protein RarD	EamA family transporter RarD [Vibrio cholerae]	ORF split into 2 ORFs in N16961. Annotated as 1 ORF in KEGG for both N16961
									VC0195				ORF split into 2 ORFs in N16961. Annotated as 1 ORF in KEGG for both N16961
1	CDS	2939321	2941075	+	atgaattggactcatacccccttg	MNWTHTPLALSALMFAMAAQ	fig|127906.87.peg.2649	VCC2649	VC0194	ggt	Gamma-glutamyltranspeptidase (EC 2.3.2.2) @ Glutathione hydrolase (EC 3.4.19.13)		
1	CDS	2941232	2942035	+	atggatacagtgaattatcgccc	MDTVNYRPSAYPAISLIEADYR	fig|127906.87.peg.2650	VCC2650	VC0192	rhaS	Transcriptional regulator, AraC family		
1	CDS	2942110	2942778	+	atgaacgaaatgtccattttagcc	MNEMSILATLAGVHFIALLSPG	fig|127906.87.peg.2651	VCC2651	VC0191	rhtC	Threonine efflux protein		
1	CDS	2945694	2943523	-	atgatcgatccctctctattgctcg	MIDPSLLLDGLNDKQREAVAA	fig|127906.87.peg.2652	VCC2652	VC0190	uvrD	ATP-dependent DNA helicase UvrD/PcrA (EC 3.6.4.12)		
1	CDS	2947874	2945832	-	atgtcaaacccacttcttaccttta	MSNPLLTFTDLPPFSAIKPEHV	fig|127906.87.peg.2653	VCC2653	VC0188	prlC	Oligopeptidase A (EC 3.4.24.70)		
1	CDS	2948154	2948993	+	ttgttaagttaccgacacagtttcc	MLSYRHSFHAGNHADVLKHIV	fig|127906.87.peg.2654	VCC2654	VC0187	rlmJ	23S rRNA (adenine(2030)-N(6))-methyltransferase (EC 2.1.1.266)		
1	CDS	2949099	2950451	+	atggcaacacattttgactacctg	MATHFDYLCIGGGSGGIASAN	fig|127906.87.peg.2655	VCC2655	VC0186	gor	Glutathione reductase (EC 1.8.1.7)		
1	CDS	2950540	2950938	+	atggttactgtcgtaaattcgaac	MVTVVNSNNFDVPRIYPPSSK	fig|127906.87.peg.2656	VCC2656	VC0185		Transposase		ORF is split up by transposase in C6706?
1	repeat	2950903	2952167	+	gtcatctgtagtggcatagtgaatttggtcacttagttagaggtgatatcatcacc		fig|127906.87.repeat.54				repeat region		
1	CDS	2950981	2951289	+	atgacaaaacgtacaagacga	MTKRTRRLFSAEFKLEAAQLV	fig|127906.87.peg.2657	VCC2657	Not present	orfA	Transposase InsN for insertion sequence element IS911		
1	CDS	2951286	2952158	+	ttgataatcaagaaactcaagca	MIIKKLKQSHSVKILCEVFNVHI	fig|127906.87.peg.2658	VCC2658	Not present	orfB	Transposase InsO for insertion sequence element IS911		
1	CDS	2952155	2953003	+	ttgaccactacaatcaaattctat	MTTTIKFYEWAAHERFITFTEE	fig|127906.87.peg.2659	VCC2659	VC0185		Transposase		ORF is split up by transposase in C6706?
1	CDS	2953000	2954688	+	atgacttacacgcctcctaagcta	MTYTPPKLTIKLRTGIKQTFTYI	fig|127906.87.peg.2660	VCC2660	VC0184		hypothetical protein		
1	CDS	2954698	2955282	+	atgacaccattgtttgaactgcct	MTPLFELPKNILKENLSEYKNI	fig|127906.87.peg.2661	VCC2661	VC0183		Phage integrase		Limited subject cover for certain strains. ORF split up in C6706
1	CDS	2955336	2956808	+	atggaaaagatataccgatattg	MEKIYRYCFDVVETYHPYKEE	fig|127906.87.peg.2662	VCC2662	VC0183		Phage integrase		Limited subject cover for certain strains. ORF split up in C6706
1	CDS	2956780	2957211	+	atgagcaaatcccaaattacgaa	MSKSQITKVELEQALGRILSGK	fig|127906.87.peg.2663	VCC2663	VC0182		hypothetical protein		Limited subject cover for certain strains
1	CDS	2957692	2957222	-	gtgatgagtgatgtcgaacttgtt	MMSDVELVFKDESDCLVVIMC	fig|127906.87.peg.2664	VCC2664	VC0181		hypothetical protein		
1	CDS	2959436	2957682	-	gtgaagcaggaattacatcatac	MKQELHHTLLGCGFRYTPAKC	fig|127906.87.peg.2665	VCC2665	VC0180		hypothetical protein		
1	CDS	2960743	2959439	-	atgacttggaactttcaccagtac	MTWNFHQYYTNRNDGLMGKL	fig|127906.87.peg.2666	VCC2666	VC0179		hypothetical protein		
1	CDS	2961830	2960763	-	atgccaaatccacctgaatatga	MPNPPEYEHLKNQVRILSLNG	fig|127906.87.peg.2667	VCC2667	VC0178		patatin-related protein		
1	CDS	2962327	2962079	-	atgcctaaaaatatcaaacaga	MPKNIKQTSSDVASLASHILKN	fig|127906.87.peg.2668	VCC2668	Not annotated as CDS		no significant database match found		
1	CDS	2963295	2962744	-	atgaagatcagtgcagagatgt	MKISAEMYKLLIERTLDGFSVIE	fig|127906.87.peg.2669	VCC2669	VC0177		Response regulator		
1	CDS	2963410	2963718	+	gtgtcattccaaaacccaatccc	MSFQNPIPLRLKEARKKAKLSC	fig|127906.87.peg.2670	VCC2670	VC0176		Transcriptional regulator		
1	CDS	2963987	2964160	+	atgattacaagcattcatgaatat	MITSIHEYRNASNSDKNRLSHI	fig|127906.87.peg.2671	VCC2671	Not annotated as CDS		hypothetical protein		
1	CDS	2964236	2965825	+	atgaataagtcctccgcaaaaa	MNKSSAKKILSTVPSPTKSNS	fig|127906.87.peg.2672	VCC2672	VC0175		deoxycytidylate deaminase-related protein		
1	CDS	2967025	2966078	-	atgccgaaatggattctatgctg	MPKWILCCNLSVALLLFAPIAS	fig|127906.87.peg.2673	VCC2673	VC0174		hypothetical protein		
1	CDS	2968138	2967200	-	atgagtgcagtatcgatcgctcc	MSAVSIAPSRWERFKQSDFLY	fig|127906.87.peg.2674	VCC2674	VC0173		Dipeptide ABC transporter, permease protein DppC (TC 3.A.1.5.2)		
1	CDS	2969118	2968141	-	atgttttcgtttctggtcaagcgcc	MFSFLVKRLYQALIVMFVISLV	fig|127906.87.peg.2675	VCC2675	VC0172		ABC transporter, permease protein 1 (cluster 5, nickel/peptides/opines)		
1	CDS	2970829	2969273	-	atgaaaaccatgaaaaacaaa	MKTMKNKLALALIAAGLSFGAI	fig|127906.87.peg.2676	VCC2676	VC0171		ABC transporter, substrate-binding protein (cluster 5, nickel/peptides/opines)		
1	CDS	2972636	2970921	-	atgtcacttttagaagtcaaaaat	MSLLEVKNLRIEYPSRHGVHA	fig|127906.87.peg.2677	VCC2677	VC0170		Dipeptide ABC transporter, ATP-binding protein DppD (TC 3.A.1.5.2)		
1	CDS	2973137	2972928	-	atgaactatttttatgatttatctga	MNYFYDLSELNWEFFGFYDW	fig|127906.87.peg.2678	VCC2678	Not annotated as CDS		FIG01205129: hypothetical protein		Limited query and subject cover for certain strains
1	rna	2973448	2973372	-	cggtgaatagcgcagtttggtagcgcatctggtttgggaccagagggtcggg		fig|127906.87.rna.112	VCCt090	VCt010		tRNA-Pro-TGG		
1	rna	2973585	2973510	-	gtggctatagctcagttggtagagccccggattgtgattccggttgtcgtgggtt		fig|127906.87.rna.113	VCCt091	VCt009		tRNA-His-GTG		
1	rna	2973732	2973656	-	cggtgaatagcgcagtttggtagcgcatctggtttgggaccagagggtcggg		fig|127906.87.rna.114	VCCt092	VCt008		tRNA-Pro-TGG		
1	rna	2973869	2973794	-	gtggctatagctcagttggtagagccccggattgtgattccggttgtcgtgggtt		fig|127906.87.rna.115	VCCt093	VCt007		tRNA-His-GTG		
1	rna	2973981	2973905	-	gcgcccgtagctcagctggatagagcgttggcctccggagccaaaggtcga		fig|127906.87.rna.116	VCCt094	VCt006		tRNA-Arg-CCG		
					ttggcaaatctttcacacaaaagcaacaaagagaggataaaaaagcgcct		Only in N16961	Not annotated as CDS	VC0169				
1	CDS	2974271	2974678	+	atggatatgtctcgatcactcttaa	MDMSRSLLSVLFAALTFSTAA	fig|127906.87.peg.2679	VCC2679	VC0168		Cytochrome c5		
1	CDS	2976760	2974745	-	atgaagctgaacccaagacaa	MKLNPRQDEAVKYVSGPCLVI	fig|127906.87.peg.2680	VCC2680	VC0167	rep	ATP-dependent DNA helicase Rep		
1	CDS	2976921	2977502	+	atgtctgaaatagtgtacggcga	MSEIVYGDKQLQILIAAEKLIAE	fig|127906.87.peg.2681	VCC2681	VC0166		Transcriptional regulator, AcrR family		
1	CDS	2977521	2978627	+	atgaaaaagtggacatttttatg	MKKWTFFMLLIAILLFGSVIGFI	fig|127906.87.peg.2682	VCC2682	VC0165		CzcABC family efflux RND transporter, membrane fusion protein		
1	CDS	2978639	2981749	+	atgcgctttactgatgtttttataaa	MRFTDVFIKRPVLAVSISFLIVL	fig|127906.87.peg.2683	VCC2683	VC0164		RND efflux system, inner membrane transporter		
1	CDS	2981865	2982116	+	atgtttgatgccaaaaaactcga	MFDAKKLEQIAKQIHEAMPQP	fig|127906.87.peg.2684	VCC2684	VC0163		Uncharacterized protein YqiC		
1	CDS	2983727	2982243	-	atggcgaattatttcaatacgctg	MANYFNTLNLREQLDQLGRC	fig|127906.87.peg.2685	VCC2685	VC0162	ilvC	Ketol-acid reductoisomerase (NADP(+)) (EC 1.1.1.86)		
1	CDS	2983865	2984761	+	gtgaacatcaaaactctgcagc	MNIKTLQLFMHLCDCKNFSKT	fig|127906.87.peg.2686	VCC2686	VC0161	ilvY	Transcriptional regulator IlvY, LysR family		
1	repeat	2984835	2984963	+	aaaaggccgctgagttgtcagcggcctttcttaatagtggcggagagatagg		fig|127906.87.repeat.55				repeat region		
1	rna	2984962	2984872	-	ggagagatggctgagtggttgaaagcaccggtcttgaaaaccggcgatggt		fig|127906.87.rna.117	VCCt095	VCt005		tRNA-Ser-TGA		
1	repeat	2985003	2990719	+	attaaaagcctggcgatgttctactctcacatggggagaccccacactacca		fig|127906.87.repeat.56				repeat region		
1	rna	2985128	2985007	-	ttgcttggcgaccatagcgttttggacccacctgattccatcccgaactcagaa		fig|127906.87.rna.118	VCr021	VCr006		5S rRNA ## 5S ribosomal RNA		
1	rna	2988141	2985253	-	ggttaagtgactaagcgtacacggtggatgcctgggcagtcagaggcgatg		fig|127906.87.rna.119	VCr022	VCr005		LSU rRNA ## 23S rRNA, large subunit ribosomal RNA		
1	rna	2988559	2988484	-	gggcgattagctcagttgggagagcacctgccttacaagcagggggtcact		fig|127906.87.rna.120	VCCt095	Not present		tRNA-Val-TAC		
1	rna	2988664	2988590	-	gggtcgttagctcagtggtagagcagttggcttttaaccaattggtcataggttc		fig|127906.87.rna.121	VCCt096	Not present		tRNA-Lys-TTT		
1	rna	2988759	2988684	-	gtcccgttcgtctagaggcctaggacaccgccctttcacggcggtaacaggg		fig|127906.87.rna.122	VCCt097	VCt005		tRNA-Glu-TTC		
1	rna	2990373	2988826	-	ttttaattgaagagtttgatcatggctcagattgaacgctggcggcaggcctaa		fig|127906.87.rna.123	VCr023	VCr004		SSU rRNA ## 16S rRNA, small subunit ribosomal RNA		
1	CDS	2990517	2990398	-	gtgtgggcactcgttgatgataat	MWALVDDNQKRFINELSDHLI	fig|127906.87.peg.2687	VCC2687	VC0160		hypothetical protein		

1	CDS	2990670	2990530	-	gtgattcagttcggtgttttaggtg	MIQFGVLGGEIANENKLKKVF	fig|127906.87.peg.2688	VCC2688	VC2753		hypothetical protein		
1	CDS	2991342	2990818	-	gtgaaagcgttactgttgtactca	MKALLLYSSQEGQTRKIIERIA	fig|127906.87.peg.2689	VCC2689	VC2755		Protoporphyrinogen IX oxidase, oxygen-independent, HemG		
1	CDS	2992822	2991365	-	atgcaatttcgttcaattatccgta	MQFRSIIRIVGLLLALFSVSML	fig|127906.87.peg.2690	VCC2690	VC2756		Trk potassium uptake system protein TrkH		
1	CDS	2993474	2992842	-	atgaatcttcatccctacctcattc	MNLHPYLIPMASVRFEEEIKKS	fig|127906.87.peg.2691	VCC2691	VC2757	ygiZ	FIG000605: protein co-occurring with transport systems (COG1739)		
1	CDS	2993685	2995856	+	atgatttaccaagccaaaaccc	MIYQAKTLQVKQLANGIAELSF	fig|127906.87.peg.2692	VCC2692	VC2758	fadB	Enoyl-CoA hydratase (EC 4.2.1.17) / Delta(3)-cis-delta(2)-trans-enoyl-CoA isomerase (EC 5.3.3.8) / 3-hydroxyacyl-CoA dehydrogenase (EC 1.1.1.35) / 3-hydroxybutyryl-CoA epimerase (EC 5.1.2.3)		
1	CDS	2995882	2997045	+	atgaatacagtagtgattgttgat	MNTVVIVDCLRTPMGRSKGG	fig|127906.87.peg.2693	VCC2693	VC2759	fadA	3-ketoacyl-CoA thiolase (EC 2.3.1.16)		
1	CDS	2998017	2997112	-	atgttttccaaatcctctttggaga	MFSKSSLEMLDAVARLGSFTA	fig|127906.87.peg.2694	VCC2694	VC2760		LysR-family transcriptional regulator YdhB		
1	CDS	2998149	2999378	+	gtgaataattttgatggacatgaa	MNNFDGHEKMKVSFFQLVYL	fig|127906.87.peg.2695	VCC2695	VC2761		Multidrug resistance transporter, Bcr/CflA family		
1	CDS	3000801	2999440	-	atgaaattcagtacggtaattctc	MKFSTVILAAGKGTRMHSNMF	fig|127906.87.peg.2696	VCC2696	VC2762	glmU	N-acetylglucosamine-1-phosphate uridyltransferase (EC 2.7.7.23) / Glucosamine-1-phosphate N-acetyltransferase (EC 2.3.1.157)		
1	CDS	3001470	3001048	-	atggcagcaataacctttcatctc	MAAITFHLDVVSAEKKLFSGLV	fig|127906.87.peg.2697	VCC2697	VC2763	atpC	ATP synthase epsilon chain (EC 3.6.3.14)		
1	CDS	3002892	3001489	-	atggctacaggtaagatcgtaca	MATGKIVQIIGAVVDVEFPQSE	fig|127906.87.peg.2698	VCC2698	VC2764	atpD	ATP synthase beta chain (EC 3.6.3.14)		
1	CDS	3003793	3002927	-	atggccggcgcaaaagagata	MAGAKEIRTKIGSVKSTQKITK	fig|127906.87.peg.2699	VCC2699	VC2765	atpG	ATP synthase gamma chain (EC 3.6.3.14)		
1	CDS	3005379	3003838	-	atgcaacttaattccacggaaat	MQLNSTEISDLIKQRIESFNVV	fig|127906.87.peg.2700	VCC2700	VC2766	atpA	ATP synthase alpha chain (EC 3.6.3.14)		
1	CDS	3005926	3005393	-	atgtcggatttgactacaatcgca	MSDLTTIARPYAKAAFDFAVEK	fig|127906.87.peg.2701	VCC2701	VC2767	atpH	ATP synthase delta chain (EC 3.6.3.14)		
1	CDS	3006405	3005941	-	atgaacgcaactctgctaggtca	MNATLLGQAISFGMFVWFCMI	fig|127906.87.peg.2702	VCC2702	VC2768	atpF	ATP synthase F0 sector subunit b (EC 3.6.3.14)		
1	CDS	3006742	3006485	-	atggaaactgtattgagcttctctc	METVLSFSAIAVAIIVGLCAVGT	fig|127906.87.peg.2703	VCC2703	VC2769	atpE	ATP synthase F0 sector subunit c (EC 3.6.3.14)		
1	CDS	3007598	3006786	-	atggctgcgccaggtgaagcgc	MAAPGEALTPSSYITHHLTNLS	fig|127906.87.peg.2704	VCC2704	VC2770	atpB	ATP synthase F0 sector subunit a (EC 3.6.3.14)		
1	CDS	3007996	3007607	-	atggtagctgtgctagcgaggca	MVAVLARQGRELAKRLLLIQFS	fig|127906.87.peg.2705	VCC2705	VC2771	atpI	ATP synthase protein I		Limited query cover for certain strains
1	CDS	3009067	3008186	-	atgactaaacgtggtttaggaaa	MTKRGLGKGLDALLATSSLAR	fig|127906.87.peg.2706	VCC2706	VC2772	parB	Chromosome (plasmid) partitioning protein ParB		
1	CDS	3009867	3009094	-	gtgggtaaaatcgtagcaatcgc	MGKIVAIANQKGGVGKTTTCIN	fig|127906.87.peg.2707	VCC2707	VC2773	parA	Chromosome (plasmid) partitioning protein ParA		
1	CDS	3010514	3009882	-	atgaatccgttacgagtcaaact	MNPLRVKLDALISKTSLTVTEC	fig|127906.87.peg.2708	VCC2708	VC2774	rsmG/gidB	16S rRNA (guanine(527)-N(7))-methyltransferase (EC 2.1.1.170)		
1	CDS	3012409	3010514	-	atgctttatcacgaaacatttgac	MLYHETFDVIVVGGGHAGTEA	fig|127906.87.peg.2709	VCC2709	VC2775	mnmG/gidA	tRNA-5-carboxymethylaminomethyl-2-thiouridine(34) synthesis protein MnmG		
1	CDS	3013318	3012884	-	atgattcatatcattactggcagc	MIHIITGSTLGGAEYVGDHLSD	fig|127906.87.peg.2710	VCC2710	VC0002	mioC	Flavoprotein MioC		
1	CDS	3014689	3013328	-	atgacaacagataccattgtcgc	MTTDTIVAQATAPGRGGVGIIR	fig|127906.87.peg.2711	VCC2711	VC0003	mnmE/trmE	tRNA-5-carboxymethylaminomethyl-2-thiouridine(34) synthesis protein MnmE		
1	CDS	3016408	3014783	-	atggattctcaacgtaatatcttgt	MDSQRNILLIALALVSFLLFQQ	fig|127906.87.peg.2712	VCC2712	VC0004	yidC	Inner membrane protein translocase and chaperone YidC, long form		
					atggcaacgcctctctcgcccttctcgtggttagccattggtattgtaaaacttta		From N16961	Not annotated as CDS	VC0005				
1	CDS	3016958	3016635	-	ttgttaactcccgaacattatcaa	MLTPEHYQKVFQQAHSAGSP	fig|127906.87.peg.2713	VCC2713	VC0006	rnpA	Ribonuclease P protein component (EC 3.1.26.5)		
1	CDS	3017141	3017004	-	atgtctaaacgcacttttcaacct	MSKRTFQPSVLKRKRTHGFR	fig|127906.87.peg.2714	VCC2714	VC0007	rpmH	LSU ribosomal protein L34p		
1	CDS	3018120	3017383	-	atgattaaattgcagaatatccac	MIKLQNIHKRFGTSEVLKGIDL	fig|127906.87.peg.2715	VCC2715	VC0008	yxeO	Probable amino-acid import ATP-binding protein YxeO		
1	CDS	3018788	3018117	-	atgggttttgactttcactacatgc	MGFDFHYMLELLPILLKYLGTT	fig|127906.87.peg.2716	VCC2716	VC0009	yxeN	Probable amino-acid permease protein YxeN		
1	CDS	3019612	3018866	-	atgaaacactggtttaacatcgc	MKHWFNIALAALALSSATVQA	fig|127906.87.peg.2717	VCC2717	VC0010	yxeM	Probable amino-acid-binding protein YxeM		
1	CDS	3019745	3019864	+	gtggatctttatgtgggtagcacg	MDLYVGSTGKMCEDLSYRSK	fig|127906.87.peg.2718	VCC2718	VC0011		FIG01200880: hypothetical protein		
2	CDS	41	523	+	atgaacaagaaagaaatccaa	MNKKEIQAYQSAFEKFNPFYG	fig|127906.87.peg.2719	VCCA0001	VCA0793		hypothetical protein	Rha family transcriptional regulator [Vibrio cholerae]	
2	CDS	524	817	+	atgattaagcgtggtttcttcaaga	MIKRGFFKRDFDGGLVVKPSL	fig|127906.87.peg.2720	VCCA0002	VCA0794		hypothetical protein		
2	CDS	1140	1988	+	atgaaatactcattaatcaagcta	MKYSLIKLSAMNSYSDCQQAI	fig|127906.87.peg.2721	VCCA0003	VCA0795	pinR	resolvase, putative		Limited subject cover for certain strains
2	CDS	2226	2447	+	atgaataaccgacttgtcgaaga	MNNRLVEETIAEETIDCCPLC	fig|127906.87.peg.2722	VCCA0004	No refSeq annotation		COG1405: Transcription initiation factor TFIIIB, Brf1 subunit/Transcription initiation factor TFIIB		
2	CDS	3323	2643	-	atgactgaatccagagtaaagtg	MTESRVKCVIFDCEGTLVDSE	fig|127906.87.peg.2723	VCCA0005	VCA0798	yieH	6-phosphogluconate phosphatase		
2	CDS	3968	3462	-	atgatttactctccctcacctgtgg	MIYSPSPVVAAKNQWLSSQVD	fig|127906.87.peg.2724	VCCA0006	VCA0799		hypothetical protein		
2	CDS	4290	4772	+	atgctcaattctattcgcactgctt	MLNSIRTALLLSFAFVLSGCDR	fig|127906.87.peg.2725	VCCA0007	VCA0800	creA	Conserved uncharacterized protein CreA	CreA family protein [Vibrio cholerae]	
2	CDS	4891	6195	+	atgaagtttcccggtcagcgcaa	MKFPGQRKSKHYFPTHARDP	fig|127906.87.peg.2726	VCCA0008	VCA0801		Inosine-guanosine kinase (EC 2.7.1.73)		
2	CDS	8245	6281	-	ttgaataaactcaaattattctttgg	MNKLKLFFGPLYPIVCAVFALL	fig|127906.87.peg.2727	VCCA0009	VCA0802	ltaS2	Phosphatidylglycerol--membrane-oligosaccharide glycerophosphotransferase (EC 2.7.8.20)		
2	CDS	9328	8336	-	atgcgcaaatggctatggctcct	MRKWLWLLLLLTTRVSAVEISI	fig|127906.87.peg.2728	VCCA0010	VCA0803	tlp	Secreted trypsin-like serine protease		
2	CDS	11403	9472	-	atgcaagatactgcaattcaatt	MQDTAIQFSDLALNSAILSALT	fig|127906.87.peg.2729	VCCA0011	VCA0804	deaD	DEAD-box ATP-dependent RNA helicase DeaD (= CshA) (EC 3.6.4.13)		
2	CDS	11777	13813	+	atgttccaagacaatccgctcct	MFQDNPLLAQLKQKIQETLPK	fig|127906.87.peg.2730	VCCA0012	VCA0805	rnb	Exoribonuclease II (EC 3.1.13.1)		
2	CDS	14321	13908	-	atggaattgcatcgctcagatag	MELHRSDSHTEQTQTPMRLK	fig|127906.87.peg.2731	VCCA0013	VCA0806		hypothetical protein	DUF3024 domain-containing protein [Vibrio cholerae]	
2	CDS	14469	15305	+	atgatcaggatggcacttgccgc	MIRMALAAVCALLFSITTMTPF	fig|127906.87.peg.2732	VCCA0014	VCA0807		Phosphate ABC transporter, substrate-binding protein PstS (TC 3.A.1.7.1)		
2	CDS	16122	15451	-	atgagagtcgctgagcttctgaa	MRVAELLKHRAENLVKHTEFK	fig|127906.87.peg.2733	VCCA0015	VCA0808		Acyl dehydratase		
2	CDS	17967	16417	-	atgcgctattccgttgtgcgattga	MRYSVVRLILGDQLNHAHSWI	fig|127906.87.peg.2734	VCCA0016	VCA0809		hypothetical protein	Cryptochrome/photolyase family protein [Vibrio cholerae]	
2	CDS	18461	19918	+	atgaaaaaacaacctaaaatga	MKKQPKMTAIALILSGISGLAY	fig|127906.87.peg.2735	VCCA0017	VCA0811	gbpA	Chitin binding protein		
2	CDS	20249	21676	+	atgaaaagtaaaatagtgaccg	MKSKIVTATLALCCLSPSFSYA	fig|127906.87.peg.2736	VCCA0018	VCA0812		Zinc metalloprotease		
2	CDS	21711	23216	+	atgaacaaactattcgtcatggc	MNKLFVMALMSAALSANAEDH	fig|127906.87.peg.2737	VCCA0019	VCA0813		Bacterial leucyl aminopeptidase (EC 3.4.11.10)		
2	CDS	24333	23404	-	atgaatgatttgtttactaaaactg	MNDLFTKTDYSLYSNAMTFVR	fig|127906.87.peg.2738	VCCA0020	VCA0814	speB	Agmatinase (EC 3.5.3.11)		
2	CDS	26298	24334	-	atgtatttcaaccgatgaaatcatt	MISTDEIIVILSVFVRYDVEQPS	fig|127906.87.peg.2739	VCCA0021	VCA0815	speA	Biosynthetic arginine decarboxylase (EC 4.1.1.19)		
2	CDS	26502	26389	-	atgacattttattgcaaagaatc	MTFLLQRIDDRFAAMPCGIKG	fig|127906.87.peg.2740	VCCA0022	VCA0816		hypothetical protein		
2	CDS	26691	27251	+	atggaatacctagctttattacaa	MEYLALLQTHYKWLMTILIIVL	fig|127906.87.peg.2741	VCCA0023	VCA0817		Small-conductance mechanosensitive channel		
2	CDS	27254	28600	+	ttgagcgaataccaagacttatc	MSEYQDLSQQIELIATAEESE	fig|127906.87.peg.2742	VCCA0024	VCA0818	mgtE	Mg/Co/Ni transporter MgtE, CBS domain-containing		
2	CDS	28802	29092	+	atgaatattcgtcctttacatgaca	MNIRPLHDKLIVERLEVENKSE	fig|127906.87.peg.2743	VCCA0025	VCA0819	groES	Heat shock protein 10 kDa family chaperone GroES		
2	CDS	29121	30713	+	atggcagcaaaacacgttcttgtt	MAAKHVLFSTDARQKMLSGV	fig|127906.87.peg.2744	VCCA0026	VCA0820	groEL	Heat shock protein 60 kDa family chaperone GroEL		
2	CDS	30746	30952	+	atgacgaaactgcctcatttaca	MTKLPHLQLSVVEAQPKTHPA	fig|127906.87.peg.2745	VCCA0027	VCA0821		hypothetical protein		
2	CDS	32454	31033	-	atgtcttacaccgtagagaaaat	MSYTVEKIGGTSMTAFDAVLE	fig|127906.87.peg.2746	VCCA0028	VCA0822		Aspartokinase (EC 2.7.2.4) associated with ectoine biosynthesis		
2	CDS	32870	32454	-	atgattgttagaacgctagaaga	MIVRTLEECRQSERRVVAENV	fig|127906.87.peg.2747	VCCA0029	VCA0823	ectC	L-ectoine synthase (EC 4.2.1.108)		
2	CDS	34171	32906	-	atggatattttaaacaccacgaa	MDIFKHHESQVQSYANHFPVL	fig|127906.87.peg.2748	VCCA0030	VCA0824	ectB	Diaminobutyrate--2-oxoglutarate transaminase (EC 2.6.1.76)		
2	CDS	34737	34195	-	atgtttaccgctacgccttggatg	MFTATPWMIYPQIMHKPALPW	fig|127906.87.peg.2749	VCCA0031	VCA0825	ectA	L-2,4-diaminobutyric acid acetyltransferase (EC 2.3.1.178)		
2	CDS	35746	35417	-	atgctggatgaattacgttgtgaa	MLDELRCEACSAGAIGLTSEE	fig|127906.87.peg.2750	VCCA0032	VCA0827	phhB	Pterin-4-alpha-carbinolamine dehydratase (EC 4.2.1.96)		
2	CDS	36530	35736	-	atgactcagtatcactctaaacc	MTQYHSKPVSEHGHIDWDQD	fig|127906.87.peg.2751	VCCA0033	VCA0828	phhA	Phenylalanine-4-hydroxylase (EC 1.14.16.1)		
2	CDS	36764	38743	+	atgtcatcgactacaccattgtgg	MSSTTPLWTPSHERISASNLQ	fig|127906.87.peg.2752	VCCA0034	VCA0829		Acetoacetyl-CoA synthetase (EC 6.2.1.16)		
2	CDS	38874	39791	+	atggattatattcacctctcccgat	MDYIHLSRFSLKHLTALHVMLN	fig|127906.87.peg.2753	VCCA0035	VCA0830	nodD2	LysR-family transcriptional regulator VCA0830		
2	CDS	39793	39987	+	atgaaaatgggtgaaaaggga	MKMGEKGKGQQTLTLSFVALI	fig|127906.87.peg.2754	VCCA0036	VCA0831		FIG01201041: hypothetical protein		
2	repeat	40100	40219	+	cccaaacaacttggcgttacaggtaggcggcaagtgagagagtccccatg		fig|127906.87.repeat.57				repeat region		
2	CDS	40869	40291	-	atggcacacgactgggatgagt	MAHDWDEYAANWEKDPATHA	fig|127906.87.peg.2755	VCCA0037	VCA0832		SAM-dependent methyltransferase		
2	CDS	41065	41943	+	atgaacctgtcacaaatcgaag	MNLSQIEAFCTIANTGSVSEAA	fig|127906.87.peg.2756	VCCA0038	VCA0833	cmpR	Transcriptional regulator		
2	CDS	42137	43033	+	atgtgttctcaatccaattttaatg	MCSQSNFNEKRVLTLSALIAS	fig|127906.87.peg.2757	VCCA0039	VCA0834		Cobalt-zinc-cadmium resistance protein		
2	CDS	44012	43065	-	gtgaaacagcaattcatcactca	MKQQFITHKHTLYGVAAILLW	fig|127906.87.peg.2758	VCCA0040	VCA0835	yddG	Permease of the drug/metabolite transporter (DMT) superfamily		
2	CDS	44661	44089	-	atgagcgaattagaaaaaatgc	MSELEKMLKGEHFDGASAEIE	fig|127906.87.peg.2759	VCCA0041	VCA0836	maa	Acetyltransferase		
2	CDS	46118	44805	-	atgagtttttcgaaaacatctcga	MSFFENISIIMALIAASCFFSMS	fig|127906.87.peg.2760	VCCA0042	VCA0837		UPF0053 inner membrane protein YtfL	MULTISPECIES: HlyC/CorC family transporter [Vibrio]	
2	CDS	46677	46273	-	atgcaagcgcaagtcaaatggg	MQAQVKWVEDFRFIGLSNSG	fig|127906.87.peg.2761	VCCA0043	VCA0838		Protein YhfA		
2	CDS	47570	48055	+	atgatttcaaaatgggcgcaacc	MISKWAQRFFQMAELVGSWS	fig|127906.87.peg.2762	VCCA0044	VCA0840		dCMP deaminase (EC 3.5.4.12)		
2	CDS	48637	48485	-	ttgaccctgagcatagcgtttatc	MTLSIAFIYTRLSIPIVMNSPAA	fig|127906.87.peg.2763	VCCA0045	VCA0842		hypothetical protein	L-serine ammonia-lyase [Vibrio cholerae]	
2	CDS	48756	50240	+	atgcccagcgagggaattaact	MPSEGINSNVKGPKSTMSPEN	fig|127906.87.peg.2764	VCCA0046	VCA0843	gap	NADP-dependent glyceraldehyde-3-phosphate dehydrogenase (EC 1.2.1.13)		
2	CDS	50541	50765	+	atgaaccgattcctgatcaccta	MNRFLITLLSLITGVFALIFSLLL	fig|127906.87.peg.2765	VCCA0047	VCA0845		hypothetical protein		
2	CDS	51428	50814	-	atgacactgacagtgtggttatc	MTLTVWLSLFTICILGAMSPGF	fig|127906.87.peg.2766	VCCA0048	VCA0846		Transporter, LysE family		
2	CDS	51572	52858	+	atggagtatgctaaagctcaaat	MEYAKAQILCRWRKVNQLKK	fig|127906.87.peg.2767	VCCA0049	VCA0847	yjeH	L-methionine and branched-chain amino acid export protein YjeH		
2	CDS	53881	52901	-	atgaatgacaaagtgcttgagtc	MNDKVLESVIEITEQKNSLALS	fig|127906.87.peg.2768	VCCA0050	VCA0848		GGDEF domain protein		
2	CDS	63803	54012	-	atgcagtctcaagtcgtcactca	MQSQVVTQPVTVTSLSGNVV	fig|127906.87.peg.2769	VCCA0051	VCA0849	algE2	RTX toxins and related Ca2+-binding proteins	retention module-containing protein [Vibrio cholerae]	
2	CDS	64889	64104	-	atgatgaaccagacctacactg	MMNQTYTAVLADDEPLLRHHI	fig|127906.87.peg.2770	VCCA0052	VCA0850		Response regulator VCA0850		
2	CDS	66128	65049	-	atgatggaacgctttgatatttca	MMERFDISAQDLKSFGFTTLF	fig|127906.87.peg.2771	VCCA0053	VCA0851	ypdA	Autolysin sensor kinase (EC 2.7.3.-)		
2	CDS	67373	66129	-	atgaggtgtgctcaggcgttagg	MRCAQALGIIGLLSCPPAWTQ	fig|127906.87.peg.2772	VCCA0054	VCA0852		hypothetical protein		
2	CDS	68121	67378	-	atgtttaaactcattttatggtggt	MFKLIFMVVLGLVVMDRSFAV	fig|127906.87.peg.2773	VCCA0055	VCA0853		hypothetical protein	Outer membrane lipoprotein-sorting protein [Vibrio cholerae]	
2	CDS	69373	68114	-	atgggcaagttgatctcacaagc	MGKLISQAWRLAALNLQRNRF	fig|127906.87.peg.2774	VCCA0056	VCA0854		LolE-like permease protein		
2	CDS	70044	69361	-	atgctgcattttcaaagtatagaa	MLHFQSIEKQYSLGVQSVPAL	fig|127906.87.peg.2775	VCCA0057	VCA0855		ABC transporter, ATP-binding protein		
2	CDS	70724	70053	-	atgaaaattcgaacttcgttgagc	MKIRTSLSLTAYVSVLLFSLSA	fig|127906.87.peg.2776	VCCA0058	VCA0856		hypothetical protein		
2	CDS	70751	70885	+	ttgtcagaacgtaaacgtcctca	MSERKRPQLLIVFVPRRSSYQ	fig|127906.87.peg.2777	VCCA0059	VCA0857		FIG01202943: hypothetical protein		
2	CDS	70886	71029	+	atggacaatatcgggagcgaat	MDNIGSEWVARSEEIRYELIKT	fig|127906.87.peg.2778	VCCA0060	VCA0858		FIG01201764: hypothetical protein		
2	CDS	71960	71037	-	atgcaacaacaagcaaccgta	MQQQATVQKVTMAQQGPELS	fig|127906.87.peg.2779	VCCA0061	VCA0859	ydhF	Oxidoreductase YdhF		
2	CDS	72252	74324	+	atgaaactgaatgccttgaccct	MKLNALTLSLLSALALPALASP	fig|127906.87.peg.2780	VCCA0062	VCA0860	malS	Periplasmic alpha-amylase (EC 3.2.1.1)		
2	CDS	74314	74499	+	ttggccgctaagccactccatca	MAAKPLHQEHTQYAPEFVEIR	fig|127906.87.peg.2781	VCCA0063	VCA0861		conserved hypothetical protein		
2	CDS	74830	74708	-	ttgaagaacaaaagcaacattg	MKNKSNIGSTQECLVANRYSQ	fig|127906.87.peg.2782	VCCA0064	No refSeq annotation		FIG01200371: hypothetical protein		Limited query cover for certain strains
2	CDS	74859	76097	+	atgttgtataaatcttccctgtgt	MLYKSSLAVAISALIAGQVSAA	fig|127906.87.peg.2783	VCCA0065	VCA0862		Long-chain fatty acid transport protein		
2	CDS	76113	78503	+	atgaaacaggttattaagctctcc	MKQVIKLSLLCSALWLAGCGD	fig|127906.87.peg.2784	VCCA0066	VCA0863		Putative lipase		
2	CDS	78981	80261	+	atgtttctttttcaaaaacaaaaa	MFLFQKQKPSTKPSHQDNIVQ	fig|127906.87.peg.2785	VCCA0067	VCA0864		Methyl-accepting chemotaxis sensor/transducer protein		
2	CDS	82171	80342	-	atgaaaatgatacaacgtcctct	MKMIQRPLNWLVLAGAATGFF	fig|127906.87.peg.2786	VCCA0068	VCA0865	hap	Vibriolysin, extracellular zinc protease (EC 3.4.24.25) @ Pseudolysin, extracellular zinc protease (EC 3.4.24.26)		
2	CDS	82187	82396	+	atgttgaatgtattaagcgttgaa	MLNVLSVEPLPSTPIAREIPRS	fig|127906.87.peg.2787	VCCA0069	VCA0866		FIG01201512: hypothetical protein		
2	CDS	83245	82592	-	atgaaacaaacccatttgcggcc	MKQTICGLAVLAALSSAPVFAH	fig|127906.87.peg.2788	VCCA0070	VCA0867	ompW	Outer membrane protein W precursor		
2	CDS	83486	83668	+	atggacacactctacaaaatcg	MDTLYKIESYSDEAVNTIAEFI	fig|127906.87.peg.2789	VCCA0071	VCA0868		hypothetical protein		
2	CDS	84199	85149	+	atgaagatcttctctcgccttggtc	MKIFSRLGHVLLICSSLIIAIPAH	fig|127906.87.peg.2790	VCCA0072	VCA0870	pbpG	Murein-DD-endopeptidase (EC 3.4.99.-)		
2	CDS	85387	86805	+	atgaagaaatcgcaagtactgg	MKKSQVLANTIKSQIEQNIWLS	fig|127906.87.peg.2791	VCCA0073	VCA0871	ydcR	DNA-binding transcriptional regulator, MocR family / aminotransferase domain		
2	CDS	86946	88526	+	atgattgatgtgttgaattatcgc	MIDVVELSRLQFALTAMFHFLF	fig|127906.87.peg.2792	VCCA0074	VCA0872		Cytochrome d ubiquinol oxidase subunit I (EC 1.10.3.-)		
2	CDS	88539	89675	+	atgtttgaatatgacacattgcgc	MFEYDTLRLIWWGLIGALLIGF	fig|127906.87.peg.2793	VCCA0075	VCA0873		Cytochrome d ubiquinol oxidase subunit II (EC 1.10.3.-)		
2	CDS	89686	89787	+	atgtggtatttcatatggatttcag	MWYFIWISGLFLAATFSILNIIN	fig|127906.87.peg.2794	VCCA0076	Not annotated as CDS		Cytochrome d ubiquinol oxidase subunit X (EC 1.10.3.-)		
2	CDS	89978	90100	+	atgagtgatgctaaaaactacac	MSDAKNYNQCVHTLTLVKHV	fig|127906.87.peg.2795	VCCA0077	Not annotated as CDS		hypothetical protein		
2	CDS	90497	90700	+	atgaagaacaaaagagttaaa	MKNKRVKLPKKNKKGAYEAK	fig|127906.87.peg.2796	VCCA0078	VCA0874		hypothetical protein		
2	CDS	92239	90917	-	atgacgatcaacatagagcaac	MTINIEQLTEQYPLVKELIELKE	fig|127906.87.peg.2797	VCCA0079	VCA0875	dsdA	D-serine ammonia-lyase (EC 4.3.1.18)		
2	CDS	92450	93394	+	atgtacactaaggataatatcttt	MYTKDNIFAKHSRINSFQLSKL	fig|127906.87.peg.2798	VCCA0080	VCA0876	dsdC	D-serine dehydratase transcriptional activator		
2	CDS	93694	94770	+	atgaagatgaaacacatgaaa	MKMKHMKPYTTMKTVLSTLAI	fig|127906.87.peg.2799	VCCA0081	VCA0877	cbh	Putative hydrolase		
2	CDS	94878	95039	+	atgatcactcacgctaacgtcaa	MITHANVNVSYLAQQMGHAD	fig|127906.87.peg.2800	VCCA0082	VCA0878		Integrase		
2	CDS	95668	96048	+	atgaaaaaaatagaaatacttat	MKKIEILIVVDCAGALATTSLIS	fig|127906.87.peg.2801	VCCA0083	VCA0880		hypothetical protein		
2	CDS	96073	96468	+	atgaaaaaagtagaaatactca	MKKVEILMVVDAAAALASRDL	fig|127906.87.peg.2802	VCCA0084	VCA0881		hypothetical protein		
2	CDS	96480	97544	+	atgttaagcccaaatgttgatacc	MLSPNVDTALNMLTDVYTDFL	fig|127906.87.peg.2803	VCCA0085	VCA0882		hypothetical protein		Limited query cover for certain strains
2	CDS	97557	98666	+	atgtcacaacaagttactcagttg	MSQQVTQLNPTQQTTQSAFL	fig|127906.87.peg.2804	VCCA0086	VCA0883		Non-hemolytic enterotoxin lytic component L1		
2	CDS	98717	99778	+	atgaatcaatcagccagcgagg	MNQSASEQLQTDIPASISAMV	fig|127906.87.peg.2805	VCCA0087	VCA0884		Non-hemolytic enterotoxin lytic component L1		
2	CDS	101906	100875	-	atgaaaatcaaagcactttcaaa	MKIKALSKLKPEQGIWMNEVD	fig|127906.87.peg.2806	VCCA0088	VCA0885	tdh	L-threonine 3-dehydrogenase (EC 1.1.1.103)		
2	CDS	103102	101909	-	atgtcttctgcttttatcagcaaat	MSSAFYQQIRTQLEEVKAEGL	fig|127906.87.peg.2807	VCCA0089	VCA0886	kbl	2-amino-3-ketobutyrate coenzyme A ligase (EC 2.3.1.29)		
2	CDS	103422	103733	+	atgacgacagtaatgaatgata	MTTVMNDKHPTPDPAEDNAF	fig|127906.87.peg.2808	VCCA0090	VCA0887		D-lactate dehydratase (EC 4.2.1.130)		C6706 have 2 ORFs, while N16961 only have 1 ORF with D-lactate dehydratase. VCA0887 annotated as pseuogene in KEGG
2	CDS	103751	104281	+	atgccaagagaagaccaaga	MPREDQEVNGLYSKYQSSFR	fig|127906.87.peg.2809	VCCA0091	VCA0887		D-lactate dehydratase (EC 4.2.1.130)		C6706 have 2 ORFs, while N16961 only have 1 ORF with D-lactate dehydratase. VCA0887 annotated as pseuogene in KEGG
2	CDS	104301	105020	+	atgcccgctgtcgatttaatcacc	MPAVDLITLISQLEESHSFSTV	fig|127906.87.peg.2810	VCCA0092	VCA0888	lasR	Transcriptional regulator, LuxR family		
2	CDS	105120	106058	+	atgctacttcccaaattgattgctc	MLLPKLIALLPDLATFIVVVNEG	fig|127906.87.peg.2811	VCCA0093	VCA0889		Transcriptional regulator, LysR family		
2	CDS	106052	106435	+	atgctaaaacgcattcatcatgc	MLKRIHHAAIICSDYPRSKAFY	fig|127906.87.peg.2812	VCCA0094	VCA0890	fosA	Uncharacterized protein YaeR with similarity to glyoxylase family	VOC family protein [Vibrio cholerae]	
2	CDS	107925	106513	-	ttgagtaccgttactacacctcaa	MSTVTTPQPTAAARRKTLYFL	fig|127906.87.peg.2813	VCCA0095	VCA0891		Uncharacterized iron-regulated membrane protein; Iron-uptake factor PiuB	PepSY domain-containing protein [Vibrio cholerae]	
2	CDS	108384	108037	-	atgaacccacaacgtaccttca	MNPQRTFHRLMLKLSLLSWLL	fig|127906.87.peg.2814	VCCA0096	VCA0892		hypothetical protein		
2	CDS	108924	108541	-	atgaaaaaaacactgattctagt	MKKTLILVAALLATPVLAAGQD	fig|127906.87.peg.2815	VCCA0097	VCA0893		Soluble cytochrome b562		
2	CDS	109747	109001	-	atgaaattagctgtagatactcac	MKLAVDTHTHTYASGHAYSTL	fig|127906.87.peg.2816	VCCA0098	VCA0894	ycdX	putative histidinol phosphatase and related hydrolases of the PHP family		
2	CDS	109972	112860	+	atgaaaaagcgtcgttattccct	MKKRRYSLSIHISSLFFILILFIG	fig|127906.87.peg.2817	VCCA0099	VCA0895		Chemotactic transducer-related protein		
2	CDS	113207	114709	+	atggtaatacctgaaaacagca	MVIPENSSIVIFGASGDLTYRK	fig|127906.87.peg.2818	VCCA0100	VCA0896	zwf	Glucose-6-phosphate 1-dehydrogenase (EC 1.1.1.49)		
2	CDS	114706	115422	+	atgatcaaccataaaatttccca	MINHKIFPTADAVVKSLADDMI	fig|127906.87.peg.2819	VCCA0101	VCA0897	pgl	6-phosphogluconolactonase (EC 3.1.1.31), eukaryotic type		
2	CDS	115436	116884	+	atgaaaggtgatatcggtgtaat	MKGDIGVIGLAVMGQNLILNMI	fig|127906.87.peg.2820	VCCA0102	VCA0898	gndA	6-phosphogluconate dehydrogenase, decarboxylating (EC 1.1.1.44)		
2	CDS	117040	117177	+	atgacaatcaacaagtactatgt	MTINKYYVLVPQALEQSTHQD	fig|127906.87.peg.2821	VCCA0103	VCA0899		hypothetical protein		
2	CDS	117496	118359	+	atgaacacattaaccaaactac	MNTLTKLLAATMTLSAQAMAA	fig|127906.87.peg.2822	VCCA0104	VCA0900		Metallo-beta-lactamase superfamily protein PA0057		
2	CDS	118353	118982	+	atggtaagggttcactacattttg	MVRVHYIFDPMCGWCFGATS	fig|127906.87.peg.2823	VCCA0105	VCA0901		Thioredoxin-like protein clustered with PA0057		
2	CDS	119851	119015	-	atgttgaaatggcttcgtgtacttc	MLKWLRVLLLCTFLVGCATKL	fig|127906.87.peg.2824	VCCA0106	VCA0902		hypothetical protein	putative lipoprotein [Vibrio cholerae]	
2	CDS	121120	119987	-	atgaaagtggttatcgccccaga	MKVVIAPDSFKESLTAKQVCD	fig|127906.87.peg.2825	VCCA0107	VCA0903	glxK	Glycerate kinase (EC 2.7.1.31)		
2	CDS	122491	121139	-	atgagtctgatcttaatcctgttag	MSLILILLAVIVFIVLATTKFKVH	fig|127906.87.peg.2826	VCCA0108	VCA0904	dsdX	D-glycerate transporter (predicted)		
2	CDS	123789	122650	-	atgatactggacagtcaattagc	MILDSQLAQQIVDRTMAIIGYN	fig|127906.87.peg.2827	VCCA0109	VCA0905	cdaR	Sugar diacid utilization regulator SdaR		
2	CDS	125666	123930	-	atgaaaattaacaccacgatga	MKINTTMKLVFSAIGVGMAITM	fig|127906.87.peg.2828	VCCA0110	VCA0906		Methyl-accepting chemotaxis sensor/transducer protein		
2	CDS	126498	125968	-	atggatcagcaagttaagcaag	MDQQVKQERLQGRLEPEIKEI	fig|127906.87.peg.2829	VCCA0111	VCA0907	hutZ	Pyridoxamine 5'-phosphate oxidase-related putative heme iron utilization protein		
2	CDS	127026	126523	-	atggaatcattacaacagcaag	MESLQQQVAQLLEQQPTLLPA	fig|127906.87.peg.2830	VCCA0112	VCA0908	hutX	Putative heme iron utilization protein		
2	CDS	128411	127050	-	atgttgattttagataatttgatga	MLILDNFDESILGANTPDPLRF	fig|127906.87.peg.2831	VCCA0113	VCA0909	hutW	Radical SAM family protein HutW, similar to coproporphyrinogen III oxidase, oxygen-independent, associated with heme uptake		
2	CDS	128689	129423	+	gtgaatctgaacagatatgtcatt	MNLNRYVIAGGLSLAFHALLLI	fig|127906.87.peg.2832	VCCA0114	VCA0910	tonB1	putative TonB-dependent receptor		
2	CDS	129423	130109	+	atggaatcgttacaacaactcca	MESLQQLQQQLGLMAWPLFIG	fig|127906.87.peg.2833	VCCA0115	VCA0911	exbB1	Ferric siderophore transport system, biopolymer transport protein ExbB		
2	CDS	130106	130522	+	atgatcaagtcctcagccaaac	MIKSSAKHNDFGLTPDLTPLLD	fig|127906.87.peg.2834	VCCA0116	VCA0912	exbD1	Biopolymer transport protein ExbD1		
2	CDS	130533	131366	+	atgaaaatcactcaatcattggc	MKITQSLALLLACCTSGISFAQ	fig|127906.87.peg.2835	VCCA0117	VCA0913	hmuT/hutB	Periplasmic hemin-binding protein		
2	CDS	131378	132409	+	gtgttaagacgatttccttttgctgt	MLRRFPFAVTISCLTLAVFFAG	fig|127906.87.peg.2836	VCCA0118	VCA0914		Hemin ABC transporter, permease protein		
2	CDS	132409	133188	+	atgcagactattgcaattcaagg	MQTIAIQGRDLCVTYGSRQVL	fig|127906.87.peg.2837	VCCA0119	VCA0915	hmuV	ABC-type hemin transport system, ATPase component		
2	CDS	133783	133322	-	atgcctaagcgtagtaaagaag	MPKRSKEDTEVTIQTIMDAVV	fig|127906.87.peg.2838	VCCA0120	VCA0917		Transcriptional regulator LuxT		
2	CDS	134306	134608	+	atgagtaaccaaacttgtgtaga	MSNQTCVENEVCEACGCAGE	fig|127906.87.peg.2839	VCCA0121	VCA0919		hypothetical protein	DUF406 family protein [Vibrio cholerae]	
2	CDS	134811	135410	+	atgatgcttcgtgttattgcccttc	MMLRVIALSLSLIAGFTHAMAL	fig|127906.87.peg.2840	VCCA0122	VCA0920		Leucine-rich-repeat protein		
2	CDS	136589	135471	-	atgtacaaacctatgacttcaac	MYKPMTSTTDPYIDIRPYNDD	fig|127906.87.peg.2841	VCCA0123	VCA0921		hypothetical protein	acyltransferase [Vibrio cholerae]	
2	CDS	136755	137363	+	atgaactacgctattcagtctgaa	MNYAIQSETRHYPYLEVSARK	fig|127906.87.peg.2842	VCCA0124	VCA0922		AraC-type DNA-binding domain-containing protein		
2	CDS	137510	139384	+	atgaaattcagccataaaattgtt	MKFSHKIVAASSVLLLATVALL	fig|127906.87.peg.2843	VCCA0125	VCA0923		Methyl-accepting chemotaxis sensor/transducer protein		
2	CDS	141264	139447	-	atgtcgtctggacttctatctcaaa	MSSGLLSQINVFPVKSLGGLA	fig|127906.87.peg.2844	VCCA0126	VCA0924		Flavodoxin reductases (ferredoxin-NADPH reductases) family 1		
2	repeat	141493	141685	+	atacccaaaccacttggagttgcaggtaggcggcaagtgagtgacaaatttg		fig|127906.87.repeat.58				repeat region		
2	CDS	141827	142855	+	atgacaacactgacgattacac	MTTLTITRPDDWHVHLRDGDV	fig|127906.87.peg.2845	VCCA0127	VCA0925	pyrC	Dihydroorotase (EC 3.5.2.3)		
2	CDS	143961	142918	-	atgttgatgattccagtgcccttcc	MLMIPVPFVVSLMLLLAVTLY	fig|127906.87.peg.2846	VCCA0128	VCA0926		Transcriptional regulator, AraC family		
2	CDS	144126	144476	+	atgacacctttactgaccgcctat	MTPLLTAYLQAWKQYFDFSG	fig|127906.87.peg.2847	VCCA0129	VCA0927	yhaI	Integral membrane protein	DUF805 domain-containing protein [Vibrio cholerae]	

2	CDS	144473	144982	+	atgagaactctattggttttactgc	MRTLLVLLLISMANLASADPLK	fig|127906.87.peg.2848	VCCA0130	VCA0928		hypothetical protein		
2	CDS	146159	145032	-	atgcagtgtgagttctttattcaaa	MQCEFFIQKRCTSCHLCAQPY	fig|127906.87.peg.2849	VCCA0131	VCA0929	rlmC/rumB	23S rRNA (uracil(747)-C(5))-methyltransferase (EC 2.1.1.189)		
2	CDS	147208	146273	-	atggcaggcgcaagcttactga	MAGASLLTLLDDIATVLDDVAL	fig|127906.87.peg.2850	VCCA0132	VCA0930	yedI	Inner membrane protein YedI	DUF808 domain-containing protein [Vibrio cholerae]	
2	CDS	148703	147321	-	atgagtgttgcacaaaatacctt	MSVAQNTFPLSELMISLTTALD	fig|127906.87.peg.2851	VCCA0133	VCA0931		Response regulator		
2	CDS	149052	149264	+	atgtctactaaaatgactggttct	MSTKMTGSVKWFNETKGFGF	fig|127906.87.peg.2852	VCCA0134	VCA0933		Cold shock protein of CSP family		
2	CDS	149918	150190	+	atggcgaatacaatgttaggtgg	MANTMLGGWGLFHELSNEDK	fig|127906.87.peg.2853	VCCA0135	VCA0935		hypothetical protein		
2	CDS	150405	151340	+	atgtctgcctcatccccctacccg	MSASSPYPIGTPGQPWQASE	fig|127906.87.peg.2854	VCCA0136	VCA0936		hypothetical protein	M14 family metallocarboxypeptidase [Vibrio cholerae]	
2	CDS	152054	151329	-	atgaattttgaagcgtttctgtcca	MNFEAFLSNTETREHSHPWG	fig|127906.87.peg.2855	VCCA0137	VCA0937	appY	Transcriptional regulator, AraC family		
2	CDS	152220	153119	+	atgatctatcttctgccatttttact	MIYLLPFFTVLIWGGNSIVNKL	fig|127906.87.peg.2856	VCCA0138	No refSeq annotation		putative membrane protein	DMT family transporter [Vibrio cholerae]	1 ORF split into 2 ORFs in N16961, none have refseq annotation in PATRIC. Annotated as 1 pseudogene named VCA0938 in KEGG
									No refSeq annotation				1 ORF split into 2 ORFs in N16961, none have refseq annotation in PATRIC. Annotated as 1 pseudogene named VCA0938 in KEGG
2	CDS	153277	154203	+	atgatagaacttaatagaattga	MIELNRIEELFDNQQFSLHELV	fig|127906.87.peg.2857	VCCA0139	VCA0939		Sensory box/GGDEF family protein		
2	CDS	154355	155116	+	gtgaaagcagtaaagcgccatc	MKAVKRHQEIIELVQAQGFVS	fig|127906.87.peg.2858	VCCA0140	VCA0940		Glycerol-3-phosphate regulon repressor GlpR		
2	CDS	155268	155756	+	atgagcagtggcaaacgagag	MSSGKREITLRFLAEPGDVNF	fig|127906.87.peg.2859	VCCA0141	VCA0941		cytosolic long-chain acyl-CoA thioester hydrolase family protein		
2	CDS	155867	156118	+	atggcgattaccctagataaaat	MAITLDKITLTETTLTNPKAVEY	fig|127906.87.peg.2860	VCCA0142	VCA0942		hypothetical protein		
2	CDS	157331	156441	-	atggctatggtacaaggcaaat	MAMVQGKSLKYRVWATHAAL	fig|127906.87.peg.2861	VCCA0143	VCA0943	malG	Maltodextrin ABC transporter, permease protein MdxG		
2	CDS	158917	157343	-	atgcagtcagttcaaggtacaca	MQSVQGTQAMSDPTAVRPAD	fig|127906.87.peg.2862	VCCA0144	VCA0944	malF	Maltodextrin ABC transporter, permease protein MdxF		
2	CDS	160173	158992	-	atgaaaaatgccctaagcacac	MKNALSTVALSTLVALGSFGA	fig|127906.87.peg.2863	VCCA0145	VCA0945	malE	Maltodextrin ABC transporter, substrate-binding protein MdxE		
2	CDS	160846	161967	+	atggcgagtgtcacgttaaaaa	MASVTLKNVCKAYGDVLISKN	fig|127906.87.peg.2864	VCCA0146	VCA0946	malK	Maltose/maltodextrin transport ATP-binding protein MalK (EC 3.6.3.19)		
2	CDS	162190	162711	+	atgaacagtcagttaacgcttag	MNSQLTLRALERGDLRFIHNL	fig|127906.87.peg.2865	VCCA0147	VCA0947	speG	Spermidine N1-acetyltransferase (EC 2.3.1.57)		
2	CDS	162716	162961	+	atggttagctgcagtcaatacga	MVSCSQYDYIELACLFHLPVK	fig|127906.87.peg.2866	VCCA0148	VCA0948		Rho-specific inhibitor of transcription termination (YaeO)		Limited query cover for certain strains
2	CDS	163707	163099	-	atggcgttagatggattaatgga	MALDGLMDAIADRGWYVWDD	fig|127906.87.peg.2867	VCCA0149	VCA0949		SM-20-related protein	SM-20 [Vibrio cholerae]	
2	CDS	163923	164243	+	atgattgtgacaacgacaccca	MIVTTTPNIEGKRIVRYCGVIA	fig|127906.87.peg.2868	VCCA0150	VCA0951		UPF0145 protein YbjQ		
2	CDS	165084	164410	-	atgaaagatgaaaacaaacta	MKDENKLNVRMLSDVCMQSR	fig|127906.87.peg.2869	VCCA0151	VCA0952	vpsT	Transcriptional regulator VpsT		
2	CDS	165940	165662	-	atggctcgcactgcagcagcttt	MARTAAALHILVKHKEQADDIL	fig|127906.87.peg.2870	VCCA0152	VCA0953	ppiC	Peptidyl-prolyl cis-trans isomerase PpiC (EC 5.2.1.8)		
2	CDS	166115	167035	+	atggctaaagtcgtcagtaaagc	MAKVVSKANQSQGMLMFTLS	fig|127906.87.peg.2871	VCCA0153	VCA0954		Chemotaxis protein CheV (EC 2.7.3.-)		
2	CDS	167635	167111	-	atggattcagtgcaaaaatccgt	MDSVQKSVMEWANELPELNT	fig|127906.87.peg.2872	VCCA0154	VCA0955		Transcriptional regulator, MarR family	MarR family transcriptional regulator [Vibrio cholerae]	
2	CDS	167807	168823	+	atgacaactgaagatttcaaaa	MTTEDFKKSTANLKKVVPLMM	fig|127906.87.peg.2873	VCCA0155	VCA0956		GGDEF domain protein		
2	CDS	169520	168942	-	atgaacatgctgacgtttgataa	MNMLTFDKTEIQHQNKPFIAE	fig|127906.87.peg.2874	VCCA0156	VCA0957	glcB	Malate synthase-related protein		
2	CDS	169753	171186	+	atggaggatctttctgcgcgctgc	MEDLSARCIRVSPEYAPSVSY	fig|127906.87.peg.2875	VCCA0157	VCA0958		Predicted transcriptional regulator	DUF3612 domain-containing protein [Vibrio cholerae]	
2	CDS	172839	171466	-	atggggttaacctcgcacaaata	MGLTSHKYKYVSIYFLALLFLG	fig|127906.87.peg.2876	VCCA0158	VCA0960		GGDEF domain protein		
2	CDS	173181	173789	+	atgatgaaaaaactgtttttggat	MMKKLFLDTHSASGEIKLTDG	fig|127906.87.peg.2877	VCCA0159	VCA0961		Alkylated DNA repair protein		
2	CDS	174076	175284	+	atgatgaacaaaactcaaatag	MMNKTQIAVVSSLITALILPTFT	fig|127906.87.peg.2878	VCCA0160	VCA0962		hypothetical protein	DUF3541 domain-containing protein [Vibrio cholerae]	
2	CDS	176232	175309	-	atgccaaaccatgttttaccagtc	MPNHVLPVLFMLLSTFSLSLT	fig|127906.87.peg.2879	VCCA0161	VCA0963		Permease of the drug/metabolite transporter (DMT) superfamily		
2	CDS	176333	177238	+	atgcgtaaattggcccccttaaa	MRKLAPLKSLYSFVAVAETGS	fig|127906.87.peg.2880	VCCA0162	VCA0964		Transcriptional regulator, LysR family		
2	CDS	177405	178580	+	atggatagctttgctggcaacca	MDSFAGNQLKEMTEMRFARK	fig|127906.87.peg.2881	VCCA0163	VCA0965		GGDEF domain protein		
2	CDS	178879	178583	-	atgggagggattgccatggctat	MGGIAMAILPKHNDRYPNYDP	fig|127906.87.peg.2882	VCCA0164	VCA0966		hypothetical protein		
2	CDS	179054	179902	+	atgactaaagaccgcgaaattc	MTKDREIRQTVPAQPTSDGD	fig|127906.87.peg.2883	VCCA0165	VCA0969	yhhW	Pirin		
2	CDS	180327	180040	-	atgatcaaggaaaacgtgtatttt	MIKENVYFDGNVKSLGFSQQD	fig|127906.87.peg.2884	VCCA0166	VCA0970		Pyrimidine/purine nucleoside phosphorylase PpnP		
2	repeat	180665	180792	+	ctatacccaaacgacttggagttgcaggtaggcggcaagtcagtgaatccc		fig|127906.87.repeat.59				repeat region		
2	CDS	180866	181306	+	atgtcaaaagagctggtctttaaa	MSKELVFKSVFWSSLSILGVA	fig|127906.87.peg.2885	VCCA0167	VCA0971		hypothetical protein	DUF2850 domain-containing protein [Vibrio cholerae]	
2	CDS	182587	181280	-	atggggaaaagtaaggaaaaa	MGKSKEKGIESSESLFQWERV	fig|127906.87.peg.2886	VCCA0168	VCA0972	mdtH	Uncharacterized MFS-type transporter	permease [Vibrio cholerae INDRE 91/1]	
2	CDS	182671	182844	+	atgggcaaagatagcggaattt	MGKDSGISQSNKNRNKYHND	fig|127906.87.peg.2887	VCCA0169	VCA0973		hypothetical protein		
2	CDS	184505	182877	-	atgccatttcgctgaaaaacttg	MPFSLKNLSIRLQVLLPVLFTM	fig|127906.87.peg.2888	VCCA0170	VCA0974	tap	Methyl-accepting chemotaxis sensor/transducer protein		
2	CDS	187137	184777	-	atggcgattcagcatctgagtttt	MAIQHLSFEQLYQVAKLEKLE	fig|127906.87.peg.2889	VCCA0171	VCA0975		ATP-dependent protease La (EC 3.4.21.53) Type II		
2	CDS	187341	187835	+	atggaattacagcagtgttggaa	MELQQCWNSFLQAEQLLKQG	fig|127906.87.peg.2890	VCCA0172	VCA0976		hypothetical protein		
2	CDS	187864	189483	+	atgtcacccaatccactttgcaa	MSPNPLLQVRNLSVSFTTNDG	fig|127906.87.peg.2891	VCCA0173	VCA0977		ABC transporter, ATP-binding protein (cluster 5, nickel/peptides/opines) / ABC transporter, ATP-binding protein (cluster 5, nickel/peptides/opines)		
2	CDS	190381	189650	-	atgatgaaatccattgctactttaa	MMKSIATLISVVLCSFSAWAQ	fig|127906.87.peg.2892	VCCA0174	VCA0978		ABC-type amino acid transport/signal transduction system, periplasmic component/domain		
2	CDS	191285	190401	-	gtgactcaaggcattaatcagttc	MTQGINQFTTTLRTIVVEVNNA	fig|127906.87.peg.2893	VCCA0175	VCA0979		Methyl-accepting chemotaxis protein		
2	CDS	191949	191305	-	atgtttagaagtattaaagtcgcc	MFRSIKVASIVQIVFAVCAVLLA	fig|127906.87.peg.2894	VCCA0176	VCA0980		Methyl-accepting chemotaxis protein		
2	CDS	192823	192080	-	atgcgtcatggactaagcaaatg	MRHGLSKWLWIPLLSLSAAFS	fig|127906.87.peg.2895	VCCA0177	VCA0981		hypothetical protein	transporter substrate-binding domain-containing protein [Vibrio cholerae]	
2	CDS	193876	192977	-	atgcgtgccgatgacttaattttgt	MRADDLILFYQVIELGSFSKVA	fig|127906.87.peg.2896	VCCA0178	VCA0982		Predicted Lactate-responsive regulator, LysR family		
2	CDS	194082	195776	+	atgaatgaaacactgctcgcgc	MNETLLALVAFSPIVVAAILLV	fig|127906.87.peg.2897	VCCA0179	VCA0983	lutP	L-lactate permease		
2	CDS	195899	197035	+	atgatcatttccgcttcgaccgat	MIISASTDYRAAAKAKLPPFLF	fig|127906.87.peg.2898	VCCA0180	VCA0984	lldD	L-lactate dehydrogenase		
2	CDS	197133	200012	+	atgaccccaccgatcgccgata	MTPPIADSLYLQLEQILASRIDF	fig|127906.87.peg.2899	VCCA0181	VCA0985		Predicted D-lactate dehydrogenase, Fe-S protein, FAD/FMN-containing		
2	CDS	200923	200096	-	atggaatcgcaacgccgtgatg	MESQRRDVFYVSDGTAITCET	fig|127906.87.peg.2900	VCCA0182	VCA0986	ppsR	Phosphoenolpyruvate synthase regulatory protein		
2	CDS	201103	203475	+	atgcaaaagaacaccctctggt	MQKNTLWFDSLSMQDVDKVG	fig|127906.87.peg.2901	VCCA0183	VCA0987	ppsA	Phosphoenolpyruvate synthase (EC 2.7.9.2)		
2	CDS	203658	205214	+	atgcgcaataaccaacctgttac	MRNNQPVTQKEVTYPPHFNLI	fig|127906.87.peg.2902	VCCA0184	VCA0988		Aerotaxis sensor receptor protein		
2	CDS	205540	205415	-	gtggagccgcagtggttacccg	MEPQWLPEYTSSIECRYAHGI	fig|127906.87.peg.2903	VCCA0185	No refSeq annotation		hypothetical protein		
2	CDS	205578	206906	+	atgcaaacttcaacgtcttctttac	MQTSTSSLAKQLFQMTWPML	fig|127906.87.peg.2904	VCCA0186	VCA0989		Na+-driven multidrug efflux pump		
2	CDS	208205	206985	-	atgtcattttcttctctcgcgttaag	MSFSSLALSADLIQALPKAITE	fig|127906.87.peg.2905	VCCA0187	VCA0990		ATP-dependent RNA helicase VCA0990		
2	CDS	209289	208363	-	atggataagtttgcagatatggtc	MDKFADMVLFASIVKNQGLAA	fig|127906.87.peg.2906	VCCA0188	VCA0991	grxB/grx2	Transcriptional regulator VCA0991, LysR family		
2	CDS	209410	210066	+	atgaaactctatatttatgaacac	MKLYIYEHCPFSARVRYVAGM	fig|127906.87.peg.2907	VCCA0189	VCA0992		Glutaredoxin 2		
2	CDS	210076	211179	+	atgagcagattatttgaaccaac	MSRLFEPTDLKQLTLQNRVVM	fig|127906.87.peg.2908	VCCA0190	VCA0993	nemA	2,4-dienoyl-CoA reductase [NADPH] (EC 1.3.1.34)		
2	CDS	212271	211300	-	atgaatgttcatctacaacccaa	MNVHLQPNMLIPRSAAQTARA	fig|127906.87.peg.2909	VCCA0191	VCA0994		hypothetical protein		
2	CDS	212637	214496	+	atgtttaagctatttgaaggtttca	MFKLFEGFTDPFPKGEPQRPF	fig|127906.87.peg.2910	VCCA0192	VCA0996		Efflux ABC transporter, permease/ATP-binding protein Atu2242		
2	CDS	214648	215352	+	atgaataaaagccttattaccaa	MNKSLITNVVALALMGVGYFL	fig|127906.87.peg.2911	VCCA0193	VCA0997		hypothetical protein	DUF445 domain-containing protein [Vibrio cholerae]	
2	CDS	216485	215442	-	atgaccgatattctttttcagccat	MTDILFQPYALNDVITLNNRIAI	fig|127906.87.peg.2912	VCCA0194	VCA0998		NADH:flavin oxidoreductases, Old Yellow Enzyme family		
2	CDS	216635	217210	+	gtgcgagtagctgaatttgaccg	MRVAEFDREQVLRSAMDEFM	fig|127906.87.peg.2913	VCCA0195	VCA0999		Transcriptional regulator, AcrR family		
2	CDS	217911	217282	-	atgattgatgtttcggtattaccgg	MIDVSVLPVYLTAVLALLLIPGF	fig|127906.87.peg.2914	VCCA0196	VCA1000		Threonine efflux protein		
2	CDS	218115	218945	+	atgagcgactcagccggcaaa	MSDSAGKIKEQAKFQIAEEFG	fig|127906.87.peg.2915	VCCA0197	VCA1001	ada	FIG229276: Transcriptional regulator, AraC family		
2	CDS	219097	219816	+	atgaatagccaagtgttaaccat	MNSQVLTIDDSPTPTRLFWQG	fig|127906.87.peg.2916	VCCA0198	VCA1002	ygaZ	FIG006238: AzlC family protein		
2	CDS	219813	220130	+	atgatcatgctttcgatattcgcga	MIMLSIFAMTALVFLSRYLFLEI	fig|127906.87.peg.2917	VCCA0199	VCA1003		FIG023406: hypothetical protein	AzlD domain-containing protein [Vibrio cholerae]	
2	CDS	220796	220182	-	atgagtcatatccaatttgccaac	MSHIQFANRNLSVGKVLCVGF	fig|127906.87.peg.2918	VCCA0200	VCA1004		2-keto-4-pentenoate hydratase (EC 4.2.1.80)		
2	CDS	221370	220912	-	atgccttctccacaagtgagctgc	MPSPQVSCQTPTHDPLLLENC	fig|127906.87.peg.2919	VCCA0201	VCA1005	ohrR	Organic hydroperoxide resistance transcriptional regulator		
2	CDS	221482	221904	+	atgagtacgatttatcaaacttcc	MSTIYQTSATASAGRNGVVST	fig|127906.87.peg.2920	VCCA0202	VCA1006	ohrB	Organic hydroperoxide resistance protein		
2	CDS	222160	223155	+	atgaaaaaagcagcaatcgca	MKKAAIAVAVLSAVVSGSTLAA	fig|127906.87.peg.2921	VCCA0203	VCA1008		Outer membrane porin	porin [Vibrio cholerae]	
2	CDS	223181	223300	+	ttgtccttctttggccaagcgattg	MSFFGQAIAWPFLLQNFERHE	fig|127906.87.peg.2922	VCCA0204	Not annotated as CDS		hypothetical protein		
2	CDS	223355	224212	+	atgctgacggctggcctcgcgtt	MLTAGLAFAIVNSVAQYASIQF	fig|127906.87.peg.2923	VCCA0205	VCA1010		Putative transporter, DME family	EamA family transporter [Vibrio cholerae]	
2	CDS	224260	224877	+	atgacaacaccgatcaaagtca	MTTPIKVTLYRWAGHFGPFKV	fig|127906.87.peg.2924	VCCA0206	VCA1011		Glutaredoxin		
2	repeat	224955	225088	+	gattatacccaaactacttggagttgcaggtaggcggcaagtgagttcatccc		fig|127906.87.repeat.60				repeat region		
2	CDS	225558	225091	-	atgagtaacgtcaatatgaaaa	MSNVNMKKPMVTDIFVEGAKI	fig|127906.87.peg.2925	VCCA0207	VCA1012	yjiG	Inner membrane protein YjiG		
2	CDS	226286	225555	-	atgagtcaatcgacagcgcagc	MSQSTAQERKVTWGCYIALAF	fig|127906.87.peg.2926	VCCA0208	VCA1013	yjiH	Uncharacterized protein YjiH	YjiH family protein [Vibrio cholerae]	
2	CDS	226678	228111	+	atgacgggccggatcgcactac	MTGRIALLSLTLFSPLSLASTP	fig|127906.87.peg.2927	VCCA0209	VCA1015	nhaD	Na+/H+ antiporter NhaD type		
2	CDS	228578	228177	-	atgaacgtggccaacttgaaac	MNVANLKPIILILSVLLLSGCFQ	fig|127906.87.peg.2928	VCCA0210	VCA1016		hypothetical protein		
2	CDS	229062	228589	-	atgagccattttactgactactca	MSHFTDYSSPLGPMTLQASS	fig|127906.87.peg.2929	VCCA0211	VCA1017	ogt	Methylated-DNA--protein-cysteine methyltransferase (EC 2.1.1.63)		
2	CDS	230421	229063	-	atgacattgcttaatgaattatccg	MTLLNELSAEVCQRARMSRDI	fig|127906.87.peg.2930	VCCA0212	VCA1018	alkA	Methylphosphotriester-DNA--protein-cysteine S-methyltransferase (EC 2.1.1.n11) / DNA-3-methyladenine glycosylase II (EC 3.2.2.21)		
2	CDS	230680	231123	+	atgaatactttattaaaaactgctt	MNTLLKTALTSPNSWAPLALR	fig|127906.87.peg.2931	VCCA0213	VCA1019	mhqP	Membrane protein, distant similarity to thiosulphate:quinone oxidoreductase DoxD		
2	CDS	232084	231200	-	atggacgtaaaagtctttcgcac	MDVKVFRTFLELAKVRHFGRA	fig|127906.87.peg.2932	VCCA0214	VCA1020		Transcriptional regulator VCA1020, LysR family		
2	CDS	232235	232594	+	atgtctgagacagcatttcgtcaa	MSETAFRQGKKRFFDTKKFPF	fig|127906.87.peg.2933	VCCA0215	VCA1021		hypothetical protein	DUF413 domain-containing protein [Vibrio cholerae]	Limited query cover for certain strains
									VCA1022				1 ORF split into 2 ORFs in N16961, both have refseq annotation in PATRIC and KEGG.
2	CDS	232673	234664	+	atgcccaacccagtttctaacgtt	MPNPVSNVYSLEEMLSLIQRE	fig|127906.87.peg.2934	VCCA0216	VCA1023		hypothetical protein	flavohemoglobin expression-modulating QEGLA motif protein [Vibrio cholerae]	1 ORF split into 2 ORFs in N16961, both have refseq annotation in PATRIC and KEGG.
2	CDS	235042	234791	-	atgaaaaatgaactggaaccga	MKNELEPSKVLSAYETVMENC	fig|127906.87.peg.2935	VCCA0217	VCA1024		hypothetical protein	DUF3081 domain-containing protein [Vibrio cholerae]	
2	CDS	235407	236207	+	atgagacttatcccactgaaagc	MRLIPLKAAAQVGKWAAAHIV	fig|127906.87.peg.2936	VCCA0218	VCA1025	nagB	Glucosamine-6-phosphate deaminase (EC 3.5.99.6)		
2	CDS	237495	236338	-	atgaatattatcggaattgcgatt	MNIIGIAIGATGLTLILIFVWMIV	fig|127906.87.peg.2937	VCCA0219	VCA1026		FOG: TPR repeat protein, SEL1 subfamily	sel1 repeat family protein [Vibrio cholerae]	
2	CDS	238484	237648	-	atggtgcttagagctttattacttg	MVLRALLLGGCIALAGCQSTA	fig|127906.87.peg.2938	VCCA0220	VCA1027		Maltose operon periplasmic protein MalM		
2	CDS	239927	238689	-	atgaaaaaagtaagtgtaattgc	MKKVSVIAAAVAATLAAGSAF	fig|127906.87.peg.2939	VCCA0221	VCA1028	lamB	Maltoporin (maltose/maltodextrin high-affinity receptor, phage lambda receptor protein)		
2	CDS	240365	242329	+	atgaacctgtcaacgcctttccca	MNLSTPFPLGATLDAEGCNFA	fig|127906.87.peg.2940	VCCA0222	VCA1029	glgX	Limit dextrin alpha-1,6-maltotetraose-hydrolase (EC 3.2.1.196)		
2	CDS	242464	242715	+	atgaaccccattctagcgatgct	MNPILAMLKENNISNEQINELF	fig|127906.87.peg.2941	VCCA0223	VCA1030		hypothetical protein	DUF2999 family protein [Vibrio cholerae]	
2	CDS	243175	244806	+	atggctttcttcaaaaatttagcga	MAFFKNLAIGKKIAVAFGVIALI	fig|127906.87.peg.2942	VCCA0224	VCA1031		Methyl-accepting chemotaxis sensor/transducer protein		1 ORF split into 2 ORFs in N16961, none have refseq annotation in PATRIC. Annotated as 1 pseudogene named VCA1031 in KEGG
									VCA1031				1 ORF split into 2 ORFs in N16961, none have refseq annotation in PATRIC. Annotated as 1 pseudogene named VCA1031 in KEGG
2	CDS	245328	244921	-	atggtgtggagaaaacgcacag	MVWRKRTAVLLGMVSLNAMG	fig|127906.87.peg.2943	VCCA0225	VCA1032		hypothetical protein		Limited query cover in N16961
2	CDS	246972	245596	-	atgacgaacaagtggattggag	MTNKWIGALGLLLSGQLMASE	fig|127906.87.peg.2944	VCCA0226	VCA1033	msmE	N-acetyl-D-glucosamine ABC transporter, substrate-binding protein		
2	CDS	248960	246981	-	atgggtttatctatcgtgcagcgc	MGLSIVQRIIAGFVLMLLLLILLC	fig|127906.87.peg.2945	VCCA0227	VCA1034		N-acetylglucosamine regulated methyl-accepting chemotaxis protein		
2	CDS	249164	249412	+	atgaacaacactacagcaaaa	MNNTTAKPTLPDRLSGNSRSF	fig|127906.87.peg.2946	VCCA0228	VCA1035		hypothetical protein	glutathione peroxidase [Vibrio cholerae]	
2	CDS	250558	249506	-	atgtttcaaactgatgatgtaaaa	MFQTDDVKIKKIKELLPPVAVL	fig|127906.87.peg.2947	VCCA0229	VCA1036	aroG	2-keto-3-deoxy-D-arabino-heptulosonate-7-phosphate synthase I alpha (EC 2.5.1.54)		Limited subject cover for certain strains
2	CDS	250860	251579	+	atgaaagtgatagagtttcacca	MKVIEFHQVSKWYGQFQALKS	fig|127906.87.peg.2948	VCCA0230	VCA1037		ABC transporter, ATP-binding protein (cluster 3, basic aa/glutamine/opines)		
2	CDS	251579	252376	+	atgataaaacgctttttccgccct	MIKRFFRPLLFAALQMMLVFA	fig|127906.87.peg.2949	VCCA0231	VCA1038	yecS	ABC transporter, permease protein (cluster 3, basic aa/glutamine/opines)		
2	CDS	252412	253248	+	atgcaattattcggacaacgttta	MQLFGQRLGRFSVKTLLTGLL	fig|127906.87.peg.2950	VCCA0232	VCA1039	fliY	ABC transporter, substrate-binding protein (cluster 3, basic aa/glutamine/opines)		
2	CDS	253249	254145	+	atgaataactatggcgcaaaatc	MNNYGAKSSSGYRFTLLDGG	fig|127906.87.peg.2951	VCCA0233	VCA1040	glnP	ABC transporter, permease protein (cluster 3, basic aa/glutamine/opines)		
2	CDS	254258	255961	+	atgaattcacaagtcacacactc	MNSQVTHWLARDPDPKTRQE	fig|127906.87.peg.2952	VCCA0234	VCA1041	pgcA	Phosphosugar mutase of unknown sugar (see annotation)	phospho-sugar mutase [Vibrio cholerae]	
2	CDS	256232	256020	-	gtgtttgccgaacatctaatcatta	MFAEHLIINGFFEGTAHAEKIQ	fig|127906.87.peg.2953	VCCA0235	VCA1042		Polymer-forming bactofilin		
2	CDS	257272	256331	-	atgaaagaaagaatgattgtttc	MKERMIVSVSSIRGTQHFYLG	fig|127906.87.peg.2954	VCCA0236	VCA1043		Peptidase, M23/M37 family		
2	CDS	257573	257716	+	atggcccgtgtaaaaaataaca	MARVKNNKNEIYINLKIIDKIKN	fig|127906.87.peg.2955	VCCA0237	VCA1044		FIG01199560: hypothetical protein		
2	CDS	257855	259804	+	atgatatcatcagacgccaagg	MISSDAKVKIQNFGRFLSNMV	fig|127906.87.peg.2956	VCCA0238	VCA1045	mtlA	PTS system, mannitol-specific IIC component / PTS system, mannitol-specific IIB component (EC 2.7.1.197) / PTS system, mannitol-specific IIA component (EC 2.7.1.197)		
2	CDS	259905	261059	+	atgaaaaagaatgcagttcattt	MKKNAVHFGAGNIGRGFIGKL	fig|127906.87.peg.2957	VCCA0239	VCA1046	mtlD	Mannitol-1-phosphate 5-dehydrogenase (EC 1.1.1.17)		
2	CDS	261159	261689	+	atggcagaaaaaattaacgaat	MAEKINESDILERLNQTHTVRC	fig|127906.87.peg.2958	VCCA0240	VCA1047	mtlR	Mannitol operon repressor		
2	CDS	261803	262738	+	atgaaaatcgctatgattggtctg	MKIAMIGLGDIAQKAYLPVLAQ	fig|127906.87.peg.2959	VCCA0241	VCA1048	yceM	Oxidoreductase, Gfo/Idh/MocA family		
2	CDS	263693	262839	-	atgaaacttactcgtacccttttga	MKLTRTLLTSALLLPAFSSLAA	fig|127906.87.peg.2960	VCCA0242	VCA1049		Metallo-beta-lactamase superfamily protein PA0057		
2	CDS	263908	264774	+	atggccgacaaactttccacctc	MADKLSTSALAKKRQQDAKQ	fig|127906.87.peg.2961	VCCA0243	VCA1050		hypothetical protein	glycerol kinase [Vibrio cholerae]	
2	CDS	265202	264771	-	atgttaagaacactaggcaaca	MLRTLGNIIWFLCGGIVMGLAW	fig|127906.87.peg.2962	VCCA0244	VCA1051	yccF	hypothetical protein	YccF domain-containing protein [Vibrio cholerae]	Limited query cover for certain strains
2	CDS	266000	265278	-	atggaatatttaggcgcgagtgc	MEYLGASAWIAMLLVFMGSFV	fig|127906.87.peg.2963	VCCA0245	VCA1052		hypothetical protein	sulfite exporter TauE/SafE family protein [Vibrio]	
2	CDS	266576	266181	-	atgtctagtgccgtatctcgtgtgc	MSSAVSRVPAASSADALAHFE	fig|127906.87.peg.2964	VCCA0246	VCA1053		Rhodanese-related sulfurtransferase		
2	CDS	267341	266703	-	atgaaagtagcaattctaggtgc	MKVAILGASGWIGSHLAAEAK	fig|127906.87.peg.2965	VCCA0247	VCA1054		Putative NADH-flavin reductase		
2	CDS	267474	268364	+	atggatcgcctaactgccatgcg	MDRLTAMRSFIEVANTASFTQ	fig|127906.87.peg.2966	VCCA0248	VCA1055		Transcriptional regulator, LysR family		
2	CDS	270719	268407	-	atgaaaattgccaacaaaatcg	MKIANKIVLSMTLLSIFSVVIAG	fig|127906.87.peg.2967	VCCA0249	VCA1056		Methyl-accepting chemotaxis sensor/transducer protein		
2	CDS	271828	271109	-	atgaaatcattagtcgttattacag	MKSLVVITGASSGIGEAIARRF	fig|127906.87.peg.2968	VCCA0250	VCA1057		Oxidoreductase, short-chain dehydrogenase/reductase family		
2	CDS	271976	272881	+	atgcaacttaaccaactcaatct	MQLNQLNLADIRAFVLIAKLGN	fig|127906.87.peg.2969	VCCA0251	VCA1058		Transcriptional regulator, LysR family		
2	CDS	272987	273592	+	atgcgcagttttatcctccgagct	MRSFILRARSAPTDSQRLLDE	fig|127906.87.peg.2970	VCCA0252	VCA1059		Uncharacterized DUF358 protein VCA1059	tRNA (pseudouridine(54)-N(1))-methyltransferase TrmY [Vibrio]	
2	CDS	274313	273657	-	atgaatcagtcctcattacttgcc	MNQSSLLAEFGDPITRVENAL	fig|127906.87.peg.2971	VCCA0253	VCA1060	ribB	3,4-dihydroxy-2-butanone 4-phosphate synthase (EC 4.1.99.12)		
2	CDS	274620	274754	+	gtgaaaagagttacggacaaa	MKRVTDKLVLNITAFEQEKSM	fig|127906.87.peg.2972	VCCA0254	VCA1061		hypothetical protein	histidine kinase [Vibrio cholerae]	
2	CDS	276091	274772	-	atgagtaaatccaataacaaaa	MSKSNNKIGVVQLTILTIVNMM	fig|127906.87.peg.2973	VCCA0255	VCA1062	potE	Putrescine/proton symporter, putrescine/ornithine antiporter PotE		
2	CDS	278321	276159	-	atgaattcattaaaaattgctacg	MNSLKIATSLSVRSCFTTQREY	fig|127906.87.peg.2974	VCCA0256	VCA1063	speF	Ornithine decarboxylase (EC 4.1.1.17)		
2	CDS	278462	278584	+	gtgaatattcttttgcttaataatga	MNILLLNNDFSWAYLEIHSVSV	fig|127906.87.peg.2975	VCCA0257	VCA1064		hypothetical protein		
2	CDS	278777	278661	-	atgaaaaagaaatcaaaaatca	MKKKSKIMAHIRRTRHIMMPSI	fig|127906.87.peg.2976	VCCA0258	Not annotated as CDS		hypothetical protein	DUF2618 domain-containing protein [Vibrio cholerae]	
2	CDS	279314	279075	-	ttgggcgctaatctttctgatgtcg	MGANLSDVAHYIKPASVWLDA	fig|127906.87.peg.2977	VCCA0259	VCA1066		hypothetical protein		
2	CDS	280746	279304	-	atggttcaactctctgtggtgtctc	MVQLSVVSRAESPEISSAIEAL	fig|127906.87.peg.2978	VCCA0260	VCA1067	calB	Aldehyde dehydrogenase (EC 1.2.1.3); Probable coniferyl aldehyde dehydrogenase (EC 1.2.1.68)		
2	repeat	280774	280895	+	aaactacttggagttgcaggtaggaggaaagtgagttcatccccatgagcat		fig|127906.87.repeat.61				repeat region		
2	CDS	281401	280919	-	atgagtctggatcgagttgaccg	MSLDRVDRDILRILHAKGRLPV	fig|127906.87.peg.2979	VCCA0261	VCA1068		PutR, transcriptional activator of PutA and PutP		
2	CDS	283488	281599	-	atgagattgagtttaaagagaaa	MRLSLKRKMVFSVVVAIAVTA	fig|127906.87.peg.2980	VCCA0262	VCA1069		Methyl-accepting chemotaxis sensor/transducer protein		
2	CDS	284069	283848	-	atgcctaaagcgtattgtcgtagt	MPKAYCRSCGKHTAHKMVMK	fig|127906.87.peg.2981	VCCA0263	VCA1070		hypothetical protein		
2	CDS	285743	284253	-	atggaaaatagcttcgctattaca	MENSFAITTTFIVYLILMLAIGV	fig|127906.87.peg.2982	VCCA0264	VCA1071	putP	Proline/sodium symporter PutP (TC 2.A.21.2.1) @ Propionate/sodium symporter		
2	CDS	286514	285816	-	atggtgcatcaagtaacccgtttt	MVHQVTRFSDAFSAWENWNL	fig|127906.87.peg.2983	VCCA0265	VCA1072		Delta 1-pyrroline-5-carboxylate dehydrogenase domain protein		
2	CDS	289655	286536	-	atgtttacagcaaccgatgtgttg	MFTATDVLNAAFIEQPLDKLW	fig|127906.87.peg.2984	VCCA0266	VCA1073	putA	Proline dehydrogenase (EC 1.5.5.2) / Delta-1-pyrroline-5-carboxylate dehydrogenase (EC 1.2.1.88)		
2	CDS	290858	289995	-	atggccgcgcaaatcccacctg	MAAQIPPDEVEMAQSHAPEA	fig|127906.87.peg.2985	VCCA0267	VCA1074		Transcriptional regulator, AraC family		
2	CDS	291250	292356	+	atgattaaacgtcgcatcccagc	MIKRRIPALLLALSPLWVSASV	fig|127906.87.peg.2986	VCCA0268	VCA1075		Chromosome segregation ATPase		
2	CDS	292516	294333	+	atgcgacaagggattcccgcttt	MRQGIPALLLALSPCLMAAPV	fig|127906.87.peg.2987	VCCA0269	VCA1077	pkn1	hypothetical protein	SUMF1/EgtB/PvdO family nonheme iron enzyme [Vibrio cholerae]	
2	CDS	294652	295392	+	gtgcctaaccatctgacattaga	MPNHLTLEQISLFKQLPGYWG	fig|127906.87.peg.2988	VCCA0270	VCA1078		Transcriptional regulator, LuxR family		
2	CDS	296056	295421	-	atggatctttcagatattcgccgc	MDLSDIRREYIHGGLRRKDLQ	fig|127906.87.peg.2989	VCCA0271	VCA1079	pdxH	Pyridoxamine 5'-phosphate oxidase (EC 1.4.3.5)		
2	CDS	296280	297671	+	atgagcaatcgcagttataacaa	MSNRSYNKLTAEELDYVDDKT	fig|127906.87.peg.2990	VCCA0272	VCA1080	prsE	Type I secretion system, membrane fusion protein LapC		
2	CDS	297680	298342	+	atgaaacgttggattgtgctgtct	MKRWIVLSLAICASSLSLALTS	fig|127906.87.peg.2991	VCCA0273	VCA1081		T1SS associated transglutaminase-like cysteine proteinase LapP		
2	CDS	298348	300258	+	atgacgctatacaaacaactag	MTLYKQLVAGMIAVFILLLISVF	fig|127906.87.peg.2992	VCCA0274	VCA1082		Membrane bound c-di-GMP receptor LapD		1 ORF split into 2 ORFs in N16961, both have refseq annotation in PATRIC. Annotated as 1 pseudogene named VCA1082 in KEGG
									VCA1083				1 ORF split into 2 ORFs in N16961, both have refseq annotation in PATRIC. Annotated as 1 pseudogene named VCA1082 in KEGG
2	CDS	300273	302387	+	atgcaagatacgttactcaattcc	MQDTLLNSLVYVSRYYGLANS	fig|127906.87.peg.2993	VCCA0275	VCA1084		Type I secretion system ATPase, LssB family LapB		
2	CDS	304192	302522	-	atgtggaaagagatatactcgaac	MWKEILELTPDRQKVIAKLGA	fig|127906.87.peg.2994	VCCA0276	VCA1085		Predicted polymerase, ortholog to Borrelia burgdorferi BB0267		
2	CDS	306157	304451	-	gtgagagtaatgatagtggatga	MRVMIVDDHGTNRELCRFILA	fig|127906.87.peg.2995	VCCA0277	VCA1086		Serine phosphatase RsbU, regulator of sigma subunit / Serine-protein kinase RsbW (EC 2.7.11.1)		
2	CDS	306480	306181	-	atgagtgtgcatacggacatcga	MSVHTDIDSHNKHITISIEGAF	fig|127906.87.peg.2996	VCCA0278	VCA1087		Anti-sigma B factor antagonist RsbV		
2	CDS	307644	306490	-	atgataacgagaaaacacaaa	MITRKHKITAILIGQILLLLVIFQ	fig|127906.87.peg.2997	VCCA0279	VCA1088		Methyl-accepting chemotaxis transducer domain protein		
2	CDS	308727	307687	-	atgaaaaaaatcaaagtcttgat	MKKIKVLIVDDSPVFRALLTQL	fig|127906.87.peg.2998	VCCA0280	VCA1089		Chemotaxis response regulator protein-glutamate methylesterase CheB (EC 3.1.1.61)		1 ORF split into 2 ORFs in N16961, none have refseq annotation in PATRIC. Annotated as 1 pseudogene named VCA1089 in KEGG
									VCA1089				1 ORF split into 2 ORFs in N16961, none have refseq annotation in PATRIC. Annotated as 1 pseudogene named VCA1089 in KEGG
2	CDS	309377	308724	-	atgtcagacagtaaacttgccac	MSDSKLATKRKLKQEEAKGQ	fig|127906.87.peg.2999	VCCA0281	VCA1090	cheD	Chemotaxis protein CheD		
2	CDS	310194	309364	-	atgttggcggtcaatgagcaaga	MLAVNEQEFELTDKDFKFIQW	fig|127906.87.peg.3000	VCCA0282	VCA1091		Chemotaxis protein methyltransferase CheR (EC 2.1.1.80)		
2	CDS	312245	310209	-	atggcattttggaatcgtaaacaa	MAFWNRKQQVVPQFVEPAKE	fig|127906.87.peg.3001	VCCA0283	VCA1092		Methyl-accepting chemotaxis protein III (mcp-III) (ribose and galactose chemoreceptor protein)		

2	CDS	312717	312259	-	atgcagcgtgaatttctcagtttc	MQREFLSFVLDDEEYGIPILEV	fig|127906.87.peg.3002	VCCA0284	VCA1093		Positive regulator of CheA protein activity (CheW)		
2	CDS	313244	312717	-	atgaattcagcgaatttgaccac	MNSANLTTSSSAALEPILSMDI	fig|127906.87.peg.3003	VCCA0285	VCA1094		Positive regulator of CheA protein activity (CheW)		
2	CDS	315415	313253	-	atggctttagatatggaacaact	MALDMEQLRKIFHVECRENLE	fig|127906.87.peg.3004	VCCA0286	VCA1095		Signal transduction histidine kinase CheA		
2	CDS	315823	315464	-	atggcaaaagttttggcagtaga	MAKVLAVDDSISIRQMVSHTL	fig|127906.87.peg.3005	VCCA0287	VCA1096		Chemotaxis regulator - transmits chemoreceptor signals to flagellar motor components CheY		
2	CDS	316103	315834	-	atgttgatactgaatgaagttctg	MLILNEVLDIAHVQECKQQLLF	fig|127906.87.peg.3006	VCCA0288	VCA1097		Anti-anti-sigma regulatory factor		
2	CDS	316373	317902	+	atgaaaaaaaggttgttcggtttg	MKKRLFGLILALGGAGIWLTKT	fig|127906.87.peg.3007	VCCA0289	VCA1098	nikA	ABC transporter, substrate-binding protein (cluster 5, nickel/peptides/opines)		
2	CDS	317895	318851	+	atgactaagattcttgttcaccgtt	MTKILVHRFYQALFVAWSVGT	fig|127906.87.peg.3008	VCCA0290	VCA1099	nikB	ABC transporter, permease protein 1 (cluster 5, nickel/peptides/opines)		
2	CDS	318853	319644	+	atgaaactgcacttatcgaaacc	MKLHLSKPQWLGVSLLILLVMI	fig|127906.87.peg.3009	VCCA0291	VCA1100	gsiD	ABC transporter, permease protein 2 (cluster 5, nickel/peptides/opines)		
2	CDS	319641	321023	+	gtgaccgcgctcctctctgtacaa	MTALLSVQQLAISTAQETLVES	fig|127906.87.peg.3010	VCCA0292	VCA1101		ABC transporter, ATP-binding protein (cluster 5, nickel/peptides/opines) / ABC transporter, ATP-binding protein (cluster 5, nickel/peptides/opines)		
2	CDS	321287	322930	+	atgccgacattcaacattaagcc	MPTFNIKPFSYLQLVFLLAAYF	fig|127906.87.peg.3011	VCCA0293	VCA1102	eptA	Lipid A phosphoethanolamine transferase, putative		
2	CDS	324425	323082	-	atgctggtctcgtttgggttcagtta	MLVSFGFSYSNAQHEVGEVYI	fig|127906.87.peg.3012	VCCA0294	VCA1104	qseC	Two-component system sensor histidine kinase		1 ORF split into 2 ORFs in N16961, none have refseq annotation in PATRIC. Annotated as 1 pseudogene named VCA1104 in KEGG
									VCA1104				1 ORF split into 2 ORFs in N16961, none have refseq annotation in PATRIC. Annotated as 1 pseudogene named VCA1104 in KEGG
2	CDS	325143	324487	-	atgcgtttactattagttgaagatg	MRLLLVEDDTLLGESMQVALS	fig|127906.87.peg.3013	VCCA0295	VCA1105	basR	Two-component transcriptional response regulator, LuxR family		
2	CDS	325780	325148	-	atgaacattatgcttaaatggatg	MNIMLKWMSLSALLLGYAHSS	fig|127906.87.peg.3014	VCCA0296	VCA1106		hypothetical protein	tetratricopeptide repeat protein [Vibrio cholerae]	1 ORF split into 2 ORFs in N16961, both have refseq annotation in PATRIC. Annotated as 1 pseudogene named VCA1106 in KEGG
									VCA1107				1 ORF split into 2 ORFs in N16961, both have refseq annotation in PATRIC. Annotated as 1 pseudogene named VCA1106 in KEGG
2	CDS	326605	325808	-	atggacattcatcaacaatatgtg	MDIHQQYVLLTGASGGIGEPIA	fig|127906.87.peg.3015	VCCA0297	VCA1108		Oxidoreductase, short-chain dehydrogenase/reductase family		
2	CDS	327298	326615	-	atgacaggatttttcaacgtttac	MTGFFQRLQKETQAAQQQML	fig|127906.87.peg.3016	VCCA0298	VCA1109		long-chain acyl-CoA synthetase		
2	CDS	328864	327341	-	atgaatcgaattctcgctgcgctg	MNRILAALQQHALHQPQKMAF	fig|127906.87.peg.3017	VCCA0299	VCA1110		Long-chain-fatty-acid--CoA ligase (EC 6.2.1.3)		
2	CDS	329477	328875	-	atgaaacctctaccttgtctagcc	MKPLPCLADLTLEVITPTHPRV	fig|127906.87.peg.3018	VCCA0300	VCA1111		hemolysin delta-VPH		
2	CDS	331777	329651	-	atgtttcctccttcttacgatgccttt	MFPPSYDAFQGVTAIGWACAI	fig|127906.87.peg.3019	VCCA0301	VCA1112		tRNA cytosine(34) acetyltransferase (EC 2.3.1.193)		
2	CDS	331905	332936	+	atgaaaactttcattaaaggattg	MKTFIKGLVLSAVVASSQVYAI	fig|127906.87.peg.3020	VCCA0302	VCA1113		Spermidine/putrescine import ABC transporter substrate-binding protein PotD (TC 3.A.1.11.1)		
2	CDS	333967	332996	-	atggcaattaaaacttctgaactg	MAIKTSELNAKLFGKADKRRA	fig|127906.87.peg.3021	VCCA0303	VCA1114	parB	Chromosome (plasmid) partitioning protein ParB		
2	CDS	335192	333975	-	atgaaaagagaacaaacgata	MKREQTIENLYQLAQLTQQVQ	fig|127906.87.peg.3022	VCCA0304	VCA1115	parA	Chromosome (plasmid) partitioning protein ParA		
2	CDS	335539	335405	-	ttgaatcctgcgcatcatttcaatc	MNPAHHFNQNGIHRNLVARFI	fig|127906.87.peg.3023	VCCA0305	Not annotated as CDS		hypothetical protein		
2	CDS	336427	338403	+	atgagctcagaagaaaaacga	MSSEEKRLIKLPRTHKDGHLFI	fig|127906.87.peg.3024	VCCA0306	VCA0002	rctB	hypothetical protein	UF3346 domain-containing protein [Vibrio cholerae]	
2	CDS	339102	338623	-	atgatcgagacttacggtatgaa	MIETYGMNELQHWLHTIHHWY	fig|127906.87.peg.3025	VCCA0307	VCA0003		hypothetical protein	transcriptional repressor [Vibrio cholerae]	
2	CDS	339447	339644	+	atgtccattaattctattgaccatg	MSINSIDHDDMTNIANKWDSIE	fig|127906.87.peg.3026	VCCA0308	VCA0004		hypothetical protein		
2	CDS	339807	340016	+	atgtctttcaaccttgcattactga	MSFNLALLTAAEKNRVELDKQ	fig|127906.87.peg.3027	VCCA0309	VCA0005		FIG018429: hypothetical protein	DUF3283 family protein [Vibrio cholerae]	
2	CDS	340104	340823	+	atgggaagaagttttgaagtgcg	MGRSFEVRKASMAKTQGAKIK	fig|127906.87.peg.3028	VCCA0310	VCA0006	yeeN	FIG007491: hypothetical protein YeeN	YebC/PmpR family DNA-binding transcriptional regulator [Vibrio cholerae]	
2	CDS	340938	341813	+	atgagagtcagctttattggatta	MRVSFIGLGVMGYPMAGHLQ	fig|127906.87.peg.3029	VCCA0311	VCA0007	garR	2-hydroxy-3-oxopropionate reductase (EC 1.1.1.60)		
2	CDS	343578	341917	-	atgagcctgacgattcgacaac	MSLTIRQRLYILSLVPLLTIALSI	fig|127906.87.peg.3030	VCCA0312	VCA0008		Methyl-accepting chemotaxis sensor/transducer protein		
					ttgtggctcacttcgttagttattatt	cccttttctcgttgaagcggtatcggtattggc	From N16961	Not annotated as CDS	VCA0009				
2	CDS	343856	344212	+	atgaaccttaaaaccgatattca	MNLKTDIQQLNNRIDTCRRKL	fig|127906.87.peg.3031	VCCA0313	VCA0010		hypothetical protein	MULTISPECIES: YibL family ribosome-associated protein [Vibrio]	
2	CDS	346987	344279	-	atgtggatcccttcgaaactgac	MWIPSKLTRPGRLHNAIVRPR	fig|127906.87.peg.3032	VCCA0314	VCA0011	malT	Transcriptional activator of maltose regulon, MalT		
2	CDS	347564	350017	+	atgaaacctactcaacagaaac	MKPTQQKQFDKKLFQENVKR	fig|127906.87.peg.3033	VCCA0315	VCA0013	malP	Glycogen phosphorylase (EC 2.4.1.1)		
2	CDS	350103	352283	+	atgaatcaagacaatgcattaa	MNQDNALKQVAAMAKIADRY	fig|127906.87.peg.3034	VCCA0316	VCA0014	malQ	4-alpha-glucanotransferase (amylomaltase) (EC 2.4.1.25)		
2	CDS	352489	354678	+	ttgaagatcacaaaaaaaccat	MKITKKPSKVQQFYDQLARAA	fig|127906.87.peg.3035	VCCA0317	VCA0015	glgB	1,4-alpha-glucan (glycogen) branching enzyme, GH-13-type (EC 2.4.1.18)		1 ORF split into 2 ORFs in N16961, both have refseq annotation in PATRIC. Annotated as 1 pseudogene named VCA1115 in KEGG
									VCA0016				1 ORF split into 2 ORFs in N16961, both have refseq annotation in PATRIC. Annotated as 1 pseudogene named VCA1115 in KEGG
2	repeat	354881	357367	+	tttttgcccgccaagcaagatctt	gctcagcgttgggcagtttttttgctttgctgat	fig|127906.87.repeat.62				repeat region		
2	CDS	355073	355591	+	atgccaactccatgttatatctcta	MPTPCYISIEGQTQGLITAGAC	fig|127906.87.peg.3036	VCCA0318	VCA0017		T6SS component Hcp		
2	CDS	355719	357803	+	atggcgacattagcgtacagca	MATLAYSIEVEGLEDETLVVR	fig|127906.87.peg.3037	VCCA0319	VCA0018		VgrG protein		
2	CDS	357803	358690	+	atgcgttcaacaaattcctttgtcg	MRSTNSFVVHNWVSLVPEME	fig|127906.87.peg.3038	VCCA0320	VCA0019		T6SS PAAR-repeat protein		
2	CDS	358687	361944	+	atgagtaatcccaatcaagctgg	MSNPNQAAKTGQTNDAQNPA	fig|127906.87.peg.3039	VCCA0321	VCA0020	vasX	Virulence factor VasX		
2	CDS	361945	362673	+	atgttaattgataaaaatgagtta	MLIDKNELEQLKVKLHSSEVIY	fig|127906.87.peg.3040	VCCA0322	VCA0021		Antitoxin TsiV2 to VasX		
2	CDS	362776	363177	+	atggtcacaacagggtatgccg	MVTTGYAVVLCGWLIYLAVQV	fig|127906.87.peg.3041	VCCA0323	VCA0022	yecN	Inner membrane protein YecN	glutathione S-transferase [Vibrio cholerae]	
2	repeat	363160	363288	+	tatccccttcctacttgaagctgca	gcggtgttggcttcgctcgttcaccccaat	fig|127906.87.repeat.63				repeat region		
2	CDS	364375	363350	-	atgtcaaaccatcagaaaatcg	MSNHQKIASLELGRVIAMLAIIA	fig|127906.87.peg.3042	VCCA0324	VCA0023	vanT	Fucose 4-O-acetylase		
2	CDS	365498	364506	-	atgcaaagcttgcgagcgtatat	MQSLRAYIQGIDMTDIIAQLQF	fig|127906.87.peg.3043	VCCA0325	VCA0024		Auxin efflux carrier family protein		
2	CDS	365776	367164	+	atgaacagaaacgatagtgtcc	MNRNDSVPLPTNTREWFLHR	fig|127906.87.peg.3044	VCCA0326	VCA0025		Sodium-dependent anion transporter family		
2	CDS	367895	367260	-	atgaaacctatgcagcgtctgac	MKPMQRLTCLLALCFAASASA	fig|127906.87.peg.3045	VCCA0327	VCA0026		putative secreted protein	DUF2057 domain-containing protein [Vibrio cholerae]	
2	CDS	368327	370876	+	atgaatcgaatgactttgtgcgc	MNRMTLCAASIACALASTAMA	fig|127906.87.peg.3046	VCCA0328	VCA0027	chiA	Chitinase (EC 3.2.1.14)		
2	CDS	371152	370898	-	atgagtgagaatgagagagaa	MSENEREIGQINQKNKADSVC	fig|127906.87.peg.3047	VCCA0329	VCA0028		hypothetical protein		
2	CDS	371242	371976	+	atgaacgaaacaaagatttcag	MNETKISANQVNEKSLQLLMC	fig|127906.87.peg.3048	VCCA0330	VCA0029		Transcriptional regulator, IclR family		
2	CDS	371983	372819	+	atgcttagcttattcaaacgctac	MLSLFKRYRLHRTSSHPTSTF	fig|127906.87.peg.3049	VCCA0331	VCA0030		Arginase/agmatinase/formimionoglutamate hydrolase, arginase family		
2	CDS	374505	372940	-	atgaagttaagcattagtcgtatt	MKLSISRILLLSVSILCTLALLSL	fig|127906.87.peg.3050	VCCA0332	VCA0031		Methyl-accepting chemotaxis sensor/transducer protein		
2	CDS	375163	374603	-	atgcacaaatctctggcactcttg	MHKSLALFILASLPVMANDYQ	fig|127906.87.peg.3051	VCCA0333	VCA0032		hypothetical protein		
2	CDS	375572	377446	+	atgtggaagcgtgatgagcaag	MWKRDEQESQFSEMIEAQLS	fig|127906.87.peg.3052	VCCA0334	VCA0033		Putative phosphatase		
2	CDS	378336	377506	-	atggtgaaaaacacaatgctca	MVKNTMLNRLNELPRLSKVLC	fig|127906.87.peg.3053	VCCA0335	VCA0034		hypothetical protein	HDOD domain-containing protein [Vibrio cholerae]	
2	CDS	378566	379318	+	atgaaagcacgcatcatgagct	MKARIMSCKRALILLLTFVLLLI	fig|127906.87.peg.3054	VCCA0336	VCA0035	pgpB	Phosphatidylglycerophosphatase B (EC 3.1.3.27)		
2	CDS	380589	379372	-	atgcagaacaactcgtttctagc	MQNNSFLARLVRGNLVLQILA	fig|127906.87.peg.3055	VCCA0337	VCA0036	sstT	Serine/threonine:Na+ symporter SstT		
2	CDS	381382	380930	-	atgaaactgaataccttgcttcttt	MKLNTLLLSSLLLSTSALAQSD	fig|127906.87.peg.3056	VCCA0338	VCA0037		Copper metallochaperone PCu(A)C, inserts Cu(I) into cytochrome oxidase subunit II		
2	CDS	381977	381396	-	atgagtaagaattggtcattgata	MSKNWSLILVVAFTLGFSIKAY	fig|127906.87.peg.3057	VCCA0339	VCA0038		Cytochrome oxidase biogenesis protein Sco1/SenC/PrrC, thiol-disulfide reductase involved in Cu(I) insertion into CoxII Cu(A) center		
2	CDS	382393	381974	-	atgaacgcactgaaagggattg	MNALKGIAILLALGAGFFASDL	fig|127906.87.peg.3058	VCCA0340	VCA0039		hypothetical protein		
2	CDS	383506	382595	-	atgaactacttgagcacctattta	MNYLSTYLKGLAMGAADVVP	fig|127906.87.peg.3059	VCCA0341	VCA0040		FIG028593: membrane protein	DUF368 domain-containing protein [Vibrio cholerae]	
2	CDS	384060	385286	+	atgaatcagatcatttcaattgga	MNQIISIGALESFLIAISVLFLGH	fig|127906.87.peg.3060	VCCA0342	VCA0041	gltS	Sodium/glutamate symporter		
2	CDS	386123	385365	-	atgaattcacggcccgctgaaa	MNSRPAEKIDNNDGQTETPRS	fig|127906.87.peg.3061	VCCA0343	VCA0042		hypothetical protein	flagellar brake protein [Vibrio cholerae]	
2	CDS	386255	386539	+	atggcaaatacagtgcaacatg	MANTVQHDANNHCYRVALEG	fig|127906.87.peg.3062	VCCA0344	VCA0043		Acetyltransferase		
2	repeat	386553	386683	+	ctatacccaaacaacttggagtt	gcaggtaggcggcaagtgagtgaatcctc	fig|127906.87.repeat.64				repeat region		
2	CDS	386772	390026	+	atgcatttgccgcattttgctctga	MHLPHFALSSVLSLTLCAIPNL	fig|127906.87.peg.3063	VCCA0345	VCA0044		Protease-related protein	protease [Vibrio cholerae]	1 ORF split into 2 ORFs in N16961, both have refseq annotation in PATRIC. Annotated as 1 pseudogene named VCA0044 in KEGG
									VCA0045				1 ORF split into 2 ORFs in N16961, both have refseq annotation in PATRIC. Annotated as 1 pseudogene named VCA0044 in KEGG
2	CDS	391351	390221	-	atggatttactgctgatcctgactt	MDLLLILTYTALCVAVFKVFKIF	fig|127906.87.peg.3064	VCCA0346	VCA0047	yiaV	Multidrug resistance efflux pump		
2	CDS	391740	391351	-	atgtttcttgattattttgcactcggg	MFLDYFALGLLIFVALVIFYGIIV	fig|127906.87.peg.3065	VCCA0347	VCA0048	yiaW	GTPase (EC 3.6.1.-)		
2	CDS	393455	391875	-	atggaaaccctgctcaataaga	METLLNKIKEAGLDAAAVAGEI	fig|127906.87.peg.3066	VCCA0348	VCA0049		GGDEF domain protein		1 ORF split into 2 ORFs in N16961, both have refseq annotation in PATRIC. Annotated as 1 pseudogene named VCA0049 in KEGG
									VCA0050				1 ORF split into 2 ORFs in N16961, both have refseq annotation in PATRIC. Annotated as 1 pseudogene named VCA0049 in KEGG
2	CDS	394923	393436	-	ttggacaaaagccaatatcttctt	MDKSQYLLELEIEQLRSRIESE	fig|127906.87.peg.3067	VCCA0349	VCA0051		hypothetical protein	ATP-dependent transcriptional regulator [Vibrio cholerae]	Limited query cover for certain strains
2	CDS	396547	395078	-	gtgcctaacgaccaactaccac	MPNDQLPPDAEGLQLNFCKTI	fig|127906.87.peg.3068	VCCA0350	VCA0052		hypothetical protein	IS1 family transposase [Vibrio cholerae]	
2	CDS	397322	396612	-	atggcaaccccacacattaacg	MATPHINAQPGDFAETVLMPG	fig|127906.87.peg.3069	VCCA0351	VCA0053	deoD	Purine nucleoside phosphorylase (EC 2.4.2.1)		
2	CDS	397644	398597	+	atgcagaccacgccactcaaa	MQTTPLKIGISACLLGAKVRFD	fig|127906.87.peg.3070	VCCA0352	VCA0055		COG1683: Uncharacterized conserved protein / FIG143828: Hypothetical protein YbgA	DUF523 and DUF1722 domain-containing protein [Vibrio cholerae]	
2	CDS	398587	399384	+	atggtttgtgatgaaaaacgttac	MVCDEKRYAIREVAEITGVKPV	fig|127906.87.peg.3071	VCCA0353	VCA0056	ycgE	Transcriptional regulator, MerR family, associated with photolyase		
2	CDS	399394	400803	+	atgagactggtttggtttcgtcgtg	MRLVWFRRDLRSFDNTALTA	fig|127906.87.peg.3072	VCCA0354	VCA0057	phrA	Deoxyribodipyrimidine photolyase (EC 4.1.99.3)		
2	CDS	400812	401732	+	atgtggtggaaagtttgggtaatc	MWWKVWVIGCALAVSWSINA	fig|127906.87.peg.3073	VCCA0355	VCA0058	ycfS	L,D-transpeptidase		
2	CDS	402066	401818	-	atgaacaagatgttaatcgcagc	MNKMLIAAAASSVLLLAGCAS	fig|127906.87.peg.3074	VCCA0356	VCA0059	lpp	Outer membrane lipoprotein	outer membrane lipoprotein [Vibrio albensis VL426]	
2	CDS	402374	402550	+	ttggctctcagcaaaggccagcc	MALSKGQPMDVQIFSRQICHS	fig|127906.87.peg.3075	VCCA0357	VCA0060		hypothetical protein		
2	CDS	402608	403954	+	ttgcaattcaaagatttaggcttag	MQFKDLGLDNRLLKNLAHYNF	fig|127906.87.peg.3076	VCCA0358	VCA0061		ATP-dependent RNA helicase VCA0061		
2	CDS	404167	406164	+	atgaaactgctcaatgtattgctg	MKLLNVLLLTTSLSVFYTYAET	fig|127906.87.peg.3077	VCCA0359	VCA0063	ptrB	Protease II (EC 3.4.21.83)		
2	CDS	406164	408305	+	atgaagctttctccagtatcagct	MKLSPVSAAVLSVLAAGFAHA	fig|127906.87.peg.3078	VCCA0360	VCA0064	fecA/hutR	TonB-dependent heme receptor HutR		
2	CDS	408315	410621	+	atgaaatcaacgttgaccttaatt	MKSTLTLIIASLLAPLASAAQW	fig|127906.87.peg.3079	VCCA0361	VCA0065		Hypothetical with regulatory P domain of a subtilisin-like proprotein convertase	proconvertase P-domain protein [Vibrio cholerae]	
2	CDS	410618	412060	+	atgaaatatcactactctttgatca	MKYHYSLISMALLMAGCGGEI	fig|127906.87.peg.3080	VCCA0362	VCA0066		Hypothetical protein in cluster with HutR, VCA0066 homolog	DUF1566 domain-containing protein [Vibrio cholerae]	
2	CDS	412057	412617	+	atgaaaaccctctcgactatggc	MKTLSTMATLLALSLPVMAQE	fig|127906.87.peg.3081	VCCA0363	VCA0067		Hypothetical protein in cluster with HutR, VCA0067 homolog	DUF1566 domain-containing protein [Vibrio cholerae]	
2	CDS	414394	412751	-	atgcgccagttactcagcggctt	MRQLLSGLSIKLQVVVPVFFTI	fig|127906.87.peg.3082	VCCA0364	VCA0068		Methyl-accepting chemotaxis sensor/transducer protein		
2	CDS	414765	415586	+	atgaaaaagacagtaatcggtg	MKKTVIGAIALMGALAVTPVMA	fig|127906.87.peg.3083	VCCA0365	VCA0070	pstS	Phosphate ABC transporter, substrate-binding protein PstS (TC 3.A.1.7.1)		
2	CDS	415664	416584	+	atgaccatcgcaacaaatagtg	MTIATNSDKLMDNTSMRSLRE	fig|127906.87.peg.3084	VCCA0366	VCA0071		Phosphate ABC transporter, permease protein PstC (TC 3.A.1.7.1)		
2	CDS	416590	417453	+	atggatcgcgtaaaactgaaac	MDRVKLKQARVLKDNILRTFIV	fig|127906.87.peg.3085	VCCA0367	VCA0072		Phosphate ABC transporter, permease protein PstA (TC 3.A.1.7.1)		
2	CDS	417482	418237	+	atgaaaacgaccaaattcaaca	MKTTKFNIENLDLFYGENQALI	fig|127906.87.peg.3086	VCCA0368	VCA0073		Phosphate ABC transporter, ATP-binding protein PstB (TC 3.A.1.7.1)		
2	CDS	419384	418284	-	atgaatacacaaactcagaaaa	MNTQTQKTLHPLILHAKTLLIFI	fig|127906.87.peg.3087	VCCA0369	VCA0074	yfiN	Transcriptional regulator CdgA		
2	CDS	419848	419381	-	atgagatggatattgctgatcttg	MRWILLILTLWCSSLALASNITI	fig|127906.87.peg.3088	VCCA0370	VCA0075		hypothetical protein	molybdopterin-dependent oxidoreductase [Vibrio cholerae]	
2	CDS	421515	420139	-	atgcaagataacgcgcaaacc	MQDNAQTLEPPAKAGTGNLW	fig|127906.87.peg.3089	VCCA0371	VCA0076		Predicted arginine uptake transporter		
2	CDS	423611	421950	-	atgtttgccatctatgaatcctata	MFAIYESYKAGQLQPKQWVNI	fig|127906.87.peg.3090	VCCA0372	VCA0077	dauA	Sulfate permease		
2	CDS	423949	424248	+	atgattaacaagaagttccttaa	MINKKFLKTKDEVEVTFECDAI	fig|127906.87.peg.3091	VCCA0373	VCA0078		1,4-alpha-glucan branching enzyme (EC 2.4.1.18)		
2	CDS	424482	425732	+	atgcgaaagaaagcgacacta	MRKKATLVGLPLLAVAAISSSL	fig|127906.87.peg.3092	VCCA0374	VCA0079		Cell wall endopeptidase, family M23/M37		Limited query cover for certain strains
2	CDS	427673	425850	-	atgaccaagactcaactgataa	MTKTQLITEKLSLDSIHHFSQL	fig|127906.87.peg.3093	VCCA0375	VCA0080		GGDEF domain protein		
2	CDS	427734	427838	+	atgcatactgttactattgatgag	MHTVTIDEVEEIFQPFDRENDS	fig|127906.87.peg.3094	VCCA0376	Not annotated as CDS		hypothetical protein		
2	CDS	428783	427896	-	atggactggctacagagcgcaa	MDWLQSAKTYIKVVEEGSFNC	fig|127906.87.peg.3095	VCCA0377	VCA0082		Transcriptional regulator, LysR family		
2	CDS	428873	430084	+	atgaacgcacaaacctctactcg	MNAQTSTRFRRTPMLLAMMIL	fig|127906.87.peg.3096	VCCA0378	VCA0083		Multidrug resistance transporter, Bcr/CflA family		
2	CDS	430572	430126	-	atggagatgacagtaggcgatg	MEMTVGDVAQRAGVKVSALH	fig|127906.87.peg.3097	VCCA0379	VCA0084	soxR	Redox-sensitive transcriptional activator SoxR		
2	CDS	430751	431953	+	atggaaaaaaataatacaacg	MEKNNTTRAGYYLTGLFFDGI	fig|127906.87.peg.3098	VCCA0380	VCA0085		Uncharacterized MFS-type transporter	MFS transporter [Vibrio cholerae]	
2	CDS	432084	432434	+	atgcaacagagtcaatttgaag	MQQSQFEALIKLLCEQATLPQ	fig|127906.87.peg.3099	VCCA0381	VCA0086		hypothetical protein		
2	CDS	432538	432672	+	atgaataatgaactcggttctgtt	MNNELGSVYLGQMIAKQAMH	fig|127906.87.peg.3100	VCCA0382	VCA0087		hypothetical protein		
2	CDS	434102	432801	-	atgaataccaaaaaaccgttatc	MNTKKPLSLTAKVILGMVAGIL	fig|127906.87.peg.3101	VCCA0383	VCA0088		Sodium/glutamate symporter		
2	CDS	435260	434367	-	atgtcgttggtgaacttgctgcaa	MSLVNLLQLLCLAAIWGGSFLI	fig|127906.87.peg.3102	VCCA0384	VCA0090	yijE	Permease of the drug/metabolite transporter (DMT) superfamily		
2	CDS	435490	435786	+	gtggctcgtcctaaaattccgcg	MARPKIPRRIECHPPASCFKPI	fig|127906.87.peg.3103	VCCA0385	VCA0091		HTH DNA-binding protein		
2	CDS	436586	435957	-	atgaagattgtaatactgcacgg	MKIVILHGLYMHGLVMQPLAQ	fig|127906.87.peg.3104	VCCA0386	VCA0092		Lipase		
2	CDS	436686	436859	+	atggagcagccgtgttggctacg	MEQPCWLRSVNPITWFKYVNI	fig|127906.87.peg.3105	VCCA0387	VCA0093		FIG01202475: hypothetical protein		
2	CDS	437087	437443	+	gtggacgtaaatatcaaatcacc	MDVNIKSPRIPINDTVALQQLE	fig|127906.87.peg.3106	VCCA0388	VCA0094	hxlR	Transcriptional regulator, HxlR family	helix-turn-helix transcriptional regulator [Vibrio cholerae]	Limited query cover for certain strains
2	CDS	437736	438410	+	atgttatttttatcgtttgttgttctatt	MLFLSFVVLFFLGISLLYFTYSS	fig|127906.87.peg.3107	VCCA0389	VCA0095		hypothetical protein		
2	CDS	438453	439676	+	atgaaaaaggcgtcattggcac	MKKASLALAVSVGLCSPAIWA	fig|127906.87.peg.3108	VCCA0390	VCA0096	lieA	hypothetical protein	endo-1,4-beta-xylanase [Vibrio cholerae]	
2	CDS	440405	439689	-	atgattgtcaccattgatatgatct	MIVTIDMICLRLAPKGIETLLVK	fig|127906.87.peg.3109	VCCA0391	VCA0097		Nudix-related transcriptional regulator NrtR		
2	CDS	440804	442111	+	atgaatccacgcctgttttctcccc	MNPRLFSPHIIRSLLDLDAYKIN	fig|127906.87.peg.3110	VCCA0392	VCA0098	pncB	Nicotinate phosphoribosyltransferase (EC 6.3.4.21)		
2	CDS	442142	443182	+	atgcccaataccccacttaaggt	MPNTPLKVGVIGYGYSAKTFH	fig|127906.87.peg.3111	VCCA0393	VCA0099	ydgJ	Predicted dehydrogenase	oxidoreductase [Vibrio cholerae]	
2	CDS	443575	443219	-	atgcgacaaaatggattaaaag	MRQNGLKALLALLSAWFSSGA	fig|127906.87.peg.3112	VCCA0394	VCA0100	pspE	Phage shock protein E		
2	CDS	445128	443590	-	gtgatttcaaaagcaccgatgag	MISKAPMRIAFAILLSPLIIFLLA	fig|127906.87.peg.3113	VCCA0395	VCA0101		Rtn protein		
2	CDS	445972	445322	-	atgaactttcaagccgctattttg	MNFQAAIFDMDGLLLDTERVC	fig|127906.87.peg.3114	VCCA0396	VCA0102		CbbY family protein		
2	CDS	446171	447940	+	gtgaaagcagtgtggataaggc	MKAVWIRQWFPGLYQFKDYQ	fig|127906.87.peg.3115	VCCA0397	VCA0103		Putative sulfate permease		
2	CDS	449694	447982	-	atgcgtctttgctttacccactctg	MRLCFTHSVICMSALPDCFTR	fig|127906.87.peg.3116	VCCA0398	VCA0104		Domain of unknown function / Pseudouridine synthase	Pseudouridylate synthase LSU rRNA-specific [Vibrio cholerae MJ-1236]	
2	CDS	450433	450717	+	atgggtaacggaatcgtagtag	MGNGIVVGHLGTDHDGFPPTI	fig|127906.87.peg.3117	VCCA0399	VCA0105		T6SS PAAR-repeat protein		
2	CDS	450701	451705	+	gtgtcaacattggttaagactctg	MSTLVKTLIFIVTLLFIGRSFSEI	fig|127906.87.peg.3118	VCCA0400	VCA0106		FIG00613524: hypothetical protein	sel1 repeat family protein [Vibrio cholerae]	
2	CDS	452137	452643	+	atgtctaaagaaggaagtgtag	MSKEGSVAPKERINIKYIPATG	fig|127906.87.peg.3119	VCCA0401	VCA0107		T6SS component TssB (ImpB/VipA)		
2	CDS	452684	454162	+	atgatgtctacgactgaaaaggt	MMSTTEKVLERPQLAQGSLLD	fig|127906.87.peg.3120	VCCA0402	VCA0108		T6SS component TssC (ImpC/VipB)		
2	CDS	454165	454602	+	atgacgtacatcgcacctgaag	MTYIAPEESAFGVGFFERLEAI	fig|127906.87.peg.3121	VCCA0403	VCA0109		T6SS lysozyme-like component TssE		
2	CDS	454608	456377	+	atgacgcaagacaagtatttcag	MTQDKYFREELAFLKEQGKEF	fig|127906.87.peg.3122	VCCA0404	VCA0110		T6SS component TssF (ImpG/VasA)		
2	CDS	456341	457357	+	atggggcacacagagcggaat	MGHTERNAAVDLNSDPAQAS	fig|127906.87.peg.3123	VCCA0405	VCA0111		T6SS component TssG (ImpH/VasB)		
2	CDS	457360	458847	+	atgaactcagtgacattaccttcg	MNSVTLPSLTLLVTNAQHLES	fig|127906.87.peg.3124	VCCA0406	VCA0112		T6SS forkhead associated domain protein ImpI/VasC		
2	CDS	458850	459326	+	gtgaacaaagtgctgtggatgct	MNKVLWMLFALTVLTACSSSD	fig|127906.87.peg.3125	VCCA0407	VCA0113		T6SS secretion lipoprotein TssJ (VasD)		
2	CDS	459333	460667	+	atgtttgcgcgtaaccgagtgatg	MFARNRVIWNEGLFIKPQHFQ	fig|127906.87.peg.3126	VCCA0408	VCA0114		T6SS component TssK (ImpJ/VasE)		
2	CDS	460670	461443	+	atgtcacagagtaaaaagagag	MSQSKKETPLASLLFDDVEKIN	fig|127906.87.peg.3127	VCCA0409	VCA0115		T6SS outer membrane component TssL (ImpK/VasF)		
2	CDS	461469	464078	+	gtgatccgtattgaattacccact	MIRIELPTLIAKLNAQSKLALEQ	fig|127906.87.peg.3128	VCCA0410	VCA0116		T6SS AAA+ chaperone ClpV (TssH)		
2	CDS	464081	465673	+	atgagtcaatggctggcgtttgca	MSQWLAFATQLVGVRKSHQL	fig|127906.87.peg.3129	VCCA0411	VCA0117		T6SS sigma-54-dependent regulator VasH		
2	CDS	465670	466326	+	atgagaattaccgttttagtggtca	MRITVLVVSLILGCAGSAHVLA	fig|127906.87.peg.3130	VCCA0412	VCA0118		Type VI secretion protein VasI		
2	CDS	466336	467745	+	atggaaatgactgaataccgtca	MEMTEYRQCVAKPISSSNPVC	fig|127906.87.peg.3131	VCCA0413	VCA0119		T6SS component TssA (ImpA)		
2	CDS	467761	471306	+	atgtggaaattcattgttggaata	MWKFIVGIVRRLKPTVVAALPI	fig|127906.87.peg.3132	VCCA0414	VCA0120		T6SS component TssM (IcmF/VasK)		
2	CDS	471357	472619	+	atgtccaatgtgatttttattgataa	MSNVIFIDNVCYRLTNDSEEIR	fig|127906.87.peg.3133	VCCA0415	VCA0121		Type VI secretion-related protein VasL		
					ttgattggggcaagggattgctca	gtgataagagttaaccctggtgcgaaaca	From N16961	Not annotated as CDS	VCA0122				
2	CDS	472872	475925	+	atggcaaggttacagtttcaatta	MARLQFQLKVDGLEDESLVVR	fig|127906.87.peg.3134	VCCA0416	VCA0123		VgrG protein		
2	CDS	475922	476290	+	atgaataacttgctttctgcgtatg	MNNLLSAYVTMLLILLSISGGA	fig|127906.87.peg.3135	VCCA0417	VCA0124		Antitoxin TsiV3 to VgrG		Limited query cover for certain strains
2	CDS	476698	476438	-	atgaaaaacactctgctgatgat	MKNTLLMIMSTLTLSACSEVGS	fig|127906.87.peg.3136	VCCA0418	VCA0125		Uncharacterized signal-peptide-containing protein VCA0125	DUF3012 domain-containing protein [Vibrio cholerae]	
2	CDS	476930	476799	-	ttggcgaaaaaatgcacatcga	MAKKCTSKRFDDIIVYSIKFAR	fig|127906.87.peg.3137	VCCA0419	Not annotated as CDS		FIG01202308: hypothetical protein		
2	CDS	476982	477401	+	atgaaaaaaagtaccctactaa	MKKSTLLNSELSYLVATLGHTI	fig|127906.87.peg.3138	VCCA0420	VCA0127	rbsD	D-ribose pyranase (EC 5.4.99.62)		
2	CDS	477428	478930	+	atgactcaagcaattttagccctg	MTQAILALSQIEKAFPGVKALD	fig|127906.87.peg.3139	VCCA0421	VCA0128	rbsA	Ribose ABC transporter, ATP-binding protein RbsA (TC 3.A.1.2.1)		
2	CDS	478945	479928	+	atgagtacccgaaccatgacta	MSTRTMTNTTSDNRKKLLSKE	fig|127906.87.peg.3140	VCCA0422	VCA0129	rbsC	Ribose ABC transporter, permease protein RbsC (TC 3.A.1.2.1)		
2	CDS	479989	480867	+	atgaaaaaactgaccactcttat	MKKLTTLISAALLSTSVSFVAQ	fig|127906.87.peg.3141	VCCA0423	VCA0130	rbsB	Ribose ABC transporter, substrate-binding protein RbsB (TC 3.A.1.2.1)		
2	CDS	481006	481926	+	atgaataagttggtggttctgggt	MNKLVVLGSVNADHVLQVPSI	fig|127906.87.peg.3142	VCCA0424	VCA0131	rbsK	Ribokinase (EC 2.7.1.15)		
2	CDS	481937	482941	+	atggccaccatgaaggatatcg	MATMKDIARLAGVSTSTVSHV	fig|127906.87.peg.3143	VCCA0425	VCA0132	rbsR	Ribose operon repressor		
2	CDS	483067	483633	+	atggatctgatgactttttttgatatt	MDLMTFFDINHTLVNIPIGGGY	fig|127906.87.peg.3144	VCCA0426	VCA0133	pnuC	Ribosyl nicotinamide transporter, PnuC-like		Limited subject cover for O395 because 2 ORF is joined into 1 ORF in N16961. According to KEGG N16961 VCA0133 is a pseudogene that codes for both ORFs
2	CDS	483679	483801	+	gtgtatgccctctctatcgaatac	MYALSIEYAILLFIAANGTRAWI	fig|127906.87.peg.3145	VCCA0427	VCA0133		Ribosyl nicotinamide transporter, PnuC-like		Limited subject cover for O395 because 2 ORF is joined into 1 ORF in N16961. According to KEGG N16961 VCA0133 is a pseudogene that codes for both ORFs
2	CDS	483798	484529	+	atgagcattcaacctcacattcat	MSIQPHIHVAQVAPRVVVCGE	fig|127906.87.peg.3146	VCCA0428	VCA0134		N-Ribosylnicotinamide phosphorylase (EC 2.4.2.1)		
2	CDS	484669	485349	+	atgcaacttaccgagaatactac	MQLTENTTGRFSGYLNAQLPA	fig|127906.87.peg.3147	VCCA0429	VCA0135		Predicted N-ribosylNicotinamide CRP-like regulator		
2	CDS	486469	485402	-	atgctaaaaccattctcgctttctc	MLKPFSLSLLALACSTSLFSSIV	fig|127906.87.peg.3148	VCCA0430	VCA0136	glpQ	Glycerophosphoryl diester phosphodiesterase (EC 3.1.4.46) periplasmic (secreted in GramPositives)		
2	CDS	487981	486623	-	atgttcgagctcttcaaaccggct	MFELFKPAAHTQRLPSDKVDS	fig|127906.87.peg.3149	VCCA0431	VCA0137	glpT	Glycerol-3-phosphate transporter		
2	CDS	488754	488407	-	atgaaaaagattattctcgccgtt	MKKIILAVTALTLSAGAFASIDT	fig|127906.87.peg.3150	VCCA0432	VCA0139		Acyl-CoA synthetase		
2	CDS	489201	490370	+	atgaaatacgggattaaaaatca	MKYGLKISALGSVILGVLAASQ	fig|127906.87.peg.3151	VCCA0433	VCA0140		Spindolin-related protein		
2	CDS	492506	490485	-	gtgattttactgctgatcgcggtgg	MILLLIAVRWGSHAVSENWQL	fig|127906.87.peg.3152	VCCA0434	VCA0141		C4-dicarboxylate transport sensor protein, putative		
2	repeat	492684	492814	+	gtataccttcctacttgaagctgc	agcggtgttggctacgttcgttgaccccaa	fig|127906.87.repeat.65				repeat region		

2	CDS	492829	494181	+	gtggaaagccaacggattgcat	MESQRIALIEDDDIVRQATGQ	fig|127906.87.peg.3153	VCCA0435	VCA0142		C4-dicarboxylate transport transcriptional regulatory protein		
2	CDS	494342	494217	-	atgagcaaaatcttgctgattga	MSKILLIEPKVHQQKTADHRA	fig|127906.87.peg.3154	VCCA0436	VCA0143		FIG01201365: hypothetical protein		Limited query/subject cover for certain strains
2	CDS	494483	495490	+	atgaaacaaaagcgccttacgc	MKQKRLTQTLLAAITSFGLIGG	fig|127906.87.peg.3155	VCCA0437	VCA0144		TRAP transporter solute receptor, TAXI family precursor		
2	CDS	495664	497805	+	atgagcaattcagtagaacaag	MSNSVEQELEKFEVPTRTRF	fig|127906.87.peg.3156	VCCA0438	VCA0146		TRAP-type uncharacterized transport system, fused permease component		
2	CDS	497802	499013	+	atgacttatgattacgtgattgttg	MTYDYVIVGGGIVGVSTGWQ	fig|127906.87.peg.3157	VCCA0439	VCA0147	lhgO	L-2-hydroxyglutarate oxidase (EC 1.1.3.15)		
2	CDS	499386	503129	+	atgaacaagaaaccattaacgc	MNKKPLTLLIAGLLGSTSAWA	fig|127906.87.peg.3158	VCCA0440	VCA0148		TagA-related protein		
2	CDS	503277	503783	+	atggctgttctagagattcttactg	MAVLEILTAPDPRLRVQSKQV	fig|127906.87.peg.3159	VCCA0441	VCA0150	def	Peptide deformylase (EC 3.5.1.88)		Limited query cover for certain strains
2	CDS	503933	505264	+	atgagcagggttaaatacactct	MSRVKYTLWVVVGIVTLLWV	fig|127906.87.peg.3160	VCCA0442	VCA0151	antC	Predicted ferric reductase	oxidoreductase [Vibrio cholerae]	
2	CDS	505662	505339	-	atgtcgatgttagcggccctctta	MSMLAALLLVLGTLFTLFASL	fig|127906.87.peg.3161	VCCA0443	VCA0152	mrpG	Na(+) H(+) antiporter subunit G		
2	CDS	505940	505662	-	gtggataattcattaccgctggcc	MDNSLPLAIHFAYLGLLLSLAM	fig|127906.87.peg.3162	VCCA0444	VCA0153	mnhF1	Na(+) H(+) antiporter subunit F		
2	CDS	506409	505933	-	atgatttatctcttcctcaacctctt	MIYLFLNLFLATAWMLLNGDY	fig|127906.87.peg.3163	VCCA0445	VCA0154	mrpE	Na(+) H(+) antiporter subunit E		
2	CDS	507902	506406	-	atgaccgcgattgggttgacctta	MTAIGLTLPVVISLLTAVAIFLA	fig|127906.87.peg.3164	VCCA0446	VCA0155	mrpD	Na(+) H(+) antiporter subunit D		
2	CDS	508252	507899	-	atggaaggattatggagcattgt	MEGLWSIVVGVFVAAGIYLML	fig|127906.87.peg.3165	VCCA0447	VCA0156	mrpC	Na(+) H(+) antiporter subunit C		
2	CDS	510927	508252	-	ttgaagacaggactcgataagc	MKTGLDKHTSLIPNTVSARLLF	fig|127906.87.peg.3166	VCCA0448	VCA0157	mrpA	Na(+) H(+) antiporter subunit A / Na(+) H(+) antiporter subunit B		
2	CDS	511271	511074	-	atgaaaaagttctgcccacactg	MKKFCPHCHCEMLERANLYIC	fig|127906.87.peg.3167	VCCA0449	VCA0158		hypothetical protein		
2	CDS	512287	511346	-	atgaaaaaatttaagaatatctta	MKKFKNILFATQGLPGHSDAL	fig|127906.87.peg.3168	VCCA0450	VCA0159		Universal stress protein family 1		
2	CDS	513697	512483	-	atgtcgaaacaaccctcactgttt	MSKQPSLFGGACIIASVCVGA	fig|127906.87.peg.3169	VCCA0451	VCA0160	mtr	Tryptophan-specific transport protein		
2	CDS	515215	513797	-	atggaaaattttaaacacttacca	MENFKHLPEPFRIRVIEPVKRT	fig|127906.87.peg.3170	VCCA0452	VCA0161	tnaA	Tryptophanase (EC 4.1.99.1)		
2	CDS	515530	515625	+	atgctcatggggatgaacaccc	MLMGMNTLAYYLQFQVVWVC	fig|127906.87.peg.3171	VCCA0453	Not annotated as CDS		FIG01201164: hypothetical protein		
2	CDS	515945	515781	-	gtgagaaaagtaaatatcttcct	MRKVNIFLHLDAEKNSGINVIH	fig|127906.87.peg.3172	VCCA0454	VCA0162		hypothetical protein		
2	CDS	515996	516208	+	atgtttgagaaaacgcatcttata	MFEKTHLIKLAQMKMPFGKYA	fig|127906.87.peg.3173	VCCA0455	VCA0163		Uncharacterized protein YpeB	DNA polymerase III subunit epsilon [Vibrio cholerae]	
2	CDS	516760	516278	-	atgcgtcttttctctgccgtactgct	MRLFSAVLLLLIFPNLSYAQV	fig|127906.87.peg.3174	VCCA0456	VCA0164		L,D-transpeptidase		
2	CDS	517169	519301	+	atgtttcctcgaaccctactcaaa	MFPRTLLKRVTSSRNYRMLAE	fig|127906.87.peg.3175	VCCA0457	VCA0165		Aerotaxis sensor receptor protein, diguanylate cyclase		
2	CDS	519547	519759	+	atgtctcagaaaatgactggttca	MSQKMTGSVKWFNETKGFGF	fig|127906.87.peg.3176	VCCA0458	VCA0166	cspA	Cold shock protein of CSP family		
2	CDS	520765	520049	-	atgaccaatatcaataccaaag	MTNINTKAATVLGLSLIIGLSAL	fig|127906.87.peg.3177	VCCA0459	VCA0167		Putative periplasmic protein	SIMPL domain-containing protein [Vibrio cholerae]	
2	CDS	522578	520926	-	atggtaaagatacacgcaccgt	MVKIHAPFQSVLFWLTLLVSLL	fig|127906.87.peg.3178	VCCA0460	VCA0168	batD	Aerotolerance protein BatD		ORF split into 3 ORFs VCA0168-VCA0170 in PATRIC. annotated as 1 ORF coding for pseudogene named VCA0168 in KEGG.
									VCA0169				ORF split into 3 ORFs VCA0168-VCA0170 in PATRIC. annotated as 1 ORF coding for pseudogene named VCA0168 in KEGG.
									VCA0170				ORF split into 3 ORFs VCA0168-VCA0170 in PATRIC. annotated as 1 ORF coding for pseudogene named VCA0168 in KEGG.
2	CDS	524470	522572	-	atgtcatcgttgattttctctaccc	MSSLIFLYPHWLGLLVPLLLLA	fig|127906.87.peg.3179	VCCA0461	VCA0171	batB	Aerotolerance protein BatB / Aerotolerance protein BatC		Limited query/subject cover for certain strains
2	CDS	525428	524472	-	ttggcaagttttgagtttatttggtg	MASFEFIWWWMLFFLPLPWV	fig|127906.87.peg.3180	VCCA0462	VCA0172	batA	Aerotolerance protein BatA		
2	CDS	525903	525415	-	atgaaggaatcgtcaaccacat	MKESSTTSLDLNGLHLPELPS	fig|127906.87.peg.3181	VCCA0463	VCA0173		FIG00657500: hypothetical protein	DUF4381 domain-containing protein [Vibrio cholerae]	
2	CDS	526835	525900	-	atgacgatgccacacgccctcc	MTMPHALPLHANGVSLCLEEL	fig|127906.87.peg.3182	VCCA0464	VCA0174		hypothetical protein PA3071		
2	CDS	527795	526839	-	atgtcgtttcaatctttcaaacttt	MSFQSFQTLQHYLNQQVIGQ	fig|127906.87.peg.3183	VCCA0465	VCA0175		MoxR-like ATPase in aerotolerance operon		
2	CDS	528098	530014	+	atgttcaaactgcatcagatgag	MFKLHQMSIKQKVVLGVTLAV	fig|127906.87.peg.3184	VCCA0466	VCA0176		Methyl-accepting chemotaxis sensor/transducer protein		
2	CDS	530071	530274	+	gtgatgaacgatttcgaaaaag	MMNDFEKELELMSQQVAEEP	fig|127906.87.peg.3185	VCCA0467	VCA0177		Chromosome segregation ATPase		
2	repeat	530344	530471	+	tatacccaaaccacttggagttgcaggtaggcggcaagtgagtaagtcccc		fig|127906.87.repeat.66				repeat region		
2	CDS	531203	530550	-	atgcacaagctaacgatagatc	MHKLTIDLVSDIVCPWCVIGYF	fig|127906.87.peg.3186	VCCA0468	VCA0178		frnE protein		
2	CDS	531849	533057	+	atggcgattttgtttggaatcatcg	MAILFGIIGVTVLILCAYLLSESF	fig|127906.87.peg.3187	VCCA0469	VCA0179	psuT	Na+ dependent nucleoside transporter NupC		
2	CDS	533308	534540	+	atgaaaaatcttgttgggcgcttt	MKNLVGRFMRYVTFDTQSKP	fig|127906.87.peg.3188	VCCA0470	VCA0180	pepT	Tripeptide aminopeptidase (EC 3.4.11.4)		
2	CDS	534712	535587	+	atggaacatctgagcatagcgg	MEHLSIAVDFLLTHKLLLTALIL	fig|127906.87.peg.3189	VCCA0471	VCA0181		Small-conductance mechanosensitive channel		
2	CDS	537176	535584	-	atgcaagaattctccttgagtact	MQEFSLSTLLEMTVGLASGAN	fig|127906.87.peg.3190	VCCA0472	VCA0182		Anaerobic nitric oxide reductase transcription regulator NorR		
2	CDS	537367	538551	+	atgctcacccaagaacacatca	MLTQEHINIIKSTIPLLESAGPA	fig|127906.87.peg.3191	VCCA0473	VCA0183	hmp/hmpA	Flavohemoglobin / Nitric oxide dioxygenase (EC 1.14.12.17)		
2	CDS	538904	538695	-	atgtctactccagttactggtacc	MSTPVTGTVKWFNETKGFGF	fig|127906.87.peg.3192	VCCA0474	VCA0184		Cold shock protein of CSP family		
2	CDS	539263	539490	+	atgaaaaacaaaccagaagta	MKNKPEVIPVSTSSVGDTECG	fig|127906.87.peg.3193	VCCA0475	VCA0185		Alternative ribosome-rescue factor A		
2	CDS	539878	540255	+	atgaaactgtttttaattctcatgct	MKLFLILMLSVSGGIASADHIQ	fig|127906.87.peg.3194	VCCA0476	VCA0186		NADH:ubiquinone oxidoreductase subunit 2		
2	CDS	540403	540281	-	atgagccaagccagtaagcgtg	MSQASKRGDSFKSASKENKQ	fig|127906.87.peg.3195	VCCA0477	VCA0187		hypothetical protein		
2	CDS	540529	540768	+	atggagatttgtgctgtttggattg	MEIVLFWIEKLILVSGILLVLTG	fig|127906.87.peg.3196	VCCA0478	VCA0188		hypothetical protein		
2	CDS	540855	541859	+	atgtctttccctgttctcatctgtga	MSFPVLICDDSPLARKQMARS	fig|127906.87.peg.3197	VCCA0479	VCA0189	rvsC	Chemotaxis protein CheC -- inhibitor of MCP methylation		
2	CDS	541859	542305	+	atgctagcgttacctgcggagttt	MLALPAEFEQFHWMVDMVQN	fig|127906.87.peg.3198	VCCA0480	VCA0190		hypothetical protein	PAS domain-containing protein [Vibrio cholerae]	
2	CDS	542355	543827	+	atgccgttacccactcattttata	MPLPTHFYTTQQLKQGEQDA	fig|127906.87.peg.3199	VCCA0481	VCA0191	nnr	NAD(P)H-hydrate epimerase (EC 5.1.99.6) / ADP-dependent (S)-NAD(P)H-hydrate dehydratase (EC 4.2.1.136)		
2	CDS	544811	543816	-	atgctcaacgtcattttttagtgc	MLNVIFFSAKHYDIASFSKLVD	fig|127906.87.peg.3200	VCCA0482	VCA0192	ldhA	D-lactate dehydrogenase (EC 1.1.1.28)		
2	CDS	545054	546337	+	atggtttcttactgcttttatgggc	MVFLLLFMGVGSYLTWQGVE	fig|127906.87.peg.3201	VCCA0483	VCA0193	nhaC	Methionine transporter MetT		
2	CDS	547181	546339	-	atgaaactcaccctcgcaaaa	MKKLTLRKKLLEKVENTTETPM	fig|127906.87.peg.3202	VCCA0484	VCA0194	kcsA	Potassium voltage-gated channel subfamily KQT; possible potassium channel, VIC family		
2	CDS	547795	547247	-	atggtacgtttttgcggctggttaa	MVRFCGWLMVFCCFALPAYA	fig|127906.87.peg.3203	VCCA0485	VCA0195		hypothetical protein	porin family protein [Vibrio cholerae]	
2	CDS	548453	549496	+	atgcgtatcgaacaagaacttac	MRIEQELKLGFKDVLFRPKRS	fig|127906.87.peg.3204	VCCA0486	VCA0197	guaC	GMP reductase (EC 1.7.1.7)		
2	CDS	550000	550971	+	atgggcttacctaagccgaaata	MGLPKPKYGHYLGSIDDFVTG	fig|127906.87.peg.3205	VCCA0487	VCA0198	haeIIIM	site-specific DNA-methyltransferase, putative		
2	CDS	551095	553011	+	atgaaacttgaagaagttttagg	MKLEEVLGYLEQMVGLKLQPI	fig|127906.87.peg.3206	VCCA0488	VCA0199		hypothetical protein	DUF262 domain-containing protein [Vibrio cholerae]	
2	CDS	553004	554128	+	atgattaacgaagtaactttaag	MINEVTLRNFKCYKNQLFELAI	fig|127906.87.peg.3207	VCCA0489	VCA0200		hypothetical protein	DUF3696 domain-containing protein [Vibrio cholerae]	
2	CDS	554125	555003	+	atgaaaactatagtttccgtttcac	MKTIVSVSNFSLDSCKFSSFM	fig|127906.87.peg.3208	VCCA0490	VCA0201		hypothetical protein		
2	repeat	555005	555633	+	tataaaaatccgccttcttagaaggcggttttaatataagccccctaaaaggg		fig|127906.87.repeat.67				repeat region		
2	CDS	555508	555071	-	atgggcgactacagaagttcatc	MGDYRSSSHVYWRCKYHIVW	fig|127906.87.peg.3209	VCCA0491	VCA0202		Mobile element protein		
					ttgaaggaaaagaaagcggcttatagccgctcttttattgtcatcctaaacca		From N16961	Not annotated as CDS	VCA0203				
2	CDS	557368	556013	-	atgggttttacctcgctcggtctgt	MGFTSLGLSAPILKAIEEKGYN	fig|127906.87.peg.3210	VCCA0492	VCA0204		ATP-dependent RNA helicase RhlE (EC 3.6.4.13)		
2	CDS	558886	557558	-	atgctatatgtagaatttcttttcta	MLYVEFLFLLLMLYIGSRYGGI	fig|127906.87.peg.3211	VCCA0493	VCA0205	dcuB	Anaerobic C4-dicarboxylate transporter DcuB		
2	CDS	559128	558997	-	ttgacctacatcaagccaagcc	MTYIKPSLSLGLIVKISPCLTQ	fig|127906.87.peg.3212	VCCA0494	Not annotated as CDS		FIG01200124: hypothetical protein		
2	CDS	559967	559137	-	atggaacacaagatccgtgaag	MEHKIREEMRVLPSIDPQFEIE	fig|127906.87.peg.3213	VCCA0495	VCA0207	nadE	NAD synthetase (EC 6.3.1.5)		
2	CDS	560070	560597	+	atgaaaaagattgcggtgtttgg	MKKIAVFGSAFNPPTLGHKSII	fig|127906.87.peg.3214	VCCA0496	VCA0208	nadD	Nicotinate-nucleotide adenylyltransferase (EC 2.7.7.18)		
					ttgagcgcctttgcaccagcagcagctttacactagcccaagcttctaaatttt		From N16961	Not annotated as CDS	VCA0209				
2	CDS	560864	562405	+	atggatctgtttgcagatatgcg	MDLFADMRQEAAGEKERVVM	fig|127906.87.peg.3215	VCCA0497	VCA0210		Response regulator		
2	CDS	564409	562463	-	atgaaaaaccgtgacgcagaa	MKNRDAEEMDSNPMFSRIGR	fig|127906.87.peg.3216	VCCA0498	VCA0211	gchK	Two-component system sensor histidine kinase		
2	CDS	564659	565633	+	atgcgctggtggatggttatagtg	MRWWMVIVWLLSGQAWAAN	fig|127906.87.peg.3217	VCCA0499	VCA0212		ABC transporter or sensor sytem substrate-binding protein		
2	CDS	567291	565711	-	atgaatttagcccctattctgact	MNLAPYSDSVYSVIPPLLAVLL	fig|127906.87.peg.3218	VCCA0500	VCA0213		Predicted lysine transporter, NhaC family		
2	CDS	568900	567695	-	atgtctgcctcacttctgtcgcaa	MSASLSVAKLTFFIAILAAVGQ	fig|127906.87.peg.3219	VCCA0501	VCA0214	emrD	Multidrug resistance transporter, Bcr/CflA family		
2	CDS	569098	568940	-	ttggttcaaggcacaataaaac	MVQGTIKPKFALEKIIKAIDIYRI	fig|127906.87.peg.3220	VCCA0502	Not annotated as CDS		hypothetical protein	Permease [Vibrio cholerae RC385]	
2	CDS	569153	569419	+	atgttgtcacttgctgtaccactac	MLSLAVPLLFMSLLGFKLKLPY	fig|127906.87.peg.3221	VCCA0503	VCA0216		hypothetical protein		
2	CDS	570489	569410	-	atgacgatgacaaaatcgactc	MTMTKSTHSPAFTGSELLNTY	fig|127906.87.peg.3222	VCCA0504	VCA0217		GGDEF domain protein		
2	CDS	571866	570610	-	atgaaaaaaagactctctattcta	MKKRLSILIAGLASLSVNAATE	fig|127906.87.peg.3223	VCCA0505	VCA0218		Thermolabile hemolysin precursor		
2	CDS	572836	575061	+	atgccaaaactcaatcgttgcg	MPKLNRCAIAIFTILSAISSPTL	fig|127906.87.peg.3224	VCCA0506	VCA0219	hlyA	Cytolysin and hemolysin, HlyA, Pore-forming toxin		
2	CDS	575360	577006	+	atgatcatcaataaatttccctta	MIINKFSLKWMLAIAVAIPAIAL	fig|127906.87.peg.3225	VCCA0507	VCA0220	hlyB	Methyl-accepting chemotaxis protein, hemolysin secretion protein HylB		
2	CDS	577354	578292	+	ttgaataaaatcattatattaatcg	MNKIIILIALSLFSSSIWAGTSAI	fig|127906.87.peg.3226	VCCA0508	VCA0221	hlyC	Lipase precursor (EC 3.1.1.3)		
2	CDS	578302	579156	+	atgaaaaaaatcgcatggagtt	MKKIAWSLGILVTIGALCAIVW	fig|127906.87.peg.3227	VCCA0509	VCA0222		Lipase activator protein, Lipase-specific foldase		
2	CDS	581988	579232	-	atgaaaacgatcaaaaaaacg	MKTIKKTLLAAAIASFFSSGLYA	fig|127906.87.peg.3228	VCCA0510	VCA0223	ina/prtV	Metalloprotease, putative zinc-binding domain		
2	CDS	582345	582073	-	atgcgcaccctatttggattatgg	MRTLFGLWVICYSTLLFANAQ	fig|127906.87.peg.3229	VCCA0511	VCA0224		hypothetical protein		
2	CDS	582938	583696	+	atgtgtcgctggctggcttatcaa	MCRWLAYQGSPIYLEQLVYQF	fig|127906.87.peg.3230	VCCA0512	VCA0225	egtC	Glutamine amidotransferases class-II		
2	repeat	583703	583895	+	ccctcctacttgaagctgcagcggtgttggctacgttagttctccccaaccacac		fig|127906.87.repeat.68				repeat region		
2	CDS	583978	583856	-	ttgaacaatttagaaatgcacaa	MNNLEMHKPSPILINSKGLQD	fig|127906.87.peg.3231	VCCA0513	Not annotated as CDS		hypothetical protein		
2	CDS	584102	585013	+	atgaaatctcgtattcattgggctg	MKSRIHWAALGLLAAFAAQAE	fig|127906.87.peg.3232	VCCA0514	VCA0227	yclQ/vctP	Ferric vibriobactin, enterobactin transport system, substrate-binding protein VctP (TC 3.A.1.14.6)		
2	CDS	585066	586004	+	ttgaaaaagttattgcttgttttagt	MKKLLLVLVVISFCSLFVGVGN	fig|127906.87.peg.3233	VCCA0515	VCA0228	feuB/vctD	Ferric vibriobactin, enterobactin transport system, permease protein VctD (TC 3.A.1.14.6)		
2	CDS	585994	586944	+	atgcaggatagaacgaaattac	MQDRTKLLLLIAISLLFAALFIG	fig|127906.87.peg.3234	VCCA0516	VCA0229	vctG	Ferric vibriobactin, enterobactin transport system, permease protein VctG (TC 3.A.1.14.6)		
2	CDS	586947	587711	+	atgattcaactggaaaagctcac	MIQLEKLTKHFGTKRVVHDAS	fig|127906.87.peg.3235	VCCA0517	VCA0230	vctC	Ferric vibriobactin, enterobactin transport system, ATP-binding protein (TC 3.A.1.14.6)		
2	CDS	588773	587757	-	atgcaaaatcgcacatcagttat	MQNRTSVMENTQNQHKVLTK	fig|127906.87.peg.3236	VCCA0518	VCA0231	soxS	Transcriptional regulator, VCA0231 ortholog	AraC family transcriptional regulator [Vibrio cholerae]	
2	CDS	588860	590839	+	atggatatttgcaagaagtctgc	MDICKKSALWLAITAAMSANA	fig|127906.87.peg.3237	VCCA0519	VCA0232	bhuA	Enterobactin receptor VctA		1 ORF split into 2 ORFs in N16961, none refseq annotated in PATRIC. Annotated as 1 pseudogene ORF named VCA0232 in KEGG
									VCA0232				1 ORF split into 2 ORFs in N16961, none refseq annotated in PATRIC. Annotated as 1 pseudogene ORF named VCA0232 in KEGG
2	CDS	590897	591217	+	atgtcgtctcaatttcaagcgttgt	MSSQFQALYQSQATLHSEHV	fig|127906.87.peg.3238	VCCA0520	VCA0233		Hypothetical protein colocalized with Enterobactin receptor VctA	DUF2218 domain-containing protein [Vibrio cholerae]	
2	CDS	591487	592680	+	atgtcgaactcgtatgtgttggtca	MSNSYVLVINSGSSSLKFAVID	fig|127906.87.peg.3239	VCCA0521	VCA0235		Acetate kinase (EC 2.7.2.1)		
2	CDS	592761	593057	+	gtgcaaggcttacgcactgtaac	MQGLRTVTQQTDLTEITKAWF	fig|127906.87.peg.3240	VCCA0522	VCA0236		hypothetical protein		
2	CDS	593744	593175	-	atgcaacgtatgactcgtactcta	MQRMTRTLSVSALILCGLLPF	fig|127906.87.peg.3241	VCCA0523	VCA0237		putative phosphoribosyl-AMP cyclohydrolase		
2	CDS	594028	595638	+	atgtttcgatatcggcttgcgtttgt	MFRYRLAFVLALLTLILAQVG	fig|127906.87.peg.3242	VCCA0524	VCA0238	yccG	Two-component system sensor histidine kinase		
2	CDS	595619	596293	+	atgcgaatcttattgattgaagat	MRILLIEDDERIARFIERGLKAE	fig|127906.87.peg.3243	VCCA0525	VCA0239	czcR	Two-component transcriptional response regulator VC_A0239, OmpR family		
2	CDS	596727	596296	-	atgaaattactccaccttcagctg	MKLLHLQLFWYEKHHTLLELE	fig|127906.87.peg.3244	VCCA0526	VCA0240		hypothetical protein		
2	CDS	597769	596885	-	atgtatcaacatttactgcgccac	MYQHLLRHRVGLYEKALPNHL	fig|127906.87.peg.3245	VCCA0527	VCA0241	ulaE	L-xylulose 5-phosphate 3-epimerase (EC 5.1.3.-)		
2	CDS	598428	597781	-	atgaccaaaccaatgattcaaa	MTKPMIQIALDQTNLTDAVAVA	fig|127906.87.peg.3246	VCCA0528	VCA0242	sgbH/ulaD	3-keto-L-gulonate 6-phosphate decarboxylase		
2	CDS	599255	598434	-	atgtataaagtgctgcctttggat	MYKVLALDLDGTVLSDDHTIHI	fig|127906.87.peg.3247	VCCA0529	VCA0243	ybhA	Predicted hydrolase	Cof-type HAD-IIB family hydrolase [Vibrio cholerae]	
2	CDS	599953	599261	-	atgtacagcgaactgaaacagc	MYSELKQRVFNANLKLPKYGL	fig|127906.87.peg.3248	VCCA0530	VCA0244	ulaF/araD	L-ribulose-5-phosphate 4-epimerase (EC 5.1.3.4)		
2	CDS	600468	599962	-	atgggactcaaacaatccttaat	MGLKQSLIENNSIQLQAKANN	fig|127906.87.peg.3249	VCCA0531	VCA0245	ulaC	PTS system, ascorbate-specific IIA component		
2	CDS	602326	600566	-	atggagttcttgtacgacgttttcta	MEFLYDVFYIFYSQVMTKAPL	fig|127906.87.peg.3250	VCCA0532	VCA0246	ulaA/sgaT	PTS system, ascorbate-specific IIC component / PTS system, ascorbate-specific IIB component (EC 2.7.1.194)		
2	CDS	602692	603447	+	atgaatgaagtccaacgacata	MNEVQRHNGILSLLEENQAIN	fig|127906.87.peg.3251	VCCA0533	VCA0247	ulaR	Ascorbate utilization transcriptional regulator UlaR, DeoR family		
2	CDS	603574	604641	+	atgagtaaagtgaatgaaatca	MSKVNEITRESWILSTFPEWG	fig|127906.87.peg.3252	VCCA0534	VCA0248	ulaG	L-ascorbate-6-phosphate lactonase UlaG		
2	CDS	605274	604741	-	atgaataacacaaaaaacctattc	MNNTKTYSKTSIALHWIVGLAF	fig|127906.87.peg.3253	VCCA0535	VCA0249		putative cytochrome b561		
2	CDS	606847	605447	-	atgaaatcatcgtatgttgttctttg	MKSSYVVLCATFAAASFTSSV	fig|127906.87.peg.3254	VCCA0536	VCA0250	tam/amy1	Glycosidase		
2	CDS	607228	607878	+	atgagtcaaaagcattggtcaa	MSQKHWSKVQLLHEVVKNPN	fig|127906.87.peg.3255	VCCA0537	VCA0253		Xenobiotic acyltransferase XAT family		
2	CDS	608263	607997	-	gtgccgccagcgcgtatcgagc	MPPARIEQLKHYQQGFLPLHE	fig|127906.87.peg.3256	VCCA0538	No refSeq annotation		hypothetical protein	Protein of uncharacterised function (DUF3111) [Vibrio cholerae]	
2	CDS	610666	608435	-	atgaaattaaccttttcctcactgg	MKLTFSSLGLLLLVSAPSFALT	fig|127906.87.peg.3257	VCCA0539	VCA0254		hypothetical protein	Glycoside hydrolase [Vibrio cholerae]	
2	CDS	611585	610755	-	atgatctgcgcctctttcgcaatc	MICASFRNHIARLGILSALLSGA	fig|127906.87.peg.3258	VCCA0540	VCA0255		hypothetical protein	DUF2861 family protein [Vibrio cholerae]	
2	CDS	612232	611582	-	atgaagcgtattttgctgattgaa	MKRILLIEDDTLLGQGLRDYLH	fig|127906.87.peg.3259	VCCA0541	VCA0256	qseB	Two-component transcriptional response regulator, OmpR family		
2	CDS	613683	612229	-	atgacgttgctgctgactcaccg	MTLLLTHRWLSLLLCSLWAVP	fig|127906.87.peg.3260	VCCA0542	VCA0257		Two-component system sensor histidine kinase		
2	CDS	614298	613786	-	atggatcgtcatccctgcatcaa	MDRHPCIKHWSELQEKEPRY	fig|127906.87.peg.3261	VCCA0543	VCA0258		Hemolysin		
2	CDS	614469	614341	-	gtggtgattaatccactattggca	MVINPLLAAKESIKTDFAANFP	fig|127906.87.peg.3262	VCCA0544	Not annotated as CDS		hypothetical protein		
2	CDS	615002	614493	-	atgataaaaaaaaacactgcttgt	MIKKTLLVIALTFTVTTAFAHSM	fig|127906.87.peg.3263	VCCA0545	VCA0260		hypothetical protein	copper-binding protein [Vibrio cholerae]	1 ORF split into 2 ORFs in N16961, both refseq annotated in PATRIC. In KEGG N16961 its 1 ORF annotated as 1 pseudogene named VCA0260
									VCA0261				1 ORF split into 2 ORFs in N16961, both refseq annotated in PATRIC. In KEGG N16961 its 1 ORF annotated as 1 pseudogene named VCA0260
2	CDS	615522	615112	-	atgcgttttcttctgttcgcatcttc	MRFSSVRIFWIALISLFAMLSS	fig|127906.87.peg.3264	VCCA0546	VCA0262		hypothetical protein		1 ORF split into 3 ORFs in N16961 ORF is annotated as 1 pseudogene named VCA0262
									No refSeq annotation				1 ORF split into 3 ORFs in N16961 ORF is annotated as 1 pseudogene named VCA0262
									No refSeq annotation				1 ORF split into 3 ORFs in N16961 ORF is annotated as 1 pseudogene named VCA0262
2	CDS	616170	615628	-	atgaatttttcttttatacaaatcaca	MNFSLYKSQQKPEIINLFQNTF	fig|127906.87.peg.3265	VCCA0547	VCA0263		Acetyltransferase		
2	CDS	617359	616181	-	atgtatttaatttcagaattagcgg	MYLISELAVKAGLSRTTLLYYE	fig|127906.87.peg.3266	VCCA0548	VCA0264	merR1	Transcriptional regulator, MerR family		
2	CDS	617543	617923	+	atgaccaattttcaatattacttca	MTNFQYYFHQLPCFDCKKTT	fig|127906.87.peg.3267	VCCA0549	VCA0265		hypothetical protein		
2	CDS	618756	617983	-	atggccattattgatcaacagac	MAIIDQQTSFDPDRLPACVVG	fig|127906.87.peg.3268	VCCA0550	VCA0266	ripA	Transcriptional regulator, AraC family		
2	CDS	618858	619997	+	atgaagacgaagccttctctctg	MKTKPSLWLMVIMLMFPQIVE	fig|127906.87.peg.3269	VCCA0551	VCA0267		Multidrug resistance transporter, Bcr/CflA family		
2	CDS	620280	622211	+	atgaaaacggcaggggggagtg	MKTAGGVMTLGFKGRIYASV	fig|127906.87.peg.3270	VCCA0552	VCA0268		Methyl-accepting chemotaxis sensor/transducer protein		
2	CDS	622227	623900	+	atgagtcatgtgttatctgatcgct	MSHVLSDRWLALSANQLNPR	fig|127906.87.peg.3271	VCCA0553	VCA0269		Glutamate decarboxylase		
2	CDS	624065	625234	+	atgaccagcaccaagtttctcgt	MTSTKFLVRFSMACMAFVPTL	fig|127906.87.peg.3272	VCCA0554	VCA0270		D-alanyl-D-alanine carboxypeptidase (EC 3.4.16.4)		
2	CDS	625412	625759	+	atggaactgaaccaagaagac	MELNQEDRKALYDVWMTKKA	fig|127906.87.peg.3273	VCCA0555	VCA0271		hypothetical protein	helix-turn-helix domain-containing protein [Vibrio cholerae]	
2	CDS	626288	625854	-	atggagcgtgactacacttatca	MERDYTYQCLRAMDKQELTE	fig|127906.87.peg.3274	VCCA0556	No refSeq annotation		FIG01200329: hypothetical protein	exoribonuclease R [Vibrio cholerae]	
2	CDS	626343	626477	+	gtgagtgccagtcaatcttatttg	MSASQSYLSKLSITFGRDEFLE	fig|127906.87.peg.3275	VCCA0557	Not annotated as CDS		FIG01205795: hypothetical protein		
2	CDS	626505	627224	+	atgaaaaagacaacgtgggtat	MKKTTWVLAMVASMSFGVQA	fig|127906.87.peg.3276	VCCA0558	VCA0274	cah	Carbonic anhydrase, alpha class (EC 4.2.1.1)		
2	repeat	627445	628075	+	tataaaaatccgccttcttagaaggcggttttaatataagccccctaaaaggg		fig|127906.87.repeat.69				repeat region		
2	CDS	627948	627511	-	atgggcgactacagaagttcatc	MGDYRSSSHVYWRCKYHIVW	fig|127906.87.peg.3277	VCCA0559	VCA0275		Mobile element protein		
2	CDS	630992	628128	-	atgacagaattacttcacagcct	MTELLHSLSTQNEFVARHNGF	fig|127906.87.peg.3278	VCCA0560	VCA0276	gvcP	Glycine dehydrogenase [decarboxylating] (glycine cleavage system P protein) (EC 1.4.4.2)		1 ORF split into 2 ORFs in N16961. None refseq annotated according to PATRIC. Annotated as 1 ORF pseudogene named VCA0276 in KEGG
									VCA0276				1 ORF split into 2 ORFs in N16961. None refseq annotated according to PATRIC. Annotated as 1 ORF pseudogene named VCA0276 in KEGG
2	CDS	630962	631108	+	gtgtgcttaggctgtgaagtaatt	MCLGCEVILSLSFTFFPCEHAI	fig|127906.87.peg.3279	VCCA0561	No refSeq annotation		FIG01201363: hypothetical protein		
2	CDS	631516	631136	-	atggacaagacactgaagttca	MDKTLKFTESHEWVRDNGDG	fig|127906.87.peg.3280	VCCA0562	VCA0277	gcvH	Glycine cleavage system H protein		
2	CDS	632860	631553	-	atgaatgcaaatctaaacaaag	MNANLNKAYPNVSLENFFSTF	fig|127906.87.peg.3281	VCCA0563	VCA0278	glyA2	Serine hydroxymethyltransferase (EC 2.1.2.1)		
								No refSeq annotation	No refSeq annotation				
2	CDS	633248	633871	+	atgtcagaagacatttatgatga	MSEDIYDEYPSITLARESGEQ	fig|127906.87.peg.3282	VCCA0564	VCA0279	puuR	Transcriptional regulator, Xre family		
2	CDS	634115	635245	+	atgactgaacaacacgaaacc	MTEQHETLLTTPLHALHIEVGA	fig|127906.87.peg.3283	VCCA0565	VCA0280	gcvT	Aminomethyltransferase (glycine cleavage system T protein) (EC 2.1.2.10)		1 ORF split into 2 ORFs in N16961. None refseq annotated according to PATRIC. Annotated as pseudogene named VCA0280 in KEGG
									VCA0280				1 ORF split into 2 ORFs in N16961. None refseq annotated according to PATRIC. Annotated as pseudogene named VCA0280 in KEGG
2	CDS	635718	636869	+	atggcaataacggatgcatggt	MAITDAWLRATNGKPYSGKTE	fig|127906.87.peg.3284	VCCA0566	VCA0281	intS	Phage integrase		
2	repeat	636845	638041	+	agggaagatgcgattaagtctctgatatgagatcatagcaaccatcaaaac		fig|127906.87.repeat.70				repeat region		
2	CDS	636915	637895	+	atgagtcatcaactgactttcgca	MSHQLTFADGEFSNKRRQTR	fig|127906.87.peg.3285	VCCA0567	VCA0282		Mobile element protein		
2	CDS	638189	639376	+	gtgaccagagctcgtaataaaa	MTRARNKNTGEIEEASQLNRY	fig|127906.87.peg.3286	VCCA0568	VCA0283		hypothetical protein		
2	CDS	639897	640427	+	atggtaaaggcagttattgtcgg	MVKAVIVGDIGTDHDGFPPTK	fig|127906.87.peg.3287	VCCA0569	VCA0284		T6SS PAAR-repeat protein		Limited query cover for certain strains
2	CDS	640427	641098	+	atgactgacactgtatcatcaac	MTDTVSSTPVADTTPYEIFLSG	fig|127906.87.peg.3288	VCCA0570	VCA0285		Toxin TseH, peptidoglycan hydrolase		Limited query cover for certain strains
2	CDS	641100	641450	+	atgaaaaaaatcataaagtggt	MKKIIKWFAILLVSTCLAVVLLA	fig|127906.87.peg.3289	VCCA0571	VCA0286		Antitoxin TsiH to toxin TseH		Limited query cover for certain strains
2	CDS	641719	643647	+	atgcctattatactcttcctgacg	MPIITLPDGSQRHFDNPVSTLE	fig|127906.87.peg.3290	VCCA0572	VCA0287	thrS	Threonyl-tRNA synthetase (EC 6.1.1.3)		
2	CDS	643813	644202	+	ttggatctcgttgagatcagccct	MDLVEISPNAEPPVCRVMDYC	fig|127906.87.peg.3291	VCCA0573	VCA0288	infC	Translation initiation factor 3		
2	CDS	644306	644500	+	atgcctaagatgaaaaacaaca	MPKMKNNKGAAKRFKKTAGG	fig|127906.87.peg.3292	VCCA0574	VCA0289	rpmI	LSU ribosomal protein L35p		
2	CDS	644542	644895	+	atgcctcgcgtaaaacgtggtgt	MPRVKRGVQARARHKKVLKQ	fig|127906.87.peg.3293	VCCA0575	VCA0290	rplT	LSU ribosomal protein L20p		
2	CDS	645939	644977	-	atgaaatcccagttttgttaagtg	MKSQFLLSVREFMQTRYYAKI	fig|127906.87.peg.3294	VCCA0576	VCA0291		Integron integrase IntI4		
2	CDS	646419	646586	+	atgggtggtattggccgttcttgtc	MGGIGRSCRLLANSERKISLT	fig|127906.87.peg.3295	VCCA0577	No refSeq annotation		hypothetical protein	MULTISPECIES: DUF3265 domain-containing protein [Vibrio]	
2	CDS	646615	646941	+	atgaatcatcagcggttcttgttc	MNPSAVLVHVPDVAKGLEWY	fig|127906.87.peg.3296	VCCA0578	VCA0294		hypothetical protein	Glyoxalase [Vibrio parahaemolyticus]	Limited query cover for certain strains
2	CDS	647249	647374	+	ttgaaagcctgctctgctgcattg	MKACSAALSFQKVCLVFKVRF	fig|127906.87.peg.3297	VCCA0579	VCA0295		hypothetical protein		
2	repeat	647563	648560	+	ggcgttagtttctatcaagaaaaatcatcaaaagctcaggttcaacgttttgaa		fig|127906.87.repeat.71				repeat region		
2	CDS	647738	647920	+	atgttgttggattttccacacggtc	MLLDFPHGQFGFTNSCIIAVIW	fig|127906.87.peg.3298	VCCA0580	VCA0297		hypothetical protein	DUF645 family protein [Vibrio cholerae]	

2	CDS	648027	648185	+	ttgagccgcggtggtttcggtggt	MSRGGFGGLCLGLVVMRCQF	fig|127906.87.peg.3299	VCCA0581	Not present		hypothetical protein		
2	CDS	648176	648427	+	atggaactgaatacggcaatcg	MELNTAIESRIIEMAWEDRTPF	fig|127906.87.peg.3300	VCCA0582	VCA0298		Alcohol dehydrogenase, class IV		
2	CDS	648580	649011	+	atgaaaaatccgacagtagag	MKNPTVENLKVLTQEFFSLHW	fig|127906.87.peg.3301	VCCA0583	VCA0299		hypothetical protein		
2	repeat	649016	649138	+	ctaacaaacgcctcaagagggactgtcaacgcatggcgtttccagtcccaa		fig|127906.87.repeat.72				repeat region		
2	CDS	649162	649791	+	atgaacttctttacgtctccattttc	MNFFTSPFSGIPLDQQVTNPN	fig|127906.87.peg.3302	VCCA0584	VCA0300		Chloramphenicol O-acetyltransferase (EC 2.3.1.28) => CatB family		Limited query cover for certain strains
2	repeat	649781	649915	+	cacgcacataacaaacgcctcaagagggactgtcaacgcgtagcgtttcca		fig|127906.87.repeat.73				repeat region		
2	CDS	649947	650699	+	atgagcgttcaaaaagtagttgtt	MSVQKVVVITGGSRGIGAATA	fig|127906.87.peg.3303	VCCA0585	VCA0301	gdhI	Oxidoreductase, short-chain dehydrogenase/reductase family		
2	repeat	650699	650827	+	atgcataacaaacgcctcaagagggactgtcaacgcgcggcgtttccagtc		fig|127906.87.repeat.74				repeat region		
2	CDS	650773	650976	+	ttgttgtgtttgggtttggttgttatgc	MLCLGLVVMRCQPLRRALVSL	fig|127906.87.peg.3304	VCCA0586	No refSeq annotation		hypothetical protein	acetyltransferase [Vibrio cholerae]	
2	CDS	651001	651183	+	atgctgttggatgttcaacacggt	MLLDVQHGQFGFIKGGIIAVIW	fig|127906.87.peg.3305	VCCA0587	VCA0302		hypothetical protein	Select seq ref|WP_000923334.1| DUF645 family protein [Vibrio cholerae]	
2	repeat	651235	651888	+	aaactaacaaacgcctcaagagggactgtcaacgcgcggcgtttccagtc		fig|127906.87.repeat.75				repeat region		
2	CDS	651361	651765	+	atgcgacctaggaaaaaggag	MRPRKKEFRVEKIIDELVAQGY	fig|127906.87.peg.3306	VCCA0588	VCA0303		hypothetical protein		
2	CDS	652061	652243	+	gtgttgttggacgttcagcgcggt	MLLDVQRGQLSFTKYSIITVIW	fig|127906.87.peg.3307	VCCA0589	VCA0304		hypothetical protein		
2	repeat	652297	652425	+	agctaacaaacgcctcaagagggactgtcaacgcgcggcgtttccagtccc		fig|127906.87.repeat.76				repeat region		
2	CDS	652597	652481	-	ttgaagcatcgaggtttgatggtt	MKHRGLMVFSDFETTELSTRS	fig|127906.87.peg.3308	VCCA0590	VCA0305		hypothetical protein		
2	CDS	652597	652779	+	atgttgttggatgctcaactcgga	MLLDAQLGKFGFTKGCIIAESV	fig|127906.87.peg.3309	VCCA0591	VCA0306		hypothetical protein	Select seq ref|WP_142565714.1| DUF645 family protein [Vibrio cholerae]	
2	repeat	652833	652960	+	agctaacaaacgcctcaagagggactgtcaacgcgtggcgtttccagtccc		fig|127906.87.repeat.77				repeat region		
2	CDS	652974	653651	+	atggtatctcgacatataccaga	MVSRHIPERLKKKIYQEANMT	fig|127906.87.peg.3310	VCCA0592	VCA0307		hypothetical protein	Select seq ref|WP_000254617.1| HNH endonuclease [Vibrio cholerae]	
2	repeat	653646	653774	+	agctaacaaacgcctcaagagggactgtcaacgcgtggcgtttccagtccc		fig|127906.87.repeat.78				repeat region		
2	CDS	653813	655021	+	atgtacgatagtgaagtaattga	MYDSEVIEYAKEQENIISKLEY	fig|127906.87.peg.3311	VCCA0593	VCA0308		dNTP triphosphohydrolase, broad substrate specificity		
2	CDS	655183	655887	+	atgagtagaaaaaaatttatgga	MSRKKFMESVGATCSNWNW	fig|127906.87.peg.3312	VCCA0594	VCA0309		5-methylcytosine-specific restriction enzyme A (EC 3.1.21.-)		
2	repeat	655896	656020	+	taacaaacgcctcaagagggactgtcaacgcgtggcgtttccagtcccaatg		fig|127906.87.repeat.79				repeat region		
2	CDS	656045	656383	+	atgaagaaattcttagcattcata	MKKFLAFIVLSNLIAVACVYLFE	fig|127906.87.peg.3313	VCCA0595	VCA0310		hypothetical protein	putative membrane protein [Vibrio cholerae]	
2	repeat	656395	657337	+	cacataacaaacgcctcaagagggactgtcaacgcgtggcgtttccagtcc		fig|127906.87.repeat.80				repeat region		
2	CDS	656854	656537	-	atgcaaaataaacaatataagc	MQNKQYKLSQLAQEHLLKIKH	fig|127906.87.peg.3314	VCCA0596	VCA0311		ParE toxin protein		
2	CDS	657115	656873	-	atgcacacactaacagcaaatg	MHTLTANDAKRNFGELLLSAC	fig|127906.87.peg.3315	VCCA0597	VCA0312		ParD protein (antitoxin to ParE)		
2	CDS	657359	657745	+	atgaatcatctagcgatggctgtc	MNHLAMAVVIKNNLVLVQKRF	fig|127906.87.peg.3316	VCCA0598	VCA0313	ytkD	MutT/Nudix family protein		
2	repeat	657738	661261	+	acacataacaaacgcctcaagagggactgtcaacgcgtggcgtttccagtc		fig|127906.87.repeat.81				repeat region		
2	CDS	657802	657915	+	gtggttgcagttgttgtgtttgagtt	MVAVVVFEFGGMRCQPLRRA	fig|127906.87.peg.3317	VCCA0599	Not present		putative acetyltransferase		
2	CDS	657924	658145	+	atgtctgaaaatcaaaaagttaa	MSENQKVKRAEPIETCSYKSP	fig|127906.87.peg.3318	VCCA0600	VCA0314		hypothetical protein	Select seq ref|WP_084981660.1| DUF1289 domain-containing protein [Vibrio cholerae]	
									VCA0315				
2	CDS	658403	658939	+	atgttcatagaatcattaaaatc	MFIESLKIRLRSLEVEDAESFY	fig|127906.87.peg.3319	VCCA0601	VCA0316		Acetyltransferase		
2	CDS	659076	659591	+	atgagagctatcttttgattcttg	MRAIFLILCAVLLNGCLGMPES	fig|127906.87.peg.3320	VCCA0602	VCA0317		Lipocalin Blc		
2	CDS	660238	659741	-	atgatgaatacggtacttctcgat	MMNTVLLDKDKHDRNRFNCG	fig|127906.87.peg.3321	VCCA0603	VCA0318		FIG001353: Acetyltransferase		
2	CDS	660507	660235	-	atggcaacgactttgcctcgcat	MATTLPRITARVDVDTQDLLAK	fig|127906.87.peg.3322	VCCA0604	VCA0319		FIG032766: hypothetical protein	DUF1778 domain-containing protein [Vibrio cholerae]	
2	CDS	660659	660811	+	gtggttgcagttgttgtgtttgagtt	MVAVVVFEFSGMRCQPLRRA	fig|127906.87.peg.3323	VCCA0605	VCA0320		conserved hypothetical protein	Select seq ref|WP_151190703.1| tRNA (guanine(37)-N(1))-methyltransferase [Vibrio mimicus]	Limited query/suject cover for certain strains
2	CDS	661275	661721	+	atgtcggaatacaaattaacaga	MSEYKLTEEIIKLSQSQLWDV	fig|127906.87.peg.3324	VCCA0606	CA0322		hypothetical protein		
2	repeat	661726	661857	+	ggctaacaaacgcctcaagagggactgtcaacgcgtagcgtttccagtccc		fig|127906.87.repeat.82				repeat region		
2	CDS	662138	661869	-	atgtataaactcgaatactccac	MYKLEYSTQFKKDFKKITKMP	fig|127906.87.peg.3325	VCCA0607	VCA0323	yafQ	mRNA interferase YafQ		Limited query cover for certain strains
2	CDS	662409	662131	-	atgagaacagaaatgctgagca	MRTEMLSTRIDHDTKIAFTNV	fig|127906.87.peg.3326	VCCA0608	VCA0324		Antitoxin DinJ (binds YafQ toxin)		
2	CDS	662695	662973	+	atgtatctggcgctgaaaaatcg	MYLALKNRSDCLIVLLSLREVS	fig|127906.87.peg.3327	VCCA0609	VCA0325		hypothetical protein	Select seq ref|WP_080033414.1| DUF3709 domain-containing protein [Vibrio cholerae]	
2	repeat	663100	663228	+	gcataacaaacgcctcaagagggactgtcaacgcgtagcgtttccagtccc		fig|127906.87.repeat.83				repeat region		
2	CDS	663288	663431	+	ttgtcggcaattcaatattgtttttc	MSAIQYCFSLPNEAAMYLVLK	fig|127906.87.peg.3328	VCCA0610	VCA0327		hypothetical protein	DUF3709 domain-containing protein [Vibrio cholerae]	
2	repeat	663564	665157	+	cttaagcataacaaacgcctcaagagggactgtcaacgcgtggcgtttcca		fig|127906.87.repeat.84				repeat region		
2	CDS	663722	664108	+	atgaaaattagtcaccttgatcat	MKISHLDHLVLTVADIPTTTNF	fig|127906.87.peg.3329	VCCA0611	VCA0328		biphenyl-2,3-diol 1,2-dioxygenase III-related protein		
2	CDS	664175	664312	+	ttgtggtgtttgagtttagtggtcat	MWCLSLVVMRCQPLRRALGV	fig|127906.87.peg.3330	VCCA0612	Not present		hypothetical protein		
2	CDS	664303	664554	+	atggaactgaatacggcaatcg	MELNTAIESRIIEMAWEDRTPF	fig|127906.87.peg.3331	VCCA0613	VCA0329		Alcohol dehydrogenase, class IV		
2	CDS	664768	665010	+	atgagcacaaggttttgtgaaaa	MSTRFCEKCGKNTEHKEVMK	fig|127906.87.peg.3332	VCCA0614	Not present		FIG032766: hypothetical protein	Select seq gb|OAB88233.1| pullulanase [Vibrio cholerae]	
2	CDS	665262	665540	+	atgtatttggcgctgaaaaatcg	MYLALKNRSDCLIVLLSLREVS	fig|127906.87.peg.3333	VCCA0615	No refSeq annotation		hypothetical protein	DUF3709 domain-containing protein [Vibrio cholerae]	
2	repeat	665663	665806	+	ttaagcataacaaacgcctcaagagggactgtcaacgcgtggcgtttccag		fig|127906.87.repeat.85				repeat region		
2	repeat	666205	666334	+	agctaacaaacgcctcaagagggactgtcaacgcgtggcgtttccagtccc		fig|127906.87.repeat.86				repeat region		
2	CDS	666362	666829	+	atgtcagaacattcagcaatcgt	MSEHSAIVTWKRKDSEAFTDN	fig|127906.87.peg.3334	VCCA0616	VCA0330		Predicted redox protein	Select seq ref|WP_001908912.1| OsmC family protein [Vibrio cholerae]	Limited query/suject cover for certain strains
2	repeat	666825	666953	+	cctaacaaacgcctcaagagggactgtcaacgcgaggcgtttccagtccca		fig|127906.87.repeat.87				repeat region		
2	CDS	666957	667355	+	atgttggtactaatacaattgatat	MLVLIQLILSKVKEFFKEWMPL	fig|127906.87.peg.3335	VCCA0617	VCA0331		hypothetical protein		Limited query/suject cover for certain strains
2	repeat	667629	667753	+	taacaagcgcttcaagagggacagccaacgcgcggcatttttactatgcgtt		fig|127906.87.repeat.88				repeat region		
					atgattaaatttgaatttgatgaaaacaaaagcagaagtaatctcgaaaagc		Only in N16961		VCA0332		Hypothetical protein		
2	CDS	668058	668303	+	atgaaagcgcatgaatttgatgc	MKAHEFDAKFESDDDDIVMDL	fig|127906.87.peg.3336	VCCA0618	VCA0333		Helix-turn-helix protein, CopG family		
2	CDS	668399	669193	+	gtgcgttgttcgccccttaacgcg	MRCSPLNAALYLLEILVKIKNV	fig|127906.87.peg.3337	VCCA0619	VCA0334		hypothetical protein		
2	repeat	669310	669849	+	ggcgttagtttctatcaagaaaaatcatcaaaagctcaggttcaacgttttgaa		fig|127906.87.repeat.89				repeat region		
2	CDS	669485	669667	+	atgttgttggattttccacacggtc	MLLDFPHGQFGFTNSCIIAVIW	fig|127906.87.peg.3338	VCCA0620	VCA0336		hypothetical protein	Select seq ref|WP_000923182.1| DUF645 family protein [Vibrio cholerae]	
2	CDS	669883	670887	+	gtgttatatgccaaagctttaagt	MLYAKALSIGDEIGFFSPSSPA	fig|127906.87.peg.3339	VCCA0621	VCA0337		Microcin C7 immunity MccF-like protein		
2	repeat	670879	671009	+	gcaaaataacaaacgcctcaagagggactgtcaacgcgtggcgtttccag		fig|127906.87.repeat.90				repeat region		
2	CDS	671040	671399	+	atgaatcagcatggaaaactca	MNQHGKLNYVEFAAKDLESTI	fig|127906.87.peg.3340	VCCA0622	VCA0338		Glyoxalase family protein		Limited query cover in certain strains
2	repeat	671393	671522	+	cgcataacaaacgcctcaagagggactgtcaacgcgtggcgtttccagtcc		fig|127906.87.repeat.91				repeat region		
2	CDS	671687	671905	+	gtgagtttgctctctcaagtcgct	MSFALSSRFEQFGAFTYGLKV	fig|127906.87.peg.3341	VCCA0623	VCA0339		hypothetical protein	Select seq ref|WP_001919226.1| DUF3709 domain-containing protein [Vibrio cholerae]	Limited query cover in certain strains
2	repeat	672027	672158	+	cttaagcataacaaacgcctcaagagggactgtcaacgcgtggcgtttccag		fig|127906.87.repeat.92				repeat region		
2	CDS	672173	672679	+	atggaagaaacagattttctaca	MEETDFLQELADELSALPDPS	fig|127906.87.peg.3342	VCCA0624	VCA0340		hypothetical protein		
2	repeat	672684	673801	+	ctaacaaacgcctcaagagggactgtcaacgcgtggcgtttccagtcccaa		fig|127906.87.repeat.93				repeat region		
2	CDS	672836	673222	+	atgaaaattagtcaccttgatcat	MKISHLDHLVLTVADIPTTTNF	fig|127906.87.peg.3343	VCCA0625	VCA0341		biphenyl-2,3-diol 1,2-dioxygenase III-related protein		
								Not present	VCA0342				
2	CDS	673289	673426	+	ttgtggtgtttgagtttagtggtcat	MWCLSLVVMRCQPLRRALGV	fig|127906.87.peg.3344	VCCA0626	Not present		hypothetical protein		
2	CDS	673417	673668	+	atggaactgaatacggcaatcg	MELNTAIESRIIEMAWEDRTPF	fig|127906.87.peg.3345	VCCA0627	VCA0343		Alcohol dehydrogenase, class IV		
2	CDS	673937	674362	+	gtggaaaaaagtaacgagaaa	MEKSNEKVQLLVNAIRFRKPY	fig|127906.87.peg.3346	VCCA0628	VCA0344		hypothetical protein		
2	repeat	674407	674542	+	cgcacctaacaaacgcctcaagagggactgtcaacgcgcggcgtttccag		fig|127906.87.repeat.94				repeat region		
2	CDS	674568	675191	+	atggtaaattttactgctgggaag	MVNFTAGKWISEPKVSEVTSE	fig|127906.87.peg.3347	VCCA0629	VCA0345		hypothetical protein	DUF1349 domain-containing protein [Vibrio cholerae]	
2	repeat	675196	675329	+	aaattcacataacaaacgcctcaagagggactgtcaacgcgtggcgtttcca		fig|127906.87.repeat.95				repeat region		
2	CDS	675404	675883	+	atgtcatttcagaatctaaatgctg	MSFQNLNAGDIVVSNFGVYQI	fig|127906.87.peg.3348	VCCA0630	VCA0346		H-REV 107-related protein		Limited query cover for certain strains
2	repeat	675885	678038	+	tataacaaacgcttcaagaaggacagccaacgcgcggcatttttactatgcg		fig|127906.87.repeat.96				repeat region		
2	CDS	676071	676484	+	atgtctaaagtcgttcaacagca	MSKVVQQQIGNIALVVENYDD	fig|127906.87.peg.3349	VCCA0631	VCA0347		Glyoxalase family protein		
2	CDS	676913	676635	-	atgattttctgggaagaagcatct	MIFWEEASLNDREKIFEFLYDF	fig|127906.87.peg.3350	VCCA0632	VCA0348		Death on curing protein, Doc toxin		
2	CDS	677194	676910	-	atggatactagaattcaatttcgt	MDTRIQFRVDEETKRLAQQMA	fig|127906.87.peg.3351	VCCA0633	VCA0349		Antitoxin DinJ (binds YafQ toxin)		
2	CDS	677389	677904	+	atgagagctatctttttgattctttg	MRAIFLILCSVLLNGCLGMPES	fig|127906.87.peg.3352	VCCA0634	VCA0350		Lipocalin Blc		
2	CDS	677981	678565	+	gtggtgtttgagtttagtggtatgc	MVFEFSGMRCQPLRRALSALT	fig|127906.87.peg.3353	VCCA0635	VCA0351		hypothetical protein		
2	repeat	678667	679323	+	taacaaacgcctcaagagggactgtcaacgcgcggcgtttccagtcccaat		fig|127906.87.repeat.97				repeat region		
2	CDS	678759	678896	+	atgcgttgccagccccttaggcg	MRCQPLRRALVSIKKNHQKLR	fig|127906.87.peg.3354	VCCA0636	Not present		conserved hypothetical protein	Select seq gb|ORQ05690.1| general secretion pathway protein GspI [Vibrio cholerae]	
2	CDS	678957	679139	+	atgttgttggattttccacacggtc	MLLDFPHGQFGFTNSCIIAVIW	fig|127906.87.peg.3355	VCCA0637	VCA0353		hypothetical protein	DUF645 family protein [Vibrio cholerae]	
2	CDS	679246	679812	+	ttgagccgcggtggtttcggtggt	MSRGGFGGLCLGLVVMRCQF	fig|127906.87.peg.3356	VCCA0638	VCA0354		hypothetical protein		
2	CDS	679961	680587	+	atggatcttaattcactactactgt	MDLNSLLLFIVACLAINMIPGPI	fig|127906.87.peg.3357	VCCA0639	VCA0355		Transporter, LysE family		
2	CDS	680728	681408	+	gtggaaaaatgttacatgtgcga	MEKCYMCDAEGNTKEHVPPK	fig|127906.87.peg.3358	VCCA0640	VCA0356		hypothetical protein		
2	CDS	681588	681749	+	atgtttcgttgtgggcaatgccgtc	MFRCGQCRHFERAKNNLKDL	fig|127906.87.peg.3359	VCCA0641	No refSeq annotation		hypothetical protein		
2	repeat	681806	681942	+	taacaaacgcctcaagagggactgtcaacgcgtggcgtttccagtcccattg		fig|127906.87.repeat.98				repeat region		
2	CDS	682160	682285	+	gtgttctggctttctttgtcgcggat	MFWLSLSRILNRGQLSLERFE	fig|127906.87.peg.3360	VCCA0642	VCA0357		hypothetical protein	Select seq ref|WP_001883058.1| DUF645 family protein [Vibrio cholerae]	
2	repeat	682339	682466	+	agctaacaaacgcctcaagagggactgtcaacgcgtggcgtttccagtccc		fig|127906.87.repeat.99				repeat region		
2	CDS	682499	682894	+	atgaaatggtttttagctttttggctt	MKWFLAFWLVLASFFSVGLHA	fig|127906.87.peg.3361	VCCA0643	VCA0358		hypothetical protein	Select seq ref|WP_000870857.1| DUF3465 domain-containing protein [Vibrio cholerae]	
2	repeat	682545	683028	+	cggtcggcttgcatgccaatgactccgttttacaacaagcttataaatcgcaac		fig|127906.87.repeat.100				repeat region		
2	CDS	683321	683022	-	atgaaaccatttaatcttaccgtc	MKPFNLTVAAKADLRDIALFTC	fig|127906.87.peg.3362	VCCA0644	VCA0359		ParE toxin protein		
2	CDS	683574	683308	-	atggagatgagctcatggctaaa	MEMSSWLKIQVSLLVNTSMAL	fig|127906.87.peg.3363	VCCA0645	No refSeq annotation		hypothetical protein	antitoxin protein parD-1 [Vibrio cholerae HCUF01]	Limited query cover for certain strains
2	repeat	683627	683757	+	cgcataacaaacgcctcaagcgggactgtcaacgcgtggcgtttccagtcc		fig|127906.87.repeat.101				repeat region		
2	CDS	683789	684367	+	atgagtgtagagttaacatctgct	MSVELTSAVFASDIVEFKENNI	fig|127906.87.peg.3364	VCCA0646	VCA0361		hypothetical protein		
2	CDS	684541	684702	+	atgtttcgttgtgggcaatgccgtc	MFRCGQCRHFERAKNNLKDL	fig|127906.87.peg.3365	VCCA0647	VCA0362		hypothetical protein		Limited query cover for certain strains
2	repeat	684759	684884	+	taacaaacgcctcaagagggactgtcaacgcgcggcgtttccagtcccattg		fig|127906.87.repeat.102				repeat region		
2	CDS	684905	685588	+	atgaaatatcgaagtaaatggg	MKYRSKWELCFNAYKKECTR	fig|127906.87.peg.3366	VCCA0648	VCA0363		hypothetical protein		
2	CDS	685802	686068	+	atgttgccatcaaaagaagaac	MLPSKEELLALLSKECELYASI	fig|127906.87.peg.3367	VCCA0649	VCA0364		hypothetical protein		
2	repeat	686084	686207	+	ctaacaaacgcctcaagagggactgtcaacgcgcggcgtttccagtcccaa		fig|127906.87.repeat.103				repeat region		
2	CDS	686223	686822	+	atggaaaatttaacaaatgatgt	MENLTNDVITILQYLLPGFVSA	fig|127906.87.peg.3368	VCCA0650	Not present		hypothetical protein		
2	CDS	687103	688038	+	ttgagtttaggtttttggggttggct	MSLGFWGWLKSNLSHLFSLL	fig|127906.87.peg.3369	VCCA0651	VCA0366		hypothetical protein	Select seq ref|WP_000051985.1| restriction endonuclease [Vibrio cholerae]	
2	CDS	688004	688144	+	atgggcttggtgggcaattggag	MGLVGNWRILYNKQFKRDSA	fig|127906.87.peg.3370	VCCA0652	No refSeq annotation		hypothetical protein	hybrid peroxiredoxin hyPrx5 [Vibrio cholerae CP1048(21)]	
2	CDS	688256	689278	+	atgctgtcatactatgagcgaaa	MLSYYERNIDYLELELKSIPTN	fig|127906.87.peg.3371	VCCA0653	VCA0367		hypothetical protein		
2	repeat	689786	691831	+	tgtagcgcgccaagataaaacgcttaaaatttcaagataaataaaattcaga		fig|127906.87.repeat.104				repeat region		
									VCA0368				
2	CDS	690452	689886	-	atgattggagatatggttcttggc	MIGDMVLGGKKGAAGAGSLS	fig|127906.87.peg.3372	VCCA0654	VCA0369		hypothetical protein	phage tail tape measure family protein [Vibrio cholerae]	
									VCA0370				
2	CDS	691450	690578	-	ttgataatcaagaaactcaagca	MIIKKLKQSHSVKILCEVFNVHI	fig|127906.87.peg.3373	VCCA0655	VCA0371		Transposase InsO for insertion sequence element IS911		
2	CDS	691755	691447	-	atgacaaaacgtacaagacga	MTKRTRRLFSAEFKLEAAQLV	fig|127906.87.peg.3374	VCCA0656	VCA0372		Transposase InsN for insertion sequence element IS911		
2	repeat	692007	692135	+	atataacaaacgcctcaagagggactgtcaacgcgcggcgtttccagtccc		fig|127906.87.repeat.105				repeat region		
2	CDS	692173	693180	+	atgtctagttttgattttcctaagtat	MSSFDFPKYQANQEVGNTGE	fig|127906.87.peg.3375	VCCA0657	VCA0374		hypothetical protein	DUF4365 domain-containing protein [Vibrio cholerae]	
2	CDS	693405	693683	+	atgtatctggcgctaaaaaatcg	MYLALKNRSDCLIVLLSLCEVS	fig|127906.87.peg.3376	VCCA0658	VCA0375		hypothetical protein	Select seq ref|WP_001883042.1| DUF3709 domain-containing protein [Vibrio cholerae]	Limited query cover for certain strains
2	repeat	693806	693938	+	cttaagcataacaaacgcctcaagagggactgtcaacgcgtggcgtttccag		fig|127906.87.repeat.106				repeat region		
2	CDS	693936	694331	+	atggcgcaaggcatgacacac	MAQGMTHTFIGTIMNTMEFVR	fig|127906.87.peg.3377	VCCA0659	VCA0376		hypothetical protein		Limited query cover for certain strains
2	repeat	694335	694473	+	cataacaaacgcctcaagagggactgtcaacgcgtagcgtttccagtccca		fig|127906.87.repeat.107				repeat region		
2	CDS	694473	694348	-	ttgcttcaagaagctaacgcccg	MLQEANARLRGWQRITTKLKH	fig|127906.87.peg.3378	VCCA0660	Not present		hypothetical protein	Plasmid stabilization element ParE%2C putative [Vibrio cholerae]	
2	CDS	694696	694815	+	gtgctttctttgtcgcggactcttaa	MLSLSRTLNRGQLSFERFEFW	fig|127906.87.peg.3379	VCCA0661	No refSeq annotation		hypothetical protein	Select seq ref|WP_080370062.1| DUF645 family protein [Vibrio cholerae]	
2	repeat	694871	694994	+	ctaacaaacgcctcaaagggactgtcaacgcgtggcgtttccagtcccattg		fig|127906.87.repeat.108				repeat region		
2	CDS	694992	695387	+	atggcgcaaggcatgacacac	MAQGMTHTFIGTIMNTMEFVR	fig|127906.87.peg.3380	VCCA0662	VCA0379		hypothetical protein		Limited query cover for certain strains
2	repeat	695391	695517	+	cataacaaacgcctcaagagggactgtcaacgcgtagcgtttccagtccca		fig|127906.87.repeat.109				repeat region		
2	CDS	695480	696067	+	gtggtaatgcgttgccagcccctt	MVMRCQPLRRALWRKVKMKA	fig|127906.87.peg.3381	VCCA0663	VCA0380		hypothetical protein	Select seq gb|APF50759.1| nitrate reductase [Vibrio cholerae]	
2	repeat	696063	696189	+	cataacaaacgcctcaagagggaccgtcaacgcgtggcgtttccagtccca		fig|127906.87.repeat.110				repeat region		
2	CDS	696379	696245	-	atggaacatccaacaaccttga	MEHPTTLKHRGLWVFSGFEP	fig|127906.87.peg.3382	VCCA0664	No refSeq annotation		hypothetical protein		
2	CDS	696418	696543	+	gtgatcttgctttctttgtcgcggac	MILLSLSRTLNRGQLSLDRFKF	fig|127906.87.peg.3383	VCCA0665	VCA0381		hypothetical protein	Select seq ref|WP_113772954.1| DUF645 family protein [Vibrio cholerae]	
2	repeat	696601	697287	+	taacaaacgcctcaagagggactgtcaacgcgtggcgtttccagtcccaatg		fig|127906.87.repeat.111				repeat region		
2	CDS	696739	697164	+	atggaaatcaaagagtttagtgc	MEIKEFSACNLESLRKLYLDSF	fig|127906.87.peg.3384	VCCA0666	VCA0382		Acetyltransferase		
2	CDS	697373	697660	+	atgtttgagaaatctacgggtttct	MFEKSTGFFGLRSWPDYAFFY	fig|127906.87.peg.3385	VCCA0667	Not present		hypothetical protein		
2	repeat	697654	697782	+	aacataacaaacgcctcaagagggactgtcaacgcgtggcgtttccagtcc		fig|127906.87.repeat.112				repeat region		
2	repeat	698608	699541	+	ctaacaaacgcctcaaagggactgccaacgcgtggcgtttccagtcccattg		fig|127906.87.repeat.113				repeat region		
2	CDS	699062	698745	-	atgcaaaataaacaatataagc	MQNKQYKLSQLAQEHLLKIKH	fig|127906.87.peg.3386	VCCA0668	VCA0385		ParE toxin protein		
2	CDS	699323	699081	-	atgcacacactaacagcaaatg	MHTLTANDAKRNFGELLLSAC	fig|127906.87.peg.3387	VCCA0669	VCA0386		ParD protein (antitoxin to ParE)		
2	CDS	699561	699427	-	atgtcctctttcttcgagctttataa	MSSFFELYNARLRGWQRITTK	fig|127906.87.peg.3388	VCCA0670			hypothetical protein	Plasmid stabilization element ParE%2C putative [Vibrio cholerae]	
2	CDS	699565	700074	+	gtggaaattcgaacaggtgaat	MEIRTGEFEDIAGITDIFNFYIE	fig|127906.87.peg.3389	VCCA0671	VCA0387	pat	Acetyltransferase, GNAT family		
2	repeat	700069	700196	+	ttataacaaacgcctcaagagggactgtcaacgcgcggcgtttccagtccca		fig|127906.87.repeat.114				repeat region		
2	CDS	700164	700784	+	atgcgttgccagccccttaggcg	MRCQPLRRALGILEGALSDIIL	fig|127906.87.peg.3390	VCCA0672	VCA0388		hypothetical protein		Limited query/subject for certain strains
2	repeat	700801	700928	+	acctaacaaacgcctcaagagggactgtcaacgcgtggcgtttccagtccc		fig|127906.87.repeat.115				repeat region		
2	CDS	700891	701082	+	gtggtagtgcgttgccagcccctt	MVVRCQPLRRALCLVHKNQQ	fig|127906.87.peg.3391	VCCA0673	?????		FIG032766: hypothetical protein	Select seq ref|WP_154401004.1| DUF3709 domain-containing protein [Vibrio cholerae]	Limited query/subject for certain strains
2	CDS	701094	701312	+	gtgagtttggctctctcaagtcgc	MSLALSSRFDQFGAFTYKLKV	fig|127906.87.peg.3392	VCCA0674	VCA0389		hypothetical protein	DUF3709 domain-containing protein [Vibrio cholerae]	
2	repeat	701439	701572	+	gcataacaaacgcctcaagagggactgtcaacgcgtggcgtttccagtccc		fig|127906.87.repeat.116				repeat region		
2	repeat	702075	702201	+	gcataacaaacgcctcaagagggactgtcaacgcgtagcgtttccagtccc		fig|127906.87.repeat.117				repeat region		
2	repeat	702595	702722	+	gctaacaaacgcctcaagcgggactgtcaacgcgtggcgtttccagtccca		fig|127906.87.repeat.118				repeat region		
2	CDS	702666	702779	+	ttgttgaggttgagtttagtgttaat	MLRLSLVLMRCQPLRRALCTF	fig|127906.87.peg.3393	VCCA0675	Not present		conserved hypothetical protein	Select seq gb|ORP22378.1| acetyltransferase [Vibrio cholerae]	
2	CDS	702808	703107	+	gtgcaagttgcaatggcattaga	MQVAMALEFKDKWLEQFYED	fig|127906.87.peg.3394	VCCA0676	VCA0391		Toxin HigB		
2	CDS	703118	703435	+	atgcgtaagacaaaacgtcgtc	MRKTKRRPVSVGEMLKVEFLE	fig|127906.87.peg.3395	VCCA0677	VCA0392		Antitoxin HigA		
2	repeat	703430	703552	+	acataacaaacgcttcaagagggacagccaacgcgtggcatttttaccatg		fig|127906.87.repeat.119				repeat region		
2	CDS	703807	703923	+	gtggccaacttaaccttgatcgtt	MANLTLIVLSFGSQRHSFWRW	fig|127906.87.peg.3396	VCCA0678	VCA0394		hypothetical protein	Tfp pilus assembly protein [Vibrio cholerae]	
2	repeat	703951	704079	+	agctaacaaacgcctcaagagggactgtcaacgcgtggcgtttccagtccc		fig|127906.87.repeat.120				repeat region		
2	CDS	704047	704862	+	atgcgttgccagccccttaggcg	MRCQPLRRALNCSQGWWIIK	fig|127906.87.peg.3397	VCCA0679	VCA0395		Phenazine biosynthesis protein PhzF like		
2	repeat	704857	704985	+	atttaacaaacgcctcaagagggactgtcaacgcgtggcgtttccagtccca		fig|127906.87.repeat.121				repeat region		
2	CDS	704990	704871	-	gtgacagctaacgcccgcctaa	MTANARLRGWQRITTKLRHN	fig|127906.87.peg.3398	VCCA0680	Not present		hypothetical protein	Plasmid stabilization element ParE%2C putative [Vibrio cholerae]	
2	CDS	705067	705897	+	atgagtctaccaataaccgatag	MSLPITDSCIVAVAKLVDDAQS	fig|127906.87.peg.3399	VCCA0681	VCA0396		hypothetical protein	Select seq ref|WP_000056616.1| abortive infection family protein [Vibrio cholerae]	
2	repeat	705891	706556	+	cagctaacaaacgcctcaagagggactgtcaacgcgcggcgtttccagtcc		fig|127906.87.repeat.122				repeat region		
2	CDS	705954	706436	+	gtggtttcggctgttgtgtttgagtt	MVSAVVFEFCVMRCQPLRRA	fig|127906.87.peg.3400	VCCA0682	VCA0397		hypothetical protein		
2	CDS	706606	706767	+	atgtttcgttgtgggcaatgccgtc	MFRCGQCRHFEKAKNNLKDL	fig|127906.87.peg.3401	VCCA0683	VCA0398		hypothetical protein		
2	repeat	706824	706949	+	taacaaacgcctcaagagggactgtcaacgcgtggcgtttccagtcccattg		fig|127906.87.repeat.123				repeat region		
2	CDS	706916	707758	+	atgcgttgtcagccccttaggcg	MRCQPLRRALGISREIVLANKI	fig|127906.87.peg.3402	VCCA0684	VCA0399		hypothetical protein		
2	repeat	707765	709076	+	ctaacaaacgcctcaagcgggactgtcaacgcgtggcgtttccagtcccaa		fig|127906.87.repeat.124				repeat region		
2	CDS	707888	708343	+	atgaggaatatggatatcaaatt	MRNMDIKLRPIESEEFDSLFS	fig|127906.87.peg.3403	VCCA0685	VCA0400		Histone acetyltransferase HPA2		

2	CDS	708527	708940	+	atgtctaaagtcgttcaacagca	MSKVVQQQIGNIALVVENYDD	fig|127906.87.peg.3404	VCCA0686	VCA0401		Glyoxalase family protein		
2	CDS	709007	709525	+	gtggtttcggttgttgtgtttgagttt	MVSVVVFEFSGMRCQPLRRA	fig|127906.87.peg.3405	VCCA0687	VCA0402		Acetyltransferase, GNAT family		Limited subject cover for certain strains
2	repeat	709515	709653	+	cacacacataacaaacgcctcaagagggactgtcaacgcgcggcgtttcc		fig|127906.87.repeat.125				repeat region		
2	repeat	710000	710666	+	acacataacaaacgcctcaagagggactgtcaacgcgcggcgtttccagt		fig|127906.87.repeat.126				repeat region		
								Not present	VCA0403				
2	CDS	710311	710481	+	atgttcaattcggtcaacttggttt	MFNSVNLVSQKATSSLKSCFF	fig|127906.87.peg.3406	VCCA0688	No refSeq annotation		hypothetical protein	DUF645 family protein [Vibrio cholerae]	
2	CDS	710709	711329	+	atgtttggtggtgtgactatttggct	MFGGVTIWLALSILIGYWASKL	fig|127906.87.peg.3407	VCCA0689	VCA0405		hypothetical protein	YrdB family protein [Vibrio cholerae]	
2	repeat	711332	712123	+	taaacataacaaacgcctcaagagggactgtcaacgcgtggcgtttccagt		fig|127906.87.repeat.127				repeat region		
2	CDS	711477	711992	+	atgagagctatctttttgattctttg	MRAIFLILCSVLLNGCLGMPES	fig|127906.87.peg.3408	VCCA0690	VCA0406		Lipocalin Blc		
2	CDS	712295	712477	+	atgttgtcggatattcaacacgat	MLSDIQHDQFGFIKGCIISVIWI	fig|127906.87.peg.3409	VCCA0691	VCA0407		hypothetical protein	Select seq ref|WP_000947513.1| DUF645 family protein [Vibrio cholerae]	
2	repeat	712530	713137	+	aaactaacaaacgcctcaagagggactgtcaacgcgcggcgtttccagtc		fig|127906.87.repeat.128				repeat region		
2	CDS	712656	713003	+	atgcaacattggggattaaaggt	MQHWGLKVSDLFSTIIIVAIGLA	fig|127906.87.peg.3410	VCCA0692	VCA0408		hypothetical protein	putative membrane protein [Vibrio cholerae O1 str. 116059]	Limited query cover for certain strains
2	CDS	713165	713623	+	atgaaagatgaaataagtaatc	MKDEISNPDLGAIYTTKILGVLL	fig|127906.87.peg.3411	VCCA0693	VCA0409		hypothetical protein		Limited query cover for certain strains
2	repeat	713631	713758	+	cctaacaaacgcctcaagagggactgtcaacgcgcggcgtttccagtccca		fig|127906.87.repeat.129				repeat region		
2	CDS	713774	714136	+	atggaagtaactttgtatgatgga	MEVTLYDGQGKPVAYVAADS	fig|127906.87.peg.3412	VCCA0694	VCA0410		hypothetical protein		
2	CDS	714436	714320	-	ttgaagcatcgagacttgctagtt	MKHRDLLVFIDFEPTELSTRST	fig|127906.87.peg.3413	VCCA0695	VCA0411		hypothetical protein		
2	CDS	714493	714618	+	gtgttcttgctttctttgtcgcggac	MFLLSLSRTLNRGQLSLDRFK	fig|127906.87.peg.3414	VCCA0696	VCA0412		hypothetical protein	Select seq ref|WP_001889214.1| DUF645 family protein [Vibrio cholerae]	
2	repeat	714672	714798	+	agctaacaaacgcctcaagagggactgtcaacgcgtggcgtttccagtccc		fig|127906.87.repeat.130				repeat region		
2	CDS	714814	715467	+	atgagttcagacgttaatgaaat	MSSDVNEIIDLFITREAKFRSW	fig|127906.87.peg.3415	VCCA0697	VCA0413		hypothetical protein		
2	repeat	715463	715591	+	cctaacaaacgcctcaagagggactgtcaacgcgcggcgtttccagtccca		fig|127906.87.repeat.131				repeat region		
2	CDS	715622	716017	+	atgaaatggtttttagcgtgttggc	MKWFLACWLVLASCFSVSLHA	fig|127906.87.peg.3416	VCCA0698	VCA0414		hypothetical protein	DUF3465 domain-containing protein [Vibrio cholerae]	
2	repeat	715668	716150	+	cggtcagcttgcatgccaatgacactgttttacaacaagcttatcaatcgcag		fig|127906.87.repeat.132				repeat region		
2	CDS	716180	716539	+	atgaaccagcatgaaaaaatca	MNQHEKINYVEFAAKDLESTK	fig|127906.87.peg.3417	VCCA0699	VCA0415		Glyoxalase family protein		
2	repeat	716534	716662	+	gcataacaaacgcctcaagagggactgtcaacgcgtggcgtttccagtccc		fig|127906.87.repeat.133				repeat region		
2	CDS	716767	717045	+	atgtatctggtgctgaaaattcag	MYLVLKIQCYCFIEMLSLRGVN	fig|127906.87.peg.3418	VCCA0700	VCA0416		hypothetical protein	DUF3709 domain-containing protein [Vibrio cholerae]	
2	repeat	717172	717986	+	gcataacaaacgcctcaagagggactgtcaacgcgtagcgtttccagtccc		fig|127906.87.repeat.134				repeat region		
2	CDS	717326	717862	+	atgttcatagaatcattaaaaatc	MFIESLKIRLRSLEVEDAESFY	fig|127906.87.peg.3419	VCCA0701	VCA0417		Acetyltransferase		
2	CDS	718001	718318	+	ttggacttatcaaaattagaacat	MDLSKLEHHFELLEELGFITNC	fig|127906.87.peg.3420	VCCA0702	Not present		hypothetical protein		
2	repeat	718322	718453	+	cacataacaaacgcctcaagagggactgtcaacgcgtggcgtttccagtcc		fig|127906.87.repeat.135				repeat region		
2	CDS	718623	718805	+	atgttgtcggatattcaacacggc	MLSDIQHGKFGFTKGSIIAVILL	fig|127906.87.peg.3421	VCCA0703	VCA0418		hypothetical protein	MULTISPECIES: DUF645 family protein [Vibrio]	
2	repeat	718857	718986	+	aaactaacaaacgcctcaagagggactgtcaacgcgtggcgtttccagtcc		fig|127906.87.repeat.136				repeat region		
2	CDS	718954	719709	+	atgcgttgccagccccttaggcg	MRCQPLRRALALKRKKVIFRT	fig|127906.87.peg.3422	VCCA0704	VCA0419		hypothetical protein		
2	repeat	719716	719843	+	ggctaacaaacgcctcaagagggactgtcaacgcgtggcgtttccagtccc		fig|127906.87.repeat.137				repeat region		
2	CDS	719778	720173	+	gtggttacggttgttgtgattgagt	MVTVVVIEFSVMRCQPLRRAL	fig|127906.87.peg.3423	VCCA0705	VCA0420		hypothetical protein	DUF4144 domain-containing protein [Vibrio cholerae]	
2	repeat	720180	720310	+	taaacataacaaacgcctcaagagggactgtcaacgcgtggcgtttccagt		fig|127906.87.repeat.138				repeat region		
2	CDS	720264	720383	+	ttgggtttagtggtaatgcgttgtc	MGLVVMRCQPLRRALAHTSH	fig|127906.87.peg.3424	VCCA0706	Not present		conserved hypothetical protein	C4-dicarboxylate ABC transporter [Vibrio cholerae]	
2	CDS	720419	720844	+	atgaaaaaattgttaatggtactg	MKKLLMVLTVMIALVGCDDAT	fig|127906.87.peg.3425	VCCA0707	VCA0420		hypothetical protein		
2	CDS	720852	720980	+	gtgctaacaaacgcctcaagag	MLTNASRGTVNARRFQSHWA	fig|127906.87.peg.3426	VCCA0708	Not present		hypothetical protein	Select seq ref|WP_141239545.1| acetyltransferase [Vibrio cholerae]	
2	repeat	720854	720979	+	gctaacaaacgcctcaagagggactgtcaacgcgcggcgtttccagtccca		fig|127906.87.repeat.139				repeat region		
2	CDS	721058	721339	+	atgagccgtattcaccttgatcaa	MSRIHLDQDIQPLSEFRAGVA	fig|127906.87.peg.3427	VCCA0709	VCA0422		Prevent host death protein, Phd antitoxin		
2	CDS	721336	721695	+	atgaaagtagtttggtcacctcta	MKVVWSPLALQKLGDAAEFIA	fig|127906.87.peg.3428	VCCA0710	VCA0423		Death on curing protein, Doc toxin		
2	repeat	721612	721745	+	cataacaaacgcctcaagagggactgtcaacgcgcggcgtttccagtccca		fig|127906.87.repeat.140				repeat region		
2	CDS	721677	722123	+	ttgcagttgttgtgtttgagtttggtg	MQLLCLSLVIVRCQPLRRALCI	fig|127906.87.peg.3429	VCCA0711	VCA0424		hypothetical protein	DUF3709 domain-containing protein [Vibrio cholerae]	
2	repeat	722244	722378	+	cttaagcataacaaacgcctcaagagggactgtcaacgcgtagcgtttccag		fig|127906.87.repeat.141				repeat region		
2	CDS	722409	722804	+	atgaaatggtttttagcttttggctt	MKWFLAFWLVLTSCFSVSLYA	fig|127906.87.peg.3430	VCCA0712	VCA0425		hypothetical protein	DUF3465 domain-containing protein [Vibrio cholerae]	
2	repeat	722461	723447	+	gcttgtatgccaatgacgccgttttacaacaagcttatcaatcgcagcaaagt		fig|127906.87.repeat.142				repeat region		
2	CDS	722994	723215	+	atgtctgaaaatcaaaacgttaa	MSENQNVKRAEPIETCSYKSP	fig|127906.87.peg.3431	VCCA0713	VCA0426		hypothetical protein	Select seq ref|WP_071189391.1| DUF1289 domain-containing protein [Vibrio cholerae]	
									VCA0427				
2	CDS	723579	724004	+	gtggaaaaaagtaacgagaaa	MEKSNEKVQLLVNAIRFRKPY	fig|127906.87.peg.3432	VCCA0714	VCA0428		hypothetical protein		
2	repeat	724049	724180	+	cgcacctaacaaacgcctcaagagggactgtcaacgcgtggcgtttccagt		fig|127906.87.repeat.143				repeat region		
2	repeat	724685	724817	+	cttaagcataacaaacgcctcaagagggactgtcaacgcgtagcgtttccag		fig|127906.87.repeat.144				repeat region		
2	CDS	724752	725147	+	gtggttacggttgttgtgtttgagtt	MVTVVVFEFGVMRCQPLRRA	fig|127906.87.peg.3433	VCCA0715	VCA0431		hypothetical protein	DUF4144 domain-containing protein [Vibrio cholerae]	Limited query/subject cover for certain strains
2	repeat	725154	725283	+	taaacataacaaacgcctcaagagggactgtcaacgcgcggcgtttccagt		fig|127906.87.repeat.145				repeat region		
2	CDS	725366	725755	+	atgtttagtcacatcatgatcggc	MFSHIMIGSNDIEKSKVFYDAIL	fig|127906.87.peg.3434	VCCA0716	VCA0432		Lactoylglutathione lyase and related lyases		
2	repeat	725765	725889	+	tataacaaacgcctcaaagggactgccaacgcgtggcgtttccagtcccatt		fig|127906.87.repeat.146				repeat region		
2	CDS	725909	726766	+	atgtacaaatatatttcgaatgag	MYKYISNELPQFFENGMLRFT	fig|127906.87.peg.3435	VCCA0717	VCA0433		hypothetical protein	DUF2971 domain-containing protein [Vibrio cholerae]	
2	repeat	726763	726888	+	ctaacaaacgcctcaagagggactgtcaacgcgtggcgtttccagtcccatt		fig|127906.87.repeat.147				repeat region		
2	CDS	727059	726943	-	ttgaagcatcgaggcttgtgggtt	MKHRGLWVFSGFEPAELSTLS	fig|127906.87.peg.3436	VCCA0718	VCA0434		hypothetical protein		
2	CDS	727065	727241	+	ttggatgttccacacggcaagttt	MDVPHGKFGFTKGCIIAVILLS	fig|127906.87.peg.3437	VCCA0719	VCA0435		hypothetical protein	Select seq ref|WP_001889238.1| DUF645 family protein [Vibrio cholerae]	
2	repeat	727297	728070	+	ctaacaaacgcctcaaagggactgccaacgcgtggcgtttccagtcccaat		fig|127906.87.repeat.148				repeat region		
2	CDS	727390	727950	+	atgcgttgtcagccccttaggcg	MRCQPLRRALCSYLSMDEKV	fig|127906.87.peg.3438	VCCA0720	VCA0436		Acetyltransferase		
2	CDS	728335	728451	+	gtggccaacttaaccttgatcgtt	MANLTLIVLSFGSQRHSFWRW	fig|127906.87.peg.3439	VCCA0721	VCA0438		hypothetical protein	Select seq gb|ORP22373.1| Tfp pilus assembly protein [Vibrio cholerae]	
2	repeat	728479	728605	+	agctaacaaacgcctcaagagggactgtcaacgcgtggcgtttccagtccc		fig|127906.87.repeat.149				repeat region		
2	CDS	728639	729643	+	gtgttatatgccaaagctttaagta	MLYAKALSIGDKIGFFSPSSPA	fig|127906.87.peg.3440	VCCA0722	VCA0439		Microcin C7 immunity MccF-like protein		
2	repeat	729635	729764	+	gcaaaataacaaacgcctcaagagggactgtcaacgcgtggcgtttccagt		fig|127906.87.repeat.150				repeat region		
2	CDS	729796	730242	+	atgacagacgaaataagacaa	MTDEIRQDFIKRIDELRIQITSR	fig|127906.87.peg.3441	VCCA0723	VCA0440		putative membrane protein		
2	repeat	730240	730369	+	taacaaacgcctcaagagggactgtcaacgcgtggcgtttccagtcccattg		fig|127906.87.repeat.151				repeat region		
2	CDS	730362	731291	+	atgtgtttacagaggtatcgtgga	MCLQRYRGIKMDDLRRKCFF	fig|127906.87.peg.3442	VCCA0724	VCA0441		hypothetical protein		
2	repeat	731288	731830	+	ataacaaacgcttcaagagggacagccaacgcgtggcatttttattatgcgtt		fig|127906.87.repeat.152				repeat region		
2	CDS	731426	731902	+	atgaacttagaagagtttcaaga	MNLEEFQESDFDLLIKWIDSDE	fig|127906.87.peg.3443	VCCA0725	VCA0442		Acetyltransferase		
2	repeat	731897	732684	+	ttataacaaacgcctcaagagggactgtcaacgcgtggcgtttccagtccca		fig|127906.87.repeat.153				repeat region		
2	CDS	732039	732554	+	atgagagctatctttttgattctttg	MRAIFLILCSVLLNGCLGMPES	fig|127906.87.peg.3444	VCCA0726	VCA0443		Lipocalin Blc		
2	CDS	732690	732571	-	atgaagtttaacgcccgcctaag	MKFNARLRGWQRITTQLKHNI	fig|127906.87.peg.3445	VCCA0727	Not present		hypothetical protein		
2	CDS	732980	732690	-	atgacctataagcttgagttcaaa	MTYKLEFKKSALKEWKKLAVP	fig|127906.87.peg.3446	VCCA0728	VCA0444		mRNA interferase RelE		
2	CDS	733218	732970	-	atgaccactagaatcctagctga	MTTRILADVAASITEFKANPMK	fig|127906.87.peg.3447	VCCA0729	VCA0445		RelB/StbD replicon stabilization protein (antitoxin to RelE/StbE)		
2	repeat	733274	733402	+	tttaacaaacgcctcaagagggactgtcaacgcgcggcgtttccagtcccaa		fig|127906.87.repeat.154				repeat region		
2	CDS	733483	733674	+	atgccagatttaacgaaagctcg	MPDLTKARCPCRGCNKVANN	fig|127906.87.peg.3448	VCCA0730	VCA0446		hypothetical protein	hemagglutinin [Vibrio cholerae]	
2	CDS	733789	734442	+	atgtcaaaaatttatcaaatggat	MSKIYQMDAVDWLKTLENCSV	fig|127906.87.peg.3449	VCCA0731	VCA0447	mbolIM	haemagglutinin associated protein		
2	repeat	734449	734580	+	ttatgcctaacaaacgcctcaagagggactgtcaacgcgtggcgtttccagt		fig|127906.87.repeat.155				repeat region		
2	CDS	734515	734649	+	gtggttacagttgttgtgtttgagtt	MVTVVVFEFSVIRCQPLRRAL	fig|127906.87.peg.3450	VCCA0732	Not present		conserved hypothetical protein	Select seq emb|CSE06985.1| putative acetyltransferase [Vibrio cholerae]	
2	CDS	734714	735139	+	gtggagaaaagtaacgagaaa	MEKSNEKVQLLVNAIRFRKPY	fig|127906.87.peg.3451	VCCA0733	VCA0448		hypothetical protein		
2	repeat	735189	735312	+	ctaacaaacgcctcaagaaggactgtcaacgcgtggcgtttccagtcccatt		fig|127906.87.repeat.156				repeat region		
2	CDS	735347	735742	+	atgaaatgtttttagctttttggctc	MKCFLAFWLVFVSFFSVGLHA	fig|127906.87.peg.3452	VCCA0734	VCA0449		hypothetical protein	DUF3465 domain-containing protein [Vibrio cholerae]	
2	repeat	735393	735875	+	cggtcggcttgcatgccaatgacgccgttttacaacaagcttatcaatcgcaa		fig|127906.87.repeat.157				repeat region		
2	CDS	735893	736423	+	atggaaaagagaagaagaca	MEKRRRQKYSLLEPIVVQSGN	fig|127906.87.peg.3453	VCCA0735	VCA0449		hypothetical protein	HEPN domain-containing protein [Vibrio cholerae]	
2	repeat	736416	736545	+	aaacataacaaacgcctcaagagggactgtcaacgcgtagcgtttccagtc		fig|127906.87.repeat.158				repeat region		
2	CDS	736561	737061	+	atgaaaaatattaaatacatttta	MKNIKYILFICVVAYMPFVSAN	fig|127906.87.peg.3454	VCCA0736	VCA0450		DNA for RVC repeated sequence		
2	CDS	737217	737615	+	atgttaacctttaaaaatgtttcac	MLTFKNVSRMLMSLVLVGASF	fig|127906.87.peg.3455	VCCA0737	Not present		hypothetical protein		
2	CDS	738179	737715	-	atgacagaatcagcacataata	MTESAHNKTKRKLINNIKDIKP	fig|127906.87.peg.3456	VCCA0738	VCA0453		hypothetical protein		
2	CDS	739410	738562	-	ttgggcaagccaaaaaaacaa	MGKPKKQHIKINQSHGGSGKC	fig|127906.87.peg.3457	VCCA0739	VCA0454		Sulfate-binding protein Sbp		
2	CDS	739547	739377	-	atgcagaaattactttagttttaa	MQKLLLVLTVLTFGSTALANDS	fig|127906.87.peg.3458	VCCA0740	Not present	sbp	Sulfate and thiosulfate binding protein CysP		
2	repeat	739653	739786	+	taaacctaacaaacgcctcaagagggactgtcaacgcgtggcgtttccagt		fig|127906.87.repeat.159				repeat region		
2	CDS	739798	740376	+	atgaacattggtatctatattacg	MNIGIYIYDEAEVLDFSGPFEV	fig|127906.87.peg.3459	VCCA0741	VCA0455	inhA	Intracellular protease		
2	repeat	740369	741033	+	gcacataacaaacgcctcaatagggactgtcaacgcgtggcgtttccagtc		fig|127906.87.repeat.160				repeat region		
2	CDS	740680	740850	+	atgttcaattcggtcaacttggttt	MFNSVNLVSQKATSSLKSCFF	fig|127906.87.peg.3460	VCCA0742	VCA0456		hypothetical protein	Select seq ref|WP_001882300.1| DUF645 family protein [Vibrio cholerae]	Limited query/subject cover in certain strains
2	CDS	741173	741787	+	atgaaaaatatcaaatttaaaat	MKNIKFKIYHPTLDLVSDRTLR	fig|127906.87.peg.3461	VCCA0743	VCA0457		hypothetical protein		Limited query/subject cover in certain strains
2	CDS	741934	742791	+	atgtcagatggtaattctgttatta	MSDGNSVINFGDLAKPATVLIE	fig|127906.87.peg.3462	VCCA0744	VCA0458		hypothetical protein	DUF2806 domain-containing protein [Vibrio cholerae]	
2	CDS	742947	743498	+	atgaaaaggattctaagttttaca	MKRILSFTLIAAIQGCANEPSV	fig|127906.87.peg.3463	VCCA0745	VCA0459		hypothetical protein	putative lipoprotein [Vibrio cholerae]	
2	CDS	743507	743659	+	ttgagccgctgcataacaaacg	MSRCITNASRGTVNAWRFQS	fig|127906.87.peg.3464	VCCA0746	Not present		hypothetical protein	Select seq ref|WP_099607149.1| acetyltransferase [Vibrio cholerae]	
2	repeat	743517	744122	+	gcataacaaacgcctcaagagggactgtcaacgcgtggcgtttccagtccc		fig|127906.87.repeat.161				repeat region		
2	CDS	743785	743988	+	atggaattattcgaaaaacgtttg	MELFEKRLFYIGISFFILIPALKD	fig|127906.87.peg.3465	VCCA0747	VCA0460		hypothetical protein	putative membrane protein [Vibrio cholerae O1 str. 116059]	
2	CDS	744119	744244	+	atgcttaatccattaaaatcaagt	MLNPLKSSVCGFRFLWHFVHV	fig|127906.87.peg.3466	VCCA0748	VCA0461		hypothetical protein		
2	CDS	744269	744442	+	atgttgagcctgcgcgaggtgag	MLSLREVSWALSSRFEQFGAE	fig|127906.87.peg.3467	VCCA0749	Not present		hypothetical protein	Select seq ref|WP_050923796.1| DUF3709 domain-containing protein [Vibrio cholerae]	
2	repeat	744633	744760	+	cataacaaacgcctcaagagggactgtcaacgcgtggcgtctccagtccca		fig|127906.87.repeat.162				repeat region		
2	CDS	744802	745071	+	atgcaagtatctgatataggtttta	MQVSDIGLILDMLAVFLLAKYS	fig|127906.87.peg.3468	VCCA0750	Not present		hypothetical protein		
2	repeat	745080	745206	+	gctaacaaacgcctcaagagggactgtcaacgcgtagcgtttccagtccca		fig|127906.87.repeat.163				repeat region		
2	repeat	745615	748288	+	agctaacaaacgcctcaagagggactgtcaacgcgtggcgtttccagtccc		fig|127906.87.repeat.164				repeat region		
2	CDS	745769	746155	+	atgaaaattagtcaccttgatcat	MKISHLDHLVLTVADIPTTTNF	fig|127906.87.peg.3469	VCCA0751	VCA0463		biphenyl-2,3-diol 1,2-dioxygenase III-related protein		
2	CDS	746222	746599	+	ttgttgtggttgagtttagtggtaat	MLWLSLVVMRCQPLRRALALF	fig|127906.87.peg.3470	VCCA0752	Not present		FIG032766: hypothetical protein	pullulanase [Vibrio cholerae]	
									VCA0464				
2	CDS	746743	747147	+	atgcgacctaggaaaaaggag	MRPRKKEFRVEKIIDELVAQGY	fig|127906.87.peg.3471	VCCA0753	VCA0465		hypothetical protein		
2	CDS	747327	747548	+	atgtctgaaaatcaaaacgttaa	MSENQNVKRAEPIETCSYKSP	fig|127906.87.peg.3472	VCCA0754	Not present		hypothetical protein	DUF1289 domain-containing protein [Vibrio cholerae]	
2	CDS	747545	747736	+	atgaagcatcttagtcttgtgtata	MKHLSLVYMMVFYRMLFGINA	fig|127906.87.peg.3473	VCCA0755	VCA0466		biphenyl-2,3-diol 1,2-dioxygenase III-related protein		
2	CDS	747714	747827	+	gtggttacggttgttgtgattgagt	MVTVVVIEFGVMRCQPLRRAL	fig|127906.87.peg.3474	VCCA0756	Not present		hypothetical protein	Select seq ref|WP_119474912.1| acetyltransferase [Vibrio cholerae]	
2	CDS	748344	748676	+	atgaaaagtgtatttgtcgaatca	MKSVFVESTIFEKYRDEYLSDI	fig|127906.87.peg.3475	VCCA0757	VCA0468		RelE-like translational repressor toxin		
2	CDS	748663	748977	+	atgagcaatcgtgatttatttgcag	MSNRDLFAELSSALVEAKQHS	fig|127906.87.peg.3476	VCCA0758	VCA0469		Antitoxin to RelE-like translational repressor toxin		
2	repeat	748973	749099	+	tataacaaacgcctcaagagggactgtcaacgcgtggcgtttccagtcccat		fig|127906.87.repeat.165				repeat region		
2	CDS	749114	749548	+	gtggttacatttagggaaatggat	MVTFREMDISDYNEVIALWAT	fig|127906.87.peg.3477	VCCA0759	VCA0470		Acetyltransferase		
2	repeat	749558	749684	+	ttataacaaacgcctcaagagggactgtcaacgcacggcgtttccagtccca		fig|127906.87.repeat.166				repeat region		
2	CDS	749716	749871	+	atgaaaaagacattagggatttt	MKKTLGILILTLLSVPAYSAIRN	fig|127906.87.peg.3478	VCCA0760	VCA0471		hypothetical protein	DUF1311 domain-containing protein [Vibrio cholerae]	
2	repeat	749850	751046	+	ggaagctgcgattaagtggtgactgaactgctccaagtcgcacgacagtgc		fig|127906.87.repeat.167				repeat region		
2	CDS	750976	749996	-	atgagtcatcaactgactttcgca	MSHQLTFADGEFSNKRRQTR	fig|127906.87.peg.3479	VCCA0761	VCA0472		Mobile element protein	Select seq ref|WP_000019370.1| IS5-like element ISVch5 family transposase [Vibrio cholerae]	
2	CDS	751104	751316	+	atgcctggaataacgattggtga	MPGITIGDGAIVASKSVVTQDV	fig|127906.87.peg.3480	VCCA0762	VCA0473	vatD	Acetyltransferase		Limited query/subject cover in certain strains
2	repeat	751346	751891	+	cataacaaacgcctcaagagggactgtcaacgcgtggcgtttccagtccca		fig|127906.87.repeat.168				repeat region		
2	CDS	751487	752179	+	atgaacttagaagagtttcaaga	MNLEEFQESDFDLLIKWIDSDE	fig|127906.87.peg.3481	VCCA0763	VCA0474		Acetyltransferase		
2	CDS	752179	752580	+	atggatatcatctgttttcctttgaa	MDIICFPFERVIEINAFILKTEPC	fig|127906.87.peg.3482	VCCA0764	VCA0475		doc protein		
2	repeat	752581	753309	+	acataacaaacgcttcaagagggacagccaacgcgtggcatttttaccatg		fig|127906.87.repeat.169				repeat region		
2	CDS	752768	753181	+	atgtctaaagtcgttcaacagca	MSKVVQQQIGNIALVVENYDD	fig|127906.87.peg.3483	VCCA0765	VCA0476		Glyoxalase family protein		
2	CDS	753377	753646	+	atgcttatatggtgttttatgagac	MLIWCFMRQVLANCSASISEL	fig|127906.87.peg.3484	VCCA0766	VCA0477		RelB/StbD replicon stabilization protein (antitoxin to RelE/StbE)		
2	CDS	753636	753923	+	atgacctataagttaaagtttctg	MTYKLKFLPAAQKEWSKLAPT	fig|127906.87.peg.3485	VCCA0767	VCA0478		mRNA interferase RelE		
2	repeat	753930	754626	+	aacaaacgcctcaagagggactgtcaacgcgtggcgtttccagtcccattga		fig|127906.87.repeat.170				repeat region		
2	CDS	754051	754506	+	atgaggaatatggatatcaaatt	MRNMDIKLRPIESEEFDSLFSV	fig|127906.87.peg.3486	VCCA0768	VCA0479		Histone acetyltransferase HPA2		
2	CDS	754828	754980	+	ttggtttcacaaaggctgcatcat	MVSQRLHHRCDLVFFVVDSKI	fig|127906.87.peg.3487	VCCA0769	VCA0480		hypothetical protein	Select seq ref|WP_001884520.1| DUF645 family protein [Vibrio cholerae]	
2	repeat	755035	756169	+	aactaacaaacgcctcaagagggactgtcaacgcgtagcgtttccagtccc		fig|127906.87.repeat.171				repeat region		
2	CDS	755679	755182	-	atgatgaatacggtacttctcgat	MMNTVLLDKDKHDRNRFNCG	fig|127906.87.peg.3488	VCCA0770	VCA0481		FIG001353: Acetyltransferase		
2	CDS	755948	755676	-	atggcaacgactttgcctcgcatc	MATTLPRITARVDVDTQDLLAK	fig|127906.87.peg.3489	VCCA0771	VCA0482		FIG032766: hypothetical protein	DUF1778 domain-containing protein [Vibrio cholerae]	
2	CDS	756179	756847	+	atgagtaaacagtacgataaag	MSKQYDKEEYIKKFETDKSHS	fig|127906.87.peg.3490	VCCA0772	VCA0483		hypothetical protein	Select seq gb|EKG45296.1| putative membrane protein [Vibrio cholerae HC-41A1]	
2	repeat	756846	756981	+	aaattcatataacaaacgcctcaagagggactgtcaacgcgtggcgtttcca		fig|127906.87.repeat.172				repeat region		
2	CDS	756999	757286	+	ttggaaaatgcattaaatgccata	MENALNAIAEAIICTNADSVAY	fig|127906.87.peg.3491	VCCA0773	VCA0484		hypothetical protein		Limited query/subject cover in certain strains
2	repeat	757281	757408	+	gcataacaaacgcctcaagagggactgtcaacgcgtggcgtttccagtccc		fig|127906.87.repeat.173				repeat region		
2	CDS	757427	757798	+	atgcaacatcttgataaattgcttg	MQHLDKLLEVAKRKSEFDKNN	fig|127906.87.peg.3492	VCCA0774	VCA0485		MazG-related protein		
2	repeat	757786	757920	+	agcacacacataacaaacgcctcaagagggactgtcaacgcgtggcgtttc		fig|127906.87.repeat.174				repeat region		
2	CDS	758021	758290	+	atggcaacggcacgtttagatat	MATARLDIRLDEEIKAKAEKAS	fig|127906.87.peg.3493	VCCA0775	VCA0486		FIG032766: hypothetical protein	DUF1778 domain-containing protein [Vibrio cholerae]	
2	CDS	758287	758820	+	gtgagttggggcaaagagtttgt	MSWGKEFVELSKSKHDRDSF	fig|127906.87.peg.3494	VCCA0776	VCA0487		FIG001353: Acetyltransferase		
2	repeat	758829	758955	+	tataacaaacgcttcaagagggacagccaacgcgcggcatttttactatgcg		fig|127906.87.repeat.175				repeat region		
2	CDS	759022	759279	+	atgaaagtagaacttgtgacgtc	MKVELVTSLKRQATKILADLHD	fig|127906.87.peg.3495	VCCA0777	VCA0488		Prevent host death protein, Phd antitoxin		
2	CDS	759267	759569	+	atggttgaaataatttggacgga	MVEIIWTELALSDLNDIAEYIAL	fig|127906.87.peg.3496	VCCA0778	VCA0489	relE2	Death on curing protein, Doc toxin		
2	repeat	759584	759716	+	gctaacaaacgcctcaagagggactgtcaacgcgtggcgtttccagtccaa		fig|127906.87.repeat.176				repeat region		
2	CDS	759707	760720	+	atgtttaatcagtatcacaaattg	MFNQYHKLPLFLYTVLCVRLS	fig|127906.87.peg.3497	VCCA0779	VCA0490		Lipase, GDXG family		
2	repeat	760717	762572	+	ttaaacataacaaacgcctcaagagggactgtcaacgcgcggcgtttccag		fig|127906.87.repeat.177				repeat region		
2	CDS	760793	761266	+	ttgttgtgtttgagtttagtggtaatc	MLCLSLVVMRCQPLRRALNAC	fig|127906.87.peg.3498	VCCA0780	VCA0491		FIG032766: hypothetical protein		Limited query/subject cover in certain strains
2	CDS	761402	761686	+	atggaaatcaaagagtttagtgc	MEIKEFSACNLESLRKLYLDSF	fig|127906.87.peg.3499	VCCA0781	VCA0492		Acetyltransferase		Limited query/subject cover in certain strains
2	CDS	761730	762167	+	atgggcgactacagaagttcatc	MGDYRSSSHVYWRCKYHIVW	fig|127906.87.peg.3500	VCCA0782	VCA0493	tnpA	Mobile element protein	Select seq ref|WP_000503164.1| MULTISPECIES: IS200/IS605-like element IS1004 family transposase [Vibrio]	
2	CDS	762282	762449	+	atgctgcttaacggtgcaaaaat	MLLNGAKMKYGNLSLKCMVQ	fig|127906.87.peg.3501	VCCA0783	VCA0494		Acetyltransferase		
2	CDS	762624	763496	+	gtggtttcacgaagcgctccactt	MVSRSAPLPRLDINGRAFVYL	fig|127906.87.peg.3502	VCCA0784	VCA0495		hypothetical protein	DUF3800 domain-containing protein [Vibrio cholerae]	
2	repeat	763503	763628	+	aaactaacaaacgcctcaaagggactatcaacgcgtggcgtttccagtccc		fig|127906.87.repeat.178				repeat region		
2	CDS	763571	764245	+	atggttgttttgtttgagtttagtggt	MVVLFEFSGNALPAPWAGVM	fig|127906.87.peg.3503	VCCA0785	VCA0496		Maleylacetoacetate isomerase (EC 5.2.1.2) @ Glutathione S-transferase		
2	repeat	764240	764366	+	acataacaaacgcctcaagagggactgtcaacgcgtagcgtttccagtccc		fig|127906.87.repeat.179				repeat region		
2	CDS	764437	764706	+	atgccagagattgatgccctatta	MPEIDALLGLSFCLYFFDNKQH	fig|127906.87.peg.3504	VCCA0786	VCA0497		hypothetical protein	DUF4160 domain-containing protein [Vibrio cholerae]	
2	CDS	764700	765221	+	atgttgaaagtcatagatgttgat	MLKVIDVDFVSDHTLELTFNDC	fig|127906.87.peg.3505	VCCA0787	VCA0498		hypothetical protein	DUF2442 domain-containing protein [Vibrio cholerae]	
2	repeat	765211	765883	+	cacgcatataacaaacgcctcaagagggactgtcaacgcgtggcgtttcca		fig|127906.87.repeat.180				repeat region		
2	CDS	765282	765431	+	ttgcagttgttgtgtttgagtttagtg	MQLLCLSLVVVRCQPLRRALV	fig|127906.87.peg.3506	VCCA0788	VCA0499		conserved hypothetical protein	tRNA (guanine(37)-N(1))-methyltransferase [Vibrio mimicus]	Limited query/subject cover in certain strains
2	CDS	765896	766291	+	atgaacgattatgaccagaactc	MNDYDQNSLKILVVGPGKEFF	fig|127906.87.peg.3507	VCCA0789	VCA0501		hypothetical protein		

2	repeat	766283	768429	+	cgcagctaacaaacgcctcaagagggactgtcaacgcgtggcgtttccagt		fig|127906.87.repeat.181				repeat region		
2	CDS	766708	766430	-	atgatttctgggaagaagcatc	MIFWEEASLNDREKIFEFLYDF	fig|127906.87.peg.3508	VCCA0790	VCA0503		Death on curing protein, Doc toxin		
2	CDS	766989	766705	-	atggatactagaattcaatttcgt	MDTRIQFRVDEETKRLAQQMA	fig|127906.87.peg.3509	VCCA0791	VCA0504		Antitoxin DinJ (binds YafQ toxin)		
2	CDS	767105	767698	+	gtggttacggttgttgtgtttgagtt	MVTVVVFEFSVMRCQPLRRA	fig|127906.87.peg.3510	VCCA0792	VCA0505		Acetyltransferase		Limited query/subject cover in certain strains
2	CDS	767882	768295	+	atgtctaaagtcgttcaacagca	MSKVVQQQIGNIALVVENYDD	fig|127906.87.peg.3511	VCCA0793	VCA0506		Glyoxalase family protein		
2	CDS	768513	768782	+	atggcatgtagttgttgtggaaaa	MACSCCGKRLNVGTIHKSDPK	fig|127906.87.peg.3512	VCCA0794	Not annotated as CDS		hypothetical protein		
2	repeat	768777	770038	+	ttgtagtggcatagtgaatttggtcacttagttagaggtgatatcatcacctcata		fig|127906.87.repeat.182				repeat region		
2	CDS	768850	769158	+	atgacaaaacgtacaagacga	MTKRTRRLFSAEFKLEAAQLV	fig|127906.87.peg.3513	VCCA0795	VCA0507		Transposase InsN for insertion sequence element IS911		
2	CDS	769155	770027	+	ttgataatcaagaaactcaagca	MIIKKLKQSHSVKILCEVFNVHI	fig|127906.87.peg.3514	VCCA0796	VCA0508		Transposase InsO for insertion sequence element IS911		
2	CDS	770184	770065	-	atggctactaaaagtattgaaat	MATKSIEMRKTRRHWGIDTVM	fig|127906.87.peg.3515	VCCA0797	VCA0509		hypothetical protein		
2	CDS	770350	773223	+	atgtactcatctcatcaatgtaaa	MYSSHQCKRLSIMAILGCLPFS	fig|127906.87.peg.3516	VCCA0798	VCA0510		hypothetical protein	Type I phosphodiesterase / nucleotide pyrophosphatase [Vibrio cholerae]	
2	CDS	773686	775806	+	gtgaaacctatcgtaatcaaacg	MKPIVIKRDGSKAPFNRDRIQA	fig|127906.87.peg.3517	VCCA0799	VCA0511	nrdD	Ribonucleotide reductase of class III (anaerobic), large subunit (EC 1.17.4.2)		
2	CDS	775811	776278	+	atgaattaccatcaatattatccg	MNYHQYYPIDVVNGPGTRCTL	fig|127906.87.peg.3518	VCCA0800	VCA0512	nrdG	Ribonucleotide reductase of class III (anaerobic), activating protein (EC 1.97.1.4)		
2	CDS	776400	777581	+	atgtttactcatttaccagcacca	MFTHLPAPVLDPILSLSVAFRN	fig|127906.87.peg.3519	VCCA0801	VCA0513		Aspartate aminotransferase (EC 2.6.1.1)		
2	CDS	777639	778322	+	atgagtcaaccactgaagttgtta	MSQPLKLLVPLMLSSGLVACA	fig|127906.87.peg.3520	VCCA0802	VCA0514		hypothetical protein	Select seq ref|WP_000082072.1| ATP-dependent zinc protease [Vibrio cholerae]	
2	CDS	778518	778402	-	atgctcagtgaaaatgaaaaag	MLSENEKASEIPLAFLYLNNFF	fig|127906.87.peg.3521	VCCA0803	VCA0515		FIG01202519: hypothetical protein		
2	CDS	780292	778556	-	atgaaaatagccattgtaaccgc	MKIAIVTACPSGVANSIIAAGLL	fig|127906.87.peg.3522	VCCA0804	VCA0516	fruA	PTS system, fructose-specific IIB component (EC 2.7.1.202) / PTS system, fructose-specific IIB component (EC 2.7.1.202) / PTS system, fructose-specific IIC component		
2	CDS	781264	780311	-	atgacaaaaaaagtggtcacca	MTKKVVTITLNPALDLTGSVNC	fig|127906.87.peg.3523	VCCA0805	VCA0517	lacC/fruK	1-phosphofructokinase (EC 2.7.1.56)		
2	CDS	782477	781275	-	atgttagaactcactacacaaga	MLELTTQDIQLQQHFANKQAA	fig|127906.87.peg.3524	VCCA0806	VCA0518	fruB	PTS system, fructose-specific IIA component (EC 2.7.1.202) / Fructose-specific phosphocarrier protein HPr		
2	CDS	782816	783796	+	atgacactggatgaaatcgcga	MTLDEIAKLAGVSKTTASYVIN	fig|127906.87.peg.3525	VCCA0807	VCA0519	cra/fruR	Fructose repressor FruR, LacI family		
2	CDS	783850	784989	+	atgaagtttctacatacttcggatt	MKFLHTSDWHLGRQFHQVSL	fig|127906.87.peg.3526	VCCA0808	VCA0520		Exonuclease SbcD		
2	CDS	784991	788032	+	atgcgtccgttaaaacttatcctc	MRPLKLILQAFGPFAGREEIDF	fig|127906.87.peg.3527	VCCA0809	VCA0521	sbcC	Exonuclease SbcC		
2	CDS	788207	790189	+	atgcttggctttcctgcctactactt	MLGFPAYYFIWEYWFPQSYEN	fig|127906.87.peg.3528	VCCA0810	VCA0522	cqsS/luxN	CAI-1 autoinducer sensor kinase/phosphatase CqsS		
2	repeat	790187	790320	+	tagctataccccttcctacttgaagctgcaggggtgttggctacgttcgctcacc		fig|127906.87.repeat.183				repeat region		
2	CDS	791587	790418	-	atgaacaagcctcaacttcctga	MNKPQLPDFIQNKIDHYIENYF	fig|127906.87.peg.3529	VCCA0811	VCA0523	cqsA	CAI-1 autoinducer synthase CqsA		
2	CDS	792746	791784	-	ttgaattggcatagatttacccaa	MNWHRFTQVQNVPPSQAALA	fig|127906.87.peg.3530	VCCA0812	VCA0524		Tellurite resistance protein		
2	CDS	794299	792893	-	atgtcaacaagagagacgttta	MSTRETFKISLLAKMPKDVINC	fig|127906.87.peg.3531	VCCA0813	VCA0526	clcA	Voltage-gated H(+)/2Cl(-) exchange transporter ClcA		
2	CDS	794766	795755	+	atgatcaatcgagttccgaccaa	MINRVPTNIITGFLGVGKTTAIL	fig|127906.87.peg.3532	VCCA0814	VCA0527	yjiA	Zinc-binding GTPase YeiR		
2	CDS	796763	795819	-	atggttaacaactcaatccaatg	MVNNSIQWFPGHMHKARKEIE	fig|127906.87.peg.3533	VCCA0815	VCA0528	rbgA	LSU ribosomal maturation GTPase RbgA (B. subtilis YlqF)		
2	CDS	797086	798948	+	atgaaacctgcaaaacaaacg	MKPAKQTTASLAFLAMGIVYG	fig|127906.87.peg.3534	VCCA0816	VCA0529	kup	Kup system potassium uptake protein		
2	CDS	802285	799046	-	atgacttggattactgacgcaaa	MTWITDANTAVARVAYQLSEV	fig|127906.87.peg.3535	VCCA0817	VCA0530	nifJ/ydbK	Pyruvate-flavodoxin oxidoreductase		
2	CDS	802388	803881	+	gtggtgacaagacttaatgaag	MVTRLNEAHKALILVALYAMVT	fig|127906.87.peg.3536	VCCA0818	VCA0531	kdpD	Osmosensitive K+ channel histidine kinase KdpD		
2	CDS	803871	804563	+	atgaacagtgaaattaagatcct	MNSEIKILIIEDEAPIQRFLTVLIS	fig|127906.87.peg.3537	VCCA0819	VCA0532	kdpE	DNA-binding response regulator KdpE		
2	CDS	804763	804999	+	atgggtggaatcagtgtgggtaa	MGGISVGKLLILGCIVALVFGTI	fig|127906.87.peg.3538	VCCA0820	VCA0533	tatA	Twin-arginine translocation protein TatA		
2	CDS	805889	805215	-	ttggattccgtactctctatttttagt	MDSVLSIFSALWHHDAFALQN	fig|127906.87.peg.3539	VCCA0821	VCA0534	yghB	DedA protein		1 ORF split into 2 ORFs in N16961, none refseq annotated. Annotated as singel ORF pseudogene in KEGG
									VCA0534				1 ORF split into 2 ORFs in N16961, none refseq annotated. Annotated as singel ORF pseudogene in KEGG
2	CDS	806432	806052	-	atgttgcatgtagtgacatcactg	MLHVVTSLLSFILLCAAPLLHG	fig|127906.87.peg.3540	VCCA0822	VCA0535		hypothetical protein		
2	CDS	807363	806566	-	atggttctgactgataaacaaca	MVLTDKQQFLDCIHYDEGGEY	fig|127906.87.peg.3541	VCCA0823	VCA0536	ykuI	Diguanylate cyclase/phosphodiesterase domain 2 (EAL)		
									VCA0537		hypothetical protein		1 ORF split into 2 ORFs in N16961, only last ORF refseq annotated in PATRIC. Annotated as 1 pseudogene ORF named VCA0537 in KEGG.
2	CDS	808394	807759	-	ttgctactggatatcgtcacgcaa	MLLDIVTQSVNEFQVDCLRLC	fig|127906.87.peg.3542	VCCA0824	VCA0537		hypothetical protein		1 ORF split into 2 ORFs in N16961, only last ORF refseq annotated in PATRIC. Annotated as 1 pseudogene ORF named VCA0537 in KEGG.
2	CDS	808781	809326	+	atgaaaaattcagacagccaac	MKNSDSQHYNLVTRSIHWISA	fig|127906.87.peg.3543	VCCA0825	VCA0538		Cytochrome b561 homolog 2		
2	CDS	809323	809892	+	atgaaaaaaacactaatggcag	MKKTLMAVGLAAVMSIPFAAN	fig|127906.87.peg.3544	VCCA0826	VCA0539		hypothetical protein	YceI family protein [Vibrio cholerae]	
2	CDS	811451	810000	-	atgtctgcagcctactctaaaaa	MSAAYSKNQNCFSPTEMMAE	fig|127906.87.peg.3545	VCCA0827	VCA0540		Formate efflux transporter FocA		
2	CDS	811668	812573	+	gtgcggtattcattaaagcaatta	MRYSLKQLAVFDAVADCGSVS	fig|127906.87.peg.3546	VCCA0828	VCA0542		Transcriptional regulator, LysR family	LysR family transcriptional regulator [Vibrio cholerae]	
2	CDS	812735	813247	+	atgtatgaggcggatatgagcag	MYEADMSTLFAIALTTGILSGV	fig|127906.87.peg.3547	VCCA0829	VCA0543	ycdZ	hypothetical protein	DUF1097 domain-containing protein [Vibrio cholerae]	
2	CDS	815110	813479	-	atgcatttagagcgaattgaaat	MHLERIEIAGFRGIRRLSLTFDI	fig|127906.87.peg.3548	VCCA0830	VCA0544		Predicted ATP-dependent endonuclease of the OLD family, YbjD subgroup	ATP-dependent endonuclease [Vibrio cholerae]	
2	CDS	815373	817115	+	atgcctatgagaagaaaaaatc	MPMRRKNQPASITLAHINDTH	fig|127906.87.peg.3549	VCCA0831	VCA0545		5'-nucleotidase (EC 3.1.3.5)		
2	CDS	817265	817360	+	atgttaagccgagattggttcgat	MLSRDWFDTLVVVAWVGIWS	fig|127906.87.peg.3550	VCCA0832	Not annotated as CDS		hypothetical protein	putative membrane protein [Vibrio cholerae]	
2	CDS	817675	818631	+	atgtccccaatcacttatcttggct	MSPITYLGFGLLTLTLVAVDLW	fig|127906.87.peg.3551	VCCA0833	VCA0546	alx	Integral membrane protein TerC		
2	CDS	818935	819123	+	atggctcaggcaatgcaaatgg	MAQAMQMGTMTVPTSKDKKT	fig|127906.87.peg.3552	VCCA0834	VCA0547		hypothetical protein		
2	CDS	819164	819301	+	ttgtttcttgaagggcgtatttgcta	MFLEGRICYEYASNKKKRLKR	fig|127906.87.peg.3553	VCCA0835	Not annotated as CDS		hypothetical protein		
2	CDS	819839	819273	-	atgtctactgaagcaactttacttg	MSTEATLLERCQSQCELCAAS	fig|127906.87.peg.3554	VCCA0836	VCA0549	phnA	Protein PhnA		
2	CDS	820032	821114	+	ttggaactgctttgcattgattattt	MELLCIDYLGKPLRLEGTMAG	fig|127906.87.peg.3555	VCCA0837	VCA0550	spoIVFB	Zn-dependent protease		
2	CDS	821353	821168	-	atgaacattcgtgatcgcatcga	MNIRDRIEAIEQEIYVACSEGD	fig|127906.87.peg.3556	VCCA0838	VCA0551		hypothetical protein		
2	CDS	823923	821566	-	atgaatttcaaatcttcgattctgtt	MNFKSSILLLLATVFSGCAMYA	fig|127906.87.peg.3557	VCCA0839	VCA0552		Fatty acid cis/trans isomerase		
2	CDS	824128	823955	-	ttggctctcaccaaaggcaagc	MALTKGKPMAVQICSRQICHS	fig|127906.87.peg.3558	VCCA0840	VCA0553		FIG01202458: hypothetical protein		
2	CDS	825511	824279	-	atgagcaagattgataaagctat	MSKIDKAMRILLAGFCINLCLG	fig|127906.87.peg.3559	VCCA0841	VCA0554		Oxalate/formate antiporter		
2	CDS	825685	825569	-	atgaaacaagcgaaatttattttt	MKQAKFIFYPSLKSDSNHKAIC	fig|127906.87.peg.3560	VCCA0842	VCA0555		hypothetical protein		
2	CDS	825934	826476	+	atgaagcataatgcactaaaaa	MKHNALKMALIAVLGVTSLTG	fig|127906.87.peg.3561	VCCA0843	VCA0556		hypothetical protein	DUF3332 domain-containing protein [Vibrio cholerae]	
2	CDS	826770	828155	+	atggatcatcgcttttcgaccaaa	MDHRFSTKLFLLLMIAWPLLFC	fig|127906.87.peg.3562	VCCA0844	VCA0557		Extracellular solute-binding protein, family 3/GGDEF domain protein		
2	rna	828279	828353	+	gcgggcatcgtataatggctattacctcagccttccaagctgatgatgcgggt		fig|127906.87.rna.124	VCCAt001	VCAt001		tRNA-Gly-TCC		
2	CDS	828450	830018	+	atggcgcgaattgcgtttacggc	MARIAFTAPHHKASETGLDILR	fig|127906.87.peg.3563	VCCA0845	VCA0558	ywrD	Gamma-glutamyltranspeptidase (EC 2.3.2.2) @ Glutathione hydrolase (EC 3.4.19.13)		
2	CDS	830096	831076	+	atgaagttacccttacgcctaag	MKLPLRLSFGCLLVMSFSATA	fig|127906.87.peg.3564	VCCA0846	VCA0559		hypothetical protein	Solitary outer membrane autotransporter beta-barrel domain [Vibrio cholerae]	
2	CDS	832311	831073	-	atgtcttctagctttgttacttcacc	MSSSFVTSPWFRFGFPLLLLV	fig|127906.87.peg.3565	VCCA0847	VCA0560		GGDEF domain protein		
2	CDS	832667	833041	+	atgcaattgagcgaagatgttca	MQLSEDVHNYMETLVGQVLG	fig|127906.87.peg.3566	VCCA0848	VCA0561		hypothetical protein	competence protein ComFB [Vibrio cholerae]	
2	CDS	833051	833329	+	gtgggattattttcgcgcctttttgg	MGLFSRLFGKPKQQEPVQVE	fig|127906.87.peg.3567	VCCA0849	VCA0562		hypothetical protein	Select seq gb|MVC69009.1| transcriptional regulator [Vibrio cholerae]	
2	CDS	833622	835160	+	atgcaaattggtgtaccaagaga	MQIGVPREQLAGETRVAASPS	fig|127906.87.peg.3568	VCCA0850	VCA0563	pntA	NAD(P) transhydrogenase N-domain of subunit alpha (EC 1.6.1.2) / NAD(P) transhydrogenase C-domain of subunit alpha (EC 1.6.1.2)		
2	CDS	835172	836548	+	atgtctgcaggattggtacaagc	MSAGLVQAAYIVAAVFFIMSLA	fig|127906.87.peg.3569	VCCA0851	VCA0564	pntB	NAD(P) transhydrogenase subunit beta (EC 1.6.1.2)		
2	CDS	836649	838148	+	atgcgttatagtttttgcatgttaga	MRYSFCMLEKTNIPLIRALNLT	fig|127906.87.peg.3570	VCCA0852	VCA0565		Two component sensor histidine kinase VCA0565	DUF3404 domain-containing protein [Vibrio cholerae]	
2	CDS	838123	838779	+	gtgaaacagacactgcttctcgt	MKQTLLLVEDDKNLADGLLVS	fig|127906.87.peg.3571	VCCA0853	VCA0566	arlR	Two-component transcriptional response regulator VCA0566, OmpR family		
2	CDS	838782	839666	+	atggttaagccaaatcctcttttat	MVKPNPLLWLTGMLLSTPAFA	fig|127906.87.peg.3572	VCCA0854	VCA0567		Uncharacterized protein VCA0567	Select seq ref|WP_000240569.1| DUF2861 family protein [Vibrio cholerae]	
2	CDS	839823	840893	+	atgggcggtgtcggggttgtatca	MGGVGVVSGNFLTGPFYNPA	fig|127906.87.peg.3573	VCCA0855	VCA0568		TraF-related protein		
2	CDS	840934	841095	+	atgtttgatgatctacccacattaa	MFDDLPTLTHAEQQVAVEKIQ	fig|127906.87.peg.3574	VCCA0856	VCA0569		UPF0181 protein YoaH	YoaH family protein [Vibrio cholerae]	
2	CDS	841996	841685	-	atgacattggtgtattcaaccgaa	MTLVYSTEVGRIKPEHEKIERF	fig|127906.87.peg.3575	VCCA0857	VCA0570		Translation initiation factor SUI1-related protein		
2	CDS	842387	842004	-	atggctgttgcaatctatcgaggt	MAVAIYRGFNLQSAANSTEIW	fig|127906.87.peg.3576	VCCA0858	VCA0571		hypothetical protein	DUF3319 domain-containing protein [Vibrio cholerae]	Limited query cover for certain strains
2	CDS	843563	842559	-	atgactaagacaacaatcctac	MTKTTILLLCGGGSSEHEISLV	fig|127906.87.peg.3577	VCCA0859	VCA0572	ddl	D-alanine--D-alanine ligase (EC 6.3.2.4)		
2	CDS	844289	843714	-	atgaaaaaaatcgcagttatcgg	MKKIAVIGLSALLAACASGEYT	fig|127906.87.peg.3578	VCCA0860	VCA0573		DamX-related protein		
2	CDS	844508	847189	+	atggaccacttgaatcaactgct	MDHLNQLLRPKSVAVIGASVR	fig|127906.87.peg.3579	VCCA0861	VCA0574		Protein lysine acetyltransferase Pat (EC 2.3.1.-)		
2	CDS	848126	847239	-	ttgttaagtccaatcactcttgaag	MLSPITLEALHILDAIERRGSFA	fig|127906.87.peg.3580	VCCA0862	VCA0575		Transcriptional regulator, LysR family	LysR family transcriptional regulator [Vibrio cholerae]	
2	CDS	848359	850440	+	atgtataaaaagtctctgctctcta	MYKKSLLSSAIMLALVPSAYAD	fig|127906.87.peg.3581	VCCA0863	VCA0576	hemR/hutA	TonB-dependent heme and hemoglobin receptor HutA; TonB-dependent hemin, ferrichrome receptor		
2	CDS	852461	850764	-	atgagcagcccgcgtttacgtgt	MSSPRLRVQFETLFEHFQGQ	fig|127906.87.peg.3582	VCCA0864	VCA0578		SgrR, sugar-phosphate stress, transcriptional activator of SgrS small RNA		
2	CDS	852775	852542	-	atggtgattcaatttagcggtggc	MVIQFSGGKIIVTPHEVVVRLG	fig|127906.87.peg.3583	VCCA0865	VCA0579		PTS system, IIA component		
2	CDS	853261	852776	-	atgagccactttactcgttcagg	MSHFYSFRTQAMSIWNKPISL	fig|127906.87.peg.3584	VCCA0866	VCA0580	ydiI	1,4-dihydroxy-2-naphthoyl-CoA hydrolase (EC 3.1.2.28) in menaquinone biosynthesis		
2	CDS	853396	854187	+	atgaaaaagacgcaactggcg	MKKTQLALTLLMTLGLAACSA	fig|127906.87.peg.3585	VCCA0867	VCA0581		Zn-dependent protease with chaperone function PA4632		
2	CDS	854781	854299	-	atgaccgacgagcaattcaacc	MTDEQFNPQDEEIEIEAIGVEV	fig|127906.87.peg.3586	VCCA0868	VCA0582		Uncharacterized S4 RNA-binding-domain protein YbcJ	Select seq ref|WP_071919823.1| urease [Vibrio cholerae]	
2	CDS	854897	855682	+	atgcgcattgggttactttttgcttc	MRIGLLFASLCLMSTCLHAKP	fig|127906.87.peg.3587	VCCA0869	VCA0583		ABC-type amino acid transport/signal transduction system, periplasmic component/domain		
2	CDS	856436	855768	-	atgattgatttttatactgcagcaa	MIDFYTAATPNGHKIAIALEEM	fig|127906.87.peg.3588	VCCA0870	VCA0584	yfcG	Glutathione S-transferase (EC 2.5.1.18)		
2	CDS	857059	856448	-	atgaaattatatgaaaccgcgat	MKLYETAMTPSCRRVSIFLKE	fig|127906.87.peg.3589	VCCA0871	VCA0585	dcmA	Glutathione S-transferase (EC 2.5.1.18)		
2	CDS	857161	857757	+	atggcaagaaaatgcaactttg	MARKCNFDREEKLHQAMTLF	fig|127906.87.peg.3590	VCCA0872	VCA0586		Transcriptional regulator, AcrR family	Select seq ref|WP_000101303.1| TetR/AcrR family transcriptional regulator [Vibrio cholerae]	
2	CDS	857754	858146	+	atgatccattgattgccctgcgc	MIHLIALRLTRGMDLKQQIVQL	fig|127906.87.peg.3591	VCCA0873	VCA0587		Predicted DNA-binding protein	DUF296 domain-containing protein [Vibrio cholerae]	
2	CDS	859822	858230	-	atgaacgattgggtattgaagat	MNDWVLKIDNLSVGFGIAGQA	fig|127906.87.peg.3592	VCCA0874	VCA0588	yejF	ABC transporter, ATP-binding protein YejF		
2	CDS	860874	859819	-	atggggatgacaaaagcaagc	MGMTKASKRVPNPLTEARWA	fig|127906.87.peg.3593	VCCA0875	VCA0589	yejE	ABC transporter, permease protein YejE		
2	CDS	861956	860874	-	atgctcagttatattctgcgccgtt	MLSYILRRLLLVVPTLWAIITINF	fig|127906.87.peg.3594	VCCA0876	VCA0590	yejB	ABC transporter, permease protein YejB		
2	CDS	863782	861965	-	atgcgcagttggaagccgattg	MRSWKPIALGVTLALSSQFSW	fig|127906.87.peg.3595	VCCA0877	VCA0591		ABC transporter, substrate-binding protein YejA		
2	CDS	864517	863996	-	atgaaacctctatatgtcgctata	MKPLYVAIHPDIKPLNQQRIMC	fig|127906.87.peg.3596	VCCA0878	VCA0592		MutT/Nudix family protein		
2	CDS	865542	864610	-	atgtcatcactcaagtatcgatta	MSSLKYRLVTRSDFDGLVCAV	fig|127906.87.peg.3597	VCCA0879	VCA0593		Exopolyphosphatase-related protein		
2	CDS	865782	866033	+	atgaataaaaccgccctattact	MNKTALLLTVVGAVLLTGCAR	fig|127906.87.peg.3598	VCCA0880	VCA0594	hlx	Hemolysin		
2	CDS	867068	866115	-	atgtcagcagtgtgtccgatttatg	MSAVCPIYVVLGKRLNANQLT	fig|127906.87.peg.3599	VCCA0881	VCA0595		hypothetical protein	Select seq ref|WP_001277066.1| YdcF family protein [Vibrio cholerae]	
									VCA0596				1 ORF split into 2 ORFs in N16961, both ORF refseq annotated, in PATRIC. Annotated as 1 orf pseudogene named VCA0596 in KEGG, no VCA0597 in KEGG.
2	CDS	867287	868759	+	atgtggaatagactcaataaatc	MWNRLNKSMMFCQMMFGLS	fig|127906.87.peg.3600	VCCA0882	VCA0597		Di-tripeptide/cation symporter		1 ORF split into 2 ORFs in N16961, both ORF refseq annotated, in PATRIC. Annotated as 1 orf pseudogene named VCA0596 in KEGG, no VCA0597 in KEGG.
2	CDS	869764	868859	-	atgtcttttatgcctatcatcttagta	MSFMPIILVLFSALLHAGWNIIG	fig|127906.87.peg.3601	VCCA0883	VCA0599		Permease of the drug/metabolite transporter (DMT) superfamily		
2	CDS	870534	869830	-	gtgcaatacgtaaaaattaaag	MQYVKIKDAIVEQIDAGMLMPI	fig|127906.87.peg.3602	VCCA0884	VCA0600	phnR	2-aminoethylphosphonate uptake and metabolism regulator		
2	CDS	872259	870559	-	atgttgaatgttgctcgtactttttca	MLNVARTFSVRILPHWSRDSL	fig|127906.87.peg.3603	VCCA0885	VCA0601	potH	2-aminoethylphosphonate ABC transporter permease protein		
2	CDS	873442	872270	-	atgaccatgcatcatcctaattta	MTMHHPNLQQHDRPQAISRS	fig|127906.87.peg.3604	VCCA0886	VCA0602		2-aminoethylphosphonate ABC transporter ATP-binding protein (TC 3.A.1.9.1)		
2	CDS	874586	873579	-	atgaacaagcgtatattgaaag	MNKRILKGTLTAIAALMSAHAF	fig|127906.87.peg.3605	VCCA0887	VCA0603		2-aminoethylphosphonate ABC transporter substrate-binding protein		
2	CDS	874830	875933	+	atgaagaatgcttatctactgctg	MKNAYLLLTPGPLSTSESVRE	fig|127906.87.peg.3606	VCCA0888	VCA0604	phnW	2-aminoethylphosphonate:pyruvate aminotransferase (EC 2.6.1.37)		
2	CDS	875951	877348	+	atgaaaacattcgatcccctctc	MKTFDPLSSYSPTHWRSEGD	fig|127906.87.peg.3607	VCCA0889	VCA0605	dgdA	4-aminobutyrate aminotransferase (EC 2.6.1.19)		
2	CDS	877358	878170	+	atgaactctccgattcaagccgt	MNSPIQAVIFDWAGTIVDFGSF	fig|127906.87.peg.3608	VCCA0890	VCA0606	phnX	Phosphonoacetaldehyde hydrolase (EC 3.11.1.1)		
2	CDS	878335	878742	+	atgtctcaacttaatactatcgtgg	MSQLNTIVVSTRDMDRISQLM	fig|127906.87.peg.3609	VCCA0891	VCA0607	mk	Regulator of nucleoside diphosphate kinase		
2	CDS	879477	878803	-	atgaagtacgattggatcttatttg	MKYDWILFDADETLFHFDAFK	fig|127906.87.peg.3610	VCCA0892	VCA0608	yjjG	Pyrimidine 5'-nucleotidase YjjG (EC 3.1.3.5)		
2	CDS	879678	879854	+	atgaagcgtttttcccgtcagtag	MKRFFPSVVMLIPFVVKRYPD	fig|127906.87.peg.3611	VCCA0893	VCA0609		hypothetical protein		
2	CDS	879859	880509	+	atgaagaaagtcgcggtattctc	MKKVAVILSGCGVFDGAEIHES	fig|127906.87.peg.3612	VCCA0894	VCA0610	elbB	Enhancing lycopene biosynthesis protein 2		
2	CDS	880662	880522	-	atgcgcaatctcgtcatccagaa	MRNLVIQKFYIPSIIDAAVSFVC	fig|127906.87.peg.3613	VCCA0895	No refSeq annotation		FIG01201248: hypothetical protein		
					atgacgagattgcgcataaccaaacagcgctataccccttggtaacgtaatcg		Only present in certain strains	Not annotated as CDS	VCA0611				
2	CDS	880780	881190	+	atgagcttacttaaagagttcaa	MSLLKEFKAFASRGNVIDMAV	fig|127906.87.peg.3614	VCCA0896	VCA0612	mscL	Large-conductance mechanosensitive channel		
2	CDS	881223	881336	+	ttgagcgccttttttccattttagcgc	MSAFFHFSAPNKSKNLVLQLIK	fig|127906.87.peg.3615	VCCA0897	VCA0613		FIG01202439: hypothetical protein		
2	CDS	881421	883169	+	atgctgccagatattgaaatttgc	MLPDIEICRATPLAPIDTIAQKA	fig|127906.87.peg.3616	VCCA0898	VCA0614	fhs	Formate--tetrahydrofolate ligase (EC 6.3.4.3)		
2	CDS	883345	884481	+	atgaaacgactctttgcttgggga	MKRLFAWGAPLFALVALTLSL	fig|127906.87.peg.3617	VCCA0899	VCA0615	msrAB	Peptide-methionine (S)-S-oxide reductase MsrA (EC 1.8.4.11) / Peptide-methionine (R)-S-oxide reductase MsrB (EC 1.8.4.12)		
2	CDS	885227	884574	-	atgtcaggtcttagcgaatccgc	MSGLSESAKLVKDALERRGLE	fig|127906.87.peg.3618	VCCA0900	VCA0616	folE	GTP cyclohydrolase I (EC 3.5.4.16) type 1		
2	CDS	885334	886614	+	gtgctacagtttgccgcaaaaga	MLQFAAKEQQHRTFPMGCCD	fig|127906.87.peg.3619	VCCA0901	VCA0617		Molybdopterin molybdenumtransferase (EC 2.10.1.1)		
2	CDS	886623	887381	+	gtggatattctcagcgatgcaga	MDILSDAEMLRYNRQIILKAFD	fig|127906.87.peg.3620	VCCA0902	VCA0618	moeB	Molybdopterin-synthase adenylyltransferase (EC 2.7.7.80)		
2	CDS	887565	887933	+	atgtccaatctcaacgttttggaa	MSNLNVLEIKSFVPAQDFTTSK	fig|127906.87.peg.3621	VCCA0903	VCA0619		hypothetical protein	Select seq ref|WP_101411943.1| glyoxalase [Vibrio cholerae]	
2	CDS	887955	888785	+	atgacctccccactcgtcacggc	MTSPLVTAQWLQQHLHDPNL	fig|127906.87.peg.3622	VCCA0904	VCA0620	sseA	3-mercaptopyruvate sulfurtransferase (EC 2.8.1.2)		
2	CDS	889865	888870	-	atgagccaaattccagagatttc	MSQIPEISVESTDLLTEIAVSYY	fig|127906.87.peg.3623	VCCA0905	VCA0621	deoR	Transcriptional regulator, SorC family	Select seq ref|WP_136345133.1| sugar-binding transcriptional regulator [Vibrio cholerae]	
2	repeat	890257	890382	+	cccaaactacttggagttgcaggtaggcggcaagtgagttcatccccatgag		fig|127906.87.repeat.184				repeat region		
					atgggctatccttctatggccgcggccttgcatgccgcagcactcaacattgc		From N16961	Not annotated as CDS	VCA0622		hypothetical protein		
2	CDS	890458	891408	+	atgagcaataaattagcgcaac	MSNKLAQLRKLTTVVADTGEII	fig|127906.87.peg.3624	VCCA0906	VCA0623	tal/talB	Transaldolase (EC 2.2.1.2)		
2	CDS	891505	893499	+	atgaatcgcaaacaacttgcca	MNRKQLANAIRALSMDGVQKA	fig|127906.87.peg.3625	VCCA0907	VCA0624		Transketolase (EC 2.2.1.1)		
2	repeat	891510	893585	+	tcgcaaacaacttgccaatgctattcgtgccctgagcatggacggcgttcaaa		fig|127906.87.repeat.185				repeat region		
2	CDS	895868	893616	-	atgcagttgaaacctttgttcact	MQLKPLFTLLPVVLSSVVQAQ	fig|127906.87.peg.3626	VCCA0908	VCA0625	hasR	Putative TonB-dependent heme receptor HasR	Select seq ref|WP_001882475.1| TonB-dependent receptor [Vibrio cholerae]	
2	CDS	896184	896696	+	atgaccacctcttccaccgtaatt	MTTSSTVIIGLHNPKSPTNVGA	fig|127906.87.peg.3627	VCCA0909	VCA0627		RNA methyltransferase, TrmH family		
2	CDS	897044	896925	-	ttgcaatttttagtagataggaat	MQFLVDRNPTRFESPTEQRLT	fig|127906.87.peg.3628	VCCA0910	No refSeq annotation		FIG01202027: hypothetical protein		
2	CDS	897140	899308	+	atgataaagcatagagcagag	MIKHRAEQDVFSQLEEICKSN	fig|127906.87.peg.3629	VCCA0911	VCA0628		hypothetical protein	Select seq ref|WP_001894482.1| preprotein translocase subunit SecA [Vibrio cholerae]	
2	CDS	900619	899579	-	atgtccgaaagagtttctttcaca	MSERVSFTLFIQILIIAAVAAGT	fig|127906.87.peg.3630	VCCA0912	VCA0629		Putative ammonia monooxygenase	Select seq ref|WP_000004193.1| AbrB family transcriptional regulator [Vibrio cholerae]	
2	CDS	901603	900632	-	atgaaaattgcgattctggatgat	MKIAILDDYQNVVRGLNAFQC	fig|127906.87.peg.3631	VCCA0913	VCA0630	yoaD	D-3-phosphoglycerate dehydrogenase (EC 1.1.1.95)		
2	CDS	902134	901619	-	atgccgttaactcgattaactttgc	MPLTRLTLQDEAAFAEFFQDF	fig|127906.87.peg.3632	VCCA0914	VCA0631		Acetyltransferase		
2	CDS	902616	902167	-	gtgtctgtagaaaaaaccgagc	MSVEKTEPVEKTPTRLPVAEC	fig|127906.87.peg.3633	VCCA0915	VCA0632	nudG	MutT/Nudix family protein		
2	CDS	903006	902656	-	atggcggctttgtttttcaagtgctt	MAALFFKCLLGALAVLIIALLSK	fig|127906.87.peg.3634	VCCA0916	VCA0633	ydgC/glpM	GlpM protein		
2	CDS	903198	903923	+	atgaaattaggaaaacggctga	MKLGKRLTQLVQQVKRDYDH	fig|127906.87.peg.3635	VCCA0917	VCA0634		hypothetical protein	tRNA (adenine-N(1))-methyltransferase [Vibrio cholerae]	
2	CDS	903933	904874	+	atgaaagcactcaatgatttgaa	MKALNDLNLFVETARQGSFSK	fig|127906.87.peg.3636	VCCA0918	VCA0635		Transcriptional regulator, LysR family	Select seq ref|WP_148530088.1| LysR family transcriptional regulator [Vibrio cholerae]	
2	CDS	905161	904871	-	atgatccatttactcgcagaaatc	MIHLLAEIKAFPDSAQQVQALL	fig|127906.87.peg.3637	VCCA0919	VCA0636		hypothetical protein	antibiotic biosynthesis monooxygenase [Vibrio cholerae]	
2	CDS	905818	905165	-	atgaacattgtacaagcgtcgca	MNIVQASQSRYSTKAFDASRK	fig|127906.87.peg.3638	VCCA0920	VCA0637	nfnB	Oxygen-insensitive NAD(P)H nitroreductase (EC 1.-.-.-) / Dihydropteridine reductase (EC 1.5.1.34)		
2	CDS	909192	906034	-	atggaaagtcgactcggtatttcg	MESRLGISGRIAAAFQNSAMT	fig|127906.87.peg.3639	VCCA0921	VCA0638	mdtC	RND efflux system, inner membrane transporter		
2	CDS	910198	909194	-	atgaaagcgttaagttggagtat	MKALSWSIALVLASSAYAQNQ	fig|127906.87.peg.3640	VCCA0922	VCA0639	mdtE	Efflux transporter, RND family, MFP subunit, AcrA/E family		
2	CDS	910766	910308	-	atgaataagctgttgtttcgagca	MNKLLFRATSLVAGLLFGLGM	fig|127906.87.peg.3641	VCCA0923	VCA0640		Predicted transporter component	Select seq ref|WP_001882493.1| YeeE/YedE family protein [Vibrio cholerae]	
2	CDS	911196	910777	-	atgacgttttctattccttgggattc	MTFSIPWDSLIGGMLLGISATL	fig|127906.87.peg.3642	VCCA0924	VCA0641		Putative transmembrane protein	YeeE/YedE family protein [Vibrio cholerae]	
2	CDS	911530	911213	-	atgccagaacccaccaccatca	MPEPTTIKIEMDEMKKNAEEV	fig|127906.87.peg.3643	VCCA0925	VCA0642		Transcriptional regulator, ArsR family	Select seq ref|WP_138042654.1| helix-turn-helix transcriptional regulator [Vibrio cholerae]	
2	CDS	911685	911870	+	atgacgattgaaaacggtgtaa	MTIENGVRVLAGSMVLLSVILT	fig|127906.87.peg.3644	VCCA0926	VCA0643		Uncharacterized membrane protein RSP_0589	Select seq gb|EEO14531.1| rhodanese-related sulfurtransferase [Vibrio cholerae TMA 21]	
2	CDS	911936	913639	+	atgacgaaaatagtgattattgg	MTKIVIIGGVAGGASAAARARF	fig|127906.87.peg.3645	VCCA0927	VCA0644	cdr	CoA-disulfide reductase (EC 1.8.1.14)		
2	CDS	914112	913732	-	atgctcaataatcccgtaggttgg	MLNNPVGWFEIYVNDMERAK	fig|127906.87.peg.3646	VCCA0928	VCA0645		Glyoxalase family protein		
2	CDS	915973	914228	-	atgatgcaaacccagccatttcg	MMQTQPFRLPRFTPFGVIEKG	fig|127906.87.peg.3647	VCCA0929	VCA0646		Putative hemolysin	Select seq ref|WP_000983833.1| GNAT family N-acetyltransferase [Vibrio cholerae]	
2	CDS	916416	915970	-	atgaaatattggtttggtttaatcc	MKYWFGLILFFFSTFTMAQDP	fig|127906.87.peg.3648	VCCA0930	VCA0647		hypothetical protein	YHS domain-containing protein [Vibrio cholerae]	
2	CDS	917173	916427	-	atgtgctccgtcacttggtctttga	MCSVTWSLSDTGYQVFFNRD	fig|127906.87.peg.3649	VCCA0931	VCA0648		hypothetical protein		
2	CDS	917715	917173	-	atgcttgctgtgacaaccttagcg	MLAVTTLAQPYRYSAAIHGCLI	fig|127906.87.peg.3650	VCCA0932	VCA0649		hypothetical protein		
2	CDS	918940	917804	-	atgacagttctttctcccgtggca	MTVLSPVACIAEHKVNAGMPL	fig|127906.87.peg.3651	VCCA0933	VCA0650		D-alanine-D-alanine ligase		
2	CDS	919518	918937	-	atggaagccgcttttagcgaatg	MEAAFSEWLTLHSSSPFWVFL	fig|127906.87.peg.3652	VCCA0934	VCA0651		hypothetical protein	Select seq ref|WP_000386919.1| DedA family protein [Vibrio cholerae]	
2	CDS	919790	920227	+	atgtctgagacaaaccaaacc	MSETNQTETPKVDLESISPELF	fig|127906.87.peg.3653	VCCA0935	VCA0652		hypothetical protein	DUF3069 domain-containing protein [Vibrio cholerae]	
2	CDS	921832	920393	-	atggactatccagtcatagcaaa	MDYPVIAKQLLESLGGKSNIQA	fig|127906.87.peg.3654	VCCA0936	VCA0653	sacX/scrA	PTS system, sucrose-specific IIB component (EC 2.7.1.211) / PTS system, sucrose-specific IIC component		
2	CDS	922133	923125	+	atggccagtttgcatgatgtcgcc	MASLHDVARLAGVSKSTVSRV	fig|127906.87.peg.3655	VCCA0937	VCA0654	ccpA	Sucrose operon repressor ScrR, LacI family		
2	CDS	923201	924847	+	atgttattagatacgctgctcgaa	MLLDTLLELAGGINNVTRILAP	fig|127906.87.peg.3656	VCCA0938	VCA0655	scrB/cscA	Sucrose-6-phosphate hydrolase (EC 3.2.1.26)		
2	CDS	925889	924969	-	atgtcaagagtatggctgacagc	MSRVWLTGDAVVDLIPDGQQ	fig|127906.87.peg.3657	VCCA0939	VCA0656	kdgK/cscK	Fructokinase (EC 2.7.1.4)		
2	CDS	926287	927825	+	atgaacgctcgctcaacacgttt	MNARSTRFTQDEVLDLIIVGG	fig|127906.87.peg.3658	VCCA0940	VCA0657	glpD	Aerobic glycerol-3-phosphate dehydrogenase (EC 1.1.5.3)		
2	CDS	929537	927927	-	gtgtatacatttgcgctcataaaa	MYTFALIKITLEQQAEMSAYTP	fig|127906.87.peg.3659	VCCA0941	VCA0658		Methyl-accepting chemotaxis sensor/transducer protein		

2	CDS	930337	929723	-	atgaaattctgggtgttgtgcgtg	MKFWVLCVTFLLTGCGSLVTD	fig|127906.87.peg.3660	VCCA0942	VCA0659		Protein F-related protein		
2	CDS	930469	930347	-	atgaatggcgaacattctaacca	MNGEHSNHGLCDGKQPICKLI	fig|127906.87.peg.3661	VCCA0943	Not annotated as CDS		hypothetical protein		
2	CDS	930559	931032	+	atgtcgaaaattattatccctttag	MSKIIIPLVVFLLAGFMIYRTWT	fig|127906.87.peg.3662	VCCA0944	VCA0661	fklB	Peptidylprolyl isomerase, FKBP-type (EC 5.2.1.8) / Macrophage infectivity potentiator		
2	CDS	931718	931113	-	gtgactgttgtcaactttagccttta	MTVVNFSLYEGFIFDMDGTLLI	fig|127906.87.peg.3663	VCCA0945	VCA0662	yqaB	Fructose-1-phosphate phosphatase YqaB (EC 3.1.3.-)		
2	CDS	931898	933661	+	atgaatattcgccataaactttata	MNIRHKLYTLGIIAILGSIAIFFT	fig|127906.87.peg.3664	VCCA0946	VCA0663		Methyl-accepting chemotaxis sensor/transducer protein		
2	CDS	934067	935434	+	atgcttgagcttctgattggcttag	MLELLIGLVVTFAVGYFIVKGY	fig|127906.87.peg.3665	VCCA0947	VCA0665	dcuD	Anaerobic C4-dicarboxylate transporter DcuC		
2	CDS	936902	935532	-	atgctctcaatctttgatatttacaa	MLSIFDIYKIGVGPSSSHTNGP	fig|127906.87.peg.3666	VCCA0948	VCA0666		L-serine dehydratase, beta subunit (EC 4.3.1.17) / L-serine dehydratase, alpha subunit (EC 4.3.1.17)		
2	CDS	938454	936913	-	atggttacgtattcaacttttattgt	MVTYSTFIVIAIYLILTILISWFVN	fig|127906.87.peg.3667	VCCA0949	VCA0667	sglT	Sodium/solute symporter		
2	CDS	938793	940088	+	atgaccaccaaaaaacacatg	MTTKKHMSEVPMIQRVAAYLA	fig|127906.87.peg.3668	VCCA0950	VCA0669	sauU	4-hydroxybenzoate transporter		
2	CDS	940185	941201	+	atgacaaaatatcaacaagcaa	MTKYQQAKRIVREYFDAIENA	fig|127906.87.peg.3669	VCCA0951	VCA0671		hypothetical protein	SnoaL-like domain-containing protein [Vibrio cholerae]	
2	CDS	941301	941411	+	atggatatctcagagctgtttatttt	MDISELFILGITQNNERITVYRS	fig|127906.87.peg.3670	VCCA0952	Not annotated as CDS		hypothetical protein		
2	CDS	941441	942472	+	atgactgataaatcacgagttat	MTDKSRVMHSEKATVTSKDV	fig|127906.87.peg.3671	VCCA0953	VCA0673	exuR	Transcriptional regulator, LacI family	Select seq ref|WP_000130347.1| LacI family DNA-binding transcriptional regulator [Vibrio cholerae]	
2	CDS	943149	942523	-	atgagttgtagagtccttttagtgg	MSCRVLLVDDHPLMRRGINQL	fig|127906.87.peg.3672	VCCA0954	VCA0674	vraR/narP	Nitrate/nitrite response regulator protein NarP		1 ORF split into 2 ORFs in N16961. None refseq annotated in PATRIC. Only 1 ORF annotated as pseudogene named VCA0674 in KEGG
									VCA0674				1 ORF split into 2 ORFs in N16961. None refseq annotated in PATRIC. Only 1 ORF annotated as pseudogene named VCA0674 in KEGG
2	CDS	944827	943139	-	atggcgaaagccatgttattgatt	MAKAMLLILFLSALTTGVAIVTL	fig|127906.87.peg.3673	VCCA0955	VCA0675	narX/narQ	Nitrate/nitrite sensor protein NarQ		
2	CDS	945060	945554	+	atggtcgacttaagtaaacgacg	MVDLSKRRWFTPNRQPNQSQ	fig|127906.87.peg.3674	VCCA0956	VCA0676	napF	Ferredoxin-type protein NapF (periplasmic nitrate reductase)		
2	CDS	945581	945901	+	atgtcacaaccctcagcttcactc	MSQPSASLHEVHISSLVVHTLF	fig|127906.87.peg.3675	VCCA0957	VCA0677	napD	Periplasmic nitrate reductase component NapD		
2	CDS	945898	948387	+	atgaaaatgaccagacgtgcgt	MKMTRRAFVKANAAASAAAV	fig|127906.87.peg.3676	VCCA0958	VCA0678	napA	Periplasmic nitrate reductase (EC 1.7.99.4)		
2	CDS	948454	948909	+	atgaaaaaacaactcattgcctt	MKKQLIALLSVFTVLAGIAHAE	fig|127906.87.peg.3677	VCCA0959	VCA0679	napB	Nitrate reductase cytochrome c550-type subunit		
2	CDS	948927	949502	+	atgaaatttctaaaagcgttttgg	MKFLKAFWLRLSSPSKAAVGV	fig|127906.87.peg.3678	VCCA0960	VCA0680	napC	Cytochrome c-type protein NapC		
2	CDS	949514	949642	+	atgagtactcttgaatcgattttttg	MSTLESIFWHVLGYSAMPVIIL	fig|127906.87.peg.3679	VCCA0961	No refSeq annotation		hypothetical protein	Select seq ref|WP_000105478.1| TIGR02808 family protein [Vibrio sp. RC586]	
2	CDS	950954	949686	-	atgaaatcggtaaatatcgaatg	MKSVNIEWNVNLRQAFFCIAR	fig|127906.87.peg.3680	VCCA0962	VCA0681		Putative metal-dependent phosphohydrolase with tandem HD motifs	HD-GYP domain-containing protein [Vibrio cholerae]	
2	CDS	951140	951766	+	atgattaccatcgcgctggtgga	MITIALVDDHTIVRSGFAQLLNI	fig|127906.87.peg.3681	VCCA0963	VCA0682	uhpA	Two-component transcriptional response regulator KOX_12280, LuxR family		
2	CDS	951769	953352	+	atggccaagctgtttccactgatt	MAKLFPLIFAFVLYSISQFSLW	fig|127906.87.peg.3682	VCCA0964	VCA0683	comP/uhpB	Two-component system sensor histidine kinase KOX_12275		
2	CDS	953420	954730	+	atgaagattgtacctatggtgcaa	MKIVPMVQPIQAMEIDQAYRH	fig|127906.87.peg.3683	VCCA0965	VCA0684	uhpC	Possible sugar-phosphate sensor or MFS family transporter KOX_12270		
2	CDS	954843	955877	+	atgattgttaatgcaaaacgcac	MIVNAKRTLLALSVLGSMMTA	fig|127906.87.peg.3684	VCCA0966	VCA0685		ABC transporter, substrate-binding protein (cluster 1, maltose/g3p/polyamine/iron)		
2	CDS	955987	958083	+	atgcatgagccttctcaagcgct	MHEPSQALKLTLHQPVKTDPV	fig|127906.87.peg.3685	VCCA0967	VCA0686		ABC transporter, permease protein 1 (cluster 1, maltose/g3p/polyamine/iron) / ABC transporter, permease protein 2 (cluster 1, maltose/g3p/polyamine/iron)		
2	CDS	958095	959150	+	gtggaaaaacaaaactttgttgt	MEKQNFVVLKNICKRFGSNTV	fig|127906.87.peg.3686	VCCA0968	VCA0687	fbpC	ABC transporter, ATP-binding protein (cluster 1, maltose/g3p/polyamine/iron); ABC transporter, ATP-binding protein (cluster 10, nitrate/sulfonate/bicarbonate)		
2	CDS	961011	959236	-	atgttccaacatgcctttacggac	MFQHAFTDYLVQLQQVNQRW	fig|127906.87.peg.3687	VCCA0969	VCA0688	phbC	Polyhydroxyalkanoic acid synthase		
2	CDS	961416	961069	-	atgtacacggaatttttaaaaca	MYTEFFKTFSDQTEKNFEPYIK	fig|127906.87.peg.3688	VCCA0970	VCA0689		hypothetical protein	Select seq ref|WP_148508126.1| phasin family protein [Vibrio cholerae]	
2	CDS	962651	961446	-	atggaaaagatctatatcgtggg	MEKIYIVGAKRSAIGAFGGSLK	fig|127906.87.peg.3689	VCCA0971	VCA0690	thlA	3-ketoacyl-CoA thiolase (EC 2.3.1.16) @ Acetyl-CoA acetyltransferase (EC 2.3.1.9)		
2	CDS	963404	962664	-	atgagaaaaatcgctttgatcac	MRKIALITGSKGGIGSAISTQLV	fig|127906.87.peg.3690	VCCA0972	VCA0691		Acetoacetyl-CoA reductase (EC 1.1.1.36)		
									VCA0692				1 ORF split into 2 ORFs in N16961. None refseq annotated in PATRIC. Only 1 ORF annotated as pseudogene named VCA0692 in KEGG
2	CDS	964977	964075	-	atggttgactatttaaaatctcgaa	MVDYLKSRIRPIRYITGIISVLLM	fig|127906.87.peg.3691	VCCA0973	VCA0692	secF	Protein translocase subunit SecF		1 ORF split into 2 ORFs in N16961. None refseq annotated in PATRIC. Only 1 ORF annotated as pseudogene named VCA0692 in KEGG
2	CDS	966819	964981	-	atgaagaaaaaaacacaaaat	MKKKTQNQAAKRLINHYSAWI	fig|127906.87.peg.3692	VCCA0974	VCA0693	secD	Protein translocase subunit SecD		
2	CDS	967346	966891	-	atgtacttgcgtgttctgatccagt	MYLRVLIQCWFALLAVVLPSQ	fig|127906.87.peg.3693	VCCA0975	VCA0694		hypothetical protein		
2	CDS	968094	967423	-	atgaaaaaaatactcatgctact	MKKILMLLVLILGLGGAGAGFY	fig|127906.87.peg.3694	VCCA0976	VCA0695		SH3 domain protein		
2	CDS	968497	970380	+	atgtacacctcagcccgtaaata	MYTSARKYFIQFAIVAFVLGFIF	fig|127906.87.peg.3695	VCCA0977	VCA0697		Sensory box/GGDEF family protein		
2	CDS	970683	971906	+	atgcaggacactttagctgtgatt	MQDTLAVILAGGMGSRLSPLT	fig|127906.87.peg.3696	VCCA0978	VCA0699	glgC	Glucose-1-phosphate adenylyltransferase (EC 2.7.7.27)		
2	repeat	971925	972055	+	tctatacccaaacaacttggagttgcaggtaggcggcaagtgagtgaatccc		fig|127906.87.repeat.186				repeat region		
2	CDS	975327	972172	-	atgcgtgtactcgaaggaaaag	MRVLEGKAGKSVFTLSTLTAS	fig|127906.87.peg.3697	VCCA0979	VCA0700		Chitodextrinase (EC 3.2.1.14)		
2	CDS	975492	975361	-	ttgcataaatcagacactcgtttg	MHKSDTRLRVIIKIHTFLSAKLS	fig|127906.87.peg.3698	VCCA0980	Not annotated as CDS		FIG01200424: hypothetical protein	Select seq emb|CSI39093.1| ClC family chloride transporter [Vibrio cholerae]	
2	repeat	975700	975827	+	atacccaaacaacttggagttacaggtaggcagcaagtgagtgactcccca		fig|127906.87.repeat.187				repeat region		
2	CDS	975999	977147	+	atgcagtttagttacgtcaaccca	MQFSYVNPTMIHFGQGQIASII	fig|127906.87.peg.3699	VCCA0981	VCA0702	yqhD	Iron-containing alcohol dehydrogenase		
2	CDS	977226	977915	+	gtgctgcatttttgagtttcgtttatc	MLHFLSFVYGRFADVIADVIAD	fig|127906.87.peg.3700	VCCA0982	VCA0703		putative membrane protein	lysoplasmalogenase [Vibrio cholerae]	Limited subject cover for certain strains
2	CDS	979174	977912	-	atgaaaagtaccgtactgctgat	MKSTVLLIDDDQDVLDSYLHLI	fig|127906.87.peg.3701	VCCA0983	VCA0704	pgtA	Phosphoglycerate transport system transcriptional regulatory protein PgtA		
2	CDS	981518	979149	-	atgagtttgaccactcgccacac	MSLTTRHTIGARLLLAFAFSTS	fig|127906.87.peg.3702	VCCA0984	VCA0705	pgtB	Phosphoglycerate transport system sensor protein PgtB		
2	CDS	982807	981515	-	atgccgcgatggatttttgccttta	MPRWIFAFIMPCLLLSLGVSTC	fig|127906.87.peg.3703	VCCA0985	VCA0706	pgtC	Phosphoglycerate transport regulatory protein PgtC		
2	CDS	983084	984463	+	atgcttaatttctttaaaacccgcc	MLNFFKTRPDLPLLSSSKAEM	fig|127906.87.peg.3704	VCCA0986	VCA0707	pgtP	Phosphoglycerate transporter protein PgtP		
2	CDS	984585	986045	+	atgtgtaaaaccaaaattgtggc	MCKTKIVATLGPASQTRETLTC	fig|127906.87.peg.3705	VCCA0987	VCA0708		Pyruvate kinase (EC 2.7.1.40)		
2	CDS	989118	986164	-	atgcgtctcgccaaagccagtat	MRLAKASIGRKLLFSFSAMALL	fig|127906.87.peg.3706	VCCA0988	VCA0709	torS	Trimethylamine-N-oxide sensor histidine kinase TorS (EC 2.7.13.3)		
2	CDS	989206	990198	+	atgccaaatctgcgtttgcactca	MPNLRLHSFSLISALLLLSALPA	fig|127906.87.peg.3707	VCCA0989	VCA0710	torT	Periplasmic protein TorT		
2	CDS	990253	990708	+	atgaaaaaaaccacccgcact	MKKTTRTMAAHKHVALVAHDI	fig|127906.87.peg.3708	VCCA0990	VCA0711	mgsA	Methylglyoxal synthase (EC 4.2.3.3)		
2	CDS	991397	990777	-	atgagcaaagcacttctgttagtt	MSKALLLVDVQNDFSPSGALF	fig|127906.87.peg.3709	VCCA0991	VCA0712	pncA	Nicotinamidase (EC 3.5.1.19)		
2	CDS	991635	991519	-	gtgttaatcggcgttaagatagcg	MLIGVKIAMKSLICAQAQVAAP	fig|127906.87.peg.3710	VCCA0992	VCA0713		hypothetical protein		
2	CDS	991702	992058	+	atgaaacactctcgtactggcttc	MKHSRTGFSRIIYATGYSMKG	fig|127906.87.peg.3711	VCCA0993	VCA0714	dgkA	Diacylglycerol kinase (EC 2.7.1.107)		Limited query cover for certain strains
2	CDS	992207	992515	+	atgcataacgtaaaggagcgca	MHNVKERIMSLLKISRLFAITSL	fig|127906.87.peg.3712	VCCA0994	VCA0715		hypothetical protein	cytochrome b [Vibrio cholerae]	
2	CDS	992508	993044	+	atggctaaaccatacacatggg	MAKPYTWDLFVRVTHWLVAA	fig|127906.87.peg.3713	VCCA0995	VCA0716		Cytochrome b (EC 1.10.2.2)		
2	CDS	995199	993100	-	atgattgccgacaactttgttagtc	MIADNFVSGLLPMQLNANKTA	fig|127906.87.peg.3714	VCCA0996	VCA0717	helD	DNA helicase IV (EC 3.6.4.12)		
2	CDS	995350	997527	+	atgtgctgcgctgtgccgaaaat	MCCAVPKINQLRLYWANKTVN	fig|127906.87.peg.3715	VCCA0997	VCA0718	yccS	Membrane protein	TIGR01666 family membrane protein [Vibrio cholerae]	
2	CDS	998484	997537	-	atgagtgccgaagcgcaatatc	MSAEAQYQRIYERERKARILAI	fig|127906.87.peg.3716	VCCA0998	VCA0719		Two-component system sensor histidine kinase	Select seq ref|WP_001271642.1| two-component sensor histidine kinase [Vibrio cholerae]	
2	CDS	999026	998481	-	atgcaaggcattatctataccgtt	MQGIIYTVLSDMVIEKFGVLFW	fig|127906.87.peg.3717	VCCA0999	VCA0720		guanylate cyclase-related protein		
2	CDS	999271	999495	+	atgagcaccgattgtcgaagac	MSTDCRRRLNKDLYLLIPEHPI	fig|127906.87.peg.3718	VCCA1000	VCA0721		hypothetical protein		
2	CDS	999552	999842	+	atgtccgtatcagcagtttctaatg	MSVSAVSNGSSMVMHSSKMN	fig|127906.87.peg.3719	VCCA1001	VCA0722		hypothetical protein		Limited query cover for certain strains
2	CDS	999929	1001182	+	atgcctaagcttgctttgcaacct	MPKLALQPLYSPDVATLAPDR	fig|127906.87.peg.3720	VCCA1002	VCA0723	mvaA	Hydroxymethylglutaryl-CoA reductase (EC 1.1.1.34)		
2	CDS	1002381	1001281	-	atgagtgaagtgattctgcaactg	MSEVILQLQKRLGETQLDVDL	fig|127906.87.peg.3721	VCCA1003	VCA0724	modC	Molybdenum ABC transporter ATP-binding protein ModC		
2	CDS	1003090	1002395	-	atgggtctctccgattacgaatgg	MGLSDYEWAALALSLKVAGYA	fig|127906.87.peg.3722	VCCA1004	VCA0725	modB	Molybdenum ABC transporter permease protein ModB		
2	CDS	1003852	1003094	-	atgttgacacgaattctgctcctg	MLTRILLLLCVALNAPLASAKE	fig|127906.87.peg.3723	VCCA1005	VCA0726	modA	Molybdenum ABC transporter, substrate-binding protein ModA		
2	CDS	1005318	1003864	-	atgaaaaaatggttaatggtgca	MKKWLMVQGTTSDAGKSVLV	fig|127906.87.peg.3724	VCCA1006	VCA0727	cobQ	Cobyric acid synthase (EC 6.3.5.10)		
2	CDS	1008092	1005921	-	ttgggtaacaatatcgatactatt	MGNNIDTIQSIGLENFKGYRAL	fig|127906.87.peg.3725	VCCA1007	VCA0728		hypothetical protein	Select seq ref|WP_000527687.1| AAA family ATPase [Vibrio cholerae]	
2	CDS	1008708	1008085	-	atggaatcattattggagattagt	MESLLEISFTDCSPARRDIYPV	fig|127906.87.peg.3726	VCCA1008	VCA0729		hypothetical protein		
2	CDS	1010540	1008711	-	atgattgcaaggaatgcgtttgat	MIARNAFDYTVDRILRRRCVPY	fig|127906.87.peg.3727	VCCA1009	VCA0730		hypothetical protein		
2	CDS	1011544	1011239	-	atgcaaaatcgatttaaggttctg	MQNRFKVLLGVILLFPMFAFAK	fig|127906.87.peg.3728	VCCA1010	VCA0731		hypothetical protein		Limited query cover for certain strains
2	CDS	1011747	1012145	+	atgaaacgtactattatcgctctg	MKRTIIALTSALILAPTLVIAADN	fig|127906.87.peg.3729	VCCA1011	VCA0732		hypothetical protein	Select seq ref|WP_119773018.1| YgiW/YdeI family stress tolerance OB fold protein [Vibrio cholerae]	
2	CDS	1012186	1012338	+	atgttttcggacatcgctgtactttt	MFSDIAVLFSPWFTYPISLRFL	fig|127906.87.peg.3730	VCCA1012	VCA0733		hypothetical protein		
2	CDS	1012397	1012915	+	atgctgaaaaaaacgcacctga	MLKKTHLSLLLGLTLATPALAN	fig|127906.87.peg.3731	VCCA1013	VCA0734		hypothetical protein		Limited query/subject cover for certain strains
2	CDS	1013159	1013824	+	atggctgtcggttctgaagatctg	MAVGSEDLNKLALLLKPGMKV	fig|127906.87.peg.3732	VCCA1014	VCA0735		hypothetical protein	Select seq ref|WP_000268204.1| flagellar brake protein [Vibrio cholerae]	
2	CDS	1016389	1013816	-	ttgaacattcgacctagccaaat	MNIRPSQIKHKQRIASFITHAV	fig|127906.87.peg.3733	VCCA1015	VCA0736	luxQ	Autoinducer 2 sensor kinase/phosphatase LuxQ (EC 2.7.3.-) (EC 3.1.3.-)		
2	CDS	1017501	1016407	-	atgaaaagaaagctaatatctc	MKRKLISPLFLIAALFSSTGQA	fig|127906.87.peg.3734	VCCA1016	VCA0737	luxP	Autoinducer 2-binding periplasmic protein LuxP precursor		
2	CDS	1018873	1017713	-	atgaatattcaaacaaaaagta	MNIQTKSTLAVVMAMAFSSSA	fig|127906.87.peg.3735	VCCA1017	VCA0738		hypothetical protein	Select seq ref|WP_001025860.1| conjugal transfer protein TraF [Vibrio cholerae]	
2	CDS	1019280	1018975	-	atggaaaacttggctgagttacc	MENLAELPSPWFVYLVRCANI	fig|127906.87.peg.3736	VCCA1018	VCA0739		UPF0213 protein YhbQ	GIY-YIG nuclease family protein [Vibrio cholerae]	
2	CDS	1019947	1019297	-	atgaatattcaactctccccactc	MNIQLSPLEARVIGCLIEKEVT	fig|127906.87.peg.3737	VCCA1019	VCA0740	yceH	Protein of unknown function YceH	YceH family protein [Vibrio cholerae]	
2	CDS	1020186	1019989	-	atgcgtaattctgagattttggttc	MRNSEILVPTPPLQTELDAVAI	fig|127906.87.peg.3738	VCCA1020	No refSeq annotation		FIG01200784: hypothetical protein		1 ORF split into 2 ORFs in N16961. None refseq annotated in PATRIC or KEGG
2	CDS	1020295	1020179	-	atgcggcttatcttgaccaataaa	MRLILTNKSPFKARANCTSEIM	fig|127906.87.peg.3739	VCCA1021	No refSeq annotation		hypothetical protein		
2	CDS	1020374	1020694	+	atgaacagcgtatttgaaattgtg	MNSVFEIVSLARRKNKLQRELI	fig|127906.87.peg.3740	VCCA1022	VCA0741		UPF0265 protein YeeX	DUF496 family protein [Vibrio cholerae]	
2	CDS	1020694	1020843	+	atgattccagacccaccgcaag	MIPDPPQGGFYLARQSVSLIRI	fig|127906.87.peg.3741	VCCA1023	VCA0742		hypothetical protein		
2	CDS	1021107	1021358	+	atggatatgagtatggttgaaatt	MDMSMVEILGYAASIMVAVSL	fig|127906.87.peg.3742	VCCA1024	VCA0743		hypothetical protein	uroporphyrinogen decarboxylase [Vibrio cholerae]	
2	CDS	1022956	1021439	-	atgactgagcaaaaatacgtcg	MTEQKYVVALDQGTTSSRAV	fig|127906.87.peg.3743	VCCA1025	VCA0744	glpK	Glycerol kinase (EC 2.7.1.30)		
									VCA0745				1 ORF split into 2 ORFs in N16961. None refseq annotated in PATRIC. Only 1 ORF annotated as pseudogene named VCA0745 in KEGG
2	CDS	1023940	1023086	-	atgaaccaaacaaagcaacat	MNQTKQHTLLGQCIAEFIGTG	fig|127906.87.peg.3744	VCCA1026	VCA0745	glpF	Glycerol uptake facilitator protein		1 ORF split into 2 ORFs in N16961. None refseq annotated in PATRIC. Only 1 ORF annotated as pseudogene named VCA0745 in KEGG
2	CDS	1024242	1024108	-	atgcgttgtttaacaaaaaacga	MRCLTKNEFVNNKIVLEHSLIA	fig|127906.87.peg.3745	VCCA1027	Not annotated as CDS		hypothetical protein		
2	CDS	1024436	1024278	-	atgattaacactgttcgtaacatg	MINTVRNMYDTFNVRLSEYVR	fig|127906.87.peg.3746	VCCA1028	VCA0746		FIG01201624: hypothetical protein		
2	CDS	1024595	1026238	+	atgacggcttatcaaagaatgga	MTAYQRMETDVVIIGGGATGT	fig|127906.87.peg.3747	VCCA1029	VCA0747	glpA	Anaerobic glycerol-3-phosphate dehydrogenase subunit A (EC 1.1.5.3)		
2	CDS	1026235	1027545	+	atgatgcattatgatgtggccgtc	MMHYDVAVIGGGIAGYSAALR	fig|127906.87.peg.3748	VCCA1030	VCA0748	glpB	Anaerobic glycerol-3-phosphate dehydrogenase subunit B (EC 1.1.5.3)		
2	CDS	1027542	1028783	+	atgaaccatgcctttattcactctg	MNHAFIHSAPQNTTFDQCIKC	fig|127906.87.peg.3749	VCCA1031	VCA0749	glpC	Anaerobic glycerol-3-phosphate dehydrogenase subunit C (EC 1.1.5.3)		
2	CDS	1030013	1028925	-	atgactcaatgttatgccgaaatc	MTQCYAEITGWGKCLPPATLS	fig|127906.87.peg.3750	VCCA1032	VCA0751		3-oxoacyl-[acyl-carrier-protein] synthase, KASIII (EC 2.3.1.180)		
2	CDS	1030182	1030616	+	atgtcaacattcaatacccgctg	MSTFNTRCPSCHSLNRVPEEF	fig|127906.87.peg.3751	VCCA1033	VCA0752	trxC	Thioredoxin 2		
2	CDS	1030805	1031959	+	gtggaaatccattcttctgcacca	MEIHSSAPNRIAVPVIALTLYAI	fig|127906.87.peg.3752	VCCA1034	VCA0753	ycaD	Uncharacterized MFS-type transporter	MFS transporter [Vibrio cholerae]	
2	CDS	1032086	1032874	+	gtgaaaccactcaaacgctacc	MKPLKRYQYERYAVLCNLAYF	fig|127906.87.peg.3753	VCCA1035	VCA0754		Lipase-related protein		
2	rna	1032980	1032907	-	ggcgcgttggcagagtggccatgcagcggattgcaaatccgtgaacctcgg		fig|127906.87.rna.125	VCCAt002	VCAt002		tRNA-Cys-GCA		
2	rna	1033091	1033005	-	gccctggtggtgaaatcggtagacacaagggatttaaaatccctcgactttcg		fig|127906.87.rna.126	VCCAt003	VCAt003		tRNA-Leu-TAA		
2	CDS	1033809	1033309	-	atgagcacatacccacgttcgttt	MSTYPRSFSHIGISVPDLEAAV	fig|127906.87.peg.3754	VCCA1036	VCA0754a		Glyoxalase family protein		
2	CDS	1033984	1034889	+	atgctcaacccaatctggctaca	MLNPIWLQTFKTLVDTGHFTK	fig|127906.87.peg.3755	VCCA1037	VCA0756		Transcriptional regulator, LysR family	Select seq ref|WP_000933929.1| LysR family transcriptional regulator [Vibrio cholerae]	
2	CDS	1035610	1034942	-	atgaaagagcaacacctatggc	MKEQHLWQLLEGLGTSLQLTA	fig|127906.87.peg.3756	VCCA1038	VCA0757	artM	Arginine ABC transporter, permease protein ArtM		
2	CDS	1036296	1035607	-	atggtgttaacgggctactctttat	MVLTGYSLSLVQASWMTLQIA	fig|127906.87.peg.3757	VCCA1039	VCA0758	artQ	Arginine ABC transporter, permease protein ArtQ		
2	CDS	1037029	1036298	-	atgaaaaagatcctactcgcgtc	MKKILLASLIGLVSASAAAQQE	fig|127906.87.peg.3758	VCCA1040	VCA0759	artI	Arginine ABC transporter, substrate-binding protein ArtI		
2	CDS	1037846	1037103	-	atgcagtatgtccgcatgagtatt	MQYVRMSIRVKGINKSYGTTQ	fig|127906.87.peg.3759	VCCA1041	VCA0760	artP	Arginine ABC transporter, ATP-binding protein ArtP		
2	rna	1038200	1038127	-	ggcgcgttggcagagtggccatgcagcggattgcaaatccgtgaacctcgg		fig|127906.87.rna.127	VCCAt004	VCAt004		tRNA-Cys-GCA		
2	CDS	1039869	1038424	-	atgttaggggcggatgcgtttaat	MLGADAFNLVVAIAESGLIDTA	fig|127906.87.peg.3760	VCCA1042	VCA0762	viaA/yieM	hypothetical protein	Select seq ref|WP_000899954.1| ATPase RavA stimulator ViaA [Vibrio cholerae]	
2	CDS	1041536	1039878	-	atgctgcaacccacccacagct	MLQPTHSSHARKALLSERINK	fig|127906.87.peg.3761	VCCA1043	VCA0763	ravA	ATPase RavA		
2	CDS	1041888	1042403	+	atgagcaaaatcatccatcaatc	MSKIIHQWKSIALVEEDVLLPN	fig|127906.87.peg.3762	VCCA1044	VCA0764		ADP-ribose pyrophosphatase (EC 3.6.1.13)		
2	CDS	1042412	1043419	+	atggactttcgatcggataccgtt	MDFRSDTVTQPTDAMRQAMA	fig|127906.87.peg.3763	VCCA1045	VCA0765	ltaE/ybjU	Low-specificity L-threonine aldolase (EC 4.1.2.48)		
2	CDS	1043548	1043856	+	atgcgcgttgtgtgctctctgctttg	MRVVCSLLCLTLVSTPLFASGI	fig|127906.87.peg.3764	VCCA1046	VCA0766		Cytochrome c553		
2	CDS	1043960	1044694	+	atgattgaaaaaaaacagggg	MIEKKQGRRSAQDAQKTRYH	fig|127906.87.peg.3765	VCCA1047	VCA0767	ttgR	Transcriptional regulator, AcrR family	TetR/AcrR family transcriptional regulator [Vibrio cholerae]	
2	CDS	1046062	1044794	-	atgtcagaactctccatcactttttt	MSELSITFSQLGLDSRLLNTLS	fig|127906.87.peg.3766	VCCA1048	VCA0768		ATP-dependent RNA helicase VCA0768		
2	CDS	1046680	1046099	-	atgaatttccttgcccatttgcata	MNFLAHLHIAHHCQSSLLGNL	fig|127906.87.peg.3767	VCCA1049	VCA0769	acpH	Acyl carrier protein phosphodiesterase (EC 3.1.4.14)		
2	CDS	1046836	1046717	-	atgagtgaccacaatcacaatg	MSDHNHNDECLELLDAMEIGF	fig|127906.87.peg.3768	VCCA1050	VCA0770		hypothetical protein		
2	CDS	1047200	1048402	+	atgacgcaatcgaagcttttagg	MTQSKLLGSTLIIAGTTIGAGM	fig|127906.87.peg.3769	VCCA1051	VCA0772	tyrP	Tyrosine-specific transport protein		
2	CDS	1048630	1050051	+	atgagagaagttcaattcaggac	MREVQFRTIDRVFIRMSINDKM	fig|127906.87.peg.3770	VCCA1052	VCA0773		Methyl-accepting chemotaxis sensor/transducer protein		
2	CDS	1051153	1050137	-	atgaaagtattggttacaggtgg	MKVLVTGGMGYIGSHTCIQMI	fig|127906.87.peg.3771	VCCA1053	VCA0774	galE	UDP-glucose 4-epimerase (EC 5.1.3.2)		
2	CDS	1051345	1051476	+	gtggcatcgcacttaattcagaa	MASHLIQNAPKHAKFIDQTLIS	fig|127906.87.peg.3772	VCCA1054	Not annotated as CDS		FIG01206651: hypothetical protein		
2	CDS	1051473	1052213	+	gtgagcaaacggacaatccaa	MSKRTIQLNCDMGESFGVWT	fig|127906.87.peg.3773	VCCA1055	VCA0776	lamB	Lactam utilization protein LamB		
2	CDS	1052217	1052927	+	atgtccatcacgtttgaaattgag	MSITFEIEPIAEGSILVRFQHPA	fig|127906.87.peg.3774	VCCA1056	VCA0777	kipI	Allophanate hydrolase 2 subunit 1 (EC 3.5.1.54)		
2	CDS	1053037	1053966	+	atgctaggtaagctgcacgtgat	MLGKLHVIKAGPLTLLHDLGRY	fig|127906.87.peg.3775	VCCA1057	VCA0778	kipA	Allophanate hydrolase 2 subunit 2 (EC 3.5.1.54)		
2	CDS	1055451	1054018	-	atgacattgcgatctcaagatcc	MTLRSQDPRIAIIGGGIAGATA	fig|127906.87.peg.3776	VCCA1058	VCA0779		hypothetical protein	FAD-dependent oxidoreductase [Vibrio cholerae]	
2	CDS	1056726	1055560	-	atgaaaattgccattgcaggaac	MKIAIAGTGYVGLSNGILLAQH	fig|127906.87.peg.3777	VCCA1059	VCA0780	ugd	UDP-glucose 6-dehydrogenase (EC 1.1.1.22)		
2	CDS	1059562	1057115	-	atgaaggccttgcctcccgcatc	MKALPPASLNRRLLRWSLSEI	fig|127906.87.peg.3778	VCCA1060	VCA0781		ABC transporter, fused permease protein		
2	CDS	1060236	1059559	-	atgcaaacatccatcatttcagc	MQTSIISAQSLSKLVSTNQEHL	fig|127906.87.peg.3779	VCCA1061	VCA0782		ABC-type antimicrobial peptide transport system, ATPase component		
2	CDS	1060235	1060834	+	atgactcgactactttccttttttgttt	MTRLLSFLFVVLFSAAASSKTL	fig|127906.87.peg.3780	VCCA1062	VCA0783		Arylesterase precursor (EC 3.1.1.2)		
2	CDS	1060924	1062132	+	atgcacatcaagccaatcattca	MHIKPIIQGVVARSAHPYGCE	fig|127906.87.peg.3781	VCCA1063	VCA0784		Enoyl-[acyl-carrier-protein] reductase [NADH] (EC 1.3.1.9), FabV => refractory to triclosan		
2	CDS	1062301	1064184	+	atggcaccgatcctttcacactcg	MAPILSHSIPIPSSMQANWQQ	fig|127906.87.peg.3782	VCCA1064	VCA0785		GGDEF domain protein		
2	CDS	1064310	1065959	+	atggatcgactaattgcaacact	MDRLIATLKKLEKQNYRAYQQ	fig|127906.87.peg.3783	VCCA1065	VCA0786		Isopentenyl-diphosphate Delta-isomerase (EC 5.3.3.2)		
					atgcagccctttgtcagttgcatgacctttgaaacctgttttctagggatttcttcca		Only in N16961	Not annotated as CDS	VCA0787		Hypothetical protein		
2	CDS	1066746	1066117	-	atgtcagacagtcaccagcttgc	MSDSHQLAATFQTYMENPLLV	fig|127906.87.peg.3784	VCCA1066	VCA0788		DnaJ-related protein		
2	CDS	1066982	1067569	+	atgcccttgtcgcgacctctcatc	MPLSRPLIGLTLFYSLIFVFSAI	fig|127906.87.peg.3785	VCCA1067	VCA0789		Predicted membrane protein	DUF2238 domain-containing protein [Vibrio cholerae]	
2	CDS	1067743	1069122	+	atgacacatcaagaaaacctct	MTHQENLSNPQEVNKKVTKR	fig|127906.87.peg.3786	VCCA1068	VCA0790		possible integrase		
2	repeat	1069106	1070364	+	ttgtagtggtcaactaaaattggccacggttttagagttttcccaatataatcgttc		fig|127906.87.repeat.188				repeat region		
2	CDS	1069987	1069115	-	ttgataatcaagaaactcaagca	MIIKKLKQSHSVKILCEVFNVHI	fig|127906.87.peg.3787	VCCA1069	VCA0791		Transposase InsO for insertion sequence element IS911		
2	CDS	1070292	1069984	-	atgacaaaacgtacaagacga	MTKRTRRLFSAEFKLEAAQLV	fig|127906.87.peg.3788	VCCA1070	VCA0792		Transposase InsN for insertion sequence element IS911		

Table S3. Variant analysis between V. cholerae C6706 and N16961

Chr	Pos	Ref	Var	Score	Var_cov	Var_frac	Type	Ref_nt	Var_nt	Ref_nt_pos_change	Ref_aa_pos_change	Frameshift	Gene_ID	Locus_tag_C6706	Locus_tag_N16961	Gene_name	Function
1	27804	C	T	26321	769	1.00	Nonsyn	cgt	cAt	74G>A	Arg25His		fig|243277.254.peg.28	VCC0019	VC0030	*ilvM*	Acetolactate synthase small subunit (EC 2.2.1.6)
1	34254	C	T	21442	699	0.99	Nonsyn	gcc	Acc	28G>A	Ala10Thr		fig|243277.254.peg.33	VCC0024	VC0035		YihE protein, a ser/thr kinase implicated in LPS synthesis and Cpx signalling
1	137105	TAACAGAAACAGAAACAGAAACAGAAACAGAAACAGAAACAGA	TAACAGAAACAGAAACAGAAACAGAAACAGAAACAGAAACAGA	1904.7	205	0.53	Insertion	gaaacagaaacagaaacagaaacagaaacagaaacagaaacagaaac	AAACAGAAACAGAAACAGAAACAGAAACAGAAACAGAAACAGA	206_211dupAAACAG	Glu69_Thr70dup		fig|243277.254.peg.135	VCC0126	VC0147		Signal recognition particle receptor FtsY
1	185099	G	T	23893	692	1.00	Nonsyn	cca	cAa	1373C>A	Pro458Gln		fig|243277.254.peg.173	VCC2661/2	VC0183		Phage integrase
1	254869	T	C	23398	706	0.99	Synon	act	acC	117T>C	Thr39Thr		fig|243277.254.peg.236	VCC2595	VC0249		Long-chain-fatty-acid--CoA ligase (EC 6.2.1.3)
1	281442	C	T	24344	712	1.00	Synon	gcg	gcA	1038G>A	Ala346Ala		fig|243277.254.peg.262	VCC2572	VC0276	*purH*	IMP cyclohydrolase (EC 3.5.4.10) / Phosphoribosylaminoimidazolecarboxamide formyltransferase (EC 2.1.2.3)
1	286337	C	T	25418	752	0.99	Nonsyn	act	aTt	686C>T	Thr229Ile		fig|243277.254.peg.267	VCC2568	VC0280	*cadB*	Lysine/cadaverine antiporter membrane protein CadB
1	335660	G	C	6391.7	219	0.98	Nonsyn	cga	cCa	335G>C	Arg112Pro		fig|243277.254.peg.304	VCC2532	VC0321	*tuf*	Translation elongation factor Tu
1	347722	T	C	24117	697	1.00	Synon	gat	gaC	3219T>C	Asp1073Asp		fig|243277.254.peg.312	VCC2524	VC0329		DNA-directed RNA polymerase beta' subunit (EC 2.7.7.6)
1	364550	C	A	21612	660	0.99	Nonsyn	ggt	Tgt	136G>T	Gly46Cys		fig|243277.254.peg.325	VCC2511	VC0342		Epoxyqueuosine reductase (EC 1.17.99.6) QueG
1	378633	A	G	22809	655	1.00	Nonsyn	aaa	aGa	128A>G	Lys43Arg		fig|243277.254.peg.342	VCC2494	VC0359	*rpsL*	SSU ribosomal protein S12p (S23e)
1	521838	T	A	20649	607	1.00	Nonsyn	aaa	aTa	557A>T	Lys186Ile		fig|243277.254.peg.469	VCC2365/6	VC0489		Putative hemolysin
1	522136	C	A	19186	591	0.99	Nonsyn	gaa	Taa	259G>T	Glu87*		fig|243277.254.peg.469	VCC2365/6	VC0489		Putative hemolysin
1	558833	G	A	19838	608	0.99	Synon	ccg	ccA	471G>A	Pro157Pro		fig|243277.254.peg.511	VCC2325	VC0525		2-amino-4-hydroxy-6-hydroxymethyldihydropteridine pyrophosphokinase (EC 2.7.6.3)
1	575610	A	G	20473	601	0.99	Nonsyn	tac	tGc	914A>G	Tyr305Cys		fig|243277.254.peg.528	VCC2308	VC0543	*recA*	RecA protein
1	612959	GAC	GAAC	16421	525	0.98	Insertion	gtcgtc	GTTCgtc	217_218insT	Val74fs	yes	fig|243277.254.peg.566	VCC2271	VC0583	*HapR*	Quorum-sensing regulator of virulence HapR
1	656622	G	A	16717	508	0.99	Synon	ggc	ggT	705C>T	Gly235Gly		fig|243277.254.peg.599	VCC2238	VC0620		Chitobiose ABC transporter, substrate-binding protein
1	697749	A	G	17911	518	1.00	Nonsyn	gtg	gCg	1664T>C	Val555Ala		fig|243277.254.peg.633	VCC2204	VC0653		diguanylate cyclase/phosphodiesterase (GGDEF & EAL domains) with PAS/PAC sensor(s)
1	720770	C	T	18709	545	1.00	Nonsyn	ccc	Tcc	25C>T	Pro9Ser		fig|243277.254.peg.656	VCC2182	VC0674		Prolipoprotein diacylglyceryl transferase
1	743698	C	A	16487	494	1.00	Synon	cgc	cgA	162C>A	Arg54Arg		fig|243277.254.peg.676	VCC2162	VC0695		2-keto-3-deoxy-D-arabino-heptulosonate-7-phosphate synthase I alpha (EC 2.5.1.54)
1	775461	T	G	17756	523	1.00	Nonsyn	acc	Ccc	1366A>C	Thr456Pro		fig|243277.254.peg.700	VCC2138	VC0722		Exopolyphosphatase (EC 3.6.1.11)
1	805327	C	T	18111	528	1.00	Synon	gac	gaT	1518C>T	Asp506Asp		fig|243277.254.peg.725	VCC2113	VC0752	*hscA*	Chaperone protein HscA
1	881248	CTTAA	CTTTAA	6244.7	208	0.98	Insertion	ggcttaatg	ggCTTTAAtg	1118dupT	Leu373fs	yes	fig|243277.254.peg.790	VCC2048	VC0821/2		hypothetical protein
1	899563	A	G	16593	484	1.00	Nonsyn	atg	Gtg	886A>G	Met296Val		fig|243277.254.peg.806	VCC2033	VC0837		Toxin co-regulated pilus biosynthesis protein F, putative outer membrane channel for TcpA extrusion
1	1091211	G	A	12062	357	1.00	Nonsyn	ggc	Agc	997G>A	Gly333Ser		fig|243277.254.peg.981	VCC1859	VC1021	*luxO*	Regulatory protein LuxO
1	1112676	TCGGCGATAACG	TG	9372.4	343	1.00	Deletion	gccgttatcgccgat	gcCAt	139_148delGTTATCGCCG	Val47fs	yes	fig|243277.254.peg.1001	VCC1838	VC1043		Long-chain fatty acid transport protein
1	1116888	A	G	13318	391	0.99	Nonsyn	gac	gGc	680A>G	Asp227Gly		fig|243277.254.peg.1005	VCC1834	VC1047	*fadJ*	Enoyl-CoA hydratase (EC 4.2.1.17) / Delta(3)-cis-delta(2)-trans-enoyl-CoA isomerase (EC 5.3.3.8) / 3-hydroxyacyl-CoA dehydrogenase (EC 1.1.1.35) / 3-hydroxybutyryl-CoA epimerase (EC 5.1.2.3)
1	1216033	G	T	12756	368	1.00	Synon	atc	atA	1020C>A	Ile340Ile		fig|243277.254.peg.1101	VCC1738	VC1145		Putative transport protein YbjL
1	1262261	C	T	11482	357	0.99	Synon	gcg	gcA	1167G>A	Ala389Ala		fig|243277.254.peg.1142	VCC1697	VC1188		NAD-dependent malic enzyme (EC 1.1.1.38)
1	1373399	GAAGG	GAGG	8937	291	1.00	Deletion	tccttc	tCCTC	1153delT	Phe385fs	yes	fig|243277.254.peg.1241	VCC1597/8	VC1295		Response regulator
1	1402970	T	G	9846.4	287	1.00	Nonsyn	tta	ttC	687A>C	Leu229Phe		fig|243277.254.peg.1265	VCC1573	VC1319	*rstB*	Two-component system sensor histidine kinase
1	1466928	A	T	10718	312	0.99	Synon	cct	ccA	1344T>A	Pro448Pro		fig|243277.254.peg.1315	VCC1521	VC1374		DnaK-related protein
1	1571665	C	T	397.17	19	1.00	Synon	gtg	gtA	105G>A	Val35Val		fig|243277.254.peg.1401	VCC1435	VC1462		RstB phage-related integrase
1	1591828	A	G	9153.6	269	1.00	Synon	tat	taC	1170T>C	Tyr390Tyr		fig|243277.254.peg.1425	VCC1405	VC1486	*uup*	Bis-ABC ATPase Uup
1	1728150	T	G	8073.6	243	0.99	Nonsyn	aga	agC	915A>C	Arg305Ser		fig|243277.254.peg.1548	VCC1222	VC1614		23S rRNA (adenine(1618)-N(6))-methyltransferase (EC 2.1.1.181)
1	1778527	CGATAATCCAGATAATCCAGATAATCCAGATAATCCAGATAATCC	CGATAATCCAGATAATCCAGATAATCCAGATAATCCAGATAATCC	2655.1	97	0.95	Deletion	aatccagataatccagataatccagataatccagataatccagataat	TCCAGATAATCCAGATAATCCAGATAATCCAGATAATCCAGATAA	1749_1757delTAATCCAGA	Asp583_Pro585del		fig|243277.254.peg.1583	VCC1188	VC1650	*prt/vcc*	Microbial collagenase, secreted (EC 3.4.24.3)
1	1784614	G	A	10346	310	1.00	Nonsyn	cac	Tac	1675C>T	His559Tyr		fig|243277.254.peg.1586	VCC1185	VC1653	*vieS*	Sensory box sensor histidine kinase/response regulator VieS
1	1921705	A	G	11919	354	0.99	Nonsyn	tcg	Ccg	130T>C	Ser44Pro		fig|243277.254.peg.1696	VCC1073	VC1771		hypothetical protein
1	1958051	C	T	10954	334	0.99	Synon	ggg	ggA	933G>A	Gly311Gly		fig|243277.254.peg.1743	VCC1026	VC1817	*acoR*	sigma-54-dependent transcriptional regulator
1	1989285	G	A	10945	326	0.99	Nonsyn	gtg	Atg	463G>A	Val155Met		fig|243277.254.peg.1772	VCC0996	VC1849	*ppiB*	Peptidyl-prolyl cis-trans isomerase PpiB (EC 5.2.1.8)
1	2034684	C	T	11518	367	0.99	Synon	ggc	ggT	576C>T	Gly192Gly		fig|243277.254.peg.1810	VCC0958	VC1887		hypothetical protein
1	2044594	T	C	13449	397	0.99	Synon	att	atC	51T>C	Ile17Ile		fig|243277.254.peg.1821	VCC0947	VC1898		Methyl-accepting chemotaxis sensor/transducer protein
1	2118347	A	G	13401	405	1.00	Nonsyn	aca	Gca	277A>G	Thr93Ala		fig|243277.254.peg.1889	VCC0880	VC1967		Methyl-accepting chemotaxis sensor/transducer protein
1	2338446	G	A	17006	496	1.00	Nonsyn	ccg	cTg	863C>T	Pro288Leu		fig|243277.254.peg.2098	VCC0672	VC2191	*FlgK*	Flagellar hook-associated protein FlgK
1	2352451	G	A	15398	478	1.00	Nonsyn	gcg	Acg	283G>A	Ala95Thr		fig|243277.254.peg.2115	VCC0655	VC2208	*FlgT*	Flagellar protein FlgT
1	2437098	G	A	17164	515	1.00	Nonsyn	gcc	Acc	871G>A	Ala291Thr		fig|243277.254.peg.2187	VCC0583	VC2281	*lysX_1/rimK*	Ribosomal protein S6--L-glutamate ligase
1	2437224	C	T	16081	514	0.99	Synon	gac	gaT	105C>T	Asp35Asp		fig|243277.254.peg.2188	VCC0582	VC2282		FIG003737: Predicted deacylase
1	2489368	G	A	19182	580	1.00	Nonsyn	ctc	Ttc	1237C>T	Leu413Phe		fig|243277.254.peg.2234	VCC0536	VC2338	*LacZ*	beta-galactosidase (EC 3.2.1.23)
1	2653262	A	G	23419	685	1.00	Synon	cta	ctG	543A>G	Leu181Leu		fig|243277.254.peg.2364	VCC0405	VC2472	*ygfZ*	tRNA-modifying protein YgfZ
1	2683691	G	T	17727	536	1.00	Synon	ggg	ggT	51G>T	Gly17Gly		fig|243277.254.peg.2387	VCC0382	VC2498		FIG023103: Predicted transmembrane protein
1	2787100	G	A	20014	583	1.00	Synon	ttc	ttT	672C>T	Phe224Phe		fig|243277.254.peg.2501	VCC0269	VC2618	*argD*	Succinylornithine transaminase (EC 2.6.1.81)
1	2839044	A	G	25320	726	0.99	Nonsyn	acg	Gcg	868A>G	Thr290Ala		fig|243277.254.peg.2550	VCC0220	VC2671	*mepA_2*	Na+-driven multidrug efflux pump
1	2843849	G	A	22711	696	1.00	Synon	acc	acT	51C>T	Thr17Thr		fig|243277.254.peg.2555	VCC0215	VC2676	*ftsN*	Cell division protein
2	25054	C	T	13277	412	1.00	Nonsyn	gct	gTt	1661C>T	Ala554Val		fig|243277.254.peg.2670	VCCA0321	VCA0020	*vasX*	Virulence factor VasX
2	42362	C	T	13931	405	1.00	Nonsyn	gac	Aac	682G>A	Asp228Asn		fig|243277.254.peg.2684	VCCA0335	VCA0034		hypothetical protein
2	71492	G	A	13937	419	1.00	Nonsyn	gaa	Aaa	622G>A	Glu208Lys		fig|243277.254.peg.2709	VCCA0360	VCA0064	*fecA/hutR*	TonB-dependent heme receptor HutR
2	111098	G	T	13560	405	0.99	Nonsyn	cgc	cTc	221G>T	Arg74Leu		fig|243277.254.peg.2746	VCCA0397	VCA0103		Putative sulfate permease
2	198672	T	C	12200	358	1.00	Nonsyn	tat	Cat	616T>C	Tyr206His		fig|243277.254.peg.2819	VCCA0470	VCA0180	*pepT*	Tripeptide aminopeptidase (EC 3.4.11.4)
2	260890	A	G	10984	329	0.99	Nonsyn	att	Gtt	523A>G	Ile175Val		fig|243277.254.peg.2875	VCCA0525	VCA0239	*czcR*	Two-component transcriptional response regulator VC_A0239, OmpR family
2	266710	G	A	11178	331	1.00	Synon	cgc	cgT	366C>T	Arg122Arg		fig|243277.254.peg.2882	VCCA0532	VCA0246	*ulaA/sgaT*	PTS system, ascorbate-specific IIC component / PTS system, ascorbate-specific IIB component (EC 2.7.1.194)
2	270399	T	G	12663	375	0.99	Nonsyn	atg	Ctg	1198A>C	Met400Leu		fig|243277.254.peg.2886	VCCA0536	VCA0250	*tam/amy1*	Glycosidase
2	273273	ACGC	AC	1050.6	47	1.00	Deletion	ccgcgt	ccGT	2142_2143delGC	Arg715fs	yes	fig|243277.254.peg.2889	VCCA0539	VCA0254		hypothetical protein
2	280759	C	T	11499	339	0.99	Nonsyn	gtc	Atc	163G>A	Val55Ile		fig|243277.254.peg.2896	VCCA0547	VCA0263		Acetyltransferase
2	285840	C	T	13060	397	0.99	Synon	acc	acT	810C>T	Thr270Thr		fig|243277.254.peg.2901	VCCA0552	VCA0268		Methyl-accepting chemotaxis sensor/transducer protein
2	303934	TACCAGAACCAGAACCAGAACCAGAACCAGAACCAGAACCAGA	TACCAGAACCAGAACCAGAACCAGAACCAGAACCAGAACCAGA	1333.3	46	0.84	Deletion	agaaccagaaccagaaccagaaccagaaccagaaccagaaccagaacc	ACCAGAACCAGAACCAGAACCAGAACCAGAACCAGAACCAGAA	1063_1080delCCAGAACCAGAACCAGAA	Pro355_Glu360del		fig|243277.254.peg.2917	VCCA0568	VCA0283		hypothetical protein
2	472782	G	C	9714.9	291	1.00	Nonsyn	acg	aGg	254C>G	Thr85Arg		fig|243277.254.peg.3171	VCCA0822	VCA0535		hypothetical protein
2	484944	A	G	10616	306	1.00	Nonsyn	atg	Gtg	667A>G	Met223Val		fig|243277.254.peg.3182	VCCA0833	VCA0546	*alx*	Integral membrane protein TerC
2	494298	A	G	10124	300	1.00	Nonsyn	gac	gGc	926A>G	Asp309Gly		fig|243277.254.peg.3192	VCCA0844	VCA0557		Extracellular solute-binding protein, family 3/GGDEF domain protein
2	518297	A	G	10338	303	1.00	Nonsyn	tcc	Ccc	763T>C	Ser255Pro		fig|243277.254.peg.3212	VCCA0864	VCA0578		SgrR, sugar-phosphate stress, transcriptional activator of SgrS small RNA
2	566650	G	A	11054	326	1.00	Nonsyn	gcg	gTg	626C>T	Ala209Val		fig|243277.254.peg.3260	VCCA0912	VCA0629		Putative ammonia monooxygenase
2	620722	G	A	11070	326	1.00	Nonsyn	cgc	cAc	647G>A	Arg216His		fig|243277.254.peg.3313	VCCA0965	VCA0684	*uhpC*	Possible sugar-phosphate sensor or MFS family transporter KOX_12270
2	884517	C	T	11992	371	1.00	Nonsyn	gcc	Acc	1207G>A	Ala403Thr		fig|243277.254.peg.3552	VCCA0133	VCA0931		Response regulator
2	889931	C	G	14051	409	1.00	Nonsyn	gcc	gGc	692C>G	Ala231Gly		fig|243277.254.peg.3557	VCCA0138			putative membrane protein
2	918423	CACAAAACAAAACAG	CACAAAACAG	8544.1	294	0.97	Deletion	ctctgttttgttttgtg	ctCTGTTTTGTGc	1177_1181delTGTTT	Cys397fs	yes	fig|243277.254.peg.3587	VCCA0168	VCA0972	*mdtH*	Uncharacterized MFS-type transporter
2	1037637	G	A	14690	439	0.99	Nonsyn	atg	atA	339G>A	Met113Ile		fig|243277.254.peg.3693	VCCA0275	VCA1084	*lapB*	Type I secretion system ATPase, LssB family LapB
2	1051656	C	T	14882	433	1.00	Synon	gag	gaA	786G>A	Glu262Glu		fig|243277.254.peg.3704	VCCA0286	VCA1095		Signal transduction histidine kinase CheA

Table S4. Variant analysis between QS-proficient and QS-deficient V. cholerae C6706 strains

Chr	Pos	Ref	Var	Score	Var_cov	Var_frac	Type	Ref_nt	Var_nt	Ref_nt_pos_change	Ref_aa_pos_change	Frameshift	Gene_ID	Locus_tag_QS+C6706	Locus_tag_QS-C6706	Locus_tag_N16961	Gene_name	Function
1	772970	G	A	12062	357	1.00	Nonsyn	ggc	Agc	997G>A	Gly333Ser		fig|948564.8.peg.691	IT766_03570	VCC1859	VC1021	*luxO*	Regulatory protein LuxO

Table S5: *V. cholerae* strains N16961, QS-deficient and QS-proficient C6706 have comparable fitness.

**Strain no.**	**Inserts**	**Area under the growth curves** (arb. units)	**Relative fitness**	***N***	***P*-values**
3479	C6706 QS-deficient	1406 ± 18.0	1.00	8	
1810	N16961	1346 ± 6.0	0.96	3	<0.0001
3802	C6706 QS-proficient	1450 ± 33.5	1.03	16	0.0018
